# Expression levels of NONO, a nuclear protein primarily involved in paraspeckles function, are associated with several deregulated molecular pathways and poor clinical outcome in multiple myeloma

**DOI:** 10.1007/s12672-022-00582-2

**Published:** 2022-11-11

**Authors:** Domenica Ronchetti, Vanessa Katia Favasuli, Ilaria Silvestris, Katia Todoerti, Federica Torricelli, Niccolò Bolli, Alessia Ciarrocchi, Elisa Taiana, Antonino Neri

**Affiliations:** 1 Hematology, Fondazione Cà Granda IRCCS Policlinico, 20122 Milan, Italy; 2 Department of Oncology and Hemato-oncology, University of Milan, 20122 Milan, Italy; 3 Laboratory of Translational Research, Azienda USL-IRCCS Reggio Emilia, 42123 Reggio Emilia, Italy; 4 Scientific Directorate, Azienda USL-IRCCS Reggio Emilia, 42123 Reggio Emilia, Italy; 5 Present Address: Department of Pathology and Laboratory Medicine, Fondazione IRCCS Istituto Nazionale dei Tumori, Milan, Italy

**Keywords:** NONO, Multiple myeloma, Therapeutic target, Warburg effect

## Abstract

**Purpose:**

The NONO protein belongs to the multifunctional family of proteins that can bind DNA, RNA and proteins. It is located in the nucleus of most mammalian cells and can affect almost every step of gene regulation. Dysregulation of NONO has been found in many types of cancer; however, data regarding its expression and relevance in Multiple Myeloma (MM) are virtually absent.

**Methods:**

We took advantage of a large cohort of MM patients enrolled in the Multiple Myeloma Research Foundation CoMMpass study to elucidate better the clinical and biological relevance of NONO expression in the context of the MM genomic landscape and transcriptome.

**Results:**

NONO is overexpressed in pathological samples compared to normal controls. In addition, higher NONO expression levels are significant independent prognostic markers of worse clinical outcome in MM. Our results indicate that NONO deregulation may play a pathogenetic role in MM by affecting cell cycle, DNA repair mechanisms, and influencing translation by regulating ribosome biogenesis and assembly. Furthermore, our data suggest NONO involvement in the metabolic reprogramming of glucose metabolism from respiration to aerobic glycolysis, a phenomenon known as the ‘Warburg Effect’ that supports rapid cancer cell growth, survival, and invasion.

**Conclusion:**

These findings strongly support the need of future investigations for the understanding of the mechanisms of deregulation and the biological role and activity of NONO in MM.

**Supplementary Information:**

The online version contains supplementary material available at 10.1007/s12672-022-00582-2.

## Introduction

Multiple Myeloma (MM) is a malignant proliferation of bone marrow (BM) plasma cells (PCs) characterized by different clinical course and a highly heterogeneous genetic background with both structural chromosomal alterations and specific genes mutations [1]. Clinically, despite the use of powerful and effective drugs, resistant clones arise upon the selective pressure of subsequent different lines of treatment and, notwithstanding the remarkable improvement in treatment and patient care, lead to the definition of MM as an incurable disease [2]. These data strongly support the need to identify novel prognostic biomarkers for MM.

In this context, great attention was devoted to long non-coding RNAs (lncRNAs), whose deregulated expression has been observed in many human cancers, including MM [3, 4], suggesting that lncRNAs may have an oncogenic or tumor-suppressive role in tumor development and progression [5, 6]. Among lncRNAs relevantly expressed in malignant PCs, we have recently focused on the nuclear paraspeckle assembly transcript 1 (NEAT1) demonstrating that its targeting impairs the DNA repair machinery and triggers anti-tumor activity in MM [7]. NEAT1 is an essential structural component of paraspeckles (PSs), which are dynamic and membraneless nuclear bodies that affect different cellular functions, including mRNA nuclear retention, micro-RNA processing, molecular sponge for RNA binding proteins, DNA damage repair (DDR) systems regulation, and stress response [8].

One of the essential proteins to prevent NEAT1 degradations is NONO (non-POU domain-containing octamer-binding protein) [9]. NONO belongs to the multifunctional DBHS (Drosophila behaviour/human splicing) family of proteins that can bind DNA, RNA and proteins [10, 11]. It is located within the nucleus of most mammalian cells and is primarily distributed in PSs. NONO, along with SFPQ, represents an essential PS protein; in detail, NONO heterodimerizes with SFPQ and binds NEAT1, thus allowing PSs assembling. Moreover, NONO is involved in almost every step of gene regulation, like RNA splicing, DNA unwinding, transcriptional regulation, and nuclear retention of defective RNA [11, 12].

Dysregulation of NONO has been found in many types of cancer, where NONO activities are well documented [12]; otherwise, data regarding its expression and relevance in MM are virtually absent. The only available data have recently included NONO in a five-genes RNA sequencing-based prognostic signature very reproducible in different MM databases [13].

Besides its essential role for PSs, which are crucial organelles for MM cells survival, NONO could play important roles independently from PSs, leading to the hypothesis that its targeting could trigger several and independent effects in MM cells. Hence, a better understanding of the roles of NONO in MM could make it therapeutically valuable and useful for the development of novel pharmacological approaches. Moreover, the identification of NONO as a target in MM could have a significant translational impact as a protein allows for possibilities of targeting much greater than RNA targets.

Here, we better elucidated the clinical and biological relevance of NONO expression in MM in the context of the genomic landscape and transcriptome, by taking advantage of a large cohort of MM patients enrolled in the Multiple Myeloma Research Foundation (MMRF) CoMMpass study.

## Materials and methods

Full details of quantitative real-time PCR, immunofluorescence, RNA FISH, and proteomic assays are provided in Supplementary Data.

### Multi-Omics data in CoMMpass study

Multi-omics data about bone marrow MM samples at baseline (BM_1) were freely accessible from MMRF CoMMpass Study (https://research.themmrf.org/) including more than 1000 MM patients from several worldwide sites and retrieved from the Interim Analysis 15a (MMRF_CoMMpass_IA15a, accessed on 16 October 2020). Details about molecular and clinical data of the CoMMpass cohort selected for the present study are described in Supplementary Data.

### Statistical and survival analyses

Wilcoxon rank-sum and Kruskal-Wallis tests were applied to assess differential expression patterns between two or multiple molecular groups. Dunn’s test was used for pairwise comparisons. *P*-values were corrected using Benjamini-Hochberg (BH) method, and adjusted *p*-values < 0.05 were considered significant. Survival analyses were performed using survival and survminer packages in R Bioconductor (version 4.0.0). The median cut-point value was used to stratify MM cases of CoMMpass cohort in high and low NONO expression groups.

### Differential expression analysis on CoMMpass MM cohort

A global dataset of 774 BM_1 MM cases was stratified according to RNA-seq NONO expression levels. Further steps of analysis were described in Supplementary Methods.

### Functional enrichment analysis on differentially expressed protein-coding genes

Gene Set Enrichment Analysis (GSEA) was performed on the pre-ranked differentially expressed (DE) protein-coding gene lists based on the fold change (FC) values by computing 1000 permutations and using default analysis conditions. Further details about GSEA analysis were reported in Supplementary Methods.

## Results and discussion

**Fig. 1:**
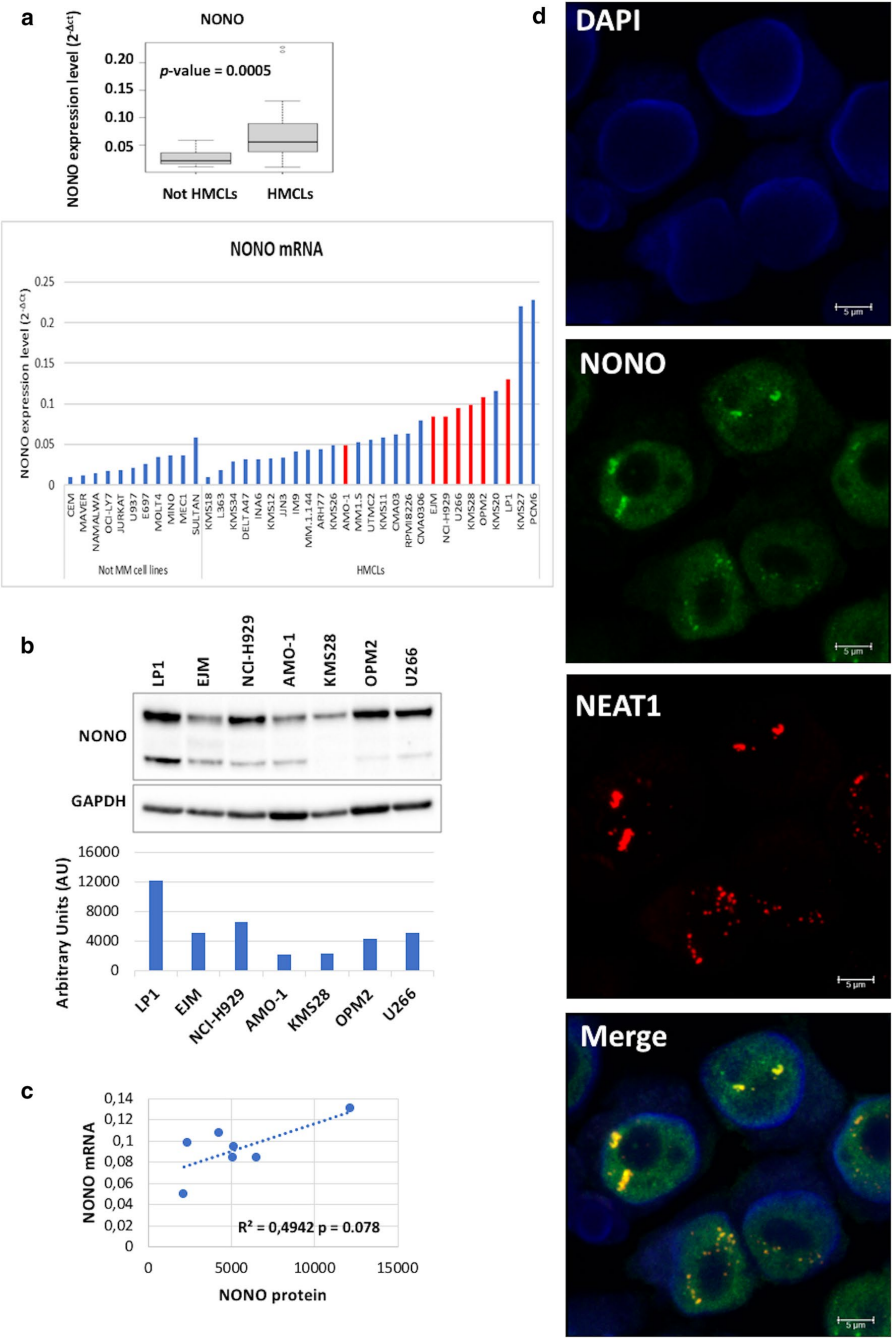
**a** NONO expression in hematological tumors. Boxplot of NONO mRNA expression in 27 MM, 5 lymphoma, and 6 leukemia cell lines based on the quantitative real-time PCR (qRT-PCR) approach described in Supplementary Data. The histogram details NONO expression in each cell line, including: one diffuse large B-cell lymphoma (OCILY7), two mantle cell lymphomas (MAVER and MINO), two Burkitt lymphomas (SULTAN and NAMALWA), one chronic lymphatic leukemia (MEC1), one acute myeloid leukemia (U937), three T-cell acute lymphoblastic leukemia (CEM, MOLT4, and JURKAT), and one B-cell acute lymphoblastic leukemia (697). **b** WB of NONO in indicated HMCLs. GAPDH protein expression was included for protein loading normalization. Densitometric analysis of immunoreactive bands is reported in the histogram below. **c** Pearson correlation between NONO mRNA and protein expression levels. **d** Confocal microscopy results of NEAT1 specific RNA-FISH and NONO IF in AMO-1 cells (scale bar 5 μm)

We investigated NONO expression levels in a panel of human MM cell lines (HMCLs) and other types of hematological tumors and found a significantly higher NONO expression levels in HMCLs (Fig. 1a). Furthermore, NONO expression was examined at the protein level (Fig. 1b) and we found a correlation between the expression of NONO at mRNA and protein level (Fig. 1c). NONO expression was also revealed by immunofluorescence (IF) analysis coupled with RNA-FISH to detect NEAT1; our results pointed out that NONO is mainly located in the nucleus of PCs, mostly associated with NEAT1 in PSs (Fig. 1d); however, a fraction of NONO does not colocalize with NEAT1, suggesting that NONO could play important roles independently from PSs.

Next, we investigated NONO expression levels in a proprietary, publicly available, RNA dataset profiled by microarrays that includes four healthy donors, 50 MM and 21 plasma cell leukemia (PCL) patients (GSE116294). We observed that NONO median expression level in PCL samples was significantly higher than in normal and MM groups, whereas no significant difference was found among MM patients and healthy donors (Supplementary Fig. S1a). These results were confirmed in a different dataset profiled by microarrays and including nine healthy donors (N), 170 MM, 24 primary and 12 secondary plasma cell leukemia, and 18 human myeloma cell lines (combined proprietary dataset GSE66293 and GSE47552 [14], Supplementary Fig. S1b), and also in a publicly available dataset (combined GSE159426 and GSE120795 [15]) profiled by RNA sequencing and including 11 healthy donors and 58 MM patients (Supplementary Fig. S1c). These findings prompted us to investigate the NONO expression pattern in a larger cohort of MM patients that could be more representative of the clinical and genomic heterogeneity of the disease. To this end, we evaluated BM PC expression levels from 774 MM patients included in the CoMMpass dataset (Supplementary Fig. S1d). In detail, NONO expression spanned a wide range of estimated values (38.5-991.8; median: 193.6) of transcripts per million (TPM). In addition, MM without chromosomal translocations or with MYC translocations showed NONO expression levels significantly lower than patients with other translocation, namely t(11;14), MAF translocations, t(4;14), or double translocations.

To assess NONO expression profiles in relation to major molecular aberrations in MM, we investigated 660 MM patients of the CoMMpass cohort for which expression, Non-Synonymous (NS) somatic mutations, and Copy Number Alterations (CNAs) data were available by RNA-sequencing (RNA-seq), Whole Exome Sequencing (WES) and next generation sequencing (NGS)-based FISH (FISH-WES), respectively (Supplementary Table S1). Significantly higher NONO expression levels were observed in MM patients carrying 1q-gain, t(4;14), del(1p), del(13q), t(11;14), MAF translocations, t(4;14), or the occurrence of NS somatic mutations in the *DIS3* gene, whereas lower expression levels were evidenced in hyperdiploid (HD) cases (Supplementary Fig. S2a), likely comprising the majority of the translocation-negative samples described above. No significant differences in NONO expression levels were observed in relation to del(17p)/TP53, t(6;14), or the occurrence of NS somatic mutations in *RAS*/*BRAF*, *TRAF3*, *TP53*, or *FAM46C* genes (Supplementary Fig. S2b). Finally, in apparent contradiction with above results (Supplementary Fig. S1d), MYC-translocated patients did not show any significantly lower NONO expression level as compared to its complementary set, as the group without MYC translocation includes the translocation-negative samples with low NONO expression level. Overall, our data indicated that many high-risk MM molecular subtypes are associated with higher NONO expression levels, likely reflecting the differential association of such lesions with hyperdiploid or IGH-translocated karyotypes.

**Fig. 2:**
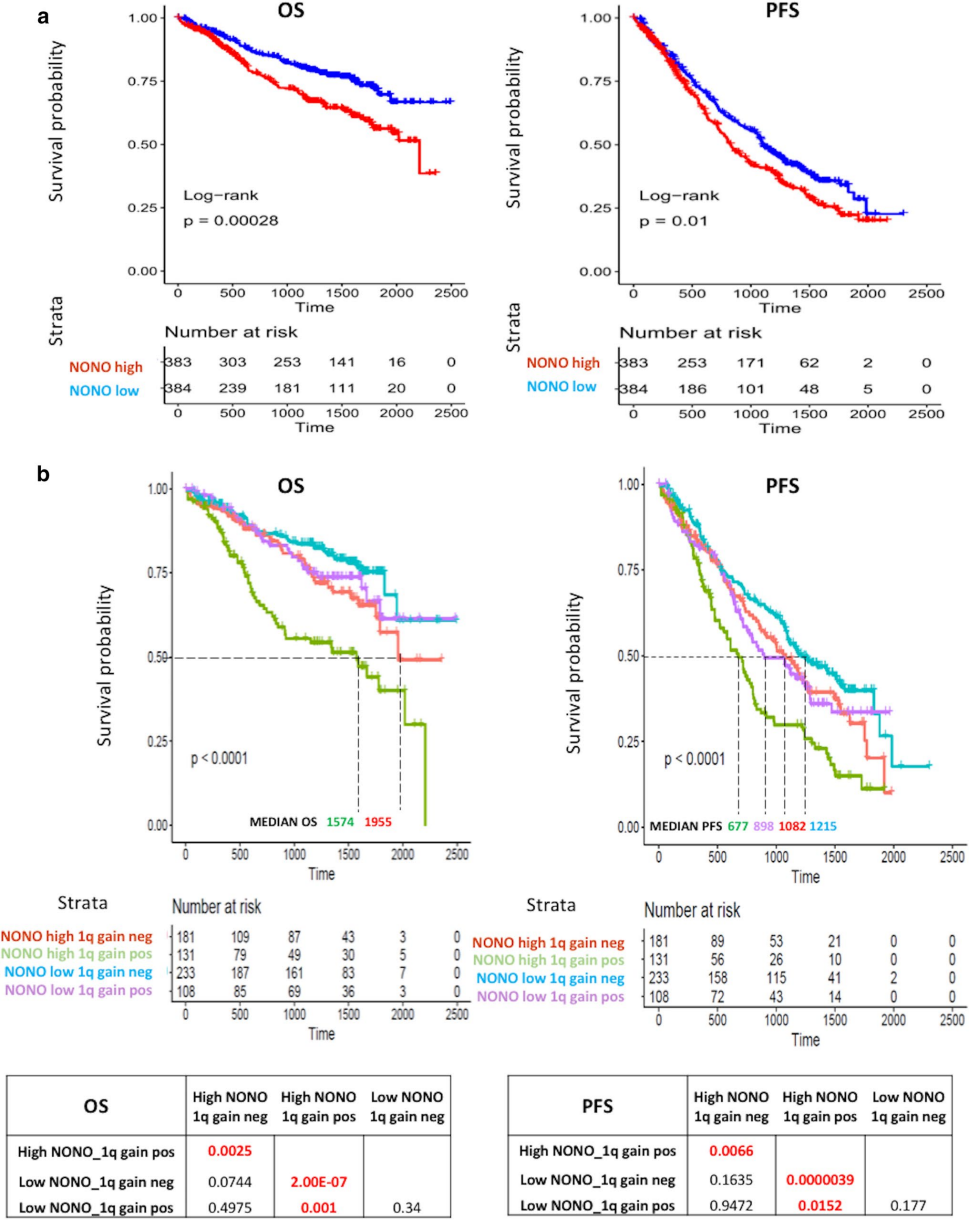
**a** Kaplan-Meier survival curves in the CoMMpass global dataset including 767 MM. MM cases were stratified in high and low NONO expression groups, accordingly to the median expression level across the dataset. Log-rank test p-value measuring the global difference between survival curves and number of samples at risk in each group across time are reported. **b** Kaplan-Meier survival curves in 653 MM with expression, molecular and clinical data available. Log-rank test p-value measuring the global difference between survival curves and the number of samples at risk in each group across time are reported. Log-rank test p-values of pairwise comparisons are also reported; significant adjusted p-values by BH correction (< 0.05) are in red-bold. Median OS and PFS is indicated for each curves

In order to investigate the relevance of NONO expression levels in clinical outcome, we considered 767 MM patients with available clinical data. High versus low expression groups were determined according to the median cut-off value for NONO expression level across the entire dataset. Interestingly, higher expression levels were associated with a poorer clinical outcome in terms of both overall survival (OS) and progression free survival (PFS) (Fig. 2a).

Based on this evidence and on the observed differential NONO expression patterns according to main molecular alterations (Supplementary Fig. S2), we evaluated the possible impact on survival of NONO expression levels in combination with the other significantly associated molecular variables, like t(11;14), t(4;14), MAF translocations, del(13q)/RB1, 1q-gain, del(1p)/CDKN2C, HD, or *DIS3* mutations. Notably, the combination of higher NONO expression level with the occurrence of 1q-gain was associated with the poorest survival rate in OS and PFS (Fig. 2b). Also, the combination of higher NONO expression level with the occurrence of del(13q)/RB1 was associated with a significantly shorter PFS (Supplementary Fig. S3). Conversely, no significant differences in OS or PFS were detected for NONO expression combined with t(11,14), t(4;14), MAF translocations, del(1p)/CDKN2C, HD, or *DIS3* mutations (Supplementary Fig. S3-S6).

**Fig. 3:**
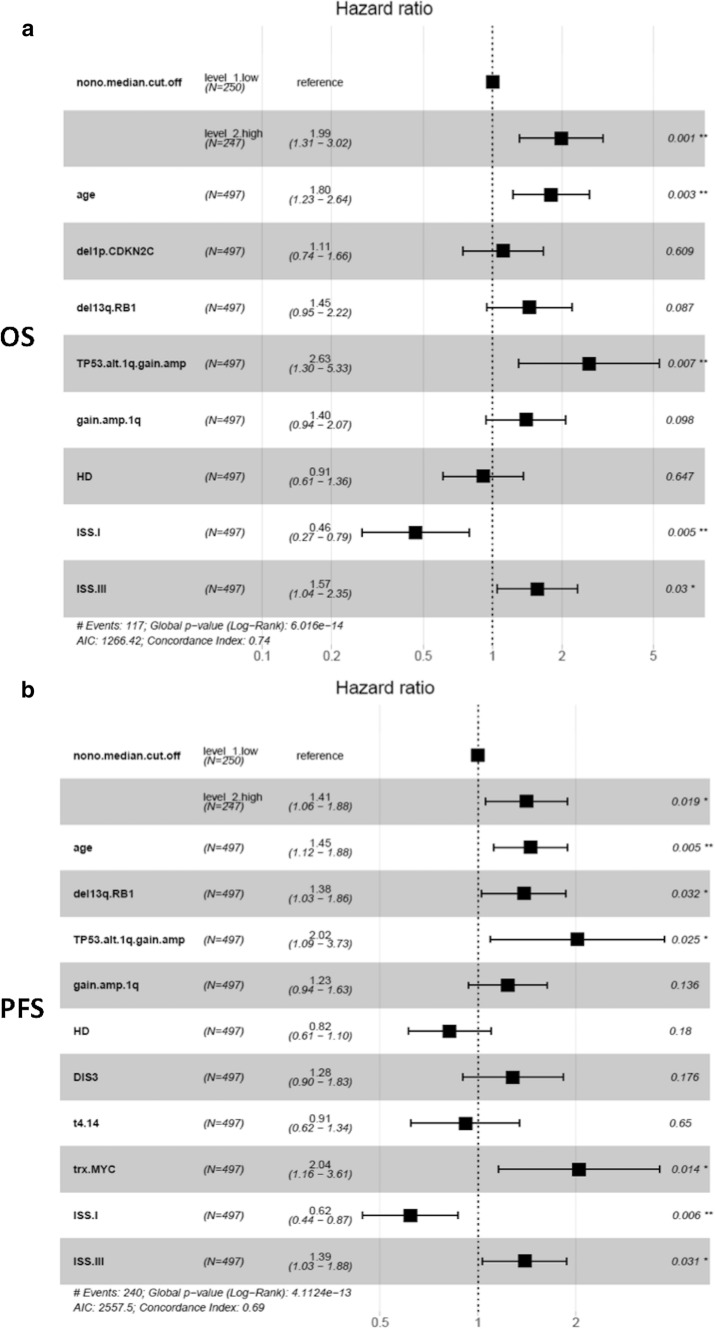
Forest plot of cox regression multivariate analysis considering all features with adjusted p-value < 0.05 in univariate analysis with regards to OS (**a**) and PFS (**b**) in 497 BM-1 MM cases. Hazard Ratio, 95% Confidence Interval and Log-rank p-value are indicated in the plot for each variable. Significant p-value: * ≤ 0.05; ** ≤ 0.01; *** ≤ 0.001; **** ≤ 0.0001

To verify if high NONO expression levels may represent an independent variable in predicting OS and PFS, we tested high NONO expression condition and other main molecular or clinical features by Cox regression univariate analysis in 497 MM samples for which all information were available. Concerning OS, a significantly higher risk of death was observed for cases with higher NONO expression level (Hazard Ratio, HR = 2.4, 95% CI 1.7–3.5, BH adj. p-value = 0.00002), together with older age (equal or over 65 years), ISS stage III, and distinct molecular variables such as del(13q)/RB1, del(1p)/CDKN2C, and 1q gain/amplification alone or in combination with TP53 alterations; conversely, ISS stage I and HD cases showed a 69% and 36% death risk reduction, respectively (Supplementary Fig. S7a). Moreover, when all significant variables were tested in multivariate analysis, higher NONO expression level retained significance (Fig. 3a). With regards to PFS, higher NONO expression level was associated with a significantly higher risk of disease progression (Hazard Ratio, HR = 1.7, 95% CI 1.3–2.2, BH adj. p-value = 0.0004), as well as older age, ISS stage III, and distinct molecular variables such as del(13q)/RB1, 1q gain/amplification alone or in combination with TP53 alterations, MYC translocation, t(4;14) translocation, and the presence of *DIS3* mutations; on the contrary, ISS stage I and HD cases showed a 56% and 34% progression risk reduction, respectively (Supplementary Fig. S7b). Moreover, when all significant variables were tested in multivariate analysis, higher NONO expression level retained significance (Fig. 3b). Overall, these data from the CoMMpass cohort demonstrated the clinical impact of NONO expression levels in MM, which is independent from known genetic prognostic factors.

**Fig. 4:**
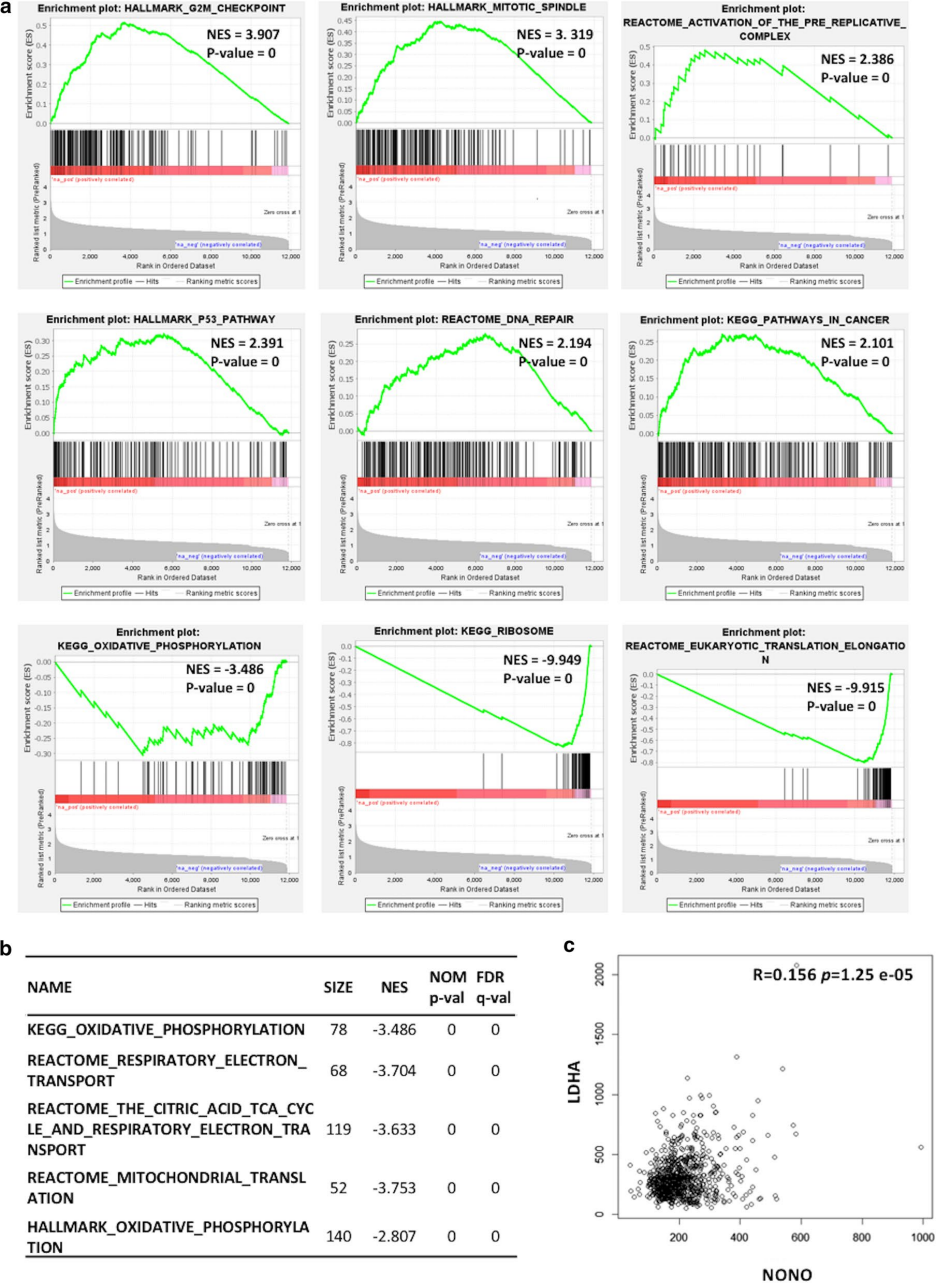
**a** Enrichment plots of selected GSEA gene sets significantly modulated in NONO IV versus I quartile. Normalized Enrichment Score (NES) and nominal p-value are reported for each plot. **b** Selected GSEA gene sets related to oxidative phosphorylation significantly downregulated in NONO IV versus I quartile. **c** NONO and LDHA Spearman’s correlation in the CoMMpass global dataset including 774 MM cases

These observations prompted us to gain a better understanding of the roles of NONO in MM, based on the notion that NONO is involved in many different PS related or independent processes, which suggests that its targeting could trigger several and independent effects in MM cells. To gain insights into the possible role of deregulated NONO in MM, we investigated the transcriptional pattern associated with NONO expression levels in 774 MM samples included in the RNA-seq CoMMpass dataset, stratifying them according to NONO expression levels. Therefore, global expression profiles of annotated protein-coding genes (18.818 annotated protein coding genes by Ensembl Biomart) were compared in the two extreme quartiles of NONO expression. A list of 11.872 differentially expressed protein-coding genes was obtained by limma analysis at a low stringency level (FDR 10% cut-off) (Supplementary Table S2), the majority of which resulted up-regulated (83%) in patients with higher NONO expression (Supplementary Fig. S8). Interestingly, the functional annotation analysis of this signature, aimed at identifying highly significant represented categories, revealed the enrichment in biological processes like proteasome-mediated protein catabolism, mitotic spindle organization, ncRNA metabolic process, and double-strand break repair (Supplementary Fig. S9). In addition, to identify which molecular pathways could be modulated in relation to NONO expression, we performed a Gene Set Enrichment Analysis (GSEA) on the list of DE coding genes ranked based on FC values, using different gene set collections. More in details, in MM cases with higher NONO expression levels we found the positive modulation of pathways related to mitosis and cell cycle checkpoint, DNA repair processes, DNA replication, TP53 signaling, and pathways in cancer (Fig. 4a and Supplementary Table S3). On the contrary, genes involved in RNA and protein metabolism, like those coding for ribosome structural constituents, were down-regulated in higher NONO expression cases (Fig. 4a). Notably, in MM patients with higher NONO expression levels we found the negative modulation of gene sets related to oxidative phosphorylation and Krebs Cycle (Fig. 4b and Supplementary Table S4). Furthermore, we detected a significant positive correlation between the expression levels of NONO and LDHA, which is a key glycolytic enzyme (Fig. 4c). As already described in hepatocellular carcinoma [16], our data suggest NONO involvement in the metabolic reprogramming of glucose metabolism from respiration to aerobic glycolysis, a phenomenon known as the ‘Warburg Effect’ that supports rapid cancer cell growth, survival, and invasion [17].

In conclusion, NONO expression levels are significant prognostic markers of clinical outcome in MM in multivariate analyses. Moreover, our results indicate that NONO deregulation may play a pathogenetic role in MM by affecting cell cycle, DNA repair mechanisms, and influencing translation by regulating ribosome biogenesis and assembly. Furthermore, our data suggest NONO involvement in the metabolic switch of tumor PCs. Taken together, these findings strongly support the need of future investigations for the understanding of the mechanisms of deregulation and the biological role and activity of NONO in MM.

## Electronic supplementary material

Supplementary Material 1

## Data Availability

Not applicable.

## Abbreviations

NONO: Non-POU Domain Containing Octamer Binding
MM: Multiple Myeloma
PC: Plasma cell
PCL: Plasma cell leukemia
PS: Paraspeckle
NEAT1: Nuclear paraspeckle assembly transcript 1
HR: Hazard Ratio
CI: Confidence interval
BH: Benjamini-Hochberg
DDR: DNA damage repair
HMCL: Human multiple myeloma cell line
SFPQ: Splicing Factor Proline and Glutamine Rich
OS: Overall survival
PFS: Progression free survival
qRT-PCR: Quantitative Reverse Transcription-polymerase Chain Reaction
HD: Hyperdiploid
ISS: International staging system
RPA32: Replication protein A 32
lncRNA: Long non-coding RNA
TP53: Tumor Protein p53
DE: Differentially expressed
DIS3: Exosome endoribonuclease and 3-prime-5-prime exoribonuclease
LDHA: Lactate dehydrogenase A
