# Supplementary material for: Expression levels of NONO, a nuclear protein primarily involved in paraspeckles function, are associated with several deregulated molecular pathways and poor clinical outcome in multiple myeloma
Source: Discov Oncol. 2022 Nov 11;13:124. doi: 10.1007/s12672-022-00582-2 (PMC9652193; doi:10.1007/s12672-022-00582-2)
Supplement: Supplementary file 1 — Supplementary Material 1 [file 12672_2022_582_MOESM1_ESM.pdf]

Expression levels of NONO, a nuclear protein primarily involved in paraspeckles function, are associated with several deregulated molecular pathways and poor clinical outcome in multiple myeloma

Domenica Ronchetti[1,2], Vanessa Katia Favasuli[1,2], Ilaria Silvestris[1,2], Katia Todoerti[1#], Federica Torricelli[3,] Niccolò Bolli[1,2], Alessia Ciarrocchi[3], Elisa Taiana[1,°*], Antonino Neri[4,°]

[1]Hematology, Fondazione Cà Granda IRCCS Policlinico, 20122 Milan, Italy

[2]Department of Oncology and Hemato-oncology, University of Milan, Italy 20122 Milan,

[3]Laboratory of Translational Research, Azienda USL-IRCCS Reggio Emilia, 42123 Reggio Emilia, Italy

[4]Scientific Directorate, Azienda USL-IRCCS Reggio Emilia, 42123 Reggio Emilia, Italy

° ET and AN contributed equally as co-senior author.

*Author to whom correspondence should be addressed:

elisa.taiana@policlinico.mi.it

#Present address:

KT: Department of Pathology and Laboratory Medicine, Fondazione IRCCS Istituto Nazionale dei Tumori, Milan, Italy

SUPPLEMENTARY METHODS

Reverse transcription and quantitative PCR

Total RNA was extracted using TRIzol® Reagent (Invitrogen, Life Technologies) according to the manufacturer's instructions. The purity and concentration of total RNA were determined by the NanoDrop 1000 spectrophotometer (Thermo Fisher Scientific). The ratios of absorption (260 nm/280 nm) of all samples were between 1.8 and 2.0. cDNA was synthesized from 500 ng of total RNA with random primers using the High Capacity cDNA Reverse Transcriptase Kit (Invitrogen) according to the manufacturer`s instructions. To evaluate the expression levels of NONO, RT-PCR was performed using SYBR green PCR Master Mix (Applied Biosystems) after optimization of the primer conditions. 10 ng of reverse-transcribed RNAs were mixed with 300 nM of specific forward and reverse primers in a final volume of 10 µl. RT-PCR was performed on an Applied Biosystems StepOnePlus Real-Time PCR system for 40 cycles. Data were analyzed using the $2^{-\Delta Ct}$ method or the $2^{-\Delta\Delta Ct}$ method to measure the relative changes in each gene's expression compared with GAPDH expression. To determine RNA levels by qPCR, the following primers were used:

**Primer**	**Sequence (5' - 3')**
GAPDH_Fw	5' - ACAGTCAGCCGCATCTTCTT - 3'
GAPDH_Rw	5' - AATGAAGGGGTCATTGATGG - 3'
NONO_Fw	5' - AACATCAAGGAGGCTCGTGA - 3'
NONO_Rw	5' - GTCGCCGCATCATTTCTTCT - 3'

Immunofluorescence

0.1 x 10^6 cells were harvested, centrifuged onto glass slides (Cytospin 4, Thermo Scientific), then fixed in 4% paraformaldehyde in PBS1X for 12 min at 22°C, followed by three 5-min washes in PBS. Cells were permeabilized (0.1% Triton X-100 in PBS, 15-min), washed in PBS (3X, 5 min each), blocked 1 h at 22°C with 1.5% BSA in PBS, and then incubated 1 hour at 4°C with specific primary antibodies (1:200).

Thereafter, slides incubated with primary antibody specific for NONO were washed three times in PBS and mounted under coverslips with DAPI-containing Vectashield (Vector Laboratories). Slides incubated with primary antibody specific for NONO. Images were acquired by Leica TCS SP8 confocal laser scanning microscope (DMi8); acquisitions were performed with 40X and 63X immersion oil objectives. Conversion of imaged z-stacks into average intensity projections was processed by Leica Microsystem software (Leica Application Suite X - LAS X).

**Protein**	**Ab Cod.**		**Source**	**Application**
NONO (nmt55 / p54nrb)	ab208404	Abcam	Alexa Fluor 488-conjugated	IF

RNA FISH

RNA FISH was performed to evaluate the expression of NEAT1. We used the Stellaris RNA FISH kit (Biosearch Technologies), according to the manufacturer's instructions. For NEAT1 detection, we took advantage of a commercial set of Quasar® 570-labeled oligos (Stellaris, Biosearch Technologies) able to bind the 5' end of NEAT1 transcript. Images were acquired by Leica TCS SP8 confocal laser scanning microscope (DMi8); acquisitions were performed with 40X and 63X oil immersion objectives. Conversion of imaged z-stacks into average intensity projections was processed by Leica Microsystem software (Leica Application Suite X - LAS X).

Proteomic assays

Cells were homogenized in lysis buffer M-PER® Mammalian Protein Extraction Reagent (Thermo Scientific, Italy) and Halt Protease and Phosphatase inhibitor cocktail, EDTA-free, 100X, (Thermo Scientific). Whole cell lysates (40 µg per cell line) from cell lines were separated using Bolt™ 4-12% Bis-Tris Plus Acriliamide Gels (Invitrogen), electro-transferred onto nitrocellulose membranes (Bio-Rad, Hercules, CA, USA), and immunoblotted with specific primary antibody (listed below). Membranes were washed three times in PBST solution and then incubated with a secondary antibody conjugated with horseradish peroxidase (HRP) in BSA 2% - PBST for 2 hours at RT. Chemiluminescence was developed using Clarity ECL Western Blot Substrate Kit (BIO-RAD) and signal intensity was detected by the use of ChemiDoc MP System (Bio-Rad). The experiments were repeated at least three times.

**Protein**	**Antibody**		**Source**	**Application**
NONO (nmt55 / p54nrb)	ab70335	Abcam	Rabbit pAb	WB
GAPDH	sc-32233	Santa Cruz Biotechnology	Mouse mAb	WB
Anti-mouse IgG	#7076	Cell Signaling	HRP-linked	WB
Anti-rabbit IgG	sc-2004	Santa Cruz Biotechnology	HRP-linked	WB

Multi-Omics Data in CoMMpass Study

Multi-omics data about bone marrow MM samples at baseline (BM_1) were freely available from MMRF CoMMpass Study (https://research.themmrf.org/) and obtained from the Interim Analysis 15a (MMRF_CoMMpass_IA15a).

Transcript per Million (TPM) reads values of the NONO transcript were retrieved using Salmon gene expression quantification data (MMRF_CoMMpass_IA15a_E74GTF_Salmon_V7.2_Filtered_Gene_TPM) in 774 BM_1 MM patients.

Clinical data regarding Overall Survival (OS) and Progression free Survival (PFS) were considered in 767 MM patients for which both RNA-seq expression and survival data were available.

Non-synonymous (NS) somatic mutation variants and counts data were obtained from whole exome sequencing (WES) analyses, main IgH translocations were inferred from RNA-seq spike expression estimates of known target genes and Copy Number Alteration (CNA) data were retrieved by means of Next generation Sequencing (NGS)-based fluorescence in situ hybridization (FISH) [1] in 497 MM cases for which all data were available [2].

The presence of a specific CNA was considered when occurring in at least one of the investigated cytoband at a 20 percent cut-off for each considered chromosomal aberration, as previously reported [2].

Survival analysis

Survival analyses were performed using survival [3-4] and survminer [5] packages in R Bioconductor (version 4.0.0). Kaplan-Meier analysis was applied on OS and PFS data in NONO high/low expression groups according to median NONO expression level, using survminer package. Log-Rank test p-value was calculated to measure the global difference between survival curves. Cox proportional hazards model was applied as univariate analysis on single molecular variables, age and International Staging System (ISS) groups in relation to OS data in 497 MM cases for which all information were accessible. Cox regression multivariate analysis was applied on all significant features after BH correction. Forest plot was used to summarize Cox Proportional Hazard Model.

Differential expression analysis

Global expression profiles of 18.818 protein-coding genes annotated by Ensembl Biomart were compared in IV versus I NONO quartile from the entire 774 MM RNA-seq dataset (194 MM cases in each group). Differentially expressed protein coding genes were selected by limma analysis, as previously described [2], at a False Discovery Rate (FDR) < 10%.

Functional annotation enrichment analysis

Gene Set Enrichment Analysis (GSEA version 4.1.0) [6] was performed under default conditions on pre-ranked protein coding gene list based on fold-change values. Significant gene sets of Hallmark, Reactome, and KEGG were selected on the base of nominal p-value <0.05. Normalized Enrichment Score (NES) and False Discovery Rate (FDR) q-value are also reported.

1. Miller, C. et al. A comparison of clinical FISH and sequencing based FISH estimates in multiple myeloma: An MMRF CoMMpass analysis. In: Hematology TASo, editor. The American Society of Hematology; 2016: Blood; 2016. p. 374.
2. Todoerti K, Ronchetti D, Favasuli V, Maura F, Morabito F, Bolli N, Taiana E, Neri A. DIS3 mutations in multiple myeloma impact the transcriptional signature and clinical outcome. Haematologica. 2021.
3. Therneau T (2021). A Package for Survival Analysis in R_. R package version 3.2-11, <URL: https://CRAN.R-project.org/package=survival>.
4. Terry M. Therneau, Patricia M. Grambsch (2000). _Modeling Survival Data: Extending the Cox Model_. Springer, New York. ISBN 0-387-98784-3.
5. Alboukadel Kassambara, Marcin Kosinski and Przemyslaw Biecek (2021). survminer: Drawing Survival Curves using 'ggplot2'. R package version 0.4.9. https://CRAN.R-project.org/package=survminer
6. Subramanian A, Tamayo P, Mootha VK, et al. Gene set enrichment analysis: a knowledge-based approach for interpreting genome-wide expression profiles. PNAS. 2005; 102(43):15545-50.

a

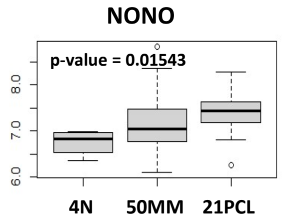

	N	MM
MM	0.061	
PCL	**0.0060**	**0.0144**

b

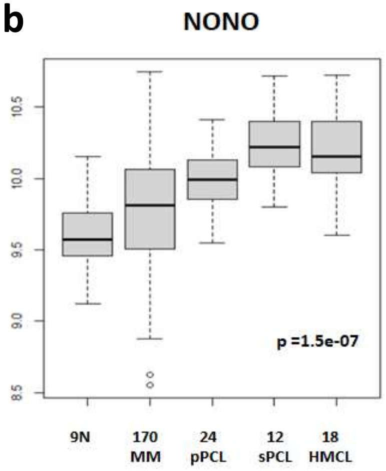

	N	MM	pPCL	sPCL
MM	0.062			
pPCL	**0.0032**	**0.006**		
sPCL	**0.0001**	**0.0000**	**0.0338**	
HMCL	**0.0001**	**0.0000**	0.0716	0.30

c

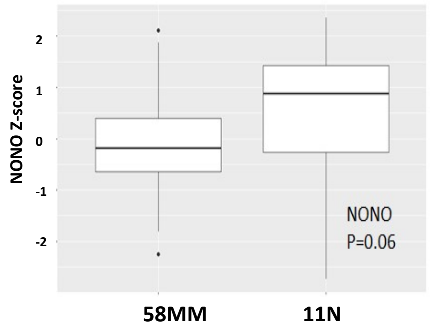

d

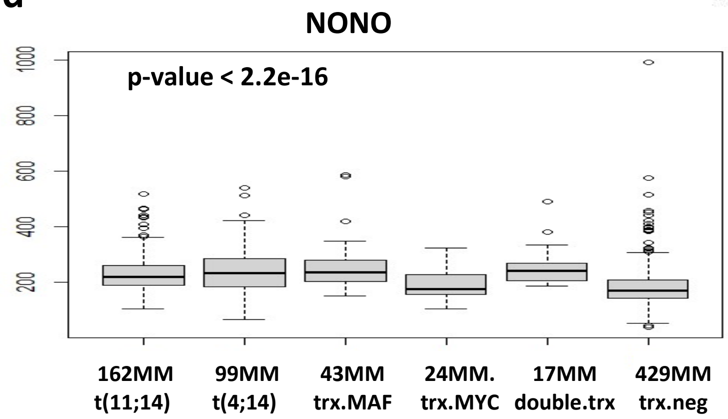

	t(11;14)	t(4;14)	Trx.MAF	Trx.MYC	Double trx
t(4;14)	0.3664				
Trx.MAF	0.1504	0.2316			
Trx.MYC	**0.0004***	**0.0003***	**0.0002***		
Double trx	0.1286	0.1751	0.3488	**0.0006***	
Trx neg	**0.0000***	**0.0000***	**0.0000***	**0.0000**	**0.0000***

Supplementary Figure S1: a) Box plots of NONO expression level in healthy donors (N) and PC dyscrasias (proprietary dataset, GSE116294). Total RNA samples from highly purified bone marrow CD138+ plasma cells were profiled by Gene 2.0 ST array. **b)** Box plots of NONO expression level in healthy donors (N), MM, primary and secondary plasma cell leukemia (pPCL and sPCL), and human myeloma cell lines (HMCL) (GSE66293 and GSE47552). Total RNA samples from highly purified bone marrow CD138+ plasma cells were profiled by Gene 1.0 ST array. **c)** Box plots of NONO expression levels in healthy donors (N) and MM patients (GSE159426 and GSE120795). CD138+ enriched bone marrow cells underwent RNA-sequencing analyses; after RPKM normalization, the two dataset were combined and the z-score was calculated for each transcript **d)** Box plot of NONO expression level in main IgH translocation groups in 774 cases of the CoMMpass cohort. Kruskal-Wallis test was applied to assess differences in expression levels between groups. The significant pairwise comparison performed by the Dunn's test are marked red-bold in the table.

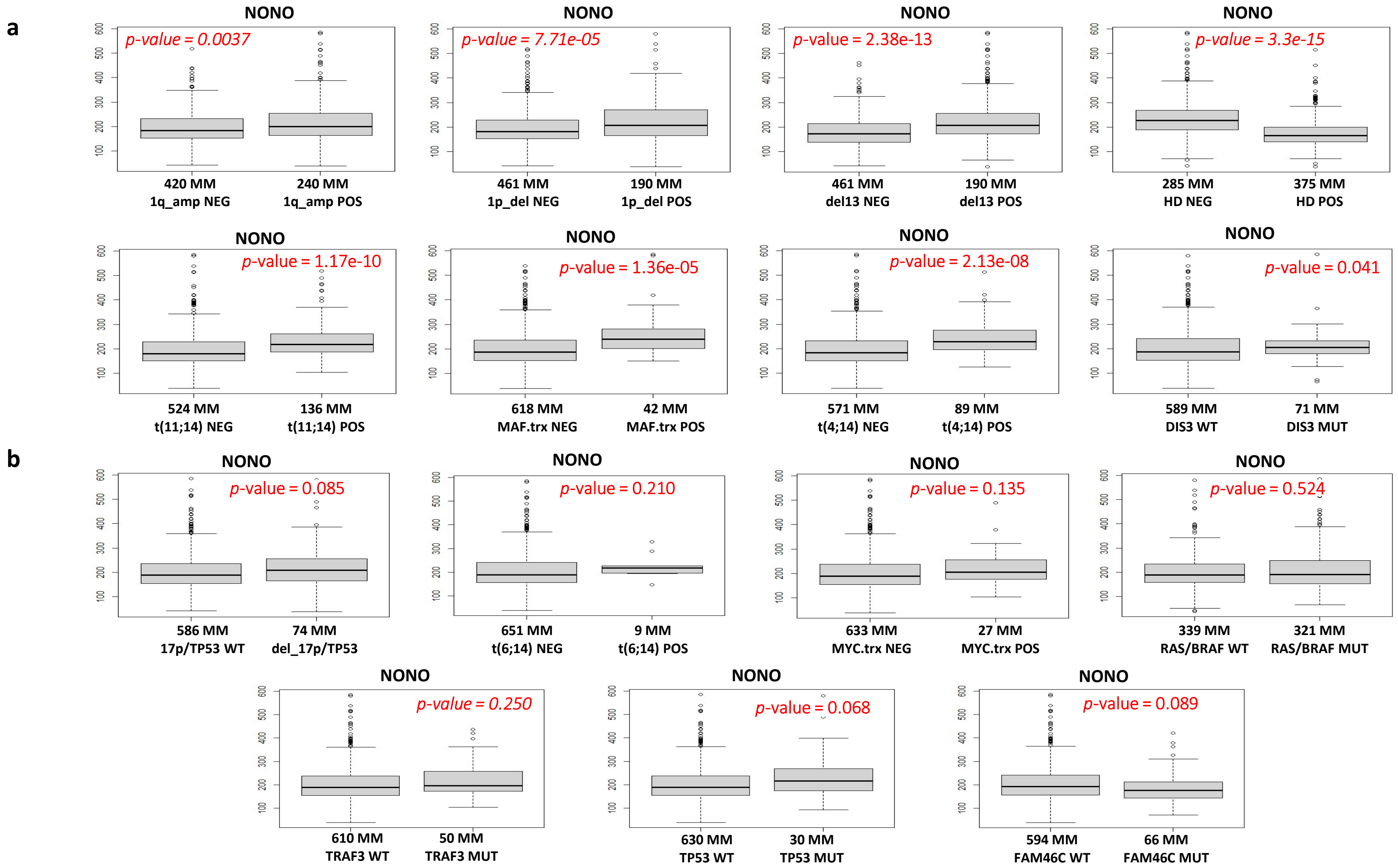

Supplementary Figure S2 (a) Significant differences in NONO expression level as shown by box plots of 660 MM cases stratified according to the presence of 1q-gain, t(4;14), del(1p), del(13q), hyperdiploidy (HD), t(11;14), MAF translocations, t(4;14), or the occurrence of NS somatic mutations in DIS3 gene. **(b)** Not-significant differences in NONO expression level as shown by box plots in NONO expression level in 660 MM cases stratified according to the presence of del(17p)/TP53, t(6;14), MYC translocations, or the occurrence of NS somatic mutations in RAS/BRAF, TRAF3, TP53, or FAM46C genes. For each plot, differential expression was tested by Wilcoxon rank-sum test with continuity correction. P-values were corrected by BH adjustment.

NONO expression level and del13

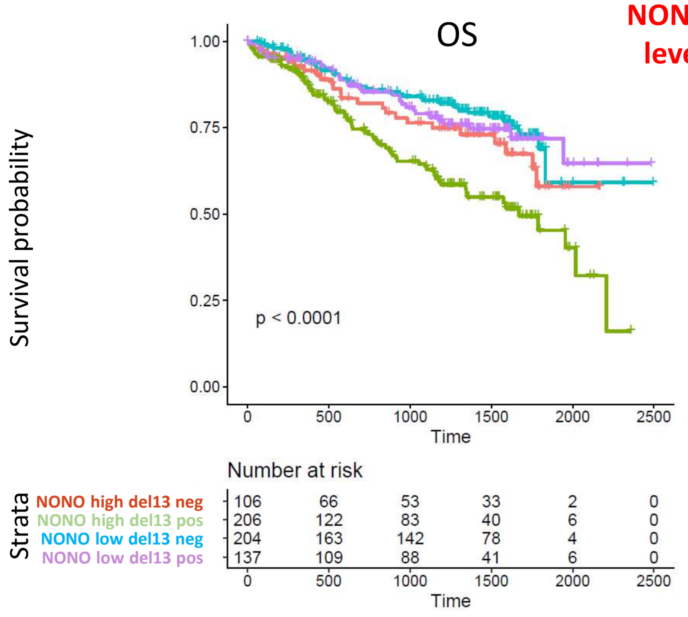

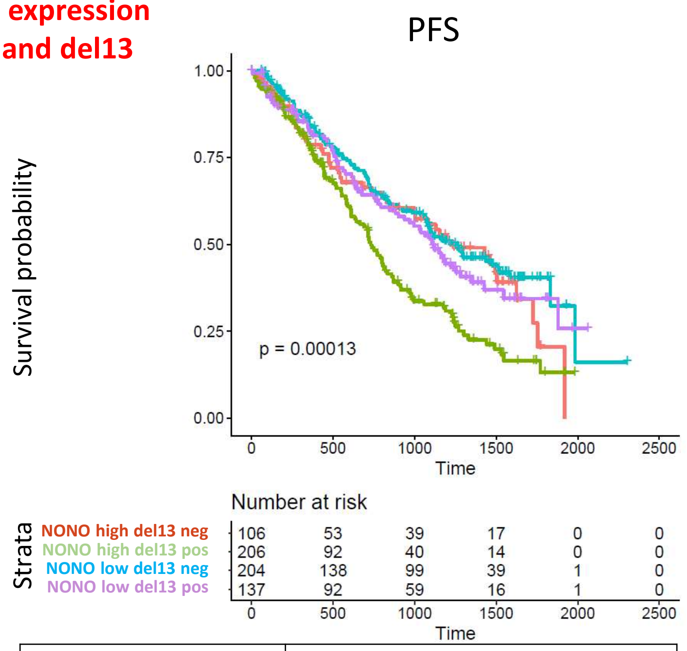

OS	High NONO del13 neg	High NONO del13 pos	Low NONO del13 neg
High NONO_del13 pos	0.0574		
Low NONO_del13 neg	0.2928	**3.00E-05**	
Low NONO_del13 pos	0.5261	**0.0011**	0.6315

PFS	High NONO del13 neg	High NONO del13 pos	Low NONO del13 neg
High NONO_del13 pos	**0.019**		
Low NONO_del13 neg	0.456	**6.8E-05**	
Low NONO_del13 pos	0.899	**0.010**	0.3456

NONO expression level and del1p

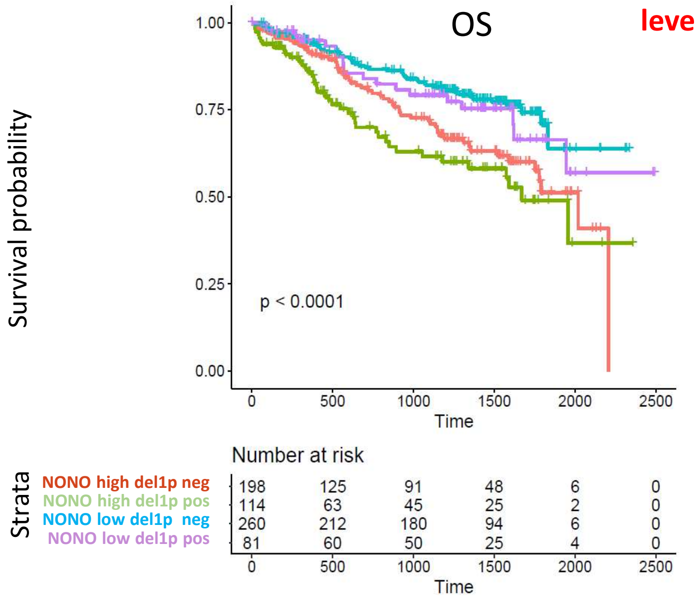

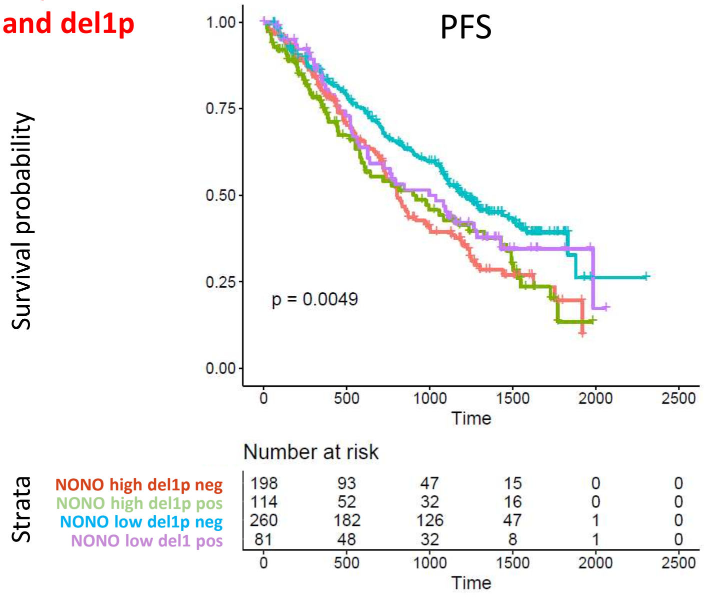

OS	High NONO del1p neg	High NONO del1p pos	Low NONO del1p neg
High NONO_del1p pos	0.19203		
Low NONO_del1p neg	**0.00429**	**0.00019**	
Low NONO_del1p pos	0.1431	**0.03193**	0.532

PFS	High NONO del1p neg	High NONO del1p pos	Low NONO del1p neg
High NONO_del1p pos	0.963		
Low NONO_del1p neg	**0.01**	**0.021**	
Low NONO_del1p pos	0.374	0.374	0.374

Supplementary Figure S3: Kaplan-Meier survival curves in 653 MM with expression, molecular and clinical data available. Log-rank test p-value measuring the global difference between survival curves and number of samples at risk in each group across time are reported. Log-rank test p-values of pairwise comparisons are also reported. Significant adjusted p-values by BH correction (< 0.05) are in red-bold.

NONO expression level and HD

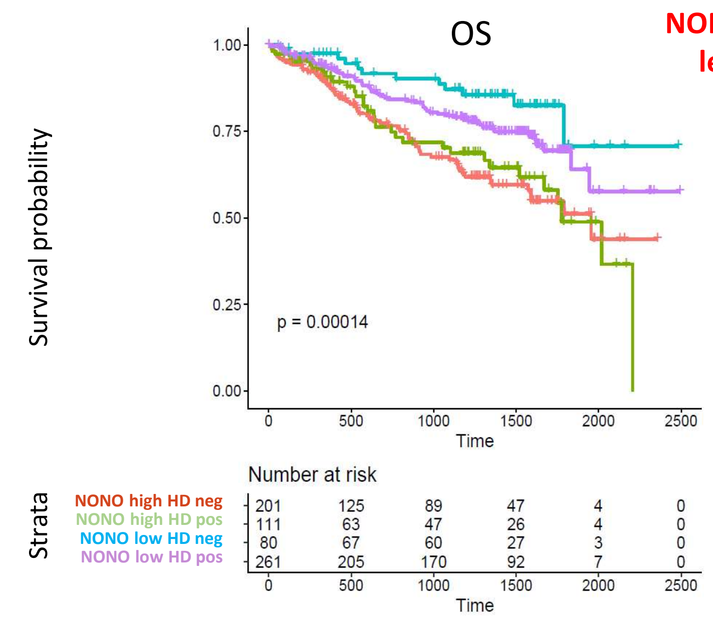

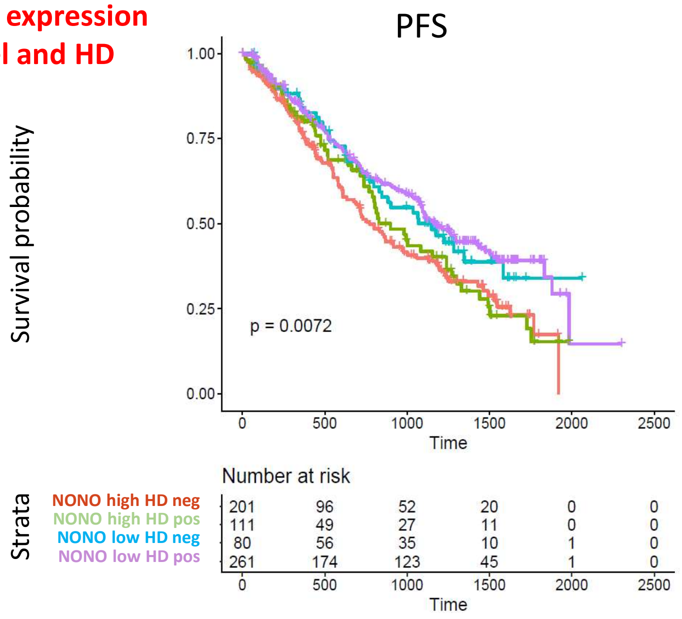

OS	High NONO HD neg	High NONO HD n pos	Low NONO HD neg
High NONO_HD pos	0.696		
Low NONO_HD neg	**0.002**	**0.0053**	
Low NONO_HD pos	**0.0038**	**0.0381**	0.126

PFS	High NONO HD neg	High NONO HD n pos	Low NONO HD neg
High NONO_HD pos	0.715		
Low NONO_HD neg	0.146	0.324	
Low NONO_HD pos	**0.009**	0.088	0.715

NONO expression level and t(11;14)

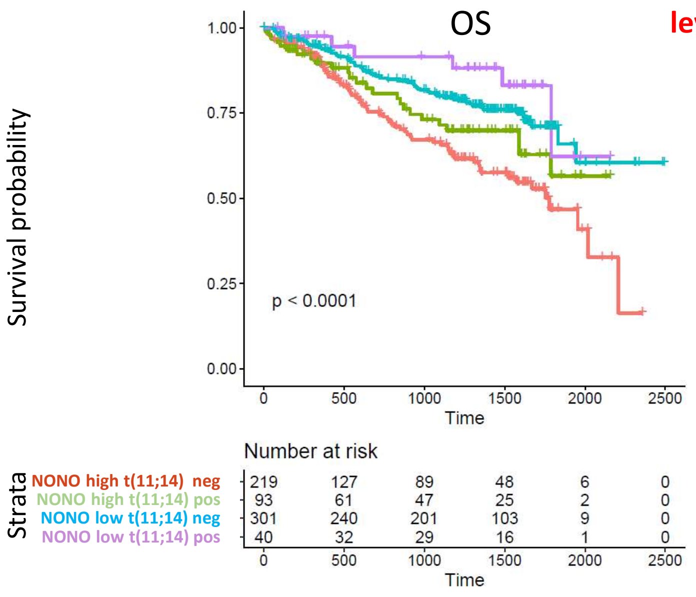

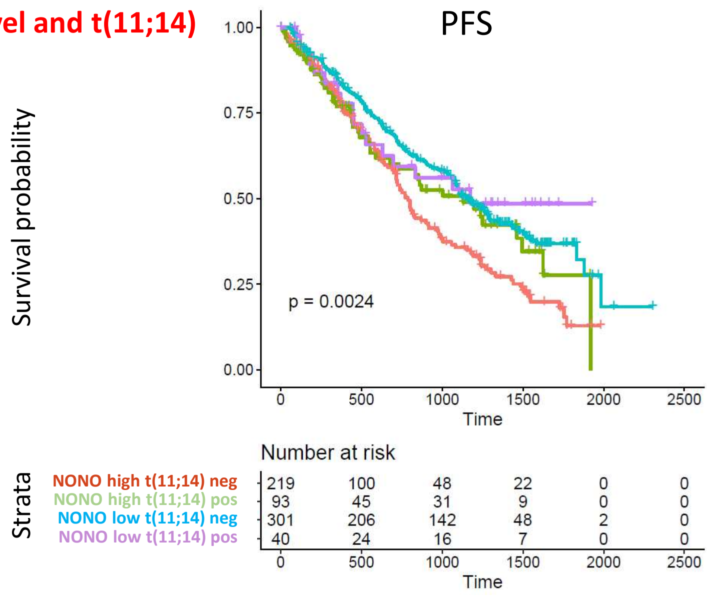

OS	High NONO t(11;14) neg	High NONO t(11;14) pos	Low NONO t(11;14) neg
High NONO_t(11;14) pos	0.208		
Low NONO_t(11;14) neg	**0.00015**	0.175	
Low NONO_t(11;14) pos	**0.0143**	0.154	0.280

PFS	High NONO t(11;14) neg	High NONO t(11;14) pos	Low NONO t(11;14) neg
High NONO_t(11;14) pos	0.332		
Low NONO_t(11;14) neg	**0.0011**	0.429	
Low NONO_t(11;14) pos	0.136	0.429	0.735

Supplementary Figure S4: Kaplan-Meier survival curves in 653 MM with expression, molecular and clinical data available. Log-rank test p-value measuring the global difference between survival curves and number of samples at risk in each group across time are reported. Log-rank test p-values of pairwise comparisons are also reported. Significant adjusted p-values by BH correction (< 0.05) are in red-bold.

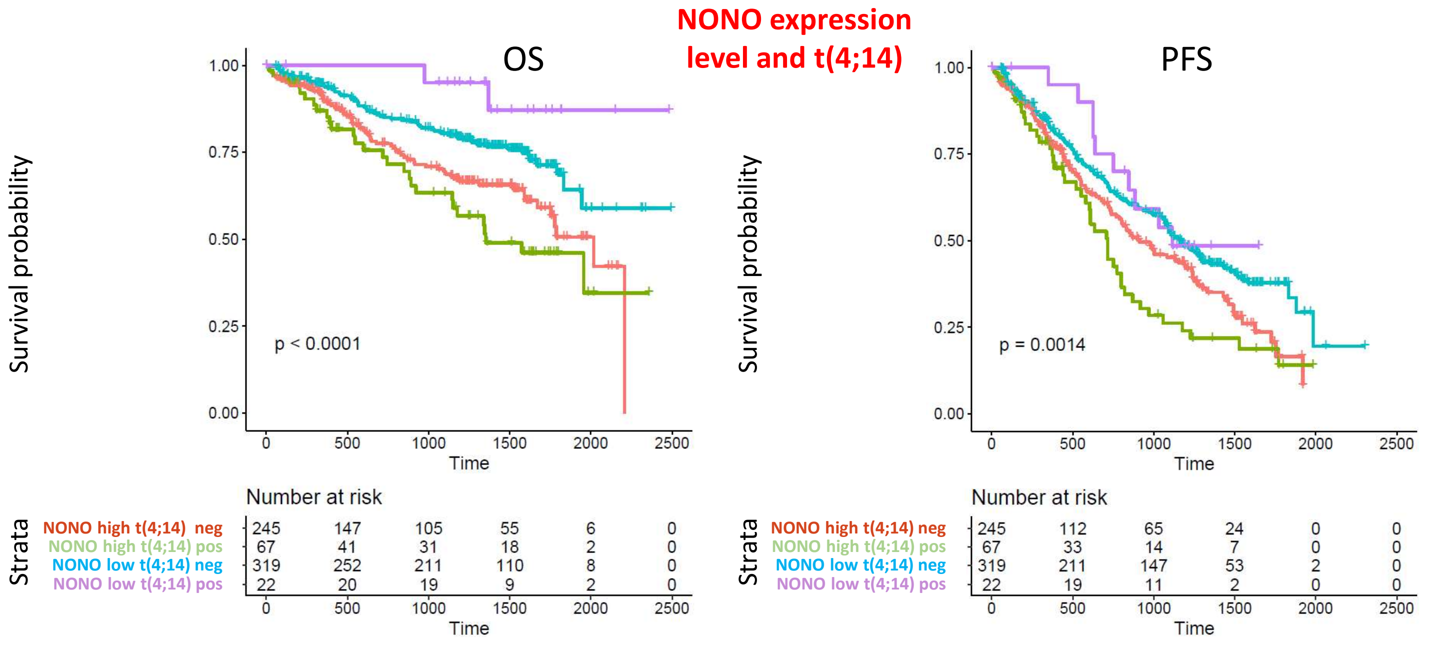

OS	High NONO t(4;14) neg	High NONO t(4;14) pos	Low NONO t(4;14) neg
High NONO_t(4;14) pos	0.2171		
Low NONO_t(4;14) neg	**0.0048**	**0.0009**	
Low NONO_t(4;14) pos	**0.0130**	**0.0048**	0.129

PFS	High NONO t(4;14) neg	High NONO t(4;14) pos	Low NONO t(4;14) neg
High NONO_t(4;14) pos	0.160		
Low NONO_t(4;14) neg	**0.035**	**0.0026**	
Low NONO_t(4;14) pos	0.238	**0.0351**	0.611

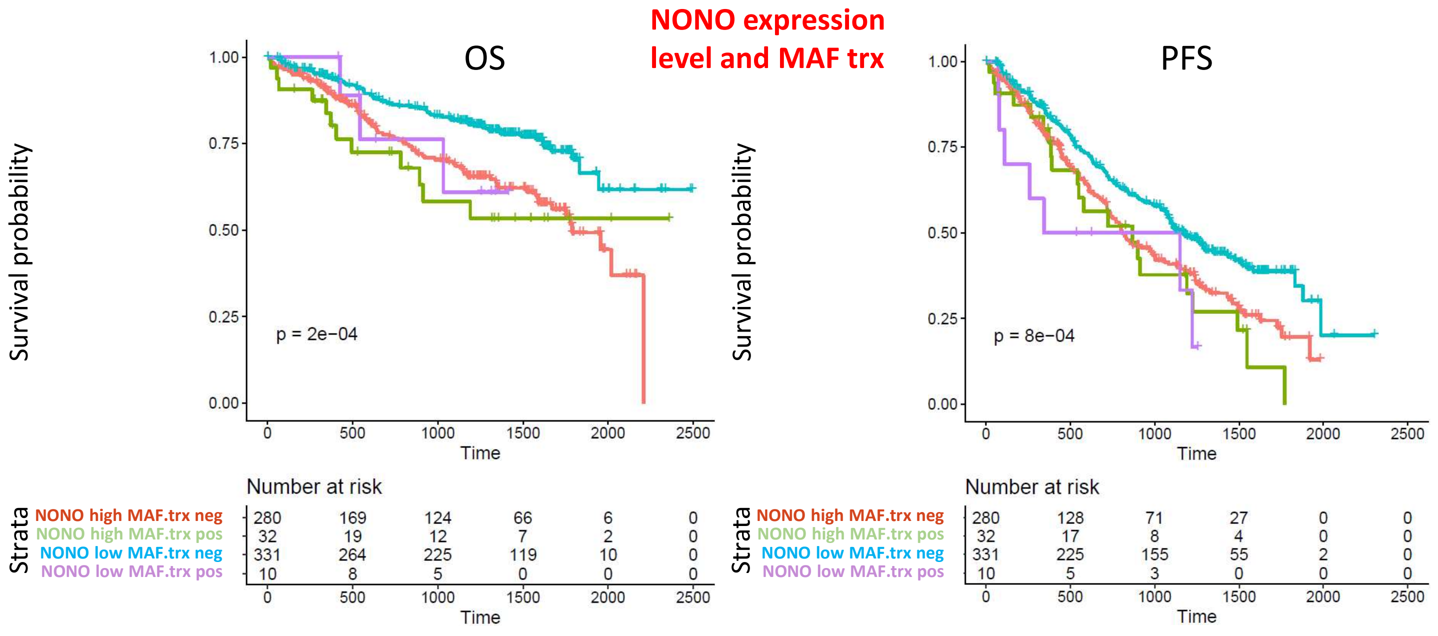

OS	High NONO MAF.trx neg	High NONO MAF.trx pos	Low NONO MAF.trx neg
High NONO_MAF.trx pos	0.704		
Low NONO_MAF.trx neg	**0.00024**	**0.016**	
Low NONO_MAF.trx pos	0.981	0.704	0.555

PFS	High NONO MAF.trx neg	High NONO MAF.trx pos	Low NONO MAF.trx neg
High NONO_MAF.trx pos	0.460		
Low NONO_MAF.trx neg	**0.0064**	**0.038**	
Low NONO_MAF.trx pos	0.318	0.460	0.058

Supplementary Figure S5: Kaplan-Meier survival curves in 653 MM with expression, molecular and clinical data available. Log-rank test p-value measuring the global difference between survival curves and number of samples at risk in each group across time are reported. Log-rank test p-values of pairwise comparisons are also reported. Significant adjusted p-values by BH correction (< 0.05) are in red-bold.

NONO expression level and *DIS3* mutation (DIS3m)

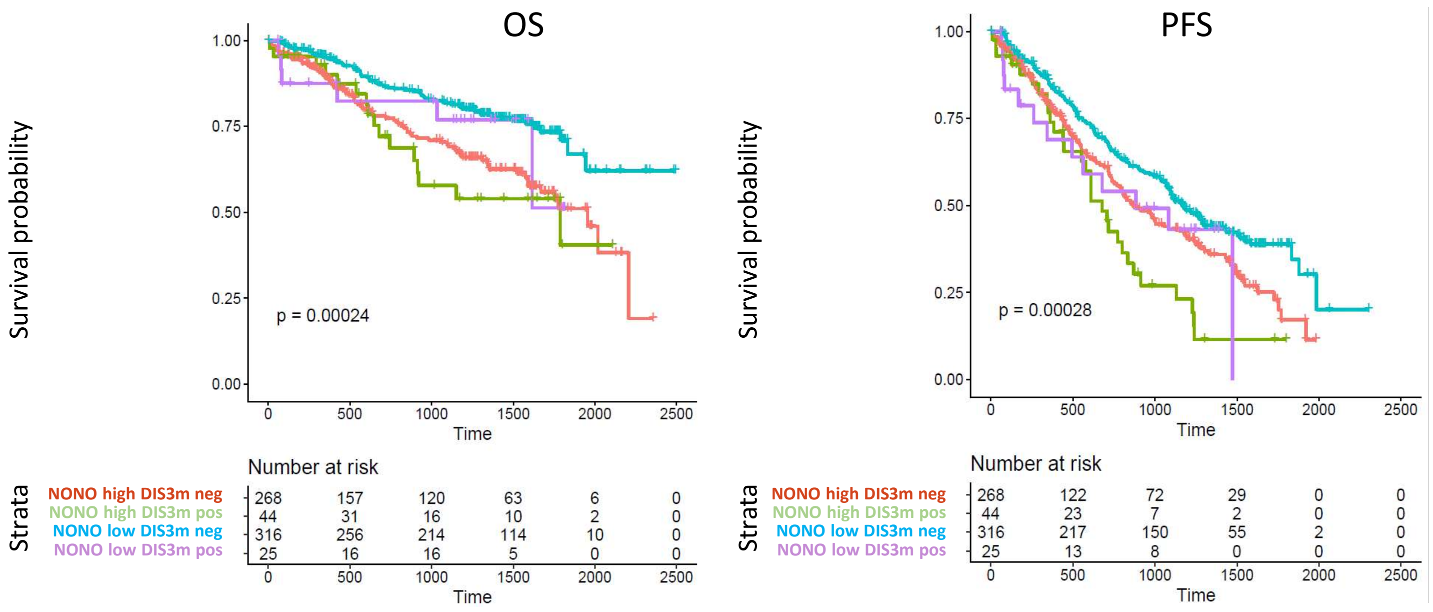

OS	High NONO DIS3m neg	High NONO DIS3m pos	Low NONO DIS3m neg
**High NONO_DIS3m pos**	0.566		
**Low NONO_DIS3m neg**	**0.00034**	**0.0070**	
**Low NONO_DIS3m pos**	0.566	0.565	0.565

PFS	High NONO DIS3m neg	High NONO DIS3m pos	Low NONO DIS3m neg
**High NONO_DIS3m pos**	0.061		
**Low NONO_DIS3m neg**	**0.014**	**0.00046**	
**Low NONO_DIS3m pos**	0.753	0.249	0.249

Supplementary Figure S6: Kaplan-Meier survival curves in 653 MM with expression, molecular and clinical data available. Log-rank test p-value measuring the global difference between survival curves and number of samples at risk in each group across time are reported. Log-rank test p-values of pairwise comparisons are also reported. Significant adjusted p-values by BH correction (< 0.05) are in red-bold.

a

Variable	N (%)	OS Univariate Cox Analysis		
		HR (95% CI)	P-value	Adj. P-value
**high *NONO***	247 (50)	2.4 (1.7-3.5)	0.000003	**0.000023******
**Age (≥ 65 yrs)**	206 (41)	2.12 (1.46-3.08)	0.0000868	**0.0004*****
**ISS I**	187 (38)	0.31 (0.19-0.50)	1.86E-06	**0.000021******
**ISS II**	171 (34)	1.13 (0.78-1.64)	0.524	0.7089
**ISS III**	139 (28)	2.45 (1.70-3.53)	1.62E-06	**0.00002139******
**del(1p)/CDKN2C**	143 (29)	1.61 (1.10-2.35)	0.0148	**0.03807***
**del(13q)/RB1**	258 (52)	2.11 (1.44-3.09)	0.000119	**0.00045*****
**HD**	281 (56)	0.64 (0.44-0.92)	0.0149	**0.0380***
**TP53.alterations**	40 (8)	1.05 (0.53-2.07)	0.892	0.932
**1q21 gain/amp**	164 (33)	1.68 (1.17-2.43)	0.0051	**0.0167***
**TP53.alterations + 1q21 gain/amp**	19 (4)	3.63 (1.89-6.97)	0.000103	**0.00045*****
**DIS3 mut**	50 (10)	1.63 (1.06-2.50)	0.0264	0.057
**N-RAS mut**	117 (23)	0.90 (0.62-1.32)	0.596	0.760
**K-RAS mut**	121 (24)	1.07 (0.77-1.49)	0.694	0.795
**BRAF mut**	36 (7)	1.18 (0.60-2.31)	0.628	0.760
**FAM46C mut**	49 (10)	0.78 (0.42-1.43)	0.419	0.602
**TRAF3 mut**	38 (7)	0.42 (0.17-1.06)	0.0668	0.117
**t(11;14)**	102 (20)	0.92 (0.58-1.46)	0.726	0.795
**t(4;14)**	69 (14)	1.63 (1.04-2.55)	0.0335	0.064
**MAF.trx**	33 (7)	1.96 (1.08-3.57)	0.0276	0.057
**MYC.trx**	20 (4)	1.92 (0.94-3.94)	0.0748	0.117

b

Variable	N (%)	PFS Univariate Cox Analysis		
		HR (95% CI)	P-value	Adj. P-value
**high *NONO***	247 (50)	1.7 (1.3-2.2)	0.000068	**0.00039******
**Age (≥ 65 yrs)**	206 (41)	1,59 (1,23-2,05)	0.000372	**0.0017****
**ISS I**	187 (38)	0,44 (0,34-0,61)	0.00000012	**0.00000276*******
**ISS II**	171 (34)	1,2 (0,91-1,5)	0.213	0.326
**ISS III**	139 (28)	1,9 (1,48-2,5)	0.00000114	**0.00001311******
**del(1p)/CDKN2C**	143 (29)	1,3 (0,98-1,71)	0.0646	0.114
**del(13q)/RB1**	258 (52)	1,7 (1,32-2,21)	0.0000471	**0.000361*****
**HD**	281 (56)	0,66 (0,52-0,86)	0.00166	**0.0054****
**TP53.alterations**	40 (8)	0,85 (0,52-1,4)	0.511	0.691
**1q21 gain/amp**	164 (33)	1,52 (1,17-1,97)	0.00151	**0.0054****
**TP53.alterations + 1q21 gain/amp**	19 (4)	2,46 (1,38-4,41)	0.00243	**0.0069****
**DIS3 mut**	50 (10)	1,62 (1,18-2,22)	0,00308	**0.0078****
**N-RAS mut**	117 (23)	1,01 (0,79-1,29)	0.924	0.924
**K-RAS mut**	121 (24)	1,03 (0,80-1,31)	0.827	0.864
**BRAF mut**	36 (7)	0,88 (0,52-1,48)	0.626	0.757
**FAM46C mut**	49 (10)	1,07 (0,74-1,52)	0.728	0.797
**TRAF3 mut**	38 (7)	0,80 (0,52-1,22)	0.297	0.426
**t(11;14)**	102 (20)	0,94 (0,68-1,30)	0.694	0.797
**t(4;14)**	69 (14)	1,6 (1,14-2,23)	0.00612	**0.0140***
**MAF.trx**	33 (7)	1,63 (1,03-2,57)	0.0376	0.072
**MYC.trx**	20 (4)	1,94 (1,13-3,33)	0.0163	**0.0340***

Supplementary Figure S7: Results of Cox regression univariate analysis using OS **(a)** or PFS **(b)** data on NONO expression groups, age equal to or greater than 65 years, ISS subgroups and main molecular alterations in 497 BM-1 MM cases for which all data were available. Number (N) of positive cases is indicated for each variable. Hazard Ratio, 95% Confidence Interval and Log-rank p-value are reported for each variable. In red bold are depicted all significant variables after BH correction. *≤0.05; **≤0.01;***≤0.001;****≤0.0001

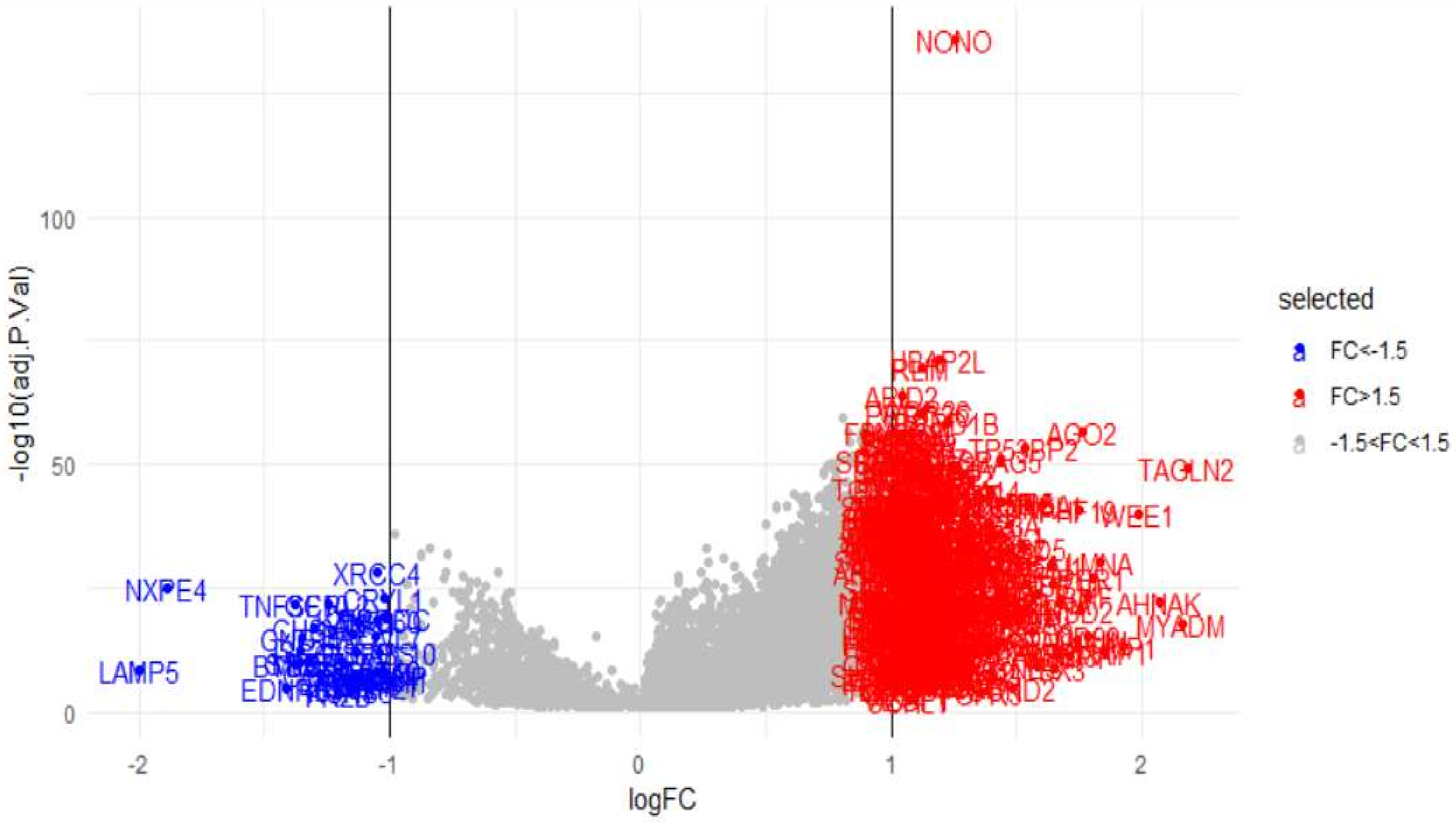

Supplementary Figure S8: Volcano plot of 11872 significant DE protein coding transcripts between NONO extreme quartiles. Up and down-regulated transcripts with at least 1.5 FC absolute values are highlighted in red and blue, respectively.

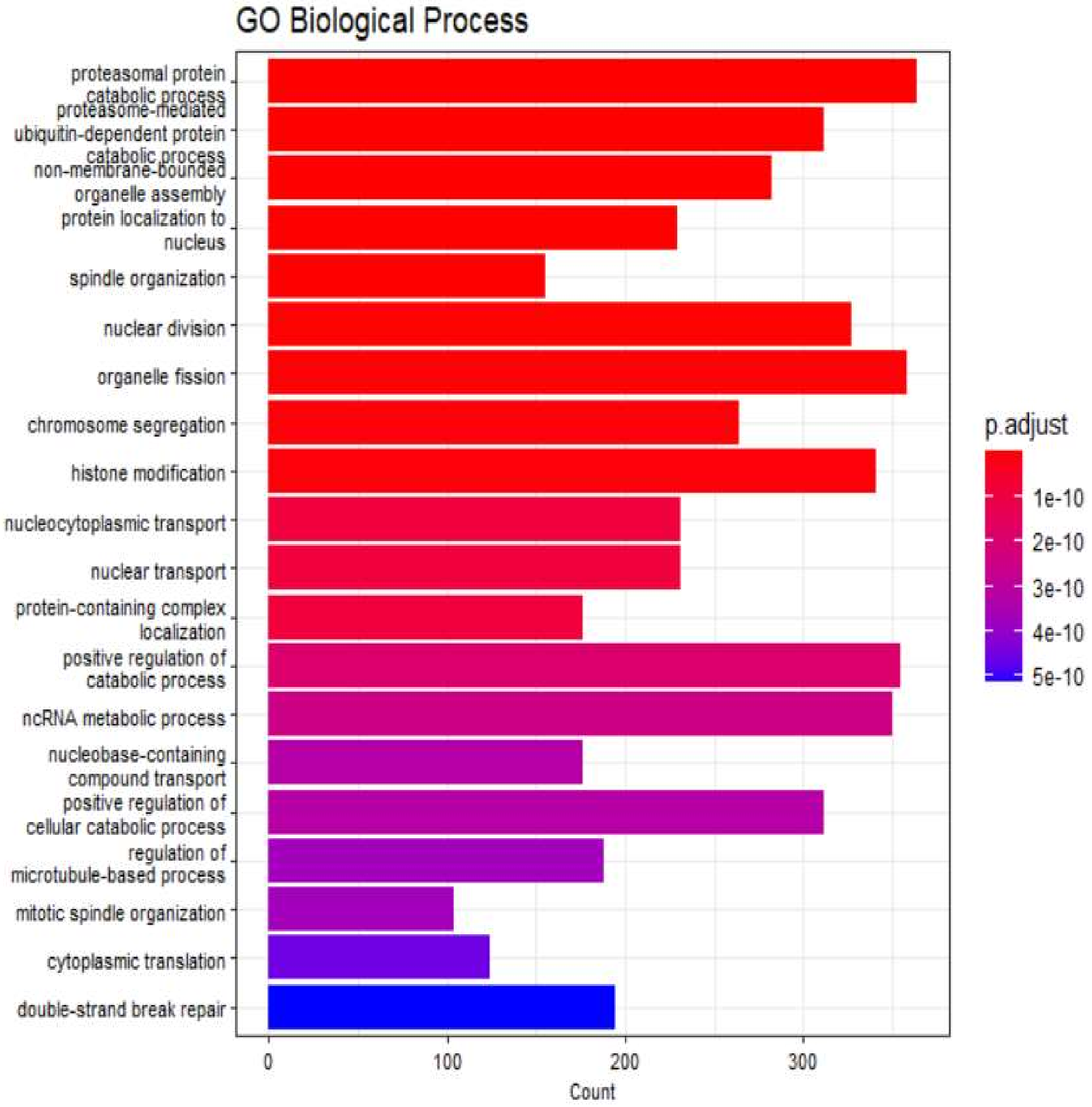

Supplementary Figure S9: Barplot of the top 20 GO biological processes, performed by cluster Profiler analysis on DE global protein gene list.

Supplementary material reporting the whole original membranes used for Figures in the manuscript showing western blot results. All bands and molecular weight markers are reported

FIGURE 1b

NONO

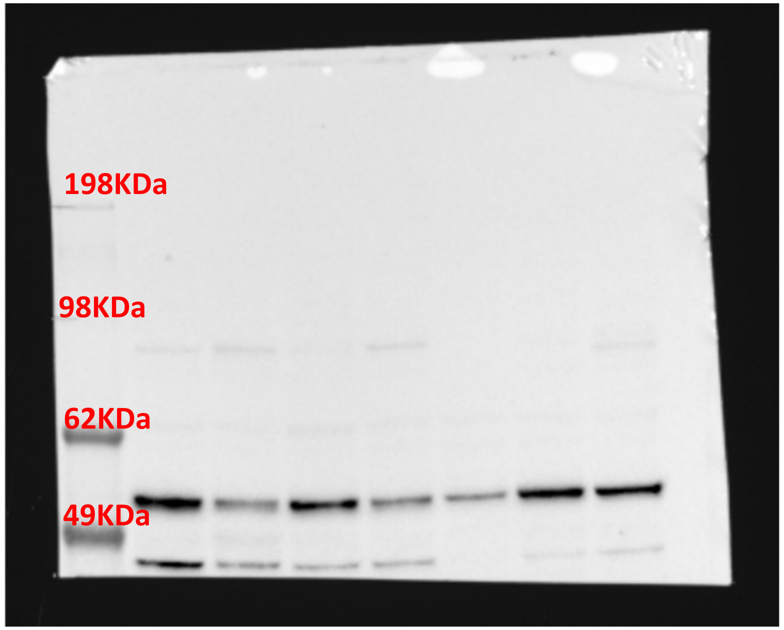

GAPDH

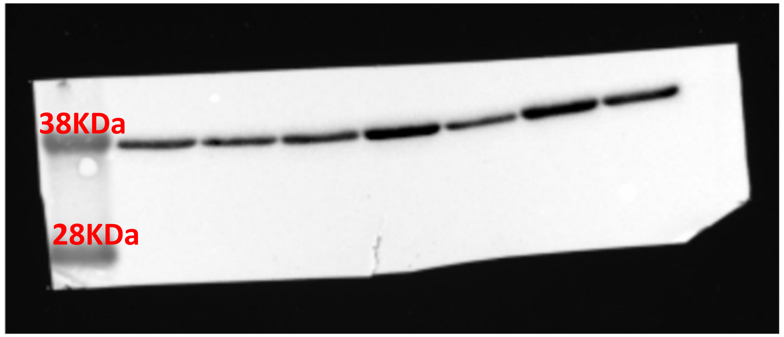

Supplementary Table S1. Number and relative frequency of main IgH translocations (trx), copy number alterations (CNAs) and non-synonymous (NS) somatic mutations, in 660 BM-1 MM cases of MMRF_CoMMpass_IA15a cohort with available data about NONO expression by RNA sequencing (RNA-seq), IgH trx by RNA-seq, NS somatic mutations by Whole Exome sequencing (WES) and CNAs by next generation sequencing (NGS)-based FISH (FISH-WES).

**IgH trx (RNA-seq)**	**N (%)**
t(11;14)/CCND1	136 (20.6%)
t(6;14)/CCND3	9 (1.4%)
t(4;14)/WHSC1/FGFR3	89 (13.5%)
t(14;16)/MAF;t(14;20)/MAFB; t(8;16)/MAFA	42 (6.4%)
t(8;14)/MYC	27 (4.1%)
**CNA (FISH-WES)**	**N (%)**
del(13)(q14)/(q34)/RB1_20%	346 (52.4%)
1q21 gain_20%	240 (36.4%)
del(1)(p22)/CDKN2C_20%	199 (30.2%)
del(17)(p13)/TP53_20%	74 (11.2%)
HD	375 (56.8%)
**NS Somatic Mutation (WES)**	**N (%)**
DIS3	71 (10.8%)
N-RAS	146 (22.1%)
H-RAS	0 (0%)
K-RAS	160 (24.2%)
BRAF	51 (7.7%)
TP53	30 (4.5%)
FAM46C	66 (10%)
TRAF3	50 (7.6%)

Supplementary Table S2. List of significant differentially expressed protein coding genes between NONO extreme quartiles, by limma analysis at FDR 10% cut-off. 9808 up- and 2064 down-regulated transcripts in quartile IV vs I are respectively ordered according to adjusted Pvalue. Fold change (FC), p-value and B statistics parameters are reported for each gene.

Gene ID	Gene Symbol	FC	P.Value	adj.P.Val	B
ENSG00000147140	NONO	2.3861	1.27E-140	2.40E-136	309.5448
ENSG00000143569	UBAP2L	2.2820	5.66E-76	5.32E-72	162.3760
ENSG00000131263	RLIM	2.1723	3.14E-74	1.97E-70	158.3912
ENSG00000189079	ARID2	2.0587	2.23E-68	1.05E-64	145.0184
ENSG00000162923	WDR26	2.1974	2.59E-65	9.76E-62	138.0117
ENSG00000117523	PRRC2C	2.1614	1.32E-64	4.15E-61	136.3947
ENSG00000151532	VTI1A	1.7541	9.89E-64	2.66E-60	134.3973
ENSG00000124789	NUP153	1.9848	1.86E-63	4.38E-60	133.7686
ENSG00000101413	RPRD1B	2.3410	1.02E-62	2.14E-59	132.0771
ENSG00000135829	DHX9	1.8706	8.02E-62	1.51E-58	130.0328
ENSG00000124209	RAB22A	1.7772	1.36E-60	2.13E-57	127.2214
ENSG00000143756	FBXO28	2.0154	1.29E-60	2.13E-57	127.2725
ENSG00000101146	RAE1	1.8471	2.46E-60	3.31E-57	126.6333
ENSG00000123908	AGO2	3.3879	2.35E-60	3.31E-57	126.6790
ENSG00000084463	WBP11	2.1089	3.00E-60	3.77E-57	126.4362
ENSG00000136699	SMPD4	2.0644	5.20E-59	6.11E-56	123.6052
ENSG00000080371	RAB21	2.0599	1.13E-58	1.25E-55	122.8321
ENSG00000111530	CAND1	1.8396	1.29E-58	1.35E-55	122.7012
ENSG00000144580	CNOT9	1.7874	1.83E-58	1.81E-55	122.3551
ENSG00000124177	CHD6	2.0388	3.04E-58	2.86E-55	121.8496
ENSG00000213064	SFT2D2	2.1107	5.09E-58	4.56E-55	121.3388
ENSG00000096746	HNRNPH3	1.8573	8.12E-58	6.94E-55	120.8757
ENSG00000143373	ZNF687	2.0977	1.35E-57	1.11E-54	120.3695
ENSG00000135870	RC3H1	2.1567	9.47E-57	7.19E-54	118.4362
ENSG00000143514	TP53BP2	2.8942	9.56E-57	7.19E-54	118.4273
ENSG00000111647	UHRF1BP1L	1.9138	2.49E-56	1.80E-53	117.4767
ENSG00000138081	FBXO11	1.9015	7.55E-56	5.26E-53	116.3744
ENSG00000104472	CHRAC1	1.8721	9.38E-56	6.30E-53	116.1595
ENSG00000109381	ELF2	2.1919	1.22E-55	7.93E-53	115.8970
ENSG00000132323	ILKAP	1.7931	2.16E-55	1.35E-52	115.3334
ENSG00000135801	TAF5L	2.0215	5.36E-55	3.25E-52	114.4293
ENSG00000164754	RAD21	1.9429	8.42E-55	4.95E-52	113.9801
ENSG00000196850	PPTC7	2.2126	1.04E-54	5.96E-52	113.7659
ENSG00000076382	SPAG5	2.6997	1.68E-54	9.29E-52	113.2945
ENSG00000196504	PRPF40A	1.7559	2.06E-54	1.11E-51	113.0896
ENSG00000117625	RCOR3	2.0636	2.32E-54	1.21E-51	112.9728
ENSG00000163444	TMEM183A	1.7780	2.63E-54	1.34E-51	112.8507
ENSG00000108510	MED13	1.9647	3.21E-54	1.59E-51	112.6525
ENSG00000197111	PCBP2	1.7354	3.35E-54	1.62E-51	112.6080
ENSG00000165288	BRWD3	1.8092	3.62E-54	1.70E-51	112.5319
ENSG00000156502	SUPV3L1	2.0070	3.91E-54	1.79E-51	112.4566
ENSG00000143436	MRPL9	1.7911	6.55E-54	2.93E-51	111.9438
ENSG00000102225	CDK16	2.0189	8.43E-54	3.69E-51	111.6926
ENSG00000158711	ELK4	1.9865	1.32E-53	5.66E-51	111.2452
ENSG00000108883	EFTUD2	1.6930	1.93E-53	8.06E-51	110.8708
ENSG00000149658	YTHDF1	1.7175	2.28E-53	9.32E-51	110.7054
ENSG0000068878	PSME4	1.9867	2.76E-53	1.10E-50	110.5150
ENSG00000134644	PUM1	1.8698	3.26E-53	1.28E-50	110.3489

Gene ID	Gene Symbol	FC	P.Value	adj.P.Val	B
ENSG00000066084	DIP2B	1.6719	3.68E-53	1.41E-50	110.2287
ENSG00000143614	GATAD2B	1.8775	4.49E-53	1.69E-50	110.0307
ENSG00000151553	FHIP2A	2.3584	5.64E-53	2.08E-50	109.8061
ENSG00000158710	TAGLN2	4.5436	9.53E-53	3.45E-50	109.2842
ENSG00000068308	OTUD5	1.7082	1.56E-52	5.54E-50	108.7940
ENSG00000151893	CACUL1	1.9457	1.91E-52	6.64E-50	108.5958
ENSG00000136738	STAM	1.8600	2.60E-52	8.89E-50	108.2880
ENSG00000154845	PPP4R1	2.2275	5.08E-52	1.71E-49	107.6224
ENSG00000100221	JOSD1	2.0788	6.23E-52	2.02E-49	107.4195
ENSG00000187522	HSPA14	1.7053	6.17E-52	2.02E-49	107.4297
ENSG00000007168	PAFAH1B1	1.9563	7.59E-52	2.42E-49	107.2234
ENSG00000239305	RNF103	2.1537	1.10E-51	3.45E-49	106.8557
ENSG00000095485	CWF19L1	1.6633	1.19E-51	3.57E-49	106.7780
ENSG00000105879	CBLL1	2.0108	1.20E-51	3.57E-49	106.7722
ENSG00000153207	AHCTF1	2.1145	1.19E-51	3.57E-49	106.7788
ENSG00000143751	SDE2	2.4391	1.81E-51	5.33E-49	106.3601
ENSG00000136715	SAP130	1.8873	1.89E-51	5.47E-49	106.3173
ENSG00000146872	TLK2	1.6627	3.91E-51	1.12E-48	105.5952
ENSG00000156313	RPGR	2.1632	4.19E-51	1.18E-48	105.5281
ENSG00000143621	ILF2	1.7193	9.91E-51	2.74E-48	104.6731
ENSG00000152242	C18orf25	1.8354	1.66E-50	4.54E-48	104.1576
ENSG00000022840	RNF10	1.9122	1.88E-50	5.06E-48	104.0348
ENSG00000005339	CREBBP	2.2176	2.26E-50	5.99E-48	103.8541
ENSG00000143862	ARL8A	2.2620	2.29E-50	5.99E-48	103.8406
ENSG00000116560	SFPQ	1.9395	2.54E-50	6.54E-48	103.7395
ENSG00000067900	ROCK1	2.1156	3.14E-50	7.99E-48	103.5267
ENSG00000180182	MED14	1.7240	5.58E-50	1.40E-47	102.9560
ENSG00000099381	SETD1A	1.7106	7.80E-50	1.93E-47	102.6238
ENSG00000101654	RNMT	1.8437	7.99E-50	1.95E-47	102.6001
ENSG00000157625	TAB3	1.7638	1.49E-49	3.59E-47	101.9811
ENSG00000171456	ASXL1	1.9634	1.81E-49	4.31E-47	101.7877
ENSG00000116786	PLEKHM2	1.8577	2.90E-49	6.82E-47	101.3199
ENSG00000118873	RAB3GAP2	1.9780	4.00E-49	9.28E-47	101.0019
ENSG00000074054	CLASP1	1.8538	5.71E-49	1.31E-46	100.6476
ENSG00000102081	FMR1	2.3347	7.07E-49	1.58E-46	100.4357
ENSG00000124486	USP9X	1.9077	7.03E-49	1.58E-46	100.4402
ENSG00000135913	USP37	1.7247	1.15E-48	2.55E-46	99.9501
ENSG00000156304	SCAF4	1.9295	2.29E-48	5.02E-46	99.2666
ENSG00000198625	MDM4	2.2541	2.71E-48	5.86E-46	99.1005
ENSG00000169905	TOR1AIP2	2.0123	2.97E-48	6.35E-46	99.0106
ENSG00000139613	SMARCC2	1.8701	3.34E-48	7.07E-46	98.8922
ENSG00000139218	SCAF11	1.6928	4.09E-48	8.56E-46	98.6916
ENSG00000198815	FOXJ3	1.6965	4.68E-48	9.68E-46	98.5579
ENSG00000066117	SMARCD1	1.6744	5.03E-48	1.02E-45	98.4874
ENSG00000166225	FRS2	1.8150	5.05E-48	1.02E-45	98.4827
ENSG00000196470	SIAH1	2.0046	6.77E-48	1.35E-45	98.1925
ENSG00000103275	UBE2I	1.7000	7.11E-48	1.41E-45	98.1429
ENSG00000116903	EXOC8	2.0359	8.17E-48	1.60E-45	98.0050
ENSG00000118298	CA14	2.6230	8.34E-48	1.62E-45	97.9851
ENSG00000198455	ZXDB	1.9402	1.09E-47	2.10E-45	97.7160
ENSG00000158796	DEDD	1.8282	2.13E-47	4.05E-45	97.0538
ENSG00000144136	SLC20A1	2.2807	2.15E-47	4.05E-45	97.0431
ENSG00000047410	TPR	1.9214	2.18E-47	4.06E-45	97.0304
ENSG00000154640	BTG3	2.4606	3.60E-47	6.65E-45	96.5316

Gene ID	Gene Symbol	FC	P.Value	adj.P.Val	B
ENSG00000182944	EWSR1	1.7898	3.64E-47	6.65E-45	96.5212
ENSG00000110880	CORO1C	2.3533	4.04E-47	7.30E-45	96.4189
ENSG00000183283	DAZAP2	1.6776	4.44E-47	7.95E-45	96.3256
ENSG00000010244	ZNF207	1.5292	5.06E-47	8.97E-45	96.1956
ENSG00000152102	FAM168B	1.6690	6.25E-47	1.10E-44	95.9851
ENSG00000082153	BZW1	1.7878	6.77E-47	1.17E-44	95.9063
ENSG00000176853	FAM91A1	1.8486	6.71E-47	1.17E-44	95.9141
ENSG00000153187	HNRNPU	1.8758	7.58E-47	1.30E-44	95.7938
ENSG00000138050	THUMPD2	1.7473	8.75E-47	1.48E-44	95.6510
ENSG00000169895	SYAP1	1.8789	8.94E-47	1.50E-44	95.6291
ENSG00000102054	RBBP7	1.6471	1.49E-46	2.49E-44	95.1195
ENSG00000065548	ZC3H15	1.5849	1.86E-46	3.04E-44	94.9019
ENSG00000168488	ATXN2L	1.8097	1.85E-46	3.04E-44	94.9065
ENSG00000169375	SIN3A	1.9989	2.39E-46	3.88E-44	94.6534
ENSG00000067596	DHX8	1.7619	2.45E-46	3.94E-44	94.6287
ENSG00000116350	SRSF4	1.8044	2.49E-46	3.97E-44	94.6126
ENSG00000114933	INO80D	1.8431	2.66E-46	4.20E-44	94.5472
ENSG00000103404	USP31	1.9402	2.89E-46	4.50E-44	94.4641
ENSG00000129315	CCNT1	1.7085	2.89E-46	4.50E-44	94.4630
ENSG00000175073	VCPIP1	1.8411	3.07E-46	4.73E-44	94.4048
ENSG00000174010	KLHL15	1.9521	3.74E-46	5.72E-44	94.2078
ENSG00000119953	SMNDC1	1.6334	3.82E-46	5.79E-44	94.1879
ENSG00000109111	SUPT6H	1.8381	4.19E-46	6.30E-44	94.0961
ENSG00000111011	RSRC2	1.9086	4.27E-46	6.37E-44	94.0774
ENSG00000119787	ATL2	1.7824	4.91E-46	7.28E-44	93.9377
ENSG00000167986	DDB1	1.5482	5.13E-46	7.54E-44	93.8951
ENSG00000160741	CRTC2	2.1510	5.43E-46	7.92E-44	93.8385
ENSG00000111615	KRR1	1.7040	5.55E-46	8.03E-44	93.8170
ENSG00000159259	CHAF1B	1.8045	7.91E-46	1.14E-43	93.4643
ENSG00000204120	GIGYF2	1.7596	1.28E-45	1.82E-43	92.9891
ENSG00000008294	SPAG9	2.3139	1.35E-45	1.91E-43	92.9324
ENSG00000115216	NRBP1	1.9402	1.62E-45	2.27E-43	92.7554
ENSG00000175104	TRAF6	1.9364	1.73E-45	2.40E-43	92.6903
ENSG00000115520	COQ10B	1.7094	1.94E-45	2.68E-43	92.5761
ENSG00000143322	ABL2	1.7480	2.09E-45	2.87E-43	92.5003
ENSG00000108187	PBLD	1.9530	2.11E-45	2.87E-43	92.4925
ENSG00000116871	MAP7D1	2.3545	2.27E-45	3.08E-43	92.4163
ENSG00000090061	CCNK	1.7896	2.49E-45	3.34E-43	92.3281
ENSG00000139624	CERS5	1.5774	2.60E-45	3.47E-43	92.2817
ENSG00000156671	SAMD8	1.8234	2.74E-45	3.62E-43	92.2328
ENSG00000204165	CXorf65	2.7716	3.16E-45	4.16E-43	92.0902
ENSG00000145414	NAF1	1.7177	3.53E-45	4.62E-43	91.9790
ENSG00000077235	GTF3C1	1.8124	3.64E-45	4.72E-43	91.9493
ENSG00000169239	CA5B	2.7125	4.06E-45	5.23E-43	91.8409
ENSG00000133703	KRAS	2.0162	5.06E-45	6.40E-43	91.6219
ENSG00000156650	KAT6B	1.8407	5.07E-45	6.40E-43	91.6205
ENSG00000198408	OGA	2.0958	5.06E-45	6.40E-43	91.6224
ENSG00000100393	EP300	1.8524	5.51E-45	6.91E-43	91.5382
ENSG00000176915	ANKLE2	1.7436	5.61E-45	6.99E-43	91.5195
ENSG00000165195	PIGA	3.0510	6.05E-45	7.49E-43	91.4451
ENSG00000111300	NAA25	1.7164	7.03E-45	8.59E-43	91.2954
ENSG00000162702	ZNF281	2.1678	7.00E-45	8.59E-43	91.2996
ENSG00000035928	RFC1	1.6648	7.36E-45	8.94E-43	91.2498
ENSG00000152795	HNRNPDL	1.5632	8.77E-45	1.06E-42	91.0761

Gene ID	Gene Symbol	FC	P.Value	adj.P.Val	B
ENSG00000107863	ARHGAP21	2.1361	1.07E-44	1.28E-42	90.8818
ENSG00000158480	SPATA2	1.9696	1.08E-44	1.28E-42	90.8727
ENSG00000139083	ETV6	2.2996	1.10E-44	1.30E-42	90.8503
ENSG00000176624	MEX3C	2.1560	1.13E-44	1.32E-42	90.8284
ENSG00000004487	KDM1A	1.5901	1.17E-44	1.37E-42	90.7874
ENSG00000061936	SFSWAP	1.6898	1.31E-44	1.52E-42	90.6757
ENSG00000174606	ANGEL2	1.7291	1.55E-44	1.79E-42	90.5123
ENSG00000127481	UBR4	1.9965	1.71E-44	1.96E-42	90.4154
ENSG00000104824	HNRNPL	1.4591	1.95E-44	2.22E-42	90.2833
ENSG00000065665	SEC61A2	2.0362	1.97E-44	2.23E-42	90.2723
ENSG00000119326	CTNNAL1	2.9169	2.01E-44	2.26E-42	90.2548
ENSG00000160714	UBE2Q1	1.6644	2.07E-44	2.32E-42	90.2249
ENSG00000109606	DHX15	1.7905	2.61E-44	2.90E-42	89.9945
ENSG00000005483	KMT2E	1.9600	2.71E-44	3.00E-42	89.9558
ENSG00000122958	VPS26A	1.7783	2.77E-44	3.04E-42	89.9362
ENSG00000143190	POU2F1	2.0707	2.84E-44	3.11E-42	89.9099
ENSG00000089234	BRAP	1.4959	2.92E-44	3.17E-42	89.8823
ENSG00000101945	SUV39H1	1.8060	3.15E-44	3.41E-42	89.8067
ENSG00000101596	SMCHD1	2.1708	3.65E-44	3.93E-42	89.6597
ENSG00000116747	RO60	1.9925	3.80E-44	4.06E-42	89.6199
ENSG00000107951	MTPAP	1.5746	3.83E-44	4.08E-42	89.6115
ENSG00000101367	MAPRE1	1.5731	4.34E-44	4.58E-42	89.4875
ENSG00000133226	SRRM1	1.7096	4.36E-44	4.58E-42	89.4835
ENSG00000204574	ABCF1	1.6944	4.47E-44	4.67E-42	89.4604
ENSG00000089022	MAPKAPK5	1.4553	4.64E-44	4.82E-42	89.4229
ENSG00000197170	PSMD12	1.7074	6.55E-44	6.77E-42	89.0807
ENSG00000163811	WDR43	1.7474	7.50E-44	7.71E-42	88.9458
ENSG00000107937	GTPBP4	1.6971	8.51E-44	8.70E-42	88.8204
ENSG00000132823	OSER1	2.1460	1.14E-43	1.16E-41	88.5286
ENSG00000119403	PHF19	3.3605	1.34E-43	1.35E-41	88.3701
ENSG00000032219	ARID4A	2.1025	1.70E-43	1.71E-41	88.1346
ENSG00000124198	ARFGEF2	1.8429	1.88E-43	1.88E-41	88.0349
ENSG00000138750	NUP54	1.8491	2.29E-43	2.28E-41	87.8387
ENSG00000156860	FBRS	2.0093	2.45E-43	2.43E-41	87.7703
ENSG00000101216	GMEB2	1.5644	3.80E-43	3.74E-41	87.3350
ENSG00000106462	EZH2	2.3625	4.57E-43	4.48E-41	87.1504
ENSG00000122218	COPA	1.7696	4.67E-43	4.55E-41	87.1302
ENSG00000139641	ESYT1	1.9254	6.05E-43	5.87E-41	86.8721
ENSG00000134758	RNF138	1.6981	6.74E-43	6.51E-41	86.7652
ENSG00000166483	WEE1	3.9680	7.49E-43	7.19E-41	86.6610
ENSG00000068323	TFE3	1.7097	7.89E-43	7.54E-41	86.6089
ENSG00000064313	TAF2	1.5901	9.11E-43	8.65E-41	86.4668
ENSG00000213782	DDX47	1.8260	9.92E-43	9.38E-41	86.3822
ENSG00000169762	TAPT1	2.2450	1.00E-42	9.43E-41	86.3717
ENSG00000062485	CS	1.5614	1.07E-42	9.98E-41	86.3107
ENSG00000185359	HGS	2.0468	1.42E-42	1.33E-40	86.0230
ENSG00000136044	APPL2	1.9106	1.52E-42	1.41E-40	85.9565
ENSG00000073584	SMARCE1	1.5893	1.86E-42	1.72E-40	85.7579
ENSG00000104517	UBR5	1.9581	1.88E-42	1.73E-40	85.7448
ENSG00000162408	NOL9	1.7201	2.30E-42	2.10E-40	85.5481
ENSG00000110906	KCTD10	1.6524	2.47E-42	2.24E-40	85.4783
ENSG00000109220	CHIC2	1.9249	2.53E-42	2.28E-40	85.4545
ENSG00000079332	SAR1A	1.6125	2.90E-42	2.61E-40	85.3161
ENSG00000116698	SMG7	1.7632	3.29E-42	2.95E-40	85.1906

Gene ID	Gene Symbol	FC	P.Value	adj.P.Val	B
ENSG00000124422	USP22	1.9083	3.75E-42	3.34E-40	85.0615
ENSG00000161526	SAP30BP	1.9268	5.70E-42	5.06E-40	84.6457
ENSG00000116273	PHF13	2.0049	5.96E-42	5.26E-40	84.6022
ENSG00000205208	C4orf46	1.8731	6.11E-42	5.37E-40	84.5778
ENSG00000023287	RB1CC1	2.0219	6.28E-42	5.49E-40	84.5507
ENSG00000144028	SNRNP200	1.6556	6.73E-42	5.87E-40	84.4807
ENSG00000147679	UTP23	1.5890	6.85E-42	5.93E-40	84.4645
ENSG00000173273	TNKS	2.1036	7.90E-42	6.82E-40	84.3224
ENSG00000108239	TBC1D12	1.5796	8.22E-42	7.06E-40	84.2830
ENSG00000100888	CHD8	1.7415	9.27E-42	7.93E-40	84.1633
ENSG00000023734	STRAP	1.7048	1.04E-41	8.84E-40	84.0481
ENSG00000110851	PRDM4	1.8199	1.04E-41	8.84E-40	84.0467
ENSG00000173064	HECTD4	1.7659	1.08E-41	9.08E-40	84.0151
ENSG00000011021	CLCN6	1.9323	1.42E-41	1.20E-39	83.7371
ENSG00000138376	BARD1	1.9531	1.48E-41	1.24E-39	83.6981
ENSG00000186432	KPNA4	1.6425	1.62E-41	1.35E-39	83.6104
ENSG00000134453	RBM17	1.6756	1.75E-41	1.45E-39	83.5334
ENSG00000025770	NCAPH2	1.8681	1.89E-41	1.55E-39	83.4580
ENSG00000137404	NRM	2.0147	1.88E-41	1.55E-39	83.4597
ENSG00000126012	KDM5C	1.8149	1.93E-41	1.58E-39	83.4377
ENSG00000133773	CCDC59	1.6974	1.99E-41	1.62E-39	83.4045
ENSG00000143793	C1orf35	1.9995	2.09E-41	1.69E-39	83.3576
ENSG00000163374	YY1AP1	1.6727	2.73E-41	2.21E-39	83.0898
ENSG00000081721	DUSP12	1.7566	2.79E-41	2.24E-39	83.0693
ENSG00000010072	SPRTN	1.7268	2.88E-41	2.31E-39	83.0369
ENSG00000125834	STK35	1.5939	3.36E-41	2.67E-39	82.8867
ENSG00000166226	CCT2	1.5863	3.47E-41	2.75E-39	82.8543
ENSG00000065613	SLK	1.8792	3.50E-41	2.76E-39	82.8459
ENSG00000170852	KBTBD2	1.7626	3.61E-41	2.84E-39	82.8145
ENSG00000141279	NPEPPS	1.8314	3.85E-41	3.02E-39	82.7505
ENSG00000157106	SMG1	2.0283	6.00E-41	4.68E-39	82.3100
ENSG00000070831	CDC42	1.6946	6.75E-41	5.25E-39	82.1922
ENSG00000162526	TSSK3	2.1371	7.81E-41	6.04E-39	82.0485
ENSG00000033122	LRRC7	2.0839	8.16E-41	6.28E-39	82.0050
ENSG00000112200	ZNF451	1.9039	8.18E-41	6.28E-39	82.0020
ENSG00000188295	ZNF669	1.7145	8.95E-41	6.84E-39	81.9133
ENSG00000101782	RIOK3	1.9056	9.15E-41	6.97E-39	81.8909
ENSG00000073614	KDM5A	1.7872	9.58E-41	7.27E-39	81.8450
ENSG00000125827	TMX4	2.4387	1.07E-40	8.09E-39	81.7342
ENSG00000163877	SNIP1	1.8369	1.17E-40	8.83E-39	81.6433
ENSG00000165891	E2F7	2.6265	1.24E-40	9.28E-39	81.5903
ENSG00000153827	TRIP12	1.7356	1.29E-40	9.63E-39	81.5497
ENSG00000122257	RBBP6	1.8125	1.31E-40	9.73E-39	81.5357
ENSG00000108381	ASPA	1.4164	1.58E-40	1.17E-38	81.3470
ENSG00000091039	OSBPL8	1.7865	1.64E-40	1.21E-38	81.3102
ENSG00000110944	IL23A	2.7668	1.67E-40	1.23E-38	81.2912
ENSG00000185049	NELFA	1.6705	1.84E-40	1.34E-38	81.1990
ENSG00000108349	CASC3	1.6060	2.16E-40	1.57E-38	81.0388
ENSG00000135823	STX6	1.7268	2.19E-40	1.59E-38	81.0235
ENSG00000090905	TNRC6A	1.8175	2.39E-40	1.73E-38	80.9357
ENSG00000184922	FMNL1	2.1342	2.60E-40	1.87E-38	80.8541
ENSG00000163349	HIPK1	1.9332	2.61E-40	1.88E-38	80.8497
ENSG00000169967	MAP3K2	1.6930	2.91E-40	2.08E-38	80.7436
ENSG00000177084	POLE	1.9682	3.13E-40	2.23E-38	80.6712

Gene ID	Gene Symbol	FC	P.Value	adj.P.Val	B
ENSG00000086758	HUWE1	1.6690	3.31E-40	2.35E-38	80.6144
ENSG00000062598	ELMO2	1.8835	3.43E-40	2.43E-38	80.5795
ENSG00000162769	FLVCR1	1.7666	3.47E-40	2.44E-38	80.5689
ENSG00000168172	HOOK3	1.8477	3.78E-40	2.65E-38	80.4838
ENSG00000165392	WRN	2.0574	3.81E-40	2.67E-38	80.4743
ENSG00000116731	PRDM2	2.0061	3.95E-40	2.75E-38	80.4402
ENSG00000151923	TIAL1	1.5412	4.17E-40	2.90E-38	80.3848
ENSG00000183741	CBX6	2.4972	4.22E-40	2.92E-38	80.3733
ENSG00000173744	AGFG1	1.6513	4.25E-40	2.93E-38	80.3668
ENSG00000141551	CSNK1D	1.9952	4.42E-40	3.03E-38	80.3274
ENSG00000164080	RAD54L2	1.7936	4.43E-40	3.03E-38	80.3261
ENSG00000258890	CEP95	2.0212	4.88E-40	3.33E-38	80.2288
ENSG00000164924	YWHAZ	1.7421	5.17E-40	3.51E-38	80.1711
ENSG00000116754	SRSF11	1.9496	5.54E-40	3.75E-38	80.1032
ENSG00000131470	PSMC3IP	2.0289	6.33E-40	4.27E-38	79.9708
ENSG00000077157	PPP1R12B	1.7299	7.03E-40	4.72E-38	79.8676
ENSG00000100991	TRPC4AP	1.6852	7.05E-40	4.72E-38	79.8644
ENSG00000104738	MCM4	2.4355	1.01E-39	6.76E-38	79.5036
ENSG00000135093	USP30	1.9022	1.27E-39	8.47E-38	79.2772
ENSG00000171865	RNASEH1	1.5485	1.28E-39	8.48E-38	79.2723
ENSG00000113569	NUP155	1.6575	1.30E-39	8.58E-38	79.2565
ENSG00000197386	HTT	1.9009	1.40E-39	9.21E-38	79.1829
ENSG00000076108	BAZ2A	2.0100	1.56E-39	1.03E-37	79.0734
ENSG00000155592	ZKSCAN2	1.5177	1.60E-39	1.04E-37	79.0525
ENSG00000125319	HROB	1.7776	1.72E-39	1.12E-37	78.9782
ENSG00000120370	GORAB	1.9417	1.92E-39	1.25E-37	78.8680
ENSG00000156802	ATAD2	2.2564	2.11E-39	1.36E-37	78.7775
ENSG00000162819	BROX	1.8781	2.13E-39	1.37E-37	78.7662
ENSG00000185753	CXorf38	1.5795	2.19E-39	1.41E-37	78.7383
ENSG00000099917	MED15	1.8005	2.28E-39	1.46E-37	78.6991
ENSG00000198265	HELZ	1.8264	2.61E-39	1.66E-37	78.5647
ENSG00000186918	ZNF395	2.4489	3.60E-39	2.29E-37	78.2447
ENSG00000170881	RNF139	1.6681	3.74E-39	2.37E-37	78.2076
ENSG00000185947	ZNF267	2.0053	3.90E-39	2.46E-37	78.1662
ENSG00000184634	MED12	1.9394	3.97E-39	2.50E-37	78.1479
ENSG00000081760	AACS	1.6139	4.01E-39	2.52E-37	78.1384
ENSG00000138668	HNRNPD	1.5290	4.39E-39	2.74E-37	78.0493
ENSG00000166913	YWHAB	1.5738	4.48E-39	2.79E-37	78.0283
ENSG00000119541	VPS4B	1.6973	4.66E-39	2.89E-37	77.9893
ENSG00000116604	MEF2D	1.9523	5.70E-39	3.53E-37	77.7894
ENSG00000141458	NPC1	1.6150	5.97E-39	3.68E-37	77.7431
ENSG00000111642	CHD4	1.6499	6.01E-39	3.69E-37	77.7376
ENSG00000166847	DCTN5	1.5200	6.57E-39	4.03E-37	77.6487
ENSG00000131051	RBM39	1.7361	8.52E-39	5.21E-37	77.3905
ENSG00000121644	DESI2	1.8062	8.75E-39	5.33E-37	77.3638
ENSG00000101247	NDUFAF5	1.9909	9.43E-39	5.73E-37	77.2897
ENSG00000106144	CASP2	1.7002	1.01E-38	6.13E-37	77.2183
ENSG00000188021	UBQLN2	1.7325	1.04E-38	6.25E-37	77.1966
ENSG00000115977	AAK1	1.8953	1.11E-38	6.65E-37	77.1319
ENSG00000126775	ATG14	1.8533	1.19E-38	7.12E-37	77.0611
ENSG00000025772	TOMM34	1.7070	1.23E-38	7.34E-37	77.0267
ENSG00000123374	CDK2	1.8802	1.34E-38	8.00E-37	76.9387
ENSG00000164951	PDP1	2.0687	1.41E-38	8.37E-37	76.8907
ENSG00000162971	TYW5	1.5277	1.46E-38	8.64E-37	76.8556

Gene ID	Gene Symbol	FC	P.Value	adj.P.Val	B
ENSG00000122033	MTIF3	0.5075	1.56E-38	9.22E-37	76.7887
ENSG00000106346	USP42	1.7990	1.63E-38	9.61E-37	76.7442
ENSG00000069248	NUP133	1.6898	1.74E-38	1.02E-36	76.6811
ENSG00000102390	PBDC1	1.5589	1.87E-38	1.10E-36	76.6082
ENSG00000124171	PARD6B	1.4803	2.01E-38	1.17E-36	76.5370
ENSG00000077721	UBE2A	1.7327	2.05E-38	1.19E-36	76.5195
ENSG00000047315	POLR2B	1.6264	2.23E-38	1.29E-36	76.4363
ENSG00000176407	KCMF1	1.5171	2.34E-38	1.35E-36	76.3869
ENSG00000147050	KDM6A	1.8569	2.41E-38	1.38E-36	76.3606
ENSG00000079134	THOC1	1.7131	2.48E-38	1.42E-36	76.3297
ENSG00000102100	SLC35A2	2.1611	2.62E-38	1.50E-36	76.2772
ENSG00000146463	ZMYM4	1.5951	2.86E-38	1.63E-36	76.1900
ENSG00000111596	CNOT2	1.5358	2.88E-38	1.64E-36	76.1829
ENSG00000066557	LRRC40	1.6478	2.97E-38	1.68E-36	76.1527
ENSG00000038219	BOD1L1	1.8460	3.35E-38	1.89E-36	76.0334
ENSG00000117000	RLF	1.9651	3.44E-38	1.94E-36	76.0063
ENSG00000185728	YTHDF3	1.5765	3.69E-38	2.07E-36	75.9354
ENSG00000100614	PPM1A	2.0422	3.76E-38	2.11E-36	75.9164
ENSG00000164715	LMTK2	1.7072	3.91E-38	2.18E-36	75.8787
ENSG00000115808	STRN	1.6343	4.30E-38	2.39E-36	75.7854
ENSG00000173960	UBXN2A	1.6816	4.59E-38	2.55E-36	75.7198
ENSG00000198742	SMURF1	2.0573	4.64E-38	2.57E-36	75.7079
ENSG00000108039	XPNPEP1	1.6232	4.71E-38	2.60E-36	75.6936
ENSG00000158615	PPP1R15B	1.8814	5.06E-38	2.79E-36	75.6221
ENSG00000071127	WDR1	1.8043	5.15E-38	2.83E-36	75.6046
ENSG00000075568	TMEM131	1.6844	5.88E-38	3.21E-36	75.4744
ENSG00000101193	GID8	1.4659	6.38E-38	3.48E-36	75.3921
ENSG00000110367	DDX6	1.7923	6.80E-38	3.70E-36	75.3300
ENSG00000119900	OGFRL1	2.6677	6.95E-38	3.77E-36	75.3077
ENSG00000149554	CHEK1	1.8926	7.38E-38	3.99E-36	75.2483
ENSG00000132716	DCAF8	1.8731	8.16E-38	4.40E-36	75.1487
ENSG00000164823	OSGIN2	1.8596	8.21E-38	4.41E-36	75.1430
ENSG00000170471	RALGAPB	1.6197	9.20E-38	4.93E-36	75.0295
ENSG00000157181	ODR4	1.7329	9.39E-38	5.02E-36	75.0097
ENSG00000109332	UBE2D3	1.5149	1.00E-37	5.34E-36	74.9456
ENSG00000174437	ATP2A2	1.8453	1.02E-37	5.42E-36	74.9272
ENSG00000105993	DNAJB6	1.6674	1.03E-37	5.44E-36	74.9213
ENSG00000157540	DYRK1A	1.7333	1.25E-37	6.59E-36	74.7269
ENSG00000143622	RIT1	1.8248	1.30E-37	6.84E-36	74.6870
ENSG00000159346	ADIPOR1	1.5868	1.30E-37	6.84E-36	74.6845
ENSG00000126767	ELK1	1.6458	1.35E-37	7.07E-36	74.6497
ENSG00000054267	ARID4B	1.8295	1.39E-37	7.25E-36	74.6219
ENSG00000185436	IFNLR1	2.2884	1.41E-37	7.34E-36	74.6068
ENSG00000089280	FUS	1.7605	1.42E-37	7.36E-36	74.6013
ENSG00000224470	ATXN1L	1.9514	1.48E-37	7.65E-36	74.5600
ENSG00000168092	PAFAH1B2	1.6469	1.74E-37	8.97E-36	74.3991
ENSG00000081913	PHLPP1	1.9237	2.12E-37	1.09E-35	74.2027
ENSG00000101109	STK4	1.8205	2.25E-37	1.16E-35	74.1413
ENSG00000138802	SEC24B	1.6805	2.76E-37	1.41E-35	73.9402
ENSG00000093167	LRRFIP2	1.7165	3.24E-37	1.66E-35	73.7793
ENSG00000181472	ZBTB2	1.7996	3.27E-37	1.66E-35	73.7724
ENSG00000126746	ZNF384	1.6379	3.51E-37	1.78E-35	73.7013
ENSG00000187555	USP7	1.6640	3.77E-37	1.91E-35	73.6288
ENSG00000010404	IDS	2.3431	3.88E-37	1.96E-35	73.6010

Gene ID	Gene Symbol	FC	P.Value	adj.P.Val	B
ENSG00000179295	PTPN11	1.5156	4.10E-37	2.07E-35	73.5473
ENSG00000168264	IRF2BP2	2.4622	4.30E-37	2.16E-35	73.5003
ENSG00000115761	NOL10	1.4752	5.57E-37	2.79E-35	73.2431
ENSG00000198026	ZNF335	1.6386	5.96E-37	2.98E-35	73.1752
ENSG00000100485	SOS2	1.7799	6.18E-37	3.07E-35	73.1400
ENSG00000128245	YWHAH	2.0193	6.16E-37	3.07E-35	73.1419
ENSG00000125107	CNOT1	1.6721	6.35E-37	3.15E-35	73.1129
ENSG00000115524	SF3B1	1.7563	6.59E-37	3.26E-35	73.0761
ENSG00000141026	MED9	1.6105	7.65E-37	3.78E-35	72.9276
ENSG00000068354	TBC1D25	1.5793	8.69E-37	4.28E-35	72.8012
ENSG00000108100	CCNY	1.4749	8.77E-37	4.31E-35	72.7917
ENSG00000065970	FOXJ2	1.6756	8.88E-37	4.35E-35	72.7802
ENSG00000215251	FASTKD5	1.7182	9.34E-37	4.56E-35	72.7298
ENSG00000118260	CREB1	1.6490	9.98E-37	4.86E-35	72.6640
ENSG00000083896	YTHDC1	2.0013	1.06E-36	5.17E-35	72.6002
ENSG00000115137	DNAJC27	1.6334	1.10E-36	5.35E-35	72.5638
ENSG00000143379	SETDB1	1.5856	1.11E-36	5.35E-35	72.5626
ENSG00000121749	TBC1D15	1.9498	1.35E-36	6.49E-35	72.3668
ENSG00000151461	UPF2	1.5386	1.44E-36	6.95E-35	72.2973
ENSG00000213047	DENND1B	2.1932	1.48E-36	7.09E-35	72.2741
ENSG00000196233	LCOR	1.7796	1.49E-36	7.11E-35	72.2694
ENSG00000123575	FAM199X	1.7169	1.86E-36	8.89E-35	72.0448
ENSG00000177731	FLII	2.0487	1.91E-36	9.08E-35	72.0214
ENSG00000058272	PPP1R12A	1.7521	1.99E-36	9.47E-35	71.9775
ENSG00000065526	SPEN	1.8316	2.11E-36	9.99E-35	71.9217
ENSG00000092201	SUPT16H	1.5986	2.32E-36	1.10E-34	71.8264
ENSG00000198160	MIER1	1.5911	2.99E-36	1.41E-34	71.5740
ENSG00000134308	YWHAQ	1.4912	3.65E-36	1.72E-34	71.3762
ENSG00000151014	NOCT	1.9807	3.77E-36	1.77E-34	71.3455
ENSG00000062650	WAPL	1.4758	3.85E-36	1.80E-34	71.3250
ENSG00000165813	CCDC186	1.8949	3.91E-36	1.82E-34	71.3098
ENSG00000100354	TNRC6B	2.0004	4.27E-36	1.99E-34	71.2225
ENSG00000188994	ZNF292	1.6927	5.05E-36	2.35E-34	71.0552
ENSG00000055609	KMT2C	2.0572	5.14E-36	2.38E-34	71.0368
ENSG00000125484	GTF3C4	1.6899	6.05E-36	2.80E-34	70.8762
ENSG00000139722	VPS37B	2.0749	6.71E-36	3.10E-34	70.7724
ENSG00000143753	DEGS1	1.6464	7.46E-36	3.43E-34	70.6677
ENSG00000122299	ZC3H7A	1.6636	9.26E-36	4.24E-34	70.4530
ENSG00000221823	PPP3R1	1.5431	9.26E-36	4.24E-34	70.4538
ENSG00000143761	ARF1	1.6353	9.33E-36	4.26E-34	70.4455
ENSG00000143337	TOR1AIP1	1.7349	9.55E-36	4.35E-34	70.4233
ENSG00000095574	IKZF5	1.8140	1.00E-35	4.55E-34	70.3762
ENSG00000114742	WDR48	1.8913	1.03E-35	4.69E-34	70.3434
ENSG00000151694	ADAM17	1.7062	1.06E-35	4.80E-34	70.3186
ENSG00000127314	RAP1B	1.6922	1.06E-35	4.80E-34	70.3148
ENSG00000036257	CUL3	1.5077	1.17E-35	5.29E-34	70.2175
ENSG00000240053	LY6G5B	2.0287	1.24E-35	5.57E-34	70.1635
ENSG00000151332	MBIP	1.7476	1.30E-35	5.82E-34	70.1172
ENSG00000089916	GPATCH2L	1.8734	1.34E-35	6.00E-34	70.0856
ENSG00000136861	CDK5RAP2	1.6330	1.55E-35	6.92E-34	69.9414
ENSG00000015568	RGPD5	2.8833	1.63E-35	7.25E-34	69.8928
ENSG00000182774	RPS17	0.5591	1.80E-35	8.01E-34	69.7914
ENSG00000029363	BCLAF1	1.8249	1.93E-35	8.54E-34	69.7248
ENSG00000179284	DAND5	1.2006	2.17E-35	9.59E-34	69.6075

Gene ID	Gene Symbol	FC	P.Value	adj.P.Val	B
ENSG00000123066	MED13L	1.9276	2.23E-35	9.84E-34	69.5799
ENSG00000176244	ACBD7	1.5605	2.56E-35	1.12E-33	69.4449
ENSG00000175215	CTDSP2	1.7631	2.84E-35	1.24E-33	69.3427
ENSG00000019995	ZRANB1	1.5210	2.88E-35	1.26E-33	69.3273
ENSG00000166266	CUL5	1.7830	2.97E-35	1.29E-33	69.2987
ENSG00000115548	KDM3A	1.8542	3.14E-35	1.37E-33	69.2416
ENSG00000163960	UBXN7	1.8652	3.20E-35	1.39E-33	69.2223
ENSG00000110080	ST3GAL4	2.0217	3.30E-35	1.43E-33	69.1936
ENSG00000133026	MYH10	1.9763	3.58E-35	1.55E-33	69.1131
ENSG00000165525	NEMF	1.5711	3.74E-35	1.61E-33	69.0684
ENSG00000104765	BNIP3L	1.9796	3.88E-35	1.67E-33	69.0330
ENSG00000138439	FAM117B	1.6541	4.02E-35	1.73E-33	68.9960
ENSG00000156735	BAG4	1.5620	4.46E-35	1.91E-33	68.8948
ENSG00000163161	ERCC3	1.5314	4.65E-35	1.99E-33	68.8529
ENSG00000173442	EHBP1L1	1.7842	5.22E-35	2.23E-33	68.7378
ENSG00000152223	EPG5	1.6663	5.65E-35	2.41E-33	68.6588
ENSG00000131759	RARA	2.3489	5.99E-35	2.54E-33	68.6015
ENSG00000064012	CASP8	1.7379	6.11E-35	2.59E-33	68.5818
ENSG00000225828	FAM229A	2.4069	6.15E-35	2.60E-33	68.5749
ENSG00000130856	ZNF236	1.8906	6.22E-35	2.62E-33	68.5646
ENSG00000141568	FOXK2	1.6299	6.47E-35	2.72E-33	68.5249
ENSG00000262246	CORO7	2.7068	6.89E-35	2.90E-33	68.4617
ENSG00000165102	HGSNAT	2.0900	6.92E-35	2.90E-33	68.4577
ENSG00000166747	AP1G1	1.9048	7.20E-35	3.01E-33	68.4192
ENSG00000087365	SF3B2	1.5475	7.61E-35	3.18E-33	68.3636
ENSG00000136807	CDK9	1.6212	8.22E-35	3.42E-33	68.2878
ENSG00000139620	KANSL2	1.6221	9.08E-35	3.77E-33	68.1885
ENSG00000153094	BCL2L11	2.4693	1.05E-34	4.34E-33	68.0477
ENSG00000156875	MFSD14A	1.7986	1.11E-34	4.60E-33	67.9876
ENSG00000167548	KMT2D	1.6840	1.13E-34	4.67E-33	67.9704
ENSG00000102317	RBM3	1.5775	1.18E-34	4.88E-33	67.9245
ENSG00000182831	C16orf72	1.5877	1.24E-34	5.09E-33	67.8799
ENSG00000165527	ARF6	1.7238	1.26E-34	5.17E-33	67.8617
ENSG00000129460	NGDN	1.6573	1.27E-34	5.20E-33	67.8551
ENSG00000112282	MED23	1.7304	1.35E-34	5.52E-33	67.7936
ENSG00000162972	MAIP1	1.5328	1.37E-34	5.60E-33	67.7775
ENSG00000179454	KLHL28	1.9204	1.57E-34	6.40E-33	67.6427
ENSG00000155508	CNOT8	1.4879	1.67E-34	6.76E-33	67.5861
ENSG00000123130	ACOT9	1.6342	1.82E-34	7.36E-33	67.4988
ENSG00000078804	TP53INP2	2.5544	1.83E-34	7.40E-33	67.4922
ENSG00000011007	ELOA	1.5665	2.60E-34	1.05E-32	67.1438
ENSG00000124193	SRSF6	1.5725	2.81E-34	1.13E-32	67.0676
ENSG00000101391	CDK5RAP1	1.5391	2.95E-34	1.18E-32	67.0196
ENSG00000196498	NCOR2	1.8026	3.08E-34	1.23E-32	66.9757
ENSG00000158161	EYA3	1.6062	3.19E-34	1.28E-32	66.9407
ENSG00000055483	USP36	1.6067	3.21E-34	1.28E-32	66.9353
ENSG00000142661	MYOM3	1.5339	3.22E-34	1.28E-32	66.9317
ENSG00000143368	SF3B4	1.7312	3.35E-34	1.33E-32	66.8928
ENSG00000184083	FAM120C	1.8178	3.54E-34	1.40E-32	66.8391
ENSG00000166889	PATL1	1.6259	3.79E-34	1.50E-32	66.7700
ENSG00000099783	HNRNPM	1.4732	3.92E-34	1.55E-32	66.7383
ENSG00000114391	RPL24	0.5863	4.05E-34	1.59E-32	66.7061
ENSG00000163930	BAP1	1.6503	4.11E-34	1.61E-32	66.6917
ENSG00000182481	KPNA2	1.8892	4.25E-34	1.67E-32	66.6576

Gene ID	Gene Symbol	FC	P.Value	adj.P.Val	B
ENSG00000100461	RBM23	1.5491	4.48E-34	1.75E-32	66.6048
ENSG00000136521	NDUFB5	0.5466	4.83E-34	1.89E-32	66.5299
ENSG00000163714	U2SURP	1.5989	5.13E-34	2.00E-32	66.4709
ENSG00000131778	CHD1L	1.8180	5.62E-34	2.18E-32	66.3809
ENSG00000131725	WDR44	1.7247	5.68E-34	2.20E-32	66.3697
ENSG00000184675	AMER1	1.4948	6.05E-34	2.34E-32	66.3076
ENSG00000122359	ANXA11	2.1972	6.14E-34	2.37E-32	66.2923
ENSG00000101057	MYBL2	2.2350	6.23E-34	2.40E-32	66.2786
ENSG00000164587	RPS14	0.5461	7.62E-34	2.93E-32	66.0784
ENSG00000116984	MTR	1.7822	8.32E-34	3.19E-32	65.9918
ENSG00000172661	WASHC2C	1.6440	9.96E-34	3.82E-32	65.8127
ENSG00000115464	USP34	1.5939	1.03E-33	3.93E-32	65.7816
ENSG00000131626	PPFIA1	1.6817	1.06E-33	4.04E-32	65.7524
ENSG00000224531	SMIM13	1.6018	1.07E-33	4.09E-32	65.7385
ENSG00000165169	DYNLT3	1.8439	1.08E-33	4.10E-32	65.7325
ENSG00000117362	APH1A	1.6220	1.13E-33	4.28E-32	65.6891
ENSG00000120798	NR2C1	1.6427	1.20E-33	4.54E-32	65.6278
ENSG00000100483	VCPKMT	1.8882	1.23E-33	4.64E-32	65.6045
ENSG00000164104	HMGB2	2.0651	1.23E-33	4.64E-32	65.6034
ENSG00000085999	RAD54L	1.8395	1.31E-33	4.94E-32	65.5394
ENSG00000105143	SLC1A6	1.2593	1.34E-33	5.02E-32	65.5206
ENSG00000011566	MAP4K3	2.0483	1.36E-33	5.10E-32	65.5041
ENSG00000127328	RAB3IP	1.7577	1.36E-33	5.10E-32	65.5019
ENSG00000116809	ZBTB17	1.7150	1.37E-33	5.13E-32	65.4935
ENSG00000052749	RRP12	2.4555	1.72E-33	6.40E-32	65.2716
ENSG00000135945	REV1	1.6044	1.74E-33	6.46E-32	65.2608
ENSG00000169682	SPNS1	1.5925	1.81E-33	6.70E-32	65.2226
ENSG00000111707	SUDS3	1.5108	1.83E-33	6.77E-32	65.2100
ENSG00000179632	MAF1	1.6363	1.90E-33	7.02E-32	65.1719
ENSG00000183054	RGPD6	2.6038	1.92E-33	7.07E-32	65.1632
ENSG00000108406	DHX40	1.6925	1.93E-33	7.11E-32	65.1566
ENSG00000161013	MGAT4B	1.8804	1.94E-33	7.11E-32	65.1540
ENSG00000102218	RP2	1.6558	2.01E-33	7.38E-32	65.1146
ENSG00000079387	SENP1	1.5304	2.02E-33	7.41E-32	65.1095
ENSG00000148516	ZEB1	1.7537	2.17E-33	7.93E-32	65.0403
ENSG00000119280	C1orf198	2.0534	2.29E-33	8.35E-32	64.9871
ENSG00000143387	CTSK	1.9591	2.31E-33	8.41E-32	64.9774
ENSG00000075415	SLC25A3	1.4046	2.38E-33	8.66E-32	64.9470
ENSG00000078747	ITCH	1.5540	2.56E-33	9.30E-32	64.8747
ENSG00000143702	CEP170	1.6809	2.97E-33	1.07E-31	64.7300
ENSG00000182149	IST1	1.9196	2.98E-33	1.08E-31	64.7266
ENSG00000186566	GPATCH8	1.6270	3.12E-33	1.13E-31	64.6790
ENSG00000140829	DHX38	1.8139	3.37E-33	1.21E-31	64.6036
ENSG00000116580	GON4L	1.6467	3.61E-33	1.30E-31	64.5358
ENSG00000177169	ULK1	1.9988	3.96E-33	1.42E-31	64.4440
ENSG00000162402	USP24	1.9296	4.13E-33	1.48E-31	64.4016
ENSG00000102007	PLP2	1.8942	4.24E-33	1.51E-31	64.3759
ENSG00000170144	HNRNPA3	1.4534	4.54E-33	1.62E-31	64.3087
ENSG00000168556	ING2	1.9849	4.64E-33	1.65E-31	64.2869
ENSG00000153561	RMND5A	1.6034	4.65E-33	1.65E-31	64.2842
ENSG00000179833	SERTAD2	2.0178	4.94E-33	1.75E-31	64.2254
ENSG00000076685	NT5C2	1.5957	5.13E-33	1.81E-31	64.1875
ENSG00000101557	USP14	1.7006	5.37E-33	1.90E-31	64.1412
ENSG00000174871	CNIH2	1.9006	5.98E-33	2.11E-31	64.0348

Gene ID	Gene Symbol	FC	P.Value	adj.P.Val	B
ENSG00000160201	U2AF1	1.5788	6.08E-33	2.14E-31	64.0178
ENSG00000119737	GPR75	1.4472	7.11E-33	2.49E-31	63.8638
ENSG00000126070	AGO3	1.4924	7.49E-33	2.62E-31	63.8121
ENSG00000118816	CCNI	1.5318	8.23E-33	2.88E-31	63.7181
ENSG00000025293	PHF20	1.4798	8.27E-33	2.89E-31	63.7138
ENSG00000166685	COG1	1.6811	8.30E-33	2.89E-31	63.7103
ENSG00000064995	TAF11	1.4873	8.65E-33	3.01E-31	63.6693
ENSG00000085721	RRN3	1.5362	9.33E-33	3.24E-31	63.5934
ENSG00000069431	ABCC9	1.4810	1.01E-32	3.52E-31	63.5105
ENSG00000151208	DLG5	2.6999	1.05E-32	3.63E-31	63.4770
ENSG00000171988	JMJD1C	2.4698	1.10E-32	3.79E-31	63.4331
ENSG00000095787	WAC	1.4744	1.15E-32	3.98E-31	63.3826
ENSG00000160789	LMNA	3.5607	1.25E-32	4.29E-31	63.3058
ENSG00000179021	C3orf38	1.6141	1.27E-32	4.37E-31	63.2868
ENSG00000263874	LINC00672	1.5844	1.35E-32	4.64E-31	63.2252
ENSG00000159202	UBE2Z	1.5965	1.38E-32	4.72E-31	63.2053
ENSG00000174106	LEMD3	1.5862	1.38E-32	4.73E-31	63.2026
ENSG00000166377	ATP9B	1.5929	1.49E-32	5.06E-31	63.1324
ENSG00000166676	TVP23A	1.3917	1.59E-32	5.42E-31	63.0626
ENSG00000163346	PBXIP1	2.3546	1.67E-32	5.68E-31	63.0141
ENSG00000143815	LBR	1.8939	1.70E-32	5.76E-31	62.9991
ENSG00000162814	SPATA17	1.7453	1.75E-32	5.91E-31	62.9724
ENSG00000024048	UBR2	1.7084	1.78E-32	6.03E-31	62.9507
ENSG00000121774	KHDRBS1	1.4687	1.94E-32	6.54E-31	62.8677
ENSG00000130703	OSBPL2	1.4866	1.99E-32	6.69E-31	62.8442
ENSG00000159377	PSMB4	1.5091	2.02E-32	6.78E-31	62.8285
ENSG00000124091	GCNT7	1.2067	2.03E-32	6.82E-31	62.8215
ENSG00000197818	SLC9A8	1.6453	2.09E-32	6.98E-31	62.7961
ENSG00000104164	BLOC1S6	1.5693	2.22E-32	7.42E-31	62.7328
ENSG00000115762	PLEKHB2	1.6867	2.22E-32	7.42E-31	62.7324
ENSG00000143924	EML4	1.7151	2.24E-32	7.45E-31	62.7259
ENSG00000157954	WIPI2	1.7113	2.36E-32	7.86E-31	62.6721
ENSG00000170871	KIAA0232	1.5886	2.40E-32	7.95E-31	62.6586
ENSG00000166783	MARF1	1.6672	2.43E-32	8.04E-31	62.6461
ENSG00000136758	YME1L1	1.5045	2.61E-32	8.63E-31	62.5733
ENSG00000204138	PHACTR4	1.4852	2.84E-32	9.39E-31	62.4884
ENSG00000040199	PHLPP2	2.4071	2.95E-32	9.73E-31	62.4513
ENSG00000196843	ARID5A	2.8418	3.00E-32	9.87E-31	62.4356
ENSG00000143324	XPR1	1.7386	3.32E-32	1.09E-30	62.3343
ENSG00000136636	KCTD3	1.9779	3.40E-32	1.11E-30	62.3126
ENSG00000198909	MAP3K3	1.6420	3.43E-32	1.12E-30	62.3014
ENSG00000111696	NT5DC3	1.6663	3.57E-32	1.17E-30	62.2626
ENSG00000163788	SNRK	1.5996	3.62E-32	1.18E-30	62.2479
ENSG00000100439	ABHD4	2.0116	4.02E-32	1.31E-30	62.1458
ENSG00000075413	MARK3	1.5619	4.60E-32	1.49E-30	62.0125
ENSG00000170540	ARL6IP1	1.6411	4.70E-32	1.53E-30	61.9894
ENSG00000151725	CENPU	2.2160	4.77E-32	1.54E-30	61.9759
ENSG00000139718	SETD1B	1.6096	5.45E-32	1.76E-30	61.8443
ENSG00000147526	TACC1	2.8336	5.46E-32	1.76E-30	61.8408
ENSG00000180998	GPR137C	1.3972	5.51E-32	1.77E-30	61.8327
ENSG00000116679	IVNS1ABP	1.8239	6.19E-32	1.99E-30	61.7168
ENSG00000110046	ATG2A	2.1482	6.36E-32	2.04E-30	61.6898
ENSG00000119969	HELLS	1.9900	6.40E-32	2.05E-30	61.6843
ENSG00000249715	FER1L5	1.2947	6.45E-32	2.07E-30	61.6757

Gene ID	Gene Symbol	FC	P.Value	adj.P.Val	B
ENSG00000077254	USP33	1.6251	6.67E-32	2.13E-30	61.6435
ENSG00000259305	ZHX1-C8orf76	1.4671	6.82E-32	2.18E-30	61.6207
ENSG00000126003	PLAGL2	1.7273	7.15E-32	2.28E-30	61.5741
ENSG00000069956	MAPK6	1.6519	7.83E-32	2.49E-30	61.4848
ENSG00000154370	TRIM11	1.6424	8.58E-32	2.72E-30	61.3936
ENSG00000205937	RNPS1	1.4474	8.81E-32	2.79E-30	61.3669
ENSG00000119285	HEATR1	1.7599	9.47E-32	3.00E-30	61.2952
ENSG00000104973	MED25	1.6180	1.02E-31	3.21E-30	61.2251
ENSG00000133997	MED6	1.5900	1.05E-31	3.32E-30	61.1907
ENSG00000197329	PELI1	3.1153	1.08E-31	3.39E-30	61.1681
ENSG00000056097	ZFR	1.4475	1.09E-31	3.43E-30	61.1530
ENSG00000196081	ZNF724	1.4027	1.10E-31	3.45E-30	61.1457
ENSG00000116977	LGALS8	1.8613	1.13E-31	3.55E-30	61.1179
ENSG00000111450	STX2	1.8063	1.36E-31	4.26E-30	60.9352
ENSG00000115211	EIF2B4	1.4757	1.42E-31	4.44E-30	60.8915
ENSG00000013725	CD6	1.7525	1.47E-31	4.57E-30	60.8616
ENSG00000011114	BTBD7	1.4809	1.54E-31	4.80E-30	60.8104
ENSG00000113368	LMNB1	2.0490	1.57E-31	4.88E-30	60.7929
ENSG00000049245	VAMP3	1.5455	1.60E-31	4.95E-30	60.7779
ENSG00000144426	NBEAL1	1.5115	1.61E-31	4.97E-30	60.7713
ENSG00000123360	PDE1B	1.9661	1.72E-31	5.31E-30	60.7048
ENSG00000120063	GNA13	1.7862	1.81E-31	5.58E-30	60.6530
ENSG00000133641	C12orf29	1.4948	1.86E-31	5.72E-30	60.6270
ENSG00000101365	IDH3B	1.4855	1.97E-31	6.06E-30	60.5683
ENSG00000108061	SHOC2	1.5681	2.08E-31	6.39E-30	60.5144
ENSG00000116539	ASH1L	1.6761	2.33E-31	7.14E-30	60.4024
ENSG00000204410	MSH5	2.0244	2.39E-31	7.30E-30	60.3787
ENSG00000164031	DNAJB14	1.5978	2.45E-31	7.47E-30	60.3544
ENSG00000131876	SNRPA1	1.6517	2.47E-31	7.54E-30	60.3438
ENSG00000013293	SLC7A14	1.5164	2.49E-31	7.59E-30	60.3363
ENSG00000130779	CLIP1	1.8091	2.51E-31	7.62E-30	60.3298
ENSG00000151929	BAG3	2.3795	2.54E-31	7.71E-30	60.3174
ENSG00000153006	SREK1IP1	1.5248	2.65E-31	8.04E-30	60.2737
ENSG00000178209	PLEC	2.1851	3.20E-31	9.69E-30	60.0875
ENSG00000127334	DYRK2	2.0700	3.22E-31	9.72E-30	60.0822
ENSG00000198646	NCOA6	1.4866	3.46E-31	1.04E-29	60.0098
ENSG00000104064	GABPB1	1.8613	3.97E-31	1.19E-29	59.8754
ENSG00000180228	PRKRA	1.4758	4.07E-31	1.22E-29	59.8507
ENSG00000165219	GAPVD1	1.5951	4.31E-31	1.29E-29	59.7933
ENSG00000198952	SMG5	1.6082	4.31E-31	1.29E-29	59.7941
ENSG00000166224	SGPL1	1.6354	4.35E-31	1.30E-29	59.7845
ENSG00000143442	POGZ	1.7149	4.38E-31	1.31E-29	59.7768
ENSG00000135108	FBXO21	1.5174	4.45E-31	1.33E-29	59.7617
ENSG00000143093	STRIP1	1.7056	4.62E-31	1.37E-29	59.7249
ENSG00000173588	CEP83	1.6745	4.67E-31	1.39E-29	59.7134
ENSG00000103264	FBXO31	1.8117	5.09E-31	1.51E-29	59.6286
ENSG00000135476	ESPL1	1.9670	5.40E-31	1.60E-29	59.5703
ENSG00000070961	ATP2B1	2.3498	5.45E-31	1.61E-29	59.5611
ENSG00000181222	POLR2A	1.8123	5.64E-31	1.67E-29	59.5260
ENSG00000221944	TIGD1	1.5288	5.69E-31	1.68E-29	59.5182
ENSG00000088930	XRN2	1.4118	5.99E-31	1.76E-29	59.4672
ENSG00000206418	RAB12	1.7154	6.30E-31	1.85E-29	59.4163
ENSG00000141298	SSH2	1.9061	6.43E-31	1.89E-29	59.3970
ENSG00000101199	ARFGAP1	1.7872	6.55E-31	1.92E-29	59.3788

Gene ID	Gene Symbol	FC	P.Value	adj.P.Val	B
ENSG00000172466	ZNF24	1.6327	6.68E-31	1.95E-29	59.3592
ENSG00000102078	SLC25A14	1.6337	6.84E-31	2.00E-29	59.3356
ENSG00000094841	UPRT	1.4049	7.01E-31	2.05E-29	59.3105
ENSG00000112031	MTRF1L	1.6093	7.24E-31	2.11E-29	59.2789
ENSG00000002834	LASP1	1.6845	7.37E-31	2.14E-29	59.2609
ENSG00000120616	EPC1	1.7035	7.49E-31	2.17E-29	59.2452
ENSG00000034152	MAP2K3	2.1876	7.65E-31	2.22E-29	59.2236
ENSG00000135365	PHF21A	1.6528	8.10E-31	2.34E-29	59.1679
ENSG00000105821	DNAJC2	1.5168	8.35E-31	2.41E-29	59.1371
ENSG00000247746	USP51	1.4151	8.36E-31	2.41E-29	59.1360
ENSG00000172493	AFF1	1.7946	8.65E-31	2.49E-29	59.1020
ENSG00000114098	ARMC8	1.4634	8.80E-31	2.53E-29	59.0850
ENSG00000213085	CFAP45	2.6673	9.29E-31	2.67E-29	59.0313
ENSG00000107959	PITRM1	1.7551	9.42E-31	2.70E-29	59.0181
ENSG00000147133	TAF1	1.6402	1.02E-30	2.93E-29	58.9374
ENSG00000160062	ZBTB8A	1.5765	1.05E-30	3.01E-29	58.9072
ENSG00000107771	CCSER2	1.6167	1.21E-30	3.46E-29	58.7670
ENSG00000164758	MED30	1.7737	1.25E-30	3.57E-29	58.7352
ENSG00000100345	MYH9	1.9079	1.31E-30	3.73E-29	58.6908
ENSG00000167978	SRRM2	1.9422	1.34E-30	3.80E-29	58.6705
ENSG00000089818	NECAP1	1.5453	1.42E-30	4.03E-29	58.6113
ENSG00000107651	SEC23IP	1.4898	1.43E-30	4.03E-29	58.6067
ENSG00000188529	SRSF10	1.7935	1.42E-30	4.03E-29	58.6075
ENSG00000115935	WIPF1	1.6537	1.47E-30	4.16E-29	58.5737
ENSG00000052126	PLEKHA5	1.8854	1.52E-30	4.28E-29	58.5455
ENSG00000170185	USP38	1.5735	1.52E-30	4.28E-29	58.5433
ENSG00000127184	COX7C	0.6735	1.53E-30	4.31E-29	58.5351
ENSG00000168066	SF1	1.6885	1.66E-30	4.66E-29	58.4566
ENSG00000125835	SNRPB	1.5308	1.76E-30	4.93E-29	58.3983
ENSG00000083168	KAT6A	1.6585	1.83E-30	5.13E-29	58.3589
ENSG00000133247	KMT5C	1.9607	1.84E-30	5.14E-29	58.3548
ENSG00000213516	RBMXL1	1.5530	1.85E-30	5.17E-29	58.3479
ENSG00000138814	PPP3CA	1.7279	1.88E-30	5.25E-29	58.3318
ENSG00000091651	ORC6	1.6898	1.90E-30	5.28E-29	58.3244
ENSG00000160710	ADAR	1.7727	1.99E-30	5.54E-29	58.2746
ENSG00000113810	SMC4	1.9866	2.00E-30	5.56E-29	58.2698
ENSG00000047056	WDR37	1.4949	2.02E-30	5.59E-29	58.2635
ENSG00000042429	MED17	1.6317	2.06E-30	5.69E-29	58.2434
ENSG00000213079	SCAF8	1.6083	2.06E-30	5.70E-29	58.2411
ENSG00000111445	RFC5	1.7535	2.15E-30	5.92E-29	58.2017
ENSG00000141232	TOB1	2.2157	2.31E-30	6.36E-29	58.1292
ENSG00000172071	EIF2AK3	2.2690	2.32E-30	6.37E-29	58.1263
ENSG00000135966	TGFBRAP1	1.4485	2.34E-30	6.40E-29	58.1177
ENSG00000142541	RPL13A	0.5844	2.33E-30	6.40E-29	58.1181
ENSG00000177565	TBL1XR1	1.8044	2.50E-30	6.84E-29	58.0518
ENSG00000181544	FANCB	1.7312	2.50E-30	6.84E-29	58.0490
ENSG00000152422	XRCC4	0.4826	2.57E-30	7.00E-29	58.0246
ENSG00000140750	ARHGAP17	2.0523	2.57E-30	7.01E-29	58.0225
ENSG00000165943	MOAP1	1.5330	2.80E-30	7.62E-29	57.9377
ENSG00000166233	ARIH1	1.6367	2.94E-30	7.99E-29	57.8899
ENSG00000177689	MAGEB10	1.1821	3.00E-30	8.14E-29	57.8705
ENSG00000053438	NNAT	1.5284	3.08E-30	8.36E-29	57.8420
ENSG00000121350	PYROXD1	1.5727	3.12E-30	8.44E-29	57.8314
ENSG00000164548	TRA2A	1.5381	3.25E-30	8.78E-29	57.7905

Gene ID	Gene Symbol	FC	P.Value	adj.P.Val	B
ENSG00000175203	DCTN2	1.4765	3.28E-30	8.85E-29	57.7813
ENSG00000100836	PABPN1	1.4559	3.29E-30	8.88E-29	57.7771
ENSG00000180098	TRNAU1AP	1.5907	3.38E-30	9.10E-29	57.7511
ENSG00000087470	DNM1L	1.6311	3.53E-30	9.49E-29	57.7077
ENSG00000174953	DHX36	1.4916	3.74E-30	1.00E-28	57.6512
ENSG00000126581	BECN1	1.3787	3.76E-30	1.01E-28	57.6450
ENSG00000217128	FNIP1	1.7184	3.77E-30	1.01E-28	57.6429
ENSG00000135968	GCC2	1.6647	3.91E-30	1.05E-28	57.6063
ENSG00000179051	RCC2	1.8241	3.95E-30	1.05E-28	57.5979
ENSG00000214367	HAUS3	1.8102	4.04E-30	1.08E-28	57.5747
ENSG00000100241	SBF1	1.6574	4.05E-30	1.08E-28	57.5709
ENSG00000108256	NUFIP2	1.6921	4.08E-30	1.08E-28	57.5649
ENSG00000177733	HNRNPA0	1.4172	4.19E-30	1.11E-28	57.5374
ENSG00000100731	PCNX1	1.8517	4.36E-30	1.15E-28	57.4999
ENSG00000124201	ZNFX1	1.8510	4.49E-30	1.19E-28	57.4697
ENSG00000143971	ETAA1	1.5612	4.49E-30	1.19E-28	57.4696
ENSG00000160271	RALGDS	1.8617	4.56E-30	1.20E-28	57.4547
ENSG00000172943	PHF8	1.5521	4.62E-30	1.22E-28	57.4416
ENSG00000147166	ITGB1BP2	1.5060	4.68E-30	1.23E-28	57.4286
ENSG00000140575	IQGAP1	1.6032	4.78E-30	1.26E-28	57.4069
ENSG00000092820	EZR	2.4891	5.03E-30	1.32E-28	57.3577
ENSG00000109458	GAB1	1.8608	5.03E-30	1.32E-28	57.3570
ENSG00000135976	ANKRD36	1.9094	5.10E-30	1.34E-28	57.3427
ENSG00000018610	STEEP1	1.5048	5.13E-30	1.34E-28	57.3368
ENSG00000158941	CCAR2	1.6552	5.13E-30	1.34E-28	57.3374
ENSG00000234545	FAM133B	1.4913	5.30E-30	1.38E-28	57.3055
ENSG00000078061	ARAF	1.4880	5.41E-30	1.41E-28	57.2855
ENSG00000117479	SLC19A2	1.8710	5.72E-30	1.49E-28	57.2289
ENSG00000114120	SLC25A36	1.7346	5.88E-30	1.53E-28	57.2019
ENSG00000072364	AFF4	1.7220	6.17E-30	1.60E-28	57.1539
ENSG00000169155	ZBTB43	2.1971	6.84E-30	1.77E-28	57.0519
ENSG00000171824	EXOSC10	1.4935	6.88E-30	1.78E-28	57.0468
ENSG00000109046	WSB1	1.7552	7.02E-30	1.81E-28	57.0262
ENSG00000165806	CASP7	1.6894	7.12E-30	1.83E-28	57.0127
ENSG00000112081	SRSF3	1.4388	7.17E-30	1.84E-28	57.0064
ENSG00000096384	HSP90AB1	1.5659	7.27E-30	1.87E-28	56.9924
ENSG00000075539	FRYL	1.5808	7.54E-30	1.93E-28	56.9562
ENSG00000106948	AKNA	2.5792	8.14E-30	2.09E-28	56.8805
ENSG00000119689	DLST	1.5702	8.77E-30	2.24E-28	56.8060
ENSG00000136942	RPL35	0.6120	8.77E-30	2.24E-28	56.8059
ENSG00000068400	GRIPAP1	1.5902	1.02E-29	2.59E-28	56.6598
ENSG00000118482	PHF3	1.5958	1.05E-29	2.67E-28	56.6311
ENSG00000173698	ADGRG2	1.1115	1.05E-29	2.68E-28	56.6235
ENSG00000127993	RBM48	1.4843	1.10E-29	2.80E-28	56.5811
ENSG00000008128	CDK11A	1.7224	1.11E-29	2.82E-28	56.5714
ENSG00000147065	MSN	1.7581	1.13E-29	2.85E-28	56.5585
ENSG00000175197	DDIT3	2.5616	1.17E-29	2.96E-28	56.5196
ENSG00000007923	DNAJC11	1.4977	1.20E-29	3.03E-28	56.4962
ENSG00000181555	SETD2	1.5327	1.36E-29	3.44E-28	56.3694
ENSG00000011258	MBTD1	1.5596	1.44E-29	3.62E-28	56.3179
ENSG00000166170	BAG5	1.5106	1.45E-29	3.65E-28	56.3090
ENSG00000165699	TSC1	1.6394	1.50E-29	3.77E-28	56.2740
ENSG00000248329	APELA	1.3169	1.52E-29	3.82E-28	56.2612
ENSG00000089094	KDM2B	1.5905	1.54E-29	3.87E-28	56.2454

Gene ID	Gene Symbol	FC	P.Value	adj.P.Val	B
ENSG00000101972	STAG2	1.7073	1.54E-29	3.87E-28	56.2466
ENSG00000116954	RRAGC	1.7909	1.58E-29	3.96E-28	56.2210
ENSG00000094631	HDAC6	1.7728	1.78E-29	4.46E-28	56.1026
ENSG00000114982	KANSL3	1.5137	1.79E-29	4.48E-28	56.0963
ENSG00000166860	ZBTB39	1.4980	1.84E-29	4.59E-28	56.0700
ENSG00000148339	SLC25A25	1.8929	1.88E-29	4.67E-28	56.0514
ENSG00000139636	LMBR1L	1.9432	1.95E-29	4.84E-28	56.0160
ENSG00000082512	TRAF5	2.4126	1.97E-29	4.89E-28	56.0047
ENSG00000101412	E2F1	2.3525	2.01E-29	4.99E-28	55.9824
ENSG00000198746	GPATCH3	1.4401	2.07E-29	5.12E-28	55.9553
ENSG00000153922	CHD1	1.7429	2.10E-29	5.20E-28	55.9397
ENSG00000164338	UTP15	1.6220	2.17E-29	5.36E-28	55.9082
ENSG00000108840	HDAC5	1.5043	2.18E-29	5.38E-28	55.9036
ENSG00000163040	CCDC74A	1.7958	2.20E-29	5.42E-28	55.8948
ENSG00000124228	DDX27	1.4493	2.28E-29	5.60E-28	55.8611
ENSG00000173120	KDM2A	1.7202	2.31E-29	5.66E-28	55.8482
ENSG00000143437	ARNT	1.7456	2.34E-29	5.75E-28	55.8321
ENSG00000163596	ICA1L	1.5627	2.37E-29	5.80E-28	55.8216
ENSG00000157837	SPPL3	1.4525	2.53E-29	6.20E-28	55.7551
ENSG00000105856	HBP1	1.7885	2.56E-29	6.24E-28	55.7461
ENSG00000228727	SAPCD1	1.9978	2.64E-29	6.43E-28	55.7156
ENSG00000132664	POLR3F	1.5172	2.66E-29	6.48E-28	55.7070
ENSG00000111581	NUP107	1.6151	2.87E-29	6.99E-28	55.6307
ENSG00000144566	RAB5A	1.4271	2.90E-29	7.05E-28	55.6201
ENSG00000242028	HYPK	1.9538	2.92E-29	7.09E-28	55.6133
ENSG00000143549	TPM3	1.7151	3.01E-29	7.29E-28	55.5848
ENSG00000066027	PPP2R5A	1.6576	3.09E-29	7.49E-28	55.5571
ENSG00000179094	PER1	3.4955	3.14E-29	7.58E-28	55.5435
ENSG00000197279	ZNF165	2.2684	3.36E-29	8.13E-28	55.4734
ENSG00000142867	BCL10	1.6183	3.38E-29	8.16E-28	55.4678
ENSG00000213639	PPP1CB	1.5262	3.40E-29	8.18E-28	55.4643
ENSG00000163635	ATXN7	1.7282	3.60E-29	8.66E-28	55.4070
ENSG00000160584	SIK3	1.7759	3.71E-29	8.90E-28	55.3777
ENSG00000100225	FBXO7	1.4516	3.84E-29	9.21E-28	55.3424
ENSG00000143374	TARS2	1.5932	3.84E-29	9.21E-28	55.3419
ENSG00000066777	ARFGEF1	1.4365	4.13E-29	9.88E-28	55.2706
ENSG00000125386	FAM193A	1.5530	4.15E-29	9.92E-28	55.2659
ENSG00000100330	MTMR3	1.7353	4.17E-29	9.97E-28	55.2597
ENSG00000107581	EIF3A	1.4365	4.31E-29	1.03E-27	55.2269
ENSG00000105372	RPS19	0.5822	4.40E-29	1.05E-27	55.2069
ENSG00000126107	HECTD3	1.7583	4.44E-29	1.05E-27	55.1996
ENSG00000107854	TNKS2	1.5015	4.48E-29	1.06E-27	55.1907
ENSG00000125841	NRSN2	2.1724	4.58E-29	1.09E-27	55.1669
ENSG00000147144	CCDC120	1.7001	4.68E-29	1.11E-27	55.1466
ENSG00000162775	RBM15	1.5896	4.69E-29	1.11E-27	55.1443
ENSG00000153292	ADGRF1	1.3027	4.72E-29	1.12E-27	55.1378
ENSG00000115307	AUP1	1.4205	5.26E-29	1.24E-27	55.0312
ENSG00000138593	SECISBP2L	1.9532	5.48E-29	1.29E-27	54.9895
ENSG00000174231	PRPF8	1.5569	5.59E-29	1.32E-27	54.9702
ENSG00000101290	CDS2	1.6619	5.63E-29	1.32E-27	54.9626
ENSG00000112531	QKI	1.8005	5.67E-29	1.33E-27	54.9560
ENSG00000108179	PPIF	1.7508	5.69E-29	1.33E-27	54.9533
ENSG00000165732	DDX21	1.5775	5.73E-29	1.34E-27	54.9459
ENSG00000156853	ZNF689	1.5017	6.36E-29	1.49E-27	54.8426

Gene ID	Gene Symbol	FC	P.Value	adj.P.Val	B
ENSG00000135241	PNPLA8	1.7097	6.71E-29	1.57E-27	54.7900
ENSG00000130640	TUBGCP2	1.4458	6.78E-29	1.58E-27	54.7784
ENSG00000141867	BRD4	1.5907	6.82E-29	1.59E-27	54.7725
ENSG00000101558	VAPA	1.4904	6.88E-29	1.60E-27	54.7642
ENSG00000132294	EFR3A	1.5576	7.23E-29	1.68E-27	54.7149
ENSG00000211460	TSN	1.4945	7.75E-29	1.80E-27	54.6462
ENSG00000213625	LEPROT	1.5096	8.02E-29	1.86E-27	54.6121
ENSG00000162231	NXF1	1.8981	8.08E-29	1.87E-27	54.6050
ENSG00000105325	FZR1	1.6916	8.31E-29	1.92E-27	54.5770
ENSG00000109118	PHF12	1.5783	9.06E-29	2.09E-27	54.4923
ENSG00000135837	CEP350	1.6496	9.40E-29	2.17E-27	54.4551
ENSG00000136504	KAT7	1.5344	9.73E-29	2.24E-27	54.4206
ENSG00000165997	ARL5B	1.7867	9.73E-29	2.24E-27	54.4214
ENSG00000173166	RAPH1	2.6677	1.04E-28	2.38E-27	54.3598
ENSG00000100109	TFIP11	1.5426	1.16E-28	2.65E-27	54.2511
ENSG00000173039	RELA	1.7971	1.17E-28	2.68E-27	54.2399
ENSG00000255152	MSH5-SAPCD1	1.9365	1.27E-28	2.91E-27	54.1584
ENSG00000173575	CHD2	1.7314	1.29E-28	2.94E-27	54.1454
ENSG00000117592	PRDX6	1.4944	1.30E-28	2.98E-27	54.1308
ENSG00000119725	ZNF410	1.4673	1.37E-28	3.12E-27	54.0841
ENSG00000075856	SART3	1.5557	1.38E-28	3.14E-27	54.0758
ENSG00000085224	ATRX	1.5866	1.44E-28	3.28E-27	54.0322
ENSG00000196550	FAM72A	2.1013	1.46E-28	3.32E-27	54.0196
ENSG00000185864	NPIPB4	1.9723	1.56E-28	3.54E-27	53.9534
ENSG00000170469	SPATA24	0.6252	1.60E-28	3.63E-27	53.9296
ENSG00000149923	PPP4C	1.4423	1.62E-28	3.66E-27	53.9182
ENSG00000101421	CHMP4B	1.8423	1.64E-28	3.72E-27	53.9021
ENSG00000152382	TADA1	1.5796	1.72E-28	3.89E-27	53.8571
ENSG00000104312	RIPK2	1.8271	1.75E-28	3.96E-27	53.8373
ENSG00000009780	FAM76A	1.5095	1.86E-28	4.20E-27	53.7771
ENSG00000136492	BRIP1	1.6292	1.87E-28	4.22E-27	53.7715
ENSG00000196338	NLGN3	1.4728	2.05E-28	4.61E-27	53.6822
ENSG00000111711	GOLT1B	1.5078	2.08E-28	4.67E-27	53.6688
ENSG00000175305	CCNE2	2.1172	2.12E-28	4.75E-27	53.6503
ENSG00000142534	RPS11	0.6384	2.16E-28	4.84E-27	53.6322
ENSG00000119402	FBXW2	1.4519	2.18E-28	4.88E-27	53.6227
ENSG00000087460	GNAS	1.5759	2.24E-28	5.02E-27	53.5926
ENSG00000101448	EPPIN	1.3371	2.25E-28	5.02E-27	53.5924
ENSG00000153310	CYRIB	1.6430	2.44E-28	5.44E-27	53.5111
ENSG00000170832	USP32	1.6384	2.69E-28	5.99E-27	53.4138
ENSG00000183888	SRARP	1.1103	2.70E-28	6.01E-27	53.4099
ENSG00000041988	THAP3	1.6083	2.72E-28	6.04E-27	53.4041
ENSG00000103540	CCP110	1.5786	2.75E-28	6.11E-27	53.3909
ENSG00000243284	VSIG8	1.4563	2.78E-28	6.17E-27	53.3797
ENSG00000183735	TBK1	1.4280	2.81E-28	6.23E-27	53.3703
ENSG00000120539	MASTL	1.6365	2.94E-28	6.51E-27	53.3245
ENSG00000025156	HSF2	1.5889	3.01E-28	6.65E-27	53.3023
ENSG00000197380	DACT3	1.1987	3.03E-28	6.68E-27	53.2965
ENSG00000138346	DNA2	1.6153	3.15E-28	6.95E-27	53.2571
ENSG00000114978	MOB1A	1.4448	3.37E-28	7.43E-27	53.1890
ENSG00000053254	FOXN3	1.8026	3.38E-28	7.43E-27	53.1875
ENSG00000243716	NPIPB5	1.9767	3.38E-28	7.44E-27	53.1862
ENSG00000139645	ANKRD52	1.5661	3.60E-28	7.90E-27	53.1245
ENSG00000048405	ZNF800	1.5994	3.64E-28	7.99E-27	53.1129

Gene ID	Gene Symbol	FC	P.Value	adj.P.Val	B
ENSG00000147439	BIN3	1.5895	3.66E-28	8.03E-27	53.1071
ENSG00000166024	R3HCC1L	1.4092	3.75E-28	8.20E-27	53.0846
ENSG00000170802	FOXN2	1.6689	3.86E-28	8.43E-27	53.0565
ENSG00000213551	DNAJC9	1.4257	3.92E-28	8.55E-27	53.0407
ENSG00000204396	VWA7	2.2142	4.08E-28	8.90E-27	52.9998
ENSG00000181773	GPR3	1.7513	4.45E-28	9.68E-27	52.9152
ENSG00000138757	G3BP2	1.5308	4.49E-28	9.76E-27	52.9061
ENSG00000135018	UBQLN1	1.4199	4.53E-28	9.85E-27	52.8960
ENSG00000143507	DUSP10	3.1216	4.93E-28	1.07E-26	52.8126
ENSG00000170836	PPM1D	1.4955	5.09E-28	1.10E-26	52.7809
ENSG00000100811	YY1	1.3930	5.11E-28	1.11E-26	52.7774
ENSG00000178772	CPN2	1.2274	5.19E-28	1.12E-26	52.7613
ENSG00000102738	MRPS31	0.5251	6.05E-28	1.31E-26	52.6106
ENSG00000144597	EAF1	1.6006	6.14E-28	1.32E-26	52.5955
ENSG00000153201	RANBP2	1.6549	6.74E-28	1.45E-26	52.5029
ENSG00000058056	USP13	1.9166	7.17E-28	1.54E-26	52.4424
ENSG00000185122	HSF1	1.4670	7.63E-28	1.64E-26	52.3806
ENSG00000176927	EFCAB5	1.4160	7.89E-28	1.69E-26	52.3472
ENSG00000140941	MAP1LC3B	1.9381	8.10E-28	1.74E-26	52.3205
ENSG00000138434	ITPRID2	1.4794	8.21E-28	1.76E-26	52.3081
ENSG00000213281	NRAS	1.6455	8.94E-28	1.91E-26	52.2231
ENSG00000123607	TTC21B	1.4952	9.27E-28	1.98E-26	52.1880
ENSG00000196396	PTPN1	1.9913	9.93E-28	2.12E-26	52.1191
ENSG00000029993	HMGB3	2.9092	9.95E-28	2.12E-26	52.1169
ENSG00000180667	YOD1	2.0908	1.02E-27	2.17E-26	52.0924
ENSG00000133059	DSTYK	1.5253	1.10E-27	2.33E-26	52.0217
ENSG00000135930	EIF4E2	1.3464	1.13E-27	2.41E-26	51.9883
ENSG00000166507	NDST2	1.4734	1.14E-27	2.43E-26	51.9782
ENSG00000006451	RALA	1.5249	1.19E-27	2.52E-26	51.9398
ENSG00000010017	RANBP9	1.4211	1.22E-27	2.59E-26	51.9146
ENSG00000164916	FOXK1	1.5690	1.25E-27	2.65E-26	51.8906
ENSG00000139625	MAP3K12	1.9034	1.25E-27	2.65E-26	51.8893
ENSG00000135999	EPC2	1.5015	1.29E-27	2.72E-26	51.8626
ENSG00000008083	JARID2	1.6134	1.31E-27	2.75E-26	51.8483
ENSG00000126883	NUP214	1.4312	1.32E-27	2.78E-26	51.8366
ENSG00000167553	TUBA1C	1.7228	1.34E-27	2.82E-26	51.8207
ENSG00000182606	TRAK1	1.6925	1.41E-27	2.96E-26	51.7734
ENSG00000127311	HELB	1.7416	1.49E-27	3.13E-26	51.7184
ENSG00000176225	RTTN	1.8099	1.50E-27	3.14E-26	51.7112
ENSG00000162734	PEA15	2.1939	1.51E-27	3.16E-26	51.7039
ENSG00000107372	ZFAND5	1.8637	1.54E-27	3.21E-26	51.6874
ENSG00000155096	AZIN1	1.5106	1.96E-27	4.11E-26	51.4432
ENSG00000122882	ECD	1.5042	2.01E-27	4.20E-26	51.4192
ENSG00000138018	SELENOI	1.4269	2.09E-27	4.35E-26	51.3834
ENSG00000170889	RPS9	0.6516	2.09E-27	4.36E-26	51.3806
ENSG00000167522	ANKRD11	1.6496	2.10E-27	4.37E-26	51.3765
ENSG00000013441	CLK1	1.8206	2.18E-27	4.54E-26	51.3388
ENSG00000198399	ITSN2	1.5433	2.24E-27	4.64E-26	51.3154
ENSG00000165119	HNRNPK	1.3272	2.51E-27	5.21E-26	51.1999
ENSG00000158019	BABAM2	1.4402	2.52E-27	5.22E-26	51.1960
ENSG00000170289	CNGB3	1.4062	2.61E-27	5.39E-26	51.1636
ENSG00000049540	ELN	1.4223	2.70E-27	5.57E-26	51.1298
ENSG00000165650	PDZD8	1.5432	2.72E-27	5.61E-26	51.1217
ENSG00000186591	UBE2H	1.6369	2.78E-27	5.74E-26	51.0987

Gene ID	Gene Symbol	FC	P.Value	adj.P.Val	B
ENSG00000004059	ARF5	1.4869	2.93E-27	6.04E-26	51.0469
ENSG00000117036	ETV3	1.9442	2.95E-27	6.08E-26	51.0391
ENSG00000163466	ARPC2	1.4605	3.03E-27	6.24E-26	51.0126
ENSG00000204590	GNL1	1.4590	3.11E-27	6.39E-26	50.9877
ENSG00000110713	NUP98	1.5437	3.16E-27	6.48E-26	50.9730
ENSG00000162642	C1orf52	1.6282	3.19E-27	6.54E-26	50.9622
ENSG00000119682	AREL1	1.5547	3.44E-27	7.04E-26	50.8880
ENSG00000239857	GET4	1.5440	3.54E-27	7.24E-26	50.8593
ENSG00000132819	RBM38	2.2484	3.61E-27	7.37E-26	50.8412
ENSG00000141452	RMC1	1.5164	3.84E-27	7.84E-26	50.7787
ENSG00000001497	LAS1L	1.4066	3.85E-27	7.85E-26	50.7771
ENSG00000072401	UBE2D1	1.6569	4.08E-27	8.29E-26	50.7203
ENSG00000157764	BRAF	1.4951	4.07E-27	8.29E-26	50.7213
ENSG00000121101	TEX14	2.5637	4.12E-27	8.37E-26	50.7097
ENSG00000183032	SLC25A21	1.6745	4.25E-27	8.62E-26	50.6799
ENSG00000082701	GSK3B	1.4345	4.33E-27	8.77E-26	50.6603
ENSG00000170379	TCAF2	2.0740	4.33E-27	8.77E-26	50.6600
ENSG00000133640	LRRIQ1	1.1890	4.34E-27	8.79E-26	50.6574
ENSG00000170807	LMOD2	1.2583	4.48E-27	9.05E-26	50.6269
ENSG00000040487	SLC66A1	1.6173	4.61E-27	9.31E-26	50.5980
ENSG00000100206	DMC1	1.9605	4.76E-27	9.60E-26	50.5663
ENSG00000163781	TOPBP1	1.6050	4.87E-27	9.79E-26	50.5446
ENSG00000196369	SRGAP2B	2.0711	4.87E-27	9.79E-26	50.5444
ENSG00000168214	RBPJ	1.5407	4.96E-27	9.98E-26	50.5249
ENSG00000100982	PCIF1	1.4807	5.49E-27	1.10E-25	50.4252
ENSG00000168878	SFTPB	1.1974	5.56E-27	1.12E-25	50.4123
ENSG00000100220	RTCB	1.5869	5.65E-27	1.13E-25	50.3960
ENSG00000089154	GCN1	1.6757	5.68E-27	1.14E-25	50.3917
ENSG00000134375	TIMM17A	1.5689	6.08E-27	1.21E-25	50.3250
ENSG00000137634	NXPE4	0.2701	6.33E-27	1.26E-25	50.2847
ENSG00000182827	ACBD3	1.6511	6.78E-27	1.35E-25	50.2169
ENSG00000063601	MTMR1	1.6364	6.92E-27	1.38E-25	50.1956
ENSG00000164111	ANXA5	1.5686	6.95E-27	1.38E-25	50.1917
ENSG00000137275	RIPK1	1.5399	7.64E-27	1.52E-25	50.0977
ENSG00000101752	MIB1	1.7383	7.80E-27	1.55E-25	50.0774
ENSG00000188895	MSL1	1.5090	8.00E-27	1.59E-25	50.0520
ENSG00000215301	DDX3X	1.7992	8.35E-27	1.66E-25	50.0101
ENSG00000145216	FIP1L1	1.4872	8.66E-27	1.71E-25	49.9739
ENSG00000198492	YTHDF2	1.4171	8.97E-27	1.77E-25	49.9391
ENSG00000155252	PI4K2A	1.5328	9.18E-27	1.81E-25	49.9157
ENSG00000076944	STXBP2	1.5395	9.35E-27	1.85E-25	49.8978
ENSG00000125812	GZF1	1.7500	9.42E-27	1.86E-25	49.8903
ENSG00000187605	TET3	1.4816	9.67E-27	1.91E-25	49.8641
ENSG00000125447	GGA3	1.5158	9.79E-27	1.93E-25	49.8528
ENSG00000254087	LYN	1.7010	9.95E-27	1.96E-25	49.8360
ENSG00000185019	UBOX5	1.4251	9.98E-27	1.96E-25	49.8329
ENSG00000186260	MRTFB	1.5930	1.04E-26	2.04E-25	49.7934
ENSG00000138032	PPM1B	1.4400	1.09E-26	2.13E-25	49.7468
ENSG00000134283	PPHLN1	1.3513	1.16E-26	2.27E-25	49.6849
ENSG00000123562	MORF4L2	1.4942	1.23E-26	2.40E-25	49.6300
ENSG00000158023	CFAP251	1.3428	1.27E-26	2.48E-25	49.5968
ENSG00000128191	DGCR8	1.5501	1.28E-26	2.50E-25	49.5857
ENSG00000178607	ERN1	2.0364	1.37E-26	2.67E-25	49.5207
ENSG00000176208	ATAD5	1.5585	1.39E-26	2.70E-25	49.5081

Gene ID	Gene Symbol	FC	P.Value	adj.P.Val	B
ENSG00000078674	PCM1	1.7108	1.40E-26	2.72E-25	49.4977
ENSG00000110700	RPS13	0.6216	1.45E-26	2.82E-25	49.4618
ENSG00000204344	STK19	1.4937	1.49E-26	2.89E-25	49.4384
ENSG00000145780	FEM1C	2.0661	1.49E-26	2.90E-25	49.4341
ENSG00000164776	PHKG1	1.7435	1.75E-26	3.39E-25	49.2783
ENSG00000143393	PI4KB	1.5067	1.78E-26	3.44E-25	49.2606
ENSG00000012983	MAP4K5	1.5352	1.79E-26	3.45E-25	49.2565
ENSG00000100796	PPP4R3A	1.4807	1.85E-26	3.57E-25	49.2236
ENSG00000154920	EME1	1.8498	1.89E-26	3.64E-25	49.2019
ENSG00000134698	AGO4	1.8347	1.99E-26	3.84E-25	49.1476
ENSG00000198315	ZKSCAN8	1.6563	2.07E-26	3.99E-25	49.1094
ENSG00000133858	ZFC3H1	1.5812	2.08E-26	4.01E-25	49.1037
ENSG00000079313	REXO1	1.6264	2.18E-26	4.19E-25	49.0588
ENSG00000116670	MAD2L2	1.5972	2.21E-26	4.24E-25	49.0458
ENSG00000178385	PLEKHM3	1.5768	2.38E-26	4.57E-25	48.9711
ENSG00000137821	LRRC49	1.4322	2.45E-26	4.69E-25	48.9438
ENSG00000134759	ELP2	1.4818	2.46E-26	4.70E-25	48.9418
ENSG00000130055	GDPD2	1.1325	2.54E-26	4.86E-25	48.9069
ENSG00000153975	ZUP1	1.8643	2.57E-26	4.91E-25	48.8958
ENSG00000124222	STX16	1.5731	2.58E-26	4.92E-25	48.8938
ENSG00000178982	EIF3K	0.6982	2.60E-26	4.95E-25	48.8868
ENSG00000117569	PTBP2	1.8283	2.84E-26	5.41E-25	48.7964
ENSG00000121892	PDS5A	1.4072	2.85E-26	5.43E-25	48.7930
ENSG00000049768	FOXP3	1.1598	2.86E-26	5.43E-25	48.7914
ENSG00000186468	RPS23	0.5947	2.93E-26	5.56E-25	48.7675
ENSG00000197021	EOLA2	1.6816	2.95E-26	5.59E-25	48.7604
ENSG00000108582	CPD	1.6254	3.14E-26	5.94E-25	48.6991
ENSG00000143398	PIP5K1A	1.5604	3.15E-26	5.96E-25	48.6952
ENSG00000105849	POLR1F	1.6312	3.29E-26	6.23E-25	48.6511
ENSG00000147536	GINS4	1.6288	3.31E-26	6.26E-25	48.6451
ENSG00000165782	PIP4P1	1.6232	3.52E-26	6.64E-25	48.5854
ENSG00000165914	TTC7B	1.6562	3.56E-26	6.71E-25	48.5732
ENSG00000092850	TEKT2	1.2080	3.59E-26	6.76E-25	48.5657
ENSG00000085511	MAP3K4	1.6538	3.65E-26	6.87E-25	48.5485
ENSG00000204599	TRIM39	1.5657	3.69E-26	6.94E-25	48.5378
ENSG00000141522	ARHGDIA	1.4931	3.86E-26	7.24E-25	48.4947
ENSG00000155275	TRMT44	1.4890	3.89E-26	7.30E-25	48.4857
ENSG00000100297	MCM5	1.8521	3.96E-26	7.42E-25	48.4678
ENSG00000085840	ORC1	1.5626	4.01E-26	7.50E-25	48.4565
ENSG00000143318	CASQ1	1.4538	4.02E-26	7.52E-25	48.4532
ENSG00000171241	SHCBP1	2.2062	4.11E-26	7.68E-25	48.4316
ENSG00000120438	TCP1	1.4713	4.28E-26	7.98E-25	48.3921
ENSG00000119685	TTLL5	1.5276	4.33E-26	8.07E-25	48.3805
ENSG00000008838	MED24	1.6042	4.35E-26	8.10E-25	48.3751
ENSG00000063177	RPL18	0.6074	4.40E-26	8.19E-25	48.3631
ENSG00000108671	PSMD11	1.4961	4.52E-26	8.40E-25	48.3373
ENSG00000145495	MARCHF6	1.5666	4.54E-26	8.43E-25	48.3331
ENSG00000086712	TXLNG	1.4437	4.76E-26	8.84E-25	48.2857
ENSG00000165861	ZFYVE1	1.6399	5.47E-26	1.01E-24	48.1486
ENSG00000167258	CDK12	1.5181	5.57E-26	1.03E-24	48.1298
ENSG00000136541	ERMN	2.6971	5.78E-26	1.07E-24	48.0943
ENSG00000069345	DNAJA2	1.4310	5.82E-26	1.08E-24	48.0870
ENSG00000165972	CCDC38	1.4346	5.83E-26	1.08E-24	48.0856
ENSG00000065559	MAP2K4	1.4414	5.84E-26	1.08E-24	48.0842

Gene ID	Gene Symbol	FC	P.Value	adj.P.Val	B
ENSG00000187325	TAF9B	1.5649	6.03E-26	1.11E-24	48.0519
ENSG00000100056	ESS2	1.5002	6.06E-26	1.12E-24	48.0465
ENSG00000173171	MTX1	1.5738	6.35E-26	1.17E-24	48.0004
ENSG00000118900	UBN1	1.4672	6.37E-26	1.17E-24	47.9983
ENSG00000160218	TRAPPC10	1.5207	6.41E-26	1.18E-24	47.9918
ENSG00000115750	TAF1B	1.4862	6.78E-26	1.24E-24	47.9363
ENSG00000060749	QSER1	1.4920	7.39E-26	1.35E-24	47.8502
ENSG00000133131	MORC4	1.5393	8.12E-26	1.49E-24	47.7566
ENSG00000177981	ASB8	1.4454	8.56E-26	1.57E-24	47.7047
ENSG00000144395	CCDC150	1.7226	9.65E-26	1.76E-24	47.5859
ENSG00000171681	ATF7IP	1.6238	9.70E-26	1.77E-24	47.5812
ENSG00000102858	MGRN1	1.4576	9.75E-26	1.78E-24	47.5758
ENSG00000102606	ARHGEF7	1.5870	9.95E-26	1.81E-24	47.5558
ENSG00000162413	KLHL21	1.7917	1.02E-25	1.86E-24	47.5313
ENSG00000102984	ZNF821	2.1465	1.02E-25	1.86E-24	47.5302
ENSG00000178440	TIMM23B-AGAP6	1.4546	1.03E-25	1.86E-24	47.5260
ENSG00000138448	ITGAV	1.6494	1.04E-25	1.88E-24	47.5141
ENSG00000185022	MAFF	2.9890	1.04E-25	1.88E-24	47.5148
ENSG00000130816	DNMT1	1.7120	1.13E-25	2.04E-24	47.4312
ENSG00000196683	TOMM7	0.6905	1.15E-25	2.07E-24	47.4157
ENSG00000182899	RPL35A	0.6432	1.16E-25	2.09E-24	47.4052
ENSG00000070444	MNT	1.5965	1.24E-25	2.24E-24	47.3371
ENSG00000087152	ATXN7L3	1.4866	1.26E-25	2.28E-24	47.3188
ENSG00000143258	USP21	1.5970	1.28E-25	2.31E-24	47.3043
ENSG00000139266	MARCHF9	1.7076	1.38E-25	2.49E-24	47.2300
ENSG00000160783	PMF1	1.4882	1.41E-25	2.53E-24	47.2141
ENSG00000155330	C16orf87	1.6752	1.41E-25	2.53E-24	47.2128
ENSG00000070495	JMJD6	1.6334	1.42E-25	2.55E-24	47.2019
ENSG00000154124	OTULIN	1.6229	1.43E-25	2.57E-24	47.1953
ENSG00000160753	RUSC1	1.7627	1.45E-25	2.61E-24	47.1801
ENSG00000168175	MAPK1IP1L	1.5764	1.51E-25	2.70E-24	47.1445
ENSG00000152104	PTPN14	1.1438	1.51E-25	2.71E-24	47.1403
ENSG00000000419	DPM1	1.4082	1.60E-25	2.86E-24	47.0844
ENSG00000197121	PGAP1	1.5954	1.66E-25	2.96E-24	47.0508
ENSG00000143845	ETNK2	1.3222	1.74E-25	3.10E-24	47.0019
ENSG00000144357	UBR3	1.5865	1.80E-25	3.21E-24	46.9671
ENSG00000129351	ILF3	1.4704	1.87E-25	3.34E-24	46.9286
ENSG00000079277	MKNK1	1.5162	2.02E-25	3.60E-24	46.8539
ENSG00000173451	THAP2	1.7598	2.05E-25	3.65E-24	46.8381
ENSG00000084093	REST	1.5169	2.16E-25	3.83E-24	46.7885
ENSG00000143786	CNIH3	1.4504	2.29E-25	4.07E-24	46.7285
ENSG00000139697	SBNO1	1.4727	2.39E-25	4.23E-24	46.6889
ENSG00000173276	ZBTB21	1.8085	2.49E-25	4.41E-24	46.6466
ENSG00000147044	CASK	1.9722	2.50E-25	4.42E-24	46.6449
ENSG00000156639	ZFAND3	1.5016	2.50E-25	4.42E-24	46.6432
ENSG00000104081	BMF	3.4494	2.52E-25	4.45E-24	46.6358
ENSG00000011275	RNF216	1.5531	2.60E-25	4.58E-24	46.6051
ENSG00000167595	PROSER3	1.5526	2.63E-25	4.63E-24	46.5942
ENSG00000241973	PI4KA	1.5311	2.84E-25	4.99E-24	46.5185
ENSG00000133193	FAM104A	1.3799	2.93E-25	5.15E-24	46.4874
ENSG00000120029	ARMH3	1.3558	2.97E-25	5.22E-24	46.4731
ENSG00000169629	RGPD8	1.8651	3.09E-25	5.42E-24	46.4341
ENSG00000124688	MAD2L1BP	1.5613	3.10E-25	5.44E-24	46.4301
ENSG00000131773	KHDRBS3	2.2829	3.12E-25	5.47E-24	46.4231

Gene ID	Gene Symbol	FC	P.Value	adj.P.Val	B
ENSG00000145592	RPL37	0.6156	3.18E-25	5.57E-24	46.4052
ENSG00000069696	DRD4	1.4901	3.25E-25	5.68E-24	46.3843
ENSG00000144036	EXOC6B	1.4730	3.38E-25	5.90E-24	46.3454
ENSG00000060339	CCAR1	1.4138	3.46E-25	6.04E-24	46.3215
ENSG00000101577	LPIN2	1.7082	3.63E-25	6.34E-24	46.2734
ENSG00000221983	UBA52	0.6898	3.77E-25	6.57E-24	46.2369
ENSG00000166105	GLB1L3	1.2948	3.80E-25	6.61E-24	46.2301
ENSG00000188738	FSIP2	1.6979	3.84E-25	6.68E-24	46.2189
ENSG00000167378	IRGQ	1.5914	3.88E-25	6.73E-24	46.2093
ENSG00000164164	OTUD4	1.5329	4.17E-25	7.24E-24	46.1369
ENSG00000198900	TOP1	1.5316	4.21E-25	7.30E-24	46.1273
ENSG00000116127	ALMS1	1.5557	4.24E-25	7.34E-24	46.1216
ENSG00000114648	KLHL18	1.5549	4.36E-25	7.54E-24	46.0928
ENSG00000165475	CRYL1	0.4917	4.36E-25	7.54E-24	46.0934
ENSG00000103549	RNF40	1.4268	4.43E-25	7.65E-24	46.0777
ENSG00000132549	VPS13B	1.5009	4.53E-25	7.83E-24	46.0543
ENSG00000177485	ZBTB33	1.4953	4.54E-25	7.83E-24	46.0531
ENSG00000120334	CENPL	1.6046	4.57E-25	7.88E-24	46.0458
ENSG00000141560	FN3KRP	1.4818	4.86E-25	8.36E-24	45.9856
ENSG00000103035	PSMD7	1.5335	4.92E-25	8.46E-24	45.9737
ENSG00000160094	ZNF362	1.7286	4.99E-25	8.57E-24	45.9602
ENSG00000107443	CCNJ	1.4267	5.01E-25	8.60E-24	45.9559
ENSG00000143367	TUFT1	1.8735	5.05E-25	8.66E-24	45.9479
ENSG00000137216	TMEM63B	1.5744	5.35E-25	9.16E-24	45.8908
ENSG00000188342	GTF2F2	0.5343	5.41E-25	9.26E-24	45.8797
ENSG00000156858	PRR14	1.5221	5.49E-25	9.39E-24	45.8644
ENSG00000063978	RNF4	1.4152	5.57E-25	9.51E-24	45.8512
ENSG00000182095	TNRC18	1.6083	5.63E-25	9.62E-24	45.8394
ENSG00000178502	KLHL11	1.6460	5.97E-25	1.02E-23	45.7821
ENSG00000138069	RAB1A	1.4370	6.08E-25	1.04E-23	45.7634
ENSG00000158526	TSR2	1.4436	6.08E-25	1.04E-23	45.7643
ENSG00000112038	OPRM1	1.2191	6.16E-25	1.05E-23	45.7511
ENSG00000198723	TEX45	1.7709	6.37E-25	1.08E-23	45.7171
ENSG00000124251	TP53TG5	1.3709	6.45E-25	1.10E-23	45.7049
ENSG00000162244	RPL29	0.6067	7.39E-25	1.25E-23	45.5700
ENSG00000089693	MLF2	1.4620	7.43E-25	1.26E-23	45.5650
ENSG00000163257	DCAF16	1.5322	7.50E-25	1.27E-23	45.5554
ENSG00000176153	GPX2	1.2687	7.70E-25	1.30E-23	45.5301
ENSG00000174238	PITPNA	1.5218	7.84E-25	1.33E-23	45.5120
ENSG00000072786	STK10	1.6055	8.02E-25	1.35E-23	45.4903
ENSG00000206560	ANKRD28	2.2154	8.02E-25	1.35E-23	45.4891
ENSG00000115073	ACTR1B	1.4367	8.39E-25	1.41E-23	45.4448
ENSG00000163633	C4orf36	1.5241	8.77E-25	1.48E-23	45.4011
ENSG00000165244	ZNF367	1.8316	8.79E-25	1.48E-23	45.3988
ENSG00000173976	RAX2	1.1058	9.01E-25	1.52E-23	45.3741
ENSG00000163867	ZMYM6	1.4134	9.06E-25	1.52E-23	45.3689
ENSG00000165733	BMS1	1.3682	9.15E-25	1.53E-23	45.3597
ENSG00000103496	STX4	1.5522	9.32E-25	1.56E-23	45.3406
ENSG00000162714	ZNF496	1.5587	9.63E-25	1.61E-23	45.3090
ENSG00000165359	INTS6L	1.7943	9.71E-25	1.63E-23	45.3004
ENSG00000006432	MAP3K9	1.6168	1.02E-24	1.71E-23	45.2474
ENSG00000100592	DAAM1	1.9091	1.03E-24	1.71E-23	45.2462
ENSG00000115306	SPTBN1	1.7329	1.03E-24	1.73E-23	45.2381
ENSG00000204469	PRRC2A	1.4787	1.15E-24	1.92E-23	45.1303

Gene ID	Gene Symbol	FC	P.Value	adj.P.Val	B
ENSG00000185658	BRWD1	1.4638	1.16E-24	1.93E-23	45.1271
ENSG00000133056	PIK3C2B	1.9194	1.16E-24	1.94E-23	45.1213
ENSG00000111704	NANOG	1.1293	1.17E-24	1.94E-23	45.1199
ENSG00000081154	PCNP	1.3062	1.17E-24	1.94E-23	45.1164
ENSG00000168676	KCTD19	1.4281	1.18E-24	1.96E-23	45.1080
ENSG00000137075	RNF38	1.4941	1.19E-24	1.98E-23	45.0986
ENSG00000167900	TK1	2.1502	1.20E-24	1.99E-23	45.0903
ENSG00000055044	NOP58	1.4364	1.21E-24	2.01E-23	45.0794
ENSG00000169991	IFFO2	2.2801	1.25E-24	2.06E-23	45.0542
ENSG00000112312	GMNN	1.7156	1.26E-24	2.09E-23	45.0387
ENSG00000075702	WDR62	1.6578	1.27E-24	2.10E-23	45.0340
ENSG00000108055	SMC3	1.5206	1.32E-24	2.17E-23	44.9982
ENSG00000179361	ARID3B	1.8682	1.33E-24	2.20E-23	44.9860
ENSG00000122970	IFT81	1.5297	1.36E-24	2.24E-23	44.9687
ENSG00000164938	TP53INP1	2.0916	1.45E-24	2.38E-23	44.9058
ENSG00000083845	RPS5	0.6103	1.46E-24	2.40E-23	44.8990
ENSG00000175166	PSMD2	1.4386	1.46E-24	2.40E-23	44.8972
ENSG00000138688	KIAA1109	1.5804	1.50E-24	2.47E-23	44.8670
ENSG00000196428	TSC22D2	1.6617	1.54E-24	2.52E-23	44.8445
ENSG00000109466	KLHL2	1.6207	1.54E-24	2.53E-23	44.8409
ENSG00000172782	FADS6	1.3039	1.57E-24	2.57E-23	44.8255
ENSG00000156030	MIDEAS	1.7880	1.62E-24	2.65E-23	44.7933
ENSG00000136937	NCBP1	1.3940	1.67E-24	2.73E-23	44.7640
ENSG00000187514	PTMA	1.4131	1.68E-24	2.75E-23	44.7554
ENSG00000096401	CDC5L	1.3906	1.69E-24	2.76E-23	44.7499
ENSG00000273274	ZBTB8B	1.0595	1.71E-24	2.79E-23	44.7377
ENSG00000123338	NCKAP1L	1.5097	1.77E-24	2.88E-23	44.7056
ENSG00000161904	LEMD2	1.5323	1.77E-24	2.88E-23	44.7058
ENSG00000056586	RC3H2	1.4909	1.82E-24	2.95E-23	44.6804
ENSG00000178719	GRINA	1.6715	1.83E-24	2.97E-23	44.6727
ENSG00000141385	AFG3L2	1.4558	1.85E-24	3.01E-23	44.6598
ENSG00000185883	ATP6V0C	1.5044	1.86E-24	3.01E-23	44.6582
ENSG00000183684	ALYREF	1.4250	1.92E-24	3.12E-23	44.6233
ENSG00000143320	CRABP2	1.8863	1.97E-24	3.19E-23	44.6011
ENSG00000198060	MARCHF5	1.4370	1.97E-24	3.19E-23	44.5981
ENSG00000142731	PLK4	2.0266	2.05E-24	3.32E-23	44.5589
ENSG00000170677	SOCS6	2.0370	2.09E-24	3.37E-23	44.5431
ENSG00000178691	SUZ12	1.4556	2.09E-24	3.37E-23	44.5426
ENSG00000160224	AIRE	1.1311	2.11E-24	3.40E-23	44.5321
ENSG00000150990	DHX37	1.4493	2.13E-24	3.43E-23	44.5231
ENSG00000103066	PLA2G15	1.9063	2.14E-24	3.44E-23	44.5192
ENSG00000106635	BCL7B	1.4129	2.17E-24	3.49E-23	44.5044
ENSG00000140093	SERPINA10	1.2134	2.17E-24	3.49E-23	44.5025
ENSG00000131043	AAR2	1.4097	2.19E-24	3.51E-23	44.4974
ENSG00000197694	SPTAN1	1.6972	2.26E-24	3.62E-23	44.4648
ENSG00000168884	TNIP2	1.6959	2.34E-24	3.74E-23	44.4315
ENSG00000079335	CDC14A	2.0839	2.38E-24	3.81E-23	44.4119
ENSG00000164967	RPP25L	0.5647	2.39E-24	3.82E-23	44.4101
ENSG00000101166	PRELID3B	1.3420	2.43E-24	3.89E-23	44.3916
ENSG00000108861	DUSP3	1.5764	2.51E-24	4.01E-23	44.3609
ENSG00000163125	RPRD2	1.6367	2.58E-24	4.12E-23	44.3320
ENSG00000100441	KHNYN	1.5971	2.62E-24	4.18E-23	44.3182
ENSG00000174501	ANKRD36C	1.8139	2.63E-24	4.19E-23	44.3146
ENSG00000152518	ZFP36L2	2.2571	2.66E-24	4.24E-23	44.3019

Gene ID	Gene Symbol	FC	P.Value	adj.P.Val	B
ENSG00000171530	TBCA	0.6898	2.87E-24	4.56E-23	44.2281
ENSG00000174444	RPL4	0.6401	2.98E-24	4.73E-23	44.1908
ENSG00000163636	PSMD6	1.3375	3.04E-24	4.83E-23	44.1707
ENSG00000084676	NCOA1	1.5436	3.05E-24	4.83E-23	44.1684
ENSG00000100099	HPS4	1.5667	3.13E-24	4.97E-23	44.1409
ENSG00000205189	ZBTB10	2.4928	3.22E-24	5.09E-23	44.1149
ENSG00000124942	AHNAK	4.2018	3.27E-24	5.17E-23	44.0991
ENSG00000176438	SYNE3	1.9775	3.32E-24	5.24E-23	44.0849
ENSG00000134533	RERG	1.4381	3.41E-24	5.38E-23	44.0584
ENSG00000144713	RPL32	0.6444	3.43E-24	5.41E-23	44.0517
ENSG00000156804	FBXO32	2.7041	3.46E-24	5.46E-23	44.0415
ENSG00000164944	VIRMA	1.4225	3.49E-24	5.49E-23	44.0353
ENSG00000197183	NOL4L	2.0844	3.49E-24	5.49E-23	44.0347
ENSG00000185088	RPS27L	0.6608	3.51E-24	5.53E-23	44.0275
ENSG00000109475	RPL34	0.6190	3.53E-24	5.55E-23	44.0224
ENSG00000204930	FAM221B	1.3115	3.61E-24	5.67E-23	44.0004
ENSG00000184007	PTP4A2	1.4196	3.77E-24	5.92E-23	43.9572
ENSG00000242689	CNTF	1.5198	3.80E-24	5.95E-23	43.9507
ENSG00000214717	ZBED1	1.6141	4.01E-24	6.28E-23	43.8972
ENSG00000143674	MAP3K21	2.0249	4.05E-24	6.34E-23	43.8872
ENSG00000153046	CDYL	1.5517	4.24E-24	6.63E-23	43.8421
ENSG00000260230	FRRS1L	1.0556	4.33E-24	6.77E-23	43.8207
ENSG00000196924	FLNA	3.1829	4.38E-24	6.84E-23	43.8093
ENSG00000168005	SPINDOC	1.5473	4.45E-24	6.95E-23	43.7931
ENSG00000130479	MAP1S	1.6354	4.51E-24	7.03E-23	43.7805
ENSG00000188338	SLC38A3	1.2720	4.56E-24	7.11E-23	43.7684
ENSG00000149179	C11orf49	1.4298	4.63E-24	7.21E-23	43.7539
ENSG00000168036	CTNNB1	1.5509	4.75E-24	7.38E-23	43.7296
ENSG00000130402	ACTN4	1.6544	4.86E-24	7.55E-23	43.7065
ENSG00000100281	HMGXB4	1.5184	5.01E-24	7.78E-23	43.6760
ENSG00000160691	SHC1	1.8352	5.07E-24	7.87E-23	43.6639
ENSG00000173926	MARCHF3	1.8722	5.32E-24	8.24E-23	43.6171
ENSG00000174839	DENND6A	1.4327	5.44E-24	8.42E-23	43.5952
ENSG00000134108	ARL8B	1.4910	5.81E-24	8.99E-23	43.5292
ENSG00000137221	TJAP1	1.4769	5.81E-24	8.99E-23	43.5299
ENSG00000109536	FRG1	1.3821	5.83E-24	9.01E-23	43.5260
ENSG00000108091	CCDC6	2.2571	5.96E-24	9.20E-23	43.5042
ENSG00000079616	KIF22	1.7077	6.27E-24	9.67E-23	43.4546
ENSG00000121797	CCRL2	0.4226	6.35E-24	9.79E-23	43.4414
ENSG00000074695	LMAN1	1.6294	6.60E-24	1.02E-22	43.4031
ENSG00000166401	SERPINB8	2.5184	7.10E-24	1.09E-22	43.3316
ENSG00000121858	TNFSF10	0.3853	7.28E-24	1.12E-22	43.3068
ENSG00000153498	SPACA7	1.2752	7.86E-24	1.21E-22	43.2305
ENSG00000163362	INAVA	2.4558	8.12E-24	1.25E-22	43.1986
ENSG00000139890	REM2	1.7328	8.55E-24	1.31E-22	43.1478
ENSG00000143321	HDGF	1.6938	8.62E-24	1.32E-22	43.1395
ENSG00000165355	FBXO33	1.7919	8.85E-24	1.35E-22	43.1136
ENSG00000141441	GAREM1	1.9720	8.88E-24	1.36E-22	43.1103
ENSG00000115474	KCNJ13	1.3070	8.98E-24	1.37E-22	43.0986
ENSG00000198689	SLC9A6	1.5214	9.39E-24	1.43E-22	43.0546
ENSG00000163249	CCNYL1	1.5789	9.51E-24	1.45E-22	43.0421
ENSG00000197063	MAFG	1.6269	9.93E-24	1.51E-22	42.9998
ENSG00000105855	ITGB8	1.8403	1.01E-23	1.54E-22	42.9817
ENSG00000124226	RNF114	1.3444	1.05E-23	1.60E-22	42.9451

Gene ID	Gene Symbol	FC	P.Value	adj.P.Val	B
ENSG00000126351	THRA	1.6380	1.10E-23	1.67E-22	42.8995
ENSG00000106682	EIF4H	1.3219	1.11E-23	1.69E-22	42.8885
ENSG00000174748	RPL15	0.6913	1.16E-23	1.76E-22	42.8447
ENSG00000197586	ENTPD6	1.6250	1.23E-23	1.87E-22	42.7845
ENSG00000140396	NCOA2	1.5100	1.26E-23	1.90E-22	42.7666
ENSG00000214491	SEC14L6	1.1276	1.27E-23	1.92E-22	42.7567
ENSG00000113441	LNPEP	1.4762	1.30E-23	1.96E-22	42.7357
ENSG00000120784	ZFP30	1.4727	1.31E-23	1.98E-22	42.7266
ENSG00000115760	BIRC6	1.4676	1.31E-23	1.98E-22	42.7251
ENSG00000108774	RAB5C	1.3914	1.35E-23	2.03E-22	42.6974
ENSG00000168411	RFWD3	1.5447	1.36E-23	2.06E-22	42.6852
ENSG00000197753	LHFPL5	1.4424	1.36E-23	2.06E-22	42.6857
ENSG00000149600	COMMD7	1.4634	1.37E-23	2.06E-22	42.6837
ENSG00000148672	GLUD1	1.3635	1.40E-23	2.11E-22	42.6570
ENSG00000196236	XPNPEP3	1.4290	1.42E-23	2.14E-22	42.6453
ENSG00000175213	ZNF408	1.6242	1.47E-23	2.21E-22	42.6125
ENSG00000127586	CHTF18	1.9026	1.50E-23	2.25E-22	42.5928
ENSG00000148840	PPRC1	1.5723	1.50E-23	2.25E-22	42.5893
ENSG00000214655	ZSWIM8	1.5681	1.50E-23	2.25E-22	42.5897
ENSG00000164134	NAA15	1.4708	1.52E-23	2.28E-22	42.5759
ENSG00000145390	USP53	1.8279	1.53E-23	2.29E-22	42.5705
ENSG00000142197	DOP1B	1.6091	1.54E-23	2.30E-22	42.5675
ENSG00000181938	GINS3	1.5858	1.54E-23	2.30E-22	42.5636
ENSG00000143633	C1orf131	1.5241	1.56E-23	2.34E-22	42.5492
ENSG00000123737	EXOSC9	1.4406	1.62E-23	2.41E-22	42.5174
ENSG00000106328	FSCN3	2.0131	1.65E-23	2.46E-22	42.4981
ENSG00000257315	ZBED6	1.8874	1.68E-23	2.50E-22	42.4794
ENSG00000078246	TULP3	1.5446	1.72E-23	2.55E-22	42.4578
ENSG00000172059	KLF11	2.5717	1.80E-23	2.67E-22	42.4128
ENSG00000185262	UBALD2	2.0418	1.80E-23	2.67E-22	42.4113
ENSG00000184205	TSPYL2	2.0814	1.82E-23	2.70E-22	42.3992
ENSG00000146263	MMS22L	1.6246	1.88E-23	2.79E-22	42.3680
ENSG00000064703	DDX20	1.4172	2.27E-23	3.37E-22	42.1807
ENSG00000157349	DDX19B	1.4821	2.29E-23	3.40E-22	42.1706
ENSG00000164961	WASHC5	1.3919	2.32E-23	3.43E-22	42.1615
ENSG00000100603	SNW1	1.4181	2.32E-23	3.43E-22	42.1590
ENSG00000168137	SETD5	1.5603	2.39E-23	3.53E-22	42.1315
ENSG00000115268	RPS15	0.6530	2.54E-23	3.76E-22	42.0687
ENSG00000025800	KPNA6	1.4148	2.55E-23	3.76E-22	42.0655
ENSG00000054118	THRAP3	1.4921	2.56E-23	3.77E-22	42.0641
ENSG00000141428	C18orf21	1.3516	2.56E-23	3.77E-22	42.0639
ENSG00000163913	IFT122	1.5451	2.56E-23	3.77E-22	42.0615
ENSG00000104848	KCNA7	1.0854	2.67E-23	3.92E-22	42.0216
ENSG00000117758	STX12	1.6132	2.79E-23	4.11E-22	41.9758
ENSG00000187231	SESTD1	2.3539	2.89E-23	4.25E-22	41.9417
ENSG00000197622	CDC42SE1	1.7087	2.93E-23	4.30E-22	41.9295
ENSG00000182541	LIMK2	1.7371	2.96E-23	4.34E-22	41.9197
ENSG00000118492	ADGB	1.2099	3.00E-23	4.39E-22	41.9056
ENSG00000174527	MYO1H	1.2532	3.00E-23	4.39E-22	41.9061
ENSG00000172738	TMEM217	1.3399	3.02E-23	4.42E-22	41.8974
ENSG00000139908	TSSK4	1.6412	3.15E-23	4.60E-22	41.8581
ENSG00000167106	FAM102A	2.1417	3.17E-23	4.63E-22	41.8503
ENSG00000009413	REV3L	1.3949	3.22E-23	4.70E-22	41.8347
ENSG00000148730	EIF4EBP2	1.5053	3.25E-23	4.74E-22	41.8256

Gene ID	Gene Symbol	FC	P.Value	adj.P.Val	B
ENSG00000137154	RPS6	0.6266	3.27E-23	4.77E-22	41.8188
ENSG00000145425	RPS3A	0.6681	3.29E-23	4.79E-22	41.8134
ENSG00000114127	XRN1	1.7052	3.31E-23	4.81E-22	41.8095
ENSG00000108854	SMURF2	1.4316	3.40E-23	4.94E-22	41.7827
ENSG00000066427	ATXN3	1.4219	3.41E-23	4.95E-22	41.7792
ENSG00000138834	MAPK8IP3	1.8950	3.42E-23	4.96E-22	41.7764
ENSG00000101812	H2BW2	1.2561	3.45E-23	5.01E-22	41.7665
ENSG00000105792	CFAP69	1.5053	3.46E-23	5.01E-22	41.7651
ENSG00000146247	PHIP	1.6572	3.56E-23	5.15E-22	41.7366
ENSG00000118193	KIF14	1.5553	3.61E-23	5.22E-22	41.7223
ENSG00000109320	NFKB1	1.7725	3.85E-23	5.57E-22	41.6582
ENSG00000129292	PHF20L1	1.4420	3.90E-23	5.64E-22	41.6445
ENSG00000179750	APOBEC3B	2.4310	3.93E-23	5.68E-22	41.6378
ENSG00000128578	STRIP2	1.4698	3.94E-23	5.69E-22	41.6352
ENSG00000165376	CLDN2	1.2482	4.07E-23	5.87E-22	41.6029
ENSG00000153147	SMARCA5	1.4815	4.10E-23	5.90E-22	41.5966
ENSG00000168769	TET2	1.6394	4.13E-23	5.94E-22	41.5890
ENSG00000149926	TLCD3B	1.4253	4.23E-23	6.08E-22	41.5653
ENSG00000139233	LLPH	1.3342	4.25E-23	6.11E-22	41.5605
ENSG00000159592	GPBP1L1	1.3923	4.30E-23	6.18E-22	41.5488
ENSG00000183856	IQGAP3	1.8039	4.37E-23	6.27E-22	41.5336
ENSG00000105321	CCDC9	1.4495	4.44E-23	6.37E-22	41.5165
ENSG00000155744	FAM126B	1.6169	4.48E-23	6.42E-22	41.5079
ENSG00000160051	IQCC	1.4479	4.52E-23	6.47E-22	41.5001
ENSG00000152217	SETBP1	1.8478	4.55E-23	6.51E-22	41.4933
ENSG00000171943	SRGAP2C	1.7792	4.85E-23	6.93E-22	41.4305
ENSG00000114867	EIF4G1	1.4617	5.04E-23	7.20E-22	41.3923
ENSG00000258366	RTEL1	1.5807	5.28E-23	7.54E-22	41.3454
ENSG00000117139	KDM5B	1.7709	5.38E-23	7.67E-22	41.3280
ENSG00000109762	SNX25	1.5380	5.53E-23	7.88E-22	41.3003
ENSG00000141027	NCOR1	1.4490	5.67E-23	8.08E-22	41.2755
ENSG00000254858	MPV17L2	0.5312	5.71E-23	8.12E-22	41.2693
ENSG00000099331	MYO9B	1.5588	5.71E-23	8.12E-22	41.2682
ENSG00000138182	KIF20B	1.5368	5.75E-23	8.18E-22	41.2610
ENSG00000076201	PTPN23	1.5070	5.83E-23	8.27E-22	41.2487
ENSG00000231256	CFAP97D1	1.0930	5.85E-23	8.30E-22	41.2449
ENSG00000124614	RPS10	0.6933	5.88E-23	8.33E-22	41.2400
ENSG00000183864	TOB2	1.5294	5.94E-23	8.42E-22	41.2290
ENSG00000172318	B3GALT1	1.1227	6.15E-23	8.71E-22	41.1944
ENSG00000130939	UBE4B	1.3797	6.21E-23	8.78E-22	41.1854
ENSG00000182872	RBM10	1.3682	6.25E-23	8.84E-22	41.1789
ENSG00000135597	REPS1	1.4866	6.28E-23	8.87E-22	41.1746
ENSG00000066654	THUMPD1	1.5068	6.75E-23	9.52E-22	41.1027
ENSG00000074657	ZNF532	1.8496	6.75E-23	9.52E-22	41.1030
ENSG00000057657	PRDM1	1.7201	6.76E-23	9.53E-22	41.1012
ENSG00000072864	NDE1	1.5242	6.83E-23	9.62E-22	41.0909
ENSG00000148303	RPL7A	0.6237	7.26E-23	1.02E-21	41.0310
ENSG00000112280	COL9A1	1.1914	7.28E-23	1.02E-21	41.0277
ENSG00000165434	PGM2L1	1.4436	7.45E-23	1.05E-21	41.0059
ENSG00000179988	PSTK	1.5155	7.50E-23	1.05E-21	40.9993
ENSG00000162434	JAK1	1.6372	7.56E-23	1.06E-21	40.9905
ENSG00000089737	DDX24	1.5980	7.71E-23	1.08E-21	40.9720
ENSG00000141646	SMAD4	1.5600	7.73E-23	1.08E-21	40.9685
ENSG00000186335	SLC36A2	1.1372	7.85E-23	1.10E-21	40.9541

Gene ID	Gene Symbol	FC	P.Value	adj.P.Val	B
ENSG00000110497	AMBRA1	1.3745	7.94E-23	1.11E-21	40.9426
ENSG00000132507	EIF5A	1.4121	8.07E-23	1.13E-21	40.9266
ENSG00000169203	NPIPB12	1.8286	8.10E-23	1.13E-21	40.9226
ENSG00000087274	ADD1	1.4732	8.18E-23	1.14E-21	40.9135
ENSG00000164190	NIPBL	1.4779	8.19E-23	1.14E-21	40.9112
ENSG00000076924	XAB2	1.4769	8.21E-23	1.14E-21	40.9095
ENSG00000141738	GRB7	2.6852	8.39E-23	1.17E-21	40.8881
ENSG00000258102	MAP1LC3B2	1.1882	8.39E-23	1.17E-21	40.8877
ENSG00000116062	MSH6	1.6208	8.47E-23	1.18E-21	40.8790
ENSG00000154710	RABGEF1	1.4606	8.50E-23	1.18E-21	40.8744
ENSG00000116852	KIF21B	2.8376	8.64E-23	1.20E-21	40.8586
ENSG00000011523	CEP68	1.6433	8.78E-23	1.22E-21	40.8430
ENSG00000174373	RALGAPA1	1.4971	8.91E-23	1.24E-21	40.8281
ENSG00000170242	USP47	1.4850	9.00E-23	1.25E-21	40.8181
ENSG00000125818	PSMF1	1.4014	9.13E-23	1.26E-21	40.8044
ENSG00000111752	PHC1	1.9382	9.15E-23	1.27E-21	40.8023
ENSG00000161204	ABCF3	1.5277	9.21E-23	1.27E-21	40.7957
ENSG00000158122	PRXL2C	1.7557	9.27E-23	1.28E-21	40.7893
ENSG00000112701	SENP6	1.4258	9.81E-23	1.35E-21	40.7330
ENSG00000090924	PLEKHG2	2.2236	9.92E-23	1.37E-21	40.7222
ENSG00000015153	YAF2	1.5109	1.01E-22	1.39E-21	40.7068
ENSG00000100024	UPB1	1.4846	1.01E-22	1.39E-21	40.7065
ENSG00000152284	TCF7L1	1.4064	1.05E-22	1.44E-21	40.6696
ENSG00000164244	PRRC1	1.4290	1.09E-22	1.50E-21	40.6294
ENSG00000080824	HSP90AA1	1.6264	1.10E-22	1.51E-21	40.6230
ENSG00000109685	NSD2	3.3864	1.15E-22	1.58E-21	40.5776
ENSG00000080298	RFX3	1.4509	1.22E-22	1.68E-21	40.5139
ENSG00000143228	NUF2	2.2821	1.25E-22	1.71E-21	40.4961
ENSG00000038358	EDC4	1.6150	1.55E-22	2.12E-21	40.2838
ENSG00000101191	DIDO1	1.4188	1.58E-22	2.16E-21	40.2640
ENSG00000133398	MED10	1.4104	1.61E-22	2.20E-21	40.2429
ENSG00000139132	FGD4	2.2750	1.62E-22	2.21E-21	40.2391
ENSG00000114796	KLHL24	1.7408	1.62E-22	2.21E-21	40.2379
ENSG00000005981	ASB4	1.1869	1.63E-22	2.22E-21	40.2338
ENSG00000181220	ZNF746	1.4179	1.63E-22	2.22E-21	40.2312
ENSG00000147251	DOCK11	2.3013	1.64E-22	2.23E-21	40.2268
ENSG00000015479	MATR3	1.3303	1.67E-22	2.27E-21	40.2093
ENSG00000167670	CHAF1A	1.6862	1.69E-22	2.31E-21	40.1926
ENSG00000130024	PHF10	1.5255	1.72E-22	2.34E-21	40.1763
ENSG00000174799	CEP135	1.5025	1.72E-22	2.34E-21	40.1751
ENSG00000090060	PAPOLA	1.3963	1.73E-22	2.36E-21	40.1696
ENSG00000184481	FOXO4	1.4764	1.74E-22	2.36E-21	40.1668
ENSG00000069399	BCL3	2.0913	1.77E-22	2.40E-21	40.1491
ENSG00000164327	RICTOR	1.8070	1.78E-22	2.42E-21	40.1419
ENSG00000182446	NPLOC4	1.5089	1.83E-22	2.48E-21	40.1177
ENSG00000121864	ZNF639	1.5412	1.89E-22	2.56E-21	40.0848
ENSG00000171735	CAMTA1	1.3852	2.00E-22	2.70E-21	40.0289
ENSG00000110422	HIPK3	1.5010	2.03E-22	2.75E-21	40.0125
ENSG00000188846	RPL14	0.6693	2.05E-22	2.77E-21	40.0044
ENSG00000198700	IPO9	1.5677	2.14E-22	2.89E-21	39.9607
ENSG00000066739	ATG2B	1.7653	2.15E-22	2.89E-21	39.9587
ENSG00000144228	SPOPL	1.4412	2.20E-22	2.97E-21	39.9339
ENSG00000132670	PTPRA	1.3997	2.31E-22	3.12E-21	39.8845
ENSG00000119950	MXI1	1.6922	2.49E-22	3.35E-21	39.8132

Gene ID	Gene Symbol	FC	P.Value	adj.P.Val	B
ENSG00000153914	SREK1	1.5632	2.68E-22	3.60E-21	39.7404
ENSG00000124571	XPO5	1.5012	2.73E-22	3.66E-21	39.7219
ENSG00000168038	ULK4	1.5058	2.88E-22	3.87E-21	39.6667
ENSG00000115368	WDR75	1.3991	2.89E-22	3.88E-21	39.6653
ENSG00000123240	OPTN	1.7800	3.02E-22	4.05E-21	39.6206
ENSG00000219073	CELA3B	1.4143	3.05E-22	4.09E-21	39.6108
ENSG00000047644	WWC3	1.6514	3.15E-22	4.22E-21	39.5779
ENSG00000158470	B4GALT5	1.5377	3.16E-22	4.23E-21	39.5753
ENSG00000198218	QRICH1	1.4552	3.19E-22	4.27E-21	39.5667
ENSG00000091879	ANGPT2	1.1302	3.25E-22	4.34E-21	39.5498
ENSG00000100784	RPS6KA5	1.4913	3.29E-22	4.39E-21	39.5376
ENSG00000170776	AKAP13	1.6993	3.30E-22	4.40E-21	39.5335
ENSG00000214046	SMIM7	0.6835	3.41E-22	4.55E-21	39.4996
ENSG00000198064	NPIPB13	1.7628	3.49E-22	4.65E-21	39.4775
ENSG00000168385	SEPTIN2	1.3793	3.52E-22	4.69E-21	39.4694
ENSG00000100226	GTPBP1	1.6470	3.58E-22	4.76E-21	39.4541
ENSG00000198960	ARMCX6	1.5087	3.58E-22	4.76E-21	39.4524
ENSG00000063660	GPC1	1.5231	3.62E-22	4.81E-21	39.4412
ENSG00000144369	FAM171B	1.9429	3.66E-22	4.86E-21	39.4300
ENSG00000203737	GPR52	1.1441	3.66E-22	4.86E-21	39.4306
ENSG00000148606	POLR3A	1.3853	3.68E-22	4.88E-21	39.4245
ENSG00000146221	TCTE1	1.3719	3.76E-22	4.98E-21	39.4053
ENSG00000159147	DONSON	1.4902	3.94E-22	5.22E-21	39.3580
ENSG00000105419	MEIS3	1.1995	3.95E-22	5.22E-21	39.3563
ENSG00000100764	PSMC1	1.3851	4.00E-22	5.29E-21	39.3424
ENSG00000140299	BNIP2	1.3863	4.21E-22	5.56E-21	39.2928
ENSG00000154945	ANKRD40	1.4280	4.30E-22	5.68E-21	39.2709
ENSG00000150477	KIAA1328	1.6682	4.33E-22	5.71E-21	39.2650
ENSG00000162783	IER5	2.0538	4.38E-22	5.77E-21	39.2539
ENSG00000077458	FAM76B	1.6144	4.40E-22	5.80E-21	39.2489
ENSG00000158195	WASF2	1.5175	4.42E-22	5.82E-21	39.2450
ENSG00000179604	CDC42EP4	1.5609	4.51E-22	5.94E-21	39.2239
ENSG00000153879	CEBPG	1.5028	4.53E-22	5.96E-21	39.2200
ENSG00000106819	ASPN	1.2880	4.54E-22	5.96E-21	39.2190
ENSG00000179119	SPTY2D1	1.5350	4.61E-22	6.05E-21	39.2036
ENSG00000161714	PLCD3	1.5830	4.73E-22	6.20E-21	39.1781
ENSG00000078369	GNB1	1.4285	4.74E-22	6.22E-21	39.1747
ENSG00000127946	HIP1	1.7131	4.75E-22	6.22E-21	39.1734
ENSG00000156273	BACH1	1.7712	4.90E-22	6.42E-21	39.1421
ENSG00000182197	EXT1	1.6552	5.00E-22	6.54E-21	39.1234
ENSG00000105640	RPL18A	0.6450	5.04E-22	6.59E-21	39.1140
ENSG00000114784	EIF1B	1.4311	5.15E-22	6.73E-21	39.0933
ENSG00000120948	TARDBP	1.3555	5.16E-22	6.74E-21	39.0908
ENSG00000196072	BLOC1S2	1.4098	5.18E-22	6.76E-21	39.0872
ENSG00000103544	VPS35L	1.4657	5.33E-22	6.95E-21	39.0585
ENSG00000165516	KLHDC2	1.4826	5.38E-22	7.01E-21	39.0502
ENSG00000134602	STK26	1.5491	5.38E-22	7.01E-21	39.0492
ENSG00000132376	INPP5K	1.4788	5.46E-22	7.10E-21	39.0359
ENSG00000175595	ERCC4	1.4813	5.56E-22	7.22E-21	39.0181
ENSG00000130255	RPL36	0.6180	5.63E-22	7.31E-21	39.0060
ENSG00000255112	CHMP1B	1.4639	5.73E-22	7.44E-21	38.9870
ENSG00000141627	DYM	1.3560	5.81E-22	7.53E-21	38.9746
ENSG00000095397	WHRN	2.7352	5.83E-22	7.56E-21	38.9704
ENSG00000104643	MTMR9	1.4419	5.83E-22	7.56E-21	38.9704

Gene ID	Gene Symbol	FC	P.Value	adj.P.Val	B
ENSG00000020633	RUNX3	1.8984	5.94E-22	7.69E-21	38.9528
ENSG00000141543	EIF4A3	1.6832	5.98E-22	7.74E-21	38.9455
ENSG00000115806	GORASP2	1.4329	6.08E-22	7.86E-21	38.9293
ENSG00000067365	METTL22	1.5146	6.31E-22	8.15E-21	38.8927
ENSG00000149187	CELF1	1.5773	6.46E-22	8.34E-21	38.8696
ENSG00000158769	F11R	1.6604	6.63E-22	8.55E-21	38.8442
ENSG00000163558	PRKCI	1.4582	6.66E-22	8.59E-21	38.8385
ENSG00000076604	TRAF4	2.4022	6.74E-22	8.68E-21	38.8270
ENSG00000145241	CENPC	1.5896	6.74E-22	8.68E-21	38.8269
ENSG00000146457	WTAP	1.4852	6.84E-22	8.80E-21	38.8125
ENSG00000161011	SQSTM1	1.7551	6.93E-22	8.91E-21	38.8004
ENSG00000173812	EIF1	1.4433	7.00E-22	9.00E-21	38.7895
ENSG00000170385	SLC30A1	1.7145	7.16E-22	9.19E-21	38.7680
ENSG00000144567	RETREG2	1.4253	7.31E-22	9.39E-21	38.7463
ENSG00000156970	BUB1B	2.1087	7.74E-22	9.92E-21	38.6906
ENSG00000171316	CHD7	1.7704	7.84E-22	1.00E-20	38.6783
ENSG00000088179	PTPN4	1.4317	7.88E-22	1.01E-20	38.6727
ENSG00000144655	CSRNP1	2.2912	8.07E-22	1.03E-20	38.6490
ENSG00000163719	MTMR14	1.5059	8.24E-22	1.05E-20	38.6284
ENSG00000100151	PICK1	1.5562	8.29E-22	1.06E-20	38.6221
ENSG00000133794	ARNTL	1.7576	8.35E-22	1.07E-20	38.6154
ENSG00000102226	USP11	1.6502	8.36E-22	1.07E-20	38.6141
ENSG00000029153	ARNTL2	1.9572	8.49E-22	1.08E-20	38.5984
ENSG00000084112	SSH1	1.4762	8.55E-22	1.09E-20	38.5919
ENSG00000178171	AMER3	1.0449	8.69E-22	1.11E-20	38.5763
ENSG00000113575	PPP2CA	1.4120	9.07E-22	1.15E-20	38.5334
ENSG00000122490	SLC66A2	1.5260	9.38E-22	1.19E-20	38.5009
ENSG00000124380	SNRNP27	1.3427	9.37E-22	1.19E-20	38.5013
ENSG00000143774	GUK1	1.4132	9.39E-22	1.19E-20	38.4992
ENSG00000158859	ADAMTS4	1.1589	9.52E-22	1.21E-20	38.4853
ENSG00000147119	CHST7	1.9299	9.93E-22	1.26E-20	38.4438
ENSG00000158773	USF1	1.5887	9.96E-22	1.26E-20	38.4407
ENSG00000049618	ARID1B	1.5334	1.01E-21	1.28E-20	38.4235
ENSG00000130338	TULP4	1.4376	1.02E-21	1.29E-20	38.4154
ENSG00000141030	COPS3	1.3393	1.02E-21	1.29E-20	38.4156
ENSG00000083642	PDS5B	1.4581	1.03E-21	1.31E-20	38.4054
ENSG00000067334	DNTTIP2	1.5161	1.06E-21	1.34E-20	38.3787
ENSG00000136527	TRA2B	1.5567	1.07E-21	1.35E-20	38.3743
ENSG00000121671	CRY2	1.5502	1.08E-21	1.36E-20	38.3649
ENSG00000170037	CNTROB	1.6181	1.09E-21	1.37E-20	38.3518
ENSG00000173889	PHC3	1.5034	1.11E-21	1.40E-20	38.3316
ENSG00000198089	SFI1	1.6249	1.13E-21	1.42E-20	38.3184
ENSG00000100425	BRD1	1.4256	1.17E-21	1.47E-20	38.2836
ENSG00000188501	LCTL	1.2303	1.19E-21	1.49E-20	38.2680
ENSG00000248771	SMIM31	1.2810	1.20E-21	1.51E-20	38.2536
ENSG00000215421	ZNF407	1.3344	1.22E-21	1.53E-20	38.2418
ENSG00000056277	ZNF280C	1.4377	1.25E-21	1.56E-20	38.2194
ENSG00000105193	RPS16	0.6665	1.26E-21	1.59E-20	38.2054
ENSG00000101343	CRNKL1	1.4453	1.27E-21	1.59E-20	38.2011
ENSG00000089902	RCOR1	1.5374	1.28E-21	1.60E-20	38.1946
ENSG00000047578	KATNIP	1.5002	1.28E-21	1.60E-20	38.1914
ENSG00000139977	NAA30	1.4732	1.28E-21	1.60E-20	38.1918
ENSG00000163162	RNF149	1.5678	1.32E-21	1.65E-20	38.1637
ENSG00000168564	CDKN2AIP	1.4784	1.37E-21	1.71E-20	38.1248

Gene ID	Gene Symbol	FC	P.Value	adj.P.Val	B
ENSG00000134056	MRPS36	0.6968	1.37E-21	1.72E-20	38.1228
ENSG00000107829	FBXW4	1.4472	1.41E-21	1.75E-20	38.1002
ENSG00000120685	PROSER1	1.6245	1.41E-21	1.75E-20	38.1004
ENSG00000168397	ATG4B	1.4370	1.47E-21	1.84E-20	38.0538
ENSG00000152061	RABGAP1L	1.5294	1.48E-21	1.84E-20	38.0523
ENSG00000162885	B3GALNT2	1.5549	1.49E-21	1.85E-20	38.0452
ENSG00000167632	TRAPPC9	1.4415	1.50E-21	1.86E-20	38.0375
ENSG00000104450	SPAG1	1.5397	1.52E-21	1.89E-20	38.0226
ENSG00000124788	ATXN1	1.8006	1.53E-21	1.90E-20	38.0149
ENSG00000237515	SHISA9	1.3695	1.53E-21	1.90E-20	38.0153
ENSG00000101400	SNTA1	1.7970	1.55E-21	1.92E-20	38.0070
ENSG00000130312	MRPL34	0.6232	1.57E-21	1.95E-20	37.9884
ENSG00000137720	C11orf1	0.6461	1.58E-21	1.95E-20	37.9868
ENSG00000162736	NCSTN	1.4352	1.59E-21	1.96E-20	37.9812
ENSG00000102962	CCL22	1.1169	1.63E-21	2.01E-20	37.9567
ENSG00000162482	AKR7A3	1.2423	1.70E-21	2.10E-20	37.9113
ENSG00000114744	COMMD2	1.4138	1.73E-21	2.14E-20	37.8944
ENSG00000176148	TCP11L1	1.5672	1.74E-21	2.15E-20	37.8906
ENSG00000169057	MECP2	1.5662	1.77E-21	2.18E-20	37.8743
ENSG00000137409	MTCH1	1.4650	1.78E-21	2.19E-20	37.8697
ENSG00000144644	GADL1	1.2158	1.78E-21	2.20E-20	37.8657
ENSG00000272514	CFAP206	1.1504	1.79E-21	2.20E-20	37.8611
ENSG00000148734	NPFFR1	1.0713	1.80E-21	2.22E-20	37.8546
ENSG00000205678	TECRL	1.1911	1.80E-21	2.22E-20	37.8535
ENSG00000124440	HIF3A	1.1729	1.81E-21	2.23E-20	37.8481
ENSG00000125652	ALKBH7	0.6925	1.86E-21	2.28E-20	37.8238
ENSG00000197046	SIGLEC15	1.2496	1.87E-21	2.29E-20	37.8205
ENSG00000166526	ZNF3	1.4332	1.90E-21	2.33E-20	37.8022
ENSG00000188687	SLC4A5	1.5870	1.94E-21	2.38E-20	37.7799
ENSG00000183977	PP2D1	1.4150	1.96E-21	2.41E-20	37.7694
ENSG00000175832	ETV4	1.7497	2.00E-21	2.45E-20	37.7523
ENSG00000144535	DIS3L2	1.3992	2.05E-21	2.51E-20	37.7262
ENSG00000213853	EMP2	1.4335	2.08E-21	2.54E-20	37.7152
ENSG00000074755	ZZEF1	1.5971	2.12E-21	2.59E-20	37.6941
ENSG00000100092	SH3BP1	1.8657	2.25E-21	2.74E-20	37.6371
ENSG00000166441	RPL27A	0.6565	2.26E-21	2.76E-20	37.6307
ENSG00000122477	LRRC39	1.5527	2.26E-21	2.76E-20	37.6290
ENSG00000142937	RPS8	0.6969	2.31E-21	2.82E-20	37.6079
ENSG00000181322	NME9	1.3027	2.46E-21	3.00E-20	37.5467
ENSG00000198836	OPA1	1.4892	2.48E-21	3.01E-20	37.5405
ENSG00000162601	MYSM1	1.5914	2.52E-21	3.07E-20	37.5224
ENSG00000198812	LRRC10	1.0704	2.55E-21	3.10E-20	37.5120
ENSG00000006453	BAIAP2L1	1.7033	2.56E-21	3.11E-20	37.5077
ENSG00000078177	N4BP2	1.6791	2.60E-21	3.15E-20	37.4936
ENSG00000151503	NCAPD3	1.6334	2.66E-21	3.23E-20	37.4697
ENSG00000148604	RGR	1.1423	2.67E-21	3.24E-20	37.4654
ENSG00000119314	PTBP3	1.4889	2.74E-21	3.32E-20	37.4414
ENSG00000026025	VIM	2.1510	2.78E-21	3.37E-20	37.4248
ENSG00000172954	LCLAT1	1.4178	2.79E-21	3.37E-20	37.4229
ENSG00000150656	CNDP1	1.6201	2.79E-21	3.38E-20	37.4213
ENSG00000197747	S100A10	2.8540	2.81E-21	3.39E-20	37.4167
ENSG00000157796	WDR19	1.8535	2.81E-21	3.40E-20	37.4150
ENSG00000170075	GPR37L1	1.0479	2.87E-21	3.46E-20	37.3945
ENSG00000141367	CLTC	1.4420	2.89E-21	3.49E-20	37.3877

Gene ID	Gene Symbol	FC	P.Value	adj.P.Val	B
ENSG00000214128	TMEM213	1.1758	2.95E-21	3.55E-20	37.3679
ENSG00000008513	ST3GAL1	2.5064	2.96E-21	3.57E-20	37.3627
ENSG00000223496	EXOSC6	1.4380	3.02E-21	3.64E-20	37.3439
ENSG00000132000	PODNL1	1.2902	3.08E-21	3.70E-20	37.3250
ENSG00000158669	GPAT4	1.4968	3.10E-21	3.73E-20	37.3172
ENSG00000182220	ATP6AP2	1.4377	3.17E-21	3.81E-20	37.2964
ENSG00000092978	GPATCH2	1.6477	3.21E-21	3.85E-20	37.2854
ENSG00000140854	KATNB1	1.5766	3.33E-21	3.99E-20	37.2485
ENSG00000102921	N4BP1	1.6332	3.40E-21	4.07E-20	37.2274
ENSG00000204524	ZNF805	1.4346	3.40E-21	4.07E-20	37.2280
ENSG00000047849	MAP4	1.6401	3.41E-21	4.09E-20	37.2234
ENSG00000092853	CLSPN	1.6524	3.47E-21	4.16E-20	37.2058
ENSG00000198369	SPRED2	2.1360	3.60E-21	4.31E-20	37.1701
ENSG00000100997	ABHD12	1.6639	3.74E-21	4.47E-20	37.1324
ENSG00000119396	RAB14	1.3587	3.74E-21	4.47E-20	37.1320
ENSG00000009950	MLXIPL	1.1150	3.76E-21	4.49E-20	37.1281
ENSG00000167962	ZNF598	1.5438	3.94E-21	4.70E-20	37.0807
ENSG00000162385	MAGOH	1.3677	3.98E-21	4.75E-20	37.0711
ENSG00000135605	TEC	2.0161	4.06E-21	4.84E-20	37.0507
ENSG00000101544	ADNP2	1.7557	4.08E-21	4.86E-20	37.0476
ENSG00000165458	INPPL1	1.5361	4.15E-21	4.94E-20	37.0291
ENSG00000135269	TES	1.6062	4.16E-21	4.95E-20	37.0268
ENSG00000138279	ANXA7	1.4155	4.16E-21	4.95E-20	37.0273
ENSG00000087087	SRRT	1.4120	4.27E-21	5.07E-20	37.0020
ENSG00000133392	MYH11	1.4571	4.27E-21	5.07E-20	37.0017
ENSG00000048707	VPS13D	1.4206	4.30E-21	5.10E-20	36.9959
ENSG00000134686	PHC2	1.5251	4.34E-21	5.14E-20	36.9871
ENSG00000123473	STIL	1.4803	4.41E-21	5.23E-20	36.9701
ENSG00000112964	GHR	1.3226	4.57E-21	5.41E-20	36.9349
ENSG00000053501	USE1	0.7010	4.73E-21	5.60E-20	36.9001
ENSG00000204961	PCDHA9	1.0491	4.98E-21	5.89E-20	36.8503
ENSG00000186684	CYP27C1	1.0798	5.08E-21	6.00E-20	36.8308
ENSG00000117868	ESYT2	1.4945	5.11E-21	6.03E-20	36.8254
ENSG00000133112	TPT1	0.6071	5.23E-21	6.18E-20	36.8011
ENSG00000091136	LAMB1	1.5655	5.40E-21	6.37E-20	36.7702
ENSG00000169515	CCDC8	1.1185	5.54E-21	6.52E-20	36.7455
ENSG00000156384	SFR1	1.6105	5.59E-21	6.58E-20	36.7367
ENSG00000111206	FOXM1	1.9190	5.67E-21	6.68E-20	36.7214
ENSG00000183891	TTC32	1.5368	5.69E-21	6.70E-20	36.7179
ENSG00000206199	ANKUB1	1.1536	5.84E-21	6.87E-20	36.6921
ENSG00000137860	SLC28A2	1.1658	5.87E-21	6.89E-20	36.6881
ENSG00000160299	PCNT	1.5085	6.02E-21	7.07E-20	36.6629
ENSG00000114423	CBLB	1.6257	6.18E-21	7.25E-20	36.6368
ENSG00000107643	MAPK8	1.5508	6.26E-21	7.35E-20	36.6233
ENSG00000133606	MKRN1	1.3942	6.32E-21	7.41E-20	36.6146
ENSG00000119596	YLPM1	1.5033	6.37E-21	7.46E-20	36.6072
ENSG00000102057	KCND1	1.4248	6.37E-21	7.46E-20	36.6065
ENSG00000117153	KLHL12	1.4698	6.38E-21	7.46E-20	36.6052
ENSG00000104897	SF3A2	1.4209	6.47E-21	7.56E-20	36.5916
ENSG00000149273	RPS3	0.6364	6.52E-21	7.62E-20	36.5838
ENSG00000131381	RBSN	1.4234	6.58E-21	7.68E-20	36.5748
ENSG00000138621	PPCDC	0.4953	6.73E-21	7.86E-20	36.5518
ENSG00000090238	YPEL3	1.7320	6.90E-21	8.04E-20	36.5283
ENSG00000138433	CIR1	1.4138	7.23E-21	8.42E-20	36.4821

Gene ID	Gene Symbol	FC	P.Value	adj.P.Val	B
ENSG00000151576	QTRT2	1.4843	7.26E-21	8.45E-20	36.4778
ENSG00000170759	KIF5B	1.3771	7.26E-21	8.45E-20	36.4773
ENSG00000172404	DNAJB7	1.1652	7.27E-21	8.45E-20	36.4766
ENSG00000141252	VPS53	1.4632	7.33E-21	8.52E-20	36.4685
ENSG00000206262	FOXL2NB	1.0951	7.34E-21	8.53E-20	36.4660
ENSG00000177291	GJD4	1.1151	7.48E-21	8.69E-20	36.4476
ENSG00000111786	SRSF9	1.2816	7.52E-21	8.72E-20	36.4431
ENSG00000198901	PRC1	1.7184	7.65E-21	8.88E-20	36.4254
ENSG00000068697	LAPTM4A	1.4590	7.80E-21	9.04E-20	36.4063
ENSG00000108262	GIT1	1.4383	7.90E-21	9.15E-20	36.3943
ENSG00000175311	ANKS4B	1.0776	8.00E-21	9.26E-20	36.3813
ENSG00000162825	NBPF20	2.0363	8.14E-21	9.42E-20	36.3644
ENSG00000172409	CLP1	1.5193	8.24E-21	9.52E-20	36.3529
ENSG00000150873	C2orf50	1.1357	8.32E-21	9.61E-20	36.3428
ENSG00000203666	EFCAB2	1.7273	8.47E-21	9.78E-20	36.3256
ENSG00000006634	DBF4	1.4419	8.76E-21	1.01E-19	36.2913
ENSG00000184900	SUMO3	1.4248	8.79E-21	1.01E-19	36.2881
ENSG00000172530	BANP	1.4370	8.82E-21	1.02E-19	36.2846
ENSG00000149798	CDC42EP2	1.2798	8.83E-21	1.02E-19	36.2836
ENSG00000172426	RSPH9	1.2293	8.99E-21	1.03E-19	36.2667
ENSG00000188610	FAM72B	1.6177	9.38E-21	1.08E-19	36.2238
ENSG00000106459	NRF1	1.3130	1.03E-20	1.19E-19	36.1293
ENSG00000104756	KCTD9	1.5298	1.04E-20	1.20E-19	36.1203
ENSG00000175782	SLC35E3	1.4272	1.05E-20	1.20E-19	36.1154
ENSG00000102302	FGD1	1.2275	1.12E-20	1.28E-19	36.0529
ENSG00000136881	BAAT	1.2628	1.13E-20	1.29E-19	36.0417
ENSG00000153721	CNKSR3	1.0745	1.14E-20	1.31E-19	36.0280
ENSG00000170049	KCNAB3	1.4664	1.18E-20	1.35E-19	36.0002
ENSG00000139182	CLSTN3	1.5970	1.19E-20	1.37E-19	35.9863
ENSG00000176842	IRX5	1.3488	1.20E-20	1.38E-19	35.9774
ENSG00000241852	C8orf58	1.5407	1.22E-20	1.40E-19	35.9605
ENSG00000259075	POC1B-GALNT4	1.5220	1.25E-20	1.43E-19	35.9407
ENSG00000198920	KIAA0753	1.4623	1.30E-20	1.48E-19	35.9041
ENSG00000112406	HECA	1.7778	1.31E-20	1.49E-19	35.8979
ENSG00000003756	RBM5	1.4265	1.31E-20	1.50E-19	35.8919
ENSG00000162664	ZNF326	1.4989	1.31E-20	1.50E-19	35.8916
ENSG00000115540	MOB4	1.3770	1.35E-20	1.54E-19	35.8646
ENSG00000152380	FAM151B	1.3648	1.36E-20	1.55E-19	35.8569
ENSG00000161405	IKZF3	1.8141	1.38E-20	1.57E-19	35.8460
ENSG00000178381	ZFAND2A	1.5352	1.41E-20	1.60E-19	35.8233
ENSG00000176105	YES1	1.6693	1.41E-20	1.60E-19	35.8221
ENSG00000163378	EOGT	1.5029	1.43E-20	1.62E-19	35.8100
ENSG00000137770	CTDSPL2	1.4929	1.44E-20	1.64E-19	35.7977
ENSG00000198242	RPL23A	0.6992	1.46E-20	1.65E-19	35.7885
ENSG00000026036	RTEL1-TNFRSF6B	1.5165	1.48E-20	1.68E-19	35.7725
ENSG00000105641	SLC5A5	1.0835	1.53E-20	1.74E-19	35.7391
ENSG00000123505	AMD1	1.4906	1.55E-20	1.76E-19	35.7279
ENSG00000166579	NDEL1	1.4530	1.55E-20	1.76E-19	35.7268
ENSG00000244462	RBM12	1.4165	1.57E-20	1.77E-19	35.7161
ENSG00000185950	IRS2	2.1399	1.58E-20	1.78E-19	35.7105
ENSG00000110324	IL10RA	2.8431	1.58E-20	1.79E-19	35.7061
ENSG00000119041	GTF3C3	1.4512	1.61E-20	1.82E-19	35.6894
ENSG00000163749	CCDC158	1.1512	1.64E-20	1.85E-19	35.6715
ENSG00000114942	EEF1B2	0.6470	1.70E-20	1.92E-19	35.6364

Gene ID	Gene Symbol	FC	P.Value	adj.P.Val	B
ENSG00000166908	PIP4K2C	1.4522	1.83E-20	2.06E-19	35.5644
ENSG00000007047	MARK4	1.3576	1.87E-20	2.10E-19	35.5440
ENSG00000189362	NEMP2	1.4800	1.89E-20	2.12E-19	35.5341
ENSG00000166822	TMEM170A	1.4651	1.92E-20	2.16E-19	35.5170
ENSG00000103319	EEF2K	1.5219	1.93E-20	2.17E-19	35.5130
ENSG00000198046	ZNF667	1.3637	1.97E-20	2.22E-19	35.4892
ENSG00000197385	ZNF860	0.4824	1.99E-20	2.23E-19	35.4815
ENSG00000135974	C2orf49	1.3333	2.00E-20	2.24E-19	35.4761
ENSG00000121879	PIK3CA	1.5974	2.01E-20	2.25E-19	35.4718
ENSG00000166068	SPRED1	2.2951	2.04E-20	2.29E-19	35.4563
ENSG00000136238	RAC1	1.3228	2.11E-20	2.36E-19	35.4247
ENSG00000101624	CEP76	1.4288	2.16E-20	2.42E-19	35.4005
ENSG00000103495	MAZ	1.4064	2.16E-20	2.42E-19	35.3978
ENSG00000203722	RAET1G	1.3143	2.27E-20	2.53E-19	35.3529
ENSG00000123146	ADGRE5	1.9615	2.27E-20	2.53E-19	35.3520
ENSG00000116191	RALGPS2	1.8329	2.27E-20	2.54E-19	35.3508
ENSG00000123124	WWP1	1.3866	2.28E-20	2.55E-19	35.3454
ENSG00000116194	ANGPTL1	1.4622	2.36E-20	2.63E-19	35.3144
ENSG00000075218	GTSE1	1.7556	2.38E-20	2.66E-19	35.3031
ENSG00000163050	COQ8A	1.5167	2.42E-20	2.70E-19	35.2860
ENSG00000104331	BPNT2	1.3937	2.45E-20	2.73E-19	35.2769
ENSG00000066279	ASPM	1.8611	2.48E-20	2.76E-19	35.2649
ENSG00000133872	SARAF	1.5870	2.58E-20	2.87E-19	35.2257
ENSG00000151338	MIPOL1	1.4405	2.58E-20	2.87E-19	35.2231
ENSG00000160752	FDPS	1.4200	2.62E-20	2.91E-19	35.2094
ENSG00000163866	SMIM12	1.3764	2.73E-20	3.03E-19	35.1686
ENSG00000149679	CABLES2	1.5280	2.75E-20	3.05E-19	35.1624
ENSG00000188167	TMPPE	1.2935	2.75E-20	3.05E-19	35.1614
ENSG00000004864	SLC25A13	1.4256	2.90E-20	3.21E-19	35.1101
ENSG00000007516	BAIAP3	2.0698	3.04E-20	3.36E-19	35.0636
ENSG00000142634	EFHD2	1.8628	3.04E-20	3.37E-19	35.0610
ENSG00000173692	PSMD1	1.3606	3.08E-20	3.41E-19	35.0482
ENSG00000243660	ZNF487	1.6577	3.16E-20	3.50E-19	35.0229
ENSG00000137818	RPLP1	0.6538	3.17E-20	3.50E-19	35.0212
ENSG00000177453	NIM1K	1.3382	3.24E-20	3.58E-19	34.9989
ENSG00000106546	AHR	2.5325	3.30E-20	3.65E-19	34.9805
ENSG00000102908	NFAT5	1.5676	3.34E-20	3.68E-19	34.9702
ENSG00000124214	STAU1	1.3387	3.36E-20	3.70E-19	34.9649
ENSG00000178741	COX5A	0.6697	3.38E-20	3.72E-19	34.9579
ENSG00000197603	CPLANE1	1.5532	3.42E-20	3.77E-19	34.9464
ENSG00000127081	ZNF484	1.3528	3.42E-20	3.77E-19	34.9447
ENSG00000058673	ZC3H11A	1.4246	3.44E-20	3.78E-19	34.9416
ENSG00000205922	ONECUT3	1.0692	3.54E-20	3.89E-19	34.9118
ENSG00000166869	CHP2	1.1728	3.56E-20	3.92E-19	34.9054
ENSG00000145675	PIK3R1	1.5354	3.58E-20	3.93E-19	34.9005
ENSG00000198373	WWP2	1.3982	3.59E-20	3.94E-19	34.8981
ENSG00000112305	SMAP1	1.3829	3.60E-20	3.95E-19	34.8960
ENSG00000163806	SPDYA	1.2828	3.64E-20	3.99E-19	34.8851
ENSG00000033030	ZCCHC8	1.4612	3.72E-20	4.08E-19	34.8622
ENSG00000170325	PRDM10	1.3437	3.75E-20	4.10E-19	34.8563
ENSG00000131013	PPIL4	1.5207	3.79E-20	4.15E-19	34.8436
ENSG00000196912	ANKRD36B	1.8023	3.82E-20	4.17E-19	34.8375
ENSG00000033627	ATP6V0A1	1.6107	3.87E-20	4.23E-19	34.8236
ENSG00000079482	OPHN1	1.4201	3.94E-20	4.31E-19	34.8054

Gene ID	Gene Symbol	FC	P.Value	adj.P.Val	B
ENSG00000006652	IFRD1	2.0354	3.97E-20	4.33E-19	34.7999
ENSG00000060237	WNK1	1.5815	4.15E-20	4.53E-19	34.7541
ENSG00000164287	CDC20B	1.2021	4.21E-20	4.59E-19	34.7409
ENSG00000183813	CCR4	1.1116	4.23E-20	4.61E-19	34.7351
ENSG00000092199	HNRNPC	1.3344	4.35E-20	4.74E-19	34.7076
ENSG00000097007	ABL1	1.3473	4.37E-20	4.76E-19	34.7036
ENSG00000107104	KANK1	0.4587	4.37E-20	4.76E-19	34.7035
ENSG00000101236	RNF24	1.4406	4.39E-20	4.78E-19	34.6991
ENSG00000112851	ERBIN	1.5207	4.40E-20	4.78E-19	34.6979
ENSG00000156232	WHAMM	1.5517	4.44E-20	4.82E-19	34.6883
ENSG00000088280	ASAP3	1.6197	4.50E-20	4.88E-19	34.6751
ENSG00000123411	IKZF4	1.3849	4.50E-20	4.88E-19	34.6747
ENSG00000118007	STAG1	1.3857	4.51E-20	4.89E-19	34.6719
ENSG00000094975	SUCO	1.5310	4.60E-20	4.98E-19	34.6538
ENSG00000147854	UHRF2	1.4432	4.60E-20	4.98E-19	34.6535
ENSG00000132017	DCAF15	1.4609	4.62E-20	5.00E-19	34.6484
ENSG00000153443	UBALD1	1.5533	4.64E-20	5.02E-19	34.6443
ENSG00000183763	TRAIP	1.6750	4.66E-20	5.04E-19	34.6406
ENSG00000139112	GABARAPL1	2.4570	4.72E-20	5.10E-19	34.6274
ENSG00000169045	HNRNPH1	1.5323	4.91E-20	5.31E-19	34.5878
ENSG00000106436	MYL10	1.1152	4.93E-20	5.32E-19	34.5856
ENSG00000035687	ADSS2	1.4410	4.97E-20	5.36E-19	34.5772
ENSG00000117614	SYF2	1.3858	4.98E-20	5.37E-19	34.5754
ENSG00000173875	ZNF791	1.5227	5.00E-20	5.39E-19	34.5701
ENSG00000178150	ZNF114	1.2865	5.01E-20	5.39E-19	34.5696
ENSG00000180447	GAS1	1.8791	5.15E-20	5.55E-19	34.5409
ENSG00000171453	POLR1C	1.4610	5.20E-20	5.59E-19	34.5326
ENSG00000146350	TBC1D32	1.4356	5.22E-20	5.61E-19	34.5287
ENSG00000158545	ZC3H18	1.4397	5.24E-20	5.63E-19	34.5248
ENSG00000065491	TBC1D22B	1.3510	5.36E-20	5.76E-19	34.5017
ENSG00000211455	STK38L	1.6493	5.54E-20	5.95E-19	34.4686
ENSG00000114023	FAM162A	0.6501	5.85E-20	6.28E-19	34.4151
ENSG00000078269	SYNJ2	1.7637	6.04E-20	6.47E-19	34.3845
ENSG00000130940	CASZ1	1.8437	6.08E-20	6.52E-19	34.3773
ENSG00000196600	SLC22A25	1.2141	6.18E-20	6.61E-19	34.3618
ENSG00000119397	CNTRL	1.5079	6.32E-20	6.76E-19	34.3393
ENSG00000150456	EEF1AKMT1	0.6142	6.41E-20	6.85E-19	34.3259
ENSG00000122966	CIT	1.7006	6.46E-20	6.90E-19	34.3181
ENSG00000179562	GCC1	1.3882	6.51E-20	6.96E-19	34.3100
ENSG00000156026	MCU	1.4668	6.52E-20	6.96E-19	34.3090
ENSG00000196313	POM121	1.4205	6.82E-20	7.28E-19	34.2640
ENSG00000197714	ZNF460	1.6383	7.06E-20	7.53E-19	34.2295
ENSG00000011304	PTBP1	1.3260	7.10E-20	7.57E-19	34.2242
ENSG00000121621	KIF18A	1.6627	7.16E-20	7.62E-19	34.2166
ENSG00000198276	UCKL1	1.4293	7.26E-20	7.73E-19	34.2024
ENSG00000078814	MYH7B	1.4247	7.41E-20	7.88E-19	34.1827
ENSG00000131153	GINS2	1.8388	7.53E-20	8.00E-19	34.1667
ENSG00000104856	RELB	2.1878	7.65E-20	8.13E-19	34.1506
ENSG00000187741	FANCA	1.8148	7.68E-20	8.16E-19	34.1468
ENSG00000141446	ESCO1	1.3302	7.94E-20	8.43E-19	34.1136
ENSG00000100426	ZBED4	1.3586	8.02E-20	8.51E-19	34.1044
ENSG00000151151	IPMK	1.3424	8.04E-20	8.52E-19	34.1020
ENSG00000159840	ZYX	2.7417	8.10E-20	8.59E-19	34.0936
ENSG00000175376	EIF1AD	1.4384	8.26E-20	8.75E-19	34.0754

Gene ID	Gene Symbol	FC	P.Value	adj.P.Val	B
ENSG00000135723	FHOD1	1.7452	8.29E-20	8.77E-19	34.0717
ENSG00000118058	KMT2A	1.7168	8.39E-20	8.88E-19	34.0591
ENSG00000186118	TEX38	1.1958	8.61E-20	9.11E-19	34.0334
ENSG00000105392	CRX	1.2265	8.62E-20	9.11E-19	34.0329
ENSG00000090263	MRPS33	0.6866	8.64E-20	9.13E-19	34.0301
ENSG00000204304	PBX2	1.3958	9.89E-20	1.04E-18	33.8973
ENSG00000112592	TBP	1.4116	1.02E-19	1.08E-18	33.8628
ENSG00000262152	GREP1	1.0883	1.03E-19	1.08E-18	33.8602
ENSG00000114030	KPNA1	1.4050	1.06E-19	1.11E-18	33.8326
ENSG00000100401	RANGAP1	1.4743	1.08E-19	1.14E-18	33.8070
ENSG00000204256	BRD2	1.4652	1.12E-19	1.18E-18	33.7755
ENSG00000115649	CNPPD1	1.5590	1.13E-19	1.18E-18	33.7690
ENSG00000144048	DUSP11	1.3504	1.13E-19	1.18E-18	33.7694
ENSG00000176393	RNPEP	1.4707	1.13E-19	1.18E-18	33.7692
ENSG00000134744	TUT4	1.5152	1.15E-19	1.21E-18	33.7488
ENSG00000164346	NSA2	0.6741	1.15E-19	1.21E-18	33.7451
ENSG00000146278	PNRC1	1.4592	1.17E-19	1.23E-18	33.7327
ENSG00000145391	SETD7	1.7121	1.18E-19	1.24E-18	33.7222
ENSG00000204420	MPIG6B	1.5936	1.18E-19	1.24E-18	33.7214
ENSG00000092148	HECTD1	1.4144	1.21E-19	1.27E-18	33.6991
ENSG00000013375	PGM3	1.6792	1.24E-19	1.29E-18	33.6774
ENSG00000164976	MYORG	1.2825	1.25E-19	1.31E-18	33.6639
ENSG00000158417	EIF5B	1.3887	1.28E-19	1.34E-18	33.6418
ENSG00000113732	ATP6V0E1	0.6579	1.30E-19	1.36E-18	33.6249
ENSG00000171443	ZNF524	0.7083	1.35E-19	1.41E-18	33.5869
ENSG00000166046	TCP11L2	1.8059	1.38E-19	1.45E-18	33.5647
ENSG00000100664	EIF5	1.6100	1.39E-19	1.45E-18	33.5582
ENSG00000116514	RNF19B	1.6524	1.40E-19	1.46E-18	33.5537
ENSG00000169180	XPO6	1.3924	1.41E-19	1.46E-18	33.5499
ENSG00000187189	TSPYL4	1.5531	1.41E-19	1.47E-18	33.5450
ENSG00000139370	SLC15A4	1.5079	1.42E-19	1.47E-18	33.5423
ENSG00000126249	PDCD2L	0.5459	1.42E-19	1.48E-18	33.5395
ENSG00000006607	FARP2	1.5844	1.42E-19	1.48E-18	33.5368
ENSG00000181523	SGSH	1.6597	1.48E-19	1.54E-18	33.4977
ENSG00000132522	GPS2	1.4016	1.50E-19	1.56E-18	33.4868
ENSG00000186166	CENATAC	1.4837	1.56E-19	1.62E-18	33.4494
ENSG00000179820	MYADM	4.4754	1.59E-19	1.65E-18	33.4291
ENSG00000118308	IRAG2	2.2844	1.61E-19	1.66E-18	33.4187
ENSG00000152332	UHMK1	1.6426	1.61E-19	1.66E-18	33.4184
ENSG00000168795	ZBTB5	1.5210	1.68E-19	1.74E-18	33.3711
ENSG00000164463	CREBRF	1.7450	1.69E-19	1.75E-18	33.3661
ENSG00000125691	RPL23	0.6740	1.70E-19	1.76E-18	33.3621
ENSG00000085719	CPNE3	1.5040	1.70E-19	1.76E-18	33.3615
ENSG00000090432	MUL1	1.4238	1.71E-19	1.77E-18	33.3558
ENSG00000153107	ANAPC1	1.3565	1.75E-19	1.81E-18	33.3343
ENSG00000186153	WWOX	1.4749	1.93E-19	2.00E-18	33.2349
ENSG00000164220	F2RL2	1.0992	1.96E-19	2.02E-18	33.2204
ENSG00000187905	LRRC74B	1.1184	1.99E-19	2.05E-18	33.2067
ENSG00000058600	POLR3E	1.3754	2.02E-19	2.08E-18	33.1940
ENSG00000154274	C4orf19	1.2514	2.03E-19	2.09E-18	33.1888
ENSG00000163481	RNF25	1.3990	2.06E-19	2.12E-18	33.1716
ENSG00000169756	LIMS1	1.6154	2.11E-19	2.18E-18	33.1468
ENSG00000122566	HNRNPA2B1	1.3431	2.16E-19	2.22E-18	33.1249
ENSG00000102316	MAGED2	1.6005	2.17E-19	2.23E-18	33.1197

Gene ID	Gene Symbol	FC	P.Value	adj.P.Val	B
ENSG00000136754	ABI1	1.3669	2.19E-19	2.25E-18	33.1109
ENSG00000198087	CD2AP	1.4576	2.23E-19	2.29E-18	33.0944
ENSG00000261873	SMIM36	1.2512	2.30E-19	2.36E-18	33.0631
ENSG00000165863	C10orf82	1.1224	2.32E-19	2.38E-18	33.0534
ENSG00000185013	NT5C1B	1.3262	2.37E-19	2.43E-18	33.0338
ENSG00000254772	EEF1G	0.6842	2.37E-19	2.43E-18	33.0324
ENSG00000169504	CLIC4	1.6335	2.41E-19	2.47E-18	33.0168
ENSG00000164610	RP9	1.3843	2.42E-19	2.48E-18	33.0117
ENSG00000154240	CEP112	1.4151	2.44E-19	2.49E-18	33.0058
ENSG00000147224	PRPS1	1.4780	2.45E-19	2.51E-18	32.9993
ENSG00000107796	ACTA2	1.4514	2.46E-19	2.51E-18	32.9969
ENSG00000175224	ATG13	1.3949	2.48E-19	2.53E-18	32.9884
ENSG00000166484	MAPK7	1.4254	2.49E-19	2.54E-18	32.9854
ENSG00000185158	LRRC37B	1.4343	2.49E-19	2.54E-18	32.9847
ENSG00000090989	EXOC1	1.4546	2.55E-19	2.60E-18	32.9605
ENSG00000161202	DVL3	1.3818	2.67E-19	2.72E-18	32.9169
ENSG00000027075	PRKCH	1.4408	2.68E-19	2.73E-18	32.9129
ENSG00000197256	KANK2	1.6957	2.69E-19	2.74E-18	32.9074
ENSG00000186660	ZFP91	1.3287	2.73E-19	2.77E-18	32.8956
ENSG00000182645	CCDC172	1.1010	2.74E-19	2.79E-18	32.8896
ENSG00000204628	RACK1	0.6568	2.88E-19	2.92E-18	32.8429
ENSG00000133030	MPRIP	1.4512	2.90E-19	2.95E-18	32.8344
ENSG00000132849	PATJ	1.7031	2.91E-19	2.95E-18	32.8326
ENSG00000141582	CBX4	1.6292	2.95E-19	3.00E-18	32.8163
ENSG00000116095	PLEKHA3	1.3796	3.08E-19	3.13E-18	32.7737
ENSG00000118197	DDX59	1.4959	3.13E-19	3.17E-18	32.7584
ENSG00000123595	RAB9A	1.4620	3.13E-19	3.17E-18	32.7584
ENSG00000081320	STK17B	2.0065	3.17E-19	3.21E-18	32.7458
ENSG00000158435	CNOT11	1.3235	3.22E-19	3.26E-18	32.7299
ENSG00000165660	ABRAXAS2	1.3439	3.28E-19	3.32E-18	32.7120
ENSG00000146904	EPHA1	1.4064	3.31E-19	3.34E-18	32.7054
ENSG00000164024	METAP1	1.3628	3.37E-19	3.41E-18	32.6860
ENSG00000137992	DBT	1.4038	3.44E-19	3.47E-18	32.6657
ENSG00000173852	DPY19L1	1.6557	3.46E-19	3.49E-18	32.6608
ENSG00000111405	ENDOU	1.8696	3.47E-19	3.50E-18	32.6567
ENSG00000082458	DLG3	1.6346	3.68E-19	3.71E-18	32.5993
ENSG00000231500	RPS18	0.6154	3.89E-19	3.92E-18	32.5456
ENSG00000087111	PIGS	1.4287	3.91E-19	3.94E-18	32.5397
ENSG00000160633	SAFB	1.3683	3.92E-19	3.95E-18	32.5369
ENSG00000109618	SEPSECS	1.4486	4.13E-19	4.15E-18	32.4854
ENSG00000166887	VPS39	1.4707	4.13E-19	4.15E-18	32.4850
ENSG00000088808	PPP1R13B	1.4751	4.15E-19	4.17E-18	32.4802
ENSG00000178690	DYNAP	1.1938	4.17E-19	4.19E-18	32.4765
ENSG00000177600	RPLP2	0.6746	4.21E-19	4.22E-18	32.4669
ENSG00000144218	AFF3	1.2564	4.24E-19	4.25E-18	32.4601
ENSG00000004897	CDC27	1.3235	4.26E-19	4.27E-18	32.4543
ENSG00000135334	AKIRIN2	1.6038	4.33E-19	4.34E-18	32.4399
ENSG00000204104	TRAF3IP1	1.3828	4.40E-19	4.41E-18	32.4228
ENSG00000225830	ERCC6	1.3928	4.41E-19	4.42E-18	32.4197
ENSG00000108424	KPNB1	1.4042	4.47E-19	4.47E-18	32.4079
ENSG00000151247	EIF4E	1.3675	4.52E-19	4.52E-18	32.3968
ENSG00000101452	DHX35	1.3646	4.56E-19	4.56E-18	32.3872
ENSG00000169902	TPST1	2.0605	4.69E-19	4.68E-18	32.3602
ENSG00000146410	MTFR2	1.5784	4.75E-19	4.74E-18	32.3472

Gene ID	Gene Symbol	FC	P.Value	adj.P.Val	B
ENSG00000138095	LRPPRC	1.3123	4.76E-19	4.75E-18	32.3461
ENSG00000116983	HPCAL4	1.3247	4.85E-19	4.84E-18	32.3263
ENSG00000147548	NSD3	1.5079	4.91E-19	4.89E-18	32.3151
ENSG00000039523	RIPOR1	2.1055	4.91E-19	4.89E-18	32.3144
ENSG00000141959	PFKL	1.4610	4.99E-19	4.97E-18	32.2991
ENSG00000124795	DEK	2.2046	5.09E-19	5.07E-18	32.2784
ENSG00000104825	NFKBIB	1.4518	5.15E-19	5.13E-18	32.2667
ENSG00000117505	DR1	1.4845	5.15E-19	5.13E-18	32.2669
ENSG00000167881	SRP68	1.3416	5.22E-19	5.19E-18	32.2539
ENSG00000092871	RFFL	1.4788	5.25E-19	5.22E-18	32.2478
ENSG00000161970	RPL26	0.6495	5.32E-19	5.28E-18	32.2349
ENSG00000129518	EAPP	1.3633	5.40E-19	5.36E-18	32.2202
ENSG00000100083	GGA1	1.4501	5.48E-19	5.43E-18	32.2064
ENSG00000197860	SGTB	1.5105	5.49E-19	5.44E-18	32.2051
ENSG00000077150	NFKB2	2.1291	5.49E-19	5.44E-18	32.2037
ENSG00000112306	RPS12	0.6768	5.50E-19	5.44E-18	32.2034
ENSG00000172766	NAA16	1.6231	5.59E-19	5.53E-18	32.1862
ENSG00000157500	APPL1	1.4218	5.66E-19	5.60E-18	32.1743
ENSG00000170260	ZNF212	1.4369	5.84E-19	5.77E-18	32.1432
ENSG00000099290	WASHC2A	1.5189	5.87E-19	5.80E-18	32.1378
ENSG00000182310	SPACA6	1.6065	6.14E-19	6.07E-18	32.0937
ENSG00000113272	THG1L	0.6132	6.25E-19	6.17E-18	32.0761
ENSG00000108515	ENO3	1.6892	6.26E-19	6.17E-18	32.0751
ENSG00000145016	RUBCN	1.5669	6.39E-19	6.30E-18	32.0544
ENSG00000052795	FNIP2	1.6289	6.48E-19	6.39E-18	32.0408
ENSG00000115758	ODC1	1.5225	6.61E-19	6.51E-18	32.0206
ENSG00000110697	PITPNM1	1.7056	6.70E-19	6.59E-18	32.0081
ENSG00000160908	ZNF394	1.3702	6.70E-19	6.60E-18	32.0072
ENSG00000101096	NFATC2	1.6535	6.71E-19	6.60E-18	32.0066
ENSG00000119599	DCAF4	1.5460	6.77E-19	6.66E-18	31.9971
ENSG00000158427	TMSB15B	1.8114	6.97E-19	6.85E-18	31.9686
ENSG00000140153	WDR20	1.3403	7.10E-19	6.97E-18	31.9504
ENSG00000127540	UQCR11	0.7152	7.37E-19	7.23E-18	31.9136
ENSG00000152076	CCDC74B	1.3174	7.44E-19	7.30E-18	31.9046
ENSG00000086061	DNAJA1	1.4257	7.48E-19	7.34E-18	31.8987
ENSG00000122406	RPL5	0.6595	7.55E-19	7.40E-18	31.8896
ENSG00000115207	GTF3C2	1.3576	7.56E-19	7.40E-18	31.8889
ENSG00000106714	CNTNAP3	1.4019	7.71E-19	7.55E-18	31.8692
ENSG00000176903	PNMA1	1.9897	7.82E-19	7.65E-18	31.8556
ENSG00000105127	AKAP8	1.3522	7.83E-19	7.66E-18	31.8536
ENSG00000180432	CYP8B1	1.0606	7.84E-19	7.66E-18	31.8531
ENSG00000145990	GFOD1	1.7098	7.88E-19	7.70E-18	31.8472
ENSG00000167281	RBFOX3	1.1977	8.19E-19	7.99E-18	31.8098
ENSG00000079432	CIC	1.4779	8.28E-19	8.08E-18	31.7992
ENSG00000167604	NFKBID	1.9808	8.35E-19	8.15E-18	31.7901
ENSG00000203668	CHML	1.7934	8.40E-19	8.19E-18	31.7849
ENSG00000138670	RASGEF1B	2.1601	8.44E-19	8.22E-18	31.7799
ENSG00000034677	RNF19A	1.5801	8.49E-19	8.26E-18	31.7744
ENSG00000134531	EMP1	2.3293	8.59E-19	8.36E-18	31.7621
ENSG00000146676	PURB	1.4004	8.64E-19	8.40E-18	31.7573
ENSG00000014164	ZC3H3	1.4150	8.66E-19	8.42E-18	31.7540
ENSG00000076984	MAP2K7	1.4203	8.71E-19	8.47E-18	31.7483
ENSG00000116584	ARHGEF2	1.7032	8.80E-19	8.54E-18	31.7391
ENSG00000182795	C1orf116	1.3750	8.84E-19	8.58E-18	31.7345

Gene ID	Gene Symbol	FC	P.Value	adj.P.Val	B
ENSG00000075292	ZNF638	1.3550	9.10E-19	8.83E-18	31.7054
ENSG00000124831	LRRFIP1	1.5951	9.24E-19	8.96E-18	31.6906
ENSG00000104728	ARHGEF10	1.3408	9.31E-19	9.02E-18	31.6830
ENSG00000198108	CHSY3	0.4057	9.58E-19	9.28E-18	31.6553
ENSG00000154305	MIA3	1.4641	9.72E-19	9.42E-18	31.6400
ENSG00000176986	SEC24C	1.3819	9.79E-19	9.47E-18	31.6337
ENSG00000124145	SDC4	1.4061	9.80E-19	9.48E-18	31.6323
ENSG00000150403	TMCO3	1.6581	9.94E-19	9.61E-18	31.6185
ENSG00000056972	TRAF3IP2	1.4871	1.02E-18	9.84E-18	31.5948
ENSG00000204531	POU5F1	1.3879	1.03E-18	9.92E-18	31.5859
ENSG00000090273	NUDC	1.3527	1.03E-18	9.96E-18	31.5811
ENSG00000143768	LEFTY2	2.0765	1.04E-18	1.01E-17	31.5718
ENSG00000135094	SDS	1.5903	1.05E-18	1.02E-17	31.5611
ENSG00000164603	BMT2	1.3297	1.05E-18	1.02E-17	31.5616
ENSG00000134864	GGACT	0.6051	1.06E-18	1.02E-17	31.5525
ENSG00000143476	DTL	1.9131	1.06E-18	1.02E-17	31.5522
ENSG00000137337	MDC1	1.5093	1.08E-18	1.04E-17	31.5376
ENSG00000166313	APBB1	1.9713	1.08E-18	1.04E-17	31.5367
ENSG00000100697	DICER1	1.4913	1.09E-18	1.04E-17	31.5316
ENSG00000140505	CYP1A2	1.1826	1.10E-18	1.06E-17	31.5190
ENSG00000185591	SP1	1.4875	1.12E-18	1.07E-17	31.5036
ENSG00000183747	ACSM2A	1.4002	1.12E-18	1.08E-17	31.4981
ENSG00000162852	CNST	1.4866	1.13E-18	1.08E-17	31.4925
ENSG00000167491	GATAD2A	1.3935	1.13E-18	1.08E-17	31.4910
ENSG00000256683	ZNF350	1.9948	1.13E-18	1.08E-17	31.4914
ENSG00000198198	SZT2	1.4531	1.14E-18	1.09E-17	31.4842
ENSG00000164258	NDUFS4	0.7023	1.19E-18	1.14E-17	31.4367
ENSG00000147872	PLIN2	1.9787	1.22E-18	1.17E-17	31.4158
ENSG00000138767	CNOT6L	1.5574	1.25E-18	1.20E-17	31.3888
ENSG00000183569	SERHL2	1.3068	1.26E-18	1.20E-17	31.3842
ENSG00000132326	PER2	1.5249	1.33E-18	1.28E-17	31.3274
ENSG00000168807	SNTB2	1.4070	1.36E-18	1.30E-17	31.3113
ENSG00000095015	MAP3K1	1.5517	1.38E-18	1.32E-17	31.2910
ENSG00000160917	CPSF4	1.3932	1.40E-18	1.34E-17	31.2800
ENSG00000136944	LMX1B	1.0531	1.45E-18	1.38E-17	31.2475
ENSG00000129055	ANAPC13	0.6972	1.45E-18	1.39E-17	31.2433
ENSG00000163795	ZNF513	1.4153	1.49E-18	1.42E-17	31.2210
ENSG00000162378	ZYG11B	1.3283	1.50E-18	1.42E-17	31.2149
ENSG00000051341	POLQ	1.5599	1.53E-18	1.45E-17	31.1944
ENSG00000167323	STIM1	1.5064	1.54E-18	1.46E-17	31.1869
ENSG00000100918	REC8	1.8457	1.56E-18	1.48E-17	31.1743
ENSG00000196378	ZNF34	1.4095	1.57E-18	1.49E-17	31.1684
ENSG00000143858	SYT2	1.0921	1.58E-18	1.50E-17	31.1618
ENSG00000154839	SKA1	1.7296	1.59E-18	1.51E-17	31.1573
ENSG00000219626	FAM228B	1.4624	1.63E-18	1.55E-17	31.1273
ENSG00000133302	SLF1	1.3964	1.68E-18	1.59E-17	31.1032
ENSG00000169446	MMGT1	1.4313	1.68E-18	1.59E-17	31.0996
ENSG00000119537	KDSR	1.5948	1.74E-18	1.65E-17	31.0664
ENSG00000111602	TIMELESS	1.7907	1.75E-18	1.66E-17	31.0605
ENSG00000125520	SLC2A4RG	1.6393	1.78E-18	1.69E-17	31.0413
ENSG00000133874	RNF122	1.7370	1.80E-18	1.70E-17	31.0347
ENSG00000198755	RPL10A	0.7161	1.80E-18	1.70E-17	31.0350
ENSG00000204519	ZNF551	1.4264	1.79E-18	1.70E-17	31.0350
ENSG00000106636	YKT6	1.3897	1.87E-18	1.77E-17	30.9952

Gene ID	Gene Symbol	FC	P.Value	adj.P.Val	B
ENSG00000182687	GALR2	1.2200	1.90E-18	1.79E-17	30.9799
ENSG00000145623	OSMR	1.8428	2.00E-18	1.89E-17	30.9268
ENSG00000123064	DDX54	1.3406	2.00E-18	1.89E-17	30.9261
ENSG00000080823	MOK	1.5261	2.03E-18	1.92E-17	30.9113
ENSG00000141380	SS18	1.4675	2.04E-18	1.93E-17	30.9071
ENSG00000188315	C3orf62	1.4994	2.07E-18	1.95E-17	30.8946
ENSG00000171862	PTEN	1.3757	2.10E-18	1.98E-17	30.8791
ENSG00000176563	CNTD1	1.3400	2.13E-18	2.00E-17	30.8682
ENSG00000173281	PPP1R3B	1.4420	2.15E-18	2.02E-17	30.8570
ENSG00000115568	ZNF142	1.5065	2.16E-18	2.03E-17	30.8544
ENSG00000088726	TMEM40	1.2841	2.28E-18	2.14E-17	30.7984
ENSG00000221821	C6orf226	1.5305	2.29E-18	2.15E-17	30.7956
ENSG00000120910	PPP3CC	1.5246	2.29E-18	2.15E-17	30.7941
ENSG00000085415	SEH1L	1.3958	2.32E-18	2.18E-17	30.7802
ENSG00000081181	ARG2	1.5529	2.38E-18	2.23E-17	30.7576
ENSG00000198231	DDX42	1.4252	2.38E-18	2.23E-17	30.7567
ENSG00000099246	RAB18	1.3477	2.42E-18	2.27E-17	30.7404
ENSG00000135655	USP15	1.4430	2.48E-18	2.32E-17	30.7147
ENSG00000185515	BRCC3	1.4627	2.51E-18	2.35E-17	30.7026
ENSG00000114861	FOXP1	2.0512	2.64E-18	2.47E-17	30.6538
ENSG00000067992	PDK3	1.3320	2.68E-18	2.50E-17	30.6394
ENSG00000172007	RAB33B	1.4246	2.73E-18	2.55E-17	30.6224
ENSG00000137038	DMAC1	0.6457	2.74E-18	2.55E-17	30.6189
ENSG00000143951	WDPCP	1.3373	2.75E-18	2.56E-17	30.6148
ENSG00000147421	HMBOX1	1.5014	2.87E-18	2.68E-17	30.5717
ENSG00000159788	RGS12	1.6841	2.88E-18	2.69E-17	30.5683
ENSG00000177189	RPS6KA3	1.4323	2.90E-18	2.70E-17	30.5615
ENSG00000128989	ARPP19	1.4948	2.91E-18	2.71E-17	30.5583
ENSG00000198523	PLN	1.1813	3.05E-18	2.84E-17	30.5131
ENSG00000112033	PPARD	1.3668	3.06E-18	2.84E-17	30.5097
ENSG00000135686	KLHL36	1.5256	3.08E-18	2.87E-17	30.5017
ENSG00000176399	DMRTA1	1.4392	3.09E-18	2.87E-17	30.4988
ENSG00000198561	CTNND1	1.4833	3.10E-18	2.88E-17	30.4970
ENSG00000184163	C1QTNF12	1.6376	3.12E-18	2.89E-17	30.4903
ENSG00000102900	NUP93	1.4727	3.18E-18	2.95E-17	30.4695
ENSG00000074356	NCBP3	1.4317	3.19E-18	2.96E-17	30.4676
ENSG00000234127	TRIM26	1.3290	3.25E-18	3.02E-17	30.4477
ENSG00000158014	SLC30A2	1.1564	3.31E-18	3.07E-17	30.4319
ENSG00000126368	NR1D1	2.0663	3.41E-18	3.16E-17	30.4009
ENSG00000118454	ANKRD13C	1.3463	3.43E-18	3.18E-17	30.3957
ENSG00000196867	ZFP28	1.5316	3.71E-18	3.43E-17	30.3197
ENSG00000153179	RASSF3	1.7733	3.74E-18	3.46E-17	30.3115
ENSG00000123144	TRIR	0.7564	3.75E-18	3.47E-17	30.3076
ENSG00000116688	MFN2	1.3243	3.76E-18	3.48E-17	30.3049
ENSG00000013588	GPRC5A	1.2186	3.98E-18	3.68E-17	30.2492
ENSG00000055732	MCOLN3	1.2589	4.03E-18	3.72E-17	30.2368
ENSG00000085978	ATG16L1	1.3494	4.03E-18	3.72E-17	30.2365
ENSG00000125459	MSTO1	1.6847	4.10E-18	3.78E-17	30.2206
ENSG00000108010	GLRX3	1.3007	4.18E-18	3.85E-17	30.2004
ENSG00000009724	MASP2	1.4405	4.28E-18	3.94E-17	30.1779
ENSG00000171634	BPTF	1.4377	4.29E-18	3.95E-17	30.1752
ENSG00000130684	ZNF337	1.4760	4.39E-18	4.04E-17	30.1527
ENSG00000138107	ACTR1A	1.3864	4.39E-18	4.04E-17	30.1528
ENSG00000059758	CDK17	1.4612	4.40E-18	4.05E-17	30.1494

Gene ID	Gene Symbol	FC	P.Value	adj.P.Val	B
ENSG00000135447	PPP1R1A	1.2226	4.41E-18	4.05E-17	30.1478
ENSG00000110075	PPP6R3	1.3539	4.46E-18	4.10E-17	30.1360
ENSG00000143158	MPC2	1.3911	4.57E-18	4.20E-17	30.1130
ENSG00000182718	ANXA2	1.7176	4.59E-18	4.22E-17	30.1077
ENSG00000110693	SOX6	1.3873	4.60E-18	4.22E-17	30.1056
ENSG00000083799	CYLD	1.6996	4.74E-18	4.35E-17	30.0762
ENSG00000135914	HTR2B	1.1271	4.75E-18	4.35E-17	30.0742
ENSG00000130159	ECSIT	0.7004	4.77E-18	4.37E-17	30.0699
ENSG00000167005	NUDT21	1.3302	4.88E-18	4.47E-17	30.0472
ENSG00000166257	SCN3B	1.1389	4.99E-18	4.57E-17	30.0253
ENSG00000138658	ZGRF1	1.5342	5.07E-18	4.64E-17	30.0101
ENSG00000127337	YEATS4	1.3474	5.17E-18	4.72E-17	29.9919
ENSG00000129219	PLD2	1.7702	5.22E-18	4.77E-17	29.9822
ENSG00000257594	GALNT4	1.5144	5.27E-18	4.81E-17	29.9728
ENSG00000196235	SUPT5H	1.4034	5.32E-18	4.85E-17	29.9633
ENSG00000185163	DDX51	1.4684	5.32E-18	4.86E-17	29.9620
ENSG00000155729	KCTD18	1.3018	5.39E-18	4.91E-17	29.9501
ENSG00000146112	PPP1R18	1.6321	5.43E-18	4.95E-17	29.9431
ENSG00000189129	PLAC9	1.7607	5.54E-18	5.05E-17	29.9224
ENSG00000114857	NKTR	1.6287	5.56E-18	5.06E-17	29.9203
ENSG00000100883	SRP54	1.4077	5.58E-18	5.08E-17	29.9163
ENSG00000175984	DENND2C	1.6938	5.58E-18	5.08E-17	29.9155
ENSG00000158062	UBXN11	1.5997	5.65E-18	5.14E-17	29.9027
ENSG00000153944	MSI2	1.4880	5.72E-18	5.20E-17	29.8907
ENSG00000102001	CACNA1F	1.3228	5.75E-18	5.22E-17	29.8870
ENSG00000046651	OFD1	1.4868	5.78E-18	5.24E-17	29.8819
ENSG00000234828	IQCM	1.3383	5.84E-18	5.30E-17	29.8701
ENSG00000160072	ATAD3B	1.4548	6.12E-18	5.55E-17	29.8250
ENSG00000065243	PKN2	1.5186	6.16E-18	5.59E-17	29.8176
ENSG00000173473	SMARCC1	1.3717	6.20E-18	5.62E-17	29.8120
ENSG00000171861	MRM3	1.3185	6.20E-18	5.62E-17	29.8111
ENSG00000163510	CWC22	1.3152	6.23E-18	5.64E-17	29.8079
ENSG00000129355	CDKN2D	1.9816	6.28E-18	5.69E-17	29.7988
ENSG00000184508	HDDC3	0.6537	6.36E-18	5.76E-17	29.7863
ENSG00000165714	BORCS5	1.3578	6.42E-18	5.80E-17	29.7782
ENSG00000086619	ERO1B	1.5553	6.48E-18	5.85E-17	29.7687
ENSG00000167182	SP2	1.3400	6.52E-18	5.89E-17	29.7623
ENSG00000130803	ZNF317	1.3413	6.56E-18	5.92E-17	29.7567
ENSG00000111266	DUSP16	1.5343	6.63E-18	5.99E-17	29.7453
ENSG00000254440	PBOV1	1.0519	6.73E-18	6.07E-17	29.7305
ENSG00000138468	SENP7	1.4111	6.78E-18	6.11E-17	29.7240
ENSG00000196998	WDR45	1.3739	6.82E-18	6.14E-17	29.7179
ENSG00000164953	TMEM67	1.5318	6.87E-18	6.19E-17	29.7106
ENSG00000068724	TTC7A	1.4977	6.88E-18	6.19E-17	29.7097
ENSG00000102034	ELF4	1.8749	6.93E-18	6.24E-17	29.7018
ENSG00000124782	RREB1	1.4045	6.96E-18	6.26E-17	29.6976
ENSG00000124212	PTGIS	1.0472	7.02E-18	6.31E-17	29.6893
ENSG00000144481	TRPM8	1.3155	7.32E-18	6.58E-17	29.6475
ENSG00000177732	SOX12	1.6032	7.55E-18	6.78E-17	29.6178
ENSG00000131351	HAUS8	1.5026	7.57E-18	6.80E-17	29.6148
ENSG00000177663	IL17RA	1.4191	7.68E-18	6.89E-17	29.6006
ENSG00000116128	BCL9	1.6743	7.69E-18	6.89E-17	29.5999
ENSG00000168818	STX18	1.3230	7.72E-18	6.92E-17	29.5955
ENSG00000141580	WDR45B	1.3536	7.80E-18	6.98E-17	29.5858

Gene ID	Gene Symbol	FC	P.Value	adj.P.Val	B
ENSG00000141564	RPTOR	1.3718	8.04E-18	7.20E-17	29.5553
ENSG00000145868	FBXO38	1.3896	8.19E-18	7.33E-17	29.5368
ENSG00000036054	TBC1D23	1.3812	8.22E-18	7.36E-17	29.5333
ENSG00000101849	TBL1X	1.5111	8.52E-18	7.62E-17	29.4980
ENSG00000168028	RPSA	0.6860	8.73E-18	7.80E-17	29.4748
ENSG00000138385	SSB	1.3562	8.74E-18	7.81E-17	29.4731
ENSG00000173124	ACSM6	1.1292	8.74E-18	7.81E-17	29.4728
ENSG00000149380	P4HA3	1.2696	8.78E-18	7.83E-17	29.4688
ENSG00000070808	CAMK2A	1.1301	8.80E-18	7.85E-17	29.4668
ENSG00000068024	HDAC4	1.4607	9.27E-18	8.26E-17	29.4152
ENSG00000186834	HEXIM1	1.4650	9.27E-18	8.27E-17	29.4146
ENSG00000137166	FOXP4	1.4361	9.30E-18	8.28E-17	29.4123
ENSG00000106384	MOGAT3	1.1125	9.33E-18	8.31E-17	29.4083
ENSG00000140545	MFGE8	2.9466	9.35E-18	8.32E-17	29.4069
ENSG00000204564	C6orf136	1.4355	9.41E-18	8.37E-17	29.4002
ENSG00000130052	STARD8	1.5430	9.49E-18	8.44E-17	29.3917
ENSG00000188199	NUTM2B	1.2699	9.51E-18	8.45E-17	29.3902
ENSG00000134324	LPIN1	1.8736	9.53E-18	8.46E-17	29.3878
ENSG00000065802	ASB1	1.3322	9.76E-18	8.67E-17	29.3638
ENSG00000249961	TERB1	1.1678	9.79E-18	8.69E-17	29.3612
ENSG00000178074	C2orf69	1.3590	9.95E-18	8.82E-17	29.3454
ENSG00000257103	LSM14A	1.3304	9.95E-18	8.82E-17	29.3456
ENSG00000167117	ANKRD40CL	1.2231	1.02E-17	9.04E-17	29.3207
ENSG00000171962	DRC3	1.5196	1.03E-17	9.09E-17	29.3150
ENSG00000131165	CHMP1A	1.4424	1.03E-17	9.10E-17	29.3133
ENSG00000165801	ARHGEF40	2.2735	1.03E-17	9.14E-17	29.3086
ENSG00000113300	CNOT6	1.3609	1.04E-17	9.22E-17	29.2991
ENSG00000138778	CENPE	1.5036	1.05E-17	9.30E-17	29.2904
ENSG00000221914	PPP2R2A	1.4517	1.06E-17	9.37E-17	29.2825
ENSG00000166188	ZNF319	1.4177	1.07E-17	9.42E-17	29.2768
ENSG00000188243	COMMD6	0.6929	1.11E-17	9.82E-17	29.2349
ENSG00000162607	USP1	1.5185	1.12E-17	9.89E-17	29.2282
ENSG00000198205	ZXDA	1.4138	1.15E-17	1.01E-16	29.2032
ENSG00000088247	KHSRP	1.3564	1.18E-17	1.04E-16	29.1807
ENSG00000182372	CLN8	1.5139	1.18E-17	1.04E-16	29.1789
ENSG00000009830	POMT2	1.5487	1.18E-17	1.04E-16	29.1776
ENSG00000131469	RPL27	0.7388	1.19E-17	1.04E-16	29.1717
ENSG00000188827	SLX4	1.4040	1.19E-17	1.05E-16	29.1701
ENSG00000164978	NUDT2	0.6928	1.19E-17	1.05E-16	29.1667
ENSG00000087301	TXNDC16	1.4168	1.24E-17	1.09E-16	29.1266
ENSG00000106268	NUDT1	1.5155	1.25E-17	1.10E-16	29.1171
ENSG00000248333	CDK11B	1.3505	1.28E-17	1.12E-16	29.0999
ENSG00000137449	CPEB2	1.7510	1.29E-17	1.13E-16	29.0919
ENSG00000068383	INPP5A	1.4733	1.30E-17	1.14E-16	29.0785
ENSG00000178665	ZNF713	1.2365	1.30E-17	1.14E-16	29.0782
ENSG00000205659	LIN52	1.4618	1.31E-17	1.15E-16	29.0722
ENSG00000105697	HAMP	1.3260	1.33E-17	1.16E-16	29.0621
ENSG00000136813	ECPAS	1.3458	1.34E-17	1.17E-16	29.0526
ENSG00000205581	HMGN1	1.3863	1.35E-17	1.18E-16	29.0450
ENSG00000083750	RRAGB	1.3839	1.35E-17	1.19E-16	29.0409
ENSG00000174792	ODAPH	1.4067	1.38E-17	1.20E-16	29.0246
ENSG00000143107	FNDC7	1.2511	1.41E-17	1.23E-16	28.9996
ENSG00000138092	CENPO	1.5167	1.42E-17	1.24E-16	28.9956
ENSG00000143641	GALNT2	1.5068	1.43E-17	1.25E-16	28.9893

Gene ID	Gene Symbol	FC	P.Value	adj.P.Val	B
ENSG00000067560	RHOA	1.3036	1.44E-17	1.26E-16	28.9786
ENSG00000153250	RBMS1	1.7646	1.45E-17	1.27E-16	28.9714
ENSG00000151651	ADAM8	2.3653	1.48E-17	1.29E-16	28.9560
ENSG00000153165	RGPD3	1.1632	1.48E-17	1.29E-16	28.9514
ENSG00000100316	RPL3	0.7024	1.49E-17	1.29E-16	28.9500
ENSG00000144589	STK11IP	1.5493	1.49E-17	1.30E-16	28.9477
ENSG00000119862	LGALSL	1.9725	1.49E-17	1.30E-16	28.9468
ENSG00000076662	ICAM3	0.6318	1.49E-17	1.30E-16	28.9451
ENSG00000102103	PQBP1	1.2723	1.51E-17	1.31E-16	28.9332
ENSG00000137947	GTF2B	1.4443	1.51E-17	1.32E-16	28.9308
ENSG00000137193	PIM1	2.3031	1.53E-17	1.33E-16	28.9187
ENSG00000005156	LIG3	1.4758	1.54E-17	1.34E-16	28.9134
ENSG00000154978	VOPP1	1.5984	1.54E-17	1.34E-16	28.9116
ENSG00000070950	RAD18	1.4466	1.56E-17	1.35E-16	28.9014
ENSG00000136560	TANK	1.4052	1.57E-17	1.36E-16	28.8975
ENSG00000204305	AGER	1.5551	1.57E-17	1.36E-16	28.8926
ENSG00000100266	PACSIN2	1.4273	1.61E-17	1.40E-16	28.8700
ENSG00000176681	LRRC37A	1.4903	1.67E-17	1.45E-16	28.8325
ENSG00000171222	SCAND1	1.3662	1.76E-17	1.52E-16	28.7823
ENSG00000213190	MLLT11	1.5949	1.77E-17	1.53E-16	28.7773
ENSG00000183495	EP400	1.3849	1.78E-17	1.54E-16	28.7736
ENSG00000141736	ERBB2	1.4139	1.82E-17	1.57E-16	28.7501
ENSG00000099385	BCL7C	1.3753	1.85E-17	1.59E-16	28.7359
ENSG00000136709	WDR33	1.3015	1.92E-17	1.66E-16	28.6986
ENSG00000244486	SCARF2	1.3480	1.97E-17	1.70E-16	28.6719
ENSG00000140525	FANCI	1.7150	1.97E-17	1.70E-16	28.6691
ENSG00000111276	CDKN1B	1.6921	2.00E-17	1.73E-16	28.6567
ENSG00000114850	SSR3	0.7060	2.01E-17	1.73E-16	28.6523
ENSG00000081019	RSBN1	1.3954	2.06E-17	1.78E-16	28.6272
ENSG00000162924	REL	1.6810	2.11E-17	1.82E-16	28.6043
ENSG00000130787	HIP1R	1.6282	2.19E-17	1.88E-16	28.5681
ENSG00000164236	ANKRD33B	1.9492	2.19E-17	1.88E-16	28.5678
ENSG00000198799	LRIG2	1.3729	2.20E-17	1.89E-16	28.5639
ENSG00000152767	FARP1	1.6389	2.20E-17	1.89E-16	28.5621
ENSG00000071082	RPL31	0.7077	2.20E-17	1.89E-16	28.5613
ENSG00000113595	TRIM23	1.4038	2.21E-17	1.90E-16	28.5567
ENSG00000166454	ATMIN	1.4273	2.23E-17	1.92E-16	28.5477
ENSG00000065060	UHRF1BP1	1.4019	2.25E-17	1.93E-16	28.5406
ENSG00000149806	FAU	0.7471	2.34E-17	2.01E-16	28.5025
ENSG00000112029	FBXO5	1.4996	2.35E-17	2.01E-16	28.4981
ENSG00000106686	SPATA6L	1.3626	2.36E-17	2.02E-16	28.4945
ENSG00000125743	SNRPD2	0.7477	2.36E-17	2.02E-16	28.4919
ENSG00000145088	EAF2	0.4514	2.40E-17	2.05E-16	28.4779
ENSG00000197879	MYO1C	1.6968	2.40E-17	2.06E-16	28.4762
ENSG00000166025	AMOTL1	1.7442	2.47E-17	2.11E-16	28.4484
ENSG00000128617	OPN1SW	1.5427	2.48E-17	2.12E-16	28.4453
ENSG00000124444	ZNF576	0.6752	2.53E-17	2.16E-16	28.4263
ENSG00000108064	TFAM	1.4072	2.55E-17	2.18E-16	28.4177
ENSG00000132003	ZSWIM4	1.4723	2.61E-17	2.23E-16	28.3953
ENSG00000072080	SPP2	1.6019	2.68E-17	2.29E-16	28.3676
ENSG00000150712	MTMR12	1.4285	2.69E-17	2.30E-16	28.3631
ENSG00000126215	XRCC3	1.5349	2.71E-17	2.31E-16	28.3556
ENSG00000166321	NUDT13	1.5184	2.72E-17	2.32E-16	28.3518
ENSG00000197343	ZNF655	1.3383	2.73E-17	2.33E-16	28.3495

Gene ID	Gene Symbol	FC	P.Value	adj.P.Val	B
ENSG00000084234	APLP2	1.6521	2.75E-17	2.34E-16	28.3425
ENSG00000169231	THBS3	1.6313	2.77E-17	2.36E-16	28.3345
ENSG00000135249	RINT1	1.3512	2.78E-17	2.37E-16	28.3312
ENSG00000131389	SLC6A6	1.7137	2.89E-17	2.46E-16	28.2922
ENSG00000163214	DHX57	1.3658	2.97E-17	2.52E-16	28.2683
ENSG00000177042	TMEM80	0.6077	3.00E-17	2.55E-16	28.2562
ENSG00000131791	PRKAB2	1.5524	3.04E-17	2.58E-16	28.2432
ENSG00000183145	RIPPLY3	1.0946	3.12E-17	2.65E-16	28.2178
ENSG00000196535	MYO18A	1.5065	3.12E-17	2.65E-16	28.2181
ENSG00000158955	WNT9B	1.0779	3.17E-17	2.69E-16	28.2032
ENSG00000135250	SRPK2	1.4467	3.21E-17	2.72E-16	28.1893
ENSG00000126524	SBDS	1.5555	3.23E-17	2.73E-16	28.1850
ENSG00000158290	CUL4B	1.4475	3.32E-17	2.81E-16	28.1581
ENSG00000112787	FBRSL1	1.4881	3.44E-17	2.92E-16	28.1208
ENSG00000198793	MTOR	1.3872	3.47E-17	2.94E-16	28.1126
ENSG00000124333	VAMP7	1.4978	3.51E-17	2.97E-16	28.1033
ENSG00000164334	FAM170A	0.8848	3.61E-17	3.05E-16	28.0747
ENSG00000148229	POLE3	1.3076	3.64E-17	3.08E-16	28.0659
ENSG00000197016	ZNF470	1.3069	3.65E-17	3.08E-16	28.0646
ENSG00000164181	ELOVL7	0.4807	3.74E-17	3.16E-16	28.0386
ENSG00000063169	BICRA	1.3926	3.78E-17	3.19E-16	28.0295
ENSG00000101856	PGRMC1	1.4709	3.80E-17	3.21E-16	28.0237
ENSG00000002586	CD99	3.4394	3.82E-17	3.22E-16	28.0177
ENSG00000157933	SKI	1.6916	3.82E-17	3.22E-16	28.0180
ENSG00000198563	DDX39B	1.3653	3.87E-17	3.26E-16	28.0070
ENSG00000117543	DPH5	0.6930	3.90E-17	3.29E-16	27.9977
ENSG00000175768	TOMM5	0.7216	3.91E-17	3.29E-16	27.9962
ENSG00000156968	MPV17L	1.4683	3.93E-17	3.31E-16	27.9900
ENSG00000143942	CHAC2	0.5191	3.97E-17	3.34E-16	27.9804
ENSG00000175591	P2RY2	0.5952	4.00E-17	3.36E-16	27.9742
ENSG00000110429	FBXO3	1.4660	4.04E-17	3.39E-16	27.9639
ENSG00000125944	HNRNPR	1.3216	4.08E-17	3.43E-16	27.9532
ENSG00000198612	COPS8	1.3041	4.09E-17	3.43E-16	27.9520
ENSG00000155363	MOV10	1.7569	4.09E-17	3.44E-16	27.9503
ENSG00000163681	SLMAP	1.3114	4.14E-17	3.47E-16	27.9396
ENSG00000162910	MRPL55	1.4201	4.19E-17	3.51E-16	27.9272
ENSG00000118503	TNFAIP3	2.5561	4.22E-17	3.54E-16	27.9199
ENSG00000161681	SHANK1	1.2097	4.24E-17	3.55E-16	27.9151
ENSG00000163006	CCDC138	1.5581	4.30E-17	3.60E-16	27.9027
ENSG00000171365	CLCN5	1.3954	4.36E-17	3.65E-16	27.8877
ENSG00000177885	GRB2	1.4095	4.37E-17	3.65E-16	27.8862
ENSG00000026103	FAS	2.2403	4.52E-17	3.78E-16	27.8536
ENSG00000117308	GALE	1.5907	4.52E-17	3.78E-16	27.8528
ENSG00000198355	PIM3	2.0961	4.53E-17	3.78E-16	27.8513
ENSG00000068305	MEF2A	1.5270	4.56E-17	3.81E-16	27.8431
ENSG00000138162	TACC2	1.2819	4.59E-17	3.83E-16	27.8382
ENSG00000070476	ZXDC	1.3756	4.65E-17	3.88E-16	27.8245
ENSG00000159588	CCDC17	1.3270	4.66E-17	3.88E-16	27.8234
ENSG00000130827	PLXNA3	1.8403	4.68E-17	3.90E-16	27.8188
ENSG00000253457	SMIM18	1.1531	4.87E-17	4.06E-16	27.7795
ENSG00000160223	ICOSLG	2.0021	4.98E-17	4.14E-16	27.7578
ENSG00000102384	CENPI	1.4877	5.00E-17	4.17E-16	27.7522
ENSG00000128655	PDE11A	1.1009	5.07E-17	4.22E-16	27.7402
ENSG00000164638	SLC29A4	1.1113	5.08E-17	4.23E-16	27.7368

Gene ID	Gene Symbol	FC	P.Value	adj.P.Val	B
ENSG00000122557	HERPUD2	1.2988	5.11E-17	4.25E-16	27.7323
ENSG00000188505	NCCRP1	1.0897	5.27E-17	4.38E-16	27.7015
ENSG00000253250	C8orf88	1.8706	5.34E-17	4.43E-16	27.6883
ENSG00000123179	EBPL	0.6478	5.39E-17	4.47E-16	27.6791
ENSG00000102393	GLA	1.6694	5.50E-17	4.57E-16	27.6589
ENSG00000166004	CEP295	1.4036	5.61E-17	4.65E-16	27.6399
ENSG00000167513	CDT1	1.7382	5.62E-17	4.66E-16	27.6378
ENSG00000113621	TXNDC15	0.6501	5.66E-17	4.69E-16	27.6314
ENSG00000161082	CELF5	1.1710	5.69E-17	4.71E-16	27.6261
ENSG00000172687	ZNF738	1.4769	5.69E-17	4.71E-16	27.6257
ENSG00000143294	PRCC	1.4381	5.84E-17	4.83E-16	27.6006
ENSG00000110925	CSRNP2	1.4068	5.93E-17	4.91E-16	27.5841
ENSG00000147118	ZNF182	1.3292	6.29E-17	5.20E-16	27.5274
ENSG00000173681	BCLAF3	1.4110	6.31E-17	5.21E-16	27.5244
ENSG00000114779	ABHD14B	0.6798	6.46E-17	5.34E-16	27.5009
ENSG00000198042	MAK16	1.4050	6.48E-17	5.35E-16	27.4978
ENSG00000133704	IPO8	1.3937	6.49E-17	5.36E-16	27.4959
ENSG00000141391	PRELID3A	1.3914	6.55E-17	5.40E-16	27.4875
ENSG00000166508	MCM7	1.4727	6.58E-17	5.43E-16	27.4817
ENSG00000096717	SIRT1	1.4030	6.65E-17	5.48E-16	27.4719
ENSG00000069275	NUCKS1	1.6275	6.65E-17	5.48E-16	27.4714
ENSG00000142227	EMP3	1.9333	6.79E-17	5.59E-16	27.4519
ENSG00000144231	POLR2D	1.3519	6.86E-17	5.65E-16	27.4410
ENSG00000111860	CEP85L	1.5150	6.95E-17	5.72E-16	27.4286
ENSG00000134490	TMEM241	1.5232	7.14E-17	5.87E-16	27.4024
ENSG00000105875	WDR91	1.4782	7.16E-17	5.89E-16	27.3991
ENSG00000132434	LANCL2	1.4262	7.21E-17	5.92E-16	27.3928
ENSG00000102290	PCDH11X	1.1876	7.22E-17	5.93E-16	27.3903
ENSG00000088305	DNMT3B	1.5179	7.24E-17	5.94E-16	27.3885
ENSG00000149571	KIRREL3	1.2288	7.48E-17	6.14E-16	27.3562
ENSG00000171503	ETFDH	1.4303	7.51E-17	6.16E-16	27.3518
ENSG00000186105	LRRC70	1.6723	7.55E-17	6.19E-16	27.3468
ENSG00000136051	WASHC4	1.4165	7.61E-17	6.24E-16	27.3390
ENSG00000150907	FOXO1	1.6440	7.66E-17	6.28E-16	27.3325
ENSG00000167196	FBXO22	0.5954	7.66E-17	6.28E-16	27.3320
ENSG00000175336	APOF	1.1328	7.77E-17	6.36E-16	27.3180
ENSG00000113456	RAD1	1.3358	7.81E-17	6.39E-16	27.3141
ENSG00000095951	HIVEP1	1.6038	7.95E-17	6.50E-16	27.2959
ENSG00000115419	GLS	1.4481	7.98E-17	6.52E-16	27.2925
ENSG00000103342	GSPT1	1.3500	8.10E-17	6.62E-16	27.2775
ENSG00000173991	TCAP	1.3891	8.15E-17	6.66E-16	27.2716
ENSG00000168883	USP39	1.2836	8.18E-17	6.68E-16	27.2684
ENSG00000105373	NOP53	0.6137	8.29E-17	6.77E-16	27.2542
ENSG00000131504	DIAPH1	1.3976	8.32E-17	6.79E-16	27.2508
ENSG00000189030	VHLL	1.1050	8.33E-17	6.79E-16	27.2501
ENSG00000076555	ACACB	1.7943	8.62E-17	7.02E-16	27.2166
ENSG00000198393	ZNF26	1.5638	8.61E-17	7.02E-16	27.2168
ENSG00000182307	C8orf33	1.3488	8.63E-17	7.03E-16	27.2155
ENSG00000085872	CHERP	1.3781	8.87E-17	7.22E-16	27.1884
ENSG00000261652	C15orf65	0.6192	8.90E-17	7.24E-16	27.1849
ENSG00000100416	TRMU	1.4113	8.91E-17	7.25E-16	27.1833
ENSG00000139998	RAB15	2.1570	9.09E-17	7.39E-16	27.1639
ENSG00000120705	ETF1	1.3316	9.17E-17	7.45E-16	27.1554
ENSG00000182324	KCNJ14	1.3168	9.29E-17	7.55E-16	27.1424

Gene ID	Gene Symbol	FC	P.Value	adj.P.Val	B
ENSG00000154222	CC2D1B	1.4115	9.46E-17	7.68E-16	27.1244
ENSG00000065978	YBX1	1.2729	9.49E-17	7.70E-16	27.1218
ENSG00000138867	GUCD1	1.3766	9.58E-17	7.77E-16	27.1124
ENSG00000171596	NMUR1	1.1144	9.64E-17	7.81E-16	27.1064
ENSG00000176170	SPHK1	2.1383	1.02E-16	8.27E-16	27.0496
ENSG00000121039	RDH10	1.4630	1.02E-16	8.30E-16	27.0456
ENSG00000018510	AGPS	1.3524	1.07E-16	8.66E-16	27.0034
ENSG00000175183	CSRP2	1.2549	1.07E-16	8.69E-16	26.9995
ENSG00000090372	STRN4	1.3340	1.09E-16	8.82E-16	26.9848
ENSG00000124092	CTCFL	1.2828	1.14E-16	9.19E-16	26.9434
ENSG00000135679	MDM2	1.6543	1.15E-16	9.30E-16	26.9313
ENSG00000107140	TESK1	1.4519	1.15E-16	9.31E-16	26.9299
ENSG00000111145	ELK3	1.5421	1.16E-16	9.39E-16	26.9217
ENSG00000104892	KLC3	1.2167	1.16E-16	9.40E-16	26.9200
ENSG00000071626	DAZAP1	1.2649	1.17E-16	9.43E-16	26.9167
ENSG00000143537	ADAM15	1.5617	1.18E-16	9.54E-16	26.9042
ENSG00000189401	OTUD6A	1.0671	1.19E-16	9.56E-16	26.9021
ENSG00000105835	NAMPT	2.2209	1.19E-16	9.57E-16	26.9005
ENSG00000120071	KANSL1	1.4636	1.22E-16	9.85E-16	26.8722
ENSG00000161652	IZUMO2	1.1821	1.24E-16	9.96E-16	26.8605
ENSG00000073111	MCM2	1.8850	1.24E-16	1.00E-15	26.8565
ENSG00000169679	BUB1	1.8569	1.25E-16	1.01E-15	26.8498
ENSG00000100994	PYGB	1.5995	1.26E-16	1.01E-15	26.8417
ENSG00000175727	MLXIP	1.4831	1.27E-16	1.02E-15	26.8336
ENSG00000174749	FAM241A	1.3621	1.32E-16	1.06E-15	26.7998
ENSG00000139287	TPH2	1.2963	1.32E-16	1.06E-15	26.7934
ENSG00000261609	GAN	1.1976	1.33E-16	1.07E-15	26.7880
ENSG00000105202	FBL	0.7050	1.33E-16	1.07E-15	26.7851
ENSG00000160058	BSDC1	1.3584	1.36E-16	1.09E-15	26.7647
ENSG00000154001	PPP2R5E	1.3884	1.39E-16	1.12E-15	26.7430
ENSG00000197261	C6orf141	1.1581	1.40E-16	1.12E-15	26.7416
ENSG00000124181	PLCG1	1.5556	1.40E-16	1.12E-15	26.7380
ENSG00000104714	ERICH1	1.5099	1.44E-16	1.16E-15	26.7080
ENSG00000118181	RPS25	0.7165	1.45E-16	1.16E-15	26.7036
ENSG00000141086	CTRL	1.5493	1.45E-16	1.16E-15	26.7025
ENSG00000080845	DLGAP4	1.4942	1.46E-16	1.17E-15	26.6986
ENSG00000109832	DDX25	1.1863	1.46E-16	1.17E-15	26.6951
ENSG00000205857	NANOGNB	1.0909	1.46E-16	1.17E-15	26.6943
ENSG00000058091	CDK14	1.5465	1.47E-16	1.17E-15	26.6928
ENSG00000140612	SEC11A	0.7287	1.49E-16	1.19E-15	26.6785
ENSG00000196365	LONP1	1.3810	1.52E-16	1.21E-15	26.6601
ENSG00000131845	ZNF304	1.4802	1.52E-16	1.22E-15	26.6552
ENSG00000196844	PATE2	1.1153	1.54E-16	1.23E-15	26.6465
ENSG00000150347	ARID5B	2.1128	1.59E-16	1.26E-15	26.6157
ENSG00000157399	ARSL	1.3391	1.65E-16	1.32E-15	26.5740
ENSG00000136371	MTHFS	0.6652	1.66E-16	1.32E-15	26.5721
ENSG00000101695	RNF125	1.5202	1.69E-16	1.35E-15	26.5513
ENSG00000182117	NOP10	0.6822	1.70E-16	1.35E-15	26.5493
ENSG00000139324	TMTC3	1.3308	1.70E-16	1.35E-15	26.5465
ENSG00000127920	GNG11	0.3930	1.71E-16	1.36E-15	26.5435
ENSG00000173597	SULT1B1	1.3253	1.71E-16	1.36E-15	26.5388
ENSG00000155090	KLF10	1.9577	1.77E-16	1.40E-15	26.5080
ENSG00000106554	CHCHD3	1.2719	1.81E-16	1.44E-15	26.4853
ENSG00000162438	CTRC	1.2903	1.81E-16	1.44E-15	26.4854

Gene ID	Gene Symbol	FC	P.Value	adj.P.Val	B
ENSG00000161547	SRSF2	1.3756	1.87E-16	1.48E-15	26.4545
ENSG00000109686	SH3D19	1.7505	1.93E-16	1.53E-15	26.4239
ENSG00000157927	RADIL	1.1356	1.97E-16	1.56E-15	26.4023
ENSG00000065357	DGKA	1.6215	1.98E-16	1.57E-15	26.3967
ENSG00000134265	NAPG	1.3591	2.02E-16	1.60E-15	26.3789
ENSG00000006756	ARSD	1.5360	2.06E-16	1.63E-15	26.3588
ENSG00000153767	GTF2E1	1.3855	2.07E-16	1.64E-15	26.3545
ENSG00000126733	DACH2	1.1548	2.07E-16	1.64E-15	26.3519
ENSG00000071894	CPSF1	1.4828	2.08E-16	1.64E-15	26.3492
ENSG00000149761	NUDT22	0.7055	2.09E-16	1.65E-15	26.3451
ENSG00000160293	VAV2	1.8952	2.09E-16	1.65E-15	26.3446
ENSG00000187098	MITF	1.4874	2.15E-16	1.70E-15	26.3161
ENSG00000173080	RXFP4	1.2408	2.16E-16	1.71E-15	26.3090
ENSG00000118515	SGK1	2.2879	2.17E-16	1.71E-15	26.3081
ENSG00000166707	ZCCHC18	1.3913	2.19E-16	1.73E-15	26.2979
ENSG00000125347	IRF1	1.8482	2.19E-16	1.73E-15	26.2967
ENSG00000108306	FBXL20	1.3962	2.23E-16	1.75E-15	26.2809
ENSG00000123119	NECAB1	1.3973	2.25E-16	1.77E-15	26.2703
ENSG00000267041	ZNF850	1.1607	2.30E-16	1.81E-15	26.2488
ENSG00000124664	SPDEF	1.4980	2.40E-16	1.89E-15	26.2089
ENSG00000036549	ZZZ3	1.3008	2.42E-16	1.91E-15	26.1983
ENSG00000148408	CACNA1B	1.2909	2.42E-16	1.91E-15	26.1975
ENSG00000175505	CLCF1	1.7643	2.47E-16	1.95E-15	26.1770
ENSG00000204421	LY6G6C	1.3239	2.55E-16	2.00E-15	26.1483
ENSG00000132510	KDM6B	1.6725	2.57E-16	2.02E-15	26.1411
ENSG00000161835	TAMALIN	3.3436	2.61E-16	2.05E-15	26.1248
ENSG00000003402	CFLAR	1.6710	2.64E-16	2.07E-15	26.1124
ENSG00000155380	SLC16A1	1.4638	2.65E-16	2.08E-15	26.1097
ENSG00000131788	PIAS3	1.4694	2.69E-16	2.11E-15	26.0948
ENSG00000149609	C20orf144	1.1938	2.71E-16	2.13E-15	26.0862
ENSG00000154803	FLCN	1.5114	2.75E-16	2.16E-15	26.0724
ENSG00000163075	CFAP221	1.1698	2.75E-16	2.16E-15	26.0719
ENSG00000142149	HUNK	1.0505	2.77E-16	2.17E-15	26.0655
ENSG00000085433	WDR47	1.2785	2.82E-16	2.20E-15	26.0495
ENSG00000137343	ATAT1	1.4450	2.90E-16	2.27E-15	26.0190
ENSG00000197937	ZNF347	1.5349	2.91E-16	2.27E-15	26.0179
ENSG00000117360	PRPF3	1.4167	2.92E-16	2.28E-15	26.0152
ENSG00000169035	KLK7	1.0467	2.99E-16	2.33E-15	25.9911
ENSG00000157741	UBN2	1.3701	3.01E-16	2.35E-15	25.9825
ENSG00000169635	HIC2	1.3320	3.03E-16	2.36E-15	25.9790
ENSG00000165704	HPRT1	1.5433	3.05E-16	2.38E-15	25.9710
ENSG00000177971	IMP3	0.6564	3.07E-16	2.39E-15	25.9645
ENSG00000204842	ATXN2	1.3633	3.10E-16	2.41E-15	25.9564
ENSG00000175544	CABP4	1.4153	3.15E-16	2.46E-15	25.9378
ENSG00000149115	TNKS1BP1	1.4267	3.16E-16	2.46E-15	25.9353
ENSG00000231738	TSPAN19	1.5561	3.18E-16	2.47E-15	25.9308
ENSG00000171189	GRIK1	1.1027	3.19E-16	2.48E-15	25.9265
ENSG00000120837	NFYB	1.3554	3.27E-16	2.54E-15	25.9027
ENSG00000167526	RPL13	0.6960	3.29E-16	2.56E-15	25.8967
ENSG00000166823	MESP1	0.5954	3.30E-16	2.57E-15	25.8919
ENSG00000217930	PAM16	0.7549	3.31E-16	2.57E-15	25.8893
ENSG00000177000	MTHFR	1.4983	3.32E-16	2.58E-15	25.8888
ENSG00000166340	TPP1	1.5305	3.38E-16	2.62E-15	25.8705
ENSG00000101850	GPR143	1.2372	3.43E-16	2.66E-15	25.8561

Gene ID	Gene Symbol	FC	P.Value	adj.P.Val	B
ENSG00000180773	SLC36A4	1.5128	3.43E-16	2.66E-15	25.8538
ENSG00000109771	LRP2BP	1.4099	3.44E-16	2.67E-15	25.8527
ENSG00000169047	IRS1	1.7688	3.46E-16	2.68E-15	25.8466
ENSG00000165996	HACD1	1.2727	3.52E-16	2.73E-15	25.8289
ENSG00000171617	ENC1	2.0772	3.55E-16	2.75E-15	25.8204
ENSG00000167699	GLOD4	1.3807	3.56E-16	2.76E-15	25.8178
ENSG00000087008	ACOX3	1.4547	3.61E-16	2.79E-15	25.8057
ENSG00000090615	GOLGA3	1.4671	3.65E-16	2.82E-15	25.7952
ENSG00000118513	MYB	1.9388	3.68E-16	2.84E-15	25.7863
ENSG00000147155	EBP	1.3622	3.72E-16	2.87E-15	25.7756
ENSG00000137876	RSL24D1	0.7382	3.74E-16	2.89E-15	25.7711
ENSG00000148834	GSTO1	0.7121	3.87E-16	2.99E-15	25.7367
ENSG00000137106	GRHPR	0.7133	3.90E-16	3.01E-15	25.7282
ENSG00000106799	TGFBR1	1.4427	4.02E-16	3.10E-15	25.6988
ENSG00000156256	USP16	1.3049	4.02E-16	3.10E-15	25.6986
ENSG00000115355	CCDC88A	1.8246	4.03E-16	3.11E-15	25.6959
ENSG00000197045	GMFB	1.4611	4.06E-16	3.13E-15	25.6882
ENSG00000185055	EFCAB10	1.2663	4.07E-16	3.14E-15	25.6862
ENSG00000165646	SLC18A2	1.2712	4.09E-16	3.15E-15	25.6813
ENSG00000178295	GEN1	1.6398	4.09E-16	3.15E-15	25.6814
ENSG00000006327	TNFRSF12A	1.9444	4.10E-16	3.16E-15	25.6790
ENSG00000005889	ZFX	1.3812	4.15E-16	3.19E-15	25.6685
ENSG00000198728	LDB1	1.3517	4.22E-16	3.24E-15	25.6509
ENSG00000180488	MIGA1	1.2901	4.37E-16	3.36E-15	25.6166
ENSG00000146232	NFKBIE	1.9950	4.38E-16	3.37E-15	25.6139
ENSG00000186063	AIDA	1.3855	4.39E-16	3.37E-15	25.6113
ENSG00000155287	SLC25A28	1.3956	4.44E-16	3.41E-15	25.6014
ENSG00000153048	CARHSP1	1.4568	4.49E-16	3.44E-15	25.5900
ENSG00000164402	SEPTIN8	1.9681	4.53E-16	3.47E-15	25.5820
ENSG00000171119	NRTN	2.1970	4.55E-16	3.49E-15	25.5774
ENSG00000103642	LACTB	1.6299	4.61E-16	3.53E-15	25.5635
ENSG00000067829	IDH3G	1.4104	4.80E-16	3.68E-15	25.5242
ENSG00000169246	NPIPB3	1.6473	4.91E-16	3.76E-15	25.5022
ENSG00000188227	ZNF793	1.3349	4.91E-16	3.76E-15	25.5023
ENSG00000136381	IREB2	1.3886	5.04E-16	3.85E-15	25.4769
ENSG00000112245	PTP4A1	1.5469	5.07E-16	3.88E-15	25.4695
ENSG00000163605	PPP4R2	1.3044	5.15E-16	3.94E-15	25.4540
ENSG00000142751	GPN2	1.3458	5.17E-16	3.95E-15	25.4511
ENSG00000101126	ADNP	1.3550	5.22E-16	3.99E-15	25.4407
ENSG00000111837	MAK	1.1967	5.25E-16	4.01E-15	25.4355
ENSG00000160161	CILP2	1.1672	5.25E-16	4.01E-15	25.4350
ENSG00000164032	H2AZ1	1.4438	5.39E-16	4.11E-15	25.4096
ENSG00000100038	TOP3B	1.4450	5.43E-16	4.14E-15	25.4029
ENSG00000139684	ESD	0.6758	5.62E-16	4.28E-15	25.3695
ENSG00000115310	RTN4	1.4361	5.65E-16	4.30E-15	25.3644
ENSG00000138463	SLC49A4	1.4153	5.72E-16	4.36E-15	25.3508
ENSG00000105722	ERF	1.5720	5.81E-16	4.42E-15	25.3360
ENSG00000204634	TBC1D8	1.5997	5.83E-16	4.44E-15	25.3328
ENSG00000180336	MEIOC	1.3230	5.84E-16	4.44E-15	25.3308
ENSG00000188647	PTAR1	1.3978	5.85E-16	4.45E-15	25.3290
ENSG00000101224	CDC25B	1.6449	5.93E-16	4.51E-15	25.3158
ENSG00000141503	MINK1	1.4553	5.95E-16	4.52E-15	25.3127
ENSG00000100916	BRMS1L	1.4402	5.95E-16	4.52E-15	25.3122
ENSG00000196118	CCDC189	1.6440	5.96E-16	4.53E-15	25.3108

Gene ID	Gene Symbol	FC	P.Value	adj.P.Val	B
ENSG00000157992	KRTCAP3	1.6872	6.00E-16	4.55E-15	25.3041
ENSG00000183287	CCBE1	1.1641	6.09E-16	4.62E-15	25.2900
ENSG00000179456	ZBTB18	1.7217	6.16E-16	4.67E-15	25.2783
ENSG00000234719	NPIPB2	1.6199	6.22E-16	4.72E-15	25.2687
ENSG00000182473	EXOC7	1.3559	6.36E-16	4.82E-15	25.2476
ENSG00000185519	FAM131C	1.2305	6.37E-16	4.82E-15	25.2460
ENSG00000118496	FBXO30	1.4397	6.40E-16	4.84E-15	25.2407
ENSG00000134504	KCTD1	0.5945	6.40E-16	4.84E-15	25.2407
ENSG00000138592	USP8	1.3426	6.82E-16	5.16E-15	25.1784
ENSG00000165338	HECTD2	1.5956	6.83E-16	5.16E-15	25.1768
ENSG00000173825	TIGD3	1.3353	6.85E-16	5.18E-15	25.1736
ENSG00000129353	SLC44A2	2.2418	6.93E-16	5.24E-15	25.1619
ENSG00000249222	ATP5MGL	1.2917	7.15E-16	5.40E-15	25.1315
ENSG00000084734	GCKR	1.1607	7.27E-16	5.49E-15	25.1149
ENSG00000123992	DNPEP	1.3371	7.42E-16	5.60E-15	25.0957
ENSG00000123728	RAP2C	1.4878	7.54E-16	5.69E-15	25.0795
ENSG00000175155	YPEL2	1.5784	7.60E-16	5.73E-15	25.0717
ENSG00000185811	IKZF1	1.4051	7.61E-16	5.74E-15	25.0699
ENSG00000168826	ZBTB49	1.4099	7.71E-16	5.81E-15	25.0573
ENSG00000103197	TSC2	1.4176	7.75E-16	5.83E-15	25.0528
ENSG00000186188	FFAR4	1.0585	7.76E-16	5.84E-15	25.0511
ENSG00000185246	PRPF39	1.3484	7.84E-16	5.90E-15	25.0415
ENSG00000204866	IGFL2	1.1680	7.86E-16	5.91E-15	25.0388
ENSG00000110721	CHKA	1.4623	7.88E-16	5.93E-15	25.0354
ENSG00000174446	SNAPC5	0.7104	7.99E-16	6.01E-15	25.0219
ENSG00000154479	CCDC173	1.5868	8.01E-16	6.02E-15	25.0201
ENSG00000006704	GTF2IRD1	1.4245	8.03E-16	6.03E-15	25.0178
ENSG00000109445	ZNF330	1.3511	8.05E-16	6.05E-15	25.0145
ENSG00000143384	MCL1	1.6782	8.06E-16	6.05E-15	25.0142
ENSG00000119760	SUPT7L	1.3100	8.08E-16	6.06E-15	25.0112
ENSG00000158158	CNNM4	1.4227	8.09E-16	6.07E-15	25.0096
ENSG00000140374	ETFA	0.7149	8.16E-16	6.12E-15	25.0011
ENSG00000118997	DNAH7	1.3464	8.26E-16	6.19E-15	24.9892
ENSG00000272333	KMT2B	1.3781	8.47E-16	6.34E-15	24.9646
ENSG00000186329	TMEM212	1.3970	8.90E-16	6.66E-15	24.9164
ENSG00000104885	DOT1L	1.4373	9.13E-16	6.83E-15	24.8906
ENSG00000089639	GMIP	1.4568	9.16E-16	6.85E-15	24.8875
ENSG00000129951	PLPPR3	1.2712	9.28E-16	6.94E-15	24.8752
ENSG00000142677	IL22RA1	1.5138	9.30E-16	6.95E-15	24.8731
ENSG00000117385	P3H1	1.4084	9.32E-16	6.96E-15	24.8709
ENSG00000123684	LPGAT1	1.7104	9.51E-16	7.10E-15	24.8512
ENSG00000124151	NCOA3	1.6100	9.51E-16	7.10E-15	24.8506
ENSG00000213420	GPC2	1.3059	9.52E-16	7.10E-15	24.8499
ENSG00000057757	PITHD1	1.2699	9.65E-16	7.20E-15	24.8364
ENSG00000145194	ECE2	0.5874	9.73E-16	7.26E-15	24.8278
ENSG00000099795	NDUFB7	0.7434	9.83E-16	7.33E-15	24.8177
ENSG00000162461	SLC25A34	1.3479	9.94E-16	7.40E-15	24.8075
ENSG00000125676	THOC2	1.3881	1.00E-15	7.47E-15	24.7984
ENSG00000105219	CCNP	1.4794	1.03E-15	7.69E-15	24.7698
ENSG00000170509	HSD17B13	1.1282	1.04E-15	7.72E-15	24.7648
ENSG00000161381	PLXDC1	1.4106	1.05E-15	7.82E-15	24.7516
ENSG00000117724	CENPF	1.7258	1.06E-15	7.90E-15	24.7422
ENSG00000169018	FEM1B	1.4628	1.06E-15	7.90E-15	24.7407
ENSG00000005189	REXO5	1.5100	1.07E-15	7.91E-15	24.7392

Gene ID	Gene Symbol	FC	P.Value	adj.P.Val	B
ENSG00000164815	ORC5	0.6375	1.09E-15	8.06E-15	24.7202
ENSG00000109805	NCAPG	1.7017	1.13E-15	8.38E-15	24.6826
ENSG00000188996	HUS1B	1.2574	1.13E-15	8.38E-15	24.6820
ENSG00000099875	MKNK2	1.5853	1.14E-15	8.45E-15	24.6733
ENSG00000148219	ASTN2	1.4057	1.14E-15	8.45E-15	24.6722
ENSG00000110987	BCL7A	1.3349	1.14E-15	8.47E-15	24.6696
ENSG00000147231	RADX	1.9335	1.16E-15	8.61E-15	24.6535
ENSG00000141258	SGSM2	1.5456	1.16E-15	8.61E-15	24.6525
ENSG00000140511	HAPLN3	2.0004	1.17E-15	8.68E-15	24.6446
ENSG00000166619	BLCAP	1.3367	1.17E-15	8.68E-15	24.6439
ENSG00000176095	IP6K1	1.4153	1.21E-15	8.92E-15	24.6175
ENSG00000104979	C19orf53	0.7879	1.21E-15	8.96E-15	24.6126
ENSG00000163482	STK36	1.6052	1.22E-15	9.02E-15	24.6050
ENSG00000196367	TRRAP	1.4181	1.22E-15	9.05E-15	24.6015
ENSG00000112739	PRPF4B	1.3696	1.23E-15	9.05E-15	24.6009
ENSG00000163565	IFI16	1.5631	1.27E-15	9.37E-15	24.5670
ENSG00000103375	AQP8	1.1721	1.27E-15	9.38E-15	24.5650
ENSG00000136925	TSTD2	1.4217	1.27E-15	9.38E-15	24.5651
ENSG00000125633	CCDC93	1.4736	1.29E-15	9.51E-15	24.5510
ENSG00000126895	AVPR2	1.4329	1.30E-15	9.59E-15	24.5425
ENSG00000145375	SPATA5	1.3047	1.30E-15	9.61E-15	24.5398
ENSG00000189157	FAM47E	1.2869	1.32E-15	9.74E-15	24.5259
ENSG00000102241	HTATSF1	1.4560	1.33E-15	9.78E-15	24.5217
ENSG00000151882	CCL28	1.3868	1.35E-15	9.92E-15	24.5076
ENSG00000099864	PALM	1.1105	1.35E-15	9.95E-15	24.5040
ENSG00000099942	CRKL	1.3805	1.42E-15	1.04E-14	24.4576
ENSG00000188112	C6orf132	1.1243	1.42E-15	1.05E-14	24.4543
ENSG00000188177	ZC3H6	1.4483	1.43E-15	1.05E-14	24.4521
ENSG00000129990	SYT5	1.1328	1.43E-15	1.05E-14	24.4472
ENSG00000132613	MTSS2	1.5230	1.43E-15	1.05E-14	24.4474
ENSG00000185634	SHC4	1.2667	1.44E-15	1.06E-14	24.4413
ENSG00000099995	SF3A1	1.4340	1.45E-15	1.06E-14	24.4358
ENSG00000089356	FXYD3	1.2650	1.46E-15	1.07E-14	24.4312
ENSG00000130513	GDF15	1.4761	1.47E-15	1.08E-14	24.4201
ENSG00000198099	ADH4	1.1557	1.49E-15	1.09E-14	24.4100
ENSG00000174738	NR1D2	1.5061	1.51E-15	1.11E-14	24.3961
ENSG00000166592	RRAD	2.7046	1.51E-15	1.11E-14	24.3925
ENSG00000068120	COASY	1.3793	1.62E-15	1.18E-14	24.3289
ENSG00000147883	CDKN2B	1.7287	1.64E-15	1.20E-14	24.3156
ENSG00000100336	APOL4	1.3191	1.68E-15	1.23E-14	24.2928
ENSG00000164172	MOCS2	0.6153	1.68E-15	1.23E-14	24.2895
ENSG00000110060	PUS3	0.6806	1.71E-15	1.25E-14	24.2745
ENSG00000183347	GBP6	1.1281	1.73E-15	1.27E-14	24.2600
ENSG00000180530	NRIP1	1.6814	1.76E-15	1.29E-14	24.2423
ENSG00000181090	EHMT1	1.3538	1.81E-15	1.32E-14	24.2161
ENSG00000146535	GNA12	1.4815	1.84E-15	1.34E-14	24.2008
ENSG00000179335	CLK3	1.3887	1.90E-15	1.39E-14	24.1692
ENSG00000128805	ARHGAP22	1.3688	1.98E-15	1.45E-14	24.1263
ENSG00000107968	MAP3K8	2.5343	1.99E-15	1.45E-14	24.1243
ENSG00000167283	ATP5MG	0.7583	1.99E-15	1.45E-14	24.1241
ENSG00000113231	PDE8B	0.7688	2.06E-15	1.50E-14	24.0889
ENSG00000156170	NDUFAF6	1.3950	2.06E-15	1.50E-14	24.0889
ENSG00000105341	DMAC2	0.7432	2.07E-15	1.51E-14	24.0831
ENSG00000176994	SMCR8	1.5370	2.09E-15	1.52E-14	24.0739

Gene ID	Gene Symbol	FC	P.Value	adj.P.Val	B
ENSG00000166439	RNF169	1.4186	2.10E-15	1.53E-14	24.0715
ENSG00000121904	CSMD2	1.1283	2.12E-15	1.54E-14	24.0611
ENSG00000135924	DNAJB2	1.4101	2.16E-15	1.57E-14	24.0408
ENSG00000151465	CDC123	1.2675	2.17E-15	1.57E-14	24.0392
ENSG00000101447	FAM83D	1.4585	2.17E-15	1.58E-14	24.0367
ENSG00000214595	EML6	1.9156	2.18E-15	1.59E-14	24.0322
ENSG00000158864	NDUFS2	1.3802	2.22E-15	1.61E-14	24.0164
ENSG00000133275	CSNK1G2	1.3231	2.23E-15	1.62E-14	24.0097
ENSG00000100941	PNN	1.4245	2.24E-15	1.62E-14	24.0085
ENSG00000092470	WDR76	1.5630	2.25E-15	1.63E-14	24.0037
ENSG00000116030	SUMO1	1.3105	2.27E-15	1.64E-14	23.9956
ENSG00000163435	ELF3	1.9482	2.27E-15	1.64E-14	23.9938
ENSG00000167757	KLK11	1.1002	2.29E-15	1.66E-14	23.9834
ENSG00000175449	RFESD	0.6446	2.29E-15	1.66E-14	23.9834
ENSG00000108829	LRRC59	1.3694	2.32E-15	1.68E-14	23.9706
ENSG00000140443	IGF1R	1.8506	2.33E-15	1.68E-14	23.9683
ENSG00000167779	IGFBP6	1.7246	2.39E-15	1.73E-14	23.9426
ENSG00000133639	BTG1	1.6714	2.44E-15	1.76E-14	23.9233
ENSG00000111666	CHPT1	1.9122	2.45E-15	1.77E-14	23.9186
ENSG00000100519	PSMC6	1.3115	2.53E-15	1.82E-14	23.8885
ENSG00000114520	SNX4	1.2766	2.54E-15	1.83E-14	23.8842
ENSG00000132740	IGHMBP2	1.3590	2.54E-15	1.83E-14	23.8840
ENSG00000088205	DDX18	1.3639	2.60E-15	1.87E-14	23.8612
ENSG00000140743	CDR2	1.3663	2.61E-15	1.88E-14	23.8563
ENSG00000182810	DDX28	1.3444	2.67E-15	1.92E-14	23.8356
ENSG00000108639	SYNGR2	1.5781	2.75E-15	1.98E-14	23.8061
ENSG00000144224	UBXN4	1.3421	2.77E-15	1.99E-14	23.7983
ENSG00000060069	CTDP1	1.3215	2.81E-15	2.02E-14	23.7856
ENSG00000111676	ATN1	1.4339	2.82E-15	2.03E-14	23.7807
ENSG00000175467	SART1	1.3212	2.85E-15	2.05E-14	23.7687
ENSG00000105323	HNRNPUL1	1.2901	2.90E-15	2.09E-14	23.7519
ENSG00000198517	MAFK	1.5970	2.92E-15	2.10E-14	23.7467
ENSG00000019144	PHLDB1	1.2565	2.94E-15	2.11E-14	23.7381
ENSG00000198380	GFPT1	1.4845	3.00E-15	2.15E-14	23.7211
ENSG00000131669	NINJ1	2.0399	3.04E-15	2.18E-14	23.7059
ENSG00000129933	MAU2	1.3851	3.08E-15	2.21E-14	23.6952
ENSG00000033178	UBA6	1.3883	3.10E-15	2.22E-14	23.6873
ENSG00000113361	CDH6	1.0730	3.29E-15	2.36E-14	23.6277
ENSG00000164329	TENT2	1.2849	3.41E-15	2.44E-14	23.5948
ENSG00000083937	CHMP2B	1.2980	3.41E-15	2.44E-14	23.5929
ENSG00000111906	HDDC2	0.6917	3.45E-15	2.47E-14	23.5826
ENSG00000184206	GOLGA6L4	1.4837	3.48E-15	2.49E-14	23.5742
ENSG00000168140	VASN	1.4168	3.53E-15	2.52E-14	23.5607
ENSG00000100097	LGALS1	1.8738	3.53E-15	2.53E-14	23.5592
ENSG00000057935	MTA3	1.4384	3.55E-15	2.54E-14	23.5531
ENSG00000145012	LPP	1.3893	3.59E-15	2.57E-14	23.5422
ENSG00000142599	RERE	1.5754	3.60E-15	2.57E-14	23.5400
ENSG00000139496	NUP58	1.3476	3.61E-15	2.58E-14	23.5365
ENSG00000100207	TCF20	1.3570	3.62E-15	2.58E-14	23.5359
ENSG00000131748	STARD3	1.2847	3.62E-15	2.58E-14	23.5348
ENSG00000102125	TAFAZZIN	1.5196	3.62E-15	2.59E-14	23.5336
ENSG00000105655	ISYNA1	1.8151	3.66E-15	2.61E-14	23.5252
ENSG00000101639	CEP192	1.3827	3.77E-15	2.69E-14	23.4941
ENSG00000101928	MOSPD1	1.3654	3.78E-15	2.69E-14	23.4917

Gene ID	Gene Symbol	FC	P.Value	adj.P.Val	B
ENSG00000012822	CALCOCO1	1.5143	3.88E-15	2.76E-14	23.4665
ENSG00000119138	KLF9	2.3309	3.90E-15	2.78E-14	23.4615
ENSG00000181045	SLC26A11	1.5282	3.91E-15	2.78E-14	23.4599
ENSG00000174038	C9orf131	1.4012	3.92E-15	2.79E-14	23.4569
ENSG00000177917	ARL6IP6	1.4737	3.93E-15	2.80E-14	23.4529
ENSG00000119421	NDUFA8	0.6225	3.94E-15	2.80E-14	23.4520
ENSG00000055332	EIF2AK2	1.5021	3.94E-15	2.80E-14	23.4514
ENSG00000133835	HSD17B4	0.6042	3.94E-15	2.80E-14	23.4505
ENSG00000204316	MRPL38	1.3076	3.98E-15	2.83E-14	23.4416
ENSG00000135956	TMEM127	1.3700	4.02E-15	2.85E-14	23.4325
ENSG00000148719	DNAJB12	1.2480	4.08E-15	2.89E-14	23.4172
ENSG00000174371	EXO1	1.6598	4.13E-15	2.93E-14	23.4045
ENSG00000110047	EHD1	2.5695	4.14E-15	2.93E-14	23.4038
ENSG00000189057	FAM111B	1.6200	4.15E-15	2.94E-14	23.3996
ENSG00000149480	MTA2	1.3616	4.28E-15	3.03E-14	23.3703
ENSG00000197958	RPL12	0.7093	4.28E-15	3.03E-14	23.3703
ENSG00000077684	JADE1	1.4175	4.29E-15	3.04E-14	23.3667
ENSG00000070770	CSNK2A2	1.3138	4.32E-15	3.06E-14	23.3610
ENSG00000125733	TRIP10	1.5344	4.33E-15	3.07E-14	23.3579
ENSG00000129484	PARP2	1.3648	4.37E-15	3.09E-14	23.3487
ENSG00000160321	ZNF208	1.3586	4.43E-15	3.13E-14	23.3369
ENSG00000166900	STX3	1.6477	4.55E-15	3.22E-14	23.3094
ENSG00000129757	CDKN1C	2.4292	4.56E-15	3.22E-14	23.3068
ENSG00000123091	RNF11	1.5017	4.68E-15	3.30E-14	23.2828
ENSG00000130559	CAMSAP1	1.4031	4.69E-15	3.31E-14	23.2799
ENSG00000044446	PHKA2	1.3951	4.75E-15	3.35E-14	23.2671
ENSG00000136542	GALNT5	1.6525	4.78E-15	3.37E-14	23.2604
ENSG00000144233	AMMECR1L	1.3021	4.82E-15	3.40E-14	23.2527
ENSG00000198718	TOGARAM1	1.3754	4.84E-15	3.41E-14	23.2492
ENSG00000108443	RPS6KB1	1.3475	4.91E-15	3.46E-14	23.2352
ENSG00000205177	C11orf91	1.4329	4.99E-15	3.52E-14	23.2183
ENSG00000155755	TMEM237	1.5706	5.03E-15	3.54E-14	23.2118
ENSG00000105204	DYRK1B	1.4103	5.06E-15	3.56E-14	23.2061
ENSG00000139211	AMIGO2	0.5282	5.08E-15	3.57E-14	23.2020
ENSG00000179195	ZNF664	1.3682	5.09E-15	3.58E-14	23.2002
ENSG00000122705	CLTA	0.7201	5.15E-15	3.62E-14	23.1876
ENSG00000139350	NEDD1	1.3848	5.27E-15	3.71E-14	23.1644
ENSG00000099800	TIMM13	0.6763	5.32E-15	3.74E-14	23.1552
ENSG00000106245	BUD31	1.2528	5.35E-15	3.75E-14	23.1511
ENSG00000167680	SEMA6B	1.3354	5.35E-15	3.76E-14	23.1497
ENSG00000100417	PMM1	1.3771	5.37E-15	3.77E-14	23.1462
ENSG00000182230	FAM153B	1.3957	5.40E-15	3.79E-14	23.1407
ENSG00000025796	SEC63	1.4052	5.41E-15	3.79E-14	23.1391
ENSG00000100162	CENPM	1.6671	5.52E-15	3.87E-14	23.1202
ENSG00000104671	DCTN6	1.3279	5.68E-15	3.98E-14	23.0913
ENSG00000163848	ZNF148	1.3181	5.75E-15	4.03E-14	23.0799
ENSG00000140632	GLYR1	1.3486	5.78E-15	4.05E-14	23.0744
ENSG00000099624	ATP5F1D	0.7233	5.87E-15	4.11E-14	23.0582
ENSG00000078142	PIK3C3	1.3707	6.05E-15	4.24E-14	23.0286
ENSG00000130227	XPO7	1.3834	6.09E-15	4.26E-14	23.0233
ENSG00000197323	TRIM33	1.3522	6.13E-15	4.28E-14	23.0169
ENSG00000141577	CEP131	1.3947	6.19E-15	4.33E-14	23.0064
ENSG00000177302	TOP3A	1.3491	6.24E-15	4.36E-14	22.9984
ENSG00000249860	MTRNR2L5	1.1106	6.30E-15	4.40E-14	22.9895

Gene ID	Gene Symbol	FC	P.Value	adj.P.Val	B
ENSG00000091947	TMEM101	0.7304	6.32E-15	4.41E-14	22.9867
ENSG00000143195	ILDR2	1.3573	6.40E-15	4.46E-14	22.9744
ENSG00000258315	C17orf49	1.5235	6.48E-15	4.52E-14	22.9624
ENSG00000142973	CYP4B1	1.1717	6.58E-15	4.59E-14	22.9467
ENSG00000167720	SRR	1.3519	6.65E-15	4.64E-14	22.9358
ENSG00000116830	TTF2	1.4561	6.66E-15	4.64E-14	22.9345
ENSG00000266173	STRADA	1.3280	6.66E-15	4.64E-14	22.9345
ENSG00000119778	ATAD2B	1.3614	6.70E-15	4.67E-14	22.9289
ENSG00000205078	SYCE1L	1.8659	6.71E-15	4.67E-14	22.9272
ENSG00000130985	UBA1	1.3445	6.75E-15	4.70E-14	22.9217
ENSG00000134802	SLC43A3	1.7343	6.95E-15	4.84E-14	22.8923
ENSG00000137648	TMPRSS4	1.1274	7.00E-15	4.87E-14	22.8852
ENSG00000115556	PLCD4	1.3546	7.09E-15	4.93E-14	22.8736
ENSG00000065328	MCM10	1.5231	7.18E-15	4.99E-14	22.8611
ENSG00000166147	FBN1	1.8557	7.18E-15	4.99E-14	22.8602
ENSG00000138780	GSTCD	1.3447	7.23E-15	5.02E-14	22.8535
ENSG00000068438	FTSJ1	1.3242	7.33E-15	5.09E-14	22.8405
ENSG00000111732	AICDA	1.0681	7.40E-15	5.13E-14	22.8308
ENSG00000131746	TNS4	1.1575	7.45E-15	5.17E-14	22.8244
ENSG00000203879	GDI1	1.4580	7.59E-15	5.26E-14	22.8057
ENSG00000159263	SIM2	1.1716	7.70E-15	5.33E-14	22.7925
ENSG00000166189	HPS6	1.4202	7.69E-15	5.33E-14	22.7927
ENSG00000204764	RANBP17	1.4290	7.71E-15	5.34E-14	22.7913
ENSG00000143889	HNRNPLL	1.2958	7.72E-15	5.34E-14	22.7896
ENSG00000146143	PRIM2	1.3524	7.78E-15	5.38E-14	22.7815
ENSG00000111641	NOP2	1.4788	7.83E-15	5.42E-14	22.7750
ENSG00000159348	CYB5R1	1.3886	7.88E-15	5.45E-14	22.7691
ENSG00000105426	PTPRS	2.2413	7.99E-15	5.52E-14	22.7557
ENSG00000160685	ZBTB7B	1.4290	7.99E-15	5.52E-14	22.7554
ENSG00000145365	TIFA	1.4972	8.04E-15	5.55E-14	22.7494
ENSG00000184786	DYNLT2	1.2667	8.11E-15	5.60E-14	22.7410
ENSG00000139116	KIF21A	1.4365	8.12E-15	5.61E-14	22.7396
ENSG00000197928	ZNF677	1.3190	8.24E-15	5.68E-14	22.7255
ENSG00000165105	RASEF	1.4530	8.55E-15	5.89E-14	22.6893
ENSG00000176953	NFATC2IP	1.4508	8.57E-15	5.91E-14	22.6870
ENSG00000175514	GPR152	1.1578	8.69E-15	5.99E-14	22.6732
ENSG00000091157	WDR7	1.3967	8.79E-15	6.06E-14	22.6612
ENSG00000163904	SENP2	1.3408	8.80E-15	6.06E-14	22.6607
ENSG00000162728	KCNJ9	1.0644	8.84E-15	6.09E-14	22.6557
ENSG00000085788	DDHD2	1.4300	8.85E-15	6.09E-14	22.6550
ENSG00000169567	HINT1	0.7587	8.95E-15	6.15E-14	22.6443
ENSG00000170542	SERPINB9	2.1721	8.99E-15	6.18E-14	22.6399
ENSG00000101901	ALG13	1.3318	9.09E-15	6.25E-14	22.6286
ENSG00000215784	FAM72D	1.5571	9.10E-15	6.25E-14	22.6275
ENSG00000107105	ELAVL2	1.2288	9.27E-15	6.37E-14	22.6090
ENSG00000116251	RPL22	0.7867	9.34E-15	6.41E-14	22.6024
ENSG00000137806	NDUFAF1	0.5791	9.35E-15	6.42E-14	22.6006
ENSG00000146757	ZNF92	1.5197	9.41E-15	6.46E-14	22.5945
ENSG00000008710	PKD1	1.4546	9.46E-15	6.49E-14	22.5891
ENSG00000166348	USP54	1.4542	9.53E-15	6.53E-14	22.5825
ENSG00000172922	RNASEH2C	0.7175	9.60E-15	6.58E-14	22.5755
ENSG00000108344	PSMD3	1.3299	9.63E-15	6.60E-14	22.5716
ENSG00000206538	VGLL3	1.1596	9.66E-15	6.62E-14	22.5687
ENSG00000142676	RPL11	0.7562	9.75E-15	6.68E-14	22.5596

Gene ID	Gene Symbol	FC	P.Value	adj.P.Val	B
ENSG00000196584	XRCC2	1.4580	9.76E-15	6.68E-14	22.5591
ENSG00000119707	RBM25	1.4759	9.82E-15	6.72E-14	22.5528
ENSG00000111725	PRKAB1	1.3242	9.96E-15	6.81E-14	22.5385
ENSG00000213741	RPS29	0.7172	1.01E-14	6.90E-14	22.5251
ENSG00000102265	TIMP1	3.7866	1.01E-14	6.91E-14	22.5245
ENSG00000188984	AADACL3	1.0450	1.03E-14	7.01E-14	22.5088
ENSG00000116525	TRIM62	1.3758	1.03E-14	7.07E-14	22.5014
ENSG00000118557	PMFBP1	1.3906	1.06E-14	7.22E-14	22.4799
ENSG00000136731	UGGT1	1.4209	1.06E-14	7.26E-14	22.4746
ENSG00000172748	ZNF596	1.4300	1.07E-14	7.33E-14	22.4640
ENSG00000138764	CCNG2	1.7468	1.08E-14	7.37E-14	22.4588
ENSG00000093183	SEC22C	1.3034	1.08E-14	7.39E-14	22.4549
ENSG00000150991	UBC	1.4839	1.09E-14	7.44E-14	22.4486
ENSG00000166848	TERF2IP	1.4212	1.10E-14	7.48E-14	22.4429
ENSG00000180900	SCRIB	1.4151	1.10E-14	7.50E-14	22.4397
ENSG00000126777	KTN1	1.3484	1.10E-14	7.52E-14	22.4374
ENSG00000170525	PFKFB3	2.0108	1.11E-14	7.52E-14	22.4366
ENSG00000196453	ZNF777	1.3449	1.11E-14	7.58E-14	22.4283
ENSG00000106366	SERPINE1	1.4012	1.12E-14	7.59E-14	22.4265
ENSG00000147003	CLTRN	1.1828	1.12E-14	7.61E-14	22.4237
ENSG00000232196	MTRNR2L4	1.1425	1.13E-14	7.70E-14	22.4117
ENSG00000214562	NUTM2D	1.2852	1.15E-14	7.83E-14	22.3951
ENSG00000125450	NUP85	1.3567	1.15E-14	7.83E-14	22.3941
ENSG00000112972	HMGCS1	1.3293	1.16E-14	7.90E-14	22.3856
ENSG00000130167	TSPAN16	1.1603	1.19E-14	8.10E-14	22.3609
ENSG00000006283	CACNA1G	1.0805	1.19E-14	8.10E-14	22.3599
ENSG00000097021	ACOT7	1.5144	1.20E-14	8.10E-14	22.3594
ENSG00000168026	TTC21A	1.5981	1.20E-14	8.10E-14	22.3593
ENSG00000080986	NDC80	1.6631	1.24E-14	8.40E-14	22.3228
ENSG00000184897	H1-10	1.5615	1.25E-14	8.43E-14	22.3191
ENSG00000156876	SASS6	1.3509	1.25E-14	8.49E-14	22.3116
ENSG00000134779	TPGS2	1.4785	1.26E-14	8.56E-14	22.3039
ENSG00000169814	BTD	0.7100	1.29E-14	8.74E-14	22.2828
ENSG00000119688	ABCD4	1.4241	1.30E-14	8.77E-14	22.2794
ENSG00000133138	TBC1D8B	1.4654	1.31E-14	8.82E-14	22.2729
ENSG00000075785	RAB7A	1.2796	1.31E-14	8.84E-14	22.2707
ENSG00000113658	SMAD5	1.3724	1.32E-14	8.91E-14	22.2625
ENSG00000068489	PRR11	1.5674	1.32E-14	8.92E-14	22.2613
ENSG00000006194	ZNF263	1.2862	1.33E-14	8.96E-14	22.2559
ENSG00000251192	ZNF674	1.2772	1.33E-14	8.96E-14	22.2560
ENSG00000157514	TSC22D3	1.7787	1.33E-14	8.98E-14	22.2537
ENSG00000100479	POLE2	1.6347	1.35E-14	9.13E-14	22.2372
ENSG00000176890	TYMS	1.9286	1.36E-14	9.18E-14	22.2310
ENSG00000204345	CD300LD	1.1231	1.37E-14	9.20E-14	22.2287
ENSG00000147174	GCNA	1.4687	1.37E-14	9.26E-14	22.2223
ENSG00000104497	SNX16	1.4999	1.38E-14	9.28E-14	22.2198
ENSG00000204427	ABHD16A	1.3243	1.39E-14	9.33E-14	22.2134
ENSG00000183760	ACP7	1.1187	1.39E-14	9.34E-14	22.2123
ENSG00000144445	KANSL1L	1.4435	1.39E-14	9.38E-14	22.2079
ENSG00000176928	GCNT4	0.6133	1.40E-14	9.40E-14	22.2057
ENSG00000137842	TMEM62	1.3587	1.40E-14	9.44E-14	22.2009
ENSG00000132915	PDE6A	1.1057	1.41E-14	9.47E-14	22.1972
ENSG00000178952	TUFM	1.2819	1.47E-14	9.84E-14	22.1591
ENSG00000122068	FYTTD1	1.4171	1.48E-14	9.94E-14	22.1486

Gene ID	Gene Symbol	FC	P.Value	adj.P.Val	B
ENSG00000179271	GADD45GIP1	0.7492	1.50E-14	1.01E-13	22.1342
ENSG00000166535	A2ML1	1.1191	1.51E-14	1.01E-13	22.1320
ENSG00000174514	MFSD4A	1.3024	1.52E-14	1.02E-13	22.1256
ENSG00000150048	CLEC1A	1.2580	1.52E-14	1.02E-13	22.1225
ENSG00000197912	SPG7	1.4874	1.53E-14	1.02E-13	22.1174
ENSG00000126216	TUBGCP3	1.3531	1.57E-14	1.05E-13	22.0927
ENSG00000114439	BBX	1.4435	1.61E-14	1.08E-13	22.0672
ENSG00000137962	ARHGAP29	1.3303	1.65E-14	1.10E-13	22.0444
ENSG00000096070	BRPF3	1.3574	1.65E-14	1.11E-13	22.0412
ENSG00000136451	VEZF1	1.4226	1.67E-14	1.11E-13	22.0328
ENSG00000099860	GADD45B	2.1533	1.67E-14	1.12E-13	22.0318
ENSG00000162571	TTLL10	1.2459	1.69E-14	1.13E-13	22.0211
ENSG00000198934	MAGEE1	1.3695	1.70E-14	1.14E-13	22.0102
ENSG00000169876	MUC17	1.1685	1.71E-14	1.14E-13	22.0057
ENSG00000204435	CSNK2B	1.2341	1.73E-14	1.15E-13	21.9963
ENSG00000214212	C19orf38	0.6490	1.74E-14	1.16E-13	21.9893
ENSG00000141644	MBD1	1.3794	1.76E-14	1.18E-13	21.9783
ENSG00000113742	CPEB4	1.6143	1.79E-14	1.19E-13	21.9636
ENSG00000143401	ANP32E	1.5036	1.84E-14	1.23E-13	21.9329
ENSG00000138398	PPIG	1.3276	1.86E-14	1.24E-13	21.9270
ENSG00000164576	SAP30L	1.3615	1.88E-14	1.25E-13	21.9151
ENSG00000101940	WDR13	1.3142	1.89E-14	1.26E-13	21.9078
ENSG00000085449	WDFY1	1.5022	1.90E-14	1.26E-13	21.9048
ENSG00000176894	PXMP2	1.4604	1.94E-14	1.29E-13	21.8855
ENSG00000203705	TATDN3	1.4698	1.96E-14	1.31E-13	21.8713
ENSG00000177888	ZBTB41	1.3813	2.00E-14	1.33E-13	21.8544
ENSG00000132780	NASP	1.4765	2.04E-14	1.35E-13	21.8350
ENSG00000173218	VANGL1	1.6205	2.04E-14	1.36E-13	21.8322
ENSG00000137801	THBS1	2.0952	2.05E-14	1.36E-13	21.8301
ENSG00000000460	C1orf112	1.5433	2.05E-14	1.36E-13	21.8270
ENSG00000198521	ZNF43	1.4375	2.06E-14	1.37E-13	21.8245
ENSG00000129680	MAP7D3	1.4007	2.12E-14	1.41E-13	21.7970
ENSG00000092841	MYL6	1.3349	2.22E-14	1.47E-13	21.7524
ENSG00000172809	RPL38	0.7747	2.24E-14	1.49E-13	21.7416
ENSG00000152582	SPEF2	0.5657	2.26E-14	1.50E-13	21.7349
ENSG00000072778	ACADVL	1.6428	2.29E-14	1.52E-13	21.7207
ENSG00000067191	CACNB1	1.5616	2.29E-14	1.52E-13	21.7189
ENSG00000204694	OR11A1	1.1041	2.30E-14	1.52E-13	21.7150
ENSG00000160695	VPS11	1.2794	2.31E-14	1.53E-13	21.7098
ENSG00000115966	ATF2	1.3062	2.32E-14	1.53E-13	21.7090
ENSG00000181652	ATG9B	1.3682	2.38E-14	1.57E-13	21.6820
ENSG00000138698	RAP1GDS1	1.3687	2.39E-14	1.58E-13	21.6775
ENSG00000079246	XRCC5	1.2966	2.42E-14	1.60E-13	21.6645
ENSG00000157216	SSBP3	1.4813	2.46E-14	1.63E-13	21.6500
ENSG00000108219	TSPAN14	1.6424	2.48E-14	1.64E-13	21.6397
ENSG00000186908	ZDHHC17	1.4336	2.52E-14	1.67E-13	21.6253
ENSG00000119844	AFTPH	1.3114	2.57E-14	1.70E-13	21.6057
ENSG00000057294	PKP2	2.6620	2.57E-14	1.70E-13	21.6049
ENSG00000197223	C1D	1.2956	2.57E-14	1.70E-13	21.6052
ENSG00000171649	ZIK1	1.6699	2.58E-14	1.70E-13	21.6033
ENSG00000177463	NR2C2	1.3975	2.60E-14	1.71E-13	21.5959
ENSG00000183648	NDUFB1	0.7490	2.61E-14	1.72E-13	21.5904
ENSG00000225190	PLEKHM1	1.3380	2.67E-14	1.76E-13	21.5684
ENSG00000188783	PRELP	1.0854	2.68E-14	1.76E-13	21.5671

Gene ID	Gene Symbol	FC	P.Value	adj.P.Val	B
ENSG00000164609	SLU7	1.3073	2.69E-14	1.77E-13	21.5601
ENSG00000139990	DCAF5	1.3389	2.72E-14	1.79E-13	21.5498
ENSG00000188981	MSANTD1	1.1232	2.72E-14	1.79E-13	21.5491
ENSG00000137955	RABGGTB	1.3000	2.73E-14	1.80E-13	21.5467
ENSG00000196263	ZNF471	1.3577	2.74E-14	1.80E-13	21.5449
ENSG00000136710	CCDC115	1.3470	2.74E-14	1.80E-13	21.5432
ENSG00000213923	CSNK1E	1.4971	2.74E-14	1.80E-13	21.5428
ENSG00000213762	ZNF134	1.4407	2.77E-14	1.82E-13	21.5337
ENSG00000105650	PDE4C	1.2339	2.78E-14	1.82E-13	21.5304
ENSG00000143207	COP1	1.2873	2.79E-14	1.83E-13	21.5251
ENSG00000153162	BMP6	0.5929	2.84E-14	1.86E-13	21.5085
ENSG00000166987	MBD6	1.4322	2.86E-14	1.88E-13	21.4999
ENSG00000035681	NSMAF	1.8510	2.88E-14	1.89E-13	21.4946
ENSG00000104611	SH2D4A	1.6730	2.88E-14	1.89E-13	21.4934
ENSG00000103111	MON1B	1.4642	2.89E-14	1.89E-13	21.4906
ENSG00000004961	HCCS	1.2858	3.04E-14	1.99E-13	21.4405
ENSG00000101868	POLA1	1.3964	3.08E-14	2.02E-13	21.4276
ENSG00000196150	ZNF250	1.3534	3.09E-14	2.02E-13	21.4269
ENSG00000176542	USF3	1.3004	3.12E-14	2.04E-13	21.4160
ENSG00000167552	TUBA1A	2.2647	3.23E-14	2.11E-13	21.3826
ENSG00000257704	INAFM1	1.4490	3.23E-14	2.11E-13	21.3813
ENSG00000173114	LRRN3	1.0736	3.27E-14	2.14E-13	21.3705
ENSG00000092140	G2E3	1.3389	3.30E-14	2.16E-13	21.3609
ENSG00000119231	SENP5	1.3114	3.31E-14	2.16E-13	21.3568
ENSG00000064932	SBNO2	1.6157	3.33E-14	2.17E-13	21.3517
ENSG00000162510	MATN1	1.4510	3.35E-14	2.19E-13	21.3460
ENSG00000197020	ZNF100	1.4558	3.35E-14	2.19E-13	21.3447
ENSG00000083812	ZNF324	1.3043	3.38E-14	2.20E-13	21.3379
ENSG00000158315	RHBDL2	1.0816	3.47E-14	2.26E-13	21.3119
ENSG00000134825	TMEM258	0.7432	3.48E-14	2.27E-13	21.3080
ENSG00000159164	SV2A	1.2877	3.48E-14	2.27E-13	21.3078
ENSG00000173598	NUDT4	1.3029	3.48E-14	2.27E-13	21.3073
ENSG00000151914	DST	1.8110	3.50E-14	2.28E-13	21.3018
ENSG00000149196	HIKESHI	0.7019	3.51E-14	2.28E-13	21.3010
ENSG00000137270	GCM1	1.1521	3.53E-14	2.30E-13	21.2952
ENSG00000185278	ZBTB37	1.3599	3.55E-14	2.31E-13	21.2900
ENSG00000142408	CACNG8	1.0880	3.57E-14	2.32E-13	21.2841
ENSG00000110955	ATP5F1B	1.2157	3.57E-14	2.32E-13	21.2821
ENSG00000186862	PDZD7	1.2394	3.65E-14	2.37E-13	21.2624
ENSG00000122884	P4HA1	1.6898	3.66E-14	2.38E-13	21.2580
ENSG00000143653	SCCPDH	2.1351	3.69E-14	2.39E-13	21.2518
ENSG00000183378	OVCH2	1.2005	3.70E-14	2.40E-13	21.2481
ENSG00000077348	EXOSC5	0.6169	3.77E-14	2.45E-13	21.2295
ENSG00000184381	PLA2G6	1.6786	3.80E-14	2.47E-13	21.2210
ENSG00000108518	PFN1	1.4074	3.83E-14	2.48E-13	21.2152
ENSG00000089057	SLC23A2	1.4198	3.89E-14	2.52E-13	21.2001
ENSG00000160285	LSS	1.5164	3.89E-14	2.52E-13	21.1990
ENSG00000151164	RAD9B	1.2830	3.96E-14	2.57E-13	21.1810
ENSG00000126550	HTN1	1.1995	4.00E-14	2.59E-13	21.1718
ENSG00000140455	USP3	1.5085	4.08E-14	2.64E-13	21.1522
ENSG00000131914	LIN28A	1.0679	4.09E-14	2.65E-13	21.1489
ENSG00000168569	TMEM223	0.6742	4.15E-14	2.68E-13	21.1364
ENSG00000183426	NPIPA1	1.4012	4.15E-14	2.68E-13	21.1362
ENSG00000116761	CTH	1.5744	4.15E-14	2.68E-13	21.1351

Gene ID	Gene Symbol	FC	P.Value	adj.P.Val	B
ENSG00000093000	NUP50	1.3410	4.17E-14	2.69E-13	21.1313
ENSG00000204406	MBD5	1.2832	4.18E-14	2.70E-13	21.1288
ENSG00000177628	GBA	1.5821	4.21E-14	2.72E-13	21.1223
ENSG00000189120	SP6	1.0991	4.28E-14	2.77E-13	21.1043
ENSG00000077097	TOP2B	1.3461	4.29E-14	2.77E-13	21.1020
ENSG00000173230	GOLGB1	1.3702	4.36E-14	2.81E-13	21.0865
ENSG00000153317	ASAP1	1.3853	4.37E-14	2.82E-13	21.0843
ENSG00000128908	INO80	1.2947	4.38E-14	2.82E-13	21.0822
ENSG00000115165	CYTIP	1.5747	4.38E-14	2.83E-13	21.0816
ENSG00000164045	CDC25A	1.4724	4.43E-14	2.86E-13	21.0705
ENSG00000170989	S1PR1	2.0323	4.50E-14	2.90E-13	21.0555
ENSG00000203499	IQANK1	1.1681	4.51E-14	2.90E-13	21.0538
ENSG00000160087	UBE2J2	1.2718	4.57E-14	2.94E-13	21.0408
ENSG00000185955	C7orf61	1.1940	4.58E-14	2.94E-13	21.0395
ENSG00000167130	DOLPP1	0.6467	4.60E-14	2.96E-13	21.0349
ENSG00000258555	SPECC1L-ADORA2A	1.1265	4.63E-14	2.97E-13	21.0286
ENSG00000171931	FBXW10	1.2136	4.68E-14	3.01E-13	21.0172
ENSG00000089053	ANAPC5	1.2840	4.72E-14	3.04E-13	21.0081
ENSG00000175470	PPP2R2D	1.2686	4.74E-14	3.04E-13	21.0049
ENSG00000174951	FUT1	1.2511	4.82E-14	3.09E-13	20.9892
ENSG00000187753	C9orf153	1.2359	4.88E-14	3.13E-13	20.9767
ENSG00000095319	NUP188	1.3840	4.91E-14	3.15E-13	20.9696
ENSG00000106665	CLIP2	1.4048	4.98E-14	3.19E-13	20.9561
ENSG00000082258	CCNT2	1.3762	5.03E-14	3.23E-13	20.9463
ENSG00000198964	SGMS1	1.3141	5.04E-14	3.23E-13	20.9454
ENSG00000129167	TPH1	1.1794	5.05E-14	3.24E-13	20.9418
ENSG00000187980	PLA2G2C	1.0616	5.09E-14	3.26E-13	20.9343
ENSG00000117461	PIK3R3	0.6029	5.10E-14	3.26E-13	20.9335
ENSG00000164035	EMCN	1.2796	5.21E-14	3.33E-13	20.9127
ENSG00000047230	CTPS2	1.4172	5.30E-14	3.39E-13	20.8949
ENSG00000169607	CKAP2L	1.5320	5.37E-14	3.44E-13	20.8821
ENSG00000158816	VWA5B1	1.0517	5.37E-14	3.44E-13	20.8816
ENSG00000108587	GOSR1	1.3313	5.41E-14	3.46E-13	20.8748
ENSG00000112062	MAPK14	1.3517	5.50E-14	3.52E-13	20.8582
ENSG00000141002	TCF25	1.4099	5.51E-14	3.52E-13	20.8566
ENSG00000111670	GNPTAB	1.4162	5.65E-14	3.61E-13	20.8318
ENSG00000171161	ZNF672	1.3857	5.70E-14	3.64E-13	20.8243
ENSG00000152291	TGOLN2	1.3260	5.72E-14	3.65E-13	20.8209
ENSG00000127914	AKAP9	1.4370	5.72E-14	3.65E-13	20.8194
ENSG00000253719	ATXN7L3B	1.3285	5.72E-14	3.65E-13	20.8194
ENSG00000139438	FAM222A	1.3849	5.76E-14	3.67E-13	20.8139
ENSG00000265681	RPL17	0.7254	5.79E-14	3.69E-13	20.8074
ENSG00000168228	ZCCHC4	1.2900	5.80E-14	3.69E-13	20.8065
ENSG00000163704	PRRT3	1.2858	6.05E-14	3.85E-13	20.7655
ENSG00000256188	TAS2R30	1.1116	6.08E-14	3.87E-13	20.7595
ENSG00000139372	TDG	1.3169	6.14E-14	3.91E-13	20.7498
ENSG00000118194	TNNT2	1.0878	6.22E-14	3.96E-13	20.7373
ENSG00000135776	ABCB10	1.3812	6.23E-14	3.96E-13	20.7361
ENSG00000174990	CA5A	1.4003	6.27E-14	3.99E-13	20.7291
ENSG00000015285	WAS	1.3496	6.43E-14	4.08E-13	20.7057
ENSG00000132475	H3-3B	1.5841	6.47E-14	4.11E-13	20.6982
ENSG00000099814	CEP170B	1.8666	6.48E-14	4.12E-13	20.6968
ENSG00000075886	TUBA3D	1.3195	6.55E-14	4.16E-13	20.6865
ENSG00000067066	SP100	1.3799	6.56E-14	4.16E-13	20.6861

Gene ID	Gene Symbol	FC	P.Value	adj.P.Val	B
ENSG00000140948	ZCCHC14	1.4570	6.68E-14	4.24E-13	20.6671
ENSG00000197102	DYNC1H1	1.3383	6.69E-14	4.24E-13	20.6661
ENSG00000185619	PCGF3	1.3703	6.70E-14	4.25E-13	20.6650
ENSG00000123136	DDX39A	1.3905	6.75E-14	4.28E-13	20.6576
ENSG00000006459	KDM7A	1.5364	6.80E-14	4.31E-13	20.6495
ENSG00000168477	TNXB	1.4251	6.84E-14	4.33E-13	20.6445
ENSG00000172986	GXYLT2	1.0533	6.97E-14	4.41E-13	20.6261
ENSG00000159556	ISL2	0.3901	7.05E-14	4.46E-13	20.6147
ENSG00000167967	E4F1	1.3147	7.19E-14	4.55E-13	20.5955
ENSG00000168612	ZSWIM1	1.3049	7.54E-14	4.77E-13	20.5486
ENSG00000121653	MAPK8IP1	1.5204	7.66E-14	4.85E-13	20.5326
ENSG00000173327	MAP3K11	1.3279	7.68E-14	4.86E-13	20.5300
ENSG00000170522	ELOVL6	2.2759	7.89E-14	4.99E-13	20.5039
ENSG00000161958	FGF11	1.3224	7.90E-14	4.99E-13	20.5031
ENSG00000138629	UBL7	0.7195	8.14E-14	5.14E-13	20.4735
ENSG00000026559	KCNG1	1.1085	8.14E-14	5.14E-13	20.4729
ENSG00000111605	CPSF6	1.3126	8.21E-14	5.18E-13	20.4646
ENSG00000173542	MOB1B	1.5831	8.27E-14	5.22E-13	20.4575
ENSG00000065361	ERBB3	1.2975	8.34E-14	5.26E-13	20.4497
ENSG00000146066	HIGD2A	0.7324	8.34E-14	5.26E-13	20.4500
ENSG00000076003	MCM6	1.5675	8.59E-14	5.42E-13	20.4199
ENSG00000174574	AKIRIN1	1.3101	8.61E-14	5.42E-13	20.4181
ENSG00000150779	TIMM8B	0.6182	8.63E-14	5.44E-13	20.4156
ENSG00000157330	C1orf158	1.0946	8.66E-14	5.45E-13	20.4123
ENSG00000023839	ABCC2	1.3041	8.68E-14	5.46E-13	20.4106
ENSG00000111077	TNS2	1.4455	8.71E-14	5.48E-13	20.4067
ENSG00000169032	MAP2K1	1.3430	8.74E-14	5.50E-13	20.4031
ENSG00000183617	MRPL54	0.7673	8.79E-14	5.52E-13	20.3983
ENSG00000198814	GK	1.4137	8.80E-14	5.53E-13	20.3972
ENSG00000241186	TDGF1	1.0795	8.95E-14	5.62E-13	20.3804
ENSG00000100209	HSCB	0.7601	8.97E-14	5.63E-13	20.3782
ENSG00000155256	ZFYVE27	1.2964	9.10E-14	5.72E-13	20.3635
ENSG00000186642	PDE2A	1.4916	9.12E-14	5.72E-13	20.3617
ENSG00000151414	NEK7	1.4655	9.21E-14	5.78E-13	20.3523
ENSG00000124784	RIOK1	1.3563	9.32E-14	5.85E-13	20.3402
ENSG00000174485	DENND4A	1.5600	9.34E-14	5.86E-13	20.3382
ENSG00000173517	PEAK1	1.5435	9.39E-14	5.89E-13	20.3324
ENSG00000261739	GOLGA8S	0.4565	9.44E-14	5.92E-13	20.3275
ENSG00000170417	TMEM182	1.4216	9.57E-14	5.99E-13	20.3147
ENSG00000188486	H2AX	1.5273	9.74E-14	6.10E-13	20.2968
ENSG00000136546	SCN7A	1.1503	9.75E-14	6.11E-13	20.2954
ENSG00000149657	LSM14B	1.3812	9.80E-14	6.13E-13	20.2909
ENSG00000205649	HTN3	1.2943	9.86E-14	6.17E-13	20.2851
ENSG00000240720	LRRD1	1.0816	9.87E-14	6.17E-13	20.2839
ENSG00000186283	TOR3A	1.6096	9.90E-14	6.19E-13	20.2811
ENSG00000006744	ELAC2	1.3478	9.92E-14	6.20E-13	20.2789
ENSG00000130749	ZC3H4	1.3309	9.93E-14	6.21E-13	20.2778
ENSG00000214226	C17orf67	1.4422	9.97E-14	6.23E-13	20.2743
ENSG00000108559	NUP88	1.3024	9.99E-14	6.24E-13	20.2723
ENSG00000166451	CENPN	1.4686	1.02E-13	6.34E-13	20.2554
ENSG00000196660	SLC30A10	1.0454	1.02E-13	6.36E-13	20.2528
ENSG00000067445	TRO	1.8384	1.02E-13	6.39E-13	20.2469
ENSG00000141682	PMAIP1	3.6228	1.03E-13	6.43E-13	20.2410
ENSG00000171791	BCL2	1.6183	1.04E-13	6.46E-13	20.2364

Gene ID	Gene Symbol	FC	P.Value	adj.P.Val	B
ENSG00000127603	MACF1	1.3821	1.05E-13	6.51E-13	20.2275
ENSG00000131242	RAB11FIP4	1.3986	1.05E-13	6.52E-13	20.2259
ENSG00000146963	LUC7L2	1.4075	1.06E-13	6.60E-13	20.2139
ENSG00000111727	HCFC2	1.4992	1.06E-13	6.61E-13	20.2119
ENSG00000154832	CXXC1	1.4185	1.06E-13	6.61E-13	20.2120
ENSG00000132563	REEP2	1.3416	1.06E-13	6.61E-13	20.2109
ENSG00000147437	GNRH1	1.2704	1.07E-13	6.64E-13	20.2067
ENSG00000022267	FHL1	3.1742	1.08E-13	6.68E-13	20.2000
ENSG00000166710	B2M	0.6763	1.09E-13	6.76E-13	20.1891
ENSG00000088356	PDRG1	1.3189	1.09E-13	6.80E-13	20.1824
ENSG00000176204	LRRTM4	1.3433	1.10E-13	6.84E-13	20.1762
ENSG00000118620	ZNF430	1.3369	1.10E-13	6.85E-13	20.1749
ENSG00000120519	SLC10A7	1.4038	1.16E-13	7.18E-13	20.1285
ENSG00000090686	USP48	1.4199	1.16E-13	7.18E-13	20.1271
ENSG00000177728	TMEM94	1.3944	1.16E-13	7.21E-13	20.1235
ENSG00000163376	KBTBD8	1.6222	1.17E-13	7.23E-13	20.1207
ENSG00000048342	CC2D2A	1.2785	1.17E-13	7.26E-13	20.1159
ENSG00000166888	STAT6	1.4783	1.18E-13	7.29E-13	20.1118
ENSG00000261221	ZNF865	1.2907	1.21E-13	7.48E-13	20.0856
ENSG00000085552	IGSF9	1.2424	1.23E-13	7.62E-13	20.0669
ENSG00000110911	SLC11A2	1.3181	1.25E-13	7.73E-13	20.0531
ENSG00000095380	NANS	0.7337	1.29E-13	7.96E-13	20.0236
ENSG00000167136	ENDOG	0.6587	1.29E-13	7.96E-13	20.0230
ENSG00000162490	DRAXIN	1.0439	1.30E-13	8.01E-13	20.0170
ENSG00000144645	OSBPL10	0.4857	1.32E-13	8.17E-13	19.9970
ENSG00000179059	ZFP42	1.0748	1.33E-13	8.23E-13	19.9892
ENSG00000164023	SGMS2	1.3017	1.35E-13	8.32E-13	19.9792
ENSG00000187790	FANCM	1.3582	1.35E-13	8.35E-13	19.9748
ENSG00000127564	PKMYT1	1.7959	1.37E-13	8.43E-13	19.9647
ENSG00000072501	SMC1A	1.5812	1.37E-13	8.46E-13	19.9616
ENSG00000188674	C2orf80	1.1267	1.38E-13	8.52E-13	19.9541
ENSG00000181826	RELL1	1.5659	1.40E-13	8.63E-13	19.9413
ENSG00000126653	NSRP1	1.2389	1.41E-13	8.69E-13	19.9342
ENSG00000089775	ZBTB25	1.3562	1.44E-13	8.87E-13	19.9130
ENSG00000146830	GIGYF1	1.3584	1.44E-13	8.87E-13	19.9131
ENSG00000183520	UTP11	1.2753	1.45E-13	8.92E-13	19.9069
ENSG00000108963	DPH1	1.3600	1.47E-13	9.05E-13	19.8933
ENSG00000163762	TM4SF18	1.1502	1.48E-13	9.08E-13	19.8890
ENSG00000084693	AGBL5	1.3310	1.51E-13	9.32E-13	19.8630
ENSG00000148572	NRBF2	1.3251	1.52E-13	9.35E-13	19.8596
ENSG00000103494	RPGRIP1L	1.2861	1.54E-13	9.46E-13	19.8484
ENSG00000084090	STARD7	1.2779	1.55E-13	9.50E-13	19.8437
ENSG00000267534	S1PR2	1.5815	1.57E-13	9.63E-13	19.8303
ENSG00000154429	CCSAP	1.8066	1.57E-13	9.66E-13	19.8266
ENSG00000133962	CATSPERB	1.1820	1.60E-13	9.84E-13	19.8084
ENSG00000005379	TSPOAP1	1.8594	1.62E-13	9.93E-13	19.7990
ENSG00000138600	SPPL2A	1.3349	1.62E-13	9.95E-13	19.7962
ENSG00000128408	RIBC2	1.4169	1.64E-13	1.01E-12	19.7859
ENSG00000135740	SLC9A5	1.2637	1.64E-13	1.01E-12	19.7860
ENSG00000146809	ASB15	1.0715	1.65E-13	1.01E-12	19.7803
ENSG00000157191	NECAP2	1.4098	1.66E-13	1.02E-12	19.7716
ENSG00000131023	LATS1	1.3428	1.67E-13	1.02E-12	19.7698
ENSG00000128585	MKLN1	1.3357	1.69E-13	1.04E-12	19.7545
ENSG00000115020	PIKFYVE	1.3912	1.74E-13	1.06E-12	19.7292

Gene ID	Gene Symbol	FC	P.Value	adj.P.Val	B
ENSG00000111247	RAD51AP1	1.6322	1.75E-13	1.07E-12	19.7234
ENSG00000152270	PDE3B	1.6133	1.75E-13	1.07E-12	19.7232
ENSG00000078795	PKD2L2	1.2281	1.80E-13	1.10E-12	19.6951
ENSG00000166862	CACNG2	1.0833	1.82E-13	1.11E-12	19.6849
ENSG00000061273	HDAC7	1.4115	1.84E-13	1.12E-12	19.6746
ENSG00000096093	EFHC1	1.5377	1.86E-13	1.13E-12	19.6638
ENSG00000188483	IER5L	1.5710	1.87E-13	1.14E-12	19.6560
ENSG00000179832	MROH1	1.3956	1.88E-13	1.15E-12	19.6494
ENSG00000186174	BCL9L	1.5566	1.90E-13	1.16E-12	19.6403
ENSG00000149231	CCDC82	1.4470	1.91E-13	1.16E-12	19.6377
ENSG00000107862	GBF1	1.3859	1.93E-13	1.18E-12	19.6240
ENSG00000156535	CD109	1.5156	1.94E-13	1.18E-12	19.6225
ENSG00000136002	ARHGEF4	1.1111	1.94E-13	1.18E-12	19.6203
ENSG00000141519	CCDC40	1.3019	1.96E-13	1.19E-12	19.6099
ENSG00000082515	MRPL22	1.3088	1.96E-13	1.20E-12	19.6085
ENSG00000168872	DDX19A	1.3229	1.98E-13	1.21E-12	19.5984
ENSG00000163875	MEAF6	1.3238	1.99E-13	1.21E-12	19.5935
ENSG00000054967	RELT	1.7139	2.01E-13	1.22E-12	19.5872
ENSG00000177548	RABEP2	1.4091	2.03E-13	1.23E-12	19.5764
ENSG00000182040	USH1G	1.0297	2.03E-13	1.23E-12	19.5761
ENSG00000055917	PUM2	1.3240	2.06E-13	1.25E-12	19.5630
ENSG00000156508	EEF1A1	0.7406	2.06E-13	1.25E-12	19.5597
ENSG00000148498	PARD3	3.1637	2.08E-13	1.26E-12	19.5532
ENSG00000172869	DMXL1	1.3484	2.15E-13	1.31E-12	19.5198
ENSG00000123485	HJURP	1.7164	2.16E-13	1.31E-12	19.5162
ENSG00000205832	C16orf96	1.0927	2.17E-13	1.32E-12	19.5117
ENSG00000091009	RBM27	1.2636	2.17E-13	1.32E-12	19.5083
ENSG00000158813	EDA	1.4288	2.20E-13	1.34E-12	19.4955
ENSG00000170949	ZNF160	1.6658	2.27E-13	1.38E-12	19.4640
ENSG00000183955	KMT5A	1.4391	2.28E-13	1.38E-12	19.4618
ENSG00000186564	FOXD2	1.1065	2.28E-13	1.38E-12	19.4618
ENSG00000167524	RSKR	1.6025	2.28E-13	1.38E-12	19.4611
ENSG00000205002	AARD	1.1297	2.30E-13	1.40E-12	19.4514
ENSG00000110711	AIP	0.7626	2.31E-13	1.40E-12	19.4467
ENSG00000166974	MAPRE2	1.6844	2.32E-13	1.40E-12	19.4455
ENSG00000140988	RPS2	0.5975	2.34E-13	1.42E-12	19.4364
ENSG00000170819	BFSP2	0.5391	2.34E-13	1.42E-12	19.4343
ENSG00000167325	RRM1	1.4003	2.36E-13	1.43E-12	19.4257
ENSG00000100314	CABP7	1.0902	2.37E-13	1.43E-12	19.4239
ENSG00000065809	FAM107B	2.6082	2.39E-13	1.45E-12	19.4146
ENSG00000113648	MACROH2A1	1.3097	2.43E-13	1.47E-12	19.4006
ENSG00000171877	FRMD5	1.1555	2.45E-13	1.48E-12	19.3912
ENSG00000086015	MAST2	1.3918	2.51E-13	1.52E-12	19.3674
ENSG00000154781	CCDC174	1.3112	2.52E-13	1.52E-12	19.3647
ENSG00000185668	POU3F1	1.0495	2.54E-13	1.53E-12	19.3547
ENSG00000139194	RBP5	1.5644	2.54E-13	1.53E-12	19.3542
ENSG00000126861	OMG	1.4309	2.56E-13	1.54E-12	19.3478
ENSG00000182010	RTKN2	1.2405	2.58E-13	1.56E-12	19.3385
ENSG00000147316	MCPH1	1.3939	2.59E-13	1.56E-12	19.3353
ENSG00000088881	EBF4	1.8073	2.63E-13	1.58E-12	19.3222
ENSG00000184635	ZNF93	1.4046	2.63E-13	1.58E-12	19.3215
ENSG00000132388	UBE2G1	1.3850	2.64E-13	1.59E-12	19.3176
ENSG00000109756	RAPGEF2	1.7882	2.65E-13	1.59E-12	19.3152
ENSG00000089723	OTUB2	1.2651	2.69E-13	1.62E-12	19.2977

Gene ID	Gene Symbol	FC	P.Value	adj.P.Val	B
ENSG00000129173	E2F8	1.6021	2.70E-13	1.63E-12	19.2946
ENSG00000151883	PARP8	1.5256	2.71E-13	1.63E-12	19.2932
ENSG00000135451	TROAP	1.7892	2.73E-13	1.64E-12	19.2833
ENSG00000173421	IHO1	1.0893	2.77E-13	1.66E-12	19.2717
ENSG00000134516	DOCK2	1.3010	2.77E-13	1.66E-12	19.2709
ENSG00000137745	MMP13	1.1676	2.79E-13	1.67E-12	19.2644
ENSG00000152520	PAN3	1.4138	2.79E-13	1.67E-12	19.2647
ENSG00000179010	MRFAP1	1.2965	2.81E-13	1.69E-12	19.2565
ENSG00000102128	RAB40AL	1.1031	2.82E-13	1.69E-12	19.2524
ENSG00000113649	TCERG1	1.3134	2.84E-13	1.70E-12	19.2458
ENSG00000197756	RPL37A	0.7688	2.87E-13	1.72E-12	19.2367
ENSG00000122376	SHLD2	1.3016	2.89E-13	1.73E-12	19.2291
ENSG00000104388	RAB2A	1.2622	2.96E-13	1.78E-12	19.2040
ENSG00000071205	ARHGAP10	1.7893	2.98E-13	1.79E-12	19.1973
ENSG00000164542	KIAA0895	1.5358	3.00E-13	1.79E-12	19.1930
ENSG00000197555	SIPA1L1	1.4584	3.02E-13	1.81E-12	19.1843
ENSG00000154359	LONRF1	1.8327	3.03E-13	1.81E-12	19.1818
ENSG00000172602	RND1	1.9056	3.07E-13	1.84E-12	19.1694
ENSG00000240682	ISY1	1.2357	3.11E-13	1.86E-12	19.1552
ENSG00000175643	RMI2	1.6355	3.15E-13	1.88E-12	19.1440
ENSG00000197312	DDI2	1.3837	3.16E-13	1.89E-12	19.1424
ENSG00000179046	TRIML2	1.2914	3.16E-13	1.89E-12	19.1419
ENSG00000111319	SCNN1A	1.3113	3.17E-13	1.89E-12	19.1389
ENSG00000006007	GDE1	1.2385	3.20E-13	1.91E-12	19.1274
ENSG00000214114	MYCBP	0.7114	3.25E-13	1.94E-12	19.1145
ENSG00000163539	CLASP2	1.3325	3.28E-13	1.96E-12	19.1038
ENSG00000135090	TAOK3	1.3074	3.31E-13	1.97E-12	19.0960
ENSG00000242950	ERVW-1	1.3933	3.37E-13	2.01E-12	19.0779
ENSG00000080546	SESN1	1.6766	3.38E-13	2.01E-12	19.0761
ENSG00000136830	NIBAN2	1.5070	3.37E-13	2.01E-12	19.0764
ENSG00000175826	CTDNEP1	1.2515	3.41E-13	2.03E-12	19.0648
ENSG00000160352	ZNF714	1.3687	3.45E-13	2.05E-12	19.0557
ENSG00000127452	FBXL12	1.3282	3.49E-13	2.08E-12	19.0435
ENSG00000125875	TBC1D20	1.3040	3.51E-13	2.09E-12	19.0377
ENSG00000136485	DCAF7	1.3120	3.58E-13	2.13E-12	19.0182
ENSG00000173334	TRIB1	1.5617	3.58E-13	2.13E-12	19.0178
ENSG00000100100	PIK3IP1	1.9026	3.59E-13	2.14E-12	19.0144
ENSG00000009765	IYD	1.1183	3.64E-13	2.17E-12	19.0010
ENSG00000221968	FADS3	1.7254	3.71E-13	2.21E-12	18.9823
ENSG00000133812	SBF2	1.4790	3.74E-13	2.22E-12	18.9750
ENSG00000101266	CSNK2A1	1.3189	3.75E-13	2.23E-12	18.9726
ENSG00000159208	CIART	1.5234	3.78E-13	2.24E-12	18.9651
ENSG00000169249	ZRSR2	1.3244	3.84E-13	2.28E-12	18.9499
ENSG00000103995	CEP152	1.3412	3.87E-13	2.29E-12	18.9430
ENSG00000101049	SGK2	1.1898	3.88E-13	2.30E-12	18.9390
ENSG00000149548	CCDC15	1.3255	3.90E-13	2.31E-12	18.9340
ENSG00000158423	RIBC1	1.1796	3.90E-13	2.31E-12	18.9341
ENSG00000173212	MAB21L3	1.3806	3.91E-13	2.32E-12	18.9324
ENSG00000185100	ADSS1	1.5987	3.92E-13	2.32E-12	18.9296
ENSG00000138594	TMOD3	1.3125	3.95E-13	2.34E-12	18.9231
ENSG00000116213	WRAP73	1.2966	3.95E-13	2.34E-12	18.9225
ENSG00000115661	STK16	1.2466	3.96E-13	2.34E-12	18.9202
ENSG00000080345	RIF1	1.5509	3.98E-13	2.35E-12	18.9146
ENSG00000154217	PITPNC1	1.8283	4.02E-13	2.38E-12	18.9046

Gene ID	Gene Symbol	FC	P.Value	adj.P.Val	B
ENSG00000171858	RPS21	0.7520	4.02E-13	2.38E-12	18.9036
ENSG00000220205	VAMP2	1.3766	4.03E-13	2.38E-12	18.9020
ENSG00000121152	NCAPH	1.6139	4.06E-13	2.40E-12	18.8959
ENSG00000196588	MRTFA	1.4417	4.06E-13	2.40E-12	18.8955
ENSG00000180251	SLC9A4	1.1336	4.11E-13	2.43E-12	18.8837
ENSG00000035403	VCL	1.4580	4.16E-13	2.46E-12	18.8698
ENSG00000116396	KCNC4	1.3447	4.19E-13	2.47E-12	18.8650
ENSG00000104142	VPS18	1.3183	4.22E-13	2.49E-12	18.8572
ENSG00000164828	SUN1	1.4242	4.22E-13	2.49E-12	18.8562
ENSG00000135926	TMBIM1	1.4350	4.24E-13	2.50E-12	18.8527
ENSG00000101811	CSTF2	1.4160	4.24E-13	2.50E-12	18.8515
ENSG00000135720	DYNC1LI2	1.5226	4.32E-13	2.55E-12	18.8333
ENSG00000244187	TMEM141	0.7082	4.42E-13	2.60E-12	18.8120
ENSG00000176601	MAP3K19	0.5776	4.52E-13	2.66E-12	18.7901
ENSG00000166444	DENND2B	1.8737	4.55E-13	2.68E-12	18.7840
ENSG00000147168	IL2RG	1.8447	4.56E-13	2.68E-12	18.7813
ENSG00000161243	FBXO27	1.4232	4.62E-13	2.72E-12	18.7686
ENSG00000196296	ATP2A1	1.5147	4.69E-13	2.76E-12	18.7523
ENSG00000080839	RBL1	1.4475	4.71E-13	2.77E-12	18.7497
ENSG00000147274	RBMX	1.2318	4.73E-13	2.78E-12	18.7452
ENSG00000021488	SLC7A9	1.1492	4.77E-13	2.80E-12	18.7364
ENSG00000163564	PYHIN1	1.3102	4.82E-13	2.83E-12	18.7268
ENSG00000149305	HTR3B	1.1364	4.85E-13	2.85E-12	18.7200
ENSG00000144040	SFXN5	1.4098	4.89E-13	2.87E-12	18.7131
ENSG00000187049	TMEM216	0.7170	4.97E-13	2.92E-12	18.6963
ENSG00000131095	GFAP	1.2894	5.00E-13	2.94E-12	18.6897
ENSG00000111728	ST8SIA1	1.3657	5.04E-13	2.95E-12	18.6835
ENSG00000147459	DOCK5	1.3946	5.04E-13	2.96E-12	18.6824
ENSG00000139880	CDH24	1.3151	5.08E-13	2.98E-12	18.6756
ENSG00000173626	TRAPPC3L	1.2456	5.24E-13	3.07E-12	18.6440
ENSG00000182700	IGIP	0.6837	5.29E-13	3.10E-12	18.6360
ENSG00000187109	NAP1L1	1.2828	5.30E-13	3.10E-12	18.6337
ENSG00000171109	MFN1	1.3716	5.39E-13	3.16E-12	18.6167
ENSG00000185630	PBX1	1.5978	5.47E-13	3.20E-12	18.6025
ENSG00000226761	TAS2R46	1.1189	5.50E-13	3.22E-12	18.5974
ENSG00000173011	TADA2B	1.3490	5.58E-13	3.26E-12	18.5834
ENSG00000198604	BAZ1A	1.3622	5.58E-13	3.27E-12	18.5820
ENSG00000132155	RAF1	1.3809	5.59E-13	3.27E-12	18.5802
ENSG00000165671	NSD1	1.3343	5.63E-13	3.29E-12	18.5740
ENSG00000170581	STAT2	1.4925	5.68E-13	3.32E-12	18.5655
ENSG00000171105	INSR	1.6296	5.77E-13	3.37E-12	18.5495
ENSG00000047365	ARAP2	1.7905	5.83E-13	3.40E-12	18.5404
ENSG00000095110	NXPE1	0.5574	5.83E-13	3.41E-12	18.5390
ENSG00000197779	ZNF81	1.2865	5.85E-13	3.42E-12	18.5358
ENSG00000154040	CABYR	1.2489	5.92E-13	3.46E-12	18.5242
ENSG00000110002	VWA5A	1.8324	5.93E-13	3.46E-12	18.5235
ENSG00000179921	GPBAR1	1.2939	5.95E-13	3.47E-12	18.5204
ENSG00000152127	MGAT5	1.7465	6.01E-13	3.50E-12	18.5103
ENSG00000057252	SOAT1	1.4474	6.01E-13	3.50E-12	18.5093
ENSG00000165186	PTCHD1	1.0285	6.08E-13	3.54E-12	18.4980
ENSG00000163449	TMEM169	1.0619	6.10E-13	3.55E-12	18.4948
ENSG00000160013	PTGIR	1.1565	6.11E-13	3.56E-12	18.4939
ENSG00000168243	GNG4	1.0626	6.13E-13	3.57E-12	18.4902
ENSG00000150938	CRIM1	1.9512	6.17E-13	3.59E-12	18.4834

Gene ID	Gene Symbol	FC	P.Value	adj.P.Val	B
ENSG00000150593	PDCD4	1.7394	6.20E-13	3.61E-12	18.4792
ENSG00000269720	CCDC194	1.1964	6.22E-13	3.62E-12	18.4760
ENSG00000204815	ODAD4	1.3476	6.22E-13	3.62E-12	18.4757
ENSG00000060688	SNRNP40	1.2912	6.28E-13	3.65E-12	18.4674
ENSG00000165323	FAT3	1.0826	6.28E-13	3.65E-12	18.4670
ENSG00000129925	PGAP6	1.3407	6.39E-13	3.71E-12	18.4499
ENSG00000064205	CCN5	1.0977	6.41E-13	3.72E-12	18.4462
ENSG00000144802	NFKBIZ	1.8681	6.42E-13	3.73E-12	18.4443
ENSG00000165410	CFL2	1.5716	6.43E-13	3.73E-12	18.4435
ENSG00000117713	ARID1A	1.4184	6.52E-13	3.78E-12	18.4295
ENSG00000004777	ARHGAP33	1.5214	6.53E-13	3.78E-12	18.4291
ENSG00000104381	GDAP1	1.4392	6.53E-13	3.78E-12	18.4288
ENSG00000184047	DIABLO	1.2228	6.68E-13	3.87E-12	18.4068
ENSG00000184056	VPS33B	1.2658	6.69E-13	3.87E-12	18.4051
ENSG00000198858	R3HDM4	1.3511	6.84E-13	3.96E-12	18.3832
ENSG00000136718	IMP4	1.2888	6.86E-13	3.97E-12	18.3801
ENSG00000171517	LPAR3	1.0475	6.89E-13	3.99E-12	18.3762
ENSG00000267060	PTGES3L	1.2393	7.02E-13	4.06E-12	18.3571
ENSG00000101152	DNAJC5	1.3006	7.05E-13	4.08E-12	18.3527
ENSG00000113013	HSPA9	1.2665	7.10E-13	4.10E-12	18.3468
ENSG00000132466	ANKRD17	1.3168	7.15E-13	4.13E-12	18.3394
ENSG00000062096	ARSF	1.0494	7.17E-13	4.14E-12	18.3369
ENSG00000100341	PNPLA5	1.0832	7.19E-13	4.16E-12	18.3334
ENSG00000163041	H3-3A	1.4277	7.26E-13	4.19E-12	18.3238
ENSG00000105617	LENG1	0.6992	7.33E-13	4.23E-12	18.3156
ENSG00000104375	STK3	1.2836	7.34E-13	4.24E-12	18.3133
ENSG00000213145	CRIP1	3.2888	7.39E-13	4.26E-12	18.3067
ENSG00000083857	FAT1	1.2522	7.44E-13	4.29E-12	18.3005
ENSG00000185880	TRIM69	0.6467	7.51E-13	4.33E-12	18.2918
ENSG00000121764	HCRTR1	1.1123	7.53E-13	4.34E-12	18.2890
ENSG00000157227	MMP14	1.6443	7.57E-13	4.36E-12	18.2830
ENSG00000101003	GINS1	1.5100	7.58E-13	4.36E-12	18.2826
ENSG00000180532	ZSCAN4	1.0539	7.60E-13	4.38E-12	18.2790
ENSG00000240021	TEX35	1.2928	7.67E-13	4.42E-12	18.2703
ENSG00000163810	TGM4	1.2880	7.71E-13	4.44E-12	18.2654
ENSG00000166664	CHRFAM7A	1.2738	7.76E-13	4.47E-12	18.2589
ENSG00000111554	MDM1	1.3514	7.80E-13	4.48E-12	18.2543
ENSG00000160131	VMA21	1.3430	7.86E-13	4.52E-12	18.2462
ENSG00000066422	ZBTB11	1.3182	7.94E-13	4.56E-12	18.2371
ENSG00000071859	FAM50A	1.4129	7.98E-13	4.58E-12	18.2319
ENSG00000159210	SNF8	1.2688	8.06E-13	4.63E-12	18.2220
ENSG00000138172	CALHM2	1.7048	8.09E-13	4.65E-12	18.2179
ENSG00000119720	NRDE2	1.3113	8.13E-13	4.67E-12	18.2132
ENSG00000133422	MORC2	1.3546	8.16E-13	4.68E-12	18.2101
ENSG00000156983	BRPF1	1.3109	8.23E-13	4.72E-12	18.2015
ENSG00000173846	PLK3	1.9216	8.24E-13	4.72E-12	18.2007
ENSG00000121274	TENT4B	1.3376	8.29E-13	4.75E-12	18.1941
ENSG00000116212	LRRC42	1.3113	8.31E-13	4.76E-12	18.1923
ENSG00000171320	ESCO2	1.3798	8.34E-13	4.78E-12	18.1882
ENSG00000117281	CD160	1.2977	8.41E-13	4.82E-12	18.1802
ENSG00000070190	DAPP1	1.5162	8.53E-13	4.88E-12	18.1666
ENSG00000173141	MRPL57	0.7409	8.62E-13	4.93E-12	18.1561
ENSG00000163545	NUAK2	1.6439	8.66E-13	4.95E-12	18.1517
ENSG00000173020	GRK2	1.3701	8.74E-13	5.00E-12	18.1426

Gene ID	Gene Symbol	FC	P.Value	adj.P.Val	B
ENSG00000162073	PAQR4	1.6233	8.75E-13	5.01E-12	18.1409
ENSG00000142327	RNPEPL1	1.3396	8.92E-13	5.10E-12	18.1226
ENSG00000111012	CYP27B1	1.5153	9.12E-13	5.21E-12	18.1004
ENSG00000159111	MRPL10	1.3314	9.19E-13	5.25E-12	18.0931
ENSG00000164182	NDUFAF2	0.7421	9.19E-13	5.25E-12	18.0933
ENSG00000197601	FAR1	1.3601	9.37E-13	5.35E-12	18.0741
ENSG00000176125	UFSP1	0.6673	9.46E-13	5.40E-12	18.0646
ENSG00000187556	NANOS3	1.4848	9.48E-13	5.41E-12	18.0630
ENSG00000087074	PPP1R15A	1.7083	9.50E-13	5.42E-12	18.0600
ENSG00000175764	TTLL11	0.6936	9.63E-13	5.49E-12	18.0476
ENSG00000154767	XPC	1.3436	9.67E-13	5.51E-12	18.0432
ENSG00000187720	THSD4	1.0870	1.01E-12	5.74E-12	18.0029
ENSG00000116032	GRIN3B	1.1957	1.01E-12	5.77E-12	17.9981
ENSG00000152049	KCNE4	1.3595	1.02E-12	5.82E-12	17.9899
ENSG00000196712	NF1	1.4651	1.04E-12	5.91E-12	17.9745
ENSG00000116497	S100PBP	1.5212	1.04E-12	5.94E-12	17.9691
ENSG00000206579	XKR4	1.0249	1.05E-12	5.97E-12	17.9634
ENSG00000113712	CSNK1A1	1.2654	1.05E-12	5.97E-12	17.9629
ENSG00000182950	ODF3L1	1.2647	1.05E-12	5.99E-12	17.9592
ENSG00000175581	MRPL48	0.7014	1.06E-12	6.02E-12	17.9539
ENSG00000072121	ZFYVE26	1.3223	1.08E-12	6.17E-12	17.9304
ENSG00000182934	SRPRA	1.2993	1.09E-12	6.18E-12	17.9277
ENSG00000112096	SOD2	1.5627	1.09E-12	6.19E-12	17.9258
ENSG00000163584	RPL22L1	0.6512	1.11E-12	6.28E-12	17.9122
ENSG00000088766	CRLS1	0.7591	1.11E-12	6.32E-12	17.9045
ENSG00000073169	SELENOO	1.3112	1.12E-12	6.33E-12	17.9030
ENSG00000135070	ISCA1	1.2871	1.12E-12	6.35E-12	17.9004
ENSG00000107521	HPS1	1.2669	1.13E-12	6.41E-12	17.8903
ENSG00000270106	TSNAX-DISC1	1.1196	1.14E-12	6.48E-12	17.8791
ENSG00000111142	METAP2	1.2082	1.16E-12	6.58E-12	17.8636
ENSG00000173660	UQCRH	0.7753	1.19E-12	6.75E-12	17.8391
ENSG00000104679	R3HCC1	1.3534	1.19E-12	6.75E-12	17.8380
ENSG00000109920	FNBP4	1.3867	1.19E-12	6.75E-12	17.8377
ENSG00000101745	ANKRD12	1.4671	1.20E-12	6.77E-12	17.8352
ENSG00000151229	SLC2A13	1.3917	1.20E-12	6.82E-12	17.8273
ENSG00000100722	ZC3H14	1.2950	1.22E-12	6.89E-12	17.8175
ENSG00000168118	RAB4A	1.6736	1.22E-12	6.90E-12	17.8156
ENSG00000151135	TMEM263	1.3213	1.23E-12	6.93E-12	17.8111
ENSG00000104808	DHDH	1.2280	1.24E-12	7.00E-12	17.8007
ENSG00000153044	CENPH	1.5108	1.24E-12	7.01E-12	17.7996
ENSG00000170100	ZNF778	1.3850	1.25E-12	7.05E-12	17.7931
ENSG00000123636	BAZ2B	1.4232	1.26E-12	7.12E-12	17.7836
ENSG00000095585	BLNK	1.6445	1.26E-12	7.13E-12	17.7818
ENSG00000129187	DCTD	1.2409	1.27E-12	7.15E-12	17.7791
ENSG00000155366	RHOC	2.1771	1.27E-12	7.17E-12	17.7760
ENSG00000185960	SHOX	1.1123	1.28E-12	7.23E-12	17.7674
ENSG00000101182	PSMA7	1.2488	1.29E-12	7.28E-12	17.7606
ENSG00000130775	THEMIS2	1.9418	1.30E-12	7.36E-12	17.7495
ENSG00000197140	ADAM32	1.3098	1.31E-12	7.39E-12	17.7450
ENSG00000183624	HMCES	1.3377	1.39E-12	7.81E-12	17.6907
ENSG00000240891	PLCXD2	1.6930	1.39E-12	7.85E-12	17.6853
ENSG00000185621	LMLN	1.4343	1.40E-12	7.87E-12	17.6822
ENSG00000104218	CSPP1	1.3318	1.41E-12	7.92E-12	17.6757
ENSG00000133665	DYDC2	1.0889	1.41E-12	7.93E-12	17.6738

Gene ID	Gene Symbol	FC	P.Value	adj.P.Val	B
ENSG00000166949	SMAD3	1.6940	1.42E-12	7.98E-12	17.6673
ENSG00000197620	EOLA1	1.4155	1.42E-12	8.01E-12	17.6630
ENSG00000143669	LYST	1.4675	1.43E-12	8.05E-12	17.6581
ENSG00000198547	C20orf203	1.1142	1.44E-12	8.08E-12	17.6546
ENSG00000086065	CHMP5	0.6716	1.45E-12	8.15E-12	17.6456
ENSG00000143882	ATP6V1C2	1.4355	1.46E-12	8.18E-12	17.6412
ENSG00000137574	TGS1	1.3004	1.47E-12	8.27E-12	17.6308
ENSG00000205084	TMEM231	1.5181	1.50E-12	8.41E-12	17.6138
ENSG00000115234	SNX17	1.2166	1.50E-12	8.42E-12	17.6130
ENSG00000115053	NCL	1.3532	1.50E-12	8.44E-12	17.6101
ENSG00000114126	TFDP2	1.3019	1.51E-12	8.46E-12	17.6077
ENSG00000124243	BCAS4	0.5781	1.51E-12	8.49E-12	17.6030
ENSG00000152944	MED21	1.3253	1.52E-12	8.53E-12	17.5990
ENSG00000203867	RBM20	1.1681	1.52E-12	8.53E-12	17.5987
ENSG00000088002	SULT2B1	1.0672	1.52E-12	8.53E-12	17.5983
ENSG00000106025	TSPAN12	1.7456	1.54E-12	8.64E-12	17.5853
ENSG00000112659	CUL9	1.3407	1.56E-12	8.76E-12	17.5717
ENSG00000166851	PLK1	1.6186	1.59E-12	8.89E-12	17.5561
ENSG00000122783	CYREN	1.6024	1.61E-12	9.00E-12	17.5447
ENSG00000123843	C4BPB	1.3609	1.61E-12	9.00E-12	17.5438
ENSG00000076321	KLHL20	1.3067	1.62E-12	9.08E-12	17.5350
ENSG00000166886	NAB2	1.4971	1.64E-12	9.17E-12	17.5246
ENSG00000170579	DLGAP1	1.2169	1.65E-12	9.22E-12	17.5196
ENSG00000125814	NAPB	1.3267	1.65E-12	9.23E-12	17.5176
ENSG00000172354	GNB2	1.2961	1.69E-12	9.42E-12	17.4974
ENSG00000177464	GPR4	1.0935	1.69E-12	9.43E-12	17.4963
ENSG00000126945	HNRNPH2	1.3652	1.71E-12	9.55E-12	17.4841
ENSG00000005812	FBXL3	1.3226	1.72E-12	9.60E-12	17.4788
ENSG00000153560	UBP1	1.2821	1.75E-12	9.77E-12	17.4607
ENSG00000198668	CALM1	1.3599	1.76E-12	9.84E-12	17.4536
ENSG00000112511	PHF1	1.4624	1.77E-12	9.87E-12	17.4498
ENSG00000134574	DDB2	1.3880	1.77E-12	9.87E-12	17.4497
ENSG00000139631	CSAD	1.4775	1.78E-12	9.93E-12	17.4436
ENSG00000101104	PABPC1L	1.5895	1.78E-12	9.93E-12	17.4433
ENSG00000072195	SPEG	1.7139	1.81E-12	1.01E-11	17.4289
ENSG00000179841	AKAP5	1.3564	1.84E-12	1.03E-11	17.4114
ENSG00000144824	PHLDB2	1.5792	1.85E-12	1.03E-11	17.4079
ENSG00000168434	COG7	1.3596	1.87E-12	1.04E-11	17.3953
ENSG00000185347	TEDC1	1.5044	1.88E-12	1.04E-11	17.3927
ENSG00000172367	PDZD3	1.0926	1.89E-12	1.05E-11	17.3879
ENSG00000070756	PABPC1	1.2609	1.92E-12	1.07E-11	17.3696
ENSG00000134072	CAMK1	1.4725	1.92E-12	1.07E-11	17.3693
ENSG00000185869	ZNF829	1.2403	1.95E-12	1.09E-11	17.3539
ENSG00000122482	ZNF644	1.3228	1.96E-12	1.09E-11	17.3523
ENSG00000204655	MOG	1.1804	1.99E-12	1.10E-11	17.3376
ENSG00000113263	ITK	1.2184	1.99E-12	1.11E-11	17.3347
ENSG00000153140	CETN3	0.6030	1.99E-12	1.11E-11	17.3334
ENSG00000136048	DRAM1	1.3808	2.01E-12	1.11E-11	17.3264
ENSG00000173662	TAS1R1	1.2026	2.01E-12	1.12E-11	17.3234
ENSG00000086589	RBM22	1.2688	2.04E-12	1.13E-11	17.3112
ENSG00000204060	FOXO6	1.3633	2.05E-12	1.13E-11	17.3078
ENSG00000156482	RPL30	0.7751	2.07E-12	1.15E-11	17.2943
ENSG00000178878	APOLD1	1.3705	2.08E-12	1.15E-11	17.2918
ENSG00000122729	ACO1	0.6991	2.10E-12	1.16E-11	17.2837

Gene ID	Gene Symbol	FC	P.Value	adj.P.Val	B
ENSG00000102119	EMD	1.3684	2.11E-12	1.17E-11	17.2774
ENSG00000135596	MICAL1	1.5535	2.12E-12	1.17E-11	17.2753
ENSG00000105676	ARMC6	0.6528	2.13E-12	1.18E-11	17.2703
ENSG00000107223	EDF1	0.7825	2.13E-12	1.18E-11	17.2695
ENSG00000120440	TTLL2	1.0812	2.13E-12	1.18E-11	17.2668
ENSG00000181135	ZNF707	1.3247	2.15E-12	1.19E-11	17.2583
ENSG00000213949	ITGA1	1.1396	2.16E-12	1.19E-11	17.2566
ENSG00000090889	KIF4A	1.5175	2.16E-12	1.19E-11	17.2555
ENSG00000166448	TMEM130	1.1310	2.16E-12	1.19E-11	17.2552
ENSG00000070501	POLB	1.2615	2.18E-12	1.21E-11	17.2436
ENSG00000168314	MOBP	1.1012	2.21E-12	1.22E-11	17.2324
ENSG00000129657	SEC14L1	1.3537	2.25E-12	1.24E-11	17.2146
ENSG00000114316	USP4	1.2337	2.25E-12	1.24E-11	17.2128
ENSG00000167419	LPO	1.1163	2.26E-12	1.25E-11	17.2113
ENSG00000204172	AGAP9	1.4489	2.30E-12	1.27E-11	17.1942
ENSG00000146670	CDCA5	1.7041	2.34E-12	1.29E-11	17.1782
ENSG00000187775	DNAH17	1.2441	2.34E-12	1.29E-11	17.1777
ENSG00000122965	RBM19	1.3421	2.36E-12	1.30E-11	17.1673
ENSG00000145247	OCIAD2	1.2522	2.39E-12	1.32E-11	17.1559
ENSG00000164077	MON1A	0.6331	2.39E-12	1.32E-11	17.1553
ENSG00000003393	ALS2	1.4227	2.39E-12	1.32E-11	17.1549
ENSG00000163283	ALPP	1.1153	2.40E-12	1.32E-11	17.1530
ENSG00000187994	RINL	0.7014	2.43E-12	1.34E-11	17.1392
ENSG00000004975	DVL2	1.3466	2.43E-12	1.34E-11	17.1382
ENSG00000230873	STMND1	1.1025	2.45E-12	1.35E-11	17.1295
ENSG00000127666	TICAM1	1.4182	2.47E-12	1.36E-11	17.1248
ENSG00000011198	ABHD5	1.4180	2.47E-12	1.36E-11	17.1234
ENSG00000164430	CGAS	1.4162	2.49E-12	1.37E-11	17.1159
ENSG00000111877	MCM9	1.3263	2.51E-12	1.38E-11	17.1079
ENSG00000197619	ZNF615	1.3445	2.52E-12	1.38E-11	17.1041
ENSG00000065882	TBC1D1	1.5735	2.54E-12	1.39E-11	17.0955
ENSG00000119048	UBE2B	1.3555	2.55E-12	1.40E-11	17.0928
ENSG00000145107	TM4SF19	1.1277	2.58E-12	1.41E-11	17.0820
ENSG00000081059	TCF7	1.5301	2.58E-12	1.41E-11	17.0813
ENSG00000104859	CLASRP	1.3287	2.58E-12	1.42E-11	17.0795
ENSG00000152779	SLC16A12	1.0528	2.59E-12	1.42E-11	17.0763
ENSG00000175606	TMEM70	1.3338	2.61E-12	1.43E-11	17.0694
ENSG00000133103	COG6	0.6951	2.63E-12	1.44E-11	17.0611
ENSG00000131711	MAP1B	1.4210	2.66E-12	1.46E-11	17.0497
ENSG00000152503	TRIM36	1.5223	2.67E-12	1.46E-11	17.0486
ENSG00000089220	PEBP1	1.3614	2.71E-12	1.49E-11	17.0311
ENSG00000134545	KLRC1	1.3030	2.73E-12	1.50E-11	17.0234
ENSG00000105717	PBX4	1.8028	2.76E-12	1.51E-11	17.0127
ENSG00000111816	FRK	1.2818	2.78E-12	1.52E-11	17.0088
ENSG00000100596	SPTLC2	1.3523	2.78E-12	1.52E-11	17.0077
ENSG00000123600	METTL8	0.6496	2.79E-12	1.53E-11	17.0028
ENSG00000140943	MBTPS1	1.3167	2.81E-12	1.54E-11	16.9965
ENSG00000182979	MTA1	1.3954	2.82E-12	1.54E-11	16.9931
ENSG00000162959	MEMO1	1.2486	2.89E-12	1.58E-11	16.9686
ENSG00000159579	RSPRY1	1.3015	2.91E-12	1.59E-11	16.9634
ENSG00000055813	CCDC85A	0.7527	2.91E-12	1.59E-11	16.9609
ENSG00000094916	CBX5	1.4758	2.92E-12	1.59E-11	16.9585
ENSG00000138002	IFT172	1.4598	2.98E-12	1.63E-11	16.9395
ENSG00000121989	ACVR2A	1.4442	3.02E-12	1.65E-11	16.9261

Gene ID	Gene Symbol	FC	P.Value	adj.P.Val	B
ENSG00000103126	AXIN1	1.2959	3.02E-12	1.65E-11	16.9257
ENSG00000184602	SNN	1.6314	3.07E-12	1.67E-11	16.9110
ENSG00000105983	LMBR1	1.3121	3.08E-12	1.68E-11	16.9067
ENSG00000166479	TMX3	1.3853	3.13E-12	1.71E-11	16.8909
ENSG00000241878	PISD	1.2959	3.15E-12	1.71E-11	16.8862
ENSG00000152443	ZNF776	1.3822	3.16E-12	1.72E-11	16.8817
ENSG00000070601	FRMPD1	1.2052	3.18E-12	1.73E-11	16.8751
ENSG00000116337	AMPD2	1.4292	3.22E-12	1.75E-11	16.8637
ENSG00000175283	DOLK	0.6870	3.23E-12	1.76E-11	16.8598
ENSG00000133816	MICAL2	1.2997	3.25E-12	1.77E-11	16.8542
ENSG00000101974	ATP11C	1.4783	3.28E-12	1.78E-11	16.8448
ENSG00000083312	TNPO1	1.3192	3.30E-12	1.79E-11	16.8383
ENSG00000104408	EIF3E	0.7620	3.30E-12	1.79E-11	16.8384
ENSG00000097046	CDC7	1.5342	3.31E-12	1.80E-11	16.8350
ENSG00000128298	BAIAP2L2	1.3017	3.33E-12	1.81E-11	16.8311
ENSG00000139668	WDFY2	1.4424	3.33E-12	1.81E-11	16.8308
ENSG00000185900	POMK	1.4112	3.34E-12	1.81E-11	16.8279
ENSG00000131944	FAAP24	1.2370	3.34E-12	1.81E-11	16.8262
ENSG00000119979	DENND10	1.3517	3.37E-12	1.83E-11	16.8194
ENSG00000129221	AIPL1	1.0590	3.41E-12	1.85E-11	16.8083
ENSG00000163660	CCNL1	1.5250	3.41E-12	1.85E-11	16.8069
ENSG00000136003	ISCU	1.3655	3.41E-12	1.85E-11	16.8063
ENSG00000180209	MYLPF	1.3506	3.46E-12	1.88E-11	16.7923
ENSG00000090020	SLC9A1	1.2701	3.48E-12	1.89E-11	16.7864
ENSG00000112339	HBS1L	1.3472	3.57E-12	1.93E-11	16.7623
ENSG00000130176	CNN1	1.2112	3.62E-12	1.96E-11	16.7489
ENSG00000242732	RTL5	1.6430	3.67E-12	1.99E-11	16.7344
ENSG00000137747	TMPRSS13	1.1539	3.68E-12	1.99E-11	16.7328
ENSG00000198918	RPL39	0.7121	3.73E-12	2.02E-11	16.7194
ENSG00000131828	PDHA1	1.2882	3.73E-12	2.02E-11	16.7185
ENSG00000143727	ACP1	1.2121	3.76E-12	2.03E-11	16.7113
ENSG00000113430	IRX4	1.0302	3.78E-12	2.04E-11	16.7055
ENSG00000241343	RPL36A	0.7047	3.80E-12	2.05E-11	16.7017
ENSG00000126705	AHDC1	1.4295	3.83E-12	2.07E-11	16.6932
ENSG00000048462	TNFRSF17	0.5654	3.84E-12	2.07E-11	16.6903
ENSG00000114416	FXR1	1.2660	3.85E-12	2.08E-11	16.6879
ENSG00000112394	SLC16A10	1.4359	3.89E-12	2.10E-11	16.6789
ENSG00000135148	TRAFD1	1.3655	3.92E-12	2.11E-11	16.6704
ENSG00000149532	CPSF7	1.3162	3.93E-12	2.12E-11	16.6685
ENSG00000103047	TANGO6	1.2360	3.93E-12	2.12E-11	16.6674
ENSG00000197808	ZNF461	1.4387	4.00E-12	2.16E-11	16.6503
ENSG00000142208	AKT1	1.2901	4.01E-12	2.16E-11	16.6493
ENSG00000132681	ATP1A4	1.0904	4.02E-12	2.16E-11	16.6470
ENSG00000111859	NEDD9	0.5498	4.02E-12	2.17E-11	16.6447
ENSG00000104320	NBN	1.3642	4.03E-12	2.17E-11	16.6440
ENSG00000135127	BICDL1	1.5197	4.05E-12	2.18E-11	16.6385
ENSG00000105523	FAM83E	1.1858	4.11E-12	2.21E-11	16.6246
ENSG00000003987	MTMR7	1.2963	4.14E-12	2.23E-11	16.6175
ENSG00000141505	ASGR1	1.2414	4.16E-12	2.24E-11	16.6123
ENSG00000070423	RNF126	1.3205	4.16E-12	2.24E-11	16.6113
ENSG00000241484	ARHGAP8	1.7900	4.20E-12	2.26E-11	16.6032
ENSG00000133980	VRTN	1.0278	4.20E-12	2.26E-11	16.6018
ENSG00000157870	PRXL2B	1.4628	4.22E-12	2.26E-11	16.5993
ENSG00000161920	MED11	0.6519	4.22E-12	2.26E-11	16.5990

Gene ID	Gene Symbol	FC	P.Value	adj.P.Val	B
ENSG00000151287	TEX30	1.4255	4.24E-12	2.27E-11	16.5946
ENSG00000147799	ARHGAP39	1.3772	4.26E-12	2.29E-11	16.5886
ENSG00000171206	TRIM8	1.3698	4.27E-12	2.29E-11	16.5856
ENSG00000196419	XRCC6	1.2370	4.38E-12	2.35E-11	16.5619
ENSG00000204152	TIMM23B	1.5659	4.42E-12	2.37E-11	16.5522
ENSG00000108576	SLC6A4	1.1326	4.46E-12	2.39E-11	16.5450
ENSG00000141294	LRRC46	1.2578	4.46E-12	2.39E-11	16.5441
ENSG00000240065	PSMB9	0.6488	4.49E-12	2.40E-11	16.5381
ENSG00000150687	PRSS23	1.4079	4.52E-12	2.42E-11	16.5306
ENSG00000157999	ANKRD61	1.3014	4.52E-12	2.42E-11	16.5309
ENSG00000176386	CDC26	0.7589	4.54E-12	2.43E-11	16.5268
ENSG00000215472	RPL17-C18orf32	0.6893	4.59E-12	2.46E-11	16.5155
ENSG00000100242	SUN2	1.3654	4.64E-12	2.48E-11	16.5048
ENSG00000162063	CCNF	1.3556	4.64E-12	2.48E-11	16.5045
ENSG00000197483	ZNF628	1.3188	4.69E-12	2.51E-11	16.4951
ENSG00000011426	ANLN	1.5004	4.71E-12	2.52E-11	16.4903
ENSG00000198453	ZNF568	1.4150	4.74E-12	2.53E-11	16.4853
ENSG00000107745	MICU1	1.3188	4.75E-12	2.54E-11	16.4827
ENSG00000204130	RUFY2	1.2844	4.75E-12	2.54E-11	16.4825
ENSG00000189376	C8orf76	1.2821	4.75E-12	2.54E-11	16.4821
ENSG00000149021	SCGB1A1	1.1854	4.79E-12	2.56E-11	16.4732
ENSG00000118965	WDR35	1.3230	4.81E-12	2.57E-11	16.4703
ENSG00000197217	ENTPD4	1.4995	4.85E-12	2.59E-11	16.4610
ENSG00000141527	CARD14	1.2022	4.87E-12	2.60E-11	16.4577
ENSG00000204356	NELFE	1.2635	4.90E-12	2.61E-11	16.4523
ENSG00000259494	MRPL46	0.7069	4.92E-12	2.62E-11	16.4481
ENSG00000189350	TOGARAM2	1.1910	4.92E-12	2.62E-11	16.4472
ENSG00000183690	EFHC2	1.1716	4.97E-12	2.65E-11	16.4369
ENSG00000205323	SARNP	1.2830	4.98E-12	2.65E-11	16.4363
ENSG00000130826	DKC1	1.3779	5.09E-12	2.71E-11	16.4142
ENSG00000141562	NARF	1.3631	5.10E-12	2.71E-11	16.4133
ENSG00000261649	GOLGA6L7	1.1094	5.15E-12	2.74E-11	16.4022
ENSG00000205808	PLPP6	0.6919	5.19E-12	2.76E-11	16.3950
ENSG00000243156	MICAL3	1.4983	5.22E-12	2.77E-11	16.3907
ENSG00000059728	MXD1	1.5922	5.26E-12	2.80E-11	16.3815
ENSG00000085382	HACE1	1.3263	5.32E-12	2.83E-11	16.3707
ENSG00000170085	SIMC1	1.4998	5.40E-12	2.87E-11	16.3566
ENSG00000163655	GMPS	1.3176	5.47E-12	2.90E-11	16.3444
ENSG00000055070	SZRD1	1.2959	5.51E-12	2.92E-11	16.3375
ENSG00000009954	BAZ1B	1.3222	5.52E-12	2.93E-11	16.3356
ENSG00000103042	SLC38A7	1.3723	5.62E-12	2.98E-11	16.3170
ENSG00000112655	PTK7	1.2584	5.63E-12	2.99E-11	16.3153
ENSG00000181649	PHLDA2	1.6567	5.64E-12	2.99E-11	16.3145
ENSG00000128487	SPECC1	1.6307	5.65E-12	3.00E-11	16.3125
ENSG00000204913	LRRC3C	1.1343	5.76E-12	3.06E-11	16.2925
ENSG00000127837	AAMP	1.2705	5.78E-12	3.06E-11	16.2901
ENSG00000108370	RGS9	1.1869	5.82E-12	3.08E-11	16.2834
ENSG00000115286	NDUFS7	0.7467	5.83E-12	3.09E-11	16.2808
ENSG00000168334	XIRP1	1.4362	5.98E-12	3.17E-11	16.2566
ENSG00000138326	RPS24	0.7689	6.04E-12	3.20E-11	16.2469
ENSG00000111305	GSG1	1.1599	6.10E-12	3.23E-11	16.2369
ENSG00000096654	ZNF184	1.3199	6.21E-12	3.28E-11	16.2198
ENSG00000087510	TFAP2C	1.8487	6.34E-12	3.35E-11	16.1994
ENSG00000143850	PLEKHA6	1.1956	6.37E-12	3.37E-11	16.1942

Gene ID	Gene Symbol	FC	P.Value	adj.P.Val	B
ENSG00000116771	AGMAT	1.5759	6.43E-12	3.40E-11	16.1851
ENSG00000183605	SFXN4	0.7081	6.47E-12	3.42E-11	16.1791
ENSG00000095739	BAMBI	1.9925	6.55E-12	3.46E-11	16.1669
ENSG00000213186	TRIM59	1.3188	6.56E-12	3.47E-11	16.1652
ENSG00000145901	TNIP1	1.5644	6.57E-12	3.47E-11	16.1647
ENSG00000113262	GRM6	1.0451	6.64E-12	3.51E-11	16.1540
ENSG00000139865	TTC6	1.2446	6.66E-12	3.52E-11	16.1504
ENSG00000187866	PABIR1	0.6675	6.67E-12	3.52E-11	16.1498
ENSG00000166333	ILK	1.3501	6.69E-12	3.53E-11	16.1466
ENSG00000100804	PSMB5	0.7712	6.69E-12	3.53E-11	16.1461
ENSG00000046604	DSG2	2.3808	6.73E-12	3.55E-11	16.1412
ENSG00000164091	WDR82	1.2577	6.76E-12	3.56E-11	16.1358
ENSG00000099284	MACROH2A2	1.1472	6.88E-12	3.63E-11	16.1185
ENSG00000124191	TOX2	1.7823	6.93E-12	3.65E-11	16.1114
ENSG00000198740	ZNF652	1.3051	6.94E-12	3.65E-11	16.1110
ENSG00000135749	PCNX2	1.6024	6.98E-12	3.67E-11	16.1051
ENSG00000085185	BCORL1	1.4422	7.06E-12	3.72E-11	16.0938
ENSG00000072182	ASIC4	1.1552	7.11E-12	3.74E-11	16.0868
ENSG00000198324	PHETA1	1.3223	7.21E-12	3.79E-11	16.0726
ENSG00000103160	HSDL1	1.3881	7.26E-12	3.82E-11	16.0662
ENSG00000157600	TMEM164	1.4672	7.30E-12	3.84E-11	16.0606
ENSG00000172757	CFL1	1.2885	7.48E-12	3.93E-11	16.0368
ENSG00000178718	RPP25	0.5155	7.51E-12	3.94E-11	16.0338
ENSG00000106258	CYP3A5	1.3193	7.56E-12	3.97E-11	16.0272
ENSG00000143222	UFC1	1.2802	7.57E-12	3.97E-11	16.0261
ENSG00000196700	ZNF512B	1.4781	7.65E-12	4.02E-11	16.0147
ENSG00000139505	MTMR6	1.3221	7.72E-12	4.05E-11	16.0059
ENSG00000184389	A3GALT2	1.1061	7.88E-12	4.14E-11	15.9859
ENSG00000008405	CRY1	1.6215	7.91E-12	4.15E-11	15.9824
ENSG00000062582	MRPS24	0.7311	7.91E-12	4.15E-11	15.9826
ENSG00000149970	CNKSR2	1.3804	7.96E-12	4.17E-11	15.9768
ENSG00000111412	SPRING1	1.3702	7.98E-12	4.18E-11	15.9738
ENSG00000185624	P4HB	1.2866	8.00E-12	4.19E-11	15.9711
ENSG00000160972	PPP1R16A	1.3721	8.04E-12	4.21E-11	15.9671
ENSG00000205981	DNAJC19	0.7058	8.03E-12	4.21E-11	15.9673
ENSG00000256222	MTRNR2L3	1.0707	8.05E-12	4.21E-11	15.9658
ENSG00000110400	NECTIN1	1.7059	8.20E-12	4.29E-11	15.9478
ENSG00000113194	FAF2	1.2722	8.27E-12	4.33E-11	15.9385
ENSG00000116863	ADPRS	1.2957	8.40E-12	4.39E-11	15.9237
ENSG00000102882	MAPK3	1.4106	8.41E-12	4.40E-11	15.9226
ENSG00000130522	JUND	1.5776	8.48E-12	4.43E-11	15.9149
ENSG00000144021	CIAO1	1.2345	8.57E-12	4.48E-11	15.9036
ENSG00000184517	ZFP1	1.3393	8.72E-12	4.55E-11	15.8875
ENSG00000104432	IL7	1.5612	8.79E-12	4.59E-11	15.8796
ENSG00000145685	LHFPL2	1.5797	8.78E-12	4.59E-11	15.8798
ENSG00000138459	SLC35A5	0.6254	8.85E-12	4.62E-11	15.8720
ENSG00000171940	ZNF217	1.4827	9.08E-12	4.74E-11	15.8472
ENSG00000161996	WDR90	1.7340	9.12E-12	4.76E-11	15.8426
ENSG00000179222	MAGED1	1.5013	9.18E-12	4.79E-11	15.8371
ENSG00000065135	GNAI3	1.3169	9.23E-12	4.82E-11	15.8309
ENSG00000143155	TIPRL	1.3190	9.24E-12	4.82E-11	15.8304
ENSG00000134046	MBD2	1.4050	9.28E-12	4.84E-11	15.8258
ENSG00000196507	TCEAL3	1.5613	9.35E-12	4.87E-11	15.8190
ENSG00000188786	MTF1	1.2732	9.41E-12	4.90E-11	15.8125

Gene ID	Gene Symbol	FC	P.Value	adj.P.Val	B
ENSG00000114124	GRK7	1.0978	9.44E-12	4.91E-11	15.8093
ENSG00000198887	SMC5	1.2761	9.44E-12	4.91E-11	15.8094
ENSG00000240230	COX19	1.2688	9.64E-12	5.02E-11	15.7883
ENSG00000111344	RASAL1	1.7588	9.66E-12	5.02E-11	15.7867
ENSG00000116857	TMEM9	1.4783	9.66E-12	5.02E-11	15.7869
ENSG00000146909	NOM1	1.3546	9.74E-12	5.07E-11	15.7785
ENSG00000151746	BICD1	1.3820	9.77E-12	5.08E-11	15.7757
ENSG00000092847	AGO1	1.3106	9.97E-12	5.18E-11	15.7559
ENSG00000115266	APC2	0.7905	1.00E-11	5.21E-11	15.7492
ENSG00000137312	FLOT1	1.3099	1.01E-11	5.23E-11	15.7455
ENSG00000025434	NR1H3	0.6722	1.01E-11	5.27E-11	15.7388
ENSG00000179133	C10orf67	1.0832	1.03E-11	5.37E-11	15.7193
ENSG00000107242	PIP5K1B	0.5982	1.04E-11	5.40E-11	15.7134
ENSG00000093134	VNN3	1.3708	1.05E-11	5.43E-11	15.7077
ENSG00000120314	WDR55	1.2915	1.09E-11	5.67E-11	15.6653
ENSG00000120727	PAIP2	1.2316	1.10E-11	5.69E-11	15.6621
ENSG00000148660	CAMK2G	1.2988	1.11E-11	5.76E-11	15.6504
ENSG00000181626	ANKRD62	1.0654	1.12E-11	5.83E-11	15.6375
ENSG00000143947	RPS27A	0.7869	1.13E-11	5.84E-11	15.6362
ENSG00000139675	HNRNPA1L2	1.2977	1.13E-11	5.84E-11	15.6356
ENSG00000204227	RING1	1.2780	1.14E-11	5.89E-11	15.6270
ENSG00000160716	CHRNB2	1.0630	1.14E-11	5.90E-11	15.6249
ENSG00000088727	KIF9	1.4067	1.15E-11	5.96E-11	15.6151
ENSG00000165804	ZNF219	1.4551	1.16E-11	5.99E-11	15.6089
ENSG00000187091	PLCD1	1.3862	1.16E-11	6.00E-11	15.6078
ENSG00000222009	BTBD19	1.5354	1.16E-11	6.00E-11	15.6069
ENSG00000169914	OTUD3	1.3182	1.16E-11	6.01E-11	15.6053
ENSG00000090621	PABPC4	1.4215	1.17E-11	6.06E-11	15.5963
ENSG00000112561	TFEB	1.6739	1.17E-11	6.06E-11	15.5963
ENSG00000124713	GNMT	1.3388	1.19E-11	6.13E-11	15.5844
ENSG00000112941	TENT4A	1.5001	1.19E-11	6.14E-11	15.5826
ENSG00000101384	JAG1	1.5681	1.20E-11	6.18E-11	15.5767
ENSG00000136877	FPGS	1.2614	1.20E-11	6.20E-11	15.5728
ENSG00000174521	TTC9B	1.3425	1.21E-11	6.23E-11	15.5683
ENSG00000121406	ZNF549	1.4488	1.21E-11	6.25E-11	15.5641
ENSG00000164975	SNAPC3	0.6361	1.21E-11	6.25E-11	15.5639
ENSG00000167930	FAM234A	1.3232	1.24E-11	6.42E-11	15.5386
ENSG00000140199	SLC12A6	1.5960	1.25E-11	6.45E-11	15.5336
ENSG00000134371	CDC73	1.3603	1.25E-11	6.45E-11	15.5329
ENSG00000165495	PKNOX2	1.2083	1.27E-11	6.56E-11	15.5165
ENSG00000073712	FERMT2	1.6905	1.27E-11	6.57E-11	15.5150
ENSG00000126353	CCR7	1.6378	1.28E-11	6.59E-11	15.5117
ENSG00000228300	FAM174C	0.7414	1.29E-11	6.63E-11	15.5056
ENSG00000250479	CHCHD10	0.7130	1.29E-11	6.66E-11	15.5008
ENSG00000235162	C12orf75	2.1712	1.29E-11	6.66E-11	15.5000
ENSG00000173013	CCDC96	1.2857	1.30E-11	6.66E-11	15.4993
ENSG00000147036	LANCL3	1.1199	1.30E-11	6.67E-11	15.4976
ENSG00000132481	TRIM47	1.6080	1.30E-11	6.71E-11	15.4924
ENSG00000163069	SGCB	1.7895	1.31E-11	6.75E-11	15.4861
ENSG00000134419	RPS15A	0.7773	1.33E-11	6.81E-11	15.4763
ENSG00000132792	CTNNBL1	1.2768	1.33E-11	6.81E-11	15.4759
ENSG00000196917	HCAR1	1.1152	1.33E-11	6.85E-11	15.4701
ENSG00000125910	S1PR4	0.3985	1.34E-11	6.89E-11	15.4651
ENSG00000197771	MCMBP	1.3060	1.34E-11	6.89E-11	15.4638

Gene ID	Gene Symbol	FC	P.Value	adj.P.Val	B
ENSG00000129810	SGO1	1.4380	1.35E-11	6.92E-11	15.4599
ENSG00000146147	MLIP	0.3911	1.37E-11	7.02E-11	15.4454
ENSG00000162399	BSND	1.0424	1.38E-11	7.06E-11	15.4401
ENSG00000171202	TMEM126A	0.7606	1.38E-11	7.07E-11	15.4382
ENSG00000089009	RPL6	0.8009	1.39E-11	7.14E-11	15.4287
ENSG00000143164	DCAF6	1.2903	1.40E-11	7.15E-11	15.4258
ENSG00000204175	GPRIN2	1.0166	1.40E-11	7.19E-11	15.4209
ENSG00000168955	TM4SF20	1.1256	1.41E-11	7.24E-11	15.4141
ENSG00000169564	PCBP1	1.2481	1.42E-11	7.29E-11	15.4064
ENSG00000108826	MRPL27	0.6639	1.43E-11	7.34E-11	15.4001
ENSG00000092094	OSGEP	1.3206	1.44E-11	7.36E-11	15.3971
ENSG00000168594	ADAM29	1.1538	1.45E-11	7.41E-11	15.3903
ENSG00000186496	ZNF396	1.3422	1.45E-11	7.42E-11	15.3886
ENSG00000175106	TVP23C	1.4877	1.46E-11	7.44E-11	15.3852
ENSG00000213722	DDAH2	1.4718	1.46E-11	7.48E-11	15.3800
ENSG00000141946	ZIM3	1.0364	1.47E-11	7.53E-11	15.3731
ENSG00000145861	C1QTNF2	1.0506	1.48E-11	7.54E-11	15.3711
ENSG00000125089	SH3TC1	2.1138	1.48E-11	7.57E-11	15.3678
ENSG00000011052	NME1-NME2	0.7365	1.51E-11	7.73E-11	15.3472
ENSG00000109184	DCUN1D4	1.3867	1.52E-11	7.77E-11	15.3416
ENSG00000120008	WDR11	1.4192	1.53E-11	7.79E-11	15.3381
ENSG00000174697	LEP	1.0371	1.55E-11	7.89E-11	15.3258
ENSG00000105486	LIG1	1.4414	1.56E-11	7.93E-11	15.3199
ENSG00000116793	PHTF1	1.3552	1.58E-11	8.08E-11	15.3018
ENSG00000130173	ANGPTL8	1.2040	1.59E-11	8.12E-11	15.2967
ENSG00000165506	DNAAF2	1.4297	1.59E-11	8.12E-11	15.2969
ENSG00000148358	GPR107	1.2757	1.60E-11	8.13E-11	15.2956
ENSG00000106052	TAX1BP1	1.2184	1.60E-11	8.14E-11	15.2934
ENSG00000170876	TMEM43	1.3187	1.60E-11	8.14E-11	15.2927
ENSG00000145087	STXBP5L	1.3644	1.60E-11	8.15E-11	15.2921
ENSG00000254999	BRK1	0.8132	1.61E-11	8.19E-11	15.2866
ENSG00000105851	PIK3CG	1.6582	1.61E-11	8.20E-11	15.2857
ENSG00000104267	CA2	1.9153	1.61E-11	8.20E-11	15.2849
ENSG00000186265	BTLA	0.4379	1.62E-11	8.21E-11	15.2833
ENSG00000198258	UBL5	0.7779	1.62E-11	8.23E-11	15.2806
ENSG00000107625	DDX50	1.2302	1.64E-11	8.32E-11	15.2699
ENSG00000122390	NAA60	1.2834	1.64E-11	8.33E-11	15.2682
ENSG00000122335	SERAC1	1.2558	1.64E-11	8.35E-11	15.2665
ENSG00000161533	ACOX1	1.2622	1.64E-11	8.35E-11	15.2661
ENSG00000140832	MARVELD3	1.2395	1.65E-11	8.35E-11	15.2651
ENSG00000104613	INTS10	1.3354	1.65E-11	8.36E-11	15.2640
ENSG00000161939	RNASEK-C17orf49	1.4055	1.66E-11	8.42E-11	15.2564
ENSG00000185261	KIAA0825	1.1923	1.69E-11	8.56E-11	15.2408
ENSG00000170703	TTLL6	1.1480	1.70E-11	8.60E-11	15.2357
ENSG00000183943	PRKX	1.5325	1.71E-11	8.69E-11	15.2252
ENSG00000033100	CHPF2	1.3484	1.72E-11	8.74E-11	15.2193
ENSG00000103426	CORO7-PAM16	1.2724	1.74E-11	8.81E-11	15.2107
ENSG00000132436	FIGNL1	1.4653	1.75E-11	8.88E-11	15.2026
ENSG00000128881	TTBK2	1.2856	1.76E-11	8.91E-11	15.1998
ENSG00000147592	LACTB2	0.6134	1.76E-11	8.92E-11	15.1984
ENSG00000134440	NARS1	1.2999	1.77E-11	8.97E-11	15.1929
ENSG00000178999	AURKB	1.7166	1.78E-11	9.01E-11	15.1875
ENSG00000196459	TRAPPC2	1.2348	1.79E-11	9.03E-11	15.1849
ENSG00000109787	KLF3	1.5904	1.82E-11	9.18E-11	15.1689

Gene ID	Gene Symbol	FC	P.Value	adj.P.Val	B
ENSG00000149485	FADS1	1.6840	1.82E-11	9.20E-11	15.1664
ENSG00000114735	HEMK1	0.7006	1.82E-11	9.21E-11	15.1649
ENSG00000251503	CENPS-CORT	1.2067	1.82E-11	9.21E-11	15.1652
ENSG00000139180	NDUFA9	1.2331	1.83E-11	9.22E-11	15.1633
ENSG00000010818	HIVEP2	1.4101	1.84E-11	9.30E-11	15.1552
ENSG00000165912	PACSIN3	1.6570	1.84E-11	9.30E-11	15.1552
ENSG00000150961	SEC24D	1.3543	1.85E-11	9.32E-11	15.1526
ENSG00000140682	TGFB1I1	1.1982	1.87E-11	9.44E-11	15.1392
ENSG00000062194	GPBP1	1.2461	1.88E-11	9.50E-11	15.1328
ENSG00000106070	GRB10	1.3941	1.89E-11	9.52E-11	15.1304
ENSG00000128789	PSMG2	1.2728	1.90E-11	9.56E-11	15.1265
ENSG00000101665	SMAD7	1.6910	1.90E-11	9.60E-11	15.1224
ENSG00000114480	GBE1	1.2842	1.91E-11	9.62E-11	15.1202
ENSG00000115290	GRB14	1.9426	1.93E-11	9.70E-11	15.1114
ENSG00000159217	IGF2BP1	1.1800	1.94E-11	9.79E-11	15.1024
ENSG00000092098	RNF31	1.2854	1.94E-11	9.79E-11	15.1014
ENSG00000152527	PLEKHH2	1.1984	1.99E-11	1.00E-10	15.0785
ENSG00000132702	HAPLN2	1.1211	2.00E-11	1.01E-10	15.0750
ENSG00000103194	USP10	1.2899	2.01E-11	1.01E-10	15.0706
ENSG00000213658	LAT	1.5642	2.01E-11	1.01E-10	15.0692
ENSG00000167863	ATP5PD	0.7670	2.02E-11	1.02E-10	15.0638
ENSG00000185024	BRF1	1.3077	2.03E-11	1.02E-10	15.0618
ENSG00000165046	LETM2	1.4597	2.04E-11	1.03E-10	15.0537
ENSG00000243749	TMEM35B	0.6628	2.05E-11	1.03E-10	15.0494
ENSG00000135763	URB2	1.3610	2.07E-11	1.04E-10	15.0408
ENSG00000160471	COX6B2	1.2855	2.08E-11	1.05E-10	15.0350
ENSG00000101457	DNTTIP1	1.2840	2.10E-11	1.06E-10	15.0254
ENSG00000106868	SUSD1	1.5793	2.12E-11	1.06E-10	15.0187
ENSG00000183484	GPR132	1.4564	2.15E-11	1.08E-10	15.0049
ENSG00000147533	GOLGA7	1.2828	2.17E-11	1.09E-10	14.9961
ENSG00000121579	NAA50	1.3274	2.17E-11	1.09E-10	14.9921
ENSG00000172794	RAB37	1.8939	2.18E-11	1.09E-10	14.9910
ENSG00000253352	TUG1	1.3463	2.18E-11	1.09E-10	14.9895
ENSG00000180628	PCGF5	1.4529	2.22E-11	1.11E-10	14.9735
ENSG00000163602	RYBP	1.3945	2.22E-11	1.11E-10	14.9712
ENSG00000267467	APOC4	1.2760	2.24E-11	1.12E-10	14.9640
ENSG00000198034	RPS4X	0.7400	2.24E-11	1.12E-10	14.9619
ENSG00000087157	PGS1	1.2988	2.26E-11	1.13E-10	14.9537
ENSG00000181827	RFX7	1.4123	2.26E-11	1.13E-10	14.9526
ENSG00000112218	GPR63	1.6113	2.29E-11	1.15E-10	14.9419
ENSG00000114631	PODXL2	2.3479	2.30E-11	1.15E-10	14.9360
ENSG00000130714	POMT1	1.3683	2.31E-11	1.15E-10	14.9349
ENSG00000184545	DUSP8	1.8565	2.32E-11	1.16E-10	14.9301
ENSG00000169641	LUZP1	1.2958	2.39E-11	1.20E-10	14.8992
ENSG00000039600	SOX30	0.7679	2.40E-11	1.20E-10	14.8968
ENSG00000198910	L1CAM	1.2108	2.41E-11	1.20E-10	14.8922
ENSG00000133657	ATP13A3	1.4228	2.43E-11	1.21E-10	14.8823
ENSG00000122545	SEPTIN7	1.2431	2.45E-11	1.22E-10	14.8765
ENSG00000204219	TCEA3	2.1690	2.48E-11	1.24E-10	14.8647
ENSG00000116489	CAPZA1	1.2692	2.48E-11	1.24E-10	14.8624
ENSG00000151690	MFSD6	1.4399	2.49E-11	1.24E-10	14.8603
ENSG00000110514	MADD	1.3162	2.51E-11	1.25E-10	14.8530
ENSG00000166578	IQCD	1.0756	2.51E-11	1.25E-10	14.8519
ENSG00000165030	NFIL3	1.6047	2.51E-11	1.25E-10	14.8512

Gene ID	Gene Symbol	FC	P.Value	adj.P.Val	B
ENSG00000163684	RPP14	1.2274	2.52E-11	1.25E-10	14.8490
ENSG00000176871	WSB2	1.4560	2.53E-11	1.26E-10	14.8436
ENSG00000134940	ACRV1	1.0975	2.60E-11	1.29E-10	14.8187
ENSG00000101935	AMMECR1	1.3451	2.60E-11	1.29E-10	14.8179
ENSG00000197858	GPAA1	1.3403	2.62E-11	1.31E-10	14.8085
ENSG00000104998	IL27RA	1.9587	2.63E-11	1.31E-10	14.8042
ENSG00000166797	CIAO2A	0.7799	2.67E-11	1.33E-10	14.7897
ENSG00000132677	RHBG	1.1905	2.68E-11	1.33E-10	14.7891
ENSG00000205903	ZNF316	1.3423	2.70E-11	1.34E-10	14.7811
ENSG00000165240	ATP7A	1.2722	2.73E-11	1.36E-10	14.7699
ENSG00000224420	ADM5	1.4570	2.73E-11	1.36E-10	14.7685
ENSG00000129451	KLK10	1.1192	2.74E-11	1.36E-10	14.7648
ENSG00000174132	FAM174A	0.6593	2.75E-11	1.37E-10	14.7616
ENSG00000213020	ZNF611	1.3352	2.76E-11	1.37E-10	14.7591
ENSG00000054654	SYNE2	1.6372	2.80E-11	1.39E-10	14.7434
ENSG00000125657	TNFSF9	1.4573	2.81E-11	1.39E-10	14.7417
ENSG00000137168	PPIL1	0.6306	2.81E-11	1.40E-10	14.7396
ENSG00000171863	RPS7	0.7946	2.83E-11	1.40E-10	14.7343
ENSG00000119669	IRF2BPL	1.4280	2.85E-11	1.41E-10	14.7289
ENSG00000144712	CAND2	1.5304	2.87E-11	1.42E-10	14.7208
ENSG00000159708	LRRC36	1.1414	2.92E-11	1.45E-10	14.7025
ENSG00000120733	KDM3B	1.2979	2.93E-11	1.45E-10	14.6996
ENSG00000042088	TDP1	1.3246	2.94E-11	1.46E-10	14.6969
ENSG00000068650	ATP11A	1.9749	2.96E-11	1.47E-10	14.6909
ENSG00000147604	RPL7	0.7695	2.98E-11	1.48E-10	14.6826
ENSG00000160961	ZNF333	1.3123	2.99E-11	1.48E-10	14.6808
ENSG00000134444	RELCH	1.3649	2.99E-11	1.48E-10	14.6796
ENSG00000163918	RFC4	1.3824	3.00E-11	1.48E-10	14.6782
ENSG00000139725	RHOF	1.5344	3.03E-11	1.50E-10	14.6677
ENSG00000158636	EMSY	1.3088	3.03E-11	1.50E-10	14.6662
ENSG00000161217	PCYT1A	1.2611	3.05E-11	1.51E-10	14.6623
ENSG00000248167	TRIM39-RPP21	1.1274	3.06E-11	1.51E-10	14.6580
ENSG00000134508	CABLES1	2.0507	3.08E-11	1.52E-10	14.6521
ENSG00000104967	NOVA2	1.1140	3.08E-11	1.52E-10	14.6509
ENSG00000180573	H2AC6	0.5253	3.09E-11	1.52E-10	14.6498
ENSG00000170935	NCBP2L	1.3002	3.15E-11	1.55E-10	14.6304
ENSG00000039139	DNAH5	1.1121	3.16E-11	1.56E-10	14.6276
ENSG00000168758	SEMA4C	1.5072	3.16E-11	1.56E-10	14.6263
ENSG00000072415	PALS1	1.2945	3.21E-11	1.59E-10	14.6103
ENSG00000129667	RHBDF2	1.3863	3.25E-11	1.60E-10	14.5998
ENSG00000175806	MSRA	1.5029	3.29E-11	1.62E-10	14.5884
ENSG00000132640	BTBD3	0.3847	3.32E-11	1.64E-10	14.5786
ENSG00000159267	HLCS	1.4634	3.33E-11	1.64E-10	14.5761
ENSG00000085832	EPS15	1.3373	3.33E-11	1.64E-10	14.5749
ENSG00000117262	GPR89A	1.3452	3.36E-11	1.65E-10	14.5670
ENSG00000227877	MRLN	1.2850	3.37E-11	1.66E-10	14.5643
ENSG00000174695	TMEM167A	0.7589	3.41E-11	1.68E-10	14.5506
ENSG00000171792	RHNO1	1.3479	3.42E-11	1.68E-10	14.5497
ENSG00000107864	CPEB3	1.3026	3.42E-11	1.68E-10	14.5486
ENSG00000140534	TICRR	1.3336	3.43E-11	1.69E-10	14.5474
ENSG00000183337	BCOR	1.4054	3.44E-11	1.69E-10	14.5435
ENSG00000171017	LRRC8E	1.2214	3.46E-11	1.70E-10	14.5386
ENSG00000134668	SPOCD1	1.1387	3.47E-11	1.70E-10	14.5359
ENSG00000150401	DCUN1D2	1.3369	3.48E-11	1.71E-10	14.5324

Gene ID	Gene Symbol	FC	P.Value	adj.P.Val	B
ENSG00000198917	SPOUT1	0.6844	3.49E-11	1.71E-10	14.5294
ENSG00000162613	FUBP1	1.3244	3.51E-11	1.73E-10	14.5224
ENSG00000148735	PLEKHS1	1.3704	3.52E-11	1.73E-10	14.5202
ENSG00000125457	MIF4GD	1.3241	3.56E-11	1.75E-10	14.5107
ENSG00000089682	RBM41	1.3655	3.56E-11	1.75E-10	14.5085
ENSG00000126583	PRKCG	1.2612	3.60E-11	1.76E-10	14.4999
ENSG00000186871	ERCC6L	1.1917	3.61E-11	1.77E-10	14.4956
ENSG00000137177	KIF13A	1.4259	3.62E-11	1.77E-10	14.4937
ENSG00000148773	MKI67	1.6864	3.63E-11	1.78E-10	14.4898
ENSG00000103245	CIAO3	1.3116	3.64E-11	1.78E-10	14.4894
ENSG00000113312	TTC1	0.7107	3.64E-11	1.79E-10	14.4870
ENSG00000180479	ZNF571	1.4080	3.73E-11	1.83E-10	14.4638
ENSG00000162543	UBXN10	1.1919	3.75E-11	1.83E-10	14.4601
ENSG00000189308	LIN54	1.2987	3.75E-11	1.84E-10	14.4585
ENSG00000102781	KATNAL1	1.2916	3.78E-11	1.85E-10	14.4514
ENSG00000198522	GPN1	1.2080	3.79E-11	1.86E-10	14.4483
ENSG00000167700	MFSD3	0.6997	3.80E-11	1.86E-10	14.4460
ENSG00000138111	MFSD13A	1.2934	3.82E-11	1.87E-10	14.4405
ENSG00000215695	RSC1A1	1.3762	3.83E-11	1.87E-10	14.4387
ENSG00000168646	AXIN2	1.7164	3.85E-11	1.88E-10	14.4321
ENSG00000124449	IRGC	1.0687	3.90E-11	1.91E-10	14.4211
ENSG00000156042	CFAP70	1.4174	3.90E-11	1.91E-10	14.4197
ENSG00000185305	ARL15	0.7523	3.98E-11	1.95E-10	14.3998
ENSG00000121207	LRAT	1.0467	3.99E-11	1.95E-10	14.3989
ENSG00000166716	ZNF592	1.3057	4.03E-11	1.97E-10	14.3888
ENSG00000077616	NAALAD2	1.3369	4.05E-11	1.98E-10	14.3841
ENSG00000119559	C19orf25	0.7355	4.05E-11	1.98E-10	14.3826
ENSG00000112182	BACH2	1.6832	4.07E-11	1.99E-10	14.3784
ENSG00000169826	CSGALNACT2	1.3809	4.16E-11	2.03E-10	14.3578
ENSG00000082898	XPO1	1.3598	4.20E-11	2.05E-10	14.3476
ENSG00000177370	TIMM22	1.2139	4.21E-11	2.05E-10	14.3449
ENSG00000125726	CD70	1.7047	4.28E-11	2.09E-10	14.3286
ENSG00000145349	CAMK2D	1.6242	4.32E-11	2.10E-10	14.3213
ENSG00000157045	NTAN1	1.4854	4.35E-11	2.12E-10	14.3147
ENSG00000105397	TYK2	1.2831	4.37E-11	2.13E-10	14.3097
ENSG00000141664	ZCCHC2	1.8113	4.51E-11	2.20E-10	14.2777
ENSG00000118655	DCLRE1B	1.2433	4.60E-11	2.24E-10	14.2600
ENSG00000164506	STXBP5	1.3677	4.68E-11	2.28E-10	14.2420
ENSG00000031823	RANBP3	1.2514	4.72E-11	2.29E-10	14.2345
ENSG00000158428	CATIP	1.1557	4.73E-11	2.30E-10	14.2326
ENSG00000100055	CYTH4	1.5581	4.76E-11	2.31E-10	14.2264
ENSG00000146109	ABT1	1.2510	4.78E-11	2.32E-10	14.2209
ENSG00000108960	MMD	1.4449	4.78E-11	2.33E-10	14.2206
ENSG00000124160	NCOA5	1.2757	4.80E-11	2.33E-10	14.2166
ENSG00000177283	FZD8	1.4286	4.82E-11	2.34E-10	14.2126
ENSG00000118939	UCHL3	0.7569	4.83E-11	2.35E-10	14.2105
ENSG00000162894	FCMR	2.1490	4.84E-11	2.35E-10	14.2093
ENSG00000129048	ACKR4	1.4126	4.89E-11	2.37E-10	14.1987
ENSG00000249884	RNF103-CHMP3	1.2459	4.92E-11	2.39E-10	14.1927
ENSG00000147481	SNTG1	1.1559	4.99E-11	2.42E-10	14.1795
ENSG00000158169	FANCC	1.2920	5.01E-11	2.43E-10	14.1756
ENSG00000185052	SLC24A3	0.6257	5.05E-11	2.45E-10	14.1684
ENSG00000179299	NSUN7	1.5633	5.27E-11	2.56E-10	14.1256
ENSG00000131966	ACTR10	1.2735	5.28E-11	2.56E-10	14.1250

Gene ID	Gene Symbol	FC	P.Value	adj.P.Val	B
ENSG00000167094	TTC16	1.0707	5.34E-11	2.59E-10	14.1127
ENSG00000154144	TBRG1	1.3151	5.46E-11	2.65E-10	14.0912
ENSG00000127922	SEM1	0.7678	5.52E-11	2.67E-10	14.0807
ENSG00000179397	CATSPERE	1.2512	5.54E-11	2.68E-10	14.0772
ENSG00000153898	MCOLN2	1.2988	5.57E-11	2.70E-10	14.0713
ENSG00000170873	MTSS1	2.0291	5.62E-11	2.72E-10	14.0629
ENSG00000143412	ANXA9	1.3257	5.68E-11	2.75E-10	14.0525
ENSG00000107954	NEURL1	1.4223	5.68E-11	2.75E-10	14.0520
ENSG00000072422	RHOBTB1	1.6480	5.74E-11	2.78E-10	14.0420
ENSG00000166455	C16orf46	1.1758	5.79E-11	2.80E-10	14.0333
ENSG00000116459	ATP5PB	0.7648	5.80E-11	2.80E-10	14.0318
ENSG00000100906	NFKBIA	1.8952	5.83E-11	2.82E-10	14.0272
ENSG00000174498	IGDCC3	1.1029	5.97E-11	2.88E-10	14.0040
ENSG00000148158	SNX30	1.3715	6.00E-11	2.90E-10	13.9998
ENSG00000134243	SORT1	1.8597	6.03E-11	2.91E-10	13.9935
ENSG00000139626	ITGB7	2.3961	6.05E-11	2.92E-10	13.9909
ENSG00000182325	FBXL6	1.3183	6.10E-11	2.94E-10	13.9834
ENSG00000028310	BRD9	1.2431	6.13E-11	2.96E-10	13.9774
ENSG00000108557	RAI1	1.3219	6.20E-11	2.99E-10	13.9664
ENSG00000167992	VWCE	1.4198	6.25E-11	3.01E-10	13.9591
ENSG00000138079	SLC3A1	1.1308	6.26E-11	3.02E-10	13.9582
ENSG00000122824	NUDT10	1.1761	6.26E-11	3.02E-10	13.9579
ENSG00000102003	SYP	1.2479	6.32E-11	3.04E-10	13.9488
ENSG00000134086	VHL	1.3177	6.42E-11	3.09E-10	13.9325
ENSG00000240849	PEDS1	1.2839	6.45E-11	3.10E-10	13.9287
ENSG00000177363	LRRN4CL	1.0723	6.45E-11	3.11E-10	13.9278
ENSG00000196705	ZNF431	1.3108	6.48E-11	3.12E-10	13.9239
ENSG00000167749	KLK4	1.2578	6.52E-11	3.14E-10	13.9180
ENSG00000091592	NLRP1	1.4798	6.53E-11	3.14E-10	13.9166
ENSG00000100934	SEC23A	1.2680	6.58E-11	3.16E-10	13.9092
ENSG00000185189	NRBP2	1.5004	6.60E-11	3.17E-10	13.9065
ENSG00000135164	DMTF1	1.3487	6.60E-11	3.17E-10	13.9061
ENSG00000241258	CRCP	1.2415	6.64E-11	3.19E-10	13.9003
ENSG00000100814	CCNB1IP1	1.4457	6.70E-11	3.22E-10	13.8919
ENSG00000125351	UPF3B	1.3197	6.70E-11	3.22E-10	13.8906
ENSG00000021776	AQR	1.2248	6.72E-11	3.23E-10	13.8886
ENSG00000160294	MCM3AP	1.3449	6.84E-11	3.28E-10	13.8714
ENSG00000164620	RELL2	0.6323	6.86E-11	3.29E-10	13.8679
ENSG00000163798	SLC4A1AP	1.2423	6.87E-11	3.30E-10	13.8661
ENSG00000232388	SMIM26	0.8116	6.94E-11	3.33E-10	13.8574
ENSG00000186767	SPIN4	1.3444	6.97E-11	3.34E-10	13.8519
ENSG00000168522	FNTA	1.2481	7.08E-11	3.39E-10	13.8377
ENSG00000198554	WDHD1	1.4108	7.13E-11	3.42E-10	13.8303
ENSG00000163590	PPM1L	1.3510	7.37E-11	3.53E-10	13.7974
ENSG00000067057	PFKP	1.6622	7.50E-11	3.59E-10	13.7815
ENSG00000044115	CTNNA1	1.2745	7.53E-11	3.61E-10	13.7773
ENSG00000158321	AUTS2	1.5887	7.56E-11	3.62E-10	13.7733
ENSG00000171766	GATM	0.5632	7.58E-11	3.63E-10	13.7700
ENSG00000005238	FAM214B	1.3879	7.61E-11	3.64E-10	13.7672
ENSG00000130340	SNX9	2.2696	7.77E-11	3.72E-10	13.7465
ENSG00000186625	KATNA1	1.2472	7.78E-11	3.72E-10	13.7453
ENSG00000135912	TTLL4	1.3631	7.89E-11	3.77E-10	13.7317
ENSG00000100243	CYB5R3	1.3188	7.89E-11	3.77E-10	13.7314
ENSG00000149925	ALDOA	1.2643	7.89E-11	3.77E-10	13.7312

Gene ID	Gene Symbol	FC	P.Value	adj.P.Val	B
ENSG00000064726	BTBD1	1.2133	7.93E-11	3.79E-10	13.7269
ENSG00000182177	ASB18	1.0750	8.01E-11	3.83E-10	13.7161
ENSG00000107669	ATE1	1.2877	8.07E-11	3.85E-10	13.7099
ENSG00000162222	TTC9C	0.6736	8.07E-11	3.85E-10	13.7093
ENSG00000160117	ANKLE1	1.3413	8.09E-11	3.86E-10	13.7073
ENSG00000196187	TMEM63A	1.5240	8.13E-11	3.88E-10	13.7021
ENSG00000067798	NAV3	1.3635	8.21E-11	3.92E-10	13.6929
ENSG00000255423	EBLN2	1.3115	8.29E-11	3.95E-10	13.6831
ENSG00000106211	HSPB1	1.8397	8.35E-11	3.98E-10	13.6762
ENSG00000118363	SPCS2	0.7062	8.39E-11	4.00E-10	13.6718
ENSG00000144810	COL8A1	1.2328	8.39E-11	4.00E-10	13.6711
ENSG00000158623	COPG2	0.6623	8.39E-11	4.00E-10	13.6708
ENSG00000180316	PNPLA1	1.1392	8.41E-11	4.01E-10	13.6692
ENSG00000183580	FBXL7	1.5051	8.44E-11	4.02E-10	13.6651
ENSG00000149646	CNBD2	1.0654	8.56E-11	4.07E-10	13.6522
ENSG00000139793	MBNL2	1.4891	8.62E-11	4.10E-10	13.6452
ENSG00000093009	CDC45	1.6324	8.66E-11	4.12E-10	13.6399
ENSG00000097033	SH3GLB1	1.2808	8.66E-11	4.12E-10	13.6402
ENSG00000164049	FBXW12	1.1109	8.66E-11	4.12E-10	13.6401
ENSG00000143376	SNX27	1.3032	8.69E-11	4.13E-10	13.6369
ENSG00000204681	GABBR1	1.9250	8.70E-11	4.14E-10	13.6359
ENSG00000187735	TCEA1	1.3110	8.74E-11	4.15E-10	13.6316
ENSG00000160613	PCSK7	1.3191	8.77E-11	4.17E-10	13.6275
ENSG00000196950	SLC39A10	1.4484	8.81E-11	4.19E-10	13.6234
ENSG00000189233	NUGGC	1.4169	8.84E-11	4.20E-10	13.6206
ENSG00000182809	CRIP2	1.5713	9.03E-11	4.29E-10	13.5995
ENSG00000124507	PACSIN1	1.7827	9.07E-11	4.30E-10	13.5955
ENSG00000127947	PTPN12	1.3075	9.07E-11	4.31E-10	13.5947
ENSG00000110318	CEP126	1.4890	9.15E-11	4.34E-10	13.5870
ENSG00000172955	ADH6	1.3183	9.17E-11	4.35E-10	13.5840
ENSG00000126461	SCAF1	1.2792	9.29E-11	4.40E-10	13.5721
ENSG00000152684	PELO	1.3043	9.37E-11	4.44E-10	13.5630
ENSG00000239388	ASB14	1.2602	9.47E-11	4.49E-10	13.5530
ENSG00000152952	PLOD2	2.2272	9.48E-11	4.49E-10	13.5518
ENSG00000214711	CAPN14	1.4514	9.50E-11	4.50E-10	13.5500
ENSG00000105612	DNASE2	0.6810	9.52E-11	4.51E-10	13.5480
ENSG00000167100	SAMD14	1.1717	9.52E-11	4.51E-10	13.5482
ENSG00000142961	MOB3C	0.7220	9.53E-11	4.51E-10	13.5463
ENSG00000138075	ABCG5	1.0911	9.75E-11	4.61E-10	13.5244
ENSG00000174917	MICOS13	0.8054	9.84E-11	4.66E-10	13.5152
ENSG00000163093	BBS5	1.3087	9.87E-11	4.67E-10	13.5129
ENSG00000064309	CDON	1.2052	1.00E-10	4.74E-10	13.4983
ENSG00000179889	PDXDC1	1.3508	1.01E-10	4.79E-10	13.4873
ENSG00000131495	NDUFA2	0.8056	1.01E-10	4.79E-10	13.4859
ENSG00000204576	PRR3	1.2717	1.02E-10	4.80E-10	13.4846
ENSG00000129194	SOX15	1.2115	1.02E-10	4.84E-10	13.4769
ENSG00000168003	SLC3A2	1.3756	1.03E-10	4.87E-10	13.4705
ENSG00000126768	TIMM17B	1.2201	1.04E-10	4.89E-10	13.4653
ENSG00000179134	SAMD4B	1.2654	1.04E-10	4.90E-10	13.4623
ENSG00000128641	MYO1B	1.7615	1.04E-10	4.92E-10	13.4584
ENSG00000139354	GAS2L3	1.2700	1.04E-10	4.92E-10	13.4584
ENSG00000137804	NUSAP1	1.6966	1.05E-10	4.93E-10	13.4561
ENSG00000107897	ACBD5	1.2567	1.05E-10	4.94E-10	13.4535
ENSG00000167332	OR51E2	1.0366	1.06E-10	4.99E-10	13.4436

Gene ID	Gene Symbol	FC	P.Value	adj.P.Val	B
ENSG00000144619	CNTN4	0.6786	1.06E-10	5.01E-10	13.4393
ENSG00000197930	ERO1A	1.2925	1.08E-10	5.07E-10	13.4285
ENSG00000071889	FAM3A	1.3326	1.08E-10	5.10E-10	13.4231
ENSG00000130643	CALY	1.1897	1.08E-10	5.10E-10	13.4227
ENSG00000188739	RBM34	1.3054	1.09E-10	5.12E-10	13.4183
ENSG00000143499	SMYD2	1.3553	1.09E-10	5.15E-10	13.4114
ENSG00000133477	FAM83F	1.0456	1.11E-10	5.21E-10	13.4008
ENSG00000142945	KIF2C	1.5111	1.11E-10	5.22E-10	13.3977
ENSG00000132024	CC2D1A	1.3019	1.14E-10	5.34E-10	13.3750
ENSG00000069020	MAST4	1.4896	1.14E-10	5.36E-10	13.3716
ENSG00000176383	B3GNT4	1.2534	1.14E-10	5.38E-10	13.3682
ENSG00000113240	CLK4	1.3291	1.15E-10	5.40E-10	13.3643
ENSG00000198570	RD3	1.0608	1.15E-10	5.42E-10	13.3604
ENSG00000172167	MTBP	1.3314	1.16E-10	5.45E-10	13.3552
ENSG00000172985	SH3RF3	2.0961	1.18E-10	5.54E-10	13.3384
ENSG00000169194	IL13	1.2927	1.19E-10	5.57E-10	13.3326
ENSG00000160949	TONSL	1.4252	1.20E-10	5.64E-10	13.3212
ENSG00000140945	CDH13	1.1336	1.24E-10	5.81E-10	13.2917
ENSG00000196666	FAM180B	1.0993	1.24E-10	5.81E-10	13.2907
ENSG00000196335	STK31	1.2670	1.25E-10	5.85E-10	13.2832
ENSG00000089685	BIRC5	1.6082	1.26E-10	5.92E-10	13.2720
ENSG00000165633	VSTM4	1.0986	1.27E-10	5.95E-10	13.2671
ENSG00000175556	LONRF3	1.3206	1.27E-10	5.96E-10	13.2643
ENSG00000141447	OSBPL1A	1.7953	1.28E-10	5.98E-10	13.2617
ENSG00000242247	ARFGAP3	1.2801	1.28E-10	6.01E-10	13.2561
ENSG00000169122	FAM110B	1.2840	1.29E-10	6.02E-10	13.2538
ENSG00000069869	NEDD4	1.4156	1.29E-10	6.04E-10	13.2511
ENSG00000163682	RPL9	0.6822	1.29E-10	6.04E-10	13.2512
ENSG00000170191	NANP	1.2170	1.30E-10	6.07E-10	13.2453
ENSG00000004700	RECQL	1.3458	1.31E-10	6.13E-10	13.2360
ENSG00000172243	CLEC7A	2.1881	1.31E-10	6.13E-10	13.2349
ENSG00000128463	EMC4	0.8300	1.33E-10	6.23E-10	13.2194
ENSG00000179364	PACS2	1.3488	1.38E-10	6.44E-10	13.1875
ENSG00000204843	DCTN1	1.2841	1.38E-10	6.44E-10	13.1864
ENSG00000198947	DMD	1.3888	1.38E-10	6.47E-10	13.1814
ENSG00000104447	TRPS1	1.4272	1.42E-10	6.62E-10	13.1597
ENSG00000074590	NUAK1	1.6878	1.44E-10	6.71E-10	13.1455
ENSG00000132254	ARFIP2	1.2550	1.44E-10	6.71E-10	13.1448
ENSG00000220008	LINGO3	1.2167	1.44E-10	6.71E-10	13.1449
ENSG00000144381	HSPD1	1.3087	1.44E-10	6.72E-10	13.1434
ENSG00000174827	PDZK1	1.2603	1.45E-10	6.77E-10	13.1356
ENSG00000183779	ZNF703	1.3553	1.45E-10	6.78E-10	13.1339
ENSG00000103005	USB1	1.2924	1.46E-10	6.80E-10	13.1310
ENSG00000163357	DCST1	1.1778	1.46E-10	6.80E-10	13.1309
ENSG00000105398	SULT2A1	1.0513	1.48E-10	6.90E-10	13.1165
ENSG00000101844	ATG4A	1.2476	1.50E-10	6.98E-10	13.1047
ENSG00000144868	TMEM108	0.5879	1.50E-10	7.01E-10	13.1007
ENSG00000006712	PAF1	1.2637	1.51E-10	7.05E-10	13.0949
ENSG00000107036	RIC1	1.2961	1.51E-10	7.05E-10	13.0951
ENSG00000080822	CLDND1	1.3573	1.52E-10	7.07E-10	13.0918
ENSG00000198783	ZNF830	1.2983	1.52E-10	7.07E-10	13.0910
ENSG00000049541	RFC2	1.3393	1.52E-10	7.08E-10	13.0898
ENSG00000188542	DUSP28	1.2307	1.53E-10	7.13E-10	13.0825
ENSG00000215595	C20orf202	0.8631	1.54E-10	7.16E-10	13.0788

Gene ID	Gene Symbol	FC	P.Value	adj.P.Val	B
ENSG00000107554	DNMBP	1.4752	1.55E-10	7.19E-10	13.0739
ENSG00000064547	LPAR2	1.5524	1.55E-10	7.19E-10	13.0733
ENSG00000138813	C4orf17	1.1083	1.55E-10	7.21E-10	13.0704
ENSG00000206260	PRR23A	1.0363	1.56E-10	7.25E-10	13.0647
ENSG00000180776	ZDHHC20	1.3425	1.58E-10	7.33E-10	13.0540
ENSG00000137563	GGH	1.7740	1.58E-10	7.34E-10	13.0524
ENSG00000198830	HMGN2	1.3245	1.58E-10	7.35E-10	13.0511
ENSG00000108669	CYTH1	1.5755	1.60E-10	7.45E-10	13.0375
ENSG00000178026	LRRC75B	1.4845	1.61E-10	7.47E-10	13.0349
ENSG00000173894	CBX2	1.2620	1.61E-10	7.48E-10	13.0326
ENSG00000131148	EMC8	1.2667	1.62E-10	7.51E-10	13.0294
ENSG00000139597	N4BP2L1	1.4699	1.62E-10	7.52E-10	13.0275
ENSG00000011260	UTP18	1.2182	1.62E-10	7.53E-10	13.0264
ENSG00000172339	ALG14	0.7261	1.62E-10	7.53E-10	13.0257
ENSG00000112304	ACOT13	0.6976	1.63E-10	7.54E-10	13.0235
ENSG00000076067	RBMS2	1.2891	1.63E-10	7.55E-10	13.0224
ENSG00000138071	ACTR2	1.2693	1.63E-10	7.56E-10	13.0209
ENSG00000005486	RHBDD2	1.3333	1.63E-10	7.57E-10	13.0194
ENSG00000161010	MRNIP	1.3721	1.64E-10	7.60E-10	13.0157
ENSG00000134461	ANKRD16	0.6253	1.66E-10	7.68E-10	13.0047
ENSG00000248592	STIMATE-MUSTN1	1.3519	1.67E-10	7.73E-10	12.9985
ENSG00000134109	EDEM1	1.4204	1.68E-10	7.78E-10	12.9913
ENSG00000072609	CHFR	1.5126	1.70E-10	7.85E-10	12.9823
ENSG00000253729	PRKDC	1.3275	1.71E-10	7.89E-10	12.9776
ENSG00000010810	FYN	1.9202	1.71E-10	7.92E-10	12.9742
ENSG00000171055	FEZ2	1.2602	1.71E-10	7.93E-10	12.9728
ENSG00000196110	ZNF699	1.3409	1.72E-10	7.94E-10	12.9705
ENSG00000132688	NES	3.0002	1.74E-10	8.04E-10	12.9584
ENSG00000244754	N4BP2L2	0.7015	1.75E-10	8.06E-10	12.9556
ENSG00000109906	ZBTB16	1.5745	1.75E-10	8.07E-10	12.9538
ENSG00000168116	KIAA1586	1.3140	1.75E-10	8.08E-10	12.9523
ENSG00000187268	FAM9C	1.1336	1.75E-10	8.10E-10	12.9502
ENSG00000092108	SCFD1	1.2498	1.76E-10	8.10E-10	12.9496
ENSG00000102144	PGK1	1.2518	1.76E-10	8.13E-10	12.9463
ENSG00000250317	SMIM20	0.7613	1.77E-10	8.15E-10	12.9438
ENSG00000080603	SRCAP	1.3742	1.78E-10	8.19E-10	12.9376
ENSG00000172771	EFCAB12	1.2140	1.78E-10	8.19E-10	12.9376
ENSG00000161888	SPC24	1.4973	1.78E-10	8.22E-10	12.9344
ENSG00000130032	PRRG3	1.0511	1.79E-10	8.23E-10	12.9334
ENSG00000104626	ERI1	1.4168	1.79E-10	8.25E-10	12.9298
ENSG00000187955	COL14A1	1.2587	1.80E-10	8.28E-10	12.9262
ENSG00000168778	TCTN2	1.3596	1.81E-10	8.34E-10	12.9186
ENSG00000178971	CTC1	1.4457	1.83E-10	8.41E-10	12.9110
ENSG00000169495	HTRA4	1.1587	1.89E-10	8.71E-10	12.8761
ENSG00000143390	RFX5	1.3243	1.90E-10	8.74E-10	12.8730
ENSG00000060138	YBX3	3.1365	1.90E-10	8.75E-10	12.8709
ENSG00000155545	MIER3	1.3330	1.91E-10	8.80E-10	12.8660
ENSG00000139842	CUL4A	1.2755	1.94E-10	8.91E-10	12.8528
ENSG00000237353	PATE4	1.0890	1.96E-10	9.01E-10	12.8420
ENSG00000144747	TMF1	1.3458	1.96E-10	9.03E-10	12.8397
ENSG00000146373	RNF217	1.2783	1.97E-10	9.03E-10	12.8394
ENSG00000178950	GAK	1.2993	1.97E-10	9.04E-10	12.8377
ENSG00000137411	VARS2	1.3344	1.97E-10	9.05E-10	12.8365
ENSG00000198081	ZBTB14	1.3681	1.97E-10	9.05E-10	12.8361

Gene ID	Gene Symbol	FC	P.Value	adj.P.Val	B
ENSG00000188305	PEAK3	1.2775	1.99E-10	9.13E-10	12.8274
ENSG00000167748	KLK1	1.4686	1.99E-10	9.14E-10	12.8265
ENSG00000127980	PEX1	1.2978	2.00E-10	9.16E-10	12.8236
ENSG00000196670	ZFP62	0.7264	2.01E-10	9.20E-10	12.8198
ENSG00000197785	ATAD3A	1.3076	2.03E-10	9.30E-10	12.8086
ENSG00000114999	TTL	1.3149	2.04E-10	9.34E-10	12.8038
ENSG00000204385	SLC44A4	1.1375	2.05E-10	9.39E-10	12.7989
ENSG00000173113	TRMT112	0.7845	2.05E-10	9.40E-10	12.7974
ENSG00000132141	CCT6B	1.3308	2.08E-10	9.54E-10	12.7830
ENSG00000115526	CHST10	1.5240	2.09E-10	9.56E-10	12.7807
ENSG00000160796	NBEAL2	1.4384	2.09E-10	9.57E-10	12.7791
ENSG00000148488	ST8SIA6	1.1042	2.09E-10	9.58E-10	12.7784
ENSG00000180901	KCTD2	1.2678	2.10E-10	9.63E-10	12.7732
ENSG00000070729	CNGB1	1.1666	2.13E-10	9.73E-10	12.7627
ENSG00000105327	BBC3	1.4366	2.17E-10	9.94E-10	12.7417
ENSG00000239779	WBP1	1.2290	2.18E-10	9.95E-10	12.7399
ENSG00000183092	BEGAIN	1.3450	2.19E-10	1.00E-09	12.7337
ENSG00000107077	KDM4C	1.2295	2.20E-10	1.01E-09	12.7290
ENSG00000189241	TSPYL1	1.3010	2.21E-10	1.01E-09	12.7252
ENSG00000131459	GFPT2	1.0916	2.22E-10	1.01E-09	12.7207
ENSG00000163728	TTC14	0.6853	2.26E-10	1.03E-09	12.7017
ENSG00000262814	MRPL12	0.7107	2.29E-10	1.05E-09	12.6900
ENSG00000122012	SV2C	1.5937	2.30E-10	1.05E-09	12.6845
ENSG00000122644	ARL4A	1.6155	2.32E-10	1.06E-09	12.6785
ENSG00000134049	IER3IP1	1.2806	2.33E-10	1.06E-09	12.6741
ENSG00000166833	NAV2	1.6084	2.33E-10	1.06E-09	12.6718
ENSG00000119820	YIPF4	0.6611	2.35E-10	1.07E-09	12.6669
ENSG00000160194	NDUFV3	0.7228	2.35E-10	1.07E-09	12.6633
ENSG00000141556	TBCD	1.4155	2.36E-10	1.08E-09	12.6610
ENSG00000102312	PORCN	1.2459	2.37E-10	1.08E-09	12.6582
ENSG00000132334	PTPRE	1.5785	2.37E-10	1.08E-09	12.6551
ENSG00000131471	AOC3	1.4314	2.39E-10	1.09E-09	12.6475
ENSG00000007545	CRAMP1	1.3373	2.40E-10	1.09E-09	12.6455
ENSG00000139133	ALG10	1.3703	2.42E-10	1.10E-09	12.6364
ENSG00000185298	CCDC137	1.3363	2.42E-10	1.10E-09	12.6362
ENSG00000168014	C2CD3	1.2972	2.44E-10	1.11E-09	12.6298
ENSG00000167914	GSDMA	1.1235	2.45E-10	1.12E-09	12.6224
ENSG00000136603	SKIL	1.3556	2.46E-10	1.12E-09	12.6186
ENSG00000088833	NSFL1C	1.2984	2.49E-10	1.13E-09	12.6081
ENSG00000177427	MIEF2	1.3210	2.50E-10	1.14E-09	12.6057
ENSG00000100288	CHKB	1.3490	2.50E-10	1.14E-09	12.6041
ENSG00000174945	AMZ1	1.1329	2.51E-10	1.14E-09	12.6017
ENSG00000215305	VPS16	1.2697	2.51E-10	1.14E-09	12.6001
ENSG00000136982	DSCC1	1.4287	2.52E-10	1.14E-09	12.5984
ENSG00000240694	PNMA2	1.1041	2.52E-10	1.14E-09	12.5979
ENSG00000170779	CDCA4	1.3563	2.52E-10	1.14E-09	12.5971
ENSG00000196083	IL1RAP	1.3472	2.54E-10	1.15E-09	12.5882
ENSG00000154358	OBSCN	1.7574	2.58E-10	1.17E-09	12.5724
ENSG00000112078	KCTD20	1.2753	2.58E-10	1.17E-09	12.5721
ENSG00000133313	CNDP2	1.3797	2.60E-10	1.18E-09	12.5658
ENSG00000173258	ZNF483	1.3116	2.61E-10	1.18E-09	12.5641
ENSG00000165868	HSPA12A	1.2040	2.61E-10	1.18E-09	12.5636
ENSG00000086848	ALG9	1.2653	2.62E-10	1.19E-09	12.5587
ENSG00000103174	NAGPA	1.3398	2.65E-10	1.20E-09	12.5475

Gene ID	Gene Symbol	FC	P.Value	adj.P.Val	B
ENSG00000114354	TFG	1.2180	2.66E-10	1.21E-09	12.5438
ENSG00000181852	RNF41	1.2797	2.68E-10	1.21E-09	12.5365
ENSG00000089041	P2RX7	1.6050	2.69E-10	1.22E-09	12.5314
ENSG00000177990	DPY19L2	1.5603	2.73E-10	1.24E-09	12.5179
ENSG00000204272	NBDY	1.2588	2.73E-10	1.24E-09	12.5180
ENSG00000106077	ABHD11	1.5006	2.73E-10	1.24E-09	12.5167
ENSG00000014123	UFL1	1.3263	2.74E-10	1.24E-09	12.5161
ENSG00000248483	POU5F2	1.1193	2.76E-10	1.25E-09	12.5093
ENSG00000181163	NPM1	0.7424	2.76E-10	1.25E-09	12.5088
ENSG00000103168	TAF1C	1.3820	2.76E-10	1.25E-09	12.5064
ENSG00000254206	NPIPB11	1.2892	2.78E-10	1.26E-09	12.4995
ENSG00000143157	POGK	1.4135	2.79E-10	1.26E-09	12.4957
ENSG00000070159	PTPN3	1.2889	2.80E-10	1.26E-09	12.4944
ENSG00000159593	NAE1	1.2657	2.83E-10	1.28E-09	12.4837
ENSG00000011332	DPF1	1.1949	2.85E-10	1.29E-09	12.4759
ENSG00000198795	ZNF521	1.3456	2.87E-10	1.30E-09	12.4692
ENSG00000196646	ZNF136	1.3072	2.90E-10	1.31E-09	12.4597
ENSG00000183386	FHL3	1.4169	2.91E-10	1.31E-09	12.4571
ENSG00000162882	HAAO	1.3859	2.93E-10	1.32E-09	12.4511
ENSG00000153822	KCNJ16	1.2053	2.94E-10	1.33E-09	12.4462
ENSG00000151715	TMEM45B	1.1151	2.95E-10	1.33E-09	12.4423
ENSG00000163453	IGFBP7	1.9168	3.00E-10	1.35E-09	12.4249
ENSG00000146243	IRAK1BP1	1.6622	3.04E-10	1.37E-09	12.4127
ENSG00000063244	U2AF2	1.2095	3.08E-10	1.39E-09	12.4009
ENSG00000148396	SEC16A	1.2626	3.12E-10	1.40E-09	12.3883
ENSG00000134352	IL6ST	1.5113	3.12E-10	1.41E-09	12.3870
ENSG00000100302	RASD2	1.0452	3.14E-10	1.41E-09	12.3830
ENSG00000108828	VAT1	1.4015	3.14E-10	1.41E-09	12.3818
ENSG00000107816	LZTS2	1.3553	3.15E-10	1.42E-09	12.3793
ENSG00000186994	KANK3	1.2703	3.15E-10	1.42E-09	12.3791
ENSG00000157578	LCA5L	1.2482	3.16E-10	1.42E-09	12.3761
ENSG00000156140	ADAMTS3	1.1686	3.18E-10	1.43E-09	12.3685
ENSG00000121898	CPXM2	1.1136	3.25E-10	1.46E-09	12.3488
ENSG00000085465	OVGP1	1.4452	3.26E-10	1.46E-09	12.3456
ENSG00000154734	ADAMTS1	1.6674	3.26E-10	1.47E-09	12.3443
ENSG00000162545	CAMK2N1	1.1033	3.27E-10	1.47E-09	12.3412
ENSG00000167195	GOLGA6C	1.1130	3.29E-10	1.48E-09	12.3372
ENSG00000166509	CLEC3A	1.0870	3.30E-10	1.48E-09	12.3341
ENSG00000073969	NSF	1.2853	3.30E-10	1.48E-09	12.3336
ENSG00000229117	RPL41	0.7947	3.30E-10	1.48E-09	12.3329
ENSG00000177045	SIX5	0.6441	3.31E-10	1.48E-09	12.3309
ENSG00000113657	DPYSL3	1.1848	3.31E-10	1.48E-09	12.3306
ENSG00000163528	CHCHD4	0.7247	3.32E-10	1.49E-09	12.3261
ENSG00000157214	STEAP2	1.1453	3.33E-10	1.49E-09	12.3237
ENSG00000073670	ADAM11	1.1927	3.37E-10	1.51E-09	12.3136
ENSG00000151148	UBE3B	1.2650	3.37E-10	1.51E-09	12.3115
ENSG00000165494	PCF11	1.3532	3.38E-10	1.52E-09	12.3090
ENSG00000198825	INPP5F	1.3755	3.40E-10	1.52E-09	12.3049
ENSG00000169347	GP2	1.0655	3.45E-10	1.54E-09	12.2912
ENSG00000196267	ZNF836	1.3407	3.48E-10	1.56E-09	12.2815
ENSG00000196531	NACA	0.8137	3.48E-10	1.56E-09	12.2814
ENSG00000109452	INPP4B	1.3243	3.51E-10	1.57E-09	12.2734
ENSG00000065717	TLE2	1.2543	3.52E-10	1.57E-09	12.2711
ENSG00000117632	STMN1	1.7674	3.54E-10	1.58E-09	12.2646

Gene ID	Gene Symbol	FC	P.Value	adj.P.Val	B
ENSG00000135124	P2RX4	1.3008	3.55E-10	1.59E-09	12.2611
ENSG00000086062	B4GALT1	1.6975	3.61E-10	1.61E-09	12.2455
ENSG00000134900	TPP2	1.3386	3.64E-10	1.62E-09	12.2389
ENSG00000134769	DTNA	1.6414	3.65E-10	1.63E-09	12.2359
ENSG00000115163	CENPA	1.5356	3.66E-10	1.64E-09	12.2318
ENSG00000109079	TNFAIP1	1.3302	3.68E-10	1.64E-09	12.2268
ENSG00000068028	RASSF1	1.3018	3.70E-10	1.65E-09	12.2227
ENSG00000179826	MRGPRX3	1.0506	3.70E-10	1.65E-09	12.2226
ENSG00000147789	ZNF7	1.2326	3.70E-10	1.65E-09	12.2223
ENSG00000180720	CHRM4	1.2636	3.72E-10	1.66E-09	12.2162
ENSG00000204311	PJVK	1.3398	3.72E-10	1.66E-09	12.2163
ENSG00000082126	MPP4	1.1315	3.73E-10	1.66E-09	12.2138
ENSG00000138160	KIF11	1.5072	3.74E-10	1.67E-09	12.2119
ENSG00000117650	NEK2	1.5833	3.76E-10	1.68E-09	12.2053
ENSG00000161692	DBF4B	1.3159	3.76E-10	1.68E-09	12.2056
ENSG00000142655	PEX14	1.2295	3.78E-10	1.69E-09	12.1999
ENSG00000135521	LTV1	1.2858	3.79E-10	1.69E-09	12.1976
ENSG00000178127	NDUFV2	1.2488	3.82E-10	1.70E-09	12.1916
ENSG00000159658	EFCAB14	1.2683	3.87E-10	1.72E-09	12.1777
ENSG00000170322	NFRKB	1.3214	3.89E-10	1.73E-09	12.1740
ENSG00000135338	LCA5	1.3866	3.89E-10	1.73E-09	12.1725
ENSG00000101493	ZNF516	1.6286	3.91E-10	1.74E-09	12.1669
ENSG00000141524	TMC6	2.5097	4.02E-10	1.79E-09	12.1419
ENSG00000243279	PRAF2	1.4468	4.03E-10	1.79E-09	12.1387
ENSG00000125845	BMP2	1.1627	4.04E-10	1.79E-09	12.1367
ENSG00000165264	NDUFB6	0.7692	4.12E-10	1.83E-09	12.1160
ENSG00000147465	STAR	1.0888	4.14E-10	1.84E-09	12.1112
ENSG00000090339	ICAM1	1.6667	4.16E-10	1.85E-09	12.1063
ENSG00000137575	SDCBP	1.6139	4.19E-10	1.86E-09	12.1009
ENSG00000114331	ACAP2	1.2964	4.21E-10	1.87E-09	12.0953
ENSG00000001617	SEMA3F	1.2002	4.28E-10	1.90E-09	12.0799
ENSG00000131650	KREMEN2	1.2193	4.29E-10	1.90E-09	12.0770
ENSG00000185873	TMPRSS11B	1.1159	4.31E-10	1.91E-09	12.0728
ENSG00000163939	PBRM1	1.3107	4.34E-10	1.93E-09	12.0650
ENSG00000046647	GEMIN8	1.2460	4.35E-10	1.93E-09	12.0644
ENSG00000005194	CIAPIN1	1.2785	4.35E-10	1.93E-09	12.0632
ENSG00000184925	LCN12	1.5651	4.36E-10	1.93E-09	12.0611
ENSG00000013561	RNF14	0.7180	4.37E-10	1.94E-09	12.0586
ENSG00000174243	DDX23	1.2668	4.40E-10	1.95E-09	12.0518
ENSG00000164631	ZNF12	1.2377	4.50E-10	1.99E-09	12.0301
ENSG00000186310	NAP1L3	1.4768	4.50E-10	1.99E-09	12.0301
ENSG00000169188	APEX2	1.3246	4.55E-10	2.02E-09	12.0197
ENSG00000173338	KCNK7	1.4975	4.57E-10	2.02E-09	12.0158
ENSG00000124019	FAM124B	1.0890	4.61E-10	2.04E-09	12.0080
ENSG00000197557	TTC30A	1.3219	4.62E-10	2.04E-09	12.0053
ENSG00000007384	RHBDF1	1.3970	4.62E-10	2.05E-09	12.0042
ENSG00000022976	ZNF839	1.2854	4.63E-10	2.05E-09	12.0032
ENSG00000152454	ZNF256	1.4900	4.63E-10	2.05E-09	12.0019
ENSG00000158125	XDH	1.1803	4.64E-10	2.05E-09	12.0000
ENSG00000188000	OR7D2	1.1406	4.67E-10	2.07E-09	11.9935
ENSG00000088325	TPX2	1.6531	4.71E-10	2.08E-09	11.9853
ENSG00000084710	EFR3B	1.0569	4.72E-10	2.08E-09	11.9844
ENSG00000176809	LRRC37A3	1.3559	4.75E-10	2.10E-09	11.9775
ENSG00000010318	PHF7	1.3138	4.79E-10	2.11E-09	11.9702

Gene ID	Gene Symbol	FC	P.Value	adj.P.Val	B
ENSG00000105085	MED26	1.3666	4.80E-10	2.12E-09	11.9673
ENSG00000105467	SYNGR4	1.1970	4.83E-10	2.13E-09	11.9615
ENSG00000169758	TMEM266	1.1508	4.85E-10	2.14E-09	11.9577
ENSG00000198853	RUSC2	1.2856	4.85E-10	2.14E-09	11.9576
ENSG00000080802	CNOT4	1.2443	4.86E-10	2.14E-09	11.9550
ENSG00000166896	ATP23	1.3300	4.86E-10	2.15E-09	11.9546
ENSG00000198885	ITPRIPL1	1.5326	4.88E-10	2.15E-09	11.9507
ENSG00000008323	PLEKHG6	1.1248	4.90E-10	2.16E-09	11.9480
ENSG00000186185	KIF18B	1.5336	4.91E-10	2.16E-09	11.9464
ENSG00000101189	MRGBP	1.2469	4.91E-10	2.17E-09	11.9447
ENSG00000181788	SIAH2	1.2897	4.92E-10	2.17E-09	11.9430
ENSG00000142082	SIRT3	1.2731	4.96E-10	2.18E-09	11.9359
ENSG00000073711	PPP2R3A	1.1963	5.00E-10	2.20E-09	11.9282
ENSG00000221890	NPTXR	1.1064	5.05E-10	2.22E-09	11.9186
ENSG00000168159	RNF187	1.4694	5.05E-10	2.22E-09	11.9175
ENSG00000268480	NFILZ	1.0924	5.06E-10	2.23E-09	11.9156
ENSG00000088986	DYNLL1	0.6263	5.08E-10	2.23E-09	11.9125
ENSG00000067208	EVI5	1.2821	5.09E-10	2.24E-09	11.9097
ENSG00000134318	ROCK2	1.3919	5.11E-10	2.25E-09	11.9058
ENSG00000215186	GOLGA6B	1.1504	5.19E-10	2.28E-09	11.8911
ENSG00000158089	GALNT14	1.6331	5.25E-10	2.31E-09	11.8798
ENSG00000172428	COPS9	0.8210	5.27E-10	2.31E-09	11.8774
ENSG00000162909	CAPN2	1.6110	5.27E-10	2.32E-09	11.8763
ENSG00000119772	DNMT3A	1.4069	5.28E-10	2.32E-09	11.8749
ENSG00000174718	RESF1	1.3772	5.39E-10	2.37E-09	11.8544
ENSG00000162004	CCDC78	0.5355	5.40E-10	2.37E-09	11.8522
ENSG00000065320	NTN1	1.0773	5.41E-10	2.37E-09	11.8509
ENSG00000165480	SKA3	1.4353	5.41E-10	2.37E-09	11.8510
ENSG00000183671	CMKLR2	1.0443	5.41E-10	2.37E-09	11.8509
ENSG00000197077	KIAA1671	1.0867	5.49E-10	2.41E-09	11.8365
ENSG00000134013	LOXL2	1.4226	5.50E-10	2.41E-09	11.8343
ENSG00000089157	RPLP0	0.7811	5.51E-10	2.42E-09	11.8327
ENSG00000119242	CCDC92	1.3374	5.57E-10	2.44E-09	11.8227
ENSG00000135900	MRPL44	1.2552	5.64E-10	2.47E-09	11.8102
ENSG00000105887	MTPN	1.3106	5.69E-10	2.49E-09	11.8008
ENSG00000100060	MFNG	0.5452	5.73E-10	2.51E-09	11.7945
ENSG00000204186	ZDBF2	1.4603	5.74E-10	2.51E-09	11.7926
ENSG00000183172	SMDT1	0.7847	5.80E-10	2.54E-09	11.7837
ENSG00000161395	PGAP3	1.2696	5.83E-10	2.55E-09	11.7786
ENSG00000108298	RPL19	0.8000	5.87E-10	2.57E-09	11.7713
ENSG00000174123	TLR10	0.4914	5.90E-10	2.58E-09	11.7660
ENSG00000178409	BEND3	1.2103	5.92E-10	2.59E-09	11.7630
ENSG00000103326	CAPN15	1.2493	5.93E-10	2.59E-09	11.7609
ENSG00000167741	GGT6	1.1087	5.94E-10	2.60E-09	11.7599
ENSG00000173826	KCNH6	1.0440	5.94E-10	2.60E-09	11.7600
ENSG00000177710	SLC35G5	1.0716	5.96E-10	2.60E-09	11.7567
ENSG00000198286	CARD11	2.1981	5.96E-10	2.60E-09	11.7566
ENSG00000136933	RABEPK	0.7351	5.97E-10	2.61E-09	11.7555
ENSG00000064607	SUGP2	1.2825	5.98E-10	2.61E-09	11.7529
ENSG00000075073	TACR2	0.7930	6.01E-10	2.62E-09	11.7488
ENSG00000107819	SFXN3	1.4707	6.01E-10	2.62E-09	11.7487
ENSG00000196954	CASP4	0.7044	6.05E-10	2.64E-09	11.7414
ENSG00000198863	RUNDC1	1.2411	6.13E-10	2.67E-09	11.7294
ENSG00000204351	SKIV2L	1.2962	6.18E-10	2.69E-09	11.7213

Gene ID	Gene Symbol	FC	P.Value	adj.P.Val	B
ENSG00000197566	ZNF624	1.2250	6.19E-10	2.70E-09	11.7189
ENSG00000109684	CLNK	1.1235	6.20E-10	2.70E-09	11.7182
ENSG00000164535	DAGLB	1.2375	6.20E-10	2.70E-09	11.7173
ENSG00000167635	ZNF146	0.6890	6.27E-10	2.73E-09	11.7068
ENSG00000101350	KIF3B	1.2822	6.29E-10	2.74E-09	11.7035
ENSG00000178053	MLF1	1.9133	6.37E-10	2.77E-09	11.6914
ENSG00000197599	CCDC154	0.7434	6.42E-10	2.80E-09	11.6834
ENSG00000176887	SOX11	1.1699	6.44E-10	2.80E-09	11.6806
ENSG00000185800	DMWD	1.2397	6.50E-10	2.83E-09	11.6712
ENSG00000165091	TMC1	1.1755	6.51E-10	2.83E-09	11.6707
ENSG00000182050	MGAT4C	0.5247	6.51E-10	2.83E-09	11.6702
ENSG00000116266	STXBP3	1.2607	6.55E-10	2.85E-09	11.6641
ENSG00000012963	UBR7	1.3130	6.61E-10	2.88E-09	11.6547
ENSG00000173221	GLRX	0.6192	6.69E-10	2.91E-09	11.6430
ENSG00000175220	ARHGAP1	1.2640	6.72E-10	2.92E-09	11.6389
ENSG00000204899	MZT1	0.7168	6.92E-10	3.01E-09	11.6106
ENSG00000059588	TARBP1	1.6681	6.97E-10	3.03E-09	11.6042
ENSG00000104731	KLHDC4	1.3289	6.97E-10	3.03E-09	11.6042
ENSG00000113971	NPHP3	1.3338	6.99E-10	3.03E-09	11.6015
ENSG00000142549	IGLON5	1.0399	6.99E-10	3.03E-09	11.6016
ENSG00000166912	MTMR10	1.3140	6.99E-10	3.03E-09	11.6009
ENSG00000108107	RPL28	0.7485	7.05E-10	3.06E-09	11.5927
ENSG00000179564	LSMEM2	1.1090	7.07E-10	3.07E-09	11.5896
ENSG00000172365	OR5B2	0.9246	7.11E-10	3.09E-09	11.5837
ENSG00000135842	NIBAN1	1.7457	7.13E-10	3.09E-09	11.5820
ENSG00000167941	SOST	1.0229	7.18E-10	3.11E-09	11.5753
ENSG00000183726	TMEM50A	1.2084	7.22E-10	3.13E-09	11.5689
ENSG00000213465	ARL2	0.7377	7.22E-10	3.13E-09	11.5687
ENSG00000123416	TUBA1B	1.4381	7.29E-10	3.16E-09	11.5597
ENSG00000220201	ZGLP1	0.6907	7.30E-10	3.16E-09	11.5582
ENSG00000134962	KLB	1.0660	7.32E-10	3.17E-09	11.5557
ENSG00000135932	CAB39	1.2660	7.37E-10	3.19E-09	11.5496
ENSG00000197124	ZNF682	1.5113	7.37E-10	3.19E-09	11.5496
ENSG00000168070	MAJIN	1.3917	7.50E-10	3.24E-09	11.5327
ENSG00000130561	SAG	1.1333	7.50E-10	3.24E-09	11.5325
ENSG00000198835	GJC2	1.1129	7.51E-10	3.25E-09	11.5304
ENSG00000135632	SMYD5	1.3764	7.58E-10	3.28E-09	11.5214
ENSG00000127863	TNFRSF19	1.3396	7.61E-10	3.29E-09	11.5175
ENSG00000188822	CNR2	1.0894	7.63E-10	3.30E-09	11.5159
ENSG00000165810	BTNL9	1.5208	7.64E-10	3.30E-09	11.5137
ENSG00000102886	GDPD3	1.3428	7.67E-10	3.31E-09	11.5102
ENSG00000170906	NDUFA3	0.8146	7.67E-10	3.31E-09	11.5098
ENSG00000148824	MTG1	1.3721	7.68E-10	3.32E-09	11.5092
ENSG00000024422	EHD2	1.3240	7.70E-10	3.33E-09	11.5060
ENSG00000184924	PTRHD1	0.7829	7.77E-10	3.35E-09	11.4981
ENSG00000155749	FLACC1	1.2511	7.79E-10	3.36E-09	11.4955
ENSG00000136040	PLXNC1	1.4339	7.85E-10	3.39E-09	11.4873
ENSG00000163032	VSNL1	1.1058	7.97E-10	3.44E-09	11.4727
ENSG00000091542	ALKBH5	1.2653	8.01E-10	3.45E-09	11.4676
ENSG00000105829	BET1	0.7120	8.01E-10	3.45E-09	11.4677
ENSG00000131981	LGALS3	1.5562	8.03E-10	3.46E-09	11.4661
ENSG00000087338	GMCL1	1.3001	8.03E-10	3.46E-09	11.4651
ENSG00000174547	MRPL11	0.8045	8.08E-10	3.48E-09	11.4597
ENSG00000180116	C12orf40	1.0594	8.19E-10	3.53E-09	11.4461

Gene ID	Gene Symbol	FC	P.Value	adj.P.Val	B
ENSG00000137767	SQOR	0.7701	8.21E-10	3.53E-09	11.4446
ENSG00000167470	MIDN	1.6663	8.21E-10	3.53E-09	11.4439
ENSG00000167658	EEF2	0.7366	8.25E-10	3.55E-09	11.4391
ENSG00000139154	AEBP2	1.2738	8.26E-10	3.56E-09	11.4375
ENSG00000165084	C8orf34	1.1103	8.28E-10	3.56E-09	11.4353
ENSG00000177119	ANO6	1.3087	8.28E-10	3.56E-09	11.4354
ENSG00000185480	PARPBP	1.3822	8.38E-10	3.61E-09	11.4235
ENSG00000165233	CARD19	1.3585	8.49E-10	3.65E-09	11.4116
ENSG00000164647	STEAP1	1.5708	8.55E-10	3.68E-09	11.4044
ENSG00000054690	PLEKHH1	1.4218	8.56E-10	3.68E-09	11.4031
ENSG00000173848	NET1	1.5905	8.66E-10	3.72E-09	11.3916
ENSG00000131435	PDLIM4	1.2293	8.72E-10	3.74E-09	11.3853
ENSG00000173193	PARP14	1.5198	8.75E-10	3.76E-09	11.3816
ENSG00000162851	TFB2M	1.2971	8.78E-10	3.77E-09	11.3782
ENSG00000068001	HYAL2	0.6810	8.93E-10	3.83E-09	11.3622
ENSG00000228278	ORM2	1.3114	8.97E-10	3.85E-09	11.3573
ENSG00000163399	ATP1A1	1.3398	9.04E-10	3.88E-09	11.3503
ENSG00000075426	FOSL2	1.6860	9.20E-10	3.95E-09	11.3331
ENSG00000172175	MALT1	1.4227	9.40E-10	4.03E-09	11.3121
ENSG00000187535	IFT140	1.3663	9.45E-10	4.05E-09	11.3071
ENSG00000175137	SH3BP5L	1.3911	9.46E-10	4.05E-09	11.3060
ENSG00000103510	KAT8	1.2425	9.48E-10	4.06E-09	11.3038
ENSG00000102385	DRP2	1.0828	9.49E-10	4.07E-09	11.3022
ENSG00000104371	DKK4	1.0556	9.50E-10	4.07E-09	11.3017
ENSG00000156873	PHKG2	1.2566	9.54E-10	4.09E-09	11.2972
ENSG00000070018	LRP6	1.4235	9.63E-10	4.12E-09	11.2880
ENSG00000164056	SPRY1	1.7523	9.68E-10	4.14E-09	11.2834
ENSG00000107957	SH3PXD2A	1.4236	9.70E-10	4.15E-09	11.2809
ENSG00000125945	ZNF436	1.2231	9.71E-10	4.16E-09	11.2802
ENSG00000135424	ITGA7	1.4559	9.78E-10	4.19E-09	11.2730
ENSG00000166963	MAP1A	1.3234	9.89E-10	4.23E-09	11.2620
ENSG00000148300	REXO4	0.7706	9.91E-10	4.24E-09	11.2601
ENSG00000204604	ZNF468	1.4594	9.93E-10	4.24E-09	11.2590
ENSG00000130303	BST2	0.7399	1.01E-09	4.30E-09	11.2467
ENSG00000184967	NOC4L	1.2478	1.01E-09	4.30E-09	11.2460
ENSG00000101443	WFDC2	2.4728	1.01E-09	4.33E-09	11.2381
ENSG00000013573	DDX11	1.4432	1.02E-09	4.34E-09	11.2352
ENSG00000182963	GJC1	1.1145	1.03E-09	4.39E-09	11.2250
ENSG00000197976	AKAP17A	1.3676	1.03E-09	4.42E-09	11.2185
ENSG00000166557	TMED3	0.7454	1.04E-09	4.44E-09	11.2137
ENSG00000156411	ATP5MJ	0.8279	1.04E-09	4.46E-09	11.2092
ENSG00000166780	BMERB1	1.1850	1.06E-09	4.53E-09	11.1926
ENSG00000102931	ARL2BP	1.3008	1.07E-09	4.56E-09	11.1873
ENSG00000064601	CTSA	1.3814	1.07E-09	4.57E-09	11.1845
ENSG00000125629	INSIG2	1.3813	1.08E-09	4.60E-09	11.1770
ENSG00000108387	SEPTIN4	1.3449	1.10E-09	4.69E-09	11.1592
ENSG00000228486	C2orf92	1.4238	1.11E-09	4.73E-09	11.1502
ENSG00000123395	ATG101	1.2671	1.12E-09	4.78E-09	11.1389
ENSG00000126267	COX6B1	0.8217	1.14E-09	4.84E-09	11.1279
ENSG00000162739	SLAMF6	0.5354	1.14E-09	4.85E-09	11.1253
ENSG00000008256	CYTH3	1.4827	1.14E-09	4.85E-09	11.1249
ENSG00000084070	SMAP2	1.8105	1.15E-09	4.91E-09	11.1129
ENSG00000164794	KCNV1	1.0824	1.16E-09	4.93E-09	11.1087
ENSG00000179476	C14orf28	1.3106	1.16E-09	4.94E-09	11.1054

Gene ID	Gene Symbol	FC	P.Value	adj.P.Val	B
ENSG00000187531	SIRT7	1.2360	1.17E-09	4.97E-09	11.1003
ENSG00000186510	CLCNKA	1.6320	1.17E-09	4.98E-09	11.0987
ENSG00000101888	NXT2	1.4347	1.18E-09	5.00E-09	11.0927
ENSG00000105509	HAS1	1.0838	1.18E-09	5.01E-09	11.0917
ENSG00000147601	TERF1	1.2522	1.18E-09	5.02E-09	11.0897
ENSG00000056558	TRAF1	1.4278	1.18E-09	5.02E-09	11.0885
ENSG00000159352	PSMD4	1.3143	1.20E-09	5.11E-09	11.0712
ENSG00000126247	CAPNS1	1.2173	1.20E-09	5.11E-09	11.0706
ENSG00000171469	ZNF561	0.7434	1.20E-09	5.11E-09	11.0704
ENSG00000178028	DMAP1	1.2643	1.20E-09	5.11E-09	11.0701
ENSG00000163933	RFT1	0.7094	1.21E-09	5.13E-09	11.0667
ENSG00000125869	LAMP5	0.2507	1.21E-09	5.14E-09	11.0645
ENSG00000167011	NAT16	1.0567	1.21E-09	5.14E-09	11.0641
ENSG00000174579	MSL2	1.3046	1.22E-09	5.16E-09	11.0601
ENSG00000140527	WDR93	1.0818	1.23E-09	5.20E-09	11.0524
ENSG00000137561	TTPA	1.0796	1.24E-09	5.26E-09	11.0417
ENSG00000165322	ARHGAP12	1.3110	1.26E-09	5.33E-09	11.0288
ENSG00000139719	VPS33A	1.2780	1.27E-09	5.37E-09	11.0210
ENSG00000146857	STRA8	1.3104	1.27E-09	5.38E-09	11.0191
ENSG00000139344	AMDHD1	1.2933	1.27E-09	5.39E-09	11.0170
ENSG00000113369	ARRDC3	1.4651	1.28E-09	5.41E-09	11.0123
ENSG00000147488	ST18	1.1010	1.28E-09	5.42E-09	11.0107
ENSG00000100644	HIF1A	2.0647	1.29E-09	5.48E-09	10.9999
ENSG00000178460	MCMDC2	1.1176	1.30E-09	5.49E-09	10.9980
ENSG00000198157	HMGN5	1.6305	1.30E-09	5.49E-09	10.9974
ENSG00000112877	CEP72	1.3249	1.31E-09	5.54E-09	10.9882
ENSG00000124357	NAGK	1.3429	1.32E-09	5.58E-09	10.9812
ENSG00000140451	PIF1	0.5774	1.32E-09	5.58E-09	10.9805
ENSG00000125531	FNDC11	1.3081	1.32E-09	5.60E-09	10.9775
ENSG00000006638	TBXA2R	1.1295	1.33E-09	5.63E-09	10.9718
ENSG00000005448	WDR54	1.3374	1.34E-09	5.66E-09	10.9671
ENSG00000143033	MTF2	1.2971	1.36E-09	5.73E-09	10.9544
ENSG00000196368	NUDT11	1.4937	1.36E-09	5.75E-09	10.9512
ENSG00000198925	ATG9A	1.3631	1.37E-09	5.78E-09	10.9457
ENSG00000137814	HAUS2	1.2607	1.37E-09	5.79E-09	10.9437
ENSG00000167740	CYB5D2	0.7474	1.39E-09	5.88E-09	10.9287
ENSG00000197841	ZNF181	0.7029	1.40E-09	5.89E-09	10.9269
ENSG00000238083	LRRC37A2	1.3417	1.41E-09	5.93E-09	10.9202
ENSG00000171421	MRPL36	0.7831	1.42E-09	5.99E-09	10.9099
ENSG00000205531	NAP1L4	1.2539	1.43E-09	6.05E-09	10.9002
ENSG00000182326	C1S	1.3409	1.44E-09	6.07E-09	10.8962
ENSG00000132824	SERINC3	1.2679	1.45E-09	6.09E-09	10.8927
ENSG00000135898	GPR55	0.6296	1.45E-09	6.10E-09	10.8909
ENSG00000179152	TCAIM	0.7161	1.46E-09	6.16E-09	10.8815
ENSG00000164037	SLC9B1	1.1963	1.48E-09	6.23E-09	10.8707
ENSG00000105708	ZNF14	1.2945	1.48E-09	6.24E-09	10.8674
ENSG00000159461	AMFR	1.2924	1.50E-09	6.30E-09	10.8592
ENSG00000174343	CHRNA9	1.1115	1.50E-09	6.30E-09	10.8585
ENSG00000169992	NLGN2	1.3613	1.50E-09	6.30E-09	10.8580
ENSG00000159128	IFNGR2	1.3530	1.51E-09	6.34E-09	10.8517
ENSG00000175414	ARL10	1.3474	1.51E-09	6.36E-09	10.8490
ENSG00000170264	FAM161A	1.4205	1.52E-09	6.38E-09	10.8452
ENSG00000140961	OSGIN1	1.3647	1.52E-09	6.40E-09	10.8424
ENSG00000046653	GPM6B	1.4295	1.53E-09	6.42E-09	10.8394

Gene ID	Gene Symbol	FC	P.Value	adj.P.Val	B
ENSG00000119523	ALG2	0.7325	1.54E-09	6.45E-09	10.8338
ENSG00000100156	SLC16A8	1.3574	1.54E-09	6.47E-09	10.8304
ENSG00000213930	GALT	0.7119	1.55E-09	6.52E-09	10.8230
ENSG00000254901	BORCS8	0.7629	1.56E-09	6.57E-09	10.8158
ENSG00000181191	PJA1	1.3107	1.58E-09	6.62E-09	10.8078
ENSG00000206535	LNP1	0.7022	1.58E-09	6.64E-09	10.8053
ENSG00000134690	CDCA8	1.5105	1.59E-09	6.69E-09	10.7973
ENSG00000143382	ADAMTSL4	1.4003	1.60E-09	6.73E-09	10.7917
ENSG00000139173	TMEM117	1.2013	1.62E-09	6.80E-09	10.7804
ENSG00000131508	UBE2D2	1.2078	1.64E-09	6.89E-09	10.7671
ENSG00000196839	ADA	1.5028	1.67E-09	7.00E-09	10.7523
ENSG00000196526	AFAP1	1.2491	1.67E-09	7.01E-09	10.7509
ENSG00000110172	CHORDC1	1.2881	1.68E-09	7.03E-09	10.7471
ENSG00000135631	RAB11FIP5	1.6076	1.68E-09	7.03E-09	10.7472
ENSG00000161609	KASH5	1.2402	1.68E-09	7.03E-09	10.7474
ENSG00000144485	HES6	1.5276	1.68E-09	7.04E-09	10.7457
ENSG00000160202	CRYAA	0.7730	1.68E-09	7.04E-09	10.7458
ENSG00000152463	OLAH	1.3462	1.69E-09	7.07E-09	10.7408
ENSG00000104903	LYL1	0.6516	1.69E-09	7.07E-09	10.7404
ENSG00000174448	STARD6	1.1457	1.70E-09	7.10E-09	10.7361
ENSG00000100298	APOBEC3H	1.4381	1.71E-09	7.16E-09	10.7280
ENSG00000154438	ASZ1	1.1417	1.72E-09	7.19E-09	10.7240
ENSG00000101773	RBBP8	1.3467	1.73E-09	7.22E-09	10.7190
ENSG00000078053	AMPH	1.2368	1.73E-09	7.22E-09	10.7185
ENSG00000051180	RAD51	1.3941	1.73E-09	7.25E-09	10.7151
ENSG00000198752	CDC42BPB	1.4231	1.74E-09	7.29E-09	10.7097
ENSG00000137073	UBAP2	1.2488	1.74E-09	7.29E-09	10.7091
ENSG00000166569	CPLX4	1.1969	1.75E-09	7.30E-09	10.7070
ENSG00000196437	ZNF569	1.3642	1.77E-09	7.38E-09	10.6970
ENSG00000156011	PSD3	1.2096	1.78E-09	7.44E-09	10.6889
ENSG00000140022	STON2	1.5082	1.79E-09	7.49E-09	10.6816
ENSG00000151726	ACSL1	1.8165	1.80E-09	7.51E-09	10.6792
ENSG00000253797	UTP14C	0.6980	1.80E-09	7.52E-09	10.6779
ENSG00000023892	DEF6	1.2981	1.80E-09	7.52E-09	10.6769
ENSG00000256977	LIMS3	1.2175	1.80E-09	7.52E-09	10.6768
ENSG00000126005	MMP24OS	1.2459	1.81E-09	7.54E-09	10.6737
ENSG00000167751	KLK2	1.4707	1.85E-09	7.70E-09	10.6533
ENSG00000101084	RAB5IF	1.2020	1.85E-09	7.73E-09	10.6497
ENSG00000092931	MFSD11	1.2653	1.86E-09	7.76E-09	10.6458
ENSG00000185475	TMEM179B	0.7772	1.87E-09	7.80E-09	10.6401
ENSG00000077935	SMC1B	1.2106	1.88E-09	7.82E-09	10.6382
ENSG00000130222	GADD45G	1.8770	1.88E-09	7.82E-09	10.6377
ENSG00000166428	PLD4	2.6367	1.88E-09	7.84E-09	10.6344
ENSG00000204099	NEU4	1.2229	1.88E-09	7.84E-09	10.6342
ENSG00000169258	GPRIN1	1.2843	1.89E-09	7.85E-09	10.6329
ENSG00000120800	UTP20	1.2966	1.89E-09	7.86E-09	10.6316
ENSG00000133195	SLC39A11	1.3365	1.89E-09	7.87E-09	10.6305
ENSG00000132005	RFX1	1.2797	1.91E-09	7.94E-09	10.6208
ENSG00000147364	FBXO25	1.2542	1.92E-09	7.98E-09	10.6161
ENSG00000103253	HAGHL	0.6059	1.93E-09	8.01E-09	10.6121
ENSG00000113593	PPWD1	1.2803	1.96E-09	8.13E-09	10.5981
ENSG00000249115	HAUS5	1.2893	1.96E-09	8.13E-09	10.5976
ENSG00000129116	PALLD	1.4289	1.97E-09	8.17E-09	10.5924
ENSG00000164403	SHROOM1	1.4548	1.97E-09	8.17E-09	10.5923

Gene ID	Gene Symbol	FC	P.Value	adj.P.Val	B
ENSG00000164405	UQCRQ	0.8128	1.99E-09	8.26E-09	10.5812
ENSG00000175931	UBE2O	1.3233	2.01E-09	8.34E-09	10.5714
ENSG00000106536	POU6F2	1.2793	2.02E-09	8.40E-09	10.5650
ENSG00000073282	TP63	1.2601	2.03E-09	8.42E-09	10.5617
ENSG00000091317	CMTM6	1.3350	2.04E-09	8.48E-09	10.5545
ENSG00000006015	REX1BD	0.7786	2.05E-09	8.49E-09	10.5532
ENSG00000101158	NELFCD	1.3050	2.05E-09	8.49E-09	10.5534
ENSG00000172292	CERS6	1.7456	2.06E-09	8.54E-09	10.5478
ENSG00000053918	KCNQ1	1.9363	2.08E-09	8.62E-09	10.5376
ENSG00000179163	FUCA1	0.6747	2.10E-09	8.68E-09	10.5307
ENSG00000188229	TUBB4B	1.3550	2.10E-09	8.71E-09	10.5272
ENSG00000152620	NADK2	1.2685	2.13E-09	8.81E-09	10.5164
ENSG00000145723	GIN1	0.7327	2.13E-09	8.81E-09	10.5158
ENSG00000162878	PKDCC	1.4655	2.16E-09	8.95E-09	10.5003
ENSG00000102543	CDADC1	1.2851	2.17E-09	8.96E-09	10.4988
ENSG00000105185	PDCD5	0.7844	2.20E-09	9.08E-09	10.4854
ENSG00000069493	CLEC2D	1.5456	2.20E-09	9.09E-09	10.4849
ENSG00000145975	FAM217A	1.5056	2.20E-09	9.10E-09	10.4831
ENSG00000163072	NOSTRIN	1.2016	2.20E-09	9.11E-09	10.4815
ENSG00000014216	CAPN1	1.2354	2.23E-09	9.23E-09	10.4686
ENSG00000087903	RFX2	1.3067	2.24E-09	9.28E-09	10.4637
ENSG00000198707	CEP290	1.2903	2.26E-09	9.35E-09	10.4564
ENSG00000150867	PIP4K2A	1.5225	2.29E-09	9.44E-09	10.4462
ENSG00000117407	ARTN	0.4832	2.29E-09	9.45E-09	10.4457
ENSG00000012061	ERCC1	0.8018	2.30E-09	9.49E-09	10.4406
ENSG00000188613	NANOS1	1.5955	2.30E-09	9.51E-09	10.4391
ENSG00000135953	MFSD9	1.3250	2.30E-09	9.51E-09	10.4389
ENSG00000125999	BPIFB1	1.0835	2.31E-09	9.53E-09	10.4366
ENSG00000126214	KLC1	1.2641	2.31E-09	9.53E-09	10.4365
ENSG00000156574	NODAL	1.1527	2.32E-09	9.57E-09	10.4321
ENSG00000107518	ATRNL1	1.4594	2.34E-09	9.64E-09	10.4240
ENSG00000138109	CYP2C9	1.1379	2.35E-09	9.67E-09	10.4206
ENSG00000187607	ZNF286A	1.3486	2.35E-09	9.67E-09	10.4208
ENSG00000173805	HAP1	1.0695	2.35E-09	9.70E-09	10.4176
ENSG00000106803	SEC61B	0.8526	2.36E-09	9.71E-09	10.4163
ENSG00000187726	DNAJB13	1.4431	2.36E-09	9.72E-09	10.4152
ENSG00000165533	TTC8	1.3918	2.38E-09	9.80E-09	10.4067
ENSG00000255804	OR6J1	1.0329	2.38E-09	9.80E-09	10.4068
ENSG00000128165	ADM2	1.5800	2.42E-09	9.98E-09	10.3885
ENSG00000091164	TXNL1	1.1987	2.43E-09	1.00E-08	10.3869
ENSG00000198890	PRMT6	1.4929	2.45E-09	1.01E-08	10.3786
ENSG00000181991	MRPS11	0.7748	2.46E-09	1.01E-08	10.3760
ENSG00000139219	COL2A1	1.1294	2.46E-09	1.01E-08	10.3748
ENSG00000221916	C19orf73	0.7759	2.46E-09	1.01E-08	10.3747
ENSG00000079931	MOXD1	0.5088	2.46E-09	1.01E-08	10.3732
ENSG00000246705	H2AJ	1.5196	2.47E-09	1.02E-08	10.3702
ENSG00000169031	COL4A3	1.5037	2.49E-09	1.02E-08	10.3630
ENSG00000117133	RPF1	1.2472	2.52E-09	1.04E-08	10.3491
ENSG00000132424	PNISR	1.3288	2.54E-09	1.04E-08	10.3437
ENSG00000138378	STAT4	1.6750	2.56E-09	1.05E-08	10.3363
ENSG00000077147	TM9SF3	1.2571	2.58E-09	1.06E-08	10.3285
ENSG00000165688	PMPCA	1.2144	2.58E-09	1.06E-08	10.3278
ENSG00000141837	CACNA1A	1.4501	2.61E-09	1.07E-08	10.3170
ENSG00000102753	KPNA3	1.2150	2.63E-09	1.08E-08	10.3081

Gene ID	Gene Symbol	FC	P.Value	adj.P.Val	B
ENSG00000160408	ST6GALNAC6	1.4104	2.65E-09	1.09E-08	10.3006
ENSG00000184898	RBM43	1.3333	2.65E-09	1.09E-08	10.3003
ENSG00000089820	ARHGAP4	1.3845	2.66E-09	1.09E-08	10.2998
ENSG00000100600	LGMN	1.9399	2.66E-09	1.09E-08	10.2992
ENSG00000198765	SYCP1	1.2565	2.67E-09	1.09E-08	10.2964
ENSG00000213626	LBH	1.6484	2.67E-09	1.09E-08	10.2953
ENSG00000174886	NDUFA11	0.8356	2.69E-09	1.10E-08	10.2889
ENSG00000015133	CCDC88C	1.3182	2.70E-09	1.10E-08	10.2849
ENSG00000164256	PRDM9	1.1484	2.70E-09	1.10E-08	10.2850
ENSG00000181754	AMIGO1	0.7168	2.71E-09	1.11E-08	10.2807
ENSG00000157107	FCHO2	1.3451	2.75E-09	1.13E-08	10.2645
ENSG00000158716	DUSP23	1.6571	2.76E-09	1.13E-08	10.2633
ENSG00000067955	CBFB	1.2611	2.76E-09	1.13E-08	10.2625
ENSG00000106299	WASL	1.2450	2.76E-09	1.13E-08	10.2622
ENSG00000168421	RHOH	1.7831	2.77E-09	1.13E-08	10.2572
ENSG00000197852	INKA2	1.2964	2.77E-09	1.13E-08	10.2572
ENSG00000171827	ZNF570	1.2965	2.78E-09	1.14E-08	10.2540
ENSG00000144550	CPNE9	1.1453	2.78E-09	1.14E-08	10.2538
ENSG00000169891	REPS2	1.4130	2.81E-09	1.15E-08	10.2432
ENSG00000204178	MACO1	1.2277	2.84E-09	1.16E-08	10.2354
ENSG00000171657	GPR82	1.1359	2.85E-09	1.16E-08	10.2314
ENSG00000128581	IFT22	1.4286	2.85E-09	1.17E-08	10.2301
ENSG00000153487	ING1	1.2278	2.88E-09	1.18E-08	10.2202
ENSG00000063015	SEZ6	1.1782	2.88E-09	1.18E-08	10.2193
ENSG00000172689	MS4A10	1.0446	2.90E-09	1.18E-08	10.2155
ENSG00000214517	PPME1	1.2335	2.91E-09	1.19E-08	10.2101
ENSG00000108578	BLMH	1.2617	2.93E-09	1.20E-08	10.2038
ENSG00000130822	PNCK	1.1682	2.93E-09	1.20E-08	10.2032
ENSG00000173207	CKS1B	1.5371	2.94E-09	1.20E-08	10.2014
ENSG00000104880	ARHGEF18	1.2734	2.98E-09	1.21E-08	10.1893
ENSG00000121988	ZRANB3	1.2090	2.99E-09	1.22E-08	10.1858
ENSG00000157020	SEC13	1.2293	2.99E-09	1.22E-08	10.1841
ENSG00000156675	RAB11FIP1	1.8939	3.01E-09	1.23E-08	10.1774
ENSG00000100227	POLDIP3	1.2338	3.05E-09	1.24E-08	10.1639
ENSG00000136153	LMO7	1.3277	3.06E-09	1.24E-08	10.1634
ENSG00000138381	ASNSD1	1.2151	3.06E-09	1.24E-08	10.1635
ENSG00000166086	JAM3	1.4759	3.06E-09	1.24E-08	10.1629
ENSG00000113845	TIMMDC1	0.7032	3.07E-09	1.25E-08	10.1574
ENSG00000155561	NUP205	1.2454	3.11E-09	1.27E-08	10.1464
ENSG00000130518	IQCN	1.6422	3.12E-09	1.27E-08	10.1436
ENSG00000132561	MATN2	1.2349	3.15E-09	1.28E-08	10.1332
ENSG00000158882	TOMM40L	1.2802	3.18E-09	1.29E-08	10.1250
ENSG00000168297	PXK	1.2751	3.18E-09	1.29E-08	10.1243
ENSG00000160957	RECQL4	1.5039	3.19E-09	1.30E-08	10.1227
ENSG00000176658	MYO1D	1.3606	3.20E-09	1.30E-08	10.1186
ENSG00000137094	DNAJB5	1.3489	3.20E-09	1.30E-08	10.1182
ENSG00000008283	CYB561	1.4469	3.20E-09	1.30E-08	10.1171
ENSG00000101306	MYLK2	1.0617	3.24E-09	1.32E-08	10.1053
ENSG00000205339	IPO7	0.7444	3.25E-09	1.32E-08	10.1027
ENSG00000110436	SLC1A2	1.1390	3.26E-09	1.32E-08	10.1004
ENSG00000114554	PLXNA1	1.4749	3.26E-09	1.32E-08	10.1005
ENSG00000183475	ASB7	1.2989	3.26E-09	1.32E-08	10.1002
ENSG00000157303	SUSD3	0.6188	3.26E-09	1.32E-08	10.0991
ENSG00000011422	PLAUR	1.6891	3.29E-09	1.34E-08	10.0900

Gene ID	Gene Symbol	FC	P.Value	adj.P.Val	B
ENSG00000103249	CLCN7	1.3504	3.32E-09	1.35E-08	10.0823
ENSG00000180008	SOCS4	1.2713	3.33E-09	1.35E-08	10.0802
ENSG00000160867	FGFR4	1.2973	3.35E-09	1.36E-08	10.0740
ENSG00000196935	SRGAP1	1.0973	3.35E-09	1.36E-08	10.0729
ENSG00000223501	VPS52	1.2637	3.38E-09	1.37E-08	10.0664
ENSG00000143515	ATP8B2	1.4289	3.38E-09	1.37E-08	10.0651
ENSG00000152475	ZNF837	0.7922	3.38E-09	1.37E-08	10.0640
ENSG00000123444	KBTBD4	1.2432	3.41E-09	1.38E-08	10.0556
ENSG00000145362	ANK2	1.2264	3.42E-09	1.38E-08	10.0548
ENSG00000159640	ACE	1.2678	3.42E-09	1.38E-08	10.0546
ENSG00000150722	PPP1R1C	1.4320	3.43E-09	1.39E-08	10.0510
ENSG00000126562	WNK4	1.1887	3.45E-09	1.40E-08	10.0451
ENSG00000071243	ING3	1.2688	3.50E-09	1.41E-08	10.0318
ENSG00000104833	TUBB4A	1.3519	3.51E-09	1.42E-08	10.0293
ENSG00000102158	MAGT1	1.3233	3.52E-09	1.42E-08	10.0254
ENSG00000157110	RBPMS	1.3014	3.53E-09	1.43E-08	10.0230
ENSG00000169371	SNUPN	0.7671	3.57E-09	1.44E-08	10.0110
ENSG00000022277	RTF2	1.2112	3.58E-09	1.45E-08	10.0089
ENSG00000106483	SFRP4	1.1110	3.62E-09	1.46E-08	9.9995
ENSG00000133316	WDR74	1.4386	3.62E-09	1.46E-08	9.9986
ENSG00000131686	CA6	1.1300	3.62E-09	1.46E-08	9.9982
ENSG00000162772	ATF3	1.9747	3.62E-09	1.46E-08	9.9971
ENSG00000171612	SLC25A33	1.2286	3.64E-09	1.47E-08	9.9939
ENSG00000163689	CFAP20DC	1.3269	3.65E-09	1.47E-08	9.9908
ENSG00000179774	ATOH7	1.1226	3.66E-09	1.48E-08	9.9876
ENSG00000241837	ATP5PO	0.8279	3.67E-09	1.48E-08	9.9855
ENSG00000242173	ARHGDIG	1.2930	3.68E-09	1.48E-08	9.9819
ENSG00000189143	CLDN4	1.2156	3.69E-09	1.49E-08	9.9788
ENSG00000198053	SIRPA	1.7632	3.69E-09	1.49E-08	9.9785
ENSG00000124406	ATP8A1	1.4184	3.75E-09	1.51E-08	9.9649
ENSG00000156219	ART3	1.1446	3.75E-09	1.51E-08	9.9631
ENSG00000106588	PSMA2	0.8112	3.76E-09	1.52E-08	9.9603
ENSG00000149346	SLX4IP	1.2626	3.76E-09	1.52E-08	9.9601
ENSG00000181666	ZNF875	1.5983	3.78E-09	1.52E-08	9.9569
ENSG00000180822	PSMG4	0.8034	3.83E-09	1.54E-08	9.9434
ENSG00000051108	HERPUD1	1.3975	3.84E-09	1.55E-08	9.9397
ENSG00000090612	ZNF268	1.3858	3.91E-09	1.57E-08	9.9228
ENSG00000187838	PLSCR3	1.4349	3.97E-09	1.60E-08	9.9094
ENSG00000114446	IFT57	1.3058	3.97E-09	1.60E-08	9.9075
ENSG00000172840	PDP2	1.2814	4.00E-09	1.61E-08	9.9010
ENSG00000161647	MPP3	1.2993	4.02E-09	1.62E-08	9.8964
ENSG00000137752	CASP1	0.6110	4.10E-09	1.65E-08	9.8774
ENSG00000186106	ANKRD46	0.6889	4.11E-09	1.65E-08	9.8758
ENSG00000130182	ZSCAN10	1.0943	4.12E-09	1.65E-08	9.8734
ENSG00000145386	CCNA2	1.5052	4.14E-09	1.66E-08	9.8667
ENSG00000062725	APPBP2	1.2745	4.16E-09	1.67E-08	9.8637
ENSG00000096433	ITPR3	1.4457	4.16E-09	1.67E-08	9.8635
ENSG00000159023	EPB41	1.3213	4.17E-09	1.67E-08	9.8612
ENSG00000137171	KLC4	1.2661	4.17E-09	1.68E-08	9.8597
ENSG00000143178	TBX19	1.2147	4.18E-09	1.68E-08	9.8577
ENSG00000117410	ATP6V0B	1.2804	4.20E-09	1.68E-08	9.8538
ENSG00000103274	NUBP1	1.2255	4.21E-09	1.69E-08	9.8504
ENSG00000153933	DGKE	1.3950	4.21E-09	1.69E-08	9.8502
ENSG00000185344	ATP6V0A2	1.2683	4.22E-09	1.69E-08	9.8494

Gene ID	Gene Symbol	FC	P.Value	adj.P.Val	B
ENSG00000186710	CFAP73	1.2331	4.23E-09	1.70E-08	9.8459
ENSG00000143570	SLC39A1	1.2666	4.24E-09	1.70E-08	9.8436
ENSG00000146729	NIPSNAP2	1.2356	4.26E-09	1.71E-08	9.8408
ENSG00000181218	H2AW	2.2361	4.26E-09	1.71E-08	9.8404
ENSG00000242616	GNG10	0.7323	4.26E-09	1.71E-08	9.8394
ENSG00000171823	FBXL14	1.3129	4.29E-09	1.72E-08	9.8332
ENSG00000188467	SLC24A5	1.4170	4.30E-09	1.72E-08	9.8313
ENSG00000255529	POLR2M	1.2194	4.36E-09	1.74E-08	9.8182
ENSG00000203485	INF2	1.5472	4.36E-09	1.75E-08	9.8163
ENSG00000037637	FBXO42	1.2256	4.41E-09	1.76E-08	9.8069
ENSG00000141084	RANBP10	1.2599	4.41E-09	1.77E-08	9.8055
ENSG00000143624	INTS3	1.4158	4.42E-09	1.77E-08	9.8035
ENSG00000055208	TAB2	1.3237	4.46E-09	1.78E-08	9.7963
ENSG00000173821	RNF213	1.4627	4.46E-09	1.78E-08	9.7956
ENSG00000106591	MRPL32	0.8129	4.49E-09	1.80E-08	9.7881
ENSG00000122432	SPATA1	1.2894	4.50E-09	1.80E-08	9.7869
ENSG00000186141	POLR3C	1.2433	4.51E-09	1.80E-08	9.7839
ENSG00000111371	SLC38A1	1.3529	4.55E-09	1.82E-08	9.7760
ENSG00000109189	USP46	1.2598	4.55E-09	1.82E-08	9.7754
ENSG00000038532	CLEC16A	1.2684	4.57E-09	1.82E-08	9.7721
ENSG00000058063	ATP11B	1.3370	4.58E-09	1.83E-08	9.7696
ENSG00000137868	STRA6	0.5795	4.63E-09	1.85E-08	9.7582
ENSG00000185404	SP140L	1.2959	4.67E-09	1.86E-08	9.7505
ENSG00000121807	CCR2	0.4112	4.70E-09	1.87E-08	9.7443
ENSG00000130332	LSM7	0.8127	4.72E-09	1.88E-08	9.7392
ENSG00000158164	TMSB15A	1.5669	4.74E-09	1.89E-08	9.7358
ENSG00000073060	SCARB1	1.5465	4.78E-09	1.91E-08	9.7273
ENSG00000104888	SLC17A7	1.0725	4.86E-09	1.94E-08	9.7125
ENSG00000120211	INSL4	1.0530	4.86E-09	1.94E-08	9.7121
ENSG00000137497	NUMA1	1.3250	4.92E-09	1.96E-08	9.7006
ENSG00000160145	KALRN	1.1569	5.01E-09	2.00E-08	9.6819
ENSG00000162869	PPP1R21	1.3203	5.02E-09	2.00E-08	9.6798
ENSG00000233276	GPX1	0.6374	5.10E-09	2.03E-08	9.6640
ENSG00000108599	AKAP10	1.2246	5.15E-09	2.05E-08	9.6553
ENSG00000142615	CELA2A	1.1588	5.17E-09	2.06E-08	9.6522
ENSG00000241127	YAE1	0.7074	5.22E-09	2.08E-08	9.6416
ENSG00000204371	EHMT2	1.2838	5.22E-09	2.08E-08	9.6414
ENSG00000047597	XK	1.5656	5.25E-09	2.09E-08	9.6375
ENSG00000169241	SLC50A1	1.3974	5.25E-09	2.09E-08	9.6369
ENSG00000107371	EXOSC3	0.7011	5.26E-09	2.09E-08	9.6342
ENSG00000120889	TNFRSF10B	1.5214	5.28E-09	2.10E-08	9.6310
ENSG00000184992	BRI3BP	1.4070	5.28E-09	2.10E-08	9.6306
ENSG00000131019	ULBP3	1.1870	5.33E-09	2.12E-08	9.6224
ENSG00000147127	RAB41	1.1377	5.34E-09	2.12E-08	9.6203
ENSG00000161940	BCL6B	1.3452	5.48E-09	2.18E-08	9.5952
ENSG00000128891	CCDC32	0.7289	5.49E-09	2.18E-08	9.5930
ENSG00000150630	VEGFC	0.6070	5.49E-09	2.18E-08	9.5923
ENSG00000103415	HMOX2	0.7398	5.55E-09	2.20E-08	9.5829
ENSG00000119414	PPP6C	1.1959	5.59E-09	2.22E-08	9.5753
ENSG00000073150	PANX2	1.3326	5.60E-09	2.22E-08	9.5738
ENSG00000002549	LAP3	1.3666	5.61E-09	2.22E-08	9.5722
ENSG00000155868	MED7	0.7450	5.61E-09	2.23E-08	9.5718
ENSG00000184661	CDCA2	1.3590	5.62E-09	2.23E-08	9.5699
ENSG00000158887	MPZ	1.3320	5.63E-09	2.23E-08	9.5680

Gene ID	Gene Symbol	FC	P.Value	adj.P.Val	B
ENSG00000108175	ZMIZ1	1.8192	5.64E-09	2.23E-08	9.5675
ENSG00000004478	FKBP4	1.3042	5.66E-09	2.24E-08	9.5637
ENSG00000100528	CNIH1	0.8132	5.69E-09	2.25E-08	9.5584
ENSG00000118292	C1orf54	1.4665	5.71E-09	2.26E-08	9.5544
ENSG00000096395	MLN	1.1280	5.72E-09	2.27E-08	9.5527
ENSG00000163683	SMIM14	0.6103	5.72E-09	2.27E-08	9.5527
ENSG00000126882	FAM78A	0.6925	5.76E-09	2.28E-08	9.5459
ENSG00000234465	PINLYP	0.5834	5.77E-09	2.28E-08	9.5452
ENSG00000204388	HSPA1B	1.9786	5.80E-09	2.29E-08	9.5401
ENSG00000096063	SRPK1	1.2972	5.83E-09	2.30E-08	9.5353
ENSG00000161664	ASB16	1.3044	5.85E-09	2.31E-08	9.5321
ENSG00000127054	INTS11	1.2339	5.87E-09	2.32E-08	9.5275
ENSG00000165669	FAM204A	1.1902	5.89E-09	2.33E-08	9.5254
ENSG00000089091	DZANK1	1.3368	5.92E-09	2.34E-08	9.5191
ENSG00000130589	HELZ2	1.5466	5.98E-09	2.36E-08	9.5095
ENSG00000068097	HEATR6	1.3250	6.01E-09	2.37E-08	9.5057
ENSG00000244414	CFHR1	1.3906	6.01E-09	2.37E-08	9.5051
ENSG00000109339	MAPK10	1.6013	6.01E-09	2.38E-08	9.5043
ENSG00000131941	RHPN2	1.4430	6.07E-09	2.39E-08	9.4959
ENSG00000167566	NCKAP5L	1.2099	6.07E-09	2.39E-08	9.4960
ENSG00000116668	SWT1	1.3404	6.11E-09	2.41E-08	9.4891
ENSG00000102174	PHEX	1.0994	6.11E-09	2.41E-08	9.4884
ENSG00000115267	IFIH1	1.4191	6.11E-09	2.41E-08	9.4884
ENSG00000140474	ULK3	0.7057	6.15E-09	2.43E-08	9.4820
ENSG00000118922	KLF12	1.3749	6.20E-09	2.44E-08	9.4753
ENSG00000065923	SLC9A7	1.4367	6.21E-09	2.45E-08	9.4735
ENSG00000132437	DDC	1.1003	6.23E-09	2.46E-08	9.4694
ENSG00000104635	SLC39A14	0.6642	6.25E-09	2.46E-08	9.4675
ENSG00000159596	TMEM69	0.7658	6.29E-09	2.48E-08	9.4604
ENSG00000179115	FARSA	0.6973	6.33E-09	2.49E-08	9.4549
ENSG00000067064	IDI1	1.3387	6.34E-09	2.49E-08	9.4538
ENSG00000184575	XPOT	1.3401	6.34E-09	2.50E-08	9.4532
ENSG00000174442	ZWILCH	1.3033	6.34E-09	2.50E-08	9.4525
ENSG00000119431	HDHD3	0.6793	6.38E-09	2.51E-08	9.4471
ENSG00000143851	PTPN7	1.4508	6.38E-09	2.51E-08	9.4467
ENSG00000144061	NPHP1	1.2535	6.41E-09	2.52E-08	9.4431
ENSG00000104814	MAP4K1	1.3132	6.42E-09	2.52E-08	9.4415
ENSG00000196182	STK40	1.3023	6.52E-09	2.56E-08	9.4257
ENSG00000196865	NHLRC2	1.2348	6.55E-09	2.57E-08	9.4216
ENSG00000198945	L3MBTL3	1.4318	6.64E-09	2.61E-08	9.4074
ENSG00000077782	FGFR1	1.3987	6.66E-09	2.62E-08	9.4052
ENSG00000124772	CPNE5	0.6393	6.68E-09	2.63E-08	9.4021
ENSG00000196972	SMIM10L2B	1.1834	6.69E-09	2.63E-08	9.4004
ENSG00000221886	ZBED8	0.7399	6.71E-09	2.64E-08	9.3978
ENSG00000137807	KIF23	1.5011	6.73E-09	2.64E-08	9.3945
ENSG00000173418	NAA20	1.1856	6.79E-09	2.67E-08	9.3866
ENSG00000100632	ERH	1.2199	6.79E-09	2.67E-08	9.3861
ENSG00000163808	KIF15	1.3991	6.81E-09	2.67E-08	9.3840
ENSG00000153291	SLC25A27	2.0017	6.84E-09	2.68E-08	9.3795
ENSG00000178229	ZNF543	1.2180	6.84E-09	2.68E-08	9.3792
ENSG00000184207	PGP	1.2834	6.85E-09	2.69E-08	9.3773
ENSG00000104936	DMPK	1.3266	6.88E-09	2.70E-08	9.3733
ENSG00000156467	UQCRB	0.8247	6.90E-09	2.71E-08	9.3704
ENSG00000118705	RPN2	1.3229	7.00E-09	2.74E-08	9.3569

Gene ID	Gene Symbol	FC	P.Value	adj.P.Val	B
ENSG00000232237	ASCL5	1.0378	7.02E-09	2.75E-08	9.3541
ENSG00000127483	HP1BP3	1.3365	7.02E-09	2.75E-08	9.3534
ENSG00000184304	PRKD1	1.4086	7.09E-09	2.78E-08	9.3448
ENSG00000125249	RAP2A	1.3394	7.14E-09	2.80E-08	9.3374
ENSG00000053770	AP5M1	1.2612	7.16E-09	2.80E-08	9.3348
ENSG00000142556	ZNF614	1.2210	7.24E-09	2.83E-08	9.3245
ENSG00000181982	CCDC149	1.8058	7.29E-09	2.85E-08	9.3176
ENSG00000177426	TGIF1	1.5052	7.32E-09	2.87E-08	9.3130
ENSG00000143578	CREB3L4	1.3870	7.49E-09	2.93E-08	9.2904
ENSG00000115687	PASK	1.4941	7.50E-09	2.93E-08	9.2897
ENSG00000103196	CRISPLD2	1.2263	7.53E-09	2.95E-08	9.2856
ENSG00000005471	ABCB4	1.4751	7.56E-09	2.96E-08	9.2819
ENSG00000100139	MICALL1	1.2493	7.56E-09	2.96E-08	9.2815
ENSG00000187778	MCRS1	1.2189	7.61E-09	2.97E-08	9.2759
ENSG00000131127	ZNF141	1.4206	7.63E-09	2.98E-08	9.2724
ENSG00000175497	DPP10	1.2053	7.64E-09	2.99E-08	9.2712
ENSG00000105122	RASAL3	1.4292	7.69E-09	3.01E-08	9.2648
ENSG00000136943	CTSV	1.1624	7.76E-09	3.03E-08	9.2570
ENSG00000143995	MEIS1	1.4724	7.77E-09	3.03E-08	9.2554
ENSG00000152254	G6PC2	1.0339	7.80E-09	3.04E-08	9.2516
ENSG00000136536	MARCHF7	1.2841	7.81E-09	3.05E-08	9.2509
ENSG00000124067	SLC12A4	1.4304	7.86E-09	3.07E-08	9.2442
ENSG00000115970	THADA	1.2450	7.88E-09	3.07E-08	9.2418
ENSG00000125962	ARMCX5	1.3418	7.90E-09	3.08E-08	9.2391
ENSG00000144354	CDCA7	1.3357	7.95E-09	3.10E-08	9.2336
ENSG00000055957	ITIH1	1.1089	7.97E-09	3.11E-08	9.2306
ENSG00000213397	HAUS7	1.3194	8.03E-09	3.13E-08	9.2227
ENSG00000112902	SEMA5A	1.2987	8.07E-09	3.15E-08	9.2180
ENSG00000173153	ESRRA	0.7583	8.11E-09	3.16E-08	9.2142
ENSG00000125753	VASP	1.3352	8.13E-09	3.17E-08	9.2116
ENSG00000205138	SDHAF1	0.7931	8.17E-09	3.18E-08	9.2066
ENSG00000075340	ADD2	1.4101	8.20E-09	3.19E-08	9.2030
ENSG00000126010	GRPR	1.0881	8.20E-09	3.19E-08	9.2026
ENSG00000131437	KIF3A	1.2947	8.23E-09	3.20E-08	9.1989
ENSG00000125255	SLC10A2	1.0379	8.28E-09	3.22E-08	9.1939
ENSG00000162522	KIAA1522	1.4194	8.30E-09	3.23E-08	9.1915
ENSG00000163521	GLB1L	1.2815	8.30E-09	3.23E-08	9.1906
ENSG00000078140	UBE2K	1.2149	8.31E-09	3.23E-08	9.1902
ENSG00000182612	TSPAN10	1.1776	8.32E-09	3.23E-08	9.1892
ENSG00000146859	TMEM140	1.3542	8.33E-09	3.24E-08	9.1872
ENSG00000135486	HNRNPA1	1.1732	8.34E-09	3.24E-08	9.1868
ENSG00000179256	SMCO3	1.0463	8.36E-09	3.25E-08	9.1841
ENSG00000215440	NPEPL1	1.3170	8.36E-09	3.25E-08	9.1837
ENSG00000155975	VPS37A	1.2684	8.37E-09	3.25E-08	9.1830
ENSG00000147130	ZMYM3	1.2900	8.39E-09	3.26E-08	9.1807
ENSG00000205755	CRLF2	1.1010	8.43E-09	3.27E-08	9.1759
ENSG00000102221	JADE3	1.2561	8.44E-09	3.28E-08	9.1746
ENSG00000171488	LRRC8C	1.4005	8.48E-09	3.29E-08	9.1706
ENSG00000130165	ELOF1	0.8177	8.48E-09	3.29E-08	9.1700
ENSG00000130396	AFDN	1.6365	8.51E-09	3.30E-08	9.1664
ENSG00000187147	RNF220	1.1856	8.53E-09	3.31E-08	9.1649
ENSG00000135390	ATP5MC2	0.8644	8.55E-09	3.32E-08	9.1618
ENSG00000137843	PAK6	1.5211	8.56E-09	3.32E-08	9.1614
ENSG00000177791	MYOZ1	1.0256	8.57E-09	3.32E-08	9.1598

Gene ID	Gene Symbol	FC	P.Value	adj.P.Val	B
ENSG00000143776	CDC42BPA	1.3470	8.59E-09	3.33E-08	9.1574
ENSG00000181625	SLX1B	1.7184	8.84E-09	3.43E-08	9.1296
ENSG00000145740	SLC30A5	0.7819	8.86E-09	3.43E-08	9.1275
ENSG00000100365	NCF4	0.6374	8.87E-09	3.43E-08	9.1267
ENSG00000140688	RUSF1	1.2855	8.99E-09	3.48E-08	9.1135
ENSG00000175548	ALG10B	1.3361	9.08E-09	3.52E-08	9.1033
ENSG00000135069	PSAT1	0.5677	9.09E-09	3.52E-08	9.1031
ENSG00000111897	SERINC1	1.2939	9.12E-09	3.53E-08	9.0997
ENSG00000140395	WDR61	0.7741	9.12E-09	3.53E-08	9.0993
ENSG00000145388	METTL14	1.2603	9.21E-09	3.56E-08	9.0900
ENSG00000204262	COL5A2	1.2053	9.22E-09	3.57E-08	9.0886
ENSG00000013297	CLDN11	1.2491	9.24E-09	3.57E-08	9.0870
ENSG00000159256	MORC3	1.2427	9.26E-09	3.58E-08	9.0844
ENSG00000133789	SWAP70	1.3019	9.28E-09	3.59E-08	9.0821
ENSG00000102383	ZDHHC15	1.2668	9.36E-09	3.62E-08	9.0746
ENSG00000110619	CARS1	1.2781	9.39E-09	3.63E-08	9.0708
ENSG00000159459	UBR1	1.2479	9.40E-09	3.63E-08	9.0704
ENSG00000124103	FAM209A	1.2369	9.46E-09	3.65E-08	9.0639
ENSG00000149735	GPHA2	1.1780	9.52E-09	3.68E-08	9.0577
ENSG00000134548	SPX	1.3406	9.55E-09	3.69E-08	9.0546
ENSG00000196576	PLXNB2	1.6718	9.59E-09	3.70E-08	9.0505
ENSG00000143772	ITPKB	1.5828	9.62E-09	3.71E-08	9.0479
ENSG00000204217	BMPR2	1.3190	9.67E-09	3.73E-08	9.0424
ENSG00000180233	ZNRF2	1.2603	9.78E-09	3.77E-08	9.0314
ENSG00000168958	MFF	1.1966	9.90E-09	3.82E-08	9.0198
ENSG00000108094	CUL2	1.2271	1.01E-08	3.90E-08	8.9999
ENSG00000106560	GIMAP2	0.7276	1.01E-08	3.90E-08	8.9994
ENSG00000039560	RAI14	1.2878	1.03E-08	3.95E-08	8.9857
ENSG00000167077	MEI1	0.6932	1.04E-08	4.02E-08	8.9689
ENSG00000099139	PCSK5	1.4334	1.05E-08	4.03E-08	8.9666
ENSG00000126231	PROZ	0.8015	1.05E-08	4.04E-08	8.9639
ENSG00000059915	PSD	1.2194	1.06E-08	4.09E-08	8.9508
ENSG00000164066	INTU	1.2901	1.06E-08	4.10E-08	8.9489
ENSG00000256436	TAS2R31	1.0969	1.07E-08	4.10E-08	8.9482
ENSG00000139433	GLTP	1.2309	1.07E-08	4.13E-08	8.9405
ENSG00000172845	SP3	1.2332	1.08E-08	4.14E-08	8.9384
ENSG00000103966	EHD4	1.5003	1.08E-08	4.16E-08	8.9341
ENSG00000134313	KIDINS220	1.3114	1.09E-08	4.20E-08	8.9251
ENSG00000138771	SHROOM3	1.7904	1.10E-08	4.22E-08	8.9190
ENSG00000158292	GPR153	1.1333	1.10E-08	4.23E-08	8.9176
ENSG00000074201	CLNS1A	0.7705	1.11E-08	4.27E-08	8.9081
ENSG00000086598	TMED2	1.2510	1.11E-08	4.27E-08	8.9071
ENSG00000171100	MTM1	1.2883	1.12E-08	4.30E-08	8.9005
ENSG00000124006	OBSL1	1.4469	1.12E-08	4.32E-08	8.8965
ENSG00000146592	CREB5	1.7031	1.13E-08	4.33E-08	8.8943
ENSG00000177614	PGBD5	1.2572	1.13E-08	4.33E-08	8.8920
ENSG00000214700	C12orf71	1.0785	1.16E-08	4.45E-08	8.8673
ENSG00000170035	UBE2E3	1.2205	1.16E-08	4.45E-08	8.8663
ENSG00000091536	MYO15A	1.2839	1.16E-08	4.46E-08	8.8645
ENSG00000103175	WFDC1	1.0522	1.16E-08	4.46E-08	8.8632
ENSG00000065413	ANKRD44	1.4363	1.17E-08	4.49E-08	8.8570
ENSG00000231767	RPS27AP5	0.8901	1.17E-08	4.50E-08	8.8550
ENSG00000008056	SYN1	1.0746	1.17E-08	4.50E-08	8.8534
ENSG00000169398	PTK2	1.3917	1.18E-08	4.54E-08	8.8464

Gene ID	Gene Symbol	FC	P.Value	adj.P.Val	B
ENSG00000242372	EIF6	1.2133	1.19E-08	4.54E-08	8.8444
ENSG00000185477	GPRIN3	1.7412	1.19E-08	4.57E-08	8.8382
ENSG00000147324	MFHAS1	1.3411	1.20E-08	4.58E-08	8.8360
ENSG00000103707	MTFMT	0.7653	1.20E-08	4.60E-08	8.8321
ENSG00000165632	TAF3	1.2183	1.20E-08	4.60E-08	8.8315
ENSG00000166828	SCNN1G	1.1889	1.21E-08	4.62E-08	8.8266
ENSG00000203952	CCDC160	1.0363	1.21E-08	4.63E-08	8.8256
ENSG00000139734	DIAPH3	1.2512	1.22E-08	4.66E-08	8.8183
ENSG00000164162	ANAPC10	1.2226	1.22E-08	4.68E-08	8.8150
ENSG00000115648	MLPH	1.1260	1.22E-08	4.69E-08	8.8129
ENSG00000152642	GPD1L	0.7714	1.23E-08	4.70E-08	8.8088
ENSG00000100129	EIF3L	0.8083	1.23E-08	4.71E-08	8.8068
ENSG00000137310	TCF19	1.5735	1.23E-08	4.72E-08	8.8059
ENSG00000125503	PPP1R12C	1.2243	1.25E-08	4.80E-08	8.7895
ENSG00000164761	TNFRSF11B	1.0677	1.26E-08	4.83E-08	8.7828
ENSG00000182621	PLCB1	1.6642	1.26E-08	4.83E-08	8.7817
ENSG00000110958	PTGES3	1.2146	1.29E-08	4.92E-08	8.7643
ENSG00000120913	PDLIM2	0.6317	1.29E-08	4.93E-08	8.7627
ENSG00000090487	SPG21	0.8040	1.29E-08	4.93E-08	8.7624
ENSG00000162733	DDR2	1.3844	1.31E-08	4.99E-08	8.7499
ENSG00000103184	SEC14L5	1.0429	1.31E-08	5.02E-08	8.7445
ENSG00000135917	SLC19A3	1.1059	1.32E-08	5.03E-08	8.7422
ENSG00000165304	MELK	1.4941	1.34E-08	5.11E-08	8.7263
ENSG00000242265	PEG10	1.6239	1.35E-08	5.15E-08	8.7181
ENSG00000187867	PALM3	1.0938	1.35E-08	5.16E-08	8.7169
ENSG00000171475	WIPF2	1.2532	1.36E-08	5.20E-08	8.7093
ENSG00000112041	TULP1	1.1337	1.36E-08	5.20E-08	8.7077
ENSG00000134398	ERN2	1.0839	1.38E-08	5.26E-08	8.6971
ENSG00000158042	MRPL17	0.7734	1.38E-08	5.26E-08	8.6972
ENSG00000156509	FBXO43	1.2412	1.39E-08	5.28E-08	8.6932
ENSG00000166881	NEMP1	1.3280	1.39E-08	5.29E-08	8.6914
ENSG00000172780	RAB43	1.3066	1.39E-08	5.30E-08	8.6897
ENSG00000177469	CAVIN1	1.2355	1.39E-08	5.30E-08	8.6894
ENSG00000197724	PHF2	1.3356	1.39E-08	5.30E-08	8.6886
ENSG00000100106	TRIOBP	1.2509	1.40E-08	5.31E-08	8.6856
ENSG00000171566	PLRG1	1.2595	1.40E-08	5.32E-08	8.6843
ENSG00000184731	FAM110C	1.2749	1.40E-08	5.33E-08	8.6824
ENSG00000107164	FUBP3	1.1776	1.40E-08	5.33E-08	8.6817
ENSG00000164756	SLC30A8	1.2206	1.41E-08	5.34E-08	8.6793
ENSG00000131584	ACAP3	1.3872	1.41E-08	5.35E-08	8.6784
ENSG00000160785	SLC25A44	1.2647	1.41E-08	5.37E-08	8.6741
ENSG00000143409	MINDY1	1.3380	1.42E-08	5.40E-08	8.6682
ENSG00000105245	NUMBL	1.3316	1.43E-08	5.43E-08	8.6636
ENSG00000008869	HEATR5B	1.3314	1.43E-08	5.43E-08	8.6621
ENSG00000101489	CELF4	1.1972	1.44E-08	5.46E-08	8.6570
ENSG00000107890	ANKRD26	1.2609	1.44E-08	5.47E-08	8.6559
ENSG00000270170	NCBP2AS2	1.2478	1.44E-08	5.48E-08	8.6542
ENSG00000120137	PANK3	1.2645	1.45E-08	5.49E-08	8.6522
ENSG00000132855	ANGPTL3	1.1062	1.45E-08	5.51E-08	8.6474
ENSG00000034533	ASTE1	1.2507	1.48E-08	5.62E-08	8.6288
ENSG00000120709	FAM53C	1.2466	1.49E-08	5.63E-08	8.6256
ENSG00000132952	USPL1	1.3424	1.49E-08	5.64E-08	8.6247
ENSG00000188878	FBF1	1.4196	1.50E-08	5.68E-08	8.6172
ENSG00000177519	RPRM	1.8195	1.50E-08	5.69E-08	8.6156

Gene ID	Gene Symbol	FC	P.Value	adj.P.Val	B
ENSG00000140307	GTF2A2	0.8099	1.50E-08	5.70E-08	8.6142
ENSG00000130349	MTRES1	0.7102	1.51E-08	5.71E-08	8.6123
ENSG00000104419	NDRG1	1.7359	1.51E-08	5.72E-08	8.6091
ENSG00000122367	LDB3	1.0707	1.52E-08	5.77E-08	8.6019
ENSG00000242485	MRPL20	0.8311	1.53E-08	5.79E-08	8.5978
ENSG00000198113	TOR4A	0.7428	1.53E-08	5.80E-08	8.5963
ENSG00000120690	ELF1	1.3154	1.53E-08	5.80E-08	8.5949
ENSG00000146587	RBAK	1.2205	1.54E-08	5.82E-08	8.5912
ENSG00000152465	NMT2	1.3556	1.54E-08	5.83E-08	8.5895
ENSG00000189051	RNF222	1.0467	1.54E-08	5.84E-08	8.5881
ENSG00000147852	VLDLR	1.4201	1.54E-08	5.84E-08	8.5875
ENSG00000145358	DDIT4L	1.1055	1.55E-08	5.85E-08	8.5870
ENSG00000175793	SFN	1.3912	1.55E-08	5.87E-08	8.5834
ENSG00000142864	SERBP1	1.2059	1.56E-08	5.89E-08	8.5786
ENSG00000109758	HGFAC	1.0634	1.56E-08	5.90E-08	8.5772
ENSG00000101337	TM9SF4	1.2057	1.57E-08	5.92E-08	8.5747
ENSG00000163945	UVSSA	1.3927	1.57E-08	5.92E-08	8.5743
ENSG00000198429	ZNF69	0.7460	1.58E-08	5.95E-08	8.5679
ENSG00000165185	KIAA1958	1.2454	1.58E-08	5.96E-08	8.5671
ENSG00000142173	COL6A2	1.5178	1.58E-08	5.98E-08	8.5640
ENSG00000108379	WNT3	1.1485	1.58E-08	5.98E-08	8.5633
ENSG00000185565	LSAMP	0.4963	1.59E-08	5.99E-08	8.5618
ENSG00000186714	CCDC73	1.1120	1.59E-08	5.99E-08	8.5616
ENSG00000117010	ZNF684	1.1881	1.60E-08	6.03E-08	8.5541
ENSG00000047188	YTHDC2	1.2464	1.60E-08	6.05E-08	8.5515
ENSG00000125898	FAM110A	1.3130	1.61E-08	6.07E-08	8.5484
ENSG00000211450	SELENOH	0.8137	1.62E-08	6.09E-08	8.5441
ENSG00000254995	STX16-NPEPL1	1.4794	1.62E-08	6.09E-08	8.5438
ENSG00000112685	EXOC2	1.2172	1.64E-08	6.18E-08	8.5292
ENSG00000130783	CCDC62	1.0729	1.67E-08	6.29E-08	8.5120
ENSG00000131408	NR1H2	1.2405	1.67E-08	6.29E-08	8.5122
ENSG00000253626	EIF5AL1	1.0584	1.69E-08	6.35E-08	8.5026
ENSG00000183696	UPP1	1.4793	1.70E-08	6.42E-08	8.4920
ENSG00000105173	CCNE1	0.6515	1.71E-08	6.42E-08	8.4915
ENSG00000101082	SLA2	1.2067	1.71E-08	6.43E-08	8.4903
ENSG00000180660	MAB21L1	0.6944	1.71E-08	6.44E-08	8.4876
ENSG00000205643	CDPF1	0.7476	1.71E-08	6.44E-08	8.4874
ENSG00000131558	EXOC4	1.1922	1.72E-08	6.48E-08	8.4813
ENSG00000269404	SPIB	1.3089	1.73E-08	6.50E-08	8.4793
ENSG00000049860	HEXB	0.7580	1.73E-08	6.50E-08	8.4789
ENSG00000115109	EPB41L5	1.3349	1.74E-08	6.56E-08	8.4691
ENSG00000131373	HACL1	0.8130	1.75E-08	6.58E-08	8.4667
ENSG00000102271	KLHL4	1.3467	1.75E-08	6.58E-08	8.4656
ENSG00000167302	TEPSIN	1.2957	1.76E-08	6.60E-08	8.4626
ENSG00000135677	GNS	1.2931	1.77E-08	6.64E-08	8.4571
ENSG00000168386	FILIP1L	1.2123	1.77E-08	6.65E-08	8.4552
ENSG00000115468	EFHD1	1.1383	1.77E-08	6.66E-08	8.4534
ENSG00000108439	PNPO	0.6983	1.78E-08	6.67E-08	8.4522
ENSG00000239605	STPG4	1.1824	1.78E-08	6.67E-08	8.4520
ENSG00000108700	CCL8	0.6550	1.78E-08	6.69E-08	8.4483
ENSG00000140853	NLRC5	1.5053	1.80E-08	6.76E-08	8.4388
ENSG00000061938	TNK2	1.3691	1.80E-08	6.77E-08	8.4366
ENSG00000120885	CLU	1.7548	1.81E-08	6.79E-08	8.4341
ENSG00000134294	SLC38A2	1.3629	1.81E-08	6.79E-08	8.4339

Gene ID	Gene Symbol	FC	P.Value	adj.P.Val	B
ENSG00000140623	SEPTIN12	1.0608	1.81E-08	6.80E-08	8.4313
ENSG00000175600	SUGCT	1.1235	1.82E-08	6.81E-08	8.4307
ENSG00000248643	RBM14-RBM4	1.2505	1.82E-08	6.81E-08	8.4293
ENSG00000177853	ZNF518A	1.3571	1.82E-08	6.83E-08	8.4263
ENSG00000171368	TPPP	1.3218	1.83E-08	6.86E-08	8.4231
ENSG00000173530	TNFRSF10D	1.4222	1.83E-08	6.86E-08	8.4221
ENSG00000167131	CCDC103	1.1371	1.84E-08	6.89E-08	8.4172
ENSG00000188566	NDOR1	1.2604	1.87E-08	6.99E-08	8.4039
ENSG00000104853	CLPTM1	1.2213	1.87E-08	7.00E-08	8.4020
ENSG00000101361	NOP56	1.3220	1.87E-08	7.01E-08	8.4005
ENSG00000087053	MTMR2	1.2630	1.87E-08	7.01E-08	8.4003
ENSG00000185825	BCAP31	1.2947	1.87E-08	7.01E-08	8.3995
ENSG00000117620	SLC35A3	1.2387	1.88E-08	7.02E-08	8.3978
ENSG00000197579	TOPORS	1.2442	1.89E-08	7.05E-08	8.3938
ENSG00000213719	CLIC1	1.2546	1.89E-08	7.07E-08	8.3912
ENSG00000110011	DNAJC4	0.7964	1.89E-08	7.08E-08	8.3902
ENSG00000169752	NRG4	1.3863	1.91E-08	7.14E-08	8.3818
ENSG00000137267	TUBB2A	1.7026	1.95E-08	7.30E-08	8.3592
ENSG00000135114	OASL	1.6009	1.97E-08	7.36E-08	8.3515
ENSG00000171310	CHST11	1.3808	1.98E-08	7.41E-08	8.3444
ENSG00000080815	PSEN1	1.2251	1.99E-08	7.44E-08	8.3402
ENSG00000164713	BRI3	1.3120	1.99E-08	7.44E-08	8.3401
ENSG00000115839	RAB3GAP1	1.1947	2.00E-08	7.46E-08	8.3381
ENSG00000068976	PYGM	1.3717	2.00E-08	7.46E-08	8.3379
ENSG00000168078	PBK	1.4768	2.00E-08	7.46E-08	8.3373
ENSG00000204071	TCEAL6	1.0511	2.00E-08	7.48E-08	8.3346
ENSG00000145041	DCAF1	1.2486	2.01E-08	7.50E-08	8.3318
ENSG00000166986	MARS1	1.2448	2.02E-08	7.52E-08	8.3282
ENSG00000184860	SDR42E1	1.3255	2.04E-08	7.62E-08	8.3161
ENSG00000143842	SOX13	1.0959	2.05E-08	7.63E-08	8.3141
ENSG00000154252	GAL3ST2	1.0565	2.05E-08	7.63E-08	8.3141
ENSG00000163833	FBXO40	1.1281	2.05E-08	7.63E-08	8.3135
ENSG00000149243	KLHL35	1.1760	2.05E-08	7.64E-08	8.3119
ENSG00000054392	HHAT	1.4229	2.07E-08	7.70E-08	8.3047
ENSG00000155189	AGPAT5	1.3802	2.07E-08	7.70E-08	8.3045
ENSG00000140986	RPL3L	1.1359	2.09E-08	7.79E-08	8.2935
ENSG00000172586	CHCHD1	0.7712	2.10E-08	7.80E-08	8.2911
ENSG00000074696	HACD3	0.7045	2.10E-08	7.82E-08	8.2889
ENSG00000115904	SOS1	1.3288	2.11E-08	7.84E-08	8.2858
ENSG00000149972	CNTN5	0.5095	2.11E-08	7.85E-08	8.2843
ENSG00000122126	OCRL	1.3461	2.13E-08	7.93E-08	8.2750
ENSG00000132128	LRRC41	1.2312	2.14E-08	7.98E-08	8.2688
ENSG00000121351	IAPP	1.1227	2.15E-08	7.99E-08	8.2674
ENSG00000003509	NDUFAF7	1.2457	2.17E-08	8.05E-08	8.2595
ENSG00000131480	AOC2	1.2220	2.18E-08	8.09E-08	8.2549
ENSG00000175344	CHRNA7	1.1000	2.18E-08	8.10E-08	8.2527
ENSG00000173641	HSPB7	1.3113	2.18E-08	8.10E-08	8.2525
ENSG00000138758	SEPTIN11	1.4590	2.18E-08	8.10E-08	8.2523
ENSG00000169609	C15orf40	0.8225	2.18E-08	8.11E-08	8.2512
ENSG00000163166	IWS1	1.2063	2.19E-08	8.12E-08	8.2498
ENSG00000134684	YARS1	1.2448	2.19E-08	8.13E-08	8.2482
ENSG00000182264	IZUMO1	1.0770	2.20E-08	8.17E-08	8.2432
ENSG00000176014	TUBB6	1.7265	2.22E-08	8.23E-08	8.2370
ENSG00000160233	LRRC3	1.2988	2.22E-08	8.24E-08	8.2350

Gene ID	Gene Symbol	FC	P.Value	adj.P.Val	B
ENSG00000159753	CARMIL2	1.4562	2.23E-08	8.26E-08	8.2320
ENSG00000165935	SMCO2	1.0933	2.23E-08	8.27E-08	8.2316
ENSG00000227268	KLLN	1.1290	2.23E-08	8.28E-08	8.2303
ENSG00000141994	DUS3L	1.3062	2.24E-08	8.30E-08	8.2278
ENSG00000077238	IL4R	1.6568	2.24E-08	8.30E-08	8.2269
ENSG00000164076	CAMKV	1.0758	2.24E-08	8.32E-08	8.2250
ENSG00000115421	PAPOLG	1.2361	2.26E-08	8.39E-08	8.2164
ENSG00000122952	ZWINT	1.6355	2.27E-08	8.42E-08	8.2128
ENSG00000166012	TAF1D	1.2576	2.29E-08	8.47E-08	8.2062
ENSG00000141429	GALNT1	1.3525	2.32E-08	8.58E-08	8.1943
ENSG00000223573	TINCR	1.1664	2.33E-08	8.64E-08	8.1865
ENSG00000007264	MATK	1.1888	2.34E-08	8.66E-08	8.1850
ENSG00000182327	GLTPD2	1.2772	2.35E-08	8.69E-08	8.1812
ENSG00000130595	TNNT3	1.6590	2.35E-08	8.69E-08	8.1806
ENSG00000197181	PIWIL2	1.0960	2.36E-08	8.74E-08	8.1755
ENSG00000101162	TUBB1	1.2201	2.37E-08	8.78E-08	8.1706
ENSG00000166349	RAG1	1.1591	2.38E-08	8.81E-08	8.1673
ENSG00000166603	MC4R	0.4407	2.39E-08	8.83E-08	8.1643
ENSG00000073331	ALPK1	1.3126	2.40E-08	8.87E-08	8.1599
ENSG00000187889	FYB2	1.1834	2.40E-08	8.88E-08	8.1592
ENSG00000132297	HHLA1	1.2453	2.40E-08	8.88E-08	8.1585
ENSG00000156958	GALK2	0.7925	2.40E-08	8.88E-08	8.1586
ENSG00000137672	TRPC6	1.1445	2.41E-08	8.90E-08	8.1564
ENSG00000161956	SENP3	1.1862	2.44E-08	9.00E-08	8.1453
ENSG00000182287	AP1S2	1.5573	2.45E-08	9.06E-08	8.1383
ENSG00000130638	ATXN10	1.2181	2.45E-08	9.06E-08	8.1380
ENSG00000179912	R3HDM2	1.3144	2.46E-08	9.06E-08	8.1374
ENSG00000179603	GRM8	0.7088	2.47E-08	9.10E-08	8.1334
ENSG00000119333	DYNC2I2	1.4710	2.48E-08	9.15E-08	8.1281
ENSG00000204390	HSPA1L	1.1962	2.48E-08	9.15E-08	8.1274
ENSG00000112365	ZBTB24	1.2053	2.49E-08	9.18E-08	8.1247
ENSG00000128965	CHAC1	0.5378	2.51E-08	9.25E-08	8.1172
ENSG00000121413	ZSCAN18	2.0401	2.51E-08	9.25E-08	8.1167
ENSG00000149182	ARFGAP2	1.2130	2.52E-08	9.29E-08	8.1127
ENSG00000101222	SPEF1	1.1230	2.55E-08	9.39E-08	8.1017
ENSG00000173409	ARV1	0.7238	2.56E-08	9.42E-08	8.0987
ENSG00000104219	ZDHHC2	1.3865	2.60E-08	9.56E-08	8.0838
ENSG00000128590	DNAJB9	0.7638	2.60E-08	9.56E-08	8.0832
ENSG00000232838	PET117	1.2226	2.60E-08	9.58E-08	8.0816
ENSG00000186020	ZNF529	1.3256	2.61E-08	9.60E-08	8.0793
ENSG00000139263	LRIG3	1.7082	2.61E-08	9.61E-08	8.0780
ENSG00000248487	ABHD14A	0.7515	2.61E-08	9.62E-08	8.0770
ENSG00000168806	LCMT2	0.7252	2.62E-08	9.65E-08	8.0737
ENSG00000179933	C14orf119	0.7311	2.63E-08	9.67E-08	8.0710
ENSG00000170855	TRIAP1	1.2142	2.64E-08	9.70E-08	8.0685
ENSG00000174206	KICS2	1.2488	2.64E-08	9.70E-08	8.0677
ENSG00000196689	TRPV1	1.2282	2.64E-08	9.72E-08	8.0658
ENSG00000163380	LMOD3	1.0617	2.65E-08	9.75E-08	8.0629
ENSG00000030110	BAK1	1.3140	2.68E-08	9.84E-08	8.0534
ENSG00000162729	IGSF8	1.3218	2.69E-08	9.88E-08	8.0497
ENSG00000158863	FHIP2B	1.3473	2.69E-08	9.88E-08	8.0492
ENSG00000105278	ZFR2	1.0871	2.69E-08	9.89E-08	8.0475
ENSG00000135643	KCNMB4	1.1707	2.70E-08	9.91E-08	8.0454
ENSG00000111726	CMAS	1.2642	2.70E-08	9.93E-08	8.0439

Gene ID	Gene Symbol	FC	P.Value	adj.P.Val	B
ENSG00000178852	EFCAB13	1.3710	2.70E-08	9.93E-08	8.0438
ENSG00000164199	ADGRV1	1.3645	2.71E-08	9.95E-08	8.0413
ENSG00000137285	TUBB2B	1.5766	2.72E-08	9.96E-08	8.0399
ENSG00000253251	SHLD3	1.2353	2.72E-08	9.99E-08	8.0367
ENSG00000154545	MAGED4	1.9041	2.73E-08	1.00E-07	8.0351
ENSG00000150281	CTF1	1.1603	2.76E-08	1.01E-07	8.0225
ENSG00000178974	FBXO34	1.2312	2.79E-08	1.02E-07	8.0142
ENSG00000135378	PRRG4	1.2994	2.79E-08	1.02E-07	8.0132
ENSG00000100084	HIRA	1.2159	2.81E-08	1.03E-07	8.0053
ENSG00000100605	ITPK1	1.3589	2.82E-08	1.03E-07	8.0031
ENSG00000139547	RDH16	1.1357	2.83E-08	1.04E-07	8.0007
ENSG00000169224	GCSAML	1.1998	2.83E-08	1.04E-07	7.9988
ENSG00000186187	ZNRF1	1.4556	2.84E-08	1.04E-07	7.9962
ENSG00000023228	NDUFS1	1.2399	2.85E-08	1.04E-07	7.9945
ENSG00000073921	PICALM	1.2292	2.86E-08	1.05E-07	7.9905
ENSG00000157404	KIT	0.4522	2.86E-08	1.05E-07	7.9884
ENSG00000048471	SNX29	1.2788	2.87E-08	1.05E-07	7.9857
ENSG00000115241	PPM1G	1.2006	2.89E-08	1.06E-07	7.9804
ENSG00000146094	DOK3	0.6572	2.89E-08	1.06E-07	7.9777
ENSG00000182742	HOXB4	0.7651	2.90E-08	1.06E-07	7.9744
ENSG00000132300	PTCD3	1.2847	2.93E-08	1.07E-07	7.9653
ENSG00000122565	CBX3	1.1954	2.94E-08	1.08E-07	7.9618
ENSG00000163618	CADPS	1.4405	2.95E-08	1.08E-07	7.9601
ENSG00000128276	RFPL3	1.1394	2.96E-08	1.08E-07	7.9546
ENSG00000142687	KIAA0319L	1.2448	2.97E-08	1.08E-07	7.9545
ENSG00000101882	NKAP	1.2457	2.97E-08	1.08E-07	7.9536
ENSG00000083520	DIS3	1.2312	2.98E-08	1.09E-07	7.9510
ENSG00000135211	TMEM60	0.7670	2.98E-08	1.09E-07	7.9497
ENSG00000183628	DGCR6	1.5387	2.98E-08	1.09E-07	7.9491
ENSG00000243678	NME2	0.8232	3.01E-08	1.10E-07	7.9397
ENSG00000131462	TUBG1	1.3286	3.02E-08	1.10E-07	7.9362
ENSG00000100029	PES1	1.2411	3.02E-08	1.10E-07	7.9359
ENSG00000108654	DDX5	1.2919	3.03E-08	1.11E-07	7.9319
ENSG00000063241	ISOC2	0.7606	3.04E-08	1.11E-07	7.9313
ENSG00000162946	DISC1	1.3159	3.04E-08	1.11E-07	7.9313
ENSG00000196230	TUBB	1.3929	3.04E-08	1.11E-07	7.9292
ENSG00000198954	KIFBP	1.2870	3.04E-08	1.11E-07	7.9293
ENSG00000162892	IL24	1.4244	3.05E-08	1.11E-07	7.9281
ENSG00000225366	TDGF1P3	1.0259	3.05E-08	1.11E-07	7.9274
ENSG00000244687	UBE2V1	1.1407	3.08E-08	1.12E-07	7.9170
ENSG00000177947	ODF3	1.0656	3.09E-08	1.12E-07	7.9151
ENSG00000132589	FLOT2	1.2690	3.09E-08	1.13E-07	7.9139
ENSG00000203727	SAMD5	1.0882	3.10E-08	1.13E-07	7.9118
ENSG00000166173	LARP6	1.5493	3.10E-08	1.13E-07	7.9111
ENSG00000124279	FASTKD3	0.7418	3.11E-08	1.13E-07	7.9085
ENSG00000007129	CEACAM21	1.5805	3.11E-08	1.13E-07	7.9075
ENSG00000101294	HM13	1.2385	3.11E-08	1.13E-07	7.9073
ENSG00000166394	CYB5R2	1.5397	3.13E-08	1.14E-07	7.9030
ENSG00000039987	BEST2	1.1571	3.14E-08	1.14E-07	7.8988
ENSG00000162636	FAM102B	1.3022	3.16E-08	1.15E-07	7.8938
ENSG00000196208	GREB1	1.4817	3.16E-08	1.15E-07	7.8939
ENSG00000070182	SPTB	1.4760	3.16E-08	1.15E-07	7.8919
ENSG00000181004	BBS12	1.2725	3.17E-08	1.15E-07	7.8910
ENSG00000239789	MRPS17	0.7825	3.23E-08	1.17E-07	7.8716

Gene ID	Gene Symbol	FC	P.Value	adj.P.Val	B
ENSG00000121058	COIL	1.2324	3.24E-08	1.18E-07	7.8685
ENSG00000144642	RBMS3	1.3451	3.24E-08	1.18E-07	7.8685
ENSG00000142513	ACP4	1.1439	3.24E-08	1.18E-07	7.8674
ENSG00000185842	DNAH14	1.5564	3.26E-08	1.18E-07	7.8632
ENSG00000177125	ZBTB34	1.2689	3.30E-08	1.20E-07	7.8513
ENSG00000090266	NDUFB2	0.7889	3.30E-08	1.20E-07	7.8498
ENSG00000148634	HERC4	1.2539	3.31E-08	1.20E-07	7.8489
ENSG00000177963	RIC8A	1.2028	3.39E-08	1.23E-07	7.8245
ENSG00000171631	P2RY6	0.5223	3.42E-08	1.24E-07	7.8173
ENSG00000163959	SLC51A	1.2318	3.42E-08	1.24E-07	7.8158
ENSG00000171703	TCEA2	1.3098	3.42E-08	1.24E-07	7.8159
ENSG00000204394	VARS1	1.2768	3.42E-08	1.24E-07	7.8146
ENSG00000176723	ZNF843	1.0688	3.43E-08	1.24E-07	7.8142
ENSG00000197119	SLC25A29	1.5496	3.44E-08	1.25E-07	7.8090
ENSG00000187862	TTC24	1.1286	3.50E-08	1.27E-07	7.7926
ENSG00000101546	RBFA	1.3833	3.51E-08	1.27E-07	7.7908
ENSG00000185651	UBE2L3	1.1910	3.51E-08	1.27E-07	7.7895
ENSG00000173581	CCDC106	0.6721	3.53E-08	1.28E-07	7.7866
ENSG00000196381	ZNF781	1.3510	3.53E-08	1.28E-07	7.7851
ENSG00000113905	HRG	1.1241	3.54E-08	1.28E-07	7.7818
ENSG00000172534	HCFC1	1.2726	3.59E-08	1.30E-07	7.7680
ENSG00000212124	TAS2R19	1.0909	3.60E-08	1.30E-07	7.7671
ENSG00000167178	ISLR2	0.6216	3.60E-08	1.30E-07	7.7662
ENSG00000143420	ENSA	1.2497	3.60E-08	1.30E-07	7.7654
ENSG00000101255	TRIB3	1.4645	3.62E-08	1.31E-07	7.7604
ENSG00000213799	ZNF845	1.2457	3.62E-08	1.31E-07	7.7604
ENSG00000187871	GFRAL	1.0211	3.62E-08	1.31E-07	7.7596
ENSG00000130433	CACNG6	2.3956	3.63E-08	1.31E-07	7.7593
ENSG00000094804	CDC6	1.5007	3.63E-08	1.31E-07	7.7574
ENSG00000137817	PARP6	1.2208	3.65E-08	1.32E-07	7.7526
ENSG00000135144	DTX1	1.8830	3.65E-08	1.32E-07	7.7523
ENSG00000177666	PNPLA2	1.2977	3.66E-08	1.32E-07	7.7515
ENSG00000012048	BRCA1	1.3626	3.67E-08	1.33E-07	7.7468
ENSG00000164169	PRMT9	1.2327	3.69E-08	1.33E-07	7.7433
ENSG00000163995	ABLIM2	1.1478	3.69E-08	1.33E-07	7.7424
ENSG00000155034	FBXL18	1.1852	3.70E-08	1.33E-07	7.7408
ENSG00000135473	PAN2	1.4163	3.70E-08	1.33E-07	7.7405
ENSG00000186998	EMID1	1.3313	3.75E-08	1.35E-07	7.7275
ENSG00000204669	C9orf57	1.0877	3.75E-08	1.35E-07	7.7268
ENSG00000036672	USP2	1.6359	3.75E-08	1.35E-07	7.7262
ENSG00000168476	REEP4	1.3048	3.76E-08	1.36E-07	7.7234
ENSG00000205250	E2F4	1.2414	3.80E-08	1.37E-07	7.7134
ENSG00000198932	GPRASP1	1.6954	3.86E-08	1.39E-07	7.6974
ENSG00000177613	CSTF2T	1.2933	3.87E-08	1.40E-07	7.6951
ENSG00000108799	EZH1	1.2999	3.89E-08	1.40E-07	7.6902
ENSG00000107560	RAB11FIP2	1.3230	3.91E-08	1.41E-07	7.6856
ENSG00000104960	PTOV1	1.2269	3.92E-08	1.41E-07	7.6832
ENSG00000183808	RBM12B	1.3642	3.95E-08	1.42E-07	7.6770
ENSG00000006757	PNPLA4	0.7533	3.97E-08	1.43E-07	7.6722
ENSG00000162585	FAAP20	1.2221	4.01E-08	1.44E-07	7.6615
ENSG00000150316	CWC15	1.1914	4.03E-08	1.45E-07	7.6580
ENSG00000101746	NOL4	0.4949	4.04E-08	1.45E-07	7.6545
ENSG00000151276	MAGI1	1.4849	4.06E-08	1.46E-07	7.6501
ENSG00000169857	AVEN	0.7825	4.08E-08	1.47E-07	7.6446

Gene ID	Gene Symbol	FC	P.Value	adj.P.Val	B
ENSG00000128886	ELL3	1.2530	4.09E-08	1.47E-07	7.6427
ENSG00000163507	CIP2A	1.4265	4.10E-08	1.47E-07	7.6400
ENSG00000134107	BHLHE40	1.9755	4.11E-08	1.48E-07	7.6371
ENSG00000157765	SLC34A2	1.1314	4.12E-08	1.48E-07	7.6364
ENSG00000078699	CBFA2T2	1.3322	4.12E-08	1.48E-07	7.6353
ENSG00000115641	FHL2	1.2167	4.12E-08	1.48E-07	7.6343
ENSG00000230797	YY2	1.2033	4.18E-08	1.50E-07	7.6222
ENSG00000152601	MBNL1	1.2949	4.20E-08	1.51E-07	7.6159
ENSG00000162366	PDZK1IP1	0.6269	4.21E-08	1.51E-07	7.6147
ENSG00000106261	ZKSCAN1	1.2505	4.21E-08	1.51E-07	7.6135
ENSG00000163874	ZC3H12A	1.6509	4.21E-08	1.51E-07	7.6134
ENSG00000173253	DMRT2	0.4635	4.22E-08	1.51E-07	7.6126
ENSG00000197951	ZNF71	1.2903	4.23E-08	1.52E-07	7.6108
ENSG00000172058	SERF1A	1.2631	4.25E-08	1.52E-07	7.6050
ENSG00000116198	CEP104	1.3001	4.28E-08	1.54E-07	7.5974
ENSG00000126603	GLIS2	1.1567	4.29E-08	1.54E-07	7.5972
ENSG00000159337	PLA2G4D	1.0675	4.30E-08	1.54E-07	7.5943
ENSG00000131467	PSME3	1.2123	4.30E-08	1.54E-07	7.5934
ENSG00000185615	PDIA2	2.1495	4.34E-08	1.55E-07	7.5860
ENSG00000122591	FAM126A	1.4808	4.34E-08	1.56E-07	7.5841
ENSG00000140650	PMM2	1.2241	4.36E-08	1.56E-07	7.5816
ENSG00000182749	PAQR7	1.2459	4.42E-08	1.58E-07	7.5670
ENSG00000122515	ZMIZ2	1.3691	4.43E-08	1.59E-07	7.5643
ENSG00000235034	C19orf81	2.0634	4.46E-08	1.60E-07	7.5575
ENSG00000197345	MRPL21	0.8291	4.47E-08	1.60E-07	7.5573
ENSG00000076928	ARHGEF1	1.2743	4.51E-08	1.61E-07	7.5471
ENSG00000089159	PXN	1.3648	4.53E-08	1.62E-07	7.5435
ENSG00000166317	SYNPO2L	1.0737	4.53E-08	1.62E-07	7.5429
ENSG00000255073	ZFP91-CNTF	1.1317	4.57E-08	1.64E-07	7.5344
ENSG00000196372	ASB13	1.4227	4.59E-08	1.64E-07	7.5317
ENSG00000165115	KIF27	1.2190	4.59E-08	1.64E-07	7.5314
ENSG00000172260	NEGR1	1.1997	4.59E-08	1.64E-07	7.5310
ENSG00000151458	ANKRD50	1.3425	4.61E-08	1.65E-07	7.5275
ENSG00000205129	C4orf47	1.4701	4.67E-08	1.67E-07	7.5148
ENSG00000116906	GNPAT	1.2407	4.69E-08	1.67E-07	7.5106
ENSG00000100023	PPIL2	1.2490	4.70E-08	1.68E-07	7.5082
ENSG00000133142	TCEAL4	1.3564	4.70E-08	1.68E-07	7.5074
ENSG00000183778	B3GALT5	1.0146	4.71E-08	1.68E-07	7.5048
ENSG00000175573	C11orf68	0.7550	4.72E-08	1.68E-07	7.5037
ENSG00000149575	SCN2B	1.0160	4.73E-08	1.69E-07	7.5013
ENSG00000176732	PFN4	1.2469	4.74E-08	1.69E-07	7.5004
ENSG00000130935	NOL11	1.2294	4.74E-08	1.69E-07	7.4997
ENSG00000185862	EVI2B	0.7411	4.74E-08	1.69E-07	7.4993
ENSG00000227345	PARG	1.2107	4.74E-08	1.69E-07	7.4992
ENSG00000134489	HRH4	1.0465	4.75E-08	1.69E-07	7.4968
ENSG00000100258	LMF2	1.2463	4.76E-08	1.70E-07	7.4959
ENSG00000007968	E2F2	1.6231	4.83E-08	1.72E-07	7.4812
ENSG00000214827	MTCP1	1.3019	4.86E-08	1.73E-07	7.4744
ENSG00000184905	TCEAL2	1.6136	4.87E-08	1.73E-07	7.4742
ENSG00000092203	TOX4	1.2079	4.87E-08	1.74E-07	7.4725
ENSG00000107014	RLN2	0.7117	4.88E-08	1.74E-07	7.4707
ENSG00000204516	MICB	1.2526	4.88E-08	1.74E-07	7.4706
ENSG00000042445	RETSAT	1.2278	4.93E-08	1.75E-07	7.4623
ENSG00000181458	TMEM45A	0.7145	4.95E-08	1.76E-07	7.4574

Gene ID	Gene Symbol	FC	P.Value	adj.P.Val	B
ENSG00000104522	GFUS	1.2670	4.96E-08	1.76E-07	7.4564
ENSG00000168065	SLC22A11	1.0565	4.98E-08	1.77E-07	7.4516
ENSG00000172888	ZNF621	1.2987	5.01E-08	1.78E-07	7.4455
ENSG00000180329	CCDC43	1.2269	5.02E-08	1.79E-07	7.4439
ENSG00000132661	NXT1	1.2277	5.11E-08	1.82E-07	7.4273
ENSG00000167114	SLC27A4	0.7293	5.14E-08	1.83E-07	7.4211
ENSG00000101843	PSMD10	1.2612	5.14E-08	1.83E-07	7.4204
ENSG00000080189	SLC35C2	1.2079	5.19E-08	1.84E-07	7.4121
ENSG00000137135	ARHGEF39	1.3147	5.23E-08	1.86E-07	7.4045
ENSG00000155463	OXA1L	1.1893	5.25E-08	1.87E-07	7.3998
ENSG00000078687	TNRC6C	1.3482	5.27E-08	1.87E-07	7.3959
ENSG00000042062	RIPOR3	1.4750	5.30E-08	1.88E-07	7.3914
ENSG00000213920	MDP1	0.8124	5.31E-08	1.88E-07	7.3903
ENSG00000167671	UBXN6	1.1979	5.38E-08	1.91E-07	7.3767
ENSG00000196455	PIK3R4	1.2776	5.40E-08	1.92E-07	7.3725
ENSG00000071051	NCK2	1.3132	5.42E-08	1.92E-07	7.3696
ENSG00000130147	SH3BP4	1.2202	5.42E-08	1.92E-07	7.3696
ENSG00000198826	ARHGAP11A	1.4763	5.44E-08	1.93E-07	7.3664
ENSG00000138193	PLCE1	1.1870	5.47E-08	1.94E-07	7.3603
ENSG00000147113	DIPK2B	1.0517	5.49E-08	1.95E-07	7.3569
ENSG00000089060	SLC8B1	1.3191	5.51E-08	1.95E-07	7.3541
ENSG00000121454	LHX4	1.2097	5.54E-08	1.96E-07	7.3489
ENSG00000142684	ZNF593	0.7936	5.58E-08	1.98E-07	7.3419
ENSG00000174837	ADGRE1	1.3077	5.58E-08	1.98E-07	7.3415
ENSG00000124120	TTPAL	1.2576	5.59E-08	1.98E-07	7.3404
ENSG00000165113	GKAP1	1.2435	5.59E-08	1.98E-07	7.3402
ENSG00000020129	NCDN	0.7488	5.65E-08	2.00E-07	7.3287
ENSG00000006837	CDKL3	1.1856	5.70E-08	2.02E-07	7.3213
ENSG00000187144	SPATA21	1.2145	5.71E-08	2.02E-07	7.3197
ENSG00000184990	SIVA1	1.2420	5.72E-08	2.02E-07	7.3182
ENSG00000162419	GMEB1	1.2015	5.72E-08	2.02E-07	7.3177
ENSG00000118137	APOA1	1.1717	5.76E-08	2.04E-07	7.3114
ENSG00000166275	BORCS7	0.7964	5.78E-08	2.05E-07	7.3067
ENSG00000168427	KLHL30	1.1105	5.87E-08	2.08E-07	7.2922
ENSG00000167964	RAB26	0.6087	5.88E-08	2.08E-07	7.2904
ENSG00000170500	LONRF2	1.0186	5.91E-08	2.09E-07	7.2865
ENSG00000126804	ZBTB1	1.2574	5.91E-08	2.09E-07	7.2853
ENSG00000166471	TMEM41B	1.2353	5.92E-08	2.09E-07	7.2840
ENSG00000183876	ARSI	1.1119	5.96E-08	2.10E-07	7.2783
ENSG00000071539	TRIP13	1.3782	5.97E-08	2.11E-07	7.2761
ENSG00000171451	DSEL	1.3914	6.00E-08	2.12E-07	7.2718
ENSG00000047249	ATP6V1H	1.2256	6.15E-08	2.17E-07	7.2479
ENSG00000138138	ATAD1	1.2081	6.16E-08	2.17E-07	7.2462
ENSG00000175895	PLEKHF2	1.6066	6.18E-08	2.18E-07	7.2430
ENSG00000116691	MIIP	1.2684	6.20E-08	2.19E-07	7.2390
ENSG00000005059	MCUB	1.3496	6.21E-08	2.19E-07	7.2372
ENSG00000160551	TAOK1	1.2742	6.26E-08	2.21E-07	7.2302
ENSG00000075223	SEMA3C	1.2113	6.28E-08	2.21E-07	7.2274
ENSG00000100348	TXN2	0.8446	6.30E-08	2.22E-07	7.2239
ENSG00000015592	STMN4	1.0460	6.33E-08	2.23E-07	7.2199
ENSG00000119661	DNAL1	1.2208	6.33E-08	2.23E-07	7.2198
ENSG00000158691	ZSCAN12	1.2123	6.33E-08	2.23E-07	7.2189
ENSG00000171435	KSR2	1.0569	6.33E-08	2.23E-07	7.2190
ENSG00000008086	CDKL5	1.2429	6.39E-08	2.25E-07	7.2105

Gene ID	Gene Symbol	FC	P.Value	adj.P.Val	B
ENSG00000136750	GAD2	1.0152	6.48E-08	2.28E-07	7.1965
ENSG00000169359	SLC33A1	0.7337	6.48E-08	2.28E-07	7.1963
ENSG00000134153	EMC7	0.8217	6.56E-08	2.31E-07	7.1848
ENSG00000161509	GRIN2C	1.1879	6.58E-08	2.32E-07	7.1816
ENSG00000188051	TMEM221	1.0768	6.60E-08	2.32E-07	7.1785
ENSG00000167895	TMC8	2.0882	6.61E-08	2.32E-07	7.1778
ENSG00000170382	LRRN2	0.5990	6.65E-08	2.34E-07	7.1710
ENSG00000153113	CAST	1.2677	6.67E-08	2.35E-07	7.1687
ENSG00000013016	EHD3	1.8467	6.70E-08	2.36E-07	7.1646
ENSG00000149742	SLC22A9	1.3405	6.73E-08	2.37E-07	7.1596
ENSG00000168685	IL7R	1.2211	6.79E-08	2.39E-07	7.1514
ENSG00000204536	CCHCR1	1.3647	6.84E-08	2.40E-07	7.1440
ENSG00000130413	STK33	1.3743	6.85E-08	2.41E-07	7.1432
ENSG00000049167	ERCC8	0.7995	6.93E-08	2.43E-07	7.1316
ENSG00000159648	TEPP	1.0712	6.96E-08	2.44E-07	7.1278
ENSG00000144815	NXPE3	1.5248	6.96E-08	2.45E-07	7.1271
ENSG00000112357	PEX7	0.7945	6.98E-08	2.45E-07	7.1242
ENSG00000119705	SLIRP	0.8194	6.99E-08	2.46E-07	7.1226
ENSG00000149089	APIP	0.7599	7.04E-08	2.47E-07	7.1161
ENSG00000177200	CHD9	1.3075	7.05E-08	2.47E-07	7.1155
ENSG00000203780	FANK1	1.1295	7.07E-08	2.48E-07	7.1117
ENSG00000010322	NISCH	1.2475	7.13E-08	2.50E-07	7.1044
ENSG00000196418	ZNF124	1.2351	7.15E-08	2.51E-07	7.1010
ENSG00000161265	U2AF1L4	1.2611	7.17E-08	2.51E-07	7.0986
ENSG00000261408	TEN1-CDK3	1.2384	7.19E-08	2.52E-07	7.0962
ENSG00000112137	PHACTR1	1.9786	7.24E-08	2.54E-07	7.0886
ENSG00000123989	CHPF	1.3310	7.25E-08	2.54E-07	7.0879
ENSG00000198589	LRBA	1.2419	7.26E-08	2.55E-07	7.0858
ENSG00000143224	PPOX	1.3266	7.28E-08	2.55E-07	7.0831
ENSG00000101680	LAMA1	1.0666	7.29E-08	2.55E-07	7.0826
ENSG00000168615	ADAM9	1.5412	7.34E-08	2.57E-07	7.0759
ENSG00000117525	F3	1.3029	7.43E-08	2.60E-07	7.0642
ENSG00000155026	RSPH10B	1.1515	7.43E-08	2.60E-07	7.0643
ENSG00000165171	METTL27	1.6290	7.43E-08	2.60E-07	7.0645
ENSG00000112874	NUDT12	0.5983	7.44E-08	2.60E-07	7.0632
ENSG00000108309	RUNDC3A	1.2941	7.46E-08	2.61E-07	7.0602
ENSG00000077044	DGKD	1.4747	7.48E-08	2.62E-07	7.0575
ENSG00000154957	ZNF18	1.2414	7.58E-08	2.65E-07	7.0443
ENSG00000108242	CYP2C18	1.0239	7.61E-08	2.66E-07	7.0414
ENSG00000106004	HOXA5	0.6345	7.73E-08	2.70E-07	7.0251
ENSG00000188603	CLN3	1.2355	7.80E-08	2.73E-07	7.0173
ENSG00000205707	ETFRF1	0.7782	7.80E-08	2.73E-07	7.0174
ENSG00000002746	HECW1	1.2301	7.86E-08	2.75E-07	7.0096
ENSG00000187658	C5orf52	0.8764	7.87E-08	2.75E-07	7.0077
ENSG00000105443	CYTH2	1.2054	7.90E-08	2.76E-07	7.0041
ENSG00000105290	APLP1	1.4896	7.92E-08	2.77E-07	7.0021
ENSG00000131037	EPS8L1	1.6006	7.93E-08	2.77E-07	7.0011
ENSG00000198912	C1orf174	1.2073	7.95E-08	2.78E-07	6.9985
ENSG00000132763	MMACHC	0.7353	7.96E-08	2.78E-07	6.9978
ENSG00000107362	ABHD17B	1.2764	8.04E-08	2.80E-07	6.9881
ENSG00000168256	NKIRAS2	1.2270	8.06E-08	2.81E-07	6.9851
ENSG00000150753	CCT5	0.6707	8.10E-08	2.82E-07	6.9809
ENSG00000198721	ECI2	0.7566	8.10E-08	2.83E-07	6.9800
ENSG00000160683	CXCR5	1.3016	8.11E-08	2.83E-07	6.9792

Gene ID	Gene Symbol	FC	P.Value	adj.P.Val	B
ENSG00000157193	LRP8	1.4107	8.13E-08	2.83E-07	6.9772
ENSG00000213265	TSGA13	1.0418	8.17E-08	2.85E-07	6.9725
ENSG00000156253	RWDD2B	0.7606	8.17E-08	2.85E-07	6.9719
ENSG00000054277	OPN3	1.4737	8.20E-08	2.86E-07	6.9683
ENSG00000090863	GLG1	1.3296	8.25E-08	2.88E-07	6.9622
ENSG00000165689	ENTR1	0.7563	8.31E-08	2.90E-07	6.9551
ENSG00000233701	PRR23C	1.0325	8.38E-08	2.92E-07	6.9470
ENSG00000214435	AS3MT	1.1741	8.44E-08	2.94E-07	6.9408
ENSG00000138756	BMP2K	1.6296	8.45E-08	2.94E-07	6.9392
ENSG00000184014	DENND5A	1.5920	8.48E-08	2.95E-07	6.9355
ENSG00000119522	DENND1A	1.2088	8.50E-08	2.96E-07	6.9342
ENSG00000163029	SMC6	1.2825	8.55E-08	2.97E-07	6.9282
ENSG00000103723	AP3B2	1.2773	8.57E-08	2.98E-07	6.9256
ENSG00000148225	WDR31	1.1877	8.60E-08	2.99E-07	6.9219
ENSG00000170448	NFXL1	1.2230	8.62E-08	3.00E-07	6.9198
ENSG00000129534	MIS18BP1	1.3009	8.68E-08	3.02E-07	6.9140
ENSG00000175063	UBE2C	1.6046	8.68E-08	3.02E-07	6.9130
ENSG00000156500	PABIR3	1.2295	8.71E-08	3.03E-07	6.9099
ENSG00000101194	SLC17A9	1.4823	8.72E-08	3.03E-07	6.9095
ENSG00000234409	CCDC188	1.1854	8.75E-08	3.04E-07	6.9060
ENSG00000072756	TRNT1	0.7803	8.81E-08	3.06E-07	6.8994
ENSG00000086189	DIMT1	0.8185	8.81E-08	3.06E-07	6.8988
ENSG00000139946	PELI2	1.3886	8.84E-08	3.07E-07	6.8960
ENSG00000187492	CDHR4	1.1576	8.87E-08	3.08E-07	6.8923
ENSG00000051620	HEBP2	1.2493	8.88E-08	3.08E-07	6.8919
ENSG00000134186	PRPF38B	1.2255	8.89E-08	3.09E-07	6.8903
ENSG00000034053	APBA2	1.3945	8.91E-08	3.09E-07	6.8879
ENSG00000001626	CFTR	1.3046	8.92E-08	3.09E-07	6.8871
ENSG00000160767	FAM189B	1.2908	8.93E-08	3.10E-07	6.8864
ENSG00000197283	SYNGAP1	1.3937	8.94E-08	3.10E-07	6.8849
ENSG00000103522	IL21R	1.3468	9.04E-08	3.13E-07	6.8742
ENSG00000174898	CATSPERD	1.0938	9.04E-08	3.13E-07	6.8739
ENSG00000005700	IBTK	1.2480	9.07E-08	3.14E-07	6.8709
ENSG00000069764	PLA2G10	1.1947	9.09E-08	3.15E-07	6.8688
ENSG00000168610	STAT3	1.3861	9.13E-08	3.16E-07	6.8649
ENSG00000111665	CDCA3	1.4013	9.16E-08	3.17E-07	6.8612
ENSG00000170043	TRAPPC1	1.2993	9.17E-08	3.18E-07	6.8603
ENSG00000247077	PGAM5	1.2369	9.24E-08	3.20E-07	6.8532
ENSG00000175265	GOLGA8A	1.4729	9.26E-08	3.21E-07	6.8512
ENSG00000162105	SHANK2	1.4085	9.28E-08	3.21E-07	6.8488
ENSG00000169221	TBC1D10B	1.2432	9.33E-08	3.23E-07	6.8431
ENSG00000177602	HASPIN	1.3130	9.50E-08	3.29E-07	6.8258
ENSG00000171208	NETO2	1.5946	9.63E-08	3.33E-07	6.8129
ENSG00000139436	GIT2	1.2616	9.64E-08	3.33E-07	6.8122
ENSG00000125970	RALY	1.3026	9.64E-08	3.33E-07	6.8117
ENSG00000196352	CD55	1.3953	9.79E-08	3.39E-07	6.7967
ENSG00000049769	PPP1R3F	1.3581	9.80E-08	3.39E-07	6.7961
ENSG00000136059	VILL	0.8031	9.82E-08	3.39E-07	6.7940
ENSG00000074603	DPP8	1.2028	9.85E-08	3.40E-07	6.7910
ENSG00000172349	IL16	0.6419	9.85E-08	3.41E-07	6.7906
ENSG00000167194	C16orf92	1.0979	1.00E-07	3.46E-07	6.7753
ENSG00000162341	TPCN2	1.2665	1.01E-07	3.50E-07	6.7624
ENSG00000135766	EGLN1	1.2770	1.02E-07	3.51E-07	6.7599
ENSG00000125779	PANK2	1.2050	1.02E-07	3.52E-07	6.7570

Gene ID	Gene Symbol	FC	P.Value	adj.P.Val	B
ENSG00000136146	MED4	1.2051	1.03E-07	3.54E-07	6.7510
ENSG00000215252	GOLGA8B	1.4270	1.03E-07	3.56E-07	6.7476
ENSG00000204463	BAG6	1.2028	1.03E-07	3.56E-07	6.7458
ENSG00000160563	MED27	1.1735	1.03E-07	3.57E-07	6.7439
ENSG00000006695	COX10	1.2262	1.04E-07	3.59E-07	6.7378
ENSG00000138835	RGS3	0.6539	1.05E-07	3.61E-07	6.7322
ENSG00000121481	RNF2	1.2432	1.05E-07	3.63E-07	6.7270
ENSG00000164434	FABP7	1.0324	1.05E-07	3.64E-07	6.7246
ENSG00000138617	PARP16	0.7789	1.06E-07	3.65E-07	6.7198
ENSG00000105088	OLFM2	1.1024	1.07E-07	3.68E-07	6.7142
ENSG00000176444	CLK2	1.3291	1.07E-07	3.70E-07	6.7086
ENSG00000197136	PCNX3	1.2319	1.07E-07	3.70E-07	6.7077
ENSG00000160791	CCR5	0.5707	1.08E-07	3.70E-07	6.7062
ENSG00000152409	JMY	1.3645	1.08E-07	3.71E-07	6.7050
ENSG00000140543	DET1	0.7155	1.08E-07	3.72E-07	6.7006
ENSG00000167977	KCTD5	1.2173	1.08E-07	3.72E-07	6.7007
ENSG00000176142	TMEM39A	1.2235	1.08E-07	3.73E-07	6.6976
ENSG00000114923	SLC4A3	1.0881	1.09E-07	3.74E-07	6.6967
ENSG00000145451	GLRA3	1.1272	1.09E-07	3.74E-07	6.6967
ENSG00000175697	GPR156	1.0918	1.09E-07	3.74E-07	6.6966
ENSG00000130766	SESN2	1.5135	1.09E-07	3.76E-07	6.6907
ENSG00000154319	FAM167A	1.1163	1.11E-07	3.81E-07	6.6779
ENSG00000076356	PLXNA2	1.4739	1.11E-07	3.82E-07	6.6740
ENSG00000140839	CLEC18B	0.7657	1.12E-07	3.84E-07	6.6690
ENSG00000102572	STK24	1.2620	1.12E-07	3.85E-07	6.6672
ENSG00000169255	B3GALNT1	1.5275	1.12E-07	3.86E-07	6.6637
ENSG00000122483	CCDC18	1.2907	1.14E-07	3.91E-07	6.6515
ENSG00000174851	YIF1A	0.8052	1.14E-07	3.92E-07	6.6485
ENSG00000101213	PTK6	1.2148	1.14E-07	3.93E-07	6.6456
ENSG00000166813	KIF7	1.1924	1.15E-07	3.96E-07	6.6395
ENSG00000099203	TMED1	0.8064	1.16E-07	3.97E-07	6.6355
ENSG00000176401	EID2B	1.2518	1.17E-07	4.02E-07	6.6230
ENSG00000123612	ACVR1C	0.7101	1.17E-07	4.03E-07	6.6205
ENSG00000117020	AKT3	1.4574	1.18E-07	4.04E-07	6.6181
ENSG00000184033	CTAG1B	1.6491	1.18E-07	4.04E-07	6.6177
ENSG00000161960	EIF4A1	1.2016	1.18E-07	4.04E-07	6.6168
ENSG00000213977	TAX1BP3	1.3067	1.18E-07	4.04E-07	6.6169
ENSG00000169964	TMEM42	0.7872	1.18E-07	4.05E-07	6.6153
ENSG00000128915	ICE2	0.7718	1.19E-07	4.07E-07	6.6113
ENSG00000021762	OSBPL5	1.5668	1.20E-07	4.10E-07	6.6036
ENSG00000011009	LYPLA2	1.1893	1.20E-07	4.10E-07	6.6032
ENSG00000186951	PPARA	1.2325	1.20E-07	4.10E-07	6.6029
ENSG00000179387	ELMOD2	1.2437	1.21E-07	4.14E-07	6.5942
ENSG00000214357	NEURL1B	1.1495	1.21E-07	4.15E-07	6.5913
ENSG00000169379	ARL13B	1.2334	1.21E-07	4.15E-07	6.5908
ENSG00000184949	FAM227A	1.1381	1.22E-07	4.17E-07	6.5851
ENSG00000138308	PLA2G12B	1.0442	1.22E-07	4.18E-07	6.5840
ENSG00000204131	NHSL2	1.1471	1.23E-07	4.22E-07	6.5739
ENSG00000183850	ZNF730	1.0696	1.23E-07	4.22E-07	6.5729
ENSG00000171302	CANT1	1.2251	1.24E-07	4.24E-07	6.5698
ENSG00000184203	PPP1R2	1.2607	1.24E-07	4.24E-07	6.5687
ENSG00000102452	NALCN	1.1433	1.25E-07	4.26E-07	6.5632
ENSG00000129214	SHBG	1.1374	1.25E-07	4.27E-07	6.5612
ENSG00000173486	FKBP2	0.7611	1.26E-07	4.31E-07	6.5528

Gene ID	Gene Symbol	FC	P.Value	adj.P.Val	B
ENSG00000156504	PABIR2	1.2857	1.27E-07	4.33E-07	6.5477
ENSG00000178297	TMPRSS9	1.1883	1.27E-07	4.34E-07	6.5451
ENSG00000174996	KLC2	1.2561	1.28E-07	4.37E-07	6.5380
ENSG00000011243	AKAP8L	1.2151	1.28E-07	4.38E-07	6.5367
ENSG00000183137	CEP57L1	0.7698	1.29E-07	4.40E-07	6.5313
ENSG00000004534	RBM6	1.2434	1.29E-07	4.41E-07	6.5284
ENSG00000072315	TRPC5	1.0128	1.30E-07	4.44E-07	6.5228
ENSG00000132912	DCTN4	1.2017	1.30E-07	4.45E-07	6.5200
ENSG00000112983	BRD8	1.3429	1.30E-07	4.45E-07	6.5189
ENSG00000106723	SPIN1	1.2739	1.31E-07	4.47E-07	6.5153
ENSG00000144406	UNC80	1.2081	1.32E-07	4.49E-07	6.5101
ENSG00000173699	SPATA3	1.0609	1.32E-07	4.50E-07	6.5084
ENSG00000109674	NEIL3	1.2297	1.32E-07	4.51E-07	6.5068
ENSG00000134954	ETS1	1.6781	1.33E-07	4.55E-07	6.4971
ENSG00000024526	DEPDC1	1.3864	1.34E-07	4.55E-07	6.4964
ENSG00000198794	SCAMP5	0.6246	1.34E-07	4.56E-07	6.4943
ENSG00000119203	CPSF3	1.2187	1.34E-07	4.57E-07	6.4934
ENSG00000106683	LIMK1	1.2682	1.34E-07	4.57E-07	6.4923
ENSG00000188343	CIBAR1	1.3354	1.35E-07	4.59E-07	6.4884
ENSG00000175985	PLEKHD1	1.0881	1.35E-07	4.59E-07	6.4874
ENSG00000138760	SCARB2	1.2845	1.36E-07	4.62E-07	6.4812
ENSG00000181789	COPG1	1.2071	1.36E-07	4.62E-07	6.4809
ENSG00000111684	LPCAT3	1.2111	1.37E-07	4.67E-07	6.4717
ENSG00000077264	PAK3	1.1638	1.37E-07	4.67E-07	6.4709
ENSG00000151615	POU4F2	1.0500	1.38E-07	4.70E-07	6.4638
ENSG00000131097	HIGD1B	1.1304	1.38E-07	4.71E-07	6.4616
ENSG00000116703	PDC	1.0597	1.39E-07	4.72E-07	6.4607
ENSG00000187860	CCDC157	1.1920	1.39E-07	4.72E-07	6.4597
ENSG00000256229	ZNF486	1.2304	1.40E-07	4.77E-07	6.4486
ENSG00000092964	DPYSL2	1.4310	1.40E-07	4.77E-07	6.4484
ENSG00000155313	USP25	1.3337	1.41E-07	4.79E-07	6.4442
ENSG00000171303	KCNK3	1.0337	1.42E-07	4.82E-07	6.4377
ENSG00000154582	ELOC	1.1582	1.42E-07	4.84E-07	6.4340
ENSG00000138031	ADCY3	1.5192	1.43E-07	4.86E-07	6.4297
ENSG00000156097	GPR61	1.1439	1.44E-07	4.88E-07	6.4255
ENSG00000236609	ZNF853	1.2199	1.44E-07	4.89E-07	6.4237
ENSG00000007392	LUC7L	1.2843	1.44E-07	4.90E-07	6.4225
ENSG00000181467	RAP2B	1.3572	1.45E-07	4.92E-07	6.4172
ENSG00000163519	TRAT1	0.4997	1.45E-07	4.94E-07	6.4141
ENSG00000164171	ITGA2	1.0785	1.46E-07	4.96E-07	6.4087
ENSG00000005801	ZNF195	1.2478	1.46E-07	4.97E-07	6.4076
ENSG00000040341	STAU2	1.2673	1.47E-07	4.99E-07	6.4039
ENSG00000111845	PAK1IP1	0.7414	1.48E-07	5.04E-07	6.3943
ENSG00000189195	BTBD8	1.1036	1.49E-07	5.05E-07	6.3911
ENSG00000127463	EMC1	1.2574	1.49E-07	5.06E-07	6.3892
ENSG00000238227	TMEM250	0.7254	1.51E-07	5.12E-07	6.3786
ENSG00000136141	LRCH1	1.2757	1.51E-07	5.12E-07	6.3782
ENSG00000110074	FOXRED1	1.2396	1.51E-07	5.13E-07	6.3761
ENSG00000090661	CERS4	1.6777	1.52E-07	5.14E-07	6.3743
ENSG00000138180	CEP55	1.4679	1.52E-07	5.15E-07	6.3705
ENSG00000173157	ADAMTS20	1.2577	1.52E-07	5.15E-07	6.3702
ENSG00000172938	MRGPRD	1.0332	1.53E-07	5.20E-07	6.3625
ENSG00000106006	HOXA6	0.7255	1.54E-07	5.20E-07	6.3612
ENSG00000106344	RBM28	1.2740	1.54E-07	5.20E-07	6.3611

Gene ID	Gene Symbol	FC	P.Value	adj.P.Val	B
ENSG00000106263	EIF3B	1.2190	1.54E-07	5.21E-07	6.3586
ENSG00000196440	ARMCX4	1.2630	1.55E-07	5.25E-07	6.3514
ENSG00000129195	PIMREG	1.4429	1.56E-07	5.29E-07	6.3440
ENSG00000143748	NVL	1.2714	1.56E-07	5.29E-07	6.3436
ENSG00000166435	XRRA1	0.7117	1.57E-07	5.30E-07	6.3423
ENSG00000114378	HYAL1	1.0804	1.57E-07	5.30E-07	6.3410
ENSG00000099991	CABIN1	1.2934	1.57E-07	5.32E-07	6.3386
ENSG00000184916	JAG2	1.2071	1.58E-07	5.33E-07	6.3364
ENSG00000138400	MDH1B	1.1609	1.59E-07	5.37E-07	6.3292
ENSG00000162631	NTNG1	1.5100	1.61E-07	5.46E-07	6.3131
ENSG00000115274	INO80B	1.2069	1.62E-07	5.47E-07	6.3107
ENSG00000188817	SNTN	1.0569	1.62E-07	5.48E-07	6.3091
ENSG00000197798	FAM118B	0.7804	1.63E-07	5.50E-07	6.3045
ENSG00000133983	COX16	0.8186	1.63E-07	5.50E-07	6.3038
ENSG00000189013	KIR2DL4	1.2004	1.63E-07	5.50E-07	6.3039
ENSG00000086544	ITPKC	1.2485	1.63E-07	5.51E-07	6.3028
ENSG00000123810	B9D2	1.2884	1.64E-07	5.52E-07	6.3004
ENSG00000155849	ELMO1	1.2498	1.64E-07	5.53E-07	6.2988
ENSG00000133574	GIMAP4	0.7111	1.64E-07	5.53E-07	6.2986
ENSG00000203995	ZYG11A	1.2376	1.64E-07	5.54E-07	6.2967
ENSG00000179950	PUF60	1.2208	1.65E-07	5.55E-07	6.2948
ENSG00000196420	S100A5	1.1373	1.65E-07	5.56E-07	6.2930
ENSG00000004142	POLDIP2	1.2001	1.67E-07	5.64E-07	6.2791
ENSG00000182938	OTOP3	1.0567	1.68E-07	5.66E-07	6.2758
ENSG00000131398	KCNC3	1.2323	1.68E-07	5.68E-07	6.2722
ENSG00000110881	ASIC1	1.0845	1.68E-07	5.68E-07	6.2717
ENSG00000116690	PRG4	1.1287	1.70E-07	5.74E-07	6.2615
ENSG00000131591	C1orf159	1.2039	1.70E-07	5.74E-07	6.2607
ENSG00000089335	ZNF302	0.7364	1.71E-07	5.76E-07	6.2577
ENSG00000100027	YPEL1	1.2708	1.71E-07	5.76E-07	6.2571
ENSG00000062038	CDH3	1.0917	1.72E-07	5.78E-07	6.2542
ENSG00000198792	TMEM184B	1.4176	1.73E-07	5.82E-07	6.2476
ENSG00000151500	THYN1	0.7741	1.73E-07	5.82E-07	6.2471
ENSG00000105968	H2AZ2	1.2494	1.73E-07	5.83E-07	6.2445
ENSG00000175115	PACS1	1.3460	1.73E-07	5.83E-07	6.2444
ENSG00000140848	CPNE2	1.3590	1.73E-07	5.83E-07	6.2441
ENSG00000221955	SLC12A8	1.4846	1.74E-07	5.85E-07	6.2412
ENSG00000137331	IER3	1.8341	1.75E-07	5.89E-07	6.2342
ENSG00000170340	B3GNT2	1.3250	1.76E-07	5.90E-07	6.2317
ENSG00000175592	FOSL1	1.3855	1.76E-07	5.92E-07	6.2297
ENSG00000180875	GREM2	1.0715	1.78E-07	5.98E-07	6.2189
ENSG00000139537	CCDC65	1.2977	1.79E-07	6.01E-07	6.2136
ENSG00000119013	NDUFB3	0.7507	1.81E-07	6.08E-07	6.2033
ENSG00000182185	RAD51B	1.2513	1.81E-07	6.09E-07	6.2009
ENSG00000154447	SH3RF1	1.4177	1.82E-07	6.10E-07	6.1988
ENSG00000152086	TUBA3E	1.0380	1.82E-07	6.12E-07	6.1961
ENSG00000079102	RUNX1T1	1.3475	1.83E-07	6.13E-07	6.1935
ENSG00000169750	RAC3	1.3056	1.84E-07	6.18E-07	6.1868
ENSG00000178537	SLC25A20	0.7033	1.84E-07	6.19E-07	6.1842
ENSG00000108819	PPP1R9B	1.2613	1.85E-07	6.20E-07	6.1827
ENSG00000137815	RTF1	1.2126	1.86E-07	6.23E-07	6.1783
ENSG00000175356	SCUBE2	1.1014	1.86E-07	6.25E-07	6.1750
ENSG00000076864	RAP1GAP	1.3427	1.86E-07	6.25E-07	6.1748
ENSG00000028203	VEZT	1.2199	1.87E-07	6.27E-07	6.1710

Gene ID	Gene Symbol	FC	P.Value	adj.P.Val	B
ENSG00000166262	FAM227B	0.7721	1.87E-07	6.28E-07	6.1699
ENSG00000105371	ICAM4	0.5658	1.88E-07	6.29E-07	6.1679
ENSG00000149483	TMEM138	0.8228	1.88E-07	6.30E-07	6.1664
ENSG00000184305	CCSER1	1.4930	1.88E-07	6.30E-07	6.1656
ENSG00000166863	TAC3	1.0828	1.88E-07	6.31E-07	6.1635
ENSG00000125863	MKKS	0.7944	1.89E-07	6.33E-07	6.1612
ENSG00000198431	TXNRD1	1.2649	1.89E-07	6.33E-07	6.1607
ENSG00000139364	TMEM132B	1.1222	1.91E-07	6.38E-07	6.1521
ENSG00000109705	NKX3-2	0.7159	1.91E-07	6.39E-07	6.1515
ENSG00000171016	PYGO1	1.0746	1.91E-07	6.40E-07	6.1493
ENSG00000006016	CRLF1	1.2194	1.92E-07	6.43E-07	6.1453
ENSG00000100445	SDR39U1	1.2427	1.94E-07	6.50E-07	6.1347
ENSG00000151657	KIN	1.2046	1.94E-07	6.50E-07	6.1340
ENSG00000136448	NMT1	1.2501	1.98E-07	6.62E-07	6.1159
ENSG00000130764	LRRC47	1.2087	1.99E-07	6.64E-07	6.1131
ENSG00000064787	BCAS1	0.6303	1.99E-07	6.66E-07	6.1100
ENSG00000076706	MCAM	1.7472	2.00E-07	6.69E-07	6.1054
ENSG00000130821	SLC6A8	1.3916	2.01E-07	6.71E-07	6.1028
ENSG00000008988	RPS20	0.8357	2.01E-07	6.71E-07	6.1022
ENSG00000127831	VIL1	1.0802	2.02E-07	6.75E-07	6.0969
ENSG00000196154	S100A4	2.3442	2.02E-07	6.76E-07	6.0952
ENSG00000148019	CEP78	1.3289	2.03E-07	6.77E-07	6.0929
ENSG00000140057	AK7	1.1144	2.03E-07	6.78E-07	6.0910
ENSG00000117155	SSX2IP	1.2980	2.03E-07	6.79E-07	6.0901
ENSG00000185669	SNAI3	0.6910	2.04E-07	6.82E-07	6.0846
ENSG00000197324	LRP10	1.2478	2.04E-07	6.82E-07	6.0847
ENSG00000143632	ACTA1	1.2723	2.05E-07	6.83E-07	6.0843
ENSG00000159792	PSKH1	1.2352	2.05E-07	6.85E-07	6.0812
ENSG00000112742	TTK	1.4002	2.06E-07	6.87E-07	6.0774
ENSG00000155792	DEPTOR	1.7643	2.08E-07	6.94E-07	6.0672
ENSG00000141985	SH3GL1	1.2179	2.09E-07	6.96E-07	6.0650
ENSG00000160654	CD3G	1.1142	2.09E-07	6.98E-07	6.0617
ENSG00000116785	CFHR3	1.5350	2.09E-07	6.98E-07	6.0614
ENSG00000167654	ATCAY	1.0533	2.09E-07	6.98E-07	6.0613
ENSG00000188559	RALGAPA2	1.2366	2.10E-07	6.99E-07	6.0602
ENSG00000002726	AOC1	1.5200	2.11E-07	7.02E-07	6.0551
ENSG00000157168	NRG1	1.2323	2.11E-07	7.04E-07	6.0530
ENSG00000173757	STAT5B	1.5329	2.11E-07	7.04E-07	6.0521
ENSG00000215915	ATAD3C	0.6231	2.12E-07	7.05E-07	6.0511
ENSG00000144063	MALL	1.1166	2.12E-07	7.05E-07	6.0508
ENSG00000003436	TFPI	1.3067	2.13E-07	7.09E-07	6.0454
ENSG00000006062	MAP3K14	1.3843	2.13E-07	7.10E-07	6.0436
ENSG00000131864	USP29	1.1877	2.14E-07	7.12E-07	6.0413
ENSG00000221963	APOL6	1.3293	2.14E-07	7.12E-07	6.0407
ENSG00000127870	RNF6	1.2130	2.14E-07	7.13E-07	6.0389
ENSG00000159082	SYNJ1	1.1940	2.15E-07	7.16E-07	6.0351
ENSG00000187944	C2orf66	1.0793	2.16E-07	7.17E-07	6.0338
ENSG00000213413	PVRIG	1.3196	2.16E-07	7.20E-07	6.0296
ENSG00000150054	MPP7	1.1682	2.18E-07	7.26E-07	6.0207
ENSG00000168502	MTCL1	1.4823	2.20E-07	7.31E-07	6.0139
ENSG00000127588	GNG13	1.0421	2.23E-07	7.43E-07	5.9987
ENSG00000136240	KDELR2	1.2175	2.24E-07	7.43E-07	5.9981
ENSG00000184216	IRAK1	1.2730	2.25E-07	7.46E-07	5.9942
ENSG00000123349	PFDN5	0.8650	2.26E-07	7.50E-07	5.9883

Gene ID	Gene Symbol	FC	P.Value	adj.P.Val	B
ENSG00000198948	MFAP3L	0.5250	2.26E-07	7.51E-07	5.9876
ENSG00000112799	LY86	0.5840	2.33E-07	7.72E-07	5.9598
ENSG00000115652	UXS1	1.2468	2.34E-07	7.76E-07	5.9548
ENSG00000180902	D2HGDH	1.3082	2.34E-07	7.76E-07	5.9549
ENSG00000117602	RCAN3	1.3702	2.34E-07	7.77E-07	5.9531
ENSG00000072134	EPN2	1.2621	2.35E-07	7.79E-07	5.9504
ENSG00000151632	AKR1C2	1.1893	2.35E-07	7.80E-07	5.9501
ENSG00000153814	JAZF1	1.3643	2.35E-07	7.81E-07	5.9485
ENSG00000169925	BRD3	1.2503	2.35E-07	7.81E-07	5.9484
ENSG00000169340	PDILT	1.0498	2.36E-07	7.81E-07	5.9473
ENSG00000116161	CACYBP	1.3128	2.36E-07	7.83E-07	5.9446
ENSG00000041357	PSMA4	0.7667	2.39E-07	7.91E-07	5.9356
ENSG00000198049	AVPR1B	1.0893	2.39E-07	7.91E-07	5.9345
ENSG00000234444	ZNF736	1.4658	2.40E-07	7.94E-07	5.9311
ENSG00000213023	SYT3	1.2915	2.40E-07	7.95E-07	5.9298
ENSG00000162630	B3GALT2	1.0242	2.41E-07	7.99E-07	5.9247
ENSG00000101407	TTI1	1.2607	2.42E-07	8.00E-07	5.9235
ENSG00000147164	SNX12	1.2042	2.43E-07	8.04E-07	5.9184
ENSG00000104221	BRF2	1.2100	2.43E-07	8.04E-07	5.9179
ENSG00000115317	HTRA2	1.2121	2.43E-07	8.05E-07	5.9164
ENSG00000022567	SLC45A4	1.2386	2.45E-07	8.10E-07	5.9112
ENSG00000079819	EPB41L2	1.4704	2.45E-07	8.10E-07	5.9101
ENSG00000186567	CEACAM19	1.1970	2.45E-07	8.11E-07	5.9092
ENSG00000160803	UBQLN4	1.2858	2.46E-07	8.13E-07	5.9070
ENSG00000155111	CDK19	1.2772	2.46E-07	8.13E-07	5.9065
ENSG00000124882	EREG	1.5161	2.46E-07	8.14E-07	5.9055
ENSG00000069702	TGFBR3	1.2028	2.47E-07	8.16E-07	5.9030
ENSG00000110888	CAPRIN2	1.2917	2.48E-07	8.19E-07	5.8992
ENSG00000119812	FAM98A	1.2354	2.49E-07	8.23E-07	5.8937
ENSG00000182752	PAPPA	1.1339	2.50E-07	8.26E-07	5.8900
ENSG00000154309	DISP1	1.2018	2.51E-07	8.30E-07	5.8851
ENSG00000163935	SFMBT1	1.2466	2.55E-07	8.42E-07	5.8714
ENSG00000183638	RP1L1	1.1023	2.56E-07	8.45E-07	5.8681
ENSG00000105576	TNPO2	1.2583	2.56E-07	8.45E-07	5.8678
ENSG00000037897	METTL1	1.2748	2.58E-07	8.52E-07	5.8593
ENSG00000188958	UTS2B	1.1589	2.58E-07	8.52E-07	5.8592
ENSG00000100557	CCDC198	1.2120	2.60E-07	8.56E-07	5.8541
ENSG00000125977	EIF2S2	0.8204	2.60E-07	8.56E-07	5.8540
ENSG00000130733	YIPF2	0.8043	2.60E-07	8.56E-07	5.8542
ENSG00000197275	RAD54B	1.3045	2.61E-07	8.60E-07	5.8500
ENSG00000062524	LTK	0.5446	2.61E-07	8.61E-07	5.8484
ENSG00000126970	ZC4H2	1.3176	2.62E-07	8.63E-07	5.8455
ENSG00000152939	MARVELD2	0.7340	2.62E-07	8.64E-07	5.8442
ENSG00000119616	FCF1	1.2286	2.62E-07	8.65E-07	5.8437
ENSG00000197446	CYP2F1	1.0751	2.62E-07	8.65E-07	5.8434
ENSG00000160932	LY6E	2.1269	2.63E-07	8.66E-07	5.8420
ENSG00000147099	HDAC8	1.1984	2.63E-07	8.67E-07	5.8409
ENSG00000129422	MTUS1	1.4507	2.64E-07	8.71E-07	5.8363
ENSG00000184923	NUTM2A	1.1225	2.66E-07	8.77E-07	5.8289
ENSG00000196449	YRDC	1.1885	2.70E-07	8.87E-07	5.8177
ENSG00000152253	SPC25	1.4182	2.70E-07	8.89E-07	5.8156
ENSG00000176293	ZNF135	1.1218	2.70E-07	8.89E-07	5.8155
ENSG00000178233	TMEM151B	1.1531	2.73E-07	8.98E-07	5.8057
ENSG00000049249	TNFRSF9	1.0652	2.75E-07	9.05E-07	5.7979

Gene ID	Gene Symbol	FC	P.Value	adj.P.Val	B
ENSG00000240045	STRIT1	1.0622	2.76E-07	9.06E-07	5.7962
ENSG00000115380	EFEMP1	0.4473	2.76E-07	9.07E-07	5.7951
ENSG00000213793	ZNF888	1.3612	2.76E-07	9.08E-07	5.7946
ENSG00000116285	ERRFI1	1.3400	2.77E-07	9.12E-07	5.7901
ENSG00000082438	COBLL1	1.5456	2.78E-07	9.14E-07	5.7870
ENSG00000117519	CNN3	1.8446	2.78E-07	9.14E-07	5.7870
ENSG00000101751	POLI	1.2898	2.79E-07	9.16E-07	5.7856
ENSG00000140995	DEF8	1.2718	2.82E-07	9.27E-07	5.7733
ENSG00000104140	RHOV	1.0955	2.84E-07	9.34E-07	5.7658
ENSG00000164604	GPR85	1.1511	2.85E-07	9.36E-07	5.7636
ENSG00000075420	FNDC3B	1.2768	2.85E-07	9.37E-07	5.7628
ENSG00000148700	ADD3	1.3849	2.86E-07	9.39E-07	5.7606
ENSG00000167664	TMIGD2	1.0982	2.86E-07	9.39E-07	5.7603
ENSG00000153485	TMEM251	0.7376	2.88E-07	9.45E-07	5.7536
ENSG00000078295	ADCY2	1.1653	2.88E-07	9.45E-07	5.7534
ENSG00000064225	ST3GAL6	1.5039	2.89E-07	9.47E-07	5.7514
ENSG00000108797	CNTNAP1	1.5378	2.90E-07	9.51E-07	5.7471
ENSG00000188649	CC2D2B	1.0771	2.93E-07	9.61E-07	5.7366
ENSG00000135048	CEMIP2	1.7793	2.93E-07	9.62E-07	5.7362
ENSG00000169100	SLC25A6	0.7736	2.95E-07	9.67E-07	5.7311
ENSG00000132002	DNAJB1	1.3510	2.96E-07	9.71E-07	5.7265
ENSG00000151322	NPAS3	1.3002	2.99E-07	9.81E-07	5.7165
ENSG00000108506	INTS2	1.2575	3.00E-07	9.82E-07	5.7157
ENSG00000164880	INTS1	1.2447	3.00E-07	9.82E-07	5.7152
ENSG00000013364	MVP	1.2383	3.00E-07	9.83E-07	5.7141
ENSG00000151364	KCTD14	1.4938	3.00E-07	9.84E-07	5.7128
ENSG00000155961	RAB39B	1.3932	3.02E-07	9.89E-07	5.7079
ENSG00000173653	RCE1	1.2141	3.03E-07	9.91E-07	5.7060
ENSG00000147100	SLC16A2	1.1281	3.03E-07	9.92E-07	5.7043
ENSG00000172264	MACROD2	1.5184	3.05E-07	9.97E-07	5.6997
ENSG00000248405	PRR5-ARHGAP8	1.2497	3.07E-07	1.00E-06	5.6924
ENSG00000126391	FRMD8	1.2045	3.07E-07	1.01E-06	5.6907
ENSG00000102409	BEX4	1.8042	3.09E-07	1.01E-06	5.6850
ENSG00000172775	PSME3IP1	1.2390	3.09E-07	1.01E-06	5.6845
ENSG00000140319	SRP14	1.1954	3.10E-07	1.01E-06	5.6839
ENSG00000172057	ORMDL3	1.2386	3.10E-07	1.01E-06	5.6838
ENSG00000100219	XBP1	0.7202	3.10E-07	1.01E-06	5.6827
ENSG00000130699	TAF4	1.2536	3.11E-07	1.02E-06	5.6796
ENSG00000197024	ZNF398	1.2106	3.12E-07	1.02E-06	5.6757
ENSG00000204052	LRRC73	1.0629	3.13E-07	1.02E-06	5.6724
ENSG00000198223	CSF2RA	1.2599	3.15E-07	1.03E-06	5.6678
ENSG00000264364	DYNLL2	1.2442	3.17E-07	1.03E-06	5.6618
ENSG00000132256	TRIM5	0.7251	3.18E-07	1.04E-06	5.6586
ENSG00000087206	UIMC1	1.1447	3.18E-07	1.04E-06	5.6576
ENSG00000143498	TAF1A	1.3177	3.18E-07	1.04E-06	5.6570
ENSG00000256537	SMIM10L1	1.2384	3.19E-07	1.04E-06	5.6559
ENSG00000105072	C19orf44	1.2101	3.19E-07	1.04E-06	5.6552
ENSG00000125363	AMELX	1.0281	3.20E-07	1.04E-06	5.6526
ENSG00000116918	TSNAX	1.2789	3.20E-07	1.04E-06	5.6521
ENSG00000077498	TYR	1.0265	3.23E-07	1.05E-06	5.6421
ENSG00000128159	TUBGCP6	1.2645	3.23E-07	1.05E-06	5.6420
ENSG00000112855	HARS2	1.2009	3.24E-07	1.06E-06	5.6409
ENSG00000130956	HABP4	1.3862	3.24E-07	1.06E-06	5.6389
ENSG00000128512	DOCK4	1.5047	3.27E-07	1.07E-06	5.6314

Gene ID	Gene Symbol	FC	P.Value	adj.P.Val	B
ENSG00000198648	STK39	1.4915	3.27E-07	1.07E-06	5.6315
ENSG00000105251	SHD	1.0962	3.27E-07	1.07E-06	5.6303
ENSG00000147894	C9orf72	1.2380	3.27E-07	1.07E-06	5.6302
ENSG00000163322	ABRAXAS1	1.2174	3.28E-07	1.07E-06	5.6293
ENSG00000069188	SDK2	1.2672	3.28E-07	1.07E-06	5.6287
ENSG00000053702	NRIP2	1.2255	3.28E-07	1.07E-06	5.6269
ENSG00000099326	MZF1	0.7365	3.30E-07	1.07E-06	5.6220
ENSG00000128849	CGNL1	1.1249	3.32E-07	1.08E-06	5.6160
ENSG00000146414	SHPRH	1.2446	3.33E-07	1.09E-06	5.6122
ENSG00000151881	TMEM267	0.8051	3.35E-07	1.09E-06	5.6081
ENSG00000101439	CST3	2.2360	3.36E-07	1.09E-06	5.6048
ENSG00000166446	CDYL2	1.2151	3.36E-07	1.09E-06	5.6041
ENSG00000187048	CYP4A11	1.0715	3.37E-07	1.10E-06	5.6017
ENSG00000130119	GNL3L	1.2802	3.38E-07	1.10E-06	5.5982
ENSG00000115414	FN1	1.3770	3.39E-07	1.10E-06	5.5967
ENSG00000140006	WDR89	0.7303	3.41E-07	1.11E-06	5.5896
ENSG00000137338	PGBD1	1.2390	3.42E-07	1.11E-06	5.5878
ENSG00000214160	ALG3	0.7460	3.45E-07	1.12E-06	5.5805
ENSG00000181092	ADIPOQ	1.0463	3.46E-07	1.12E-06	5.5774
ENSG00000177225	GATD1	0.7044	3.46E-07	1.12E-06	5.5760
ENSG00000164877	MICALL2	1.3600	3.47E-07	1.13E-06	5.5739
ENSG00000205730	ITPRIPL2	1.2821	3.49E-07	1.13E-06	5.5689
ENSG00000183160	TMEM119	1.3710	3.50E-07	1.14E-06	5.5663
ENSG00000242802	AP5Z1	1.2063	3.50E-07	1.14E-06	5.5654
ENSG00000198125	MB	1.1360	3.51E-07	1.14E-06	5.5634
ENSG00000040531	CTNS	1.2938	3.52E-07	1.14E-06	5.5611
ENSG00000070785	EIF2B3	0.7442	3.52E-07	1.14E-06	5.5611
ENSG00000176402	GJC3	1.1807	3.52E-07	1.14E-06	5.5594
ENSG00000129235	TXNDC17	0.7696	3.53E-07	1.14E-06	5.5579
ENSG00000120093	HOXB3	0.6960	3.53E-07	1.14E-06	5.5569
ENSG00000258947	TUBB3	1.3362	3.53E-07	1.14E-06	5.5568
ENSG00000136152	COG3	1.2658	3.58E-07	1.16E-06	5.5448
ENSG00000125378	BMP4	0.5368	3.59E-07	1.16E-06	5.5414
ENSG00000153832	FBXO36	1.1426	3.59E-07	1.16E-06	5.5400
ENSG00000134986	NREP	1.3866	3.63E-07	1.18E-06	5.5299
ENSG00000121716	PILRB	0.7024	3.63E-07	1.18E-06	5.5296
ENSG00000163428	LRRC58	1.2415	3.64E-07	1.18E-06	5.5285
ENSG00000092445	TYRO3	1.2031	3.64E-07	1.18E-06	5.5274
ENSG00000133805	AMPD3	1.5447	3.65E-07	1.18E-06	5.5250
ENSG00000142959	BEST4	1.1370	3.65E-07	1.18E-06	5.5245
ENSG00000120675	DNAJC15	0.7759	3.66E-07	1.19E-06	5.5212
ENSG00000147889	CDKN2A	1.4398	3.67E-07	1.19E-06	5.5204
ENSG00000079150	FKBP7	0.7642	3.71E-07	1.20E-06	5.5085
ENSG00000092200	RPGRIP1	1.2522	3.73E-07	1.21E-06	5.5044
ENSG00000163870	TPRA1	0.8139	3.74E-07	1.21E-06	5.5020
ENSG00000244405	ETV5	1.4566	3.75E-07	1.21E-06	5.4982
ENSG00000221986	MYBPHL	1.1898	3.75E-07	1.21E-06	5.4979
ENSG00000099617	EFNA2	1.0549	3.76E-07	1.22E-06	5.4952
ENSG00000123159	GIPC1	0.7157	3.77E-07	1.22E-06	5.4930
ENSG00000156239	N6AMT1	0.7513	3.77E-07	1.22E-06	5.4931
ENSG00000129158	SERGEF	0.8158	3.78E-07	1.22E-06	5.4917
ENSG00000198576	ARC	1.3117	3.79E-07	1.22E-06	5.4887
ENSG00000129514	FOXA1	1.3998	3.80E-07	1.23E-06	5.4868
ENSG00000107263	RAPGEF1	1.4563	3.81E-07	1.23E-06	5.4829

Gene ID	Gene Symbol	FC	P.Value	adj.P.Val	B
ENSG00000182544	MFSD5	1.1927	3.81E-07	1.23E-06	5.4828
ENSG00000162928	PEX13	1.1939	3.82E-07	1.23E-06	5.4818
ENSG00000178567	EPM2AIP1	1.2078	3.88E-07	1.25E-06	5.4658
ENSG00000156194	PPEF2	1.2426	3.89E-07	1.25E-06	5.4638
ENSG00000180758	GPR157	1.2852	3.89E-07	1.26E-06	5.4631
ENSG00000185000	DGAT1	1.2162	3.89E-07	1.26E-06	5.4626
ENSG00000154721	JAM2	1.2908	3.90E-07	1.26E-06	5.4600
ENSG00000118407	FILIP1	1.0780	3.91E-07	1.26E-06	5.4589
ENSG00000213988	ZNF90	1.2530	3.92E-07	1.26E-06	5.4558
ENSG00000179520	SLC17A8	0.7901	3.92E-07	1.27E-06	5.4548
ENSG00000103489	XYLT1	1.6528	3.94E-07	1.27E-06	5.4499
ENSG00000123500	COL10A1	1.0916	3.95E-07	1.27E-06	5.4494
ENSG00000177479	ARIH2	1.1735	3.96E-07	1.27E-06	5.4470
ENSG00000151917	BEND6	1.2057	3.96E-07	1.28E-06	5.4464
ENSG00000204381	LAYN	1.0860	3.96E-07	1.28E-06	5.4461
ENSG00000266714	MYO15B	1.9464	3.98E-07	1.28E-06	5.4419
ENSG00000117400	MPL	1.0390	3.98E-07	1.28E-06	5.4407
ENSG00000111237	VPS29	1.1755	3.98E-07	1.28E-06	5.4405
ENSG00000088386	SLC15A1	1.0739	4.00E-07	1.29E-06	5.4376
ENSG00000197782	ZNF780A	0.8259	4.05E-07	1.30E-06	5.4253
ENSG00000139289	PHLDA1	2.0068	4.06E-07	1.31E-06	5.4226
ENSG00000176182	MYPOP	0.7997	4.07E-07	1.31E-06	5.4198
ENSG00000171489	SPACA5	1.1222	4.09E-07	1.32E-06	5.4145
ENSG00000183914	DNAH2	1.1099	4.11E-07	1.32E-06	5.4098
ENSG00000070761	CFAP20	1.3821	4.12E-07	1.32E-06	5.4089
ENSG00000144895	EIF2A	0.8493	4.16E-07	1.34E-06	5.3989
ENSG00000149488	TMC2	1.0455	4.18E-07	1.34E-06	5.3938
ENSG00000068796	KIF2A	1.2464	4.20E-07	1.35E-06	5.3893
ENSG00000106415	GLCCI1	0.6780	4.23E-07	1.36E-06	5.3825
ENSG00000124508	BTN2A2	1.4319	4.23E-07	1.36E-06	5.3824
ENSG00000144648	ACKR2	1.1108	4.25E-07	1.36E-06	5.3791
ENSG00000172578	KLHL6	0.7203	4.25E-07	1.36E-06	5.3783
ENSG00000106290	TAF6	1.2589	4.25E-07	1.37E-06	5.3770
ENSG00000112599	GUCA1B	1.2231	4.27E-07	1.37E-06	5.3727
ENSG00000170145	SIK2	1.2261	4.27E-07	1.37E-06	5.3726
ENSG00000100852	ARHGAP5	1.2747	4.28E-07	1.37E-06	5.3719
ENSG00000181444	ZNF467	1.5069	4.32E-07	1.38E-06	5.3633
ENSG00000227471	AKR1B15	1.1964	4.33E-07	1.39E-06	5.3610
ENSG00000181016	LSMEM1	1.3107	4.36E-07	1.40E-06	5.3540
ENSG00000162980	ARL5A	1.2022	4.36E-07	1.40E-06	5.3537
ENSG00000135469	COQ10A	1.2860	4.36E-07	1.40E-06	5.3526
ENSG00000127804	METTL16	1.2055	4.38E-07	1.40E-06	5.3485
ENSG00000119943	PYROXD2	1.4344	4.39E-07	1.41E-06	5.3466
ENSG00000146950	SHROOM2	1.0455	4.39E-07	1.41E-06	5.3466
ENSG00000102805	CLN5	0.8148	4.40E-07	1.41E-06	5.3439
ENSG00000148337	CIZ1	1.1758	4.44E-07	1.42E-06	5.3357
ENSG00000204176	SYT15	1.1744	4.44E-07	1.42E-06	5.3356
ENSG00000139508	SLC46A3	0.6574	4.44E-07	1.42E-06	5.3350
ENSG00000171097	KYAT1	0.7571	4.51E-07	1.44E-06	5.3206
ENSG00000213398	LCAT	1.4078	4.52E-07	1.45E-06	5.3186
ENSG00000163629	PTPN13	1.4140	4.53E-07	1.45E-06	5.3169
ENSG00000177599	ZNF491	0.7973	4.54E-07	1.45E-06	5.3139
ENSG00000158220	ESYT3	1.1300	4.56E-07	1.46E-06	5.3105
ENSG00000185339	TCN2	1.7657	4.58E-07	1.47E-06	5.3049

Gene ID	Gene Symbol	FC	P.Value	adj.P.Val	B
ENSG00000102910	LONP2	1.2584	4.59E-07	1.47E-06	5.3043
ENSG00000143502	SUSD4	1.2914	4.59E-07	1.47E-06	5.3041
ENSG00000180957	PITPNB	1.2083	4.61E-07	1.48E-06	5.2988
ENSG00000134278	SPIRE1	1.4169	4.63E-07	1.48E-06	5.2959
ENSG00000111224	PARP11	1.2744	4.63E-07	1.48E-06	5.2944
ENSG00000132694	ARHGEF11	1.3448	4.64E-07	1.48E-06	5.2942
ENSG00000196323	ZBTB44	1.2480	4.68E-07	1.49E-06	5.2858
ENSG00000103257	SLC7A5	1.5733	4.69E-07	1.50E-06	5.2838
ENSG00000050628	PTGER3	1.1272	4.70E-07	1.50E-06	5.2809
ENSG00000140263	SORD	0.6841	4.73E-07	1.51E-06	5.2742
ENSG00000172062	SMN1	1.1812	4.74E-07	1.52E-06	5.2719
ENSG00000105750	ZNF85	1.3858	4.76E-07	1.52E-06	5.2681
ENSG00000104055	TGM5	1.0513	4.78E-07	1.53E-06	5.2648
ENSG00000196465	MYL6B	1.2059	4.78E-07	1.53E-06	5.2643
ENSG00000163946	TASOR	1.2266	4.81E-07	1.53E-06	5.2588
ENSG00000198870	STKLD1	1.2413	4.82E-07	1.54E-06	5.2571
ENSG00000134287	ARF3	1.2712	4.83E-07	1.54E-06	5.2545
ENSG00000101134	DOK5	1.2402	4.86E-07	1.55E-06	5.2482
ENSG00000130383	FUT5	1.2369	4.86E-07	1.55E-06	5.2483
ENSG00000145431	PDGFC	1.4800	4.93E-07	1.57E-06	5.2341
ENSG00000091622	PITPNM3	1.1171	4.94E-07	1.57E-06	5.2334
ENSG00000159871	LYPD5	1.0999	4.96E-07	1.58E-06	5.2295
ENSG00000221946	FXYD7	1.4468	4.98E-07	1.59E-06	5.2256
ENSG00000124787	RPP40	0.6670	5.02E-07	1.60E-06	5.2183
ENSG00000110375	UPK2	1.0304	5.04E-07	1.61E-06	5.2132
ENSG00000198860	TSEN15	1.3113	5.05E-07	1.61E-06	5.2122
ENSG00000172346	CSDC2	1.1006	5.06E-07	1.61E-06	5.2099
ENSG00000160007	ARHGAP35	1.2419	5.08E-07	1.62E-06	5.2064
ENSG00000186367	MINAR2	1.0958	5.10E-07	1.62E-06	5.2031
ENSG00000164082	GRM2	1.1181	5.10E-07	1.62E-06	5.2015
ENSG00000106526	ACTR3C	1.2264	5.12E-07	1.63E-06	5.1990
ENSG00000159086	PAXBP1	1.2547	5.13E-07	1.63E-06	5.1967
ENSG00000198785	GRIN3A	0.9458	5.14E-07	1.63E-06	5.1948
ENSG00000168589	DYNLRB2	1.2292	5.14E-07	1.64E-06	5.1945
ENSG00000148200	NR6A1	1.2020	5.17E-07	1.64E-06	5.1898
ENSG00000206562	METTL6	0.7771	5.17E-07	1.64E-06	5.1892
ENSG00000113448	PDE4D	1.5691	5.17E-07	1.64E-06	5.1883
ENSG00000157985	AGAP1	1.4800	5.19E-07	1.65E-06	5.1849
ENSG00000120907	ADRA1A	1.0830	5.20E-07	1.65E-06	5.1832
ENSG00000188596	CFAP54	1.4535	5.23E-07	1.66E-06	5.1771
ENSG00000131379	C3orf20	1.0904	5.24E-07	1.66E-06	5.1766
ENSG00000164930	FZD6	1.4550	5.26E-07	1.67E-06	5.1714
ENSG00000244355	LY6G6D	1.1268	5.27E-07	1.68E-06	5.1697
ENSG00000182054	IDH2	0.7469	5.32E-07	1.69E-06	5.1609
ENSG00000152439	ZNF773	1.2242	5.33E-07	1.69E-06	5.1600
ENSG00000142178	SIK1	1.4947	5.35E-07	1.70E-06	5.1565
ENSG00000178467	P4HTM	0.7496	5.38E-07	1.71E-06	5.1509
ENSG00000116254	CHD5	1.0623	5.41E-07	1.72E-06	5.1458
ENSG00000165792	METTL17	1.2430	5.44E-07	1.73E-06	5.1395
ENSG00000172296	SPTLC3	1.3369	5.50E-07	1.75E-06	5.1285
ENSG00000131969	ABHD12B	1.1586	5.52E-07	1.75E-06	5.1260
ENSG00000142784	WDTC1	1.2487	5.54E-07	1.76E-06	5.1218
ENSG00000163872	YEATS2	1.2268	5.57E-07	1.77E-06	5.1170
ENSG00000165661	QSOX2	0.7607	5.59E-07	1.77E-06	5.1139

Gene ID	Gene Symbol	FC	P.Value	adj.P.Val	B
ENSG00000119383	PTPA	0.8021	5.62E-07	1.78E-06	5.1090
ENSG00000205111	CDKL4	1.2498	5.64E-07	1.79E-06	5.1052
ENSG00000152457	DCLRE1C	1.2461	5.64E-07	1.79E-06	5.1048
ENSG00000168280	KIF5C	1.1248	5.64E-07	1.79E-06	5.1045
ENSG00000126856	PRDM7	1.1292	5.65E-07	1.79E-06	5.1028
ENSG00000143369	ECM1	1.1287	5.66E-07	1.79E-06	5.1024
ENSG00000174775	HRAS	1.2486	5.66E-07	1.79E-06	5.1019
ENSG00000113812	ACTR8	1.1920	5.69E-07	1.80E-06	5.0963
ENSG00000149577	SIDT2	1.2336	5.72E-07	1.81E-06	5.0923
ENSG00000171786	NHLH1	1.1172	5.72E-07	1.81E-06	5.0911
ENSG00000147573	TRIM55	0.6310	5.73E-07	1.81E-06	5.0904
ENSG00000186517	ARHGAP30	1.3268	5.73E-07	1.81E-06	5.0903
ENSG00000167968	DNASE1L2	0.8258	5.76E-07	1.82E-06	5.0847
ENSG00000089048	ESF1	1.2132	5.81E-07	1.84E-06	5.0772
ENSG00000139445	FOXN4	1.0680	5.81E-07	1.84E-06	5.0766
ENSG00000185245	GP1BA	1.1426	5.82E-07	1.84E-06	5.0756
ENSG00000118217	ATF6	1.3293	5.83E-07	1.84E-06	5.0726
ENSG00000180999	C1orf105	1.0836	5.84E-07	1.85E-06	5.0718
ENSG00000100150	DEPDC5	1.2045	5.85E-07	1.85E-06	5.0700
ENSG00000179304	FAM156B	1.2837	5.85E-07	1.85E-06	5.0700
ENSG00000010803	SCMH1	1.2348	5.86E-07	1.85E-06	5.0682
ENSG00000198732	SMOC1	0.5929	5.86E-07	1.85E-06	5.0677
ENSG00000164331	ANKRA2	1.2030	5.88E-07	1.86E-06	5.0654
ENSG00000188906	LRRK2	1.6271	5.88E-07	1.86E-06	5.0651
ENSG00000149262	INTS4	1.2325	5.93E-07	1.87E-06	5.0568
ENSG00000084453	SLCO1A2	1.3636	5.94E-07	1.87E-06	5.0559
ENSG00000160445	ZER1	1.2266	5.93E-07	1.87E-06	5.0560
ENSG00000161638	ITGA5	1.3797	5.96E-07	1.88E-06	5.0516
ENSG00000117399	CDC20	1.4998	5.97E-07	1.88E-06	5.0510
ENSG00000007866	TEAD3	1.2977	6.00E-07	1.89E-06	5.0456
ENSG00000160199	PKNOX1	1.1549	6.00E-07	1.89E-06	5.0456
ENSG00000171992	SYNPO	1.3516	6.01E-07	1.90E-06	5.0432
ENSG00000204315	FKBPL	1.2188	6.08E-07	1.92E-06	5.0332
ENSG00000165821	SALL2	1.3122	6.09E-07	1.92E-06	5.0313
ENSG00000079257	LXN	0.7547	6.09E-07	1.92E-06	5.0311
ENSG00000160345	C9orf116	1.4565	6.10E-07	1.92E-06	5.0289
ENSG00000179715	PCED1B	0.6450	6.11E-07	1.92E-06	5.0282
ENSG00000109171	SLAIN2	1.2211	6.11E-07	1.93E-06	5.0277
ENSG00000007944	MYLIP	1.4917	6.11E-07	1.93E-06	5.0275
ENSG00000136161	RCBTB2	0.6411	6.12E-07	1.93E-06	5.0271
ENSG00000145555	MYO10	1.3056	6.18E-07	1.94E-06	5.0176
ENSG00000104343	UBE2W	1.1900	6.19E-07	1.95E-06	5.0158
ENSG00000151239	TWF1	1.1690	6.19E-07	1.95E-06	5.0157
ENSG00000158792	SPATA2L	1.2326	6.19E-07	1.95E-06	5.0154
ENSG00000148842	CNNM2	1.2050	6.19E-07	1.95E-06	5.0153
ENSG00000124562	SNRPC	1.1585	6.20E-07	1.95E-06	5.0145
ENSG00000138675	FGF5	1.1556	6.25E-07	1.97E-06	5.0061
ENSG00000164741	DLC1	0.6424	6.27E-07	1.97E-06	5.0025
ENSG00000160211	G6PD	1.2834	6.28E-07	1.97E-06	5.0018
ENSG00000072062	PRKACA	1.2415	6.31E-07	1.98E-06	4.9970
ENSG00000110717	NDUFS8	0.8362	6.32E-07	1.99E-06	4.9952
ENSG00000113100	CDH9	1.2768	6.34E-07	1.99E-06	4.9930
ENSG00000143156	NME7	1.2520	6.34E-07	1.99E-06	4.9921
ENSG00000161980	POLR3K	0.7709	6.34E-07	1.99E-06	4.9918

Gene ID	Gene Symbol	FC	P.Value	adj.P.Val	B
ENSG00000198642	KLHL9	0.7693	6.40E-07	2.01E-06	4.9840
ENSG00000065183	WDR3	0.7178	6.50E-07	2.04E-06	4.9677
ENSG00000128268	MGAT3	0.5985	6.51E-07	2.04E-06	4.9668
ENSG00000113643	RARS1	1.1881	6.52E-07	2.05E-06	4.9653
ENSG00000269190	FBXO17	1.2618	6.54E-07	2.05E-06	4.9622
ENSG00000163121	NEURL3	1.0904	6.55E-07	2.06E-06	4.9608
ENSG00000173083	HPSE	1.3941	6.55E-07	2.06E-06	4.9606
ENSG00000176273	SLC35G1	0.7971	6.61E-07	2.07E-06	4.9525
ENSG00000103502	CDIPT	1.1878	6.61E-07	2.07E-06	4.9522
ENSG00000154227	CERS3	0.9093	6.61E-07	2.07E-06	4.9522
ENSG00000113716	HMGXB3	1.1829	6.61E-07	2.07E-06	4.9517
ENSG00000164406	LEAP2	0.7680	6.63E-07	2.08E-06	4.9494
ENSG00000163900	TMEM41A	1.2182	6.68E-07	2.09E-06	4.9425
ENSG00000177483	RBM44	1.1795	6.68E-07	2.09E-06	4.9426
ENSG00000188050	RNF133	1.4057	6.69E-07	2.10E-06	4.9404
ENSG00000117118	SDHB	1.1528	6.69E-07	2.10E-06	4.9400
ENSG00000124155	PIGT	1.2600	6.71E-07	2.10E-06	4.9372
ENSG00000197208	SLC22A4	1.2906	6.73E-07	2.11E-06	4.9342
ENSG00000203896	LIME1	1.4463	6.77E-07	2.12E-06	4.9293
ENSG00000064490	RFXANK	0.8553	6.80E-07	2.13E-06	4.9249
ENSG00000122711	SPINK4	1.1876	6.81E-07	2.13E-06	4.9231
ENSG00000136478	TEX2	0.7743	6.82E-07	2.13E-06	4.9224
ENSG00000142609	CFAP74	1.1688	6.90E-07	2.16E-06	4.9114
ENSG00000139343	SNRPF	1.1727	6.96E-07	2.18E-06	4.9030
ENSG00000111218	PRMT8	1.0111	6.97E-07	2.18E-06	4.9005
ENSG00000122787	AKR1D1	0.6809	7.00E-07	2.19E-06	4.8969
ENSG00000086475	SEPHS1	1.2461	7.02E-07	2.19E-06	4.8946
ENSG00000114209	PDCD10	1.1963	7.02E-07	2.20E-06	4.8940
ENSG00000104529	EEF1D	0.8352	7.05E-07	2.20E-06	4.8898
ENSG00000164241	C5orf63	0.7317	7.06E-07	2.21E-06	4.8882
ENSG00000151327	FAM177A1	1.1567	7.07E-07	2.21E-06	4.8869
ENSG00000047648	ARHGAP6	1.4903	7.10E-07	2.22E-06	4.8831
ENSG00000162300	ZFPL1	1.1920	7.10E-07	2.22E-06	4.8832
ENSG00000042781	USH2A	0.7334	7.11E-07	2.22E-06	4.8815
ENSG00000197444	OGDHL	1.3931	7.11E-07	2.22E-06	4.8812
ENSG00000101417	PXMP4	0.7769	7.12E-07	2.22E-06	4.8805
ENSG00000119777	TMEM214	1.2341	7.13E-07	2.23E-06	4.8796
ENSG00000218891	ZNF579	1.2488	7.13E-07	2.23E-06	4.8789
ENSG00000154639	CXADR	1.3650	7.15E-07	2.23E-06	4.8763
ENSG00000131149	GSE1	1.2847	7.15E-07	2.23E-06	4.8761
ENSG00000162889	MAPKAPK2	1.3682	7.20E-07	2.25E-06	4.8691
ENSG00000177688	SUMO4	1.0935	7.22E-07	2.25E-06	4.8672
ENSG00000011454	RABGAP1	1.2040	7.22E-07	2.25E-06	4.8669
ENSG00000233056	ERVH48-1	1.0727	7.25E-07	2.26E-06	4.8629
ENSG00000134852	CLOCK	1.2523	7.26E-07	2.26E-06	4.8619
ENSG00000073861	TBX21	0.6697	7.26E-07	2.26E-06	4.8614
ENSG00000167880	EVPL	1.1369	7.34E-07	2.29E-06	4.8510
ENSG00000171103	TRMT61B	1.2089	7.38E-07	2.30E-06	4.8458
ENSG00000160226	CFAP410	1.2339	7.40E-07	2.31E-06	4.8435
ENSG00000039068	CDH1	1.4719	7.40E-07	2.31E-06	4.8431
ENSG00000127423	AUNIP	1.1599	7.40E-07	2.31E-06	4.8432
ENSG00000169813	HNRNPF	1.2173	7.44E-07	2.32E-06	4.8384
ENSG00000135116	HRK	1.3863	7.44E-07	2.32E-06	4.8382
ENSG00000141665	FBXO15	1.2276	7.47E-07	2.32E-06	4.8347

Gene ID	Gene Symbol	FC	P.Value	adj.P.Val	B
ENSG00000095303	PTGS1	0.7066	7.47E-07	2.33E-06	4.8340
ENSG00000137218	FRS3	1.2022	7.52E-07	2.34E-06	4.8282
ENSG00000118514	ALDH8A1	1.2229	7.58E-07	2.36E-06	4.8197
ENSG00000233436	BTBD18	1.0389	7.60E-07	2.36E-06	4.8178
ENSG00000119906	SLF2	1.2971	7.65E-07	2.38E-06	4.8114
ENSG00000237649	KIFC1	1.4930	7.67E-07	2.39E-06	4.8082
ENSG00000131697	NPHP4	1.2387	7.69E-07	2.39E-06	4.8063
ENSG00000204160	ZDHHC18	1.3030	7.70E-07	2.39E-06	4.8055
ENSG00000161634	DCD	1.0797	7.72E-07	2.40E-06	4.8021
ENSG00000105767	CADM4	1.4679	7.84E-07	2.44E-06	4.7882
ENSG00000172432	GTPBP2	1.2370	7.85E-07	2.44E-06	4.7862
ENSG00000187474	FPR3	0.7615	7.87E-07	2.45E-06	4.7839
ENSG00000110171	TRIM3	1.3075	7.88E-07	2.45E-06	4.7833
ENSG00000177425	PAWR	1.3952	7.87E-07	2.45E-06	4.7834
ENSG00000187240	DYNC2H1	1.2175	7.93E-07	2.46E-06	4.7770
ENSG00000109047	RCVRN	1.1024	7.95E-07	2.47E-06	4.7739
ENSG00000079156	OSBPL6	1.2286	8.03E-07	2.49E-06	4.7642
ENSG00000213160	KLHL23	1.2343	8.06E-07	2.50E-06	4.7614
ENSG00000138382	METTL5	0.7822	8.07E-07	2.51E-06	4.7597
ENSG00000154310	TNIK	1.2538	8.09E-07	2.51E-06	4.7572
ENSG00000258643	BCL2L2-PABPN1	1.3152	8.12E-07	2.52E-06	4.7542
ENSG00000176049	JAKMIP2	1.3014	8.17E-07	2.53E-06	4.7481
ENSG00000152990	ADGRA3	1.5207	8.17E-07	2.54E-06	4.7474
ENSG00000132680	KHDC4	1.2985	8.18E-07	2.54E-06	4.7463
ENSG00000126561	STAT5A	1.6608	8.19E-07	2.54E-06	4.7455
ENSG00000160209	PDXK	1.2252	8.19E-07	2.54E-06	4.7455
ENSG00000129083	COPB1	1.1624	8.21E-07	2.55E-06	4.7431
ENSG00000167969	ECI1	0.8207	8.22E-07	2.55E-06	4.7426
ENSG00000068654	POLR1A	1.2794	8.25E-07	2.56E-06	4.7384
ENSG00000164932	CTHRC1	2.2539	8.28E-07	2.56E-06	4.7355
ENSG00000152700	SAR1B	0.8025	8.34E-07	2.58E-06	4.7281
ENSG00000185958	FAM186A	1.0660	8.35E-07	2.59E-06	4.7270
ENSG00000120802	TMPO	1.3863	8.35E-07	2.59E-06	4.7267
ENSG00000145888	GLRA1	1.0277	8.38E-07	2.60E-06	4.7232
ENSG00000118276	B4GALT6	1.1619	8.39E-07	2.60E-06	4.7223
ENSG00000167112	TRUB2	0.8002	8.39E-07	2.60E-06	4.7224
ENSG00000159720	ATP6V0D1	1.2655	8.44E-07	2.61E-06	4.7161
ENSG00000128606	LRRC17	0.8557	8.45E-07	2.62E-06	4.7152
ENSG00000205670	SMIM11A	0.7976	8.47E-07	2.62E-06	4.7136
ENSG00000154174	TOMM70	1.1803	8.48E-07	2.62E-06	4.7125
ENSG00000117090	SLAMF1	0.4761	8.49E-07	2.63E-06	4.7106
ENSG00000149308	NPAT	1.2208	8.50E-07	2.63E-06	4.7102
ENSG00000186814	ZSCAN30	1.2812	8.50E-07	2.63E-06	4.7098
ENSG00000213593	TMX2	0.8302	8.52E-07	2.63E-06	4.7080
ENSG00000178257	PRM3	1.1812	8.52E-07	2.63E-06	4.7072
ENSG00000135119	RNFT2	1.2894	8.53E-07	2.64E-06	4.7059
ENSG00000104687	GSR	1.2402	8.56E-07	2.64E-06	4.7035
ENSG00000166387	PPFIBP2	1.3436	8.56E-07	2.64E-06	4.7028
ENSG00000111846	GCNT2	1.2389	8.58E-07	2.65E-06	4.7011
ENSG00000166016	ABTB2	1.2189	8.62E-07	2.66E-06	4.6962
ENSG00000196345	ZKSCAN7	0.7633	8.67E-07	2.68E-06	4.6913
ENSG00000121281	ADCY7	1.3080	8.73E-07	2.69E-06	4.6843
ENSG00000186409	CCDC30	1.2142	8.73E-07	2.69E-06	4.6843
ENSG00000124493	GRM4	1.4225	8.73E-07	2.69E-06	4.6839

Gene ID	Gene Symbol	FC	P.Value	adj.P.Val	B
ENSG00000134222	PSRC1	1.2881	8.74E-07	2.70E-06	4.6827
ENSG00000143771	CNIH4	1.2448	8.75E-07	2.70E-06	4.6819
ENSG00000100276	RASL10A	1.1870	8.81E-07	2.72E-06	4.6757
ENSG00000152147	GEMIN6	0.7513	8.86E-07	2.73E-06	4.6702
ENSG00000128829	EIF2AK4	1.2692	8.87E-07	2.73E-06	4.6689
ENSG00000179104	TMTC2	1.2504	8.96E-07	2.76E-06	4.6593
ENSG00000172362	OR5B12	0.9469	9.05E-07	2.79E-06	4.6497
ENSG00000162994	CLHC1	1.2669	9.12E-07	2.81E-06	4.6417
ENSG00000110048	OSBP	1.1824	9.14E-07	2.82E-06	4.6398
ENSG00000107201	DDX58	0.7571	9.16E-07	2.82E-06	4.6378
ENSG00000090776	EFNB1	1.1922	9.19E-07	2.83E-06	4.6350
ENSG00000086967	MYBPC2	1.7133	9.19E-07	2.83E-06	4.6347
ENSG00000117899	MESD	0.8132	9.22E-07	2.84E-06	4.6320
ENSG00000167851	CD300A	0.5654	9.24E-07	2.85E-06	4.6295
ENSG00000124762	CDKN1A	1.4611	9.28E-07	2.86E-06	4.6256
ENSG00000162692	VCAM1	0.5179	9.30E-07	2.86E-06	4.6233
ENSG00000236699	ARHGEF38	1.2656	9.30E-07	2.86E-06	4.6234
ENSG00000163931	TKT	0.7979	9.34E-07	2.87E-06	4.6190
ENSG00000125430	HS3ST3B1	1.2411	9.34E-07	2.87E-06	4.6187
ENSG00000060491	OGFR	1.2129	9.35E-07	2.88E-06	4.6179
ENSG00000140479	PCSK6	1.2481	9.38E-07	2.89E-06	4.6147
ENSG00000124391	IL17C	1.1489	9.42E-07	2.90E-06	4.6108
ENSG00000171603	CLSTN1	1.2532	9.43E-07	2.90E-06	4.6103
ENSG00000183354	KIAA2026	1.2118	9.48E-07	2.91E-06	4.6050
ENSG00000155970	MICU3	1.3868	9.66E-07	2.97E-06	4.5871
ENSG00000100284	TOM1	1.2593	9.74E-07	2.99E-06	4.5791
ENSG00000196141	SPATS2L	1.4063	9.77E-07	3.00E-06	4.5756
ENSG00000198515	CNGA1	1.0974	9.91E-07	3.04E-06	4.5621
ENSG00000198874	TYW1	1.1838	9.93E-07	3.05E-06	4.5599
ENSG00000213983	AP1G2	1.2915	1.00E-06	3.08E-06	4.5519
ENSG00000229972	IQCF3	1.2195	1.00E-06	3.08E-06	4.5492
ENSG00000164684	ZNF704	1.2725	1.01E-06	3.09E-06	4.5483
ENSG00000130449	ZSWIM6	1.2730	1.01E-06	3.10E-06	4.5449
ENSG00000178585	CTNNBIP1	1.1913	1.01E-06	3.11E-06	4.5417
ENSG00000198837	DENND4B	1.2872	1.02E-06	3.12E-06	4.5387
ENSG00000186318	BACE1	0.7559	1.02E-06	3.12E-06	4.5381
ENSG00000156738	MS4A1	2.4665	1.02E-06	3.14E-06	4.5307
ENSG00000078328	RBFOX1	1.0863	1.03E-06	3.16E-06	4.5256
ENSG00000156052	GNAQ	1.3999	1.03E-06	3.17E-06	4.5229
ENSG00000164305	CASP3	1.2803	1.04E-06	3.18E-06	4.5173
ENSG00000257446	ZNF878	1.0950	1.04E-06	3.18E-06	4.5174
ENSG00000151702	FLI1	0.7645	1.05E-06	3.21E-06	4.5093
ENSG00000178125	PPP1R42	1.0914	1.05E-06	3.23E-06	4.5041
ENSG00000129911	KLF16	1.2540	1.05E-06	3.23E-06	4.5028
ENSG00000146013	GFRA3	1.0326	1.06E-06	3.26E-06	4.4936
ENSG00000121211	MND1	1.3671	1.08E-06	3.30E-06	4.4809
ENSG00000154928	EPHB1	0.6373	1.09E-06	3.32E-06	4.4747
ENSG00000181924	COA4	0.8032	1.09E-06	3.33E-06	4.4732
ENSG00000176020	AMIGO3	1.5221	1.09E-06	3.34E-06	4.4708
ENSG00000170122	FOXD4	0.8281	1.09E-06	3.34E-06	4.4686
ENSG00000143199	ADCY10	1.1115	1.10E-06	3.36E-06	4.4648
ENSG00000053900	ANAPC4	1.2326	1.10E-06	3.38E-06	4.4577
ENSG00000162722	TRIM58	1.3223	1.11E-06	3.38E-06	4.4564
ENSG00000136682	CBWD2	1.2125	1.11E-06	3.39E-06	4.4535

Gene ID	Gene Symbol	FC	P.Value	adj.P.Val	B
ENSG00000134716	CYP2J2	0.6178	1.11E-06	3.40E-06	4.4503
ENSG00000102109	PCSK1N	1.9758	1.11E-06	3.41E-06	4.4490
ENSG00000188766	SPRED3	1.2050	1.12E-06	3.41E-06	4.4474
ENSG00000106615	RHEB	1.1901	1.12E-06	3.43E-06	4.4436
ENSG00000117054	ACADM	0.7198	1.13E-06	3.44E-06	4.4392
ENSG00000125266	EFNB2	1.1313	1.13E-06	3.45E-06	4.4372
ENSG00000138073	PREB	0.7096	1.13E-06	3.45E-06	4.4368
ENSG00000115183	TANC1	1.4944	1.13E-06	3.45E-06	4.4358
ENSG00000105472	CLEC11A	1.4982	1.13E-06	3.45E-06	4.4353
ENSG00000100911	PSME2	0.7817	1.14E-06	3.48E-06	4.4277
ENSG00000072274	TFRC	1.2710	1.14E-06	3.49E-06	4.4258
ENSG00000138443	ABI2	1.2790	1.15E-06	3.52E-06	4.4175
ENSG00000162415	ZSWIM5	1.2178	1.15E-06	3.52E-06	4.4171
ENSG00000198353	HOXC4	0.7106	1.15E-06	3.52E-06	4.4159
ENSG00000150471	ADGRL3	1.3448	1.16E-06	3.54E-06	4.4104
ENSG00000074276	CDHR2	1.1726	1.17E-06	3.55E-06	4.4063
ENSG00000057019	DCBLD2	1.2727	1.17E-06	3.57E-06	4.4033
ENSG00000120329	SLC25A2	1.0752	1.17E-06	3.58E-06	4.3992
ENSG00000168671	UGT3A2	0.5597	1.18E-06	3.58E-06	4.3978
ENSG00000168079	SCARA5	1.1273	1.18E-06	3.59E-06	4.3969
ENSG00000072135	PTPN18	1.6004	1.18E-06	3.59E-06	4.3958
ENSG00000124541	RRP36	1.1662	1.20E-06	3.67E-06	4.3748
ENSG00000141034	GID4	1.1749	1.20E-06	3.67E-06	4.3748
ENSG00000029639	TFB1M	0.7741	1.21E-06	3.68E-06	4.3718
ENSG00000124313	IQSEC2	1.3696	1.21E-06	3.68E-06	4.3704
ENSG00000174460	ZCCHC12	1.0769	1.22E-06	3.70E-06	4.3652
ENSG00000084623	EIF3I	1.1311	1.22E-06	3.71E-06	4.3628
ENSG00000095932	SMIM24	1.2811	1.22E-06	3.71E-06	4.3628
ENSG00000135775	COG2	1.2449	1.22E-06	3.72E-06	4.3608
ENSG00000186393	KRT26	1.0322	1.22E-06	3.73E-06	4.3586
ENSG00000183647	ZNF530	1.2555	1.23E-06	3.73E-06	4.3569
ENSG00000169727	GPS1	1.2004	1.24E-06	3.77E-06	4.3460
ENSG00000172640	OR10AD1	1.1083	1.24E-06	3.78E-06	4.3458
ENSG00000127511	SIN3B	1.2333	1.26E-06	3.82E-06	4.3336
ENSG00000073605	GSDMB	1.3864	1.27E-06	3.85E-06	4.3263
ENSG00000140264	SERF2	0.8729	1.27E-06	3.85E-06	4.3260
ENSG00000165795	NDRG2	1.5203	1.27E-06	3.85E-06	4.3258
ENSG00000103657	HERC1	1.2478	1.27E-06	3.85E-06	4.3249
ENSG00000157017	GHRL	1.3163	1.27E-06	3.85E-06	4.3247
ENSG00000164742	ADCY1	1.2888	1.27E-06	3.86E-06	4.3244
ENSG00000185860	CCDC190	1.0400	1.27E-06	3.86E-06	4.3243
ENSG00000179388	EGR3	0.6294	1.28E-06	3.89E-06	4.3165
ENSG00000146828	SLC12A9	0.8006	1.29E-06	3.91E-06	4.3117
ENSG00000171953	ATPAF2	1.1793	1.29E-06	3.91E-06	4.3115
ENSG00000172183	ISG20	0.7450	1.29E-06	3.93E-06	4.3061
ENSG00000126698	DNAJC8	1.1737	1.30E-06	3.93E-06	4.3045
ENSG00000115561	CHMP3	0.8659	1.30E-06	3.95E-06	4.3000
ENSG00000141401	IMPA2	1.3543	1.30E-06	3.95E-06	4.2989
ENSG00000100105	PATZ1	1.2647	1.30E-06	3.96E-06	4.2980
ENSG00000167721	TSR1	1.2175	1.31E-06	3.99E-06	4.2902
ENSG00000241685	ARPC1A	0.7914	1.32E-06	3.99E-06	4.2895
ENSG00000088756	ARHGAP28	1.1551	1.32E-06	4.01E-06	4.2849
ENSG00000119004	CYP20A1	1.2725	1.32E-06	4.01E-06	4.2848
ENSG00000267855	NDUFA7	0.8444	1.34E-06	4.07E-06	4.2708

Gene ID	Gene Symbol	FC	P.Value	adj.P.Val	B
ENSG00000178568	ERBB4	1.3207	1.34E-06	4.07E-06	4.2698
ENSG00000130726	TRIM28	1.2022	1.35E-06	4.10E-06	4.2631
ENSG00000241563	CORT	1.1041	1.35E-06	4.10E-06	4.2621
ENSG00000142611	PRDM16	1.1587	1.36E-06	4.11E-06	4.2595
ENSG00000181830	SLC35C1	0.7546	1.36E-06	4.13E-06	4.2553
ENSG00000105877	DNAH11	1.2477	1.37E-06	4.14E-06	4.2528
ENSG00000132359	RAP1GAP2	1.5631	1.37E-06	4.15E-06	4.2512
ENSG00000132840	BHMT2	1.0706	1.38E-06	4.17E-06	4.2458
ENSG00000144959	NCEH1	0.7895	1.38E-06	4.18E-06	4.2428
ENSG00000137776	SLTM	1.1683	1.39E-06	4.22E-06	4.2349
ENSG00000095059	DHPS	0.8298	1.40E-06	4.23E-06	4.2323
ENSG00000085231	AK6	0.8548	1.40E-06	4.23E-06	4.2320
ENSG00000063176	SPHK2	0.7618	1.40E-06	4.23E-06	4.2304
ENSG00000261787	TCF24	1.0726	1.40E-06	4.24E-06	4.2297
ENSG00000130511	SSBP4	0.6874	1.41E-06	4.26E-06	4.2251
ENSG00000125534	PPDPF	1.3871	1.42E-06	4.29E-06	4.2169
ENSG00000134030	CTIF	1.2670	1.44E-06	4.36E-06	4.2014
ENSG00000154814	OXNAD1	0.7884	1.44E-06	4.36E-06	4.2010
ENSG00000138604	GLCE	0.7784	1.45E-06	4.39E-06	4.1939
ENSG00000143126	CELSR2	1.2758	1.46E-06	4.40E-06	4.1912
ENSG00000104369	JPH1	1.1138	1.46E-06	4.41E-06	4.1902
ENSG00000143479	DYRK3	1.2609	1.46E-06	4.42E-06	4.1869
ENSG00000116815	CD58	1.3414	1.47E-06	4.43E-06	4.1859
ENSG00000123975	CKS2	0.6854	1.47E-06	4.44E-06	4.1820
ENSG00000133111	RFXAP	0.7621	1.47E-06	4.44E-06	4.1819
ENSG00000126903	SLC10A3	1.2548	1.47E-06	4.45E-06	4.1795
ENSG00000168803	ADAL	0.7721	1.48E-06	4.46E-06	4.1786
ENSG00000133019	CHRM3	1.3353	1.48E-06	4.46E-06	4.1772
ENSG00000184939	ZFP90	1.2676	1.48E-06	4.46E-06	4.1772
ENSG00000157869	RAB28	1.2169	1.49E-06	4.50E-06	4.1695
ENSG00000137573	SULF1	1.1800	1.50E-06	4.52E-06	4.1646
ENSG00000158552	ZFAND2B	1.1986	1.50E-06	4.52E-06	4.1637
ENSG00000104131	EIF3J	0.8083	1.50E-06	4.53E-06	4.1618
ENSG00000128482	RNF112	1.0684	1.51E-06	4.55E-06	4.1567
ENSG00000185513	L3MBTL1	1.3462	1.51E-06	4.57E-06	4.1540
ENSG00000178531	CTXN1	1.1079	1.52E-06	4.58E-06	4.1516
ENSG00000197134	ZNF257	1.1879	1.53E-06	4.61E-06	4.1444
ENSG00000101997	CCDC22	1.2249	1.53E-06	4.61E-06	4.1441
ENSG00000187151	ANGPTL5	1.0707	1.53E-06	4.62E-06	4.1426
ENSG00000008226	DLEC1	1.4528	1.53E-06	4.62E-06	4.1422
ENSG00000108591	DRG2	1.1817	1.54E-06	4.63E-06	4.1394
ENSG00000147027	TMEM47	1.4829	1.54E-06	4.65E-06	4.1361
ENSG00000188394	GPR21	1.0420	1.55E-06	4.65E-06	4.1345
ENSG00000266967	AARSD1	1.1786	1.55E-06	4.67E-06	4.1309
ENSG00000136770	DNAJC1	0.8097	1.57E-06	4.72E-06	4.1202
ENSG00000105819	PMPCB	1.1487	1.57E-06	4.73E-06	4.1190
ENSG00000080561	MID2	1.4171	1.57E-06	4.73E-06	4.1177
ENSG00000139644	TMBIM6	1.3293	1.58E-06	4.74E-06	4.1154
ENSG00000198756	COLGALT2	1.2015	1.58E-06	4.75E-06	4.1132
ENSG00000185808	PIGP	0.8121	1.58E-06	4.76E-06	4.1111
ENSG00000106089	STX1A	1.3368	1.59E-06	4.77E-06	4.1097
ENSG00000184785	SMIM10	1.4254	1.59E-06	4.78E-06	4.1073
ENSG00000204569	PPP1R10	1.2579	1.60E-06	4.81E-06	4.1009
ENSG00000143847	PPFIA4	1.1404	1.60E-06	4.82E-06	4.0997

Gene ID	Gene Symbol	FC	P.Value	adj.P.Val	B
ENSG00000067221	STOML1	0.7715	1.60E-06	4.82E-06	4.0991
ENSG00000124785	NRN1	0.5729	1.61E-06	4.85E-06	4.0935
ENSG00000183020	AP2A2	1.1872	1.61E-06	4.85E-06	4.0935
ENSG00000130414	NDUFA10	1.1393	1.61E-06	4.85E-06	4.0924
ENSG00000162409	PRKAA2	1.0559	1.62E-06	4.85E-06	4.0921
ENSG00000143119	CD53	0.7718	1.62E-06	4.86E-06	4.0902
ENSG00000143494	VASH2	1.5199	1.62E-06	4.86E-06	4.0898
ENSG00000258839	MC1R	0.7174	1.62E-06	4.86E-06	4.0893
ENSG00000113163	CERT1	1.1819	1.63E-06	4.90E-06	4.0829
ENSG00000168255	POLR2J3	1.2782	1.63E-06	4.90E-06	4.0827
ENSG00000165406	MARCHF8	1.2188	1.63E-06	4.90E-06	4.0819
ENSG00000185917	SETD4	1.2129	1.64E-06	4.92E-06	4.0767
ENSG00000105662	CRTC1	0.7699	1.64E-06	4.93E-06	4.0760
ENSG00000112149	CD83	1.6490	1.64E-06	4.93E-06	4.0755
ENSG00000116819	TFAP2E	1.1727	1.65E-06	4.94E-06	4.0738
ENSG00000155974	GRIP1	0.7531	1.65E-06	4.94E-06	4.0723
ENSG00000095464	PDE6C	1.1789	1.65E-06	4.95E-06	4.0710
ENSG00000162552	WNT4	0.6182	1.66E-06	4.96E-06	4.0682
ENSG00000101150	TPD52L2	1.1844	1.67E-06	4.99E-06	4.0624
ENSG00000167618	LAIR2	1.2854	1.67E-06	5.00E-06	4.0608
ENSG00000172009	THOP1	1.2045	1.68E-06	5.02E-06	4.0572
ENSG00000169957	ZNF768	1.1991	1.68E-06	5.02E-06	4.0569
ENSG00000165948	IFI27L1	1.3755	1.68E-06	5.02E-06	4.0564
ENSG00000132604	TERF2	1.2019	1.68E-06	5.04E-06	4.0529
ENSG00000171840	NINJ2	0.7610	1.69E-06	5.06E-06	4.0496
ENSG00000079263	SP140	0.7606	1.69E-06	5.07E-06	4.0460
ENSG00000158901	WFDC8	1.0259	1.70E-06	5.10E-06	4.0416
ENSG00000133315	MACROD1	0.7740	1.71E-06	5.12E-06	4.0366
ENSG00000130520	LSM4	0.8457	1.71E-06	5.13E-06	4.0354
ENSG00000138892	TTLL8	1.0333	1.72E-06	5.13E-06	4.0342
ENSG00000103507	BCKDK	1.2017	1.72E-06	5.14E-06	4.0334
ENSG00000174915	PTDSS2	0.8354	1.73E-06	5.16E-06	4.0287
ENSG00000087258	GNAO1	1.0991	1.74E-06	5.20E-06	4.0216
ENSG00000188629	ZNF177	0.7429	1.74E-06	5.20E-06	4.0210
ENSG00000146555	SDK1	1.2482	1.74E-06	5.21E-06	4.0182
ENSG00000169621	APLF	1.2087	1.75E-06	5.23E-06	4.0156
ENSG00000116641	DOCK7	1.1820	1.75E-06	5.23E-06	4.0145
ENSG00000138829	FBN2	1.1706	1.75E-06	5.24E-06	4.0131
ENSG00000145912	NHP2	0.8191	1.76E-06	5.24E-06	4.0123
ENSG00000180914	OXTR	1.1192	1.76E-06	5.25E-06	4.0117
ENSG00000014914	MTMR11	1.4114	1.77E-06	5.27E-06	4.0064
ENSG00000153779	TGIF2LX	1.5581	1.77E-06	5.29E-06	4.0038
ENSG00000254806	SYS1-DBNDD2	1.2494	1.78E-06	5.30E-06	4.0010
ENSG00000188976	NOC2L	1.1991	1.78E-06	5.31E-06	3.9992
ENSG00000166197	NOLC1	1.2327	1.78E-06	5.31E-06	3.9989
ENSG00000100865	CINP	1.1701	1.78E-06	5.32E-06	3.9976
ENSG00000003056	M6PR	1.1900	1.79E-06	5.33E-06	3.9947
ENSG00000148175	STOM	0.7050	1.79E-06	5.33E-06	3.9947
ENSG00000121104	FAM117A	1.3105	1.79E-06	5.34E-06	3.9930
ENSG00000133134	BEX2	1.5212	1.79E-06	5.35E-06	3.9915
ENSG00000163535	SGO2	1.2857	1.79E-06	5.35E-06	3.9915
ENSG00000213402	PTPRCAP	2.3055	1.80E-06	5.36E-06	3.9893
ENSG00000196562	SULF2	0.4710	1.80E-06	5.37E-06	3.9876
ENSG00000121005	CRISPLD1	1.1945	1.80E-06	5.37E-06	3.9869

Gene ID	Gene Symbol	FC	P.Value	adj.P.Val	B
ENSG00000165609	NUDT5	0.8175	1.81E-06	5.39E-06	3.9837
ENSG00000166794	PPIB	0.8413	1.81E-06	5.39E-06	3.9838
ENSG00000122417	ODF2L	1.3355	1.81E-06	5.40E-06	3.9813
ENSG00000197594	ENPP1	1.2535	1.82E-06	5.41E-06	3.9787
ENSG00000142156	COL6A1	1.4724	1.82E-06	5.41E-06	3.9784
ENSG00000186288	PABPC1L2A	1.0360	1.83E-06	5.44E-06	3.9730
ENSG00000167555	ZNF528	1.4052	1.83E-06	5.46E-06	3.9705
ENSG00000087448	KLHL42	1.3301	1.84E-06	5.47E-06	3.9690
ENSG00000105329	TGFB1	1.3115	1.84E-06	5.47E-06	3.9688
ENSG00000070759	TESK2	1.2408	1.84E-06	5.48E-06	3.9667
ENSG00000120696	KBTBD7	0.8325	1.84E-06	5.48E-06	3.9665
ENSG00000099337	KCNK6	0.7655	1.84E-06	5.48E-06	3.9659
ENSG00000186222	BLOC1S4	1.2133	1.84E-06	5.48E-06	3.9660
ENSG00000142512	SIGLEC10	1.2535	1.84E-06	5.48E-06	3.9658
ENSG00000143319	ISG20L2	1.2404	1.85E-06	5.49E-06	3.9642
ENSG00000272968	RBAK-RBAKDN	1.1603	1.85E-06	5.50E-06	3.9620
ENSG00000112159	MDN1	1.2278	1.85E-06	5.51E-06	3.9602
ENSG00000151812	SLC35F4	1.1649	1.88E-06	5.59E-06	3.9466
ENSG00000167549	CORO6	1.3151	1.88E-06	5.59E-06	3.9458
ENSG00000164520	RAET1E	1.0450	1.89E-06	5.60E-06	3.9430
ENSG00000142669	SH3BGRL3	1.2408	1.89E-06	5.61E-06	3.9418
ENSG00000101323	HAO1	1.0159	1.89E-06	5.61E-06	3.9410
ENSG00000178057	NDUFAF3	0.8057	1.89E-06	5.61E-06	3.9409
ENSG00000178896	EXOSC4	0.7777	1.90E-06	5.64E-06	3.9370
ENSG00000160055	TMEM234	1.2450	1.91E-06	5.66E-06	3.9332
ENSG00000144677	CTDSPL	1.2766	1.91E-06	5.66E-06	3.9321
ENSG00000161281	COX7A1	1.1509	1.91E-06	5.67E-06	3.9312
ENSG00000141668	CBLN2	1.3175	1.91E-06	5.67E-06	3.9298
ENSG00000035720	STAP1	0.6185	1.91E-06	5.68E-06	3.9296
ENSG00000177595	PIDD1	1.2341	1.91E-06	5.68E-06	3.9288
ENSG00000111087	GLI1	1.2275	1.92E-06	5.71E-06	3.9240
ENSG00000222011	FAM185A	1.2193	1.93E-06	5.72E-06	3.9221
ENSG00000162755	KLHDC9	1.3928	1.93E-06	5.72E-06	3.9208
ENSG00000197956	S100A6	1.8388	1.94E-06	5.76E-06	3.9145
ENSG00000176619	LMNB2	1.2540	1.95E-06	5.77E-06	3.9130
ENSG00000173110	HSPA6	1.5886	1.95E-06	5.79E-06	3.9089
ENSG00000142546	NOSIP	0.8376	1.96E-06	5.81E-06	3.9057
ENSG00000104951	IL4I1	1.2438	1.96E-06	5.82E-06	3.9039
ENSG00000123552	USP45	1.2020	1.97E-06	5.82E-06	3.9037
ENSG00000105696	TMEM59L	1.1630	1.99E-06	5.88E-06	3.8936
ENSG00000105388	CEACAM5	1.0439	2.00E-06	5.91E-06	3.8884
ENSG00000006047	YBX2	1.1231	2.01E-06	5.95E-06	3.8815
ENSG00000131747	TOP2A	1.5166	2.01E-06	5.96E-06	3.8799
ENSG00000125651	GTF2F1	1.1674	2.02E-06	5.97E-06	3.8787
ENSG00000119139	TJP2	1.1914	2.02E-06	5.98E-06	3.8765
ENSG00000188886	ASTL	1.2068	2.02E-06	5.98E-06	3.8762
ENSG00000151650	VENTX	1.0679	2.03E-06	6.02E-06	3.8709
ENSG00000164253	WDR41	0.8255	2.03E-06	6.02E-06	3.8703
ENSG00000156172	CFAP418	1.1127	2.04E-06	6.04E-06	3.8664
ENSG00000132612	VPS4A	1.1851	2.04E-06	6.04E-06	3.8660
ENSG00000145996	CDKAL1	1.1695	2.05E-06	6.05E-06	3.8649
ENSG00000149591	TAGLN	1.2418	2.05E-06	6.06E-06	3.8635
ENSG00000137942	FNBP1L	1.1945	2.05E-06	6.06E-06	3.8624
ENSG00000215912	TTC34	1.1740	2.06E-06	6.08E-06	3.8601

Gene ID	Gene Symbol	FC	P.Value	adj.P.Val	B
ENSG00000041880	PARP3	0.7781	2.06E-06	6.09E-06	3.8571
ENSG00000143256	PFDN2	1.2083	2.07E-06	6.12E-06	3.8531
ENSG00000087085	ACHE	1.4031	2.07E-06	6.13E-06	3.8520
ENSG00000221978	CCNL2	1.2627	2.08E-06	6.13E-06	3.8506
ENSG00000171867	PRNP	1.3111	2.08E-06	6.14E-06	3.8499
ENSG00000261308	FIGNL2	1.1075	2.08E-06	6.14E-06	3.8500
ENSG00000154153	RETREG1	1.2337	2.10E-06	6.19E-06	3.8412
ENSG00000165502	RPL36AL	0.8232	2.12E-06	6.26E-06	3.8310
ENSG00000253313	C1orf210	1.0913	2.13E-06	6.28E-06	3.8279
ENSG00000197299	BLM	0.7230	2.13E-06	6.28E-06	3.8276
ENSG00000152763	DNAI4	1.2132	2.13E-06	6.30E-06	3.8245
ENSG00000189067	LITAF	1.2931	2.13E-06	6.30E-06	3.8241
ENSG00000165617	DACT1	1.1791	2.14E-06	6.32E-06	3.8199
ENSG00000056291	NPFFR2	1.0233	2.15E-06	6.33E-06	3.8191
ENSG00000120868	APAF1	1.2557	2.16E-06	6.37E-06	3.8133
ENSG00000142303	ADAMTS10	1.3240	2.17E-06	6.39E-06	3.8096
ENSG00000102098	SCML2	1.2006	2.17E-06	6.41E-06	3.8065
ENSG00000163738	MTHFD2L	1.1959	2.18E-06	6.43E-06	3.8037
ENSG00000115486	GGCX	1.1763	2.19E-06	6.46E-06	3.7992
ENSG00000047634	SCML1	1.4429	2.19E-06	6.47E-06	3.7975
ENSG00000095794	CREM	1.1986	2.22E-06	6.54E-06	3.7868
ENSG00000157388	CACNA1D	1.1522	2.24E-06	6.60E-06	3.7781
ENSG00000007520	TSR3	1.1852	2.25E-06	6.63E-06	3.7729
ENSG00000143418	CERS2	1.2123	2.25E-06	6.64E-06	3.7718
ENSG00000257591	ZNF625	0.8025	2.26E-06	6.64E-06	3.7708
ENSG00000093144	ECHDC1	0.7882	2.26E-06	6.64E-06	3.7706
ENSG00000116299	ELAPOR1	1.1220	2.26E-06	6.65E-06	3.7701
ENSG00000091844	RGS17	1.1008	2.26E-06	6.65E-06	3.7692
ENSG00000006282	SPATA20	1.2504	2.29E-06	6.74E-06	3.7557
ENSG00000082781	ITGB5	1.4900	2.31E-06	6.79E-06	3.7491
ENSG00000073417	PDE8A	1.2810	2.31E-06	6.80E-06	3.7481
ENSG00000188993	LRRC66	1.0906	2.31E-06	6.80E-06	3.7479
ENSG00000146707	POMZP3	1.2474	2.31E-06	6.80E-06	3.7475
ENSG00000183090	FREM3	1.1495	2.32E-06	6.81E-06	3.7459
ENSG00000087253	LPCAT2	1.4802	2.32E-06	6.81E-06	3.7456
ENSG00000171864	PRND	1.0383	2.33E-06	6.84E-06	3.7416
ENSG00000108839	ALOX12	1.1630	2.33E-06	6.84E-06	3.7414
ENSG00000135916	ITM2C	0.6565	2.33E-06	6.86E-06	3.7381
ENSG00000163251	FZD5	1.1472	2.34E-06	6.88E-06	3.7342
ENSG00000221826	PSG3	1.0491	2.34E-06	6.88E-06	3.7343
ENSG00000082805	ERC1	1.2882	2.35E-06	6.90E-06	3.7320
ENSG00000118495	PLAGL1	1.4625	2.35E-06	6.90E-06	3.7316
ENSG00000163046	ANKRD30BL	1.2757	2.35E-06	6.91E-06	3.7300
ENSG00000187678	SPRY4	1.2282	2.36E-06	6.91E-06	3.7298
ENSG00000113119	TMCO6	1.2436	2.36E-06	6.92E-06	3.7279
ENSG00000163541	SUCLG1	1.1307	2.36E-06	6.93E-06	3.7272
ENSG00000118785	SPP1	2.0006	2.36E-06	6.94E-06	3.7259
ENSG00000222046	DCDC2B	1.0960	2.38E-06	6.97E-06	3.7215
ENSG00000079805	DNM2	1.1743	2.38E-06	6.98E-06	3.7203
ENSG00000058668	ATP2B4	1.4310	2.38E-06	6.98E-06	3.7193
ENSG00000240445	FOXO3B	1.1153	2.38E-06	6.99E-06	3.7184
ENSG00000101052	IFT52	1.1930	2.39E-06	6.99E-06	3.7176
ENSG00000136514	RTP4	0.6864	2.39E-06	7.01E-06	3.7153
ENSG00000108622	ICAM2	0.7832	2.41E-06	7.06E-06	3.7086

Gene ID	Gene Symbol	FC	P.Value	adj.P.Val	B
ENSG00000071794	HLTF	0.6930	2.41E-06	7.06E-06	3.7072
ENSG00000166164	BRD7	1.2081	2.41E-06	7.07E-06	3.7068
ENSG00000147234	FRMPD3	1.4748	2.41E-06	7.07E-06	3.7066
ENSG00000154997	SEPTIN14	1.1289	2.41E-06	7.07E-06	3.7059
ENSG00000123143	PKN1	1.1608	2.42E-06	7.08E-06	3.7049
ENSG00000152128	TMEM163	1.3029	2.43E-06	7.12E-06	3.6993
ENSG00000059378	PARP12	1.2940	2.43E-06	7.12E-06	3.6990
ENSG00000179165	PXT1	1.0760	2.44E-06	7.13E-06	3.6971
ENSG00000196890	H2BU1	1.5276	2.44E-06	7.13E-06	3.6969
ENSG00000059804	SLC2A3	1.6170	2.44E-06	7.14E-06	3.6956
ENSG00000108523	RNF167	1.1707	2.45E-06	7.16E-06	3.6935
ENSG00000108342	CSF3	1.1512	2.47E-06	7.24E-06	3.6824
ENSG00000184887	BTBD6	1.2810	2.49E-06	7.27E-06	3.6776
ENSG00000067082	KLF6	1.5858	2.49E-06	7.29E-06	3.6746
ENSG00000174255	ZNF80	1.1233	2.50E-06	7.32E-06	3.6711
ENSG00000204619	PPP1R11	0.7824	2.51E-06	7.33E-06	3.6697
ENSG00000162836	ACP6	1.3257	2.52E-06	7.37E-06	3.6645
ENSG00000188833	ENTPD8	1.0587	2.54E-06	7.41E-06	3.6583
ENSG00000163659	TIPARP	1.3504	2.55E-06	7.46E-06	3.6527
ENSG00000166135	HIF1AN	1.2266	2.56E-06	7.47E-06	3.6506
ENSG00000146416	AIG1	0.7887	2.57E-06	7.50E-06	3.6469
ENSG00000171497	PPID	1.2147	2.57E-06	7.52E-06	3.6445
ENSG00000186298	PPP1CC	1.1518	2.60E-06	7.59E-06	3.6346
ENSG00000134853	PDGFRA	1.1961	2.61E-06	7.63E-06	3.6304
ENSG00000153339	TRAPPC8	1.1958	2.62E-06	7.65E-06	3.6270
ENSG00000072849	DERL2	1.1935	2.62E-06	7.66E-06	3.6262
ENSG00000100410	PHF5A	1.1540	2.62E-06	7.66E-06	3.6262
ENSG00000196757	ZNF700	1.2228	2.63E-06	7.67E-06	3.6243
ENSG00000086730	LAT2	1.5747	2.64E-06	7.70E-06	3.6209
ENSG00000176834	VSIG10	1.1980	2.64E-06	7.70E-06	3.6208
ENSG00000136104	RNASEH2B	0.7827	2.64E-06	7.70E-06	3.6195
ENSG00000116661	FBXO2	0.7552	2.67E-06	7.78E-06	3.6095
ENSG00000189184	PCDH18	1.2198	2.67E-06	7.78E-06	3.6094
ENSG00000148943	LIN7C	1.2055	2.68E-06	7.80E-06	3.6069
ENSG00000140836	ZFHX3	1.0901	2.68E-06	7.81E-06	3.6060
ENSG00000135604	STX11	1.4768	2.69E-06	7.82E-06	3.6039
ENSG00000011405	PIK3C2A	1.2448	2.69E-06	7.83E-06	3.6036
ENSG00000142511	GPR32	1.0225	2.71E-06	7.89E-06	3.5959
ENSG00000028116	VRK2	1.1997	2.71E-06	7.90E-06	3.5941
ENSG00000166503	HDGFL3	1.2673	2.72E-06	7.92E-06	3.5916
ENSG00000196739	COL27A1	1.1303	2.72E-06	7.93E-06	3.5909
ENSG00000166268	MYRFL	0.8268	2.74E-06	7.98E-06	3.5842
ENSG00000187595	ZNF385C	1.1469	2.75E-06	8.00E-06	3.5818
ENSG00000223601	EBLN1	0.7023	2.75E-06	8.00E-06	3.5812
ENSG00000131899	LLGL1	1.2046	2.75E-06	8.00E-06	3.5810
ENSG00000100526	CDKN3	1.4008	2.76E-06	8.02E-06	3.5793
ENSG00000148344	PTGES	1.1747	2.77E-06	8.07E-06	3.5730
ENSG00000102172	SMS	1.2146	2.79E-06	8.10E-06	3.5687
ENSG00000183273	CCDC60	1.0886	2.80E-06	8.14E-06	3.5640
ENSG00000100650	SRSF5	1.2561	2.80E-06	8.15E-06	3.5633
ENSG00000138623	SEMA7A	1.8458	2.80E-06	8.15E-06	3.5626
ENSG00000205352	PRR13	1.1977	2.80E-06	8.15E-06	3.5622
ENSG00000107874	CUEDC2	1.1883	2.81E-06	8.16E-06	3.5615
ENSG00000110066	KMT5B	1.1989	2.81E-06	8.17E-06	3.5594

Gene ID	Gene Symbol	FC	P.Value	adj.P.Val	B
ENSG00000165805	C12orf50	1.3212	2.81E-06	8.18E-06	3.5587
ENSG00000157873	TNFRSF14	0.8223	2.82E-06	8.19E-06	3.5573
ENSG00000267673	FDX2	0.7953	2.82E-06	8.19E-06	3.5567
ENSG00000163794	UCN	1.2303	2.83E-06	8.21E-06	3.5545
ENSG00000204852	TCTN1	1.3687	2.83E-06	8.21E-06	3.5544
ENSG00000156218	ADAMTSL3	1.2847	2.84E-06	8.24E-06	3.5502
ENSG00000182196	ARL6IP4	1.1288	2.85E-06	8.27E-06	3.5472
ENSG00000150527	MIA2	1.1900	2.87E-06	8.32E-06	3.5416
ENSG00000001084	GCLC	1.2048	2.88E-06	8.37E-06	3.5357
ENSG00000135702	CHST5	1.0598	2.89E-06	8.38E-06	3.5343
ENSG00000065268	WDR18	0.8150	2.94E-06	8.53E-06	3.5169
ENSG00000157014	TATDN2	1.1773	2.95E-06	8.57E-06	3.5124
ENSG00000095203	EPB41L4B	1.1881	2.96E-06	8.60E-06	3.5089
ENSG00000142794	NBPF3	1.2667	2.97E-06	8.60E-06	3.5084
ENSG00000115825	PRKD3	1.4696	2.97E-06	8.60E-06	3.5082
ENSG00000006659	LGALS14	1.4264	2.97E-06	8.61E-06	3.5070
ENSG00000117597	UTP25	1.2368	2.99E-06	8.67E-06	3.5002
ENSG00000149743	TRPT1	0.8391	2.99E-06	8.67E-06	3.5003
ENSG00000196616	ADH1B	1.0621	3.00E-06	8.68E-06	3.4986
ENSG00000257529	RPL36A-HNRNPH2	0.8038	3.02E-06	8.75E-06	3.4914
ENSG00000221923	ZNF880	1.4469	3.03E-06	8.77E-06	3.4882
ENSG00000187118	CMC1	0.8014	3.05E-06	8.83E-06	3.4821
ENSG00000113578	FGF1	1.0706	3.06E-06	8.87E-06	3.4775
ENSG00000172716	SLFN11	0.6669	3.07E-06	8.89E-06	3.4757
ENSG00000167772	ANGPTL4	1.2564	3.07E-06	8.89E-06	3.4755
ENSG00000170421	KRT8	1.4344	3.08E-06	8.91E-06	3.4724
ENSG00000172613	RAD9A	1.2495	3.08E-06	8.92E-06	3.4712
ENSG00000139618	BRCA2	1.2575	3.09E-06	8.93E-06	3.4699
ENSG00000041515	MYO16	1.1137	3.13E-06	9.04E-06	3.4583
ENSG00000169189	NSMCE1	1.3064	3.13E-06	9.05E-06	3.4571
ENSG00000101442	ACTR5	1.2161	3.13E-06	9.06E-06	3.4561
ENSG00000144935	TRPC1	1.2355	3.14E-06	9.09E-06	3.4525
ENSG00000184702	SEPTIN5	1.4387	3.15E-06	9.10E-06	3.4517
ENSG00000162771	FAM71A	1.3357	3.16E-06	9.14E-06	3.4466
ENSG00000162517	PEF1	1.1664	3.17E-06	9.15E-06	3.4461
ENSG00000186866	POFUT2	1.2615	3.17E-06	9.17E-06	3.4437
ENSG00000107929	LARP4B	1.2212	3.18E-06	9.19E-06	3.4412
ENSG00000120471	TP53AIP1	1.0723	3.22E-06	9.31E-06	3.4293
ENSG00000141579	ZNF750	1.0283	3.23E-06	9.32E-06	3.4274
ENSG00000142657	PGD	1.2023	3.25E-06	9.37E-06	3.4221
ENSG00000136160	EDNRB	0.3765	3.25E-06	9.39E-06	3.4206
ENSG00000162896	PIGR	1.0721	3.27E-06	9.43E-06	3.4157
ENSG00000185046	ANKS1B	1.1139	3.30E-06	9.52E-06	3.4063
ENSG00000138316	ADAMTS14	1.1459	3.30E-06	9.53E-06	3.4056
ENSG00000174136	RGMB	1.2360	3.31E-06	9.55E-06	3.4033
ENSG00000100373	UPK3A	1.1986	3.32E-06	9.58E-06	3.4005
ENSG00000087266	SH3BP2	1.3272	3.33E-06	9.62E-06	3.3962
ENSG00000153066	TXNDC11	1.2591	3.35E-06	9.65E-06	3.3928
ENSG00000169184	MN1	0.7830	3.35E-06	9.67E-06	3.3905
ENSG00000221995	TIAF1	1.2597	3.35E-06	9.67E-06	3.3903
ENSG00000167996	FTH1	1.2960	3.37E-06	9.72E-06	3.3854
ENSG00000106609	TMEM248	1.1774	3.38E-06	9.74E-06	3.3835
ENSG00000021355	SERPINB1	1.2815	3.38E-06	9.75E-06	3.3825
ENSG00000100577	GSTZ1	0.7743	3.39E-06	9.76E-06	3.3807

Gene ID	Gene Symbol	FC	P.Value	adj.P.Val	B
ENSG00000138078	PREPL	0.7302	3.39E-06	9.78E-06	3.3796
ENSG00000151779	NBAS	1.1656	3.42E-06	9.84E-06	3.3727
ENSG00000171124	FUT3	1.4038	3.42E-06	9.84E-06	3.3725
ENSG00000178761	FAM219B	0.8264	3.43E-06	9.87E-06	3.3697
ENSG00000165424	ZCCHC24	1.2536	3.44E-06	9.91E-06	3.3657
ENSG00000115875	SRSF7	1.3274	3.45E-06	9.93E-06	3.3641
ENSG00000173674	EIF1AX	1.1775	3.46E-06	9.95E-06	3.3618
ENSG00000125246	CLYBL	0.7683	3.47E-06	9.99E-06	3.3573
ENSG00000156920	ADGRG4	1.0567	3.48E-06	1.00E-05	3.3557
ENSG00000122873	CISD1	0.7553	3.49E-06	1.00E-05	3.3527
ENSG00000018189	RUFY3	1.3968	3.50E-06	1.01E-05	3.3502
ENSG00000111252	SH2B3	1.2959	3.52E-06	1.01E-05	3.3448
ENSG00000164796	CSMD3	1.1642	3.55E-06	1.02E-05	3.3372
ENSG00000197948	FCHSD1	1.3334	3.59E-06	1.03E-05	3.3259
ENSG00000136167	LCP1	2.0079	3.61E-06	1.04E-05	3.3200
ENSG00000066629	EML1	1.3083	3.62E-06	1.04E-05	3.3185
ENSG00000186806	VSIG10L	1.1812	3.64E-06	1.05E-05	3.3109
ENSG00000135407	AVIL	1.2685	3.67E-06	1.05E-05	3.3044
ENSG00000131233	GJA9	1.0734	3.67E-06	1.06E-05	3.3032
ENSG00000165383	LRRC18	1.0277	3.68E-06	1.06E-05	3.3023
ENSG00000139971	ARMH4	1.2123	3.68E-06	1.06E-05	3.3013
ENSG00000091140	DLD	1.1922	3.70E-06	1.06E-05	3.2967
ENSG00000124217	MOCS3	1.2093	3.71E-06	1.06E-05	3.2949
ENSG00000164089	ETNPPL	1.0641	3.71E-06	1.06E-05	3.2941
ENSG00000173914	RBM4B	0.7647	3.71E-06	1.07E-05	3.2936
ENSG00000165637	VDAC2	1.1260	3.71E-06	1.07E-05	3.2934
ENSG00000155229	MMS19	1.1928	3.73E-06	1.07E-05	3.2891
ENSG00000174989	FBXW8	1.3644	3.73E-06	1.07E-05	3.2888
ENSG00000198908	BHLHB9	1.1733	3.74E-06	1.07E-05	3.2849
ENSG00000188266	HYKK	0.7207	3.75E-06	1.07E-05	3.2845
ENSG00000184445	KNTC1	1.3781	3.76E-06	1.08E-05	3.2820
ENSG00000149716	LTO1	1.2046	3.79E-06	1.09E-05	3.2731
ENSG00000136379	ABHD17C	0.7528	3.82E-06	1.10E-05	3.2656
ENSG00000101842	VSIG1	1.2505	3.84E-06	1.10E-05	3.2616
ENSG00000186416	NKRF	1.2090	3.85E-06	1.10E-05	3.2576
ENSG00000120694	HSPH1	1.3465	3.86E-06	1.11E-05	3.2548
ENSG00000140332	TLE3	1.2817	3.88E-06	1.11E-05	3.2516
ENSG00000166532	RIMKLB	1.4065	3.91E-06	1.12E-05	3.2437
ENSG00000101911	PRPS2	1.3573	3.94E-06	1.13E-05	3.2370
ENSG00000143434	SEMA6C	1.1910	3.94E-06	1.13E-05	3.2366
ENSG00000167395	ZNF646	1.2375	3.95E-06	1.13E-05	3.2335
ENSG00000104921	FCER2	2.0114	3.96E-06	1.13E-05	3.2307
ENSG00000185272	RBM11	1.5316	3.97E-06	1.14E-05	3.2300
ENSG00000124098	FAM210B	1.2772	3.98E-06	1.14E-05	3.2264
ENSG00000167702	KIFC2	1.2851	4.00E-06	1.15E-05	3.2210
ENSG00000162614	NEXN	1.0884	4.04E-06	1.16E-05	3.2123
ENSG00000168310	IRF2	0.7590	4.04E-06	1.16E-05	3.2123
ENSG00000205220	PSMB10	0.7872	4.04E-06	1.16E-05	3.2112
ENSG00000171425	ZNF581	0.7732	4.05E-06	1.16E-05	3.2094
ENSG00000153815	CMIP	1.2544	4.06E-06	1.16E-05	3.2072
ENSG00000136295	TTYH3	1.4191	4.08E-06	1.17E-05	3.2016
ENSG00000151612	ZNF827	1.2560	4.11E-06	1.18E-05	3.1955
ENSG00000271092	TLCD4-RWDD3	0.8467	4.11E-06	1.18E-05	3.1955
ENSG00000132676	DAP3	1.1913	4.13E-06	1.18E-05	3.1919

Gene ID	Gene Symbol	FC	P.Value	adj.P.Val	B
ENSG00000111731	C2CD5	1.2415	4.14E-06	1.18E-05	3.1891
ENSG00000099365	STX1B	1.1555	4.14E-06	1.18E-05	3.1878
ENSG00000101298	SNPH	1.2182	4.14E-06	1.18E-05	3.1878
ENSG00000006377	DLX6	1.0945	4.15E-06	1.18E-05	3.1875
ENSG00000105618	PRPF31	1.1466	4.16E-06	1.19E-05	3.1848
ENSG00000188215	DCUN1D3	1.1477	4.18E-06	1.19E-05	3.1796
ENSG00000179862	CITED4	1.4670	4.19E-06	1.20E-05	3.1772
ENSG00000123983	ACSL3	1.2003	4.21E-06	1.20E-05	3.1722
ENSG00000172469	MANEA	0.7052	4.28E-06	1.22E-05	3.1575
ENSG00000156096	UGT2B4	1.0557	4.28E-06	1.22E-05	3.1570
ENSG00000178904	DPY19L3	1.2201	4.28E-06	1.22E-05	3.1568
ENSG00000137875	BCL2L10	1.2318	4.33E-06	1.24E-05	3.1448
ENSG00000114013	CD86	1.6409	4.34E-06	1.24E-05	3.1441
ENSG00000111783	RFX4	1.0884	4.34E-06	1.24E-05	3.1429
ENSG00000197816	CCDC180	0.7234	4.34E-06	1.24E-05	3.1427
ENSG00000138674	SEC31A	1.2138	4.35E-06	1.24E-05	3.1420
ENSG00000130717	UCK1	1.1650	4.36E-06	1.24E-05	3.1392
ENSG00000159055	MIS18A	1.2607	4.37E-06	1.24E-05	3.1373
ENSG00000175087	PDIK1L	1.2484	4.37E-06	1.24E-05	3.1374
ENSG00000105677	TMEM147	0.8140	4.39E-06	1.25E-05	3.1322
ENSG00000119636	BBOF1	1.1705	4.44E-06	1.26E-05	3.1220
ENSG00000149451	ADAM33	1.0701	4.44E-06	1.26E-05	3.1216
ENSG00000139197	PEX5	1.2041	4.45E-06	1.27E-05	3.1198
ENSG00000115129	TP53I3	1.2769	4.46E-06	1.27E-05	3.1182
ENSG00000136643	RPS6KC1	1.3090	4.49E-06	1.28E-05	3.1104
ENSG00000138639	ARHGAP24	1.2731	4.49E-06	1.28E-05	3.1101
ENSG00000117143	UAP1	1.2892	4.50E-06	1.28E-05	3.1086
ENSG00000082293	COL19A1	1.2101	4.51E-06	1.28E-05	3.1066
ENSG00000104549	SQLE	1.4170	4.52E-06	1.29E-05	3.1048
ENSG00000204909	SPINK9	1.2584	4.54E-06	1.29E-05	3.1009
ENSG00000167874	TMEM88	1.2207	4.54E-06	1.29E-05	3.0997
ENSG00000170312	CDK1	1.5156	4.54E-06	1.29E-05	3.0997
ENSG00000100678	SLC8A3	0.7614	4.56E-06	1.30E-05	3.0970
ENSG00000160188	RSPH1	1.3052	4.56E-06	1.30E-05	3.0963
ENSG00000186854	TRABD2A	0.6147	4.56E-06	1.30E-05	3.0953
ENSG00000178694	NSUN3	1.2111	4.57E-06	1.30E-05	3.0941
ENSG00000250423	KIAA1210	1.0121	4.58E-06	1.30E-05	3.0922
ENSG00000204136	GGTA1	0.6586	4.59E-06	1.31E-05	3.0889
ENSG00000138131	LOXL4	1.4049	4.61E-06	1.31E-05	3.0859
ENSG00000140694	PARN	1.1974	4.61E-06	1.31E-05	3.0859
ENSG00000137274	BPHL	1.2385	4.62E-06	1.31E-05	3.0833
ENSG00000134369	NAV1	1.2690	4.64E-06	1.32E-05	3.0798
ENSG00000065150	IPO5	1.1929	4.64E-06	1.32E-05	3.0792
ENSG00000080618	CPB2	1.0302	4.65E-06	1.32E-05	3.0773
ENSG00000166037	CEP57	1.2157	4.65E-06	1.32E-05	3.0768
ENSG00000152292	SH2D6	1.1693	4.66E-06	1.32E-05	3.0748
ENSG00000185306	C12orf56	1.1509	4.70E-06	1.33E-05	3.0669
ENSG00000142330	CAPN10	1.1919	4.70E-06	1.33E-05	3.0663
ENSG00000167723	TRPV3	1.6085	4.71E-06	1.34E-05	3.0659
ENSG00000114646	CSPG5	1.1221	4.71E-06	1.34E-05	3.0648
ENSG00000138796	HADH	1.2850	4.71E-06	1.34E-05	3.0647
ENSG00000163156	SCNM1	1.2151	4.71E-06	1.34E-05	3.0648
ENSG00000166145	SPINT1	0.6599	4.72E-06	1.34E-05	3.0634
ENSG00000125304	TM9SF2	0.8227	4.72E-06	1.34E-05	3.0625

Gene ID	Gene Symbol	FC	P.Value	adj.P.Val	B
ENSG00000168703	WFDC12	1.0933	4.74E-06	1.34E-05	3.0595
ENSG00000182308	DCAF4L1	1.1585	4.75E-06	1.35E-05	3.0576
ENSG00000154655	L3MBTL4	1.3648	4.78E-06	1.35E-05	3.0515
ENSG00000005436	GCFC2	1.2192	4.79E-06	1.36E-05	3.0489
ENSG00000100813	ACIN1	1.2243	4.80E-06	1.36E-05	3.0479
ENSG00000237765	FAM200B	1.2514	4.82E-06	1.36E-05	3.0436
ENSG00000118971	CCND2	2.7915	4.82E-06	1.37E-05	3.0428
ENSG00000072818	ACAP1	1.3337	4.83E-06	1.37E-05	3.0417
ENSG00000137198	GMPR	0.7112	4.84E-06	1.37E-05	3.0382
ENSG00000185787	MORF4L1	1.1757	4.86E-06	1.38E-05	3.0350
ENSG00000140795	MYLK3	1.2417	4.87E-06	1.38E-05	3.0339
ENSG00000163491	NEK10	1.1155	4.91E-06	1.39E-05	3.0246
ENSG00000005844	ITGAL	1.5764	4.96E-06	1.40E-05	3.0150
ENSG00000188580	NKAIN2	1.2433	4.98E-06	1.41E-05	3.0119
ENSG00000107338	SHB	1.2632	4.99E-06	1.41E-05	3.0105
ENSG00000161973	CCDC42	1.0715	4.99E-06	1.41E-05	3.0104
ENSG00000170638	TRABD	1.2066	5.01E-06	1.42E-05	3.0066
ENSG00000102786	INTS6	1.1817	5.02E-06	1.42E-05	3.0043
ENSG00000118596	SLC16A7	1.3126	5.03E-06	1.42E-05	3.0018
ENSG00000130758	MAP3K10	1.2088	5.04E-06	1.42E-05	3.0006
ENSG00000163884	KLF15	1.3587	5.05E-06	1.43E-05	2.9977
ENSG00000122691	TWIST1	1.2493	5.08E-06	1.44E-05	2.9920
ENSG00000180616	SSTR2	1.1772	5.10E-06	1.44E-05	2.9886
ENSG00000168301	KCTD6	1.1744	5.12E-06	1.45E-05	2.9846
ENSG00000127990	SGCE	1.7063	5.14E-06	1.45E-05	2.9810
ENSG00000105141	CASP14	1.0478	5.15E-06	1.46E-05	2.9786
ENSG00000168300	PCMTD1	0.7741	5.16E-06	1.46E-05	2.9779
ENSG00000112769	LAMA4	1.1362	5.21E-06	1.47E-05	2.9693
ENSG00000184368	MAP7D2	1.0746	5.21E-06	1.47E-05	2.9691
ENSG00000221887	HMSD	1.2143	5.21E-06	1.47E-05	2.9682
ENSG00000151806	GUF1	1.1985	5.26E-06	1.48E-05	2.9593
ENSG00000175182	FAM131A	1.2108	5.27E-06	1.49E-05	2.9576
ENSG00000131018	SYNE1	1.2849	5.27E-06	1.49E-05	2.9568
ENSG00000010361	FUZ	1.2232	5.28E-06	1.49E-05	2.9558
ENSG00000136045	PWP1	1.1583	5.30E-06	1.49E-05	2.9529
ENSG00000159214	CCDC24	1.3000	5.36E-06	1.51E-05	2.9411
ENSG00000198556	ZNF789	1.2046	5.37E-06	1.51E-05	2.9400
ENSG00000105982	RNF32	1.3170	5.38E-06	1.52E-05	2.9377
ENSG00000256060	TRAPPC2B	0.8308	5.41E-06	1.52E-05	2.9330
ENSG00000140092	FBLN5	1.1170	5.43E-06	1.53E-05	2.9281
ENSG00000149090	PAMR1	1.0490	5.43E-06	1.53E-05	2.9280
ENSG00000141338	ABCA8	1.1364	5.49E-06	1.55E-05	2.9181
ENSG00000078967	UBE2D4	0.8188	5.52E-06	1.56E-05	2.9127
ENSG00000185291	IL3RA	1.3847	5.53E-06	1.56E-05	2.9120
ENSG00000155816	FMN2	1.0226	5.53E-06	1.56E-05	2.9108
ENSG00000267221	C17orf113	0.7893	5.54E-06	1.56E-05	2.9096
ENSG00000121380	BCL2L14	1.2308	5.55E-06	1.56E-05	2.9081
ENSG00000138185	ENTPD1	1.4242	5.55E-06	1.56E-05	2.9071
ENSG00000204025	TRPC5OS	1.1096	5.57E-06	1.57E-05	2.9039
ENSG00000137145	DENND4C	1.2965	5.60E-06	1.57E-05	2.8998
ENSG00000162641	AKNAD1	1.0437	5.62E-06	1.58E-05	2.8955
ENSG00000167315	ACAA2	1.1893	5.63E-06	1.58E-05	2.8944
ENSG00000168924	LETM1	1.2767	5.64E-06	1.59E-05	2.8928
ENSG00000156587	UBE2L6	0.7796	5.66E-06	1.59E-05	2.8899

Gene ID	Gene Symbol	FC	P.Value	adj.P.Val	B
ENSG00000130226	DPP6	1.0508	5.66E-06	1.59E-05	2.8889
ENSG00000183960	KCNH8	1.1566	5.68E-06	1.60E-05	2.8851
ENSG00000240303	ACAD11	1.2619	5.69E-06	1.60E-05	2.8848
ENSG00000147596	PRDM14	1.0626	5.69E-06	1.60E-05	2.8834
ENSG00000100280	AP1B1	1.2089	5.70E-06	1.60E-05	2.8828
ENSG00000126790	L3HYPDH	0.7008	5.70E-06	1.60E-05	2.8819
ENSG00000142224	IL19	1.0462	5.75E-06	1.61E-05	2.8747
ENSG00000151006	PRSS53	1.1594	5.78E-06	1.62E-05	2.8690
ENSG00000255374	TAS2R43	1.0494	5.79E-06	1.63E-05	2.8673
ENSG00000100124	ANKRD54	1.1766	5.80E-06	1.63E-05	2.8649
ENSG00000122550	KLHL7	1.1804	5.83E-06	1.64E-05	2.8604
ENSG00000215018	COL28A1	1.1333	5.83E-06	1.64E-05	2.8600
ENSG00000121067	SPOP	1.2067	5.86E-06	1.64E-05	2.8563
ENSG00000100319	ZMAT5	0.8064	5.88E-06	1.65E-05	2.8522
ENSG00000130943	PKDREJ	1.0331	5.88E-06	1.65E-05	2.8522
ENSG00000114395	CYB561D2	0.8385	5.89E-06	1.65E-05	2.8505
ENSG00000242715	CCDC169	0.6997	5.90E-06	1.66E-05	2.8486
ENSG00000177034	MTX3	1.3154	5.93E-06	1.66E-05	2.8451
ENSG00000179240	GVQW3	0.7787	5.94E-06	1.66E-05	2.8434
ENSG00000124159	MATN4	1.0500	5.95E-06	1.67E-05	2.8409
ENSG00000104313	EYA1	1.3524	5.96E-06	1.67E-05	2.8390
ENSG00000072832	CRMP1	1.2333	5.98E-06	1.68E-05	2.8369
ENSG00000188186	LAMTOR4	0.8730	6.01E-06	1.68E-05	2.8324
ENSG00000268223	ARL14EPL	1.0462	6.01E-06	1.68E-05	2.8314
ENSG00000006125	AP2B1	1.2068	6.06E-06	1.70E-05	2.8237
ENSG00000198944	SOWAHA	1.0846	6.06E-06	1.70E-05	2.8230
ENSG00000188419	CHM	1.2236	6.08E-06	1.70E-05	2.8202
ENSG00000138606	SHF	0.8526	6.10E-06	1.71E-05	2.8168
ENSG00000172046	USP19	1.1785	6.13E-06	1.72E-05	2.8128
ENSG00000050767	COL23A1	1.0546	6.21E-06	1.74E-05	2.8003
ENSG00000170537	TMC7	1.0723	6.22E-06	1.74E-05	2.7992
ENSG00000138669	PRKG2	1.1407	6.24E-06	1.75E-05	2.7964
ENSG00000162735	PEX19	1.2586	6.25E-06	1.75E-05	2.7946
ENSG00000257365	FNTB	1.2056	6.26E-06	1.75E-05	2.7927
ENSG00000129480	DTD2	0.7643	6.26E-06	1.75E-05	2.7923
ENSG00000077549	CAPZB	1.1836	6.27E-06	1.75E-05	2.7914
ENSG00000117751	PPP1R8	1.1772	6.28E-06	1.76E-05	2.7891
ENSG00000143079	CTTNBP2NL	1.0922	6.28E-06	1.76E-05	2.7890
ENSG00000138036	DYNC2LI1	1.2387	6.30E-06	1.76E-05	2.7865
ENSG00000104783	KCNN4	1.9533	6.31E-06	1.76E-05	2.7853
ENSG00000105427	CNFN	1.1878	6.33E-06	1.77E-05	2.7813
ENSG00000114767	RRP9	0.7902	6.34E-06	1.77E-05	2.7806
ENSG00000111325	OGFOD2	1.1778	6.36E-06	1.78E-05	2.7775
ENSG00000115459	ELMOD3	1.1841	6.36E-06	1.78E-05	2.7770
ENSG00000130762	ARHGEF16	0.7279	6.38E-06	1.78E-05	2.7750
ENSG00000087299	L2HGDH	1.2019	6.40E-06	1.79E-05	2.7722
ENSG00000137766	UNC13C	1.2263	6.41E-06	1.79E-05	2.7705
ENSG00000107341	UBE2R2	1.1588	6.43E-06	1.79E-05	2.7672
ENSG00000134909	ARHGAP32	1.3298	6.43E-06	1.79E-05	2.7671
ENSG00000130584	ZBTB46	1.2673	6.46E-06	1.80E-05	2.7622
ENSG00000125398	SOX9	1.1572	6.49E-06	1.81E-05	2.7582
ENSG00000064419	TNPO3	1.1714	6.53E-06	1.82E-05	2.7529
ENSG00000165621	OXGR1	1.0690	6.54E-06	1.82E-05	2.7513
ENSG00000122188	LAX1	0.6498	6.56E-06	1.83E-05	2.7479

Gene ID	Gene Symbol	FC	P.Value	adj.P.Val	B
ENSG00000187066	TMEM262	1.1671	6.59E-06	1.84E-05	2.7432
ENSG00000121073	SLC35B1	1.1871	6.61E-06	1.84E-05	2.7401
ENSG00000111490	TBC1D30	1.5026	6.67E-06	1.86E-05	2.7320
ENSG00000088899	LZTS3	1.4380	6.68E-06	1.86E-05	2.7300
ENSG00000105894	PTN	0.7114	6.76E-06	1.88E-05	2.7191
ENSG00000203697	CAPN8	1.2318	6.78E-06	1.89E-05	2.7169
ENSG00000203747	FCGR3A	0.5712	6.81E-06	1.90E-05	2.7116
ENSG00000074842	MYDGF	0.8082	6.83E-06	1.90E-05	2.7099
ENSG00000128203	ASPHD2	0.7702	6.83E-06	1.90E-05	2.7095
ENSG00000183060	LYSMD4	1.2603	6.83E-06	1.90E-05	2.7088
ENSG00000180332	KCTD4	0.9664	6.84E-06	1.91E-05	2.7074
ENSG00000138303	ASCC1	1.1906	6.85E-06	1.91E-05	2.7068
ENSG00000147955	SIGMAR1	0.7857	6.85E-06	1.91E-05	2.7060
ENSG00000118960	HS1BP3	1.2022	6.87E-06	1.91E-05	2.7039
ENSG00000163154	TNFAIP8L2	0.7916	6.88E-06	1.92E-05	2.7022
ENSG00000139117	CPNE8	1.5884	6.88E-06	1.92E-05	2.7017
ENSG00000105255	FSD1	1.0556	6.89E-06	1.92E-05	2.7011
ENSG00000168754	FAM178B	1.1983	6.90E-06	1.92E-05	2.7001
ENSG00000132122	SPATA6	1.1302	6.91E-06	1.92E-05	2.6978
ENSG00000148187	MRRF	0.8353	6.92E-06	1.92E-05	2.6967
ENSG00000117448	AKR1A1	0.8460	6.93E-06	1.93E-05	2.6954
ENSG00000198342	ZNF442	0.8166	6.93E-06	1.93E-05	2.6950
ENSG00000007314	SCN4A	1.0805	6.96E-06	1.94E-05	2.6909
ENSG00000124104	SNX21	1.1973	7.01E-06	1.95E-05	2.6845
ENSG00000116885	OSCP1	1.2426	7.03E-06	1.95E-05	2.6815
ENSG00000198959	TGM2	1.1650	7.04E-06	1.96E-05	2.6810
ENSG00000003096	KLHL13	1.1529	7.06E-06	1.96E-05	2.6780
ENSG00000153446	C16orf89	1.0658	7.09E-06	1.97E-05	2.6733
ENSG00000099769	IGFALS	1.1737	7.11E-06	1.98E-05	2.6703
ENSG00000131771	PPP1R1B	1.1063	7.14E-06	1.98E-05	2.6675
ENSG00000114626	ABTB1	1.2420	7.14E-06	1.98E-05	2.6662
ENSG00000163462	TRIM46	1.2247	7.16E-06	1.99E-05	2.6648
ENSG00000196862	RGPD4	1.0169	7.19E-06	2.00E-05	2.6599
ENSG00000183340	JRKL	0.7761	7.22E-06	2.00E-05	2.6563
ENSG00000205097	FRG2	1.0355	7.26E-06	2.02E-05	2.6506
ENSG00000069018	TRPC7	1.0764	7.30E-06	2.03E-05	2.6459
ENSG00000100012	SEC14L3	1.0523	7.31E-06	2.03E-05	2.6450
ENSG00000114698	PLSCR4	1.3141	7.31E-06	2.03E-05	2.6445
ENSG00000072840	EVC	1.3538	7.32E-06	2.03E-05	2.6430
ENSG00000101898	MCTS2P	1.2166	7.33E-06	2.03E-05	2.6413
ENSG00000114757	PEX5L	1.1980	7.34E-06	2.04E-05	2.6403
ENSG00000101138	CSTF1	1.1905	7.34E-06	2.04E-05	2.6398
ENSG00000070540	WIPI1	0.7866	7.37E-06	2.04E-05	2.6366
ENSG00000130158	DOCK6	1.3324	7.39E-06	2.05E-05	2.6345
ENSG00000075239	ACAT1	0.8351	7.42E-06	2.06E-05	2.6297
ENSG00000099256	PRTFDC1	1.2908	7.42E-06	2.06E-05	2.6298
ENSG00000196421	C20orf204	1.1050	7.44E-06	2.06E-05	2.6275
ENSG00000161057	PSMC2	1.1733	7.48E-06	2.07E-05	2.6226
ENSG00000166415	WDR72	1.3221	7.49E-06	2.07E-05	2.6212
ENSG00000203908	KHDC3L	1.0117	7.50E-06	2.08E-05	2.6203
ENSG00000162592	CCDC27	1.0138	7.53E-06	2.08E-05	2.6158
ENSG00000196497	IPO4	1.2027	7.53E-06	2.08E-05	2.6158
ENSG00000109991	P2RX3	1.0629	7.55E-06	2.09E-05	2.6138
ENSG00000198791	CNOT7	1.1978	7.56E-06	2.09E-05	2.6118

Gene ID	Gene Symbol	FC	P.Value	adj.P.Val	B
ENSG00000176018	LYSMD3	1.1799	7.58E-06	2.10E-05	2.6094
ENSG00000131183	SLC34A1	0.9835	7.59E-06	2.10E-05	2.6085
ENSG00000186687	LYRM7	0.7789	7.59E-06	2.10E-05	2.6083
ENSG00000179938	GOLGA8J	1.1146	7.62E-06	2.11E-05	2.6050
ENSG00000073050	XRCC1	1.1817	7.63E-06	2.11E-05	2.6028
ENSG00000205929	C21orf62	0.7800	7.64E-06	2.11E-05	2.6020
ENSG00000143450	OAZ3	1.1725	7.66E-06	2.12E-05	2.5996
ENSG00000114450	GNB4	1.3119	7.67E-06	2.12E-05	2.5980
ENSG00000143630	HCN3	1.1961	7.69E-06	2.13E-05	2.5960
ENSG00000162368	CMPK1	1.1565	7.70E-06	2.13E-05	2.5949
ENSG00000188092	GPR89B	1.2279	7.71E-06	2.13E-05	2.5939
ENSG00000175564	UCP3	1.2454	7.71E-06	2.13E-05	2.5928
ENSG00000158055	GRHL3	1.1960	7.75E-06	2.14E-05	2.5887
ENSG00000088876	ZNF343	1.1890	7.75E-06	2.14E-05	2.5884
ENSG00000115233	PSMD14	1.1973	7.75E-06	2.14E-05	2.5880
ENSG00000137948	BRDT	1.1580	7.80E-06	2.15E-05	2.5820
ENSG00000165684	SNAPC4	1.1749	7.82E-06	2.16E-05	2.5802
ENSG00000101444	AHCY	0.8094	7.84E-06	2.16E-05	2.5775
ENSG00000054965	FAM168A	1.1970	7.85E-06	2.17E-05	2.5762
ENSG00000072133	RPS6KA6	1.4428	7.88E-06	2.17E-05	2.5730
ENSG00000107789	MINPP1	0.7503	7.90E-06	2.18E-05	2.5704
ENSG00000120725	SIL1	0.8278	7.91E-06	2.18E-05	2.5688
ENSG00000125703	ATG4C	0.7330	7.91E-06	2.18E-05	2.5688
ENSG00000145730	PAM	0.7906	7.92E-06	2.18E-05	2.5683
ENSG00000168438	CDC40	1.1929	7.95E-06	2.19E-05	2.5646
ENSG00000071054	MAP4K4	1.6848	7.98E-06	2.20E-05	2.5600
ENSG00000102802	MEDAG	1.1301	7.99E-06	2.20E-05	2.5591
ENSG00000068394	GPKOW	1.2071	8.04E-06	2.22E-05	2.5530
ENSG00000155367	PPM1J	1.0952	8.07E-06	2.22E-05	2.5498
ENSG00000169894	MUC3A	1.4879	8.10E-06	2.23E-05	2.5466
ENSG00000013619	MAMLD1	1.4075	8.13E-06	2.24E-05	2.5425
ENSG00000133706	LARS1	0.7246	8.14E-06	2.24E-05	2.5417
ENSG00000174885	NLRP6	1.3967	8.17E-06	2.25E-05	2.5376
ENSG00000179144	GIMAP7	0.8000	8.18E-06	2.25E-05	2.5367
ENSG00000163624	CDS1	1.2247	8.20E-06	2.26E-05	2.5349
ENSG00000182154	MRPL41	0.7971	8.20E-06	2.26E-05	2.5341
ENSG00000178163	ZNF518B	1.3247	8.21E-06	2.26E-05	2.5336
ENSG00000119614	VSX2	1.0139	8.24E-06	2.27E-05	2.5294
ENSG00000167984	NLRC3	1.2294	8.25E-06	2.27E-05	2.5282
ENSG00000162849	KIF26B	1.0732	8.26E-06	2.27E-05	2.5280
ENSG00000118263	KLF7	1.4183	8.27E-06	2.28E-05	2.5259
ENSG00000131979	GCH1	1.3344	8.38E-06	2.30E-05	2.5136
ENSG00000162032	SPSB3	1.2091	8.38E-06	2.30E-05	2.5138
ENSG00000182318	ZSCAN22	0.8646	8.38E-06	2.30E-05	2.5136
ENSG00000165731	RET	1.0574	8.39E-06	2.31E-05	2.5123
ENSG00000115091	ACTR3	1.2219	8.41E-06	2.31E-05	2.5100
ENSG00000011638	LDAF1	1.2160	8.43E-06	2.32E-05	2.5078
ENSG00000170209	ANKK1	0.7792	8.44E-06	2.32E-05	2.5066
ENSG00000121310	ECHDC2	0.6751	8.46E-06	2.33E-05	2.5041
ENSG00000140105	WARS1	1.4878	8.50E-06	2.34E-05	2.4997
ENSG00000172331	BPGM	1.2181	8.52E-06	2.34E-05	2.4979
ENSG00000118307	DNAI7	1.0992	8.52E-06	2.34E-05	2.4978
ENSG00000138795	LEF1	1.6617	8.53E-06	2.34E-05	2.4967
ENSG00000171747	LGALS4	1.1982	8.53E-06	2.34E-05	2.4967

Gene ID	Gene Symbol	FC	P.Value	adj.P.Val	B
ENSG00000167995	BEST1	1.3058	8.54E-06	2.35E-05	2.4952
ENSG00000073067	CYP2W1	1.0970	8.59E-06	2.36E-05	2.4903
ENSG00000083838	ZNF446	1.1816	8.61E-06	2.36E-05	2.4876
ENSG00000111832	RWDD1	0.8550	8.62E-06	2.37E-05	2.4864
ENSG00000128342	LIF	1.0694	8.65E-06	2.37E-05	2.4837
ENSG00000170633	RNF34	1.1830	8.66E-06	2.38E-05	2.4823
ENSG00000095066	HOOK2	0.7702	8.73E-06	2.39E-05	2.4745
ENSG00000152213	ARL11	0.8063	8.78E-06	2.41E-05	2.4687
ENSG00000174282	ZBTB4	1.2355	8.82E-06	2.42E-05	2.4647
ENSG00000185338	SOCS1	1.4814	8.86E-06	2.43E-05	2.4600
ENSG00000197933	ZNF823	1.1604	8.89E-06	2.44E-05	2.4572
ENSG00000169856	ONECUT1	1.0747	8.91E-06	2.44E-05	2.4551
ENSG00000166211	SPIC	0.8186	8.91E-06	2.44E-05	2.4549
ENSG00000137824	RMDN3	0.8295	8.95E-06	2.45E-05	2.4513
ENSG00000034693	PEX3	0.7667	8.96E-06	2.45E-05	2.4496
ENSG00000109861	CTSC	0.7476	8.96E-06	2.45E-05	2.4496
ENSG00000100629	CEP128	1.4292	8.98E-06	2.46E-05	2.4471
ENSG00000125968	ID1	1.6222	9.00E-06	2.46E-05	2.4454
ENSG00000182195	LDOC1	1.7285	9.02E-06	2.47E-05	2.4431
ENSG00000102053	ZC3H12B	1.1399	9.03E-06	2.47E-05	2.4423
ENSG00000135341	MAP3K7	1.1899	9.07E-06	2.48E-05	2.4381
ENSG00000188312	CENPP	1.1551	9.09E-06	2.49E-05	2.4359
ENSG00000167508	MVD	1.2637	9.10E-06	2.49E-05	2.4346
ENSG00000121486	TRMT1L	1.2498	9.11E-06	2.49E-05	2.4336
ENSG00000109881	CCDC34	1.3161	9.12E-06	2.49E-05	2.4333
ENSG00000206203	TSSK2	1.0367	9.12E-06	2.50E-05	2.4325
ENSG00000127955	GNAI1	1.2952	9.16E-06	2.51E-05	2.4287
ENSG00000126001	CEP250	1.2527	9.22E-06	2.52E-05	2.4227
ENSG00000151498	ACAD8	1.1847	9.26E-06	2.53E-05	2.4180
ENSG00000241635	UGT1A1	1.1142	9.30E-06	2.54E-05	2.4142
ENSG00000102038	SMARCA1	1.3336	9.32E-06	2.55E-05	2.4123
ENSG00000135541	AHI1	1.3057	9.32E-06	2.55E-05	2.4118
ENSG00000181894	ZNF329	1.3341	9.33E-06	2.55E-05	2.4109
ENSG00000176978	DPP7	0.7788	9.36E-06	2.56E-05	2.4076
ENSG00000109971	HSPA8	0.7621	9.42E-06	2.57E-05	2.4023
ENSG00000113273	ARSB	1.1982	9.42E-06	2.57E-05	2.4019
ENSG00000197728	RPS26	1.1807	9.43E-06	2.58E-05	2.4005
ENSG00000107672	NSMCE4A	1.1533	9.44E-06	2.58E-05	2.3999
ENSG00000125775	SDCBP2	1.2043	9.46E-06	2.58E-05	2.3981
ENSG00000163263	C1orf189	1.1257	9.47E-06	2.59E-05	2.3966
ENSG00000176371	ZSCAN2	1.2382	9.58E-06	2.61E-05	2.3862
ENSG00000099958	DERL3	0.7085	9.68E-06	2.64E-05	2.3761
ENSG00000167118	URM1	0.8590	9.71E-06	2.65E-05	2.3730
ENSG00000183837	PNMA3	1.2864	9.71E-06	2.65E-05	2.3730
ENSG00000150782	IL18	0.7230	9.73E-06	2.65E-05	2.3715
ENSG00000196371	FUT4	1.2405	9.83E-06	2.68E-05	2.3615
ENSG00000073598	FNDC8	1.0651	9.83E-06	2.68E-05	2.3612
ENSG00000111057	KRT18	1.6108	9.83E-06	2.68E-05	2.3613
ENSG00000077463	SIRT6	1.1846	9.85E-06	2.69E-05	2.3590
ENSG00000145936	KCNMB1	1.0800	9.90E-06	2.70E-05	2.3541
ENSG00000196131	VN1R2	1.0596	9.94E-06	2.71E-05	2.3509
ENSG00000100296	THOC5	1.1880	9.95E-06	2.71E-05	2.3492
ENSG00000168916	ZNF608	1.5368	9.95E-06	2.71E-05	2.3493
ENSG00000162704	ARPC5	1.3032	9.98E-06	2.72E-05	2.3468

Gene ID	Gene Symbol	FC	P.Value	adj.P.Val	B
ENSG00000122008	POLK	1.1793	1.00E-05	2.73E-05	2.3432
ENSG00000235173	HGH1	0.7152	1.00E-05	2.73E-05	2.3417
ENSG00000125895	TMEM74B	1.1879	1.00E-05	2.73E-05	2.3413
ENSG00000141424	SLC39A6	1.2426	1.00E-05	2.73E-05	2.3412
ENSG00000138823	MTTP	1.0851	1.01E-05	2.74E-05	2.3369
ENSG00000174600	CMKLR1	0.7812	1.01E-05	2.75E-05	2.3336
ENSG00000155621	C9orf85	0.8710	1.02E-05	2.77E-05	2.3274
ENSG00000172301	COPRS	1.2052	1.02E-05	2.78E-05	2.3229
ENSG00000182518	FAM104B	1.1758	1.02E-05	2.78E-05	2.3229
ENSG00000234906	APOC2	1.1482	1.03E-05	2.79E-05	2.3208
ENSG00000188624	IGFL3	1.0950	1.03E-05	2.79E-05	2.3190
ENSG00000179262	RAD23A	1.1440	1.03E-05	2.80E-05	2.3182
ENSG00000171608	PIK3CD	1.3317	1.03E-05	2.80E-05	2.3176
ENSG00000165457	FOLR2	0.6027	1.03E-05	2.80E-05	2.3173
ENSG00000148677	ANKRD1	1.0216	1.03E-05	2.80E-05	2.3156
ENSG00000131482	G6PC1	1.0334	1.03E-05	2.81E-05	2.3143
ENSG00000188076	SCGB1C1	1.1172	1.03E-05	2.81E-05	2.3125
ENSG00000134250	NOTCH2	1.3438	1.04E-05	2.81E-05	2.3114
ENSG00000126217	MCF2L	1.5111	1.04E-05	2.82E-05	2.3096
ENSG00000057468	MSH4	0.8902	1.04E-05	2.82E-05	2.3079
ENSG00000124374	PAIP2B	1.3353	1.04E-05	2.83E-05	2.3049
ENSG00000173702	MUC13	1.3364	1.04E-05	2.84E-05	2.3032
ENSG00000139549	DHH	1.0131	1.05E-05	2.84E-05	2.3012
ENSG00000186815	TPCN1	1.2787	1.05E-05	2.85E-05	2.2993
ENSG00000025039	RRAGD	0.7511	1.05E-05	2.86E-05	2.2963
ENSG00000173040	EVC2	1.2412	1.05E-05	2.86E-05	2.2944
ENSG00000176974	SHMT1	1.3178	1.06E-05	2.87E-05	2.2903
ENSG00000119912	IDE	1.2136	1.06E-05	2.88E-05	2.2883
ENSG00000118804	STBD1	1.4845	1.06E-05	2.88E-05	2.2872
ENSG00000157978	LDLRAP1	1.3094	1.06E-05	2.88E-05	2.2866
ENSG00000162384	CZIB	0.8186	1.06E-05	2.88E-05	2.2862
ENSG00000182601	HS3ST4	0.7377	1.06E-05	2.89E-05	2.2848
ENSG00000262165	C17orf114	1.1327	1.07E-05	2.89E-05	2.2846
ENSG00000182405	PGBD4	0.7913	1.07E-05	2.89E-05	2.2844
ENSG00000007237	GAS7	1.3253	1.07E-05	2.89E-05	2.2822
ENSG00000139910	NOVA1	1.1670	1.07E-05	2.91E-05	2.2770
ENSG00000012171	SEMA3B	1.2460	1.08E-05	2.92E-05	2.2727
ENSG00000163492	CCDC141	1.1674	1.08E-05	2.92E-05	2.2723
ENSG00000073578	SDHA	1.1670	1.08E-05	2.92E-05	2.2721
ENSG00000165583	SSX5	1.1913	1.08E-05	2.94E-05	2.2677
ENSG00000077514	POLD3	1.2700	1.09E-05	2.95E-05	2.2644
ENSG00000132016	BRME1	1.3352	1.09E-05	2.95E-05	2.2640
ENSG00000232593	KANTR	1.2149	1.09E-05	2.96E-05	2.2597
ENSG00000197093	GAL3ST4	1.1482	1.10E-05	2.97E-05	2.2576
ENSG00000124532	MRS2	0.7794	1.10E-05	2.98E-05	2.2536
ENSG00000170745	KCNS3	0.4499	1.11E-05	3.01E-05	2.2436
ENSG00000165828	PRAP1	1.1619	1.11E-05	3.01E-05	2.2428
ENSG00000121931	LRIF1	0.7695	1.11E-05	3.01E-05	2.2419
ENSG00000067369	TP53BP1	1.1865	1.12E-05	3.02E-05	2.2385
ENSG00000168000	BSCL2	0.7405	1.12E-05	3.04E-05	2.2346
ENSG00000155495	MAGEC1	0.5436	1.13E-05	3.05E-05	2.2314
ENSG00000110330	BIRC2	1.2680	1.13E-05	3.06E-05	2.2269
ENSG00000153558	FBXL2	1.1973	1.13E-05	3.06E-05	2.2263
ENSG00000109854	HTATIP2	0.7737	1.13E-05	3.06E-05	2.2259

Gene ID	Gene Symbol	FC	P.Value	adj.P.Val	B
ENSG00000162777	DENND2D	0.7751	1.13E-05	3.06E-05	2.2251
ENSG00000117533	VAMP4	1.1784	1.14E-05	3.07E-05	2.2228
ENSG00000239672	NME1	0.7938	1.14E-05	3.08E-05	2.2204
ENSG00000172663	TMEM134	1.2056	1.14E-05	3.08E-05	2.2192
ENSG00000127507	ADGRE2	1.2777	1.15E-05	3.10E-05	2.2147
ENSG00000185905	C16orf54	1.5503	1.15E-05	3.11E-05	2.2111
ENSG00000171773	NXNL1	1.0476	1.15E-05	3.11E-05	2.2093
ENSG00000119771	KLHL29	1.0926	1.15E-05	3.11E-05	2.2091
ENSG00000204389	HSPA1A	1.7061	1.16E-05	3.13E-05	2.2040
ENSG00000205693	MANSC4	1.0377	1.16E-05	3.14E-05	2.2001
ENSG00000160326	SLC2A6	1.3030	1.17E-05	3.16E-05	2.1940
ENSG00000154930	ACSS1	0.7672	1.18E-05	3.17E-05	2.1899
ENSG00000111886	GABRR2	1.3799	1.18E-05	3.18E-05	2.1891
ENSG00000197971	MBP	1.3396	1.18E-05	3.18E-05	2.1886
ENSG00000168679	SLC16A4	1.1093	1.18E-05	3.19E-05	2.1854
ENSG00000094755	GABRP	1.2876	1.18E-05	3.19E-05	2.1841
ENSG00000134365	CFHR4	1.1081	1.18E-05	3.19E-05	2.1839
ENSG00000069424	KCNAB2	1.2098	1.19E-05	3.20E-05	2.1798
ENSG00000182141	ZNF708	1.3325	1.19E-05	3.21E-05	2.1775
ENSG00000114686	MRPL3	0.8437	1.19E-05	3.22E-05	2.1753
ENSG00000178922	HYI	1.3649	1.20E-05	3.22E-05	2.1746
ENSG00000204767	INSYN2B	1.0531	1.20E-05	3.24E-05	2.1695
ENSG00000115705	TPO	1.2252	1.20E-05	3.24E-05	2.1688
ENSG00000100815	TRIP11	1.1898	1.21E-05	3.25E-05	2.1645
ENSG00000065883	CDK13	1.1800	1.21E-05	3.27E-05	2.1596
ENSG00000140992	PDPK1	1.1885	1.21E-05	3.27E-05	2.1595
ENSG00000166398	GARRE1	1.1880	1.22E-05	3.27E-05	2.1584
ENSG00000213390	ARHGAP19	1.1931	1.22E-05	3.28E-05	2.1569
ENSG00000116473	RAP1A	1.2414	1.22E-05	3.28E-05	2.1548
ENSG00000170632	ARMC10	0.8119	1.22E-05	3.28E-05	2.1548
ENSG00000162241	SLC25A45	0.7582	1.22E-05	3.29E-05	2.1543
ENSG00000114656	CFAP92	1.1531	1.22E-05	3.29E-05	2.1535
ENSG00000167034	NKX3-1	1.0764	1.23E-05	3.30E-05	2.1487
ENSG00000117091	CD48	0.7012	1.23E-05	3.31E-05	2.1480
ENSG00000164663	USP49	1.3488	1.23E-05	3.31E-05	2.1454
ENSG00000163701	IL17RE	1.2160	1.24E-05	3.33E-05	2.1422
ENSG00000130962	PRRG1	1.0518	1.24E-05	3.33E-05	2.1414
ENSG00000089177	KIF16B	1.2334	1.24E-05	3.33E-05	2.1409
ENSG00000214513	NOTO	1.0425	1.24E-05	3.34E-05	2.1374
ENSG00000257108	NHLRC4	0.8320	1.24E-05	3.34E-05	2.1370
ENSG00000129910	CDH15	1.2532	1.24E-05	3.35E-05	2.1357
ENSG00000101445	PPP1R16B	1.5158	1.25E-05	3.36E-05	2.1322
ENSG00000139531	SUOX	1.2089	1.25E-05	3.36E-05	2.1305
ENSG00000140280	LYSMD2	0.6255	1.25E-05	3.37E-05	2.1296
ENSG00000189227	C15orf61	1.1515	1.26E-05	3.38E-05	2.1258
ENSG00000166130	IKBIP	0.7810	1.26E-05	3.39E-05	2.1211
ENSG00000185567	AHNAK2	1.0879	1.28E-05	3.43E-05	2.1110
ENSG00000119125	GDA	1.1537	1.28E-05	3.44E-05	2.1083
ENSG00000166750	SLFN5	1.4376	1.28E-05	3.44E-05	2.1074
ENSG00000020922	MRE11	1.2447	1.29E-05	3.46E-05	2.1026
ENSG00000164209	SLC25A46	1.1444	1.29E-05	3.47E-05	2.1007
ENSG00000164885	CDK5	0.8030	1.30E-05	3.48E-05	2.0965
ENSG00000115592	PRKAG3	1.0490	1.30E-05	3.48E-05	2.0953
ENSG00000110811	P3H3	0.5910	1.30E-05	3.49E-05	2.0928

Gene ID	Gene Symbol	FC	P.Value	adj.P.Val	B
ENSG00000136810	TXN	0.8192	1.30E-05	3.50E-05	2.0909
ENSG00000174780	SRP72	1.1715	1.31E-05	3.52E-05	2.0843
ENSG00000177511	ST8SIA3	1.0986	1.32E-05	3.53E-05	2.0827
ENSG00000111850	SMIM8	0.8132	1.32E-05	3.54E-05	2.0805
ENSG00000144847	IGSF11	0.6636	1.32E-05	3.54E-05	2.0805
ENSG00000115884	SDC1	0.6853	1.32E-05	3.54E-05	2.0783
ENSG00000100335	MIEF1	1.2001	1.32E-05	3.55E-05	2.0764
ENSG00000125817	CENPB	1.1873	1.33E-05	3.55E-05	2.0750
ENSG00000137819	PAQR5	1.0655	1.33E-05	3.55E-05	2.0748
ENSG00000198715	GLMP	1.3132	1.33E-05	3.56E-05	2.0736
ENSG00000229674	H2AL3	1.0386	1.33E-05	3.56E-05	2.0733
ENSG00000146233	CYP39A1	1.2131	1.33E-05	3.56E-05	2.0729
ENSG00000140497	SCAMP2	0.8306	1.33E-05	3.56E-05	2.0722
ENSG00000105695	MAG	1.0635	1.33E-05	3.56E-05	2.0717
ENSG00000167550	RHEBL1	1.2520	1.33E-05	3.56E-05	2.0713
ENSG00000165076	PRSS37	0.9668	1.34E-05	3.58E-05	2.0683
ENSG00000130037	KCNA5	0.6460	1.34E-05	3.58E-05	2.0681
ENSG00000126860	EVI2A	0.7537	1.34E-05	3.58E-05	2.0675
ENSG00000143106	PSMA5	0.8337	1.34E-05	3.60E-05	2.0624
ENSG00000120697	ALG5	0.8256	1.35E-05	3.61E-05	2.0591
ENSG00000106624	AEBP1	1.6435	1.36E-05	3.63E-05	2.0534
ENSG00000037474	NSUN2	1.1624	1.36E-05	3.63E-05	2.0531
ENSG00000197892	KIF13B	1.2454	1.36E-05	3.64E-05	2.0510
ENSG00000160298	C21orf58	1.3086	1.37E-05	3.65E-05	2.0466
ENSG00000183281	PLGLB1	1.3015	1.38E-05	3.70E-05	2.0356
ENSG00000166855	CLPX	1.1917	1.38E-05	3.70E-05	2.0347
ENSG00000128266	GNAZ	1.2588	1.39E-05	3.70E-05	2.0338
ENSG00000137691	CFAP300	1.0416	1.39E-05	3.70E-05	2.0333
ENSG00000117601	SERPINC1	1.0483	1.39E-05	3.71E-05	2.0317
ENSG00000074966	TXK	1.2695	1.39E-05	3.71E-05	2.0312
ENSG00000186204	CYP4F12	1.2010	1.39E-05	3.72E-05	2.0292
ENSG00000126870	DYNC2I1	1.2112	1.39E-05	3.72E-05	2.0285
ENSG00000183153	GJD3	1.1205	1.40E-05	3.74E-05	2.0241
ENSG00000188396	DYNLT4	1.1511	1.40E-05	3.74E-05	2.0223
ENSG00000146038	DCDC2	1.0534	1.41E-05	3.75E-05	2.0200
ENSG00000132773	TOE1	1.1964	1.41E-05	3.75E-05	2.0195
ENSG00000087302	RTRAF	0.8839	1.41E-05	3.76E-05	2.0181
ENSG00000142544	CTU1	0.8160	1.41E-05	3.76E-05	2.0162
ENSG00000143257	NR1I3	1.1234	1.41E-05	3.77E-05	2.0139
ENSG00000164933	SLC25A32	1.1715	1.42E-05	3.78E-05	2.0128
ENSG00000106336	FBXO24	1.1667	1.42E-05	3.78E-05	2.0126
ENSG00000070031	SCT	1.1354	1.42E-05	3.79E-05	2.0100
ENSG00000172340	SUCLG2	0.8377	1.42E-05	3.79E-05	2.0096
ENSG00000146918	NCAPG2	1.3650	1.42E-05	3.80E-05	2.0070
ENSG00000175820	CCDC168	1.0177	1.42E-05	3.80E-05	2.0068
ENSG00000258873	DUXA	1.1295	1.43E-05	3.81E-05	2.0035
ENSG00000176597	B3GNT5	1.2974	1.43E-05	3.82E-05	2.0021
ENSG00000198301	SDAD1	1.1770	1.43E-05	3.82E-05	2.0008
ENSG00000135052	GOLM1	1.3822	1.44E-05	3.83E-05	1.9971
ENSG00000159231	CBR3	1.1621	1.45E-05	3.86E-05	1.9912
ENSG00000160838	LRRC71	1.1028	1.45E-05	3.86E-05	1.9898
ENSG00000150394	CDH8	1.2740	1.45E-05	3.87E-05	1.9881
ENSG00000085982	USP40	1.2801	1.46E-05	3.88E-05	1.9842
ENSG00000164746	C7orf57	1.0726	1.46E-05	3.89E-05	1.9835

Gene ID	Gene Symbol	FC	P.Value	adj.P.Val	B
ENSG00000185250	PPIL6	1.1289	1.46E-05	3.89E-05	1.9827
ENSG00000135245	HILPDA	1.2251	1.47E-05	3.90E-05	1.9793
ENSG00000137055	PLAA	1.1701	1.47E-05	3.91E-05	1.9785
ENSG00000235272	RAMACL	1.3916	1.47E-05	3.92E-05	1.9750
ENSG00000180979	LRRC57	1.2042	1.48E-05	3.93E-05	1.9717
ENSG00000161016	RPL8	0.8534	1.48E-05	3.93E-05	1.9715
ENSG00000204657	OR2H2	1.0288	1.48E-05	3.93E-05	1.9709
ENSG00000248099	INSL3	1.2380	1.48E-05	3.95E-05	1.9676
ENSG00000161800	RACGAP1	1.3699	1.49E-05	3.95E-05	1.9672
ENSG00000096696	DSP	1.1670	1.49E-05	3.97E-05	1.9612
ENSG00000122034	GTF3A	0.8491	1.50E-05	3.98E-05	1.9597
ENSG00000197019	SERTAD1	1.2473	1.50E-05	3.98E-05	1.9595
ENSG00000184787	UBE2G2	1.2184	1.50E-05	3.99E-05	1.9567
ENSG00000106018	VIPR2	0.6193	1.51E-05	4.00E-05	1.9543
ENSG00000180953	ST20	0.8000	1.51E-05	4.00E-05	1.9531
ENSG00000146833	TRIM4	0.8232	1.51E-05	4.01E-05	1.9509
ENSG00000206150	RNASE13	1.0188	1.52E-05	4.03E-05	1.9473
ENSG00000181856	SLC2A4	1.0591	1.52E-05	4.03E-05	1.9468
ENSG00000138336	TET1	1.2077	1.52E-05	4.03E-05	1.9460
ENSG00000229676	ZNF492	1.0442	1.52E-05	4.04E-05	1.9432
ENSG00000099953	MMP11	0.7914	1.52E-05	4.04E-05	1.9430
ENSG00000182179	UBA7	0.7377	1.52E-05	4.04E-05	1.9428
ENSG00000122085	MTERF4	1.1713	1.53E-05	4.07E-05	1.9366
ENSG00000161277	THAP8	0.7826	1.53E-05	4.07E-05	1.9366
ENSG00000164114	MAP9	1.4207	1.54E-05	4.07E-05	1.9352
ENSG00000215193	PEX26	1.1912	1.54E-05	4.07E-05	1.9349
ENSG00000131503	ANKHD1	1.1662	1.54E-05	4.08E-05	1.9331
ENSG00000160229	ZNF66	1.2126	1.54E-05	4.09E-05	1.9303
ENSG00000185130	H2BC13	1.2065	1.55E-05	4.10E-05	1.9291
ENSG00000177301	KCNA2	0.7262	1.55E-05	4.11E-05	1.9264
ENSG00000171204	TMEM126B	0.8382	1.55E-05	4.11E-05	1.9250
ENSG00000111540	RAB5B	1.2106	1.56E-05	4.12E-05	1.9226
ENSG00000164418	GRIK2	1.1278	1.56E-05	4.14E-05	1.9192
ENSG00000108592	FTSJ3	1.2152	1.57E-05	4.15E-05	1.9157
ENSG00000204116	CHIC1	1.2167	1.57E-05	4.15E-05	1.9158
ENSG00000232070	TMEM253	1.2307	1.57E-05	4.16E-05	1.9147
ENSG00000052344	PRSS8	1.1854	1.57E-05	4.17E-05	1.9121
ENSG00000188322	SBK1	1.0723	1.58E-05	4.18E-05	1.9079
ENSG00000272573	MUSTN1	1.1729	1.58E-05	4.19E-05	1.9059
ENSG00000170890	PLA2G1B	1.1428	1.59E-05	4.20E-05	1.9047
ENSG00000122852	SFTPA1	1.0480	1.59E-05	4.20E-05	1.9032
ENSG00000178605	GTPBP6	1.2041	1.59E-05	4.21E-05	1.9018
ENSG00000111358	GTF2H3	1.2253	1.60E-05	4.22E-05	1.8989
ENSG00000180509	KCNE1	1.1339	1.60E-05	4.23E-05	1.8975
ENSG00000183396	TMEM89	1.0545	1.60E-05	4.23E-05	1.8966
ENSG00000130244	FAM98C	0.8387	1.60E-05	4.23E-05	1.8960
ENSG00000184613	NELL2	1.3010	1.60E-05	4.24E-05	1.8936
ENSG00000155511	GRIA1	1.1730	1.61E-05	4.25E-05	1.8907
ENSG00000064999	ANKS1A	1.1595	1.61E-05	4.27E-05	1.8878
ENSG00000130035	GALNT8	1.1306	1.62E-05	4.27E-05	1.8862
ENSG00000254827	SLC22A18AS	1.0950	1.62E-05	4.27E-05	1.8860
ENSG00000198015	MRPL42	0.7954	1.62E-05	4.28E-05	1.8841
ENSG00000126243	LRFN3	0.7771	1.63E-05	4.30E-05	1.8807
ENSG00000100532	CGRRF1	1.1896	1.63E-05	4.31E-05	1.8778

Gene ID	Gene Symbol	FC	P.Value	adj.P.Val	B
ENSG00000175318	GRAMD2A	1.0970	1.63E-05	4.31E-05	1.8765
ENSG00000089250	NOS1	1.0438	1.64E-05	4.33E-05	1.8737
ENSG00000166199	ALKBH3	0.8284	1.64E-05	4.33E-05	1.8728
ENSG00000198911	SREBF2	1.2230	1.64E-05	4.34E-05	1.8710
ENSG00000157895	C12orf43	1.1317	1.65E-05	4.35E-05	1.8676
ENSG00000177138	FAM9B	1.1270	1.65E-05	4.36E-05	1.8669
ENSG00000117245	KIF17	1.1325	1.65E-05	4.36E-05	1.8658
ENSG00000105048	TNNT1	1.2052	1.65E-05	4.36E-05	1.8648
ENSG00000144031	ANKRD53	1.4860	1.66E-05	4.37E-05	1.8627
ENSG00000250641	LY6G6F-LY6G6D	1.0415	1.67E-05	4.41E-05	1.8555
ENSG00000138347	MYPN	1.0401	1.67E-05	4.41E-05	1.8548
ENSG00000179407	DNAJB8	1.0155	1.68E-05	4.43E-05	1.8501
ENSG00000197114	ZGPAT	1.1729	1.68E-05	4.44E-05	1.8473
ENSG00000115993	TRAK2	1.1746	1.69E-05	4.45E-05	1.8455
ENSG00000132205	EMILIN2	1.2422	1.69E-05	4.46E-05	1.8437
ENSG00000110274	CEP164	1.2215	1.70E-05	4.47E-05	1.8402
ENSG00000106976	DNM1	1.1405	1.70E-05	4.49E-05	1.8363
ENSG00000186838	SELENOV	1.0237	1.71E-05	4.51E-05	1.8317
ENSG00000071282	LMCD1	1.2242	1.71E-05	4.51E-05	1.8314
ENSG00000196754	S100A2	1.2222	1.71E-05	4.51E-05	1.8310
ENSG00000171903	CYP4F11	1.2289	1.72E-05	4.52E-05	1.8291
ENSG00000163817	SLC6A20	1.1469	1.73E-05	4.54E-05	1.8243
ENSG00000133863	TEX15	1.0765	1.73E-05	4.55E-05	1.8231
ENSG00000004779	NDUFAB1	0.8488	1.73E-05	4.56E-05	1.8214
ENSG00000129038	LOXL1	1.1277	1.73E-05	4.56E-05	1.8198
ENSG00000164007	CLDN19	1.0143	1.74E-05	4.57E-05	1.8189
ENSG00000171793	CTPS1	1.2387	1.74E-05	4.57E-05	1.8173
ENSG00000183134	PTGDR2	1.1181	1.75E-05	4.60E-05	1.8128
ENSG00000165061	ZMAT4	1.0957	1.75E-05	4.60E-05	1.8121
ENSG00000197405	C5AR1	1.4141	1.75E-05	4.60E-05	1.8110
ENSG00000108389	MTMR4	1.2951	1.75E-05	4.61E-05	1.8094
ENSG00000170004	CHD3	1.4776	1.75E-05	4.61E-05	1.8094
ENSG00000126787	DLGAP5	1.3551	1.76E-05	4.62E-05	1.8071
ENSG00000151835	SACS	1.1712	1.76E-05	4.62E-05	1.8063
ENSG00000134590	RTL8C	1.3606	1.76E-05	4.63E-05	1.8040
ENSG00000064199	SPA17	1.2301	1.77E-05	4.66E-05	1.7991
ENSG00000132185	FCRLA	1.7326	1.78E-05	4.67E-05	1.7959
ENSG00000173227	SYT12	1.0933	1.78E-05	4.69E-05	1.7926
ENSG00000215113	CXorf49B	1.1598	1.78E-05	4.69E-05	1.7925
ENSG00000105281	SLC1A5	0.7677	1.80E-05	4.72E-05	1.7852
ENSG00000198467	TPM2	1.4916	1.80E-05	4.74E-05	1.7822
ENSG00000157782	CABP1	1.0822	1.80E-05	4.74E-05	1.7818
ENSG00000162616	DNAJB4	1.3590	1.81E-05	4.74E-05	1.7807
ENSG00000177462	OR2T8	1.0415	1.81E-05	4.76E-05	1.7770
ENSG00000090857	PDPR	1.2662	1.81E-05	4.76E-05	1.7767
ENSG00000128944	KNSTRN	0.7859	1.81E-05	4.76E-05	1.7764
ENSG00000120253	NUP43	0.7704	1.82E-05	4.78E-05	1.7736
ENSG00000132781	MUTYH	1.2261	1.82E-05	4.79E-05	1.7710
ENSG00000083290	ULK2	1.2285	1.83E-05	4.80E-05	1.7677
ENSG00000125787	GNRH2	1.1008	1.83E-05	4.80E-05	1.7679
ENSG00000162039	MEIOB	1.0777	1.83E-05	4.81E-05	1.7668
ENSG00000078043	PIAS2	1.2447	1.84E-05	4.82E-05	1.7646
ENSG00000020577	SAMD4A	1.2486	1.85E-05	4.84E-05	1.7596
ENSG00000084110	HAL	1.2197	1.85E-05	4.85E-05	1.7570

Gene ID	Gene Symbol	FC	P.Value	adj.P.Val	B
ENSG00000119723	COQ6	1.1861	1.86E-05	4.87E-05	1.7530
ENSG00000106692	FKTN	0.7444	1.86E-05	4.88E-05	1.7525
ENSG00000164659	ELAPOR2	1.5975	1.87E-05	4.90E-05	1.7484
ENSG00000174021	GNG5	1.1746	1.87E-05	4.90E-05	1.7470
ENSG00000240654	C1QTNF9	1.0541	1.88E-05	4.91E-05	1.7451
ENSG00000167371	PRRT2	1.1838	1.88E-05	4.92E-05	1.7444
ENSG00000142168	SOD1	1.1692	1.90E-05	4.97E-05	1.7334
ENSG00000089692	LAG3	0.5295	1.90E-05	4.98E-05	1.7321
ENSG00000100170	SLC5A1	1.0286	1.90E-05	4.98E-05	1.7313
ENSG00000112578	BYSL	0.7563	1.90E-05	4.98E-05	1.7311
ENSG00000163320	CGGBP1	1.1749	1.90E-05	4.98E-05	1.7308
ENSG00000102096	PIM2	1.3559	1.91E-05	4.99E-05	1.7290
ENSG00000173402	DAG1	1.1926	1.91E-05	4.99E-05	1.7286
ENSG00000183248	PRR36	1.1058	1.91E-05	5.01E-05	1.7261
ENSG00000121690	DEPDC7	1.0790	1.92E-05	5.03E-05	1.7205
ENSG00000136518	ACTL6A	0.7992	1.93E-05	5.05E-05	1.7176
ENSG00000104365	IKBKB	1.2131	1.95E-05	5.09E-05	1.7100
ENSG00000122435	TRMT13	0.7511	1.95E-05	5.10E-05	1.7073
ENSG00000132938	MTUS2	1.1361	1.96E-05	5.12E-05	1.7048
ENSG00000143952	VPS54	1.1359	1.96E-05	5.12E-05	1.7038
ENSG00000106804	C5	1.2336	1.97E-05	5.14E-05	1.6991
ENSG00000145826	LECT2	1.1324	1.97E-05	5.14E-05	1.6991
ENSG00000102468	HTR2A	1.0160	1.98E-05	5.17E-05	1.6940
ENSG00000125648	SLC25A23	0.7856	1.98E-05	5.17E-05	1.6931
ENSG00000131089	ARHGEF9	1.2275	2.00E-05	5.23E-05	1.6836
ENSG00000166589	CDH16	1.0278	2.01E-05	5.25E-05	1.6786
ENSG00000121769	FABP3	0.6601	2.01E-05	5.26E-05	1.6775
ENSG00000113638	TTC33	0.7935	2.03E-05	5.30E-05	1.6701
ENSG00000114349	GNAT1	1.0518	2.04E-05	5.33E-05	1.6650
ENSG00000178562	CD28	1.7664	2.05E-05	5.34E-05	1.6621
ENSG00000035862	TIMP2	1.5116	2.06E-05	5.39E-05	1.6533
ENSG00000111801	BTN3A3	0.7376	2.07E-05	5.40E-05	1.6509
ENSG00000141384	TAF4B	1.2554	2.07E-05	5.40E-05	1.6505
ENSG00000013563	DNASE1L1	1.2325	2.07E-05	5.41E-05	1.6494
ENSG00000177150	FAM210A	1.2062	2.07E-05	5.41E-05	1.6488
ENSG00000120738	EGR1	0.5682	2.08E-05	5.44E-05	1.6445
ENSG00000198862	LTN1	1.1948	2.09E-05	5.44E-05	1.6432
ENSG00000143590	EFNA3	1.1830	2.09E-05	5.45E-05	1.6418
ENSG00000052841	TTC17	1.1549	2.09E-05	5.46E-05	1.6405
ENSG00000183826	BTBD9	1.2140	2.10E-05	5.46E-05	1.6392
ENSG00000140403	DNAJA4	1.3306	2.10E-05	5.47E-05	1.6388
ENSG00000170456	DENND5B	1.2462	2.12E-05	5.53E-05	1.6268
ENSG00000206140	TMEM191C	1.1861	2.12E-05	5.54E-05	1.6261
ENSG00000198178	CLEC4C	1.0510	2.13E-05	5.55E-05	1.6243
ENSG00000166902	MRPL16	0.8383	2.13E-05	5.56E-05	1.6216
ENSG00000118096	IFT46	0.8233	2.14E-05	5.56E-05	1.6212
ENSG00000179636	TPPP2	1.0460	2.15E-05	5.61E-05	1.6130
ENSG00000128626	MRPS12	0.8565	2.18E-05	5.67E-05	1.6025
ENSG00000104901	DKKL1	1.0338	2.18E-05	5.67E-05	1.6019
ENSG00000204920	ZNF155	1.2877	2.18E-05	5.68E-05	1.6012
ENSG00000122484	RPAP2	1.1708	2.19E-05	5.69E-05	1.5988
ENSG00000090932	DLL3	1.1029	2.19E-05	5.71E-05	1.5953
ENSG00000090006	LTBP4	1.2608	2.20E-05	5.72E-05	1.5934
ENSG00000159433	STARD9	1.2629	2.21E-05	5.74E-05	1.5898

Gene ID	Gene Symbol	FC	P.Value	adj.P.Val	B
ENSG00000186716	BCR	1.2292	2.21E-05	5.74E-05	1.5897
ENSG00000066923	STAG3	1.2776	2.22E-05	5.77E-05	1.5848
ENSG00000091181	IL5RA	0.5194	2.22E-05	5.78E-05	1.5838
ENSG00000182111	ZNF716	1.0830	2.22E-05	5.78E-05	1.5837
ENSG00000119650	IFT43	1.1830	2.23E-05	5.79E-05	1.5817
ENSG00000172348	RCAN2	1.1859	2.23E-05	5.80E-05	1.5797
ENSG00000144711	IQSEC1	1.2561	2.23E-05	5.81E-05	1.5780
ENSG00000011295	TTC19	1.1489	2.24E-05	5.82E-05	1.5763
ENSG00000167483	NIBAN3	1.1574	2.24E-05	5.82E-05	1.5760
ENSG00000087502	ERGIC2	0.7891	2.25E-05	5.84E-05	1.5725
ENSG00000148308	GTF3C5	1.1759	2.25E-05	5.84E-05	1.5721
ENSG00000154511	DIPK1A	0.7078	2.25E-05	5.85E-05	1.5715
ENSG00000164266	SPINK1	1.1324	2.25E-05	5.86E-05	1.5698
ENSG00000143740	SNAP47	1.2491	2.26E-05	5.88E-05	1.5656
ENSG00000074621	SLC24A1	1.1913	2.27E-05	5.88E-05	1.5651
ENSG00000164591	MYOZ3	1.0334	2.27E-05	5.89E-05	1.5639
ENSG00000131848	ZSCAN5A	0.8438	2.27E-05	5.89E-05	1.5630
ENSG00000134077	THUMPD3	1.1583	2.27E-05	5.89E-05	1.5629
ENSG00000171444	MCC	0.7458	2.28E-05	5.91E-05	1.5601
ENSG00000139428	MMAB	1.1798	2.28E-05	5.92E-05	1.5589
ENSG00000146966	DENND2A	1.1027	2.28E-05	5.92E-05	1.5588
ENSG00000154889	MPPE1	1.1903	2.28E-05	5.92E-05	1.5585
ENSG00000171135	JAGN1	0.8673	2.28E-05	5.92E-05	1.5585
ENSG00000151012	SLC7A11	1.3255	2.28E-05	5.92E-05	1.5580
ENSG00000163328	GPR155	1.3042	2.28E-05	5.92E-05	1.5573
ENSG00000169064	ZBBX	1.0827	2.29E-05	5.94E-05	1.5545
ENSG00000181722	ZBTB20	1.2628	2.29E-05	5.94E-05	1.5541
ENSG00000086717	PPEF1	1.1080	2.30E-05	5.96E-05	1.5513
ENSG00000196876	SCN8A	1.2080	2.30E-05	5.97E-05	1.5494
ENSG00000165188	RNF183	1.1385	2.30E-05	5.97E-05	1.5489
ENSG00000170915	PAQR8	1.1792	2.31E-05	5.97E-05	1.5482
ENSG00000171552	BCL2L1	0.7467	2.32E-05	6.01E-05	1.5424
ENSG00000123689	G0S2	2.0190	2.32E-05	6.02E-05	1.5409
ENSG00000176749	CDK5R1	1.1232	2.33E-05	6.04E-05	1.5382
ENSG00000101040	ZMYND8	1.2628	2.35E-05	6.08E-05	1.5309
ENSG00000099992	TBC1D10A	1.1960	2.36E-05	6.10E-05	1.5276
ENSG00000142182	DNMT3L	1.1031	2.37E-05	6.14E-05	1.5222
ENSG00000184232	OAF	0.7388	2.37E-05	6.15E-05	1.5202
ENSG00000133069	TMCC2	1.4099	2.38E-05	6.16E-05	1.5185
ENSG00000119715	ESRRB	1.1535	2.38E-05	6.16E-05	1.5173
ENSG00000154122	ANKH	1.3450	2.39E-05	6.17E-05	1.5155
ENSG00000080854	IGSF9B	1.1149	2.39E-05	6.19E-05	1.5133
ENSG00000116138	DNAJC16	1.2178	2.39E-05	6.19E-05	1.5132
ENSG00000144410	CPO	0.9199	2.40E-05	6.21E-05	1.5099
ENSG00000204370	SDHD	0.8562	2.41E-05	6.22E-05	1.5073
ENSG00000165966	PDZRN4	0.6764	2.42E-05	6.26E-05	1.5018
ENSG00000165630	PRPF18	1.1586	2.42E-05	6.27E-05	1.5004
ENSG00000162430	SELENON	1.2825	2.43E-05	6.27E-05	1.4996
ENSG00000155876	RRAGA	0.8333	2.43E-05	6.28E-05	1.4991
ENSG00000157353	FCSK	1.2311	2.44E-05	6.30E-05	1.4947
ENSG00000178826	TMEM139	1.0606	2.44E-05	6.31E-05	1.4940
ENSG00000109738	GLRB	1.1245	2.45E-05	6.32E-05	1.4923
ENSG00000148334	PTGES2	0.8473	2.45E-05	6.32E-05	1.4919
ENSG00000173627	APOBEC4	1.0547	2.45E-05	6.32E-05	1.4919

Gene ID	Gene Symbol	FC	P.Value	adj.P.Val	B
ENSG00000153993	SEMA3D	1.2820	2.47E-05	6.38E-05	1.4819
ENSG00000197150	ABCB8	1.2353	2.47E-05	6.39E-05	1.4813
ENSG00000197520	FAM177B	1.2026	2.48E-05	6.40E-05	1.4794
ENSG00000008382	MPND	0.8401	2.48E-05	6.41E-05	1.4772
ENSG00000174227	PIGG	1.2157	2.49E-05	6.43E-05	1.4751
ENSG00000162458	FBLIM1	1.0938	2.50E-05	6.45E-05	1.4720
ENSG00000146007	ZMAT2	0.8534	2.50E-05	6.45E-05	1.4713
ENSG00000068078	FGFR3	2.5672	2.50E-05	6.46E-05	1.4703
ENSG00000174628	IQCK	1.1627	2.54E-05	6.56E-05	1.4546
ENSG00000014138	POLA2	1.2539	2.55E-05	6.58E-05	1.4527
ENSG00000121940	CLCC1	0.8162	2.55E-05	6.58E-05	1.4513
ENSG00000036530	CYP46A1	1.1965	2.56E-05	6.59E-05	1.4502
ENSG00000163705	FANCD2OS	1.1230	2.56E-05	6.61E-05	1.4470
ENSG00000139190	VAMP1	1.3628	2.57E-05	6.61E-05	1.4466
ENSG00000196981	WDR5B	0.7971	2.57E-05	6.61E-05	1.4465
ENSG00000108021	TASOR2	1.2187	2.58E-05	6.65E-05	1.4411
ENSG00000109911	ELP4	0.8103	2.58E-05	6.65E-05	1.4412
ENSG00000067225	PKM	1.2180	2.58E-05	6.65E-05	1.4407
ENSG00000197713	RPE	0.7946	2.59E-05	6.66E-05	1.4392
ENSG00000148090	AUH	0.8464	2.59E-05	6.66E-05	1.4387
ENSG00000164109	MAD2L1	1.3540	2.59E-05	6.66E-05	1.4386
ENSG00000214534	ZNF705E	1.0631	2.61E-05	6.71E-05	1.4316
ENSG00000009307	CSDE1	1.1868	2.62E-05	6.76E-05	1.4251
ENSG00000168062	BATF2	0.8095	2.63E-05	6.78E-05	1.4216
ENSG00000132906	CASP9	1.1730	2.64E-05	6.79E-05	1.4208
ENSG00000186103	ARGFX	1.0729	2.64E-05	6.79E-05	1.4206
ENSG00000112309	B3GAT2	1.1015	2.64E-05	6.80E-05	1.4187
ENSG00000204977	TRIM13	1.1865	2.65E-05	6.82E-05	1.4163
ENSG00000116675	DNAJC6	1.2549	2.65E-05	6.82E-05	1.4161
ENSG00000150967	ABCB9	1.4255	2.66E-05	6.83E-05	1.4133
ENSG00000124140	SLC12A5	1.2262	2.67E-05	6.86E-05	1.4092
ENSG00000179958	DCTPP1	0.8013	2.68E-05	6.88E-05	1.4069
ENSG00000101608	MYL12A	1.2270	2.68E-05	6.88E-05	1.4063
ENSG00000163812	ZDHHC3	1.1299	2.68E-05	6.88E-05	1.4061
ENSG00000165895	ARHGAP42	1.3366	2.68E-05	6.88E-05	1.4059
ENSG00000112144	CILK1	1.2019	2.68E-05	6.89E-05	1.4045
ENSG00000131831	RAI2	1.1917	2.69E-05	6.92E-05	1.4011
ENSG00000122735	DNAI1	1.0710	2.70E-05	6.94E-05	1.3978
ENSG00000147454	SLC25A37	1.3536	2.71E-05	6.96E-05	1.3946
ENSG00000178623	GPR35	1.1944	2.71E-05	6.96E-05	1.3946
ENSG00000196123	KIAA0895L	1.2992	2.71E-05	6.96E-05	1.3946
ENSG00000204923	FBXO48	1.1588	2.71E-05	6.97E-05	1.3929
ENSG00000107186	MPDZ	1.1629	2.73E-05	7.00E-05	1.3887
ENSG00000185112	FAM43A	1.3472	2.74E-05	7.03E-05	1.3850
ENSG00000104695	PPP2CB	1.1658	2.74E-05	7.03E-05	1.3840
ENSG00000171659	GPR34	0.8106	2.75E-05	7.07E-05	1.3793
ENSG00000007372	PAX6	0.8248	2.75E-05	7.07E-05	1.3791
ENSG00000169087	HSPBAP1	1.1631	2.76E-05	7.08E-05	1.3769
ENSG00000145020	AMT	1.3130	2.77E-05	7.11E-05	1.3737
ENSG00000132846	ZBED3	0.7878	2.77E-05	7.11E-05	1.3725
ENSG00000176248	ANAPC2	1.1792	2.78E-05	7.14E-05	1.3697
ENSG00000149201	CCDC81	1.0970	2.79E-05	7.16E-05	1.3661
ENSG00000163352	LENEP	1.0593	2.79E-05	7.16E-05	1.3658
ENSG00000163608	NEPRO	1.1612	2.79E-05	7.16E-05	1.3654

Gene ID	Gene Symbol	FC	P.Value	adj.P.Val	B
ENSG00000187690	EZHIP	1.0559	2.81E-05	7.19E-05	1.3616
ENSG00000067533	RRP15	1.2556	2.81E-05	7.20E-05	1.3599
ENSG00000188549	CCDC9B	0.8382	2.82E-05	7.22E-05	1.3575
ENSG00000081923	ATP8B1	1.1013	2.83E-05	7.24E-05	1.3544
ENSG00000114638	UPK1B	1.0327	2.86E-05	7.33E-05	1.3424
ENSG00000167113	COQ4	0.8587	2.86E-05	7.34E-05	1.3418
ENSG00000151665	PIGF	0.8241	2.87E-05	7.35E-05	1.3403
ENSG00000171492	LRRC8D	0.8236	2.89E-05	7.40E-05	1.3342
ENSG00000162302	RPS6KA4	1.1855	2.89E-05	7.41E-05	1.3319
ENSG00000032742	IFT88	1.2001	2.90E-05	7.44E-05	1.3287
ENSG00000270800	RPS10-NUDT3	1.2083	2.92E-05	7.46E-05	1.3249
ENSG00000116981	NT5C1A	1.0390	2.92E-05	7.47E-05	1.3245
ENSG00000152749	GPR180	0.8179	2.92E-05	7.47E-05	1.3237
ENSG00000122779	TRIM24	1.2004	2.93E-05	7.51E-05	1.3194
ENSG00000101265	RASSF2	1.3647	2.94E-05	7.52E-05	1.3176
ENSG00000169507	SLC38A11	1.0806	2.95E-05	7.55E-05	1.3136
ENSG00000078487	ZCWPW1	1.1981	2.95E-05	7.55E-05	1.3131
ENSG00000135919	SERPINE2	1.8180	2.97E-05	7.60E-05	1.3068
ENSG00000042980	ADAM28	1.8375	2.98E-05	7.62E-05	1.3043
ENSG00000102879	CORO1A	1.4130	2.99E-05	7.63E-05	1.3025
ENSG00000163638	ADAMTS9	1.1564	2.99E-05	7.65E-05	1.3006
ENSG00000103091	WDR59	1.2024	2.99E-05	7.65E-05	1.3002
ENSG00000196329	GIMAP5	0.7481	3.00E-05	7.66E-05	1.2990
ENSG00000188522	FAM83G	1.1868	3.00E-05	7.67E-05	1.2973
ENSG00000101883	RHOXF1	1.1839	3.00E-05	7.68E-05	1.2965
ENSG00000168928	CTRB2	1.0801	3.01E-05	7.69E-05	1.2947
ENSG00000171388	APLN	1.0749	3.04E-05	7.78E-05	1.2840
ENSG00000121691	CAT	0.7865	3.05E-05	7.79E-05	1.2822
ENSG00000173567	ADGRF3	1.1730	3.05E-05	7.80E-05	1.2812
ENSG00000188706	ZDHHC9	1.2042	3.06E-05	7.82E-05	1.2781
ENSG00000205307	SAP25	1.2657	3.07E-05	7.83E-05	1.2772
ENSG00000008441	NFIX	0.8084	3.07E-05	7.83E-05	1.2763
ENSG00000167646	DNAAF3	1.0746	3.08E-05	7.85E-05	1.2744
ENSG00000160221	GATD3A	0.8343	3.09E-05	7.88E-05	1.2708
ENSG00000131323	TRAF3	1.3112	3.09E-05	7.89E-05	1.2697
ENSG00000171490	RSL1D1	0.8397	3.10E-05	7.91E-05	1.2672
ENSG00000119801	YPEL5	1.3136	3.10E-05	7.91E-05	1.2670
ENSG00000147394	ZNF185	0.7194	3.10E-05	7.91E-05	1.2665
ENSG00000185379	RAD51D	1.2289	3.10E-05	7.92E-05	1.2654
ENSG00000076053	RBM7	1.1970	3.12E-05	7.96E-05	1.2606
ENSG00000144837	PLA1A	1.0873	3.12E-05	7.97E-05	1.2592
ENSG00000164093	PITX2	1.0716	3.13E-05	7.97E-05	1.2583
ENSG00000185274	GALNT17	1.2238	3.15E-05	8.02E-05	1.2522
ENSG00000015520	NPC1L1	1.0472	3.15E-05	8.03E-05	1.2509
ENSG00000107175	CREB3	0.8590	3.16E-05	8.04E-05	1.2497
ENSG00000013306	SLC25A39	1.1581	3.16E-05	8.05E-05	1.2486
ENSG00000138686	BBS7	1.2267	3.16E-05	8.05E-05	1.2485
ENSG00000135437	RDH5	1.2433	3.17E-05	8.07E-05	1.2461
ENSG00000005249	PRKAR2B	1.3132	3.17E-05	8.07E-05	1.2459
ENSG00000086102	NFX1	1.1707	3.17E-05	8.08E-05	1.2444
ENSG00000111364	DDX55	1.2021	3.17E-05	8.08E-05	1.2443
ENSG00000176697	BDNF	1.1380	3.18E-05	8.09E-05	1.2428
ENSG00000141012	GALNS	1.2387	3.19E-05	8.11E-05	1.2408
ENSG00000095261	PSMD5	1.1812	3.19E-05	8.11E-05	1.2406

Gene ID	Gene Symbol	FC	P.Value	adj.P.Val	B
ENSG00000142252	GEMIN7	0.8499	3.19E-05	8.11E-05	1.2405
ENSG00000181215	C4orf50	0.8573	3.19E-05	8.12E-05	1.2391
ENSG00000237489	C10orf143	0.9069	3.21E-05	8.17E-05	1.2334
ENSG00000168546	GFRA2	0.7482	3.23E-05	8.22E-05	1.2271
ENSG00000219200	RNASEK	1.1922	3.24E-05	8.23E-05	1.2261
ENSG00000114786	ABHD14A-ACY1	0.8584	3.24E-05	8.24E-05	1.2247
ENSG00000115602	IL1RL1	1.1461	3.25E-05	8.25E-05	1.2228
ENSG00000135722	FBXL8	1.2447	3.25E-05	8.25E-05	1.2228
ENSG00000125257	ABCC4	0.7419	3.25E-05	8.26E-05	1.2222
ENSG00000088053	GP6	1.1428	3.26E-05	8.27E-05	1.2203
ENSG00000105928	GSDME	1.1673	3.28E-05	8.33E-05	1.2143
ENSG00000005421	PON1	1.0578	3.29E-05	8.37E-05	1.2093
ENSG00000136213	CHST12	0.7595	3.31E-05	8.40E-05	1.2052
ENSG00000196586	MYO6	1.3516	3.32E-05	8.43E-05	1.2015
ENSG00000102524	TNFSF13B	0.8034	3.33E-05	8.46E-05	1.1981
ENSG00000179477	ALOX12B	1.0557	3.36E-05	8.52E-05	1.1912
ENSG00000197457	STMN3	1.3248	3.36E-05	8.52E-05	1.1912
ENSG00000109072	VTN	1.0964	3.36E-05	8.53E-05	1.1903
ENSG00000125510	OPRL1	0.8631	3.37E-05	8.55E-05	1.1882
ENSG00000069535	MAOB	1.0645	3.38E-05	8.59E-05	1.1834
ENSG00000182983	ZNF662	1.2942	3.39E-05	8.61E-05	1.1806
ENSG00000178935	ZNF552	1.1783	3.41E-05	8.65E-05	1.1764
ENSG00000146374	RSPO3	0.7855	3.41E-05	8.65E-05	1.1761
ENSG00000146674	IGFBP3	1.4330	3.41E-05	8.65E-05	1.1759
ENSG00000164051	CCDC51	0.7885	3.41E-05	8.66E-05	1.1755
ENSG00000172456	FGGY	1.2264	3.42E-05	8.68E-05	1.1727
ENSG00000152315	KCNK13	1.2728	3.43E-05	8.70E-05	1.1701
ENSG00000103313	MEFV	1.1535	3.46E-05	8.77E-05	1.1622
ENSG00000149218	ENDOD1	1.2641	3.47E-05	8.80E-05	1.1591
ENSG00000166477	LEO1	0.8173	3.48E-05	8.82E-05	1.1571
ENSG00000130311	DDA1	1.1345	3.48E-05	8.82E-05	1.1567
ENSG00000204511	MCCD1	1.0303	3.50E-05	8.87E-05	1.1516
ENSG00000260027	HOXB7	0.7306	3.51E-05	8.89E-05	1.1495
ENSG00000167792	NDUFV1	1.1536	3.53E-05	8.95E-05	1.1425
ENSG00000269343	ZNF587B	1.2095	3.53E-05	8.95E-05	1.1426
ENSG00000113838	TBCCD1	0.8220	3.54E-05	8.95E-05	1.1420
ENSG00000105755	ETHE1	0.8557	3.54E-05	8.97E-05	1.1399
ENSG00000118689	FOXO3	1.2606	3.55E-05	9.00E-05	1.1370
ENSG00000099999	RNF215	1.2044	3.56E-05	9.00E-05	1.1364
ENSG00000137200	CMTR1	1.1819	3.56E-05	9.00E-05	1.1361
ENSG00000173818	ENDOV	1.1798	3.56E-05	9.00E-05	1.1359
ENSG00000162062	TEDC2	1.3218	3.56E-05	9.01E-05	1.1347
ENSG00000162695	SLC30A7	1.1921	3.58E-05	9.06E-05	1.1304
ENSG00000197748	CFAP43	1.1150	3.59E-05	9.08E-05	1.1281
ENSG00000239732	TLR9	1.3452	3.60E-05	9.12E-05	1.1237
ENSG00000135346	CGA	1.1412	3.62E-05	9.15E-05	1.1202
ENSG00000105997	HOXA3	0.8236	3.62E-05	9.15E-05	1.1198
ENSG00000173372	C1QA	0.5249	3.64E-05	9.21E-05	1.1132
ENSG00000145526	CDH18	1.2642	3.65E-05	9.22E-05	1.1120
ENSG00000120159	CAAP1	1.1554	3.66E-05	9.24E-05	1.1102
ENSG00000179348	GATA2	1.0908	3.66E-05	9.24E-05	1.1098
ENSG00000005007	UPF1	1.2270	3.66E-05	9.26E-05	1.1079
ENSG00000196189	SEMA4A	1.5522	3.67E-05	9.26E-05	1.1074
ENSG00000105643	ARRDC2	1.4321	3.67E-05	9.27E-05	1.1065

Gene ID	Gene Symbol	FC	P.Value	adj.P.Val	B
ENSG00000143543	JTB	1.1471	3.67E-05	9.27E-05	1.1066
ENSG00000187626	ZKSCAN4	0.8276	3.67E-05	9.28E-05	1.1052
ENSG00000107295	SH3GL2	1.0429	3.71E-05	9.37E-05	1.0956
ENSG00000178342	KCNG2	1.0292	3.71E-05	9.38E-05	1.0951
ENSG00000189337	KAZN	1.1229	3.71E-05	9.38E-05	1.0949
ENSG00000183578	TNFAIP8L3	1.0423	3.72E-05	9.40E-05	1.0924
ENSG00000163071	SPATA18	1.1087	3.74E-05	9.43E-05	1.0893
ENSG00000143520	FLG2	1.0108	3.75E-05	9.46E-05	1.0864
ENSG00000114503	NCBP2	0.8505	3.75E-05	9.47E-05	1.0857
ENSG00000137822	TUBGCP4	1.1711	3.77E-05	9.50E-05	1.0818
ENSG00000128607	KLHDC10	1.1580	3.78E-05	9.54E-05	1.0777
ENSG00000105583	WDR83OS	0.8995	3.79E-05	9.56E-05	1.0756
ENSG00000197081	IGF2R	1.2355	3.80E-05	9.59E-05	1.0725
ENSG00000100403	ZC3H7B	1.1883	3.81E-05	9.61E-05	1.0712
ENSG00000165929	TC2N	1.1952	3.83E-05	9.65E-05	1.0666
ENSG00000168763	CNNM3	1.2266	3.84E-05	9.69E-05	1.0626
ENSG00000132313	MRPL35	0.7942	3.85E-05	9.70E-05	1.0613
ENSG00000178802	MPI	0.7971	3.85E-05	9.72E-05	1.0598
ENSG00000101353	MROH8	1.1989	3.86E-05	9.73E-05	1.0583
ENSG00000135503	ACVR1B	1.2250	3.86E-05	9.73E-05	1.0583
ENSG00000104983	CCDC61	0.8042	3.86E-05	9.73E-05	1.0576
ENSG00000006530	AGK	1.1876	3.87E-05	9.74E-05	1.0572
ENSG00000161813	LARP4	1.1715	3.89E-05	9.80E-05	1.0510
ENSG00000135111	TBX3	1.0888	3.89E-05	9.80E-05	1.0505
ENSG00000181350	LRRC75A	1.1650	3.91E-05	9.84E-05	1.0465
ENSG00000145692	BHMT	1.0674	3.92E-05	9.86E-05	1.0450
ENSG00000169251	NMD3	1.1397	3.92E-05	9.87E-05	1.0441
ENSG00000168658	VWA3B	1.0595	3.92E-05	9.87E-05	1.0439
ENSG00000115947	ORC4	1.1837	3.92E-05	9.87E-05	1.0435
ENSG00000126088	UROD	1.1605	3.93E-05	9.88E-05	1.0425
ENSG00000163832	ELP6	0.8569	3.93E-05	9.88E-05	1.0425
ENSG00000130254	SAFB2	1.1780	3.94E-05	9.92E-05	1.0379
ENSG00000039319	ZFYVE16	1.1925	3.95E-05	9.93E-05	1.0375
ENSG00000187045	TMPRSS6	1.0596	3.95E-05	9.94E-05	1.0357
ENSG00000060656	PTPRU	1.3096	3.98E-05	1.00E-04	1.0297
ENSG00000116141	MARK1	1.3452	3.98E-05	1.00E-04	1.0287
ENSG00000072506	HSD17B10	1.1789	3.99E-05	1.00E-04	1.0263
ENSG00000167985	SDHAF2	0.8568	4.00E-05	1.01E-04	1.0247
ENSG00000165312	OTUD1	1.4354	4.01E-05	1.01E-04	1.0223
ENSG00000104177	MYEF2	1.3460	4.03E-05	1.01E-04	1.0175
ENSG00000129566	TEP1	1.2114	4.03E-05	1.01E-04	1.0167
ENSG00000167384	ZNF180	1.2115	4.05E-05	1.02E-04	1.0127
ENSG00000175701	MTLN	0.7455	4.05E-05	1.02E-04	1.0128
ENSG00000259224	SLC35G6	1.0299	4.05E-05	1.02E-04	1.0129
ENSG00000100578	KIAA0586	1.2094	4.07E-05	1.02E-04	1.0080
ENSG00000187824	TMEM220	1.4294	4.08E-05	1.02E-04	1.0058
ENSG00000123685	BATF3	1.3306	4.09E-05	1.03E-04	1.0036
ENSG00000011105	TSPAN9	1.0897	4.10E-05	1.03E-04	1.0010
ENSG00000131080	EDA2R	0.7292	4.10E-05	1.03E-04	1.0005
ENSG00000187243	MAGED4B	1.6636	4.10E-05	1.03E-04	1.0003
ENSG00000103260	METRN	0.6471	4.11E-05	1.03E-04	0.9992
ENSG00000110108	TMEM109	1.1887	4.12E-05	1.03E-04	0.9974
ENSG00000033867	SLC4A7	1.3043	4.12E-05	1.03E-04	0.9969
ENSG00000164989	CCDC171	0.7067	4.12E-05	1.03E-04	0.9966

Gene ID	Gene Symbol	FC	P.Value	adj.P.Val	B
ENSG00000054983	GALC	1.1847	4.13E-05	1.04E-04	0.9948
ENSG00000165521	EML5	1.2231	4.13E-05	1.04E-04	0.9946
ENSG00000215717	TMEM167B	1.1468	4.13E-05	1.04E-04	0.9937
ENSG00000134809	TIMM10	0.8384	4.14E-05	1.04E-04	0.9909
ENSG00000123178	SPRYD7	0.8529	4.15E-05	1.04E-04	0.9897
ENSG00000121570	DPPA4	1.2560	4.16E-05	1.04E-04	0.9866
ENSG00000142186	SCYL1	1.1631	4.17E-05	1.04E-04	0.9852
ENSG00000177051	FBXO46	0.8018	4.17E-05	1.05E-04	0.9849
ENSG00000170485	NPAS2	1.0859	4.19E-05	1.05E-04	0.9797
ENSG00000168894	RNF181	0.8465	4.20E-05	1.05E-04	0.9776
ENSG00000130811	EIF3G	0.8842	4.21E-05	1.06E-04	0.9753
ENSG00000123080	CDKN2C	1.5493	4.22E-05	1.06E-04	0.9744
ENSG00000188153	COL4A5	0.5863	4.24E-05	1.06E-04	0.9699
ENSG00000048052	HDAC9	1.3474	4.25E-05	1.06E-04	0.9677
ENSG00000175387	SMAD2	1.1679	4.27E-05	1.07E-04	0.9634
ENSG00000136274	NACAD	1.0719	4.27E-05	1.07E-04	0.9629
ENSG00000011485	PPP5C	0.7929	4.27E-05	1.07E-04	0.9621
ENSG00000013810	TACC3	1.3169	4.28E-05	1.07E-04	0.9614
ENSG00000067182	TNFRSF1A	1.3426	4.28E-05	1.07E-04	0.9601
ENSG00000172209	GPR22	1.0497	4.29E-05	1.07E-04	0.9573
ENSG00000198961	PJA2	1.1660	4.30E-05	1.08E-04	0.9558
ENSG00000184434	LRRC19	1.0400	4.32E-05	1.08E-04	0.9521
ENSG00000203326	ZNF525	1.3181	4.32E-05	1.08E-04	0.9519
ENSG00000175699	CCDC197	1.0534	4.32E-05	1.08E-04	0.9506
ENSG00000104689	TNFRSF10A	1.2295	4.35E-05	1.09E-04	0.9447
ENSG00000147416	ATP6V1B2	1.1936	4.35E-05	1.09E-04	0.9447
ENSG00000149100	EIF3M	0.8561	4.36E-05	1.09E-04	0.9419
ENSG00000154646	TMPRSS15	1.1652	4.37E-05	1.09E-04	0.9413
ENSG00000153391	INO80C	1.1602	4.39E-05	1.10E-04	0.9373
ENSG00000159289	GOLGA6A	1.1589	4.39E-05	1.10E-04	0.9370
ENSG00000067167	TRAM1	1.1857	4.39E-05	1.10E-04	0.9367
ENSG00000176714	CCDC121	0.7995	4.40E-05	1.10E-04	0.9348
ENSG00000177879	AP3S1	1.2165	4.43E-05	1.11E-04	0.9283
ENSG00000196591	HDAC2	1.1949	4.43E-05	1.11E-04	0.9277
ENSG00000226479	TMEM185B	1.2605	4.45E-05	1.11E-04	0.9233
ENSG00000025423	HSD17B6	1.1817	4.47E-05	1.11E-04	0.9201
ENSG00000182329	KIAA2012	1.0520	4.47E-05	1.12E-04	0.9185
ENSG00000100036	SLC35E4	1.1513	4.54E-05	1.13E-04	0.9048
ENSG00000108395	TRIM37	1.1832	4.56E-05	1.14E-04	0.8995
ENSG00000176595	KBTBD11	1.1479	4.59E-05	1.15E-04	0.8941
ENSG00000173947	PIFO	1.1086	4.59E-05	1.15E-04	0.8931
ENSG00000181873	IBA57	1.1509	4.60E-05	1.15E-04	0.8923
ENSG00000140859	KIFC3	1.3160	4.62E-05	1.15E-04	0.8879
ENSG00000159884	CCDC107	0.8279	4.62E-05	1.15E-04	0.8872
ENSG00000135372	NAT10	1.1789	4.63E-05	1.15E-04	0.8857
ENSG00000215375	MYL5	1.2268	4.66E-05	1.16E-04	0.8803
ENSG00000213672	NCKIPSD	0.7964	4.67E-05	1.16E-04	0.8786
ENSG00000180817	PPA1	0.8288	4.67E-05	1.16E-04	0.8784
ENSG00000117859	OSBPL9	1.1620	4.68E-05	1.17E-04	0.8754
ENSG00000125352	RNF113A	1.1666	4.69E-05	1.17E-04	0.8736
ENSG00000169071	ROR2	0.5976	4.73E-05	1.18E-04	0.8660
ENSG00000103855	CD276	1.5069	4.74E-05	1.18E-04	0.8630
ENSG00000204161	TMEM273	0.6855	4.77E-05	1.19E-04	0.8583
ENSG00000103148	NPRL3	1.2269	4.77E-05	1.19E-04	0.8580

Gene ID	Gene Symbol	FC	P.Value	adj.P.Val	B
ENSG00000168575	SLC20A2	1.1954	4.77E-05	1.19E-04	0.8572
ENSG00000163536	SERPINI1	0.6539	4.77E-05	1.19E-04	0.8569
ENSG00000136286	MYO1G	1.4804	4.78E-05	1.19E-04	0.8558
ENSG00000129596	CDO1	1.1503	4.79E-05	1.19E-04	0.8536
ENSG00000133935	ERG28	0.8327	4.79E-05	1.19E-04	0.8534
ENSG00000167770	OTUB1	1.1131	4.79E-05	1.19E-04	0.8530
ENSG00000136819	C9orf78	1.1231	4.82E-05	1.20E-04	0.8471
ENSG00000133961	NUMB	1.1924	4.83E-05	1.20E-04	0.8455
ENSG00000183255	PTTG1IP	1.1420	4.84E-05	1.20E-04	0.8447
ENSG00000179088	C12orf42	1.3441	4.84E-05	1.20E-04	0.8441
ENSG00000124802	EEF1E1	0.8056	4.84E-05	1.20E-04	0.8440
ENSG00000099937	SERPIND1	1.0140	4.85E-05	1.21E-04	0.8421
ENSG00000114948	ADAM23	1.2348	4.85E-05	1.21E-04	0.8418
ENSG00000137502	RAB30	1.4077	4.86E-05	1.21E-04	0.8392
ENSG00000137413	TAF8	1.1425	4.87E-05	1.21E-04	0.8379
ENSG00000154914	USP43	1.0578	4.89E-05	1.22E-04	0.8335
ENSG00000183011	NAA38	0.8062	4.92E-05	1.22E-04	0.8292
ENSG00000135316	SYNCRIP	1.1833	4.92E-05	1.22E-04	0.8282
ENSG00000164120	HPGD	1.3003	4.92E-05	1.22E-04	0.8280
ENSG00000168393	DTYMK	1.2231	4.93E-05	1.22E-04	0.8270
ENSG00000123094	RASSF8	1.2212	4.96E-05	1.23E-04	0.8201
ENSG00000103429	BFAR	1.1259	4.97E-05	1.23E-04	0.8195
ENSG00000183833	CFAP91	1.4309	4.97E-05	1.24E-04	0.8179
ENSG00000107282	APBA1	1.2396	5.02E-05	1.25E-04	0.8101
ENSG00000179954	SSC5D	1.0767	5.02E-05	1.25E-04	0.8087
ENSG00000154553	PDLIM3	1.7462	5.02E-05	1.25E-04	0.8084
ENSG00000118680	MYL12B	1.1870	5.05E-05	1.25E-04	0.8035
ENSG00000147400	CETN2	1.2808	5.05E-05	1.25E-04	0.8032
ENSG00000145741	BTF3	0.8805	5.06E-05	1.26E-04	0.8013
ENSG00000151422	FER	1.1855	5.06E-05	1.26E-04	0.8012
ENSG00000079974	RABL2B	1.2474	5.07E-05	1.26E-04	0.8006
ENSG00000134160	TRPM1	1.0523	5.07E-05	1.26E-04	0.7998
ENSG00000215183	MSMP	1.1670	5.08E-05	1.26E-04	0.7987
ENSG00000185561	TLCD2	1.0772	5.08E-05	1.26E-04	0.7977
ENSG00000171848	RRM2	1.5710	5.08E-05	1.26E-04	0.7973
ENSG00000119318	RAD23B	1.1497	5.12E-05	1.27E-04	0.7896
ENSG00000135454	B4GALNT1	1.2465	5.14E-05	1.27E-04	0.7860
ENSG00000163625	WDFY3	1.3734	5.15E-05	1.27E-04	0.7859
ENSG00000107020	PLGRKT	0.8465	5.15E-05	1.28E-04	0.7847
ENSG00000114405	C3orf14	1.3863	5.20E-05	1.29E-04	0.7764
ENSG00000186086	NBPF6	0.7010	5.21E-05	1.29E-04	0.7737
ENSG00000147246	HTR2C	1.2870	5.22E-05	1.29E-04	0.7716
ENSG00000185792	NLRP9	1.0712	5.26E-05	1.30E-04	0.7658
ENSG00000143303	METTL25B	1.2141	5.26E-05	1.30E-04	0.7651
ENSG00000198445	CCT8L2	1.0125	5.27E-05	1.30E-04	0.7632
ENSG00000127252	PLAAT1	1.1791	5.28E-05	1.31E-04	0.7617
ENSG00000131269	ABCB7	1.1480	5.28E-05	1.31E-04	0.7616
ENSG00000250506	CDK3	1.2291	5.31E-05	1.32E-04	0.7552
ENSG00000134198	TSPAN2	1.2876	5.32E-05	1.32E-04	0.7548
ENSG00000189375	TBC1D28	1.1302	5.33E-05	1.32E-04	0.7524
ENSG00000185803	SLC52A2	1.1964	5.38E-05	1.33E-04	0.7438
ENSG00000196792	STRN3	1.1588	5.38E-05	1.33E-04	0.7430
ENSG00000106367	AP1S1	0.7916	5.42E-05	1.34E-04	0.7371
ENSG00000130208	APOC1	0.5633	5.43E-05	1.34E-04	0.7339

Gene ID	Gene Symbol	FC	P.Value	adj.P.Val	B
ENSG00000163017	ACTG2	1.1304	5.43E-05	1.34E-04	0.7339
ENSG00000134343	ANO3	1.0859	5.45E-05	1.35E-04	0.7321
ENSG00000131844	MCCC2	0.8229	5.45E-05	1.35E-04	0.7318
ENSG00000179002	TAS1R2	1.0093	5.45E-05	1.35E-04	0.7319
ENSG00000056050	HPF1	0.7752	5.46E-05	1.35E-04	0.7291
ENSG00000255302	EID1	0.8301	5.46E-05	1.35E-04	0.7293
ENSG00000162365	CYP4A22	1.0395	5.47E-05	1.35E-04	0.7279
ENSG00000126803	HSPA2	1.1405	5.51E-05	1.36E-04	0.7215
ENSG00000170270	GON7	0.8120	5.52E-05	1.36E-04	0.7195
ENSG00000174992	ZG16	1.0172	5.53E-05	1.37E-04	0.7178
ENSG00000088992	TESC	1.4406	5.53E-05	1.37E-04	0.7177
ENSG00000248098	BCKDHA	0.8418	5.54E-05	1.37E-04	0.7160
ENSG00000170276	HSPB2	0.9638	5.58E-05	1.38E-04	0.7094
ENSG00000106853	PTGR1	1.1533	5.58E-05	1.38E-04	0.7082
ENSG00000143816	WNT9A	1.1696	5.59E-05	1.38E-04	0.7077
ENSG00000064489	BORCS8-MEF2B	1.1956	5.60E-05	1.38E-04	0.7052
ENSG00000144560	VGLL4	1.1856	5.62E-05	1.39E-04	0.7029
ENSG00000185499	MUC1	1.6079	5.63E-05	1.39E-04	0.7000
ENSG00000169860	P2RY1	1.1445	5.64E-05	1.39E-04	0.6996
ENSG00000179709	NLRP8	1.0101	5.64E-05	1.39E-04	0.6981
ENSG00000155903	RASA2	1.2112	5.66E-05	1.40E-04	0.6958
ENSG00000166133	RPUSD2	1.1709	5.66E-05	1.40E-04	0.6949
ENSG00000023171	GRAMD1B	1.1822	5.66E-05	1.40E-04	0.6947
ENSG00000111254	AKAP3	1.2032	5.69E-05	1.40E-04	0.6907
ENSG00000165819	METTL3	1.1892	5.74E-05	1.41E-04	0.6829
ENSG00000164627	KIF6	0.7756	5.74E-05	1.42E-04	0.6819
ENSG00000182218	HHIPL1	1.0461	5.76E-05	1.42E-04	0.6797
ENSG00000164300	SERINC5	1.1757	5.82E-05	1.44E-04	0.6683
ENSG00000132744	ACY3	1.5609	5.84E-05	1.44E-04	0.6663
ENSG00000204472	AIF1	0.5970	5.84E-05	1.44E-04	0.6664
ENSG00000184599	TAFA3	1.0105	5.84E-05	1.44E-04	0.6656
ENSG00000111261	MANSC1	0.7238	5.86E-05	1.44E-04	0.6629
ENSG00000115339	GALNT3	1.4311	5.87E-05	1.45E-04	0.6611
ENSG00000107614	TRDMT1	1.2052	5.88E-05	1.45E-04	0.6592
ENSG00000196456	ZNF775	0.7849	5.89E-05	1.45E-04	0.6574
ENSG00000157456	CCNB2	1.4016	5.90E-05	1.45E-04	0.6568
ENSG00000087128	TMPRSS11E	0.6436	5.90E-05	1.45E-04	0.6565
ENSG00000172115	CYCS	0.8227	5.90E-05	1.45E-04	0.6560
ENSG00000148680	HTR7	1.0831	5.92E-05	1.46E-04	0.6534
ENSG00000183579	ZNRF3	1.1500	5.94E-05	1.46E-04	0.6500
ENSG00000072952	IRAG1	1.1170	5.95E-05	1.46E-04	0.6483
ENSG00000267281	ATF7-NPFF	1.1475	5.95E-05	1.46E-04	0.6480
ENSG00000174720	LARP7	1.1559	5.95E-05	1.46E-04	0.6477
ENSG00000158008	EXTL1	1.0283	5.96E-05	1.46E-04	0.6473
ENSG00000178980	SELENOW	0.8381	5.97E-05	1.47E-04	0.6456
ENSG00000018408	WWTR1	1.2305	5.97E-05	1.47E-04	0.6449
ENSG00000167767	KRT80	1.0564	5.97E-05	1.47E-04	0.6445
ENSG00000064692	SNCAIP	1.1940	5.99E-05	1.47E-04	0.6418
ENSG00000125966	MMP24	1.0645	6.01E-05	1.48E-04	0.6392
ENSG00000135905	DOCK10	0.7282	6.06E-05	1.49E-04	0.6302
ENSG00000213928	IRF9	1.2275	6.08E-05	1.49E-04	0.6280
ENSG00000198818	SFT2D1	0.8370	6.08E-05	1.49E-04	0.6278
ENSG00000056998	GYG2	1.0558	6.09E-05	1.50E-04	0.6263
ENSG00000102935	ZNF423	1.1372	6.11E-05	1.50E-04	0.6238

Gene ID	Gene Symbol	FC	P.Value	adj.P.Val	B
ENSG00000183742	MACC1	1.2553	6.11E-05	1.50E-04	0.6238
ENSG00000163106	HPGDS	0.8491	6.11E-05	1.50E-04	0.6231
ENSG00000090581	GNPTG	1.2047	6.15E-05	1.51E-04	0.6171
ENSG00000126262	FFAR2	1.2315	6.15E-05	1.51E-04	0.6171
ENSG00000165192	ASB11	1.0628	6.16E-05	1.51E-04	0.6155
ENSG00000173890	GPR160	0.7567	6.16E-05	1.51E-04	0.6149
ENSG00000132357	CARD6	0.7870	6.19E-05	1.52E-04	0.6110
ENSG00000102870	ZNF629	1.2150	6.19E-05	1.52E-04	0.6102
ENSG00000188313	PLSCR1	1.3501	6.23E-05	1.53E-04	0.6048
ENSG00000072041	SLC6A15	1.1557	6.27E-05	1.54E-04	0.5988
ENSG00000147874	HAUS6	1.1570	6.28E-05	1.54E-04	0.5972
ENSG00000130487	KLHDC7B	0.7712	6.28E-05	1.54E-04	0.5963
ENSG00000197563	PIGN	1.2271	6.30E-05	1.54E-04	0.5945
ENSG00000158560	DYNC1I1	1.2106	6.30E-05	1.54E-04	0.5943
ENSG00000170166	HOXD4	1.0850	6.35E-05	1.56E-04	0.5872
ENSG00000103044	HAS3	1.1242	6.36E-05	1.56E-04	0.5855
ENSG00000197535	MYO5A	1.3058	6.37E-05	1.56E-04	0.5834
ENSG00000141854	MISP3	1.2640	6.38E-05	1.56E-04	0.5827
ENSG00000145191	EIF2B5	1.1444	6.40E-05	1.57E-04	0.5791
ENSG00000141376	BCAS3	1.1798	6.40E-05	1.57E-04	0.5786
ENSG00000079689	SCGN	1.1033	6.43E-05	1.58E-04	0.5743
ENSG00000186976	EFCAB6	1.1231	6.44E-05	1.58E-04	0.5735
ENSG00000243056	EIF4EBP3	0.7187	6.44E-05	1.58E-04	0.5732
ENSG00000204220	PFDN6	0.8384	6.44E-05	1.58E-04	0.5726
ENSG00000128645	HOXD1	1.3671	6.45E-05	1.58E-04	0.5719
ENSG00000188707	ZBED6CL	1.2087	6.45E-05	1.58E-04	0.5718
ENSG00000110786	PTPN5	1.0539	6.51E-05	1.59E-04	0.5630
ENSG00000149256	TENM4	0.8365	6.55E-05	1.60E-04	0.5578
ENSG00000188636	RTL6	1.3275	6.55E-05	1.60E-04	0.5567
ENSG00000033800	PIAS1	1.1856	6.56E-05	1.60E-04	0.5563
ENSG00000111802	TDP2	1.1425	6.56E-05	1.61E-04	0.5552
ENSG00000146083	RNF44	1.1877	6.58E-05	1.61E-04	0.5524
ENSG00000164898	FMC1	0.8180	6.63E-05	1.62E-04	0.5461
ENSG00000048991	R3HDM1	1.2111	6.67E-05	1.63E-04	0.5398
ENSG00000109265	CRACD	1.0391	6.69E-05	1.64E-04	0.5368
ENSG00000148541	FAM13C	1.0846	6.70E-05	1.64E-04	0.5357
ENSG00000105464	GRIN2D	1.1069	6.72E-05	1.64E-04	0.5333
ENSG00000163697	APBB2	1.3171	6.74E-05	1.65E-04	0.5297
ENSG00000169981	ZNF35	1.1581	6.76E-05	1.65E-04	0.5269
ENSG00000168040	FADD	1.1938	6.77E-05	1.65E-04	0.5265
ENSG00000163827	LRRC2	1.1510	6.77E-05	1.65E-04	0.5262
ENSG00000186026	ZNF284	1.1399	6.77E-05	1.65E-04	0.5261
ENSG00000184343	SRPK3	1.2844	6.78E-05	1.65E-04	0.5251
ENSG00000129250	KIF1C	1.2380	6.78E-05	1.66E-04	0.5239
ENSG00000117222	RBBP5	1.1916	6.81E-05	1.66E-04	0.5210
ENSG00000083093	PALB2	1.1710	6.81E-05	1.66E-04	0.5198
ENSG00000133065	SLC41A1	1.2185	6.83E-05	1.67E-04	0.5179
ENSG00000162600	OMA1	0.7696	6.87E-05	1.68E-04	0.5114
ENSG00000197614	MFAP5	1.1470	6.89E-05	1.68E-04	0.5093
ENSG00000183828	NUDT14	1.3300	6.93E-05	1.69E-04	0.5035
ENSG00000138792	ENPEP	1.1708	6.97E-05	1.70E-04	0.4987
ENSG00000102032	RENBP	1.2961	6.98E-05	1.70E-04	0.4970
ENSG00000155761	SPAG17	0.8205	7.01E-05	1.71E-04	0.4925
ENSG00000018280	SLC11A1	1.4726	7.01E-05	1.71E-04	0.4923

Gene ID	Gene Symbol	FC	P.Value	adj.P.Val	B
ENSG00000138495	COX17	0.8468	7.04E-05	1.72E-04	0.4890
ENSG00000149091	DGKZ	1.2119	7.04E-05	1.72E-04	0.4885
ENSG00000087884	AAMDC	0.8158	7.06E-05	1.72E-04	0.4866
ENSG00000171133	OR2K2	1.0404	7.07E-05	1.72E-04	0.4844
ENSG00000169084	DHRSX	1.2278	7.08E-05	1.73E-04	0.4836
ENSG00000153551	CMTM7	0.7483	7.11E-05	1.73E-04	0.4792
ENSG00000235453	SMIM27	0.8535	7.12E-05	1.74E-04	0.4778
ENSG00000146070	PLA2G7	0.7114	7.13E-05	1.74E-04	0.4764
ENSG00000164087	POC1A	1.2563	7.15E-05	1.74E-04	0.4749
ENSG00000117834	SLC5A9	1.0584	7.16E-05	1.74E-04	0.4730
ENSG00000197863	ZNF790	0.8169	7.17E-05	1.75E-04	0.4717
ENSG00000104205	SGK3	1.1935	7.18E-05	1.75E-04	0.4699
ENSG00000115828	QPCT	0.5844	7.20E-05	1.75E-04	0.4683
ENSG00000112146	FBXO9	1.1468	7.21E-05	1.76E-04	0.4660
ENSG00000158113	LRRC43	1.1009	7.22E-05	1.76E-04	0.4653
ENSG00000187080	OR2AK2	1.0166	7.27E-05	1.77E-04	0.4588
ENSG00000150977	RILPL2	1.2777	7.30E-05	1.77E-04	0.4552
ENSG00000134333	LDHA	1.2562	7.31E-05	1.78E-04	0.4536
ENSG00000144741	SLC25A26	0.8875	7.31E-05	1.78E-04	0.4529
ENSG00000171621	SPSB1	1.3772	7.32E-05	1.78E-04	0.4518
ENSG00000129625	REEP5	0.8562	7.37E-05	1.79E-04	0.4459
ENSG00000131187	F12	0.6951	7.37E-05	1.79E-04	0.4450
ENSG00000118520	ARG1	1.4022	7.40E-05	1.80E-04	0.4412
ENSG00000198088	NUP62CL	1.2933	7.41E-05	1.80E-04	0.4410
ENSG00000145781	COMMD10	0.8360	7.41E-05	1.80E-04	0.4403
ENSG00000119121	TRPM6	1.0652	7.42E-05	1.80E-04	0.4393
ENSG00000178386	ZNF223	1.2536	7.45E-05	1.81E-04	0.4348
ENSG00000162194	LBHD1	0.8371	7.47E-05	1.81E-04	0.4328
ENSG00000169946	ZFPM2	1.2257	7.48E-05	1.82E-04	0.4314
ENSG00000091106	NLRC4	0.8848	7.48E-05	1.82E-04	0.4310
ENSG00000198336	MYL4	0.7607	7.50E-05	1.82E-04	0.4295
ENSG00000198382	UVRAG	1.2289	7.51E-05	1.82E-04	0.4276
ENSG00000072310	SREBF1	1.2873	7.52E-05	1.83E-04	0.4262
ENSG00000154654	NCAM2	1.3990	7.52E-05	1.83E-04	0.4259
ENSG00000148688	RPP30	1.1566	7.53E-05	1.83E-04	0.4249
ENSG00000116521	SCAMP3	1.1927	7.54E-05	1.83E-04	0.4245
ENSG00000214946	TBC1D26	1.2457	7.54E-05	1.83E-04	0.4241
ENSG00000137976	DNASE2B	0.9503	7.55E-05	1.83E-04	0.4226
ENSG00000135318	NT5E	1.3984	7.58E-05	1.84E-04	0.4186
ENSG00000167766	ZNF83	1.4093	7.59E-05	1.84E-04	0.4174
ENSG00000136854	STXBP1	1.2904	7.64E-05	1.85E-04	0.4111
ENSG00000185467	KPNA7	1.0177	7.65E-05	1.86E-04	0.4104
ENSG00000198482	ZNF808	1.3126	7.68E-05	1.86E-04	0.4070
ENSG00000152240	HAUS1	1.1911	7.68E-05	1.86E-04	0.4063
ENSG00000153132	CLGN	1.4408	7.74E-05	1.88E-04	0.3987
ENSG00000188917	TRMT2B	1.2135	7.75E-05	1.88E-04	0.3975
ENSG00000102904	TSNAXIP1	1.1346	7.77E-05	1.88E-04	0.3953
ENSG00000164136	IL15	1.3777	7.78E-05	1.89E-04	0.3938
ENSG00000115514	TXNDC9	0.8137	7.83E-05	1.90E-04	0.3882
ENSG00000204869	IGFL4	1.1240	7.92E-05	1.92E-04	0.3770
ENSG00000138039	LHCGR	1.0472	7.96E-05	1.93E-04	0.3727
ENSG00000105953	OGDH	0.7961	7.99E-05	1.93E-04	0.3696
ENSG00000127325	BEST3	1.1102	8.01E-05	1.94E-04	0.3672
ENSG00000099985	OSM	1.3181	8.01E-05	1.94E-04	0.3666

Gene ID	Gene Symbol	FC	P.Value	adj.P.Val	B
ENSG00000169402	RSPH10B2	1.1151	8.03E-05	1.94E-04	0.3645
ENSG00000138119	MYOF	0.6861	8.05E-05	1.95E-04	0.3625
ENSG00000138413	IDH1	0.7800	8.05E-05	1.95E-04	0.3626
ENSG00000184313	MROH7	1.1012	8.05E-05	1.95E-04	0.3627
ENSG00000179588	ZFPM1	1.2446	8.05E-05	1.95E-04	0.3620
ENSG00000151025	GPR158	1.1149	8.08E-05	1.96E-04	0.3581
ENSG00000158715	SLC45A3	1.1412	8.09E-05	1.96E-04	0.3571
ENSG00000166924	NYAP1	1.0327	8.13E-05	1.97E-04	0.3522
ENSG00000106125	MINDY4	1.1455	8.14E-05	1.97E-04	0.3515
ENSG00000181631	P2RY13	0.7677	8.15E-05	1.97E-04	0.3504
ENSG00000196247	ZNF107	1.2208	8.17E-05	1.98E-04	0.3476
ENSG00000132970	WASF3	1.1566	8.18E-05	1.98E-04	0.3471
ENSG00000196177	ACADSB	1.1918	8.19E-05	1.98E-04	0.3456
ENSG00000161682	FAM171A2	1.0712	8.23E-05	1.99E-04	0.3415
ENSG00000189186	DCAF8L2	1.1012	8.24E-05	1.99E-04	0.3397
ENSG00000138100	TRIM54	1.1111	8.25E-05	1.99E-04	0.3389
ENSG00000135314	KHDC1	1.1947	8.27E-05	2.00E-04	0.3372
ENSG00000188157	AGRN	1.3261	8.28E-05	2.00E-04	0.3351
ENSG00000171532	NEUROD2	1.0195	8.29E-05	2.00E-04	0.3347
ENSG00000213889	PPM1N	1.2586	8.31E-05	2.01E-04	0.3322
ENSG00000163171	CDC42EP3	1.2445	8.36E-05	2.02E-04	0.3265
ENSG00000168672	LRATD2	1.5266	8.37E-05	2.02E-04	0.3252
ENSG00000019549	SNAI2	1.1633	8.38E-05	2.02E-04	0.3244
ENSG00000178177	LCORL	1.1756	8.39E-05	2.03E-04	0.3226
ENSG00000127948	POR	1.1635	8.41E-05	2.03E-04	0.3211
ENSG00000237693	IRGM	1.0782	8.41E-05	2.03E-04	0.3208
ENSG00000186790	FOXE3	1.0208	8.42E-05	2.03E-04	0.3196
ENSG00000065371	ROPN1	1.0304	8.44E-05	2.04E-04	0.3173
ENSG00000226763	SRRM5	1.1128	8.46E-05	2.04E-04	0.3153
ENSG00000121964	GTDC1	1.2270	8.48E-05	2.04E-04	0.3132
ENSG00000115204	MPV17	1.1789	8.49E-05	2.05E-04	0.3113
ENSG00000143797	MBOAT2	1.4151	8.50E-05	2.05E-04	0.3102
ENSG00000176531	PHLDB3	1.1784	8.50E-05	2.05E-04	0.3102
ENSG00000141314	RHBDL3	1.0812	8.51E-05	2.05E-04	0.3098
ENSG00000106333	PCOLCE	1.2737	8.54E-05	2.06E-04	0.3060
ENSG00000204193	TXNDC8	0.9002	8.56E-05	2.06E-04	0.3046
ENSG00000169714	CNBP	0.8928	8.59E-05	2.07E-04	0.3008
ENSG00000181904	C5orf24	0.8432	8.63E-05	2.08E-04	0.2962
ENSG00000166930	MS4A5	1.0636	8.67E-05	2.09E-04	0.2925
ENSG00000169733	RFNG	1.1756	8.70E-05	2.09E-04	0.2891
ENSG00000129467	ADCY4	1.1542	8.70E-05	2.10E-04	0.2889
ENSG00000103534	TMC5	1.1882	8.71E-05	2.10E-04	0.2873
ENSG00000132382	MYBBP1A	1.2250	8.73E-05	2.10E-04	0.2860
ENSG00000111817	DSE	1.2292	8.77E-05	2.11E-04	0.2812
ENSG00000100353	EIF3D	1.1344	8.78E-05	2.11E-04	0.2799
ENSG00000187581	COX8C	1.0483	8.80E-05	2.12E-04	0.2776
ENSG00000151136	BTBD11	1.0424	8.82E-05	2.12E-04	0.2761
ENSG00000154917	RAB6B	1.1864	8.96E-05	2.16E-04	0.2608
ENSG00000100558	PLEK2	1.1101	8.97E-05	2.16E-04	0.2600
ENSG00000259399	TGIF2-RAB5IF	1.2013	8.98E-05	2.16E-04	0.2591
ENSG00000135862	LAMC1	1.3276	9.03E-05	2.17E-04	0.2531
ENSG00000111424	VDR	0.7915	9.06E-05	2.18E-04	0.2504
ENSG00000198682	PAPSS2	1.3341	9.08E-05	2.18E-04	0.2489
ENSG00000137288	UQCC2	0.7978	9.10E-05	2.19E-04	0.2467

Gene ID	Gene Symbol	FC	P.Value	adj.P.Val	B
ENSG00000139174	PRICKLE1	1.2243	9.11E-05	2.19E-04	0.2456
ENSG00000072954	TMEM38A	0.7710	9.11E-05	2.19E-04	0.2454
ENSG00000225973	PIGBOS1	0.8804	9.17E-05	2.20E-04	0.2387
ENSG00000186073	CDIN1	1.2047	9.21E-05	2.21E-04	0.2350
ENSG00000187486	KCNJ11	0.7011	9.23E-05	2.22E-04	0.2326
ENSG00000133135	RNF128	1.1694	9.24E-05	2.22E-04	0.2316
ENSG00000124383	MPHOSPH10	1.1484	9.26E-05	2.23E-04	0.2295
ENSG00000100142	POLR2F	0.8438	9.27E-05	2.23E-04	0.2292
ENSG00000169550	MUC15	1.2598	9.28E-05	2.23E-04	0.2283
ENSG00000186281	GPAT2	1.5527	9.34E-05	2.24E-04	0.2215
ENSG00000108946	PRKAR1A	1.1776	9.35E-05	2.25E-04	0.2203
ENSG00000180083	WFDC11	1.0866	9.36E-05	2.25E-04	0.2197
ENSG00000162998	FRZB	0.4355	9.38E-05	2.25E-04	0.2176
ENSG00000151131	NOPCHAP1	0.8157	9.47E-05	2.27E-04	0.2084
ENSG00000148459	PDSS1	1.1846	9.47E-05	2.27E-04	0.2082
ENSG00000163807	KIAA1143	1.1455	9.50E-05	2.28E-04	0.2061
ENSG00000113389	NPR3	1.0842	9.52E-05	2.28E-04	0.2039
ENSG00000185008	ROBO2	1.2358	9.53E-05	2.29E-04	0.2028
ENSG00000110768	GTF2H1	0.8413	9.55E-05	2.29E-04	0.2003
ENSG00000230657	PRB4	1.0203	9.57E-05	2.29E-04	0.1989
ENSG00000069849	ATP1B3	1.2351	9.57E-05	2.30E-04	0.1986
ENSG00000148950	IMMP1L	0.8372	9.63E-05	2.31E-04	0.1928
ENSG00000005206	SPPL2B	1.1992	9.64E-05	2.31E-04	0.1922
ENSG00000001036	FUCA2	0.7905	9.65E-05	2.31E-04	0.1911
ENSG00000196693	ZNF33B	0.8365	9.66E-05	2.32E-04	0.1899
ENSG00000090565	RAB11FIP3	1.2202	9.69E-05	2.32E-04	0.1866
ENSG00000141744	PNMT	1.1052	9.69E-05	2.32E-04	0.1867
ENSG00000116016	EPAS1	0.7122	9.73E-05	2.33E-04	0.1834
ENSG00000073008	PVR	1.2500	9.73E-05	2.33E-04	0.1831
ENSG00000160948	VPS28	1.1259	9.78E-05	2.34E-04	0.1786
ENSG00000158604	TMED4	1.1158	9.81E-05	2.35E-04	0.1749
ENSG00000198876	DCAF12	0.8272	9.84E-05	2.36E-04	0.1725
ENSG00000188735	TMEM120B	1.1762	9.84E-05	2.36E-04	0.1723
ENSG00000170153	RNF150	1.0623	9.87E-05	2.36E-04	0.1692
ENSG00000185838	GNB1L	0.8588	9.87E-05	2.36E-04	0.1692
ENSG00000070669	ASNS	0.7990	9.88E-05	2.36E-04	0.1686
ENSG00000180398	MCFD2	1.1606	9.89E-05	2.37E-04	0.1680
ENSG00000175455	CCDC14	1.3089	9.90E-05	2.37E-04	0.1666
ENSG00000095637	SORBS1	1.1533	9.91E-05	2.37E-04	0.1656
ENSG00000100075	SLC25A1	1.1839	9.94E-05	2.38E-04	0.1628
ENSG00000188064	WNT7B	1.0231	9.94E-05	2.38E-04	0.1629
ENSG00000125355	TMEM255A	1.4544	9.96E-05	2.38E-04	0.1605
ENSG00000188375	H3-5	1.1040	9.97E-05	2.38E-04	0.1603
ENSG00000198796	ALPK2	1.2625	9.97E-05	2.38E-04	0.1602
ENSG00000145864	GABRB2	1.2410	9.99E-05	2.39E-04	0.1581
ENSG00000165462	PHOX2A	1.0366	1.01E-04	2.42E-04	0.1465
ENSG00000103064	SLC7A6	1.2227	1.01E-04	2.42E-04	0.1463
ENSG00000125124	BBS2	1.2055	1.01E-04	2.42E-04	0.1437
ENSG00000215131	C16orf90	1.0153	1.02E-04	2.43E-04	0.1415
ENSG00000120254	MTHFD1L	1.3479	1.02E-04	2.44E-04	0.1387
ENSG00000186010	NDUFA13	0.8731	1.02E-04	2.44E-04	0.1356
ENSG00000162390	ACOT11	1.1281	1.02E-04	2.45E-04	0.1351
ENSG00000156017	CARNMT1	0.8245	1.03E-04	2.45E-04	0.1337
ENSG00000162433	AK4	1.1190	1.03E-04	2.45E-04	0.1335

Gene ID	Gene Symbol	FC	P.Value	adj.P.Val	B
ENSG00000151176	PLBD2	1.1916	1.03E-04	2.45E-04	0.1330
ENSG00000196968	FUT11	1.1702	1.03E-04	2.45E-04	0.1325
ENSG00000171155	C1GALT1C1	0.8141	1.03E-04	2.46E-04	0.1298
ENSG00000148400	NOTCH1	1.2467	1.04E-04	2.47E-04	0.1240
ENSG00000224960	PPP4R3C	1.1916	1.04E-04	2.48E-04	0.1212
ENSG00000143252	SDHC	1.1924	1.04E-04	2.48E-04	0.1203
ENSG00000140983	RHOT2	1.1941	1.04E-04	2.48E-04	0.1197
ENSG00000102934	PLLP	1.0701	1.05E-04	2.50E-04	0.1144
ENSG00000165164	CFAP47	1.1201	1.05E-04	2.50E-04	0.1143
ENSG00000033327	GAB2	1.3443	1.05E-04	2.50E-04	0.1135
ENSG00000174469	CNTNAP2	1.2358	1.05E-04	2.51E-04	0.1099
ENSG00000130164	LDLR	1.5156	1.06E-04	2.52E-04	0.1063
ENSG00000182378	PLCXD1	1.2934	1.06E-04	2.52E-04	0.1059
ENSG00000109255	NMU	1.2526	1.06E-04	2.52E-04	0.1049
ENSG00000174109	C16orf91	1.1575	1.06E-04	2.52E-04	0.1048
ENSG00000167800	TBX10	1.1166	1.06E-04	2.52E-04	0.1031
ENSG00000106080	FKBP14	1.2736	1.07E-04	2.54E-04	0.0966
ENSG00000124253	PCK1	1.0282	1.07E-04	2.54E-04	0.0955
ENSG00000100065	CARD10	1.0846	1.07E-04	2.55E-04	0.0944
ENSG00000184281	TSSC4	1.1435	1.07E-04	2.55E-04	0.0944
ENSG00000144749	LRIG1	1.3309	1.07E-04	2.56E-04	0.0894
ENSG00000172216	CEBPB	1.2917	1.07E-04	2.56E-04	0.0892
ENSG00000148143	ZNF462	1.0748	1.08E-04	2.56E-04	0.0889
ENSG00000137491	SLCO2B1	0.6968	1.08E-04	2.56E-04	0.0880
ENSG00000137054	POLR1E	0.8229	1.08E-04	2.57E-04	0.0860
ENSG00000105948	TTC26	1.1550	1.08E-04	2.57E-04	0.0846
ENSG00000118402	ELOVL4	0.6601	1.08E-04	2.58E-04	0.0815
ENSG00000136243	NUP42	0.8285	1.08E-04	2.58E-04	0.0804
ENSG00000173706	HEG1	1.2704	1.09E-04	2.59E-04	0.0787
ENSG00000112984	KIF20A	1.2956	1.09E-04	2.59E-04	0.0784
ENSG00000164867	NOS3	1.2413	1.09E-04	2.59E-04	0.0775
ENSG00000198216	CACNA1E	1.0736	1.09E-04	2.59E-04	0.0757
ENSG00000037757	MRI1	0.7675	1.09E-04	2.59E-04	0.0750
ENSG00000109016	DHRS7B	0.8228	1.09E-04	2.60E-04	0.0734
ENSG00000158402	CDC25C	1.2216	1.09E-04	2.60E-04	0.0722
ENSG00000165501	LRR1	1.1632	1.09E-04	2.60E-04	0.0721
ENSG00000183305	MAGEA2B	1.2729	1.09E-04	2.60E-04	0.0717
ENSG00000157593	SLC35B2	1.1483	1.10E-04	2.60E-04	0.0713
ENSG00000131473	ACLY	1.1731	1.10E-04	2.61E-04	0.0699
ENSG00000160570	DEDD2	1.1586	1.10E-04	2.61E-04	0.0692
ENSG00000023909	GCLM	1.2016	1.10E-04	2.62E-04	0.0663
ENSG00000170271	FAXDC2	1.1557	1.10E-04	2.62E-04	0.0632
ENSG00000151093	OXSM	0.8571	1.12E-04	2.65E-04	0.0540
ENSG00000143217	NECTIN4	1.0699	1.12E-04	2.65E-04	0.0530
ENSG00000178538	CA8	1.4829	1.12E-04	2.65E-04	0.0522
ENSG00000011478	QPCTL	0.8323	1.12E-04	2.65E-04	0.0518
ENSG00000138964	PARVG	1.5354	1.13E-04	2.67E-04	0.0452
ENSG00000185303	SFTPA2	1.0820	1.13E-04	2.68E-04	0.0443
ENSG00000122733	PHF24	1.0819	1.13E-04	2.68E-04	0.0442
ENSG00000188015	S100A3	1.0957	1.13E-04	2.68E-04	0.0427
ENSG00000164830	OXR1	1.1819	1.13E-04	2.68E-04	0.0420
ENSG00000182533	CAV3	1.0205	1.13E-04	2.68E-04	0.0414
ENSG00000136463	TACO1	1.1514	1.13E-04	2.69E-04	0.0396
ENSG00000164825	DEFB1	0.7597	1.13E-04	2.69E-04	0.0397

Gene ID	Gene Symbol	FC	P.Value	adj.P.Val	B
ENSG00000188677	PARVB	1.2278	1.13E-04	2.69E-04	0.0389
ENSG00000134882	UBAC2	0.8166	1.14E-04	2.69E-04	0.0366
ENSG00000110448	CD5	1.1953	1.14E-04	2.70E-04	0.0360
ENSG00000187123	LYPD6	1.0937	1.14E-04	2.70E-04	0.0347
ENSG00000186462	NAP1L2	1.6120	1.15E-04	2.72E-04	0.0284
ENSG00000149294	NCAM1	0.5103	1.15E-04	2.72E-04	0.0261
ENSG00000182512	GLRX5	1.1597	1.15E-04	2.73E-04	0.0233
ENSG00000185267	CDNF	1.2134	1.16E-04	2.74E-04	0.0209
ENSG00000059769	DNAJC25	1.1391	1.16E-04	2.74E-04	0.0188
ENSG00000075914	EXOSC7	1.1220	1.16E-04	2.74E-04	0.0184
ENSG00000248485	PCP4L1	1.0685	1.16E-04	2.74E-04	0.0185
ENSG00000166278	C2	0.6125	1.16E-04	2.75E-04	0.0160
ENSG00000132603	NIP7	1.1647	1.16E-04	2.75E-04	0.0154
ENSG00000164342	TLR3	0.8360	1.16E-04	2.75E-04	0.0153
ENSG00000130150	MOSPD2	1.2046	1.16E-04	2.76E-04	0.0138
ENSG00000069812	HES2	1.0604	1.16E-04	2.76E-04	0.0134
ENSG00000128928	IVD	0.8164	1.16E-04	2.76E-04	0.0132
ENSG00000102043	MTMR8	1.1236	1.17E-04	2.77E-04	0.0084
ENSG00000119147	ECRG4	1.1044	1.17E-04	2.77E-04	0.0069
ENSG00000184156	KCNQ3	1.1258	1.17E-04	2.78E-04	0.0057
ENSG00000254959	INMT-MINDY4	1.0925	1.18E-04	2.79E-04	0.0024
ENSG00000118004	COLEC11	1.1846	1.20E-04	2.84E-04	-0.0145
ENSG00000177673	TEX44	1.0088	1.20E-04	2.85E-04	-0.0184
ENSG00000164983	TMEM65	1.2494	1.21E-04	2.87E-04	-0.0241
ENSG00000107949	BCCIP	1.1265	1.21E-04	2.87E-04	-0.0243
ENSG00000172458	IL17D	0.8686	1.21E-04	2.87E-04	-0.0260
ENSG00000166920	C15orf48	1.2823	1.21E-04	2.87E-04	-0.0262
ENSG00000152207	CYSLTR2	0.8027	1.22E-04	2.87E-04	-0.0274
ENSG00000114738	MAPKAPK3	0.8651	1.22E-04	2.89E-04	-0.0311
ENSG00000139160	ETFBKMT	1.1437	1.22E-04	2.89E-04	-0.0315
ENSG00000163882	POLR2H	0.8692	1.22E-04	2.89E-04	-0.0318
ENSG00000139351	SYCP3	1.2863	1.22E-04	2.89E-04	-0.0326
ENSG00000167613	LAIR1	1.6232	1.22E-04	2.89E-04	-0.0338
ENSG00000118420	UBE3D	0.8531	1.23E-04	2.89E-04	-0.0343
ENSG00000179168	GGN	1.1219	1.23E-04	2.90E-04	-0.0366
ENSG00000130950	NUTM2F	1.0495	1.23E-04	2.91E-04	-0.0408
ENSG00000141434	MEP1B	1.1218	1.23E-04	2.91E-04	-0.0409
ENSG00000074266	EED	1.1901	1.24E-04	2.92E-04	-0.0439
ENSG00000197745	SCGB1D4	1.0534	1.25E-04	2.94E-04	-0.0506
ENSG00000004838	ZMYND10	1.2274	1.25E-04	2.94E-04	-0.0512
ENSG00000104953	TLE6	1.2043	1.25E-04	2.95E-04	-0.0526
ENSG00000068615	REEP1	1.1493	1.25E-04	2.96E-04	-0.0556
ENSG00000074855	ANO8	1.2787	1.26E-04	2.96E-04	-0.0578
ENSG00000151466	SCLT1	1.1609	1.26E-04	2.96E-04	-0.0582
ENSG00000174236	REP15	0.9715	1.26E-04	2.97E-04	-0.0601
ENSG00000104915	STX10	0.8635	1.26E-04	2.98E-04	-0.0632
ENSG00000159423	ALDH4A1	1.2194	1.27E-04	2.98E-04	-0.0648
ENSG00000243244	STON1	1.0816	1.27E-04	2.99E-04	-0.0657
ENSG00000161040	FBXL13	1.1549	1.27E-04	2.99E-04	-0.0669
ENSG00000019485	PRDM11	1.2022	1.27E-04	3.00E-04	-0.0701
ENSG00000091972	CD200	0.6197	1.28E-04	3.01E-04	-0.0727
ENSG00000118729	CASQ2	1.1629	1.28E-04	3.01E-04	-0.0728
ENSG00000130045	NXNL2	1.2238	1.28E-04	3.02E-04	-0.0765
ENSG00000124659	TBCC	1.1614	1.29E-04	3.03E-04	-0.0797

Gene ID	Gene Symbol	FC	P.Value	adj.P.Val	B
ENSG00000175161	CADM2	1.2983	1.29E-04	3.03E-04	-0.0809
ENSG00000180104	EXOC3	1.1229	1.29E-04	3.03E-04	-0.0817
ENSG00000055130	CUL1	1.1434	1.29E-04	3.03E-04	-0.0820
ENSG00000137090	DMRT1	1.0542	1.29E-04	3.04E-04	-0.0832
ENSG00000129244	ATP1B2	1.0646	1.29E-04	3.04E-04	-0.0850
ENSG00000062822	POLD1	1.2391	1.30E-04	3.05E-04	-0.0871
ENSG00000157881	PANK4	1.1766	1.30E-04	3.06E-04	-0.0907
ENSG00000255151	GLYATL1B	1.0661	1.30E-04	3.06E-04	-0.0913
ENSG00000125823	CSTL1	1.1434	1.30E-04	3.06E-04	-0.0921
ENSG00000177144	NUDT4B	1.2025	1.30E-04	3.07E-04	-0.0923
ENSG00000115128	SF3B6	1.1378	1.30E-04	3.07E-04	-0.0936
ENSG00000075213	SEMA3A	1.4193	1.30E-04	3.07E-04	-0.0939
ENSG00000213967	ZNF726	1.2100	1.31E-04	3.07E-04	-0.0940
ENSG00000088970	KIZ	1.1569	1.31E-04	3.08E-04	-0.0974
ENSG00000120332	TNN	1.0663	1.31E-04	3.09E-04	-0.1008
ENSG00000165757	JCAD	1.0425	1.32E-04	3.11E-04	-0.1062
ENSG00000204947	ZNF425	0.8556	1.32E-04	3.11E-04	-0.1069
ENSG00000187987	ZSCAN23	0.7976	1.32E-04	3.11E-04	-0.1071
ENSG00000068971	PPP2R5B	1.1765	1.32E-04	3.11E-04	-0.1081
ENSG00000007350	TKTL1	1.1296	1.33E-04	3.11E-04	-0.1086
ENSG00000170959	DCDC1	1.0973	1.33E-04	3.11E-04	-0.1087
ENSG00000137494	ANKRD42	1.2064	1.33E-04	3.12E-04	-0.1094
ENSG00000164122	ASB5	0.7618	1.33E-04	3.12E-04	-0.1094
ENSG00000198171	DDRGK1	1.1715	1.33E-04	3.12E-04	-0.1099
ENSG00000146197	SCUBE3	1.0511	1.33E-04	3.12E-04	-0.1119
ENSG00000125149	PHAF1	1.1579	1.33E-04	3.12E-04	-0.1121
ENSG00000103365	GGA2	1.1902	1.33E-04	3.12E-04	-0.1123
ENSG00000176692	FOXC2	1.0278	1.34E-04	3.14E-04	-0.1165
ENSG00000065054	SLC9A3R2	0.6835	1.34E-04	3.14E-04	-0.1175
ENSG00000145945	FAM50B	0.7338	1.34E-04	3.14E-04	-0.1185
ENSG00000103335	PIEZO1	1.2817	1.35E-04	3.16E-04	-0.1239
ENSG00000163938	GNL3	1.1489	1.35E-04	3.17E-04	-0.1252
ENSG00000188778	ADRB3	1.0190	1.35E-04	3.17E-04	-0.1259
ENSG00000251369	ZNF550	1.2188	1.35E-04	3.17E-04	-0.1280
ENSG00000168743	NPNT	1.3341	1.35E-04	3.18E-04	-0.1288
ENSG00000196664	TLR7	1.3832	1.36E-04	3.18E-04	-0.1295
ENSG00000214960	CRPPA	1.1018	1.36E-04	3.18E-04	-0.1308
ENSG00000136270	TBRG4	1.1769	1.37E-04	3.20E-04	-0.1374
ENSG00000147485	PXDNL	0.8424	1.37E-04	3.22E-04	-0.1409
ENSG00000048392	RRM2B	1.1821	1.37E-04	3.22E-04	-0.1415
ENSG00000152133	GPATCH11	1.2415	1.37E-04	3.22E-04	-0.1423
ENSG00000173726	TOMM20	1.1069	1.38E-04	3.22E-04	-0.1432
ENSG00000136897	MRPL50	0.8385	1.38E-04	3.23E-04	-0.1448
ENSG00000213123	DYNLT2B	1.2326	1.38E-04	3.23E-04	-0.1448
ENSG00000144730	IL17RD	1.0377	1.38E-04	3.24E-04	-0.1483
ENSG00000255501	CARD18	1.1087	1.38E-04	3.24E-04	-0.1495
ENSG00000048740	CELF2	1.2767	1.39E-04	3.25E-04	-0.1517
ENSG00000214652	ZNF727	1.1139	1.39E-04	3.26E-04	-0.1534
ENSG00000136717	BIN1	0.7056	1.39E-04	3.26E-04	-0.1551
ENSG00000109270	LAMTOR3	1.1803	1.40E-04	3.27E-04	-0.1569
ENSG00000110321	EIF4G2	1.1475	1.40E-04	3.27E-04	-0.1571
ENSG00000113302	IL12B	1.0778	1.40E-04	3.28E-04	-0.1603
ENSG00000147687	TATDN1	0.8828	1.40E-04	3.29E-04	-0.1627
ENSG00000086991	NOX4	1.0578	1.41E-04	3.29E-04	-0.1643

Gene ID	Gene Symbol	FC	P.Value	adj.P.Val	B
ENSG00000028839	TBPL1	1.1524	1.41E-04	3.29E-04	-0.1655
ENSG00000164764	SBSPON	1.0756	1.41E-04	3.30E-04	-0.1685
ENSG00000153790	C7orf31	0.8506	1.42E-04	3.31E-04	-0.1708
ENSG00000132581	SDF2	0.8641	1.42E-04	3.31E-04	-0.1715
ENSG00000174721	FGFBP3	1.1392	1.42E-04	3.31E-04	-0.1717
ENSG00000215114	UBXN2B	1.1723	1.42E-04	3.32E-04	-0.1740
ENSG00000178662	CSRNP3	1.0916	1.42E-04	3.32E-04	-0.1747
ENSG00000149016	TUT1	1.1603	1.43E-04	3.33E-04	-0.1773
ENSG00000184840	TMED9	0.8579	1.43E-04	3.33E-04	-0.1780
ENSG00000171408	PDE7B	1.0229	1.43E-04	3.34E-04	-0.1784
ENSG00000104299	INTS9	1.1740	1.43E-04	3.34E-04	-0.1789
ENSG00000254852	NPIPA2	1.2312	1.43E-04	3.34E-04	-0.1807
ENSG00000267680	ZNF224	1.2085	1.43E-04	3.35E-04	-0.1824
ENSG00000196821	ILRUN	1.1770	1.45E-04	3.38E-04	-0.1907
ENSG00000185627	PSMD13	1.1245	1.47E-04	3.43E-04	-0.2039
ENSG00000173511	VEGFB	1.1657	1.47E-04	3.43E-04	-0.2051
ENSG00000139780	METTL21C	1.0321	1.47E-04	3.44E-04	-0.2073
ENSG00000107165	TYRP1	0.7715	1.47E-04	3.44E-04	-0.2074
ENSG00000166595	CIAO2B	1.1567	1.47E-04	3.44E-04	-0.2077
ENSG00000135426	TESPA1	1.2041	1.47E-04	3.44E-04	-0.2084
ENSG00000117528	ABCD3	1.1762	1.47E-04	3.44E-04	-0.2086
ENSG00000159873	CCDC117	1.2118	1.48E-04	3.44E-04	-0.2097
ENSG00000103888	CEMIP	0.7957	1.48E-04	3.45E-04	-0.2105
ENSG00000244038	DDOST	1.1610	1.48E-04	3.45E-04	-0.2105
ENSG00000187957	DNER	1.0244	1.48E-04	3.45E-04	-0.2116
ENSG00000183722	LHFPL6	1.2007	1.48E-04	3.46E-04	-0.2144
ENSG00000205639	MFSD2B	1.0978	1.49E-04	3.47E-04	-0.2164
ENSG00000137033	IL33	1.1337	1.50E-04	3.49E-04	-0.2228
ENSG00000130590	SAMD10	1.1878	1.50E-04	3.50E-04	-0.2265
ENSG00000158201	ABHD3	1.2288	1.51E-04	3.51E-04	-0.2283
ENSG00000108688	CCL7	1.1307	1.51E-04	3.52E-04	-0.2303
ENSG00000169223	LMAN2	0.8169	1.51E-04	3.52E-04	-0.2319
ENSG00000182504	CEP97	1.2644	1.51E-04	3.53E-04	-0.2331
ENSG00000186081	KRT5	1.1689	1.52E-04	3.54E-04	-0.2360
ENSG00000187105	HEATR4	1.0925	1.52E-04	3.54E-04	-0.2379
ENSG00000167994	RAB3IL1	0.7977	1.52E-04	3.54E-04	-0.2385
ENSG00000099834	CDHR5	1.0733	1.52E-04	3.55E-04	-0.2388
ENSG00000159409	CELF3	1.0254	1.52E-04	3.55E-04	-0.2403
ENSG00000151304	SRFBP1	0.8122	1.53E-04	3.56E-04	-0.2437
ENSG00000162576	MXRA8	1.3243	1.53E-04	3.57E-04	-0.2444
ENSG00000100601	ALKBH1	1.1445	1.53E-04	3.57E-04	-0.2448
ENSG00000110077	MS4A6A	0.6215	1.53E-04	3.57E-04	-0.2447
ENSG00000095777	MYO3A	1.0791	1.53E-04	3.57E-04	-0.2449
ENSG00000130313	PGLS	0.8839	1.53E-04	3.57E-04	-0.2454
ENSG00000136535	TBR1	1.0386	1.53E-04	3.57E-04	-0.2455
ENSG00000120805	ARL1	1.1711	1.53E-04	3.57E-04	-0.2457
ENSG00000176641	RNF152	1.0713	1.54E-04	3.58E-04	-0.2481
ENSG00000172361	CFAP53	1.1352	1.55E-04	3.59E-04	-0.2530
ENSG00000173264	GPR137	1.1659	1.55E-04	3.60E-04	-0.2543
ENSG00000176055	MBLAC2	0.8147	1.55E-04	3.62E-04	-0.2588
ENSG00000124920	MYRF	1.2208	1.56E-04	3.63E-04	-0.2629
ENSG00000204084	INPP5B	1.2177	1.57E-04	3.66E-04	-0.2709
ENSG00000147180	ZNF711	1.4254	1.58E-04	3.67E-04	-0.2724
ENSG00000114487	MORC1	0.6846	1.58E-04	3.67E-04	-0.2738

Gene ID	Gene Symbol	FC	P.Value	adj.P.Val	B
ENSG00000112290	WASF1	1.1900	1.58E-04	3.67E-04	-0.2742
ENSG00000196659	TTC30B	1.1804	1.58E-04	3.67E-04	-0.2742
ENSG00000129493	HEATR5A	1.1579	1.58E-04	3.68E-04	-0.2765
ENSG00000173898	SPTBN2	0.6789	1.59E-04	3.69E-04	-0.2792
ENSG00000108375	RNF43	1.1118	1.60E-04	3.71E-04	-0.2831
ENSG00000119977	TCTN3	0.8070	1.60E-04	3.71E-04	-0.2840
ENSG00000004399	PLXND1	1.4379	1.61E-04	3.74E-04	-0.2915
ENSG00000007255	TRAPPC6A	0.8466	1.61E-04	3.74E-04	-0.2930
ENSG00000076716	GPC4	1.1350	1.62E-04	3.75E-04	-0.2947
ENSG00000244005	NFS1	0.8091	1.62E-04	3.75E-04	-0.2953
ENSG00000106351	AGFG2	0.8105	1.62E-04	3.76E-04	-0.2970
ENSG00000179241	LDLRAD3	1.0616	1.62E-04	3.76E-04	-0.2972
ENSG00000198771	RCSD1	1.2595	1.62E-04	3.76E-04	-0.2974
ENSG00000204308	RNF5	0.8524	1.62E-04	3.76E-04	-0.2985
ENSG00000129474	AJUBA	0.8888	1.62E-04	3.76E-04	-0.2989
ENSG00000081985	IL12RB2	1.1924	1.63E-04	3.77E-04	-0.3015
ENSG00000104231	ZFAND1	0.8812	1.64E-04	3.80E-04	-0.3071
ENSG00000131375	CAPN7	1.1432	1.64E-04	3.80E-04	-0.3079
ENSG00000100744	GSKIP	1.1633	1.64E-04	3.80E-04	-0.3086
ENSG00000182676	PPP1R27	1.0917	1.64E-04	3.80E-04	-0.3086
ENSG00000145649	GZMA	0.8141	1.65E-04	3.82E-04	-0.3142
ENSG00000086159	AQP6	1.0110	1.65E-04	3.83E-04	-0.3149
ENSG00000143061	IGSF3	1.0462	1.66E-04	3.84E-04	-0.3186
ENSG00000103254	ANTKMT	0.8559	1.66E-04	3.84E-04	-0.3192
ENSG00000262481	TMEM256-PLSCR3	1.0835	1.67E-04	3.86E-04	-0.3236
ENSG00000165995	CACNB2	1.0919	1.67E-04	3.87E-04	-0.3253
ENSG00000106266	SNX8	1.1961	1.67E-04	3.87E-04	-0.3256
ENSG00000197165	SULT1A2	1.2077	1.67E-04	3.87E-04	-0.3267
ENSG00000157617	C2CD2	1.2529	1.68E-04	3.88E-04	-0.3302
ENSG00000112773	TENT5A	1.3724	1.68E-04	3.89E-04	-0.3308
ENSG00000168309	FAM107A	1.0432	1.68E-04	3.90E-04	-0.3333
ENSG00000089127	OAS1	0.7234	1.68E-04	3.90E-04	-0.3338
ENSG00000184194	GPR173	1.1509	1.68E-04	3.90E-04	-0.3344
ENSG00000118898	PPL	1.1399	1.69E-04	3.91E-04	-0.3360
ENSG00000051825	MPHOSPH9	1.1976	1.69E-04	3.91E-04	-0.3362
ENSG00000155066	PROM2	1.0667	1.69E-04	3.91E-04	-0.3369
ENSG00000088827	SIGLEC1	0.7504	1.69E-04	3.91E-04	-0.3374
ENSG00000260286	ARMH2	1.0280	1.69E-04	3.91E-04	-0.3384
ENSG00000012211	PRICKLE3	1.1598	1.70E-04	3.94E-04	-0.3437
ENSG00000241106	HLA-DOB	0.7390	1.70E-04	3.94E-04	-0.3439
ENSG00000205268	PDE7A	1.3186	1.71E-04	3.95E-04	-0.3466
ENSG00000128694	OSGEPL1	0.8004	1.71E-04	3.96E-04	-0.3502
ENSG00000123219	CENPK	1.3041	1.71E-04	3.97E-04	-0.3511
ENSG00000213654	GPSM3	1.2129	1.72E-04	3.98E-04	-0.3540
ENSG00000171956	FOXB1	1.0466	1.72E-04	3.98E-04	-0.3544
ENSG00000140691	ARMC5	1.1803	1.72E-04	3.98E-04	-0.3554
ENSG00000160179	ABCG1	1.2959	1.73E-04	3.99E-04	-0.3568
ENSG00000006625	GGCT	0.8177	1.73E-04	4.00E-04	-0.3609
ENSG00000122692	SMU1	1.1278	1.74E-04	4.03E-04	-0.3673
ENSG00000130054	NALF2	1.0354	1.75E-04	4.04E-04	-0.3687
ENSG00000172890	NADSYN1	1.1670	1.75E-04	4.04E-04	-0.3706
ENSG00000188868	ZNF563	0.8777	1.75E-04	4.05E-04	-0.3710
ENSG00000152495	CAMK4	1.2089	1.75E-04	4.05E-04	-0.3712
ENSG00000106355	LSM5	0.8679	1.75E-04	4.05E-04	-0.3717

Gene ID	Gene Symbol	FC	P.Value	adj.P.Val	B
ENSG00000115525	ST3GAL5	0.7982	1.76E-04	4.06E-04	-0.3755
ENSG00000026652	AGPAT4	1.2509	1.77E-04	4.08E-04	-0.3789
ENSG00000108352	RAPGEFL1	1.1660	1.77E-04	4.09E-04	-0.3820
ENSG00000135049	AGTPBP1	1.1356	1.77E-04	4.10E-04	-0.3833
ENSG00000108798	ABI3	0.8231	1.78E-04	4.10E-04	-0.3850
ENSG00000138646	HERC5	1.3151	1.78E-04	4.11E-04	-0.3865
ENSG00000186001	LRCH3	1.1840	1.78E-04	4.12E-04	-0.3883
ENSG00000198780	FAM169A	1.2810	1.79E-04	4.14E-04	-0.3935
ENSG00000145863	GABRA6	1.0693	1.80E-04	4.14E-04	-0.3946
ENSG00000179085	DPM3	1.2731	1.81E-04	4.16E-04	-0.3996
ENSG00000124701	APOBEC2	1.1116	1.81E-04	4.17E-04	-0.4019
ENSG00000089050	RBBP9	1.2091	1.81E-04	4.18E-04	-0.4034
ENSG00000135750	KCNK1	1.1589	1.81E-04	4.18E-04	-0.4042
ENSG00000174840	PDE12	1.1501	1.82E-04	4.19E-04	-0.4054
ENSG00000198062	POTEH	1.0603	1.82E-04	4.20E-04	-0.4074
ENSG00000080572	DNAAF6	1.0292	1.82E-04	4.20E-04	-0.4086
ENSG00000150773	PIH1D2	1.0964	1.82E-04	4.20E-04	-0.4093
ENSG00000163644	PPM1K	1.2259	1.83E-04	4.21E-04	-0.4103
ENSG00000188807	TMEM201	1.1583	1.83E-04	4.22E-04	-0.4122
ENSG00000160410	SHKBP1	1.1540	1.83E-04	4.22E-04	-0.4137
ENSG00000177409	SAMD9L	0.7062	1.84E-04	4.23E-04	-0.4162
ENSG00000204950	LRRC10B	1.0440	1.84E-04	4.24E-04	-0.4185
ENSG00000158109	TPRG1L	1.1711	1.84E-04	4.25E-04	-0.4197
ENSG00000225663	MCRIP1	1.1659	1.85E-04	4.26E-04	-0.4220
ENSG00000138650	PCDH10	1.1095	1.85E-04	4.26E-04	-0.4234
ENSG00000114547	ROPN1B	1.1008	1.86E-04	4.27E-04	-0.4258
ENSG00000156787	TBC1D31	1.1874	1.87E-04	4.30E-04	-0.4319
ENSG00000172425	TTC36	1.1290	1.87E-04	4.30E-04	-0.4326
ENSG00000031698	SARS1	1.1926	1.87E-04	4.31E-04	-0.4334
ENSG00000126067	PSMB2	1.1449	1.87E-04	4.31E-04	-0.4349
ENSG00000160799	CCDC12	1.1269	1.88E-04	4.32E-04	-0.4370
ENSG00000197417	SHPK	1.1488	1.89E-04	4.34E-04	-0.4410
ENSG00000159363	ATP13A2	1.2693	1.89E-04	4.36E-04	-0.4448
ENSG00000088367	EPB41L1	1.3068	1.89E-04	4.36E-04	-0.4450
ENSG00000254535	PABPC4L	1.1015	1.90E-04	4.37E-04	-0.4474
ENSG00000185760	KCNQ5	1.2476	1.91E-04	4.38E-04	-0.4502
ENSG00000187210	GCNT1	0.7318	1.91E-04	4.38E-04	-0.4508
ENSG00000108604	SMARCD2	1.1427	1.91E-04	4.39E-04	-0.4515
ENSG00000130734	ATG4D	1.1540	1.91E-04	4.39E-04	-0.4522
ENSG00000100429	HDAC10	1.2296	1.93E-04	4.43E-04	-0.4608
ENSG00000254788	CKLF-CMTM1	1.1954	1.93E-04	4.44E-04	-0.4626
ENSG00000188011	RTP5	1.0243	1.93E-04	4.44E-04	-0.4640
ENSG00000077522	ACTN2	1.0673	1.94E-04	4.46E-04	-0.4678
ENSG00000102471	NDFIP2	0.8414	1.94E-04	4.47E-04	-0.4692
ENSG00000173467	AGR3	1.0601	1.95E-04	4.47E-04	-0.4704
ENSG00000129151	BBOX1	0.6220	1.95E-04	4.47E-04	-0.4710
ENSG00000134255	CEPT1	0.8321	1.95E-04	4.48E-04	-0.4724
ENSG00000166407	LMO1	1.0555	1.95E-04	4.48E-04	-0.4728
ENSG00000203963	C1orf141	1.0881	1.95E-04	4.48E-04	-0.4729
ENSG00000175602	CCDC85B	0.8132	1.96E-04	4.49E-04	-0.4744
ENSG00000173930	SLCO4C1	1.1917	1.96E-04	4.49E-04	-0.4747
ENSG00000172403	SYNPO2	0.8533	1.96E-04	4.50E-04	-0.4766
ENSG00000140463	BBS4	1.1689	1.96E-04	4.50E-04	-0.4768
ENSG00000121390	PSPC1	0.8434	1.97E-04	4.51E-04	-0.4794

Gene ID	Gene Symbol	FC	P.Value	adj.P.Val	B
ENSG00000114054	PCCB	0.8182	1.97E-04	4.51E-04	-0.4802
ENSG00000162669	HFM1	1.1260	1.97E-04	4.51E-04	-0.4804
ENSG00000144460	NYAP2	0.8387	1.97E-04	4.52E-04	-0.4829
ENSG00000137642	SORL1	1.3708	1.98E-04	4.54E-04	-0.4862
ENSG00000185823	NPAP1	1.0717	1.98E-04	4.54E-04	-0.4868
ENSG00000165672	PRDX3	0.8009	1.99E-04	4.56E-04	-0.4908
ENSG00000155008	APOOL	1.1975	2.00E-04	4.59E-04	-0.4967
ENSG00000130560	UBAC1	0.8682	2.00E-04	4.59E-04	-0.4970
ENSG00000133401	PDZD2	1.0846	2.00E-04	4.59E-04	-0.4970
ENSG00000135414	GDF11	1.2432	2.01E-04	4.60E-04	-0.4987
ENSG00000187323	DCC	1.4751	2.01E-04	4.60E-04	-0.4994
ENSG00000163126	ANKRD23	1.2221	2.01E-04	4.60E-04	-0.5003
ENSG00000175198	PCCA	0.8587	2.01E-04	4.61E-04	-0.5011
ENSG00000070718	AP3M2	1.2027	2.01E-04	4.61E-04	-0.5024
ENSG00000164074	ABHD18	1.1392	2.02E-04	4.62E-04	-0.5037
ENSG00000168925	CTRB1	1.0553	2.02E-04	4.62E-04	-0.5052
ENSG00000127124	HIVEP3	1.1562	2.03E-04	4.64E-04	-0.5088
ENSG00000185236	RAB11B	1.1258	2.03E-04	4.64E-04	-0.5093
ENSG00000198169	ZNF251	1.1860	2.03E-04	4.66E-04	-0.5118
ENSG00000185662	SMIM23	0.8581	2.04E-04	4.66E-04	-0.5128
ENSG00000159842	ABR	1.4096	2.04E-04	4.67E-04	-0.5143
ENSG00000091436	MAP3K20	1.2257	2.05E-04	4.70E-04	-0.5201
ENSG00000168447	SCNN1B	1.4738	2.06E-04	4.71E-04	-0.5231
ENSG00000017260	ATP2C1	1.1611	2.06E-04	4.72E-04	-0.5251
ENSG00000147403	RPL10	0.8453	2.07E-04	4.73E-04	-0.5266
ENSG00000213694	S1PR3	1.0960	2.07E-04	4.73E-04	-0.5280
ENSG00000154451	GBP5	1.5711	2.08E-04	4.77E-04	-0.5345
ENSG00000184956	MUC6	1.0502	2.09E-04	4.77E-04	-0.5358
ENSG00000103202	NME4	0.7676	2.09E-04	4.78E-04	-0.5372
ENSG00000175928	LRRN1	0.7139	2.09E-04	4.78E-04	-0.5385
ENSG00000163634	THOC7	0.8723	2.09E-04	4.78E-04	-0.5388
ENSG00000177627	C12orf54	1.0398	2.09E-04	4.79E-04	-0.5390
ENSG00000168268	NT5DC2	1.4367	2.10E-04	4.79E-04	-0.5396
ENSG00000136783	NIPSNAP3A	0.8453	2.10E-04	4.79E-04	-0.5397
ENSG00000132470	ITGB4	1.2259	2.10E-04	4.79E-04	-0.5409
ENSG00000173226	IQCB1	0.8288	2.10E-04	4.80E-04	-0.5433
ENSG00000116171	SCP2	0.8470	2.11E-04	4.82E-04	-0.5468
ENSG00000186638	KIF24	1.1491	2.13E-04	4.86E-04	-0.5539
ENSG00000124134	KCNS1	1.0490	2.13E-04	4.86E-04	-0.5544
ENSG00000170291	ELP5	1.1578	2.13E-04	4.86E-04	-0.5546
ENSG00000126016	AMOT	1.2136	2.14E-04	4.88E-04	-0.5582
ENSG00000108684	ASIC2	1.0181	2.14E-04	4.88E-04	-0.5587
ENSG00000174939	ASPHD1	1.2302	2.14E-04	4.88E-04	-0.5594
ENSG00000107438	PDLIM1	1.5126	2.14E-04	4.89E-04	-0.5606
ENSG00000184378	ACTRT3	0.9503	2.14E-04	4.89E-04	-0.5608
ENSG00000187942	LDLRAD2	0.6988	2.15E-04	4.91E-04	-0.5655
ENSG00000137857	DUOX1	1.2231	2.16E-04	4.92E-04	-0.5677
ENSG00000176769	TCERG1L	1.0609	2.16E-04	4.93E-04	-0.5680
ENSG00000001630	CYP51A1	1.1797	2.16E-04	4.93E-04	-0.5698
ENSG00000026508	CD44	1.9571	2.17E-04	4.94E-04	-0.5711
ENSG00000170615	SLC26A5	1.1679	2.17E-04	4.94E-04	-0.5716
ENSG00000128652	HOXD3	1.0789	2.17E-04	4.95E-04	-0.5723
ENSG00000169410	PTPN9	1.1626	2.17E-04	4.95E-04	-0.5725
ENSG00000205704	LINC00634	1.0815	2.18E-04	4.96E-04	-0.5755

Gene ID	Gene Symbol	FC	P.Value	adj.P.Val	B
ENSG00000132704	FCRL2	1.6582	2.18E-04	4.96E-04	-0.5757
ENSG00000187166	H1-7	1.0811	2.18E-04	4.97E-04	-0.5776
ENSG00000130202	NECTIN2	1.2695	2.18E-04	4.98E-04	-0.5784
ENSG00000112697	TMEM30A	1.1770	2.19E-04	4.98E-04	-0.5797
ENSG00000162927	PUS10	1.1413	2.19E-04	4.99E-04	-0.5816
ENSG00000166143	PPP1R14D	0.7825	2.19E-04	4.99E-04	-0.5816
ENSG00000240563	L1TD1	1.0292	2.19E-04	4.99E-04	-0.5817
ENSG00000090376	IRAK3	1.3004	2.21E-04	5.02E-04	-0.5880
ENSG00000196782	MAML3	1.1200	2.21E-04	5.02E-04	-0.5884
ENSG00000164574	GALNT10	1.2152	2.21E-04	5.03E-04	-0.5890
ENSG00000145882	PCYOX1L	1.2864	2.21E-04	5.03E-04	-0.5904
ENSG00000171723	GPHN	1.2090	2.21E-04	5.04E-04	-0.5913
ENSG00000166482	MFAP4	1.2705	2.22E-04	5.05E-04	-0.5936
ENSG00000129595	EPB41L4A	0.7943	2.22E-04	5.05E-04	-0.5940
ENSG00000079101	CLUL1	1.0886	2.22E-04	5.06E-04	-0.5949
ENSG00000254093	PINX1	1.2148	2.23E-04	5.07E-04	-0.5969
ENSG00000120832	MTERF2	1.1508	2.23E-04	5.07E-04	-0.5974
ENSG00000124116	WFDC3	1.1188	2.23E-04	5.07E-04	-0.5974
ENSG00000047346	FAM214A	1.2456	2.23E-04	5.07E-04	-0.5978
ENSG00000188725	SMIM15	0.8431	2.24E-04	5.09E-04	-0.6015
ENSG00000113719	ERGIC1	1.1668	2.24E-04	5.09E-04	-0.6021
ENSG00000154783	FGD5	1.0488	2.24E-04	5.09E-04	-0.6024
ENSG00000243649	CFB	1.4057	2.25E-04	5.12E-04	-0.6077
ENSG00000137040	RANBP6	1.1746	2.26E-04	5.13E-04	-0.6092
ENSG00000146955	RAB19	0.9292	2.26E-04	5.13E-04	-0.6097
ENSG00000134363	FST	1.2024	2.27E-04	5.16E-04	-0.6154
ENSG00000188152	NUTM2G	1.0686	2.28E-04	5.18E-04	-0.6193
ENSG00000110427	KIAA1549L	1.1816	2.29E-04	5.19E-04	-0.6213
ENSG00000048649	RSF1	1.1733	2.29E-04	5.20E-04	-0.6228
ENSG00000186334	SLC36A3	1.0310	2.29E-04	5.21E-04	-0.6247
ENSG00000105254	TBCB	1.1450	2.31E-04	5.23E-04	-0.6293
ENSG00000162981	LRATD1	1.1028	2.31E-04	5.23E-04	-0.6294
ENSG00000165125	TRPV6	1.0330	2.31E-04	5.23E-04	-0.6294
ENSG00000188130	MAPK12	1.4023	2.32E-04	5.27E-04	-0.6363
ENSG00000175395	ZNF25	1.1584	2.33E-04	5.30E-04	-0.6408
ENSG00000179855	GIPC3	1.1711	2.34E-04	5.30E-04	-0.6412
ENSG00000162426	SLC45A1	1.0628	2.34E-04	5.30E-04	-0.6415
ENSG00000158987	RAPGEF6	1.1690	2.34E-04	5.30E-04	-0.6425
ENSG00000135185	TMEM243	0.8483	2.34E-04	5.31E-04	-0.6434
ENSG00000114854	TNNC1	0.8375	2.34E-04	5.31E-04	-0.6435
ENSG00000105516	DBP	0.8081	2.34E-04	5.32E-04	-0.6451
ENSG00000196427	NBPF4	0.7158	2.35E-04	5.32E-04	-0.6452
ENSG00000113407	TARS1	0.8387	2.35E-04	5.32E-04	-0.6458
ENSG00000166825	ANPEP	1.4164	2.35E-04	5.33E-04	-0.6483
ENSG00000140015	KCNH5	1.0481	2.36E-04	5.34E-04	-0.6497
ENSG00000161610	HCRT	1.1089	2.36E-04	5.35E-04	-0.6518
ENSG00000133687	TMTC1	1.2069	2.36E-04	5.35E-04	-0.6519
ENSG00000170482	SLC23A1	0.7663	2.37E-04	5.36E-04	-0.6543
ENSG00000134874	DZIP1	1.0460	2.37E-04	5.37E-04	-0.6550
ENSG00000234602	MCIDAS	1.0410	2.38E-04	5.38E-04	-0.6576
ENSG00000145687	SSBP2	1.2431	2.38E-04	5.39E-04	-0.6586
ENSG00000159189	C1QC	0.5599	2.38E-04	5.39E-04	-0.6586
ENSG00000071189	SNX13	1.1522	2.38E-04	5.39E-04	-0.6595
ENSG00000186469	GNG2	1.4218	2.38E-04	5.40E-04	-0.6608

Gene ID	Gene Symbol	FC	P.Value	adj.P.Val	B
ENSG00000100647	SUSD6	1.2016	2.39E-04	5.42E-04	-0.6648
ENSG00000006740	ARHGAP44	1.4165	2.40E-04	5.43E-04	-0.6671
ENSG00000219545	UMAD1	0.8558	2.40E-04	5.44E-04	-0.6677
ENSG00000089199	CHGB	1.0497	2.40E-04	5.44E-04	-0.6680
ENSG00000117411	B4GALT2	1.2020	2.42E-04	5.47E-04	-0.6737
ENSG00000148942	SLC5A12	1.1689	2.42E-04	5.47E-04	-0.6745
ENSG00000170921	TANC2	1.2565	2.42E-04	5.47E-04	-0.6747
ENSG00000082014	SMARCD3	0.7849	2.42E-04	5.48E-04	-0.6750
ENSG00000119915	ELOVL3	1.2426	2.43E-04	5.49E-04	-0.6779
ENSG00000157570	TSPAN18	1.1040	2.43E-04	5.50E-04	-0.6803
ENSG00000186184	POLR1D	0.8674	2.44E-04	5.51E-04	-0.6806
ENSG00000123545	NDUFAF4	0.8379	2.44E-04	5.52E-04	-0.6829
ENSG00000105357	MYH14	1.1065	2.44E-04	5.52E-04	-0.6833
ENSG00000152484	USP12	1.2642	2.45E-04	5.54E-04	-0.6873
ENSG00000119283	TRIM67	1.0170	2.46E-04	5.55E-04	-0.6883
ENSG00000104524	PYCR3	0.8231	2.46E-04	5.56E-04	-0.6903
ENSG00000131015	ULBP2	1.0701	2.46E-04	5.56E-04	-0.6907
ENSG00000007062	PROM1	1.0963	2.47E-04	5.57E-04	-0.6924
ENSG00000171159	BBLN	0.8780	2.47E-04	5.57E-04	-0.6925
ENSG00000169429	CXCL8	1.8832	2.47E-04	5.58E-04	-0.6934
ENSG00000197498	RPF2	0.8321	2.47E-04	5.58E-04	-0.6937
ENSG00000148057	IDNK	0.8445	2.47E-04	5.58E-04	-0.6946
ENSG00000146282	RARS2	1.1521	2.48E-04	5.59E-04	-0.6966
ENSG00000184345	IQCF2	1.0169	2.48E-04	5.59E-04	-0.6969
ENSG00000176101	SSNA1	0.8788	2.48E-04	5.60E-04	-0.6980
ENSG00000148377	IDI2	1.0334	2.48E-04	5.60E-04	-0.6983
ENSG00000205277	MUC12	1.2929	2.48E-04	5.60E-04	-0.6988
ENSG00000170955	CAVIN3	1.2590	2.49E-04	5.61E-04	-0.7000
ENSG00000215009	ACSM4	1.1020	2.49E-04	5.61E-04	-0.7003
ENSG00000168772	CXXC4	1.2984	2.49E-04	5.61E-04	-0.7005
ENSG00000105698	USF2	1.1314	2.49E-04	5.61E-04	-0.7008
ENSG00000172819	RARG	1.2057	2.50E-04	5.63E-04	-0.7041
ENSG00000113494	PRLR	1.0749	2.50E-04	5.63E-04	-0.7044
ENSG00000144320	LNPK	1.1914	2.50E-04	5.65E-04	-0.7067
ENSG00000158458	NRG2	0.6865	2.50E-04	5.65E-04	-0.7068
ENSG00000197037	ZSCAN25	1.1507	2.51E-04	5.66E-04	-0.7095
ENSG00000109576	AADAT	1.0543	2.52E-04	5.68E-04	-0.7135
ENSG00000164180	TMEM161B	0.8681	2.53E-04	5.70E-04	-0.7157
ENSG00000163312	HELQ	1.1455	2.53E-04	5.70E-04	-0.7161
ENSG00000268041	ERFL	0.6889	2.53E-04	5.70E-04	-0.7164
ENSG00000203740	NTMT2	1.0709	2.53E-04	5.70E-04	-0.7167
ENSG00000077420	APBB1IP	1.4414	2.53E-04	5.70E-04	-0.7170
ENSG00000197992	CLEC9A	1.3166	2.53E-04	5.70E-04	-0.7173
ENSG00000236334	PPIAL4G	1.0965	2.54E-04	5.72E-04	-0.7196
ENSG00000060642	PIGV	0.8171	2.55E-04	5.75E-04	-0.7252
ENSG00000133027	PEMT	1.1783	2.55E-04	5.75E-04	-0.7255
ENSG00000109089	CDR2L	1.0425	2.56E-04	5.77E-04	-0.7281
ENSG00000197253	TPSB2	1.0744	2.56E-04	5.77E-04	-0.7281
ENSG00000140481	CCDC33	0.9813	2.56E-04	5.77E-04	-0.7290
ENSG00000256269	HMBS	0.8303	2.57E-04	5.78E-04	-0.7303
ENSG00000232434	AJM1	1.1747	2.57E-04	5.79E-04	-0.7319
ENSG00000156966	B3GNT7	1.1150	2.58E-04	5.80E-04	-0.7334
ENSG00000084764	MAPRE3	1.2193	2.58E-04	5.80E-04	-0.7348
ENSG00000168291	PDHB	0.8546	2.58E-04	5.81E-04	-0.7351

Gene ID	Gene Symbol	FC	P.Value	adj.P.Val	B
ENSG00000049759	NEDD4L	1.4128	2.59E-04	5.81E-04	-0.7366
ENSG00000184154	LRRC51	1.1659	2.59E-04	5.82E-04	-0.7377
ENSG00000139973	SYT16	1.0211	2.60E-04	5.85E-04	-0.7430
ENSG00000107018	RLN1	0.8942	2.60E-04	5.86E-04	-0.7436
ENSG00000171496	OR1L8	0.9617	2.61E-04	5.86E-04	-0.7442
ENSG00000184557	SOCS3	1.8709	2.61E-04	5.86E-04	-0.7452
ENSG00000127129	EDN2	1.0562	2.62E-04	5.88E-04	-0.7476
ENSG00000141639	MAPK4	1.0112	2.62E-04	5.88E-04	-0.7483
ENSG00000152592	DMP1	1.0921	2.62E-04	5.89E-04	-0.7490
ENSG00000089876	DHX32	1.2619	2.62E-04	5.89E-04	-0.7492
ENSG00000175899	A2M	0.7080	2.63E-04	5.91E-04	-0.7530
ENSG00000167695	TLCD3A	1.2093	2.64E-04	5.93E-04	-0.7559
ENSG00000206052	DOK6	1.0735	2.64E-04	5.93E-04	-0.7573
ENSG00000108984	MAP2K6	1.2832	2.65E-04	5.95E-04	-0.7596
ENSG00000147434	CHRNA6	1.0529	2.65E-04	5.95E-04	-0.7602
ENSG00000144744	UBA3	1.1277	2.66E-04	5.97E-04	-0.7630
ENSG00000259120	SMIM6	1.2232	2.67E-04	6.00E-04	-0.7685
ENSG00000188283	ZNF383	1.1593	2.69E-04	6.03E-04	-0.7731
ENSG00000116752	BCAS2	1.1398	2.69E-04	6.04E-04	-0.7738
ENSG00000205336	ADGRG1	1.2996	2.69E-04	6.04E-04	-0.7738
ENSG00000185985	SLITRK2	1.2735	2.70E-04	6.05E-04	-0.7765
ENSG00000148204	CRB2	1.0723	2.71E-04	6.07E-04	-0.7794
ENSG00000154727	GABPA	1.1555	2.71E-04	6.07E-04	-0.7795
ENSG00000244242	IFITM10	1.2301	2.71E-04	6.08E-04	-0.7810
ENSG00000105649	RAB3A	0.8184	2.72E-04	6.09E-04	-0.7832
ENSG00000157542	KCNJ6	1.0268	2.72E-04	6.09E-04	-0.7832
ENSG00000205629	LCMT1	1.1006	2.72E-04	6.10E-04	-0.7843
ENSG00000120158	RCL1	1.1317	2.73E-04	6.11E-04	-0.7862
ENSG00000168209	DDIT4	1.4582	2.73E-04	6.12E-04	-0.7877
ENSG00000181541	MAB21L2	1.0075	2.74E-04	6.13E-04	-0.7899
ENSG00000115221	ITGB6	1.0746	2.74E-04	6.15E-04	-0.7920
ENSG00000108405	P2RX1	1.5856	2.74E-04	6.15E-04	-0.7924
ENSG00000139187	KLRG1	1.1619	2.75E-04	6.16E-04	-0.7943
ENSG00000136143	SUCLA2	0.8535	2.75E-04	6.17E-04	-0.7962
ENSG00000182484	WASH6P	1.1543	2.76E-04	6.18E-04	-0.7970
ENSG00000171462	DLK2	1.1186	2.77E-04	6.19E-04	-0.7998
ENSG00000122584	NXPH1	1.0490	2.77E-04	6.20E-04	-0.8012
ENSG00000131374	TBC1D5	1.2079	2.77E-04	6.21E-04	-0.8022
ENSG00000181744	DIPK2A	1.2551	2.77E-04	6.21E-04	-0.8028
ENSG00000060709	RIMBP2	1.1553	2.78E-04	6.22E-04	-0.8043
ENSG00000100726	TELO2	1.1937	2.79E-04	6.23E-04	-0.8066
ENSG00000178188	SH2B1	1.1527	2.79E-04	6.24E-04	-0.8073
ENSG00000100652	SLC10A1	1.0469	2.79E-04	6.25E-04	-0.8097
ENSG00000021645	NRXN3	1.0679	2.80E-04	6.25E-04	-0.8099
ENSG00000132622	HSPA12B	1.0881	2.80E-04	6.26E-04	-0.8103
ENSG00000160593	JAML	0.7182	2.80E-04	6.26E-04	-0.8103
ENSG00000203730	TEDDM1	1.0307	2.80E-04	6.26E-04	-0.8104
ENSG00000203805	PLPP4	1.1039	2.80E-04	6.26E-04	-0.8115
ENSG00000151348	EXT2	0.8451	2.81E-04	6.28E-04	-0.8147
ENSG00000104129	DNAJC17	0.8648	2.81E-04	6.29E-04	-0.8155
ENSG00000138496	PARP9	0.7608	2.81E-04	6.29E-04	-0.8158
ENSG00000049883	PTCD2	0.8133	2.81E-04	6.29E-04	-0.8164
ENSG00000178997	EXD1	1.0610	2.82E-04	6.31E-04	-0.8186
ENSG00000175354	PTPN2	1.1446	2.83E-04	6.32E-04	-0.8209

Gene ID	Gene Symbol	FC	P.Value	adj.P.Val	B
ENSG00000155324	GRAMD2B	1.3523	2.83E-04	6.33E-04	-0.8224
ENSG00000255823	MTRNR2L8	1.3268	2.84E-04	6.36E-04	-0.8262
ENSG00000142627	EPHA2	1.0900	2.85E-04	6.37E-04	-0.8280
ENSG00000135678	CPM	1.1832	2.86E-04	6.38E-04	-0.8301
ENSG00000166669	ATF7IP2	1.3299	2.86E-04	6.38E-04	-0.8308
ENSG00000106648	GALNTL5	1.0246	2.86E-04	6.40E-04	-0.8325
ENSG00000213619	NDUFS3	1.1043	2.87E-04	6.41E-04	-0.8344
ENSG00000141127	PRPSAP2	1.2035	2.88E-04	6.42E-04	-0.8362
ENSG00000165443	PHYHIPL	1.0708	2.88E-04	6.42E-04	-0.8371
ENSG00000100523	DDHD1	1.2038	2.89E-04	6.45E-04	-0.8411
ENSG00000169718	DUS1L	1.1408	2.90E-04	6.48E-04	-0.8451
ENSG00000214253	FIS1	0.9004	2.91E-04	6.50E-04	-0.8482
ENSG00000177239	MAN1B1	0.8722	2.91E-04	6.50E-04	-0.8491
ENSG00000146540	C7orf50	0.8416	2.92E-04	6.50E-04	-0.8492
ENSG00000126934	MAP2K2	0.8888	2.92E-04	6.52E-04	-0.8512
ENSG00000121060	TRIM25	1.2369	2.94E-04	6.55E-04	-0.8556
ENSG00000164265	SCGB3A2	1.3021	2.95E-04	6.58E-04	-0.8602
ENSG00000197191	CYSRT1	0.8738	2.95E-04	6.59E-04	-0.8619
ENSG00000123643	SLC36A1	1.1659	2.96E-04	6.59E-04	-0.8622
ENSG00000162779	AXDND1	1.1309	2.97E-04	6.63E-04	-0.8673
ENSG00000149499	EML3	1.1564	2.97E-04	6.63E-04	-0.8675
ENSG00000188269	OR7A5	1.2080	2.98E-04	6.64E-04	-0.8699
ENSG00000144671	SLC22A14	0.9583	2.99E-04	6.66E-04	-0.8726
ENSG00000102178	UBL4A	1.1823	2.99E-04	6.66E-04	-0.8732
ENSG00000132405	TBC1D14	1.1868	2.99E-04	6.67E-04	-0.8739
ENSG00000104879	CKM	1.0940	3.00E-04	6.68E-04	-0.8757
ENSG00000076513	ANKRD13A	1.1904	3.00E-04	6.69E-04	-0.8765
ENSG00000174804	FZD4	1.2172	3.01E-04	6.70E-04	-0.8787
ENSG00000205143	ARID3C	0.9283	3.01E-04	6.71E-04	-0.8798
ENSG00000167014	TERB2	1.0549	3.02E-04	6.72E-04	-0.8818
ENSG00000107147	KCNT1	1.1785	3.03E-04	6.74E-04	-0.8844
ENSG00000176623	RMDN1	1.1729	3.03E-04	6.74E-04	-0.8845
ENSG00000129749	CHRNA10	0.9078	3.03E-04	6.75E-04	-0.8867
ENSG00000111215	PRR4	1.1421	3.04E-04	6.77E-04	-0.8891
ENSG00000127366	TAS2R5	0.8869	3.05E-04	6.78E-04	-0.8907
ENSG00000179526	SHARPIN	1.1436	3.07E-04	6.83E-04	-0.8973
ENSG00000175893	ZDHHC21	0.8154	3.07E-04	6.84E-04	-0.8983
ENSG00000196503	ARL9	1.0837	3.08E-04	6.86E-04	-0.9018
ENSG00000161677	JOSD2	0.8471	3.09E-04	6.87E-04	-0.9031
ENSG00000080511	RDH8	1.0697	3.10E-04	6.89E-04	-0.9060
ENSG00000010310	GIPR	1.4117	3.11E-04	6.91E-04	-0.9095
ENSG00000001629	ANKIB1	1.1612	3.11E-04	6.92E-04	-0.9101
ENSG00000197147	LRRC8B	1.1677	3.12E-04	6.93E-04	-0.9120
ENSG00000138442	WDR12	0.7746	3.13E-04	6.95E-04	-0.9143
ENSG00000181381	DDX60L	1.3170	3.14E-04	6.98E-04	-0.9183
ENSG00000188536	HBA2	0.5202	3.14E-04	6.99E-04	-0.9198
ENSG00000058453	CROCC	1.2350	3.15E-04	6.99E-04	-0.9204
ENSG00000168591	TMUB2	1.1306	3.16E-04	7.02E-04	-0.9243
ENSG00000148965	SAA4	0.9540	3.16E-04	7.02E-04	-0.9249
ENSG00000228570	NUTM2E	1.0590	3.16E-04	7.03E-04	-0.9256
ENSG00000129255	MPDU1	0.8580	3.17E-04	7.05E-04	-0.9288
ENSG00000042286	AIFM2	1.1975	3.17E-04	7.05E-04	-0.9289
ENSG00000170315	UBB	1.1841	3.17E-04	7.05E-04	-0.9291
ENSG00000170370	EMX2	1.0267	3.19E-04	7.08E-04	-0.9333

Gene ID	Gene Symbol	FC	P.Value	adj.P.Val	B
ENSG00000129472	RAB2B	1.1788	3.19E-04	7.09E-04	-0.9349
ENSG00000242221	PSG2	1.0662	3.20E-04	7.11E-04	-0.9369
ENSG00000109511	ANXA10	1.0380	3.22E-04	7.14E-04	-0.9417
ENSG00000227507	LTB	1.5314	3.23E-04	7.16E-04	-0.9440
ENSG00000048028	USP28	1.2047	3.23E-04	7.16E-04	-0.9442
ENSG00000165899	OTOGL	0.7653	3.24E-04	7.18E-04	-0.9473
ENSG00000170653	ATF7	1.1388	3.24E-04	7.19E-04	-0.9480
ENSG00000179008	C14orf39	1.2029	3.24E-04	7.19E-04	-0.9485
ENSG00000125247	TMTC4	1.1927	3.24E-04	7.19E-04	-0.9492
ENSG00000147650	LRP12	1.3372	3.24E-04	7.19E-04	-0.9492
ENSG00000269855	RNF225	1.0437	3.24E-04	7.20E-04	-0.9496
ENSG00000164483	SAMD3	0.7309	3.25E-04	7.20E-04	-0.9501
ENSG00000119471	HSDL2	0.8110	3.25E-04	7.21E-04	-0.9514
ENSG00000005108	THSD7A	0.8811	3.25E-04	7.21E-04	-0.9520
ENSG00000116586	LAMTOR2	1.1721	3.25E-04	7.21E-04	-0.9524
ENSG00000160207	HSF2BP	1.0708	3.26E-04	7.22E-04	-0.9533
ENSG00000091732	ZC3HC1	0.8365	3.26E-04	7.23E-04	-0.9541
ENSG00000136014	USP44	1.1332	3.26E-04	7.23E-04	-0.9545
ENSG00000110723	EXPH5	1.1042	3.26E-04	7.23E-04	-0.9550
ENSG00000143194	MAEL	1.0971	3.28E-04	7.26E-04	-0.9582
ENSG00000184911	DMRTC1B	1.2225	3.28E-04	7.26E-04	-0.9592
ENSG00000069966	GNB5	1.1642	3.29E-04	7.28E-04	-0.9613
ENSG00000179580	RNF151	1.0308	3.29E-04	7.28E-04	-0.9622
ENSG00000205089	CCNI2	1.3613	3.30E-04	7.30E-04	-0.9648
ENSG00000164163	ABCE1	1.1634	3.30E-04	7.31E-04	-0.9658
ENSG00000125454	SLC25A19	0.7867	3.30E-04	7.31E-04	-0.9662
ENSG00000144659	SLC25A38	1.1222	3.31E-04	7.32E-04	-0.9671
ENSG00000173401	GLIPR1L1	1.1745	3.33E-04	7.37E-04	-0.9732
ENSG00000117500	TMED5	1.1797	3.34E-04	7.39E-04	-0.9761
ENSG00000164050	PLXNB1	1.3961	3.35E-04	7.41E-04	-0.9796
ENSG00000105355	PLIN3	1.1503	3.35E-04	7.42E-04	-0.9803
ENSG00000145832	SLC25A48	1.0624	3.35E-04	7.42E-04	-0.9806
ENSG00000132463	GRSF1	1.1197	3.36E-04	7.42E-04	-0.9813
ENSG00000177706	FAM20C	1.2092	3.36E-04	7.42E-04	-0.9813
ENSG00000126549	STATH	1.0621	3.38E-04	7.47E-04	-0.9877
ENSG00000134138	MEIS2	0.5910	3.38E-04	7.48E-04	-0.9887
ENSG00000081791	DELE1	0.8679	3.39E-04	7.50E-04	-0.9906
ENSG00000123297	TSFM	1.1722	3.39E-04	7.50E-04	-0.9914
ENSG00000196724	ZNF418	1.2546	3.39E-04	7.50E-04	-0.9914
ENSG00000139687	RB1	0.7664	3.40E-04	7.51E-04	-0.9928
ENSG00000107679	PLEKHA1	1.3607	3.40E-04	7.51E-04	-0.9934
ENSG00000132541	RIDA	0.7923	3.40E-04	7.52E-04	-0.9941
ENSG00000198033	TUBA3C	1.2024	3.41E-04	7.53E-04	-0.9953
ENSG00000166845	C18orf54	1.1721	3.41E-04	7.54E-04	-0.9965
ENSG00000120324	PCDHB10	0.8750	3.41E-04	7.54E-04	-0.9969
ENSG00000152804	HHEX	1.2604	3.41E-04	7.54E-04	-0.9971
ENSG00000105647	PIK3R2	0.8233	3.42E-04	7.55E-04	-0.9985
ENSG00000091129	NRCAM	1.3229	3.43E-04	7.56E-04	-1.0003
ENSG00000183979	NPB	1.1456	3.43E-04	7.57E-04	-1.0007
ENSG00000167861	HID1	1.3051	3.43E-04	7.58E-04	-1.0020
ENSG00000168918	INPP5D	1.2457	3.43E-04	7.58E-04	-1.0022
ENSG00000159182	PRAC1	1.0456	3.44E-04	7.60E-04	-1.0055
ENSG00000163354	DCST2	1.1517	3.45E-04	7.60E-04	-1.0059
ENSG00000188038	NRN1L	1.0735	3.45E-04	7.61E-04	-1.0060

Gene ID	Gene Symbol	FC	P.Value	adj.P.Val	B
ENSG00000138483	CCDC54	0.9783	3.45E-04	7.62E-04	-1.0074
ENSG00000175279	CENPS	1.1951	3.46E-04	7.63E-04	-1.0091
ENSG00000203663	OR2L2	1.0182	3.46E-04	7.64E-04	-1.0101
ENSG00000119630	PGF	1.1532	3.46E-04	7.64E-04	-1.0109
ENSG00000172939	OXSR1	1.1443	3.47E-04	7.65E-04	-1.0115
ENSG00000056487	PHF21B	1.0393	3.47E-04	7.65E-04	-1.0119
ENSG00000259417	CTXND1	1.0338	3.47E-04	7.66E-04	-1.0134
ENSG00000101333	PLCB4	0.8300	3.49E-04	7.69E-04	-1.0170
ENSG00000091128	LAMB4	1.0591	3.50E-04	7.72E-04	-1.0215
ENSG00000145147	SLIT2	1.2426	3.51E-04	7.73E-04	-1.0218
ENSG00000166596	CFAP52	1.0625	3.51E-04	7.73E-04	-1.0223
ENSG00000105810	CDK6	1.4260	3.52E-04	7.75E-04	-1.0250
ENSG00000163138	PACRGL	1.1614	3.52E-04	7.76E-04	-1.0263
ENSG00000249709	ZNF564	1.1316	3.53E-04	7.77E-04	-1.0276
ENSG00000086289	EPDR1	1.3413	3.53E-04	7.77E-04	-1.0282
ENSG00000111249	CUX2	1.0925	3.53E-04	7.77E-04	-1.0283
ENSG00000270757	HSPE1-MOB4	0.7464	3.54E-04	7.79E-04	-1.0308
ENSG00000144821	MYH15	1.2600	3.54E-04	7.79E-04	-1.0309
ENSG00000153404	PLEKHG4B	1.3308	3.55E-04	7.83E-04	-1.0348
ENSG00000238243	OR2W3	1.1084	3.59E-04	7.89E-04	-1.0430
ENSG00000174429	ABRA	1.0386	3.59E-04	7.91E-04	-1.0452
ENSG00000117586	TNFSF4	1.2308	3.61E-04	7.95E-04	-1.0496
ENSG00000003989	SLC7A2	1.0950	3.63E-04	7.98E-04	-1.0537
ENSG00000124102	PI3	0.8561	3.63E-04	7.98E-04	-1.0539
ENSG00000105778	AVL9	1.1343	3.65E-04	8.03E-04	-1.0590
ENSG00000127533	F2RL3	1.1442	3.65E-04	8.03E-04	-1.0600
ENSG00000166681	BEX3	1.5621	3.65E-04	8.04E-04	-1.0607
ENSG00000171960	PPIH	1.1337	3.65E-04	8.04E-04	-1.0607
ENSG00000143458	GABPB2	1.2267	3.67E-04	8.08E-04	-1.0658
ENSG00000181778	TMEM252	1.1243	3.68E-04	8.08E-04	-1.0664
ENSG00000112759	SLC29A1	1.2230	3.70E-04	8.14E-04	-1.0735
ENSG00000148948	LRRC4C	1.1657	3.71E-04	8.16E-04	-1.0756
ENSG00000103528	SYT17	1.0949	3.72E-04	8.17E-04	-1.0765
ENSG00000198018	ENTPD7	1.1487	3.72E-04	8.17E-04	-1.0770
ENSG00000066468	FGFR2	1.1485	3.73E-04	8.19E-04	-1.0793
ENSG00000108528	SLC25A11	0.8261	3.73E-04	8.19E-04	-1.0795
ENSG00000154518	ATP5MC3	0.8716	3.73E-04	8.20E-04	-1.0804
ENSG00000111269	CREBL2	0.8456	3.74E-04	8.22E-04	-1.0830
ENSG00000008130	NADK	1.1619	3.75E-04	8.25E-04	-1.0859
ENSG00000124466	LYPD3	1.0712	3.76E-04	8.25E-04	-1.0863
ENSG00000175216	CKAP5	1.1966	3.76E-04	8.25E-04	-1.0869
ENSG00000117419	ERI3	1.1410	3.76E-04	8.26E-04	-1.0876
ENSG00000166181	API5	1.1508	3.76E-04	8.26E-04	-1.0875
ENSG00000123415	SMUG1	0.8498	3.77E-04	8.28E-04	-1.0909
ENSG00000134042	MRO	0.8857	3.80E-04	8.33E-04	-1.0965
ENSG00000176909	MAMSTR	0.9097	3.80E-04	8.34E-04	-1.0972
ENSG00000132207	SLX1A	1.1917	3.82E-04	8.38E-04	-1.1023
ENSG00000127472	PLA2G5	1.0264	3.83E-04	8.39E-04	-1.1036
ENSG00000149177	PTPRJ	1.3405	3.84E-04	8.44E-04	-1.1083
ENSG00000035141	FAM136A	1.1211	3.85E-04	8.45E-04	-1.1099
ENSG00000100304	TTLL12	0.8021	3.85E-04	8.45E-04	-1.1102
ENSG00000124678	TCP11	1.0885	3.85E-04	8.45E-04	-1.1104
ENSG00000171150	SOCS5	1.1627	3.86E-04	8.45E-04	-1.1108
ENSG00000135404	CD63	1.2490	3.86E-04	8.46E-04	-1.1119

Gene ID	Gene Symbol	FC	P.Value	adj.P.Val	B
ENSG00000205593	DENND6B	1.4763	3.86E-04	8.47E-04	-1.1123
ENSG00000106789	CORO2A	1.0928	3.88E-04	8.51E-04	-1.1175
ENSG00000110536	PTPMT1	0.8923	3.88E-04	8.51E-04	-1.1175
ENSG00000000971	CFH	1.3325	3.90E-04	8.54E-04	-1.1205
ENSG00000241058	NSUN6	1.1918	3.90E-04	8.54E-04	-1.1209
ENSG00000135845	PIGC	1.1593	3.90E-04	8.55E-04	-1.1219
ENSG00000184371	CSF1	1.1815	3.90E-04	8.55E-04	-1.1227
ENSG00000117013	KCNQ4	1.0721	3.91E-04	8.57E-04	-1.1249
ENSG00000165655	ZNF503	1.1799	3.91E-04	8.57E-04	-1.1251
ENSG00000146192	FGD2	0.7420	3.92E-04	8.59E-04	-1.1272
ENSG00000133561	GIMAP6	0.8512	3.93E-04	8.61E-04	-1.1290
ENSG00000165568	AKR1E2	1.2129	3.93E-04	8.61E-04	-1.1289
ENSG00000170476	MZB1	0.7997	3.93E-04	8.61E-04	-1.1297
ENSG00000152359	POC5	0.8451	3.94E-04	8.63E-04	-1.1318
ENSG00000166793	YPEL4	1.2348	3.95E-04	8.65E-04	-1.1336
ENSG00000182362	YBEY	0.8199	3.96E-04	8.66E-04	-1.1351
ENSG00000145214	DGKQ	1.2920	3.96E-04	8.66E-04	-1.1353
ENSG00000173065	FAM222B	1.1690	3.96E-04	8.66E-04	-1.1358
ENSG00000167815	PRDX2	0.8033	3.96E-04	8.67E-04	-1.1370
ENSG00000151967	SCHIP1	1.0888	3.98E-04	8.70E-04	-1.1398
ENSG00000121741	ZMYM2	1.1883	3.99E-04	8.72E-04	-1.1423
ENSG00000163950	SLBP	1.1773	3.99E-04	8.73E-04	-1.1432
ENSG00000150768	DLAT	0.8226	4.00E-04	8.74E-04	-1.1445
ENSG00000132010	ZNF20	1.1311	4.00E-04	8.76E-04	-1.1464
ENSG00000100926	TM9SF1	0.8601	4.02E-04	8.79E-04	-1.1502
ENSG00000174938	SEZ6L2	1.3688	4.03E-04	8.80E-04	-1.1511
ENSG00000162813	BPNT1	0.7839	4.05E-04	8.86E-04	-1.1576
ENSG00000198915	RASGEF1A	1.3389	4.05E-04	8.86E-04	-1.1576
ENSG00000083807	SLC27A5	1.2836	4.06E-04	8.86E-04	-1.1582
ENSG00000168288	MMADHC	1.1106	4.06E-04	8.87E-04	-1.1591
ENSG00000170515	PA2G4	1.1380	4.06E-04	8.87E-04	-1.1596
ENSG00000170903	MSANTD4	0.8413	4.07E-04	8.90E-04	-1.1619
ENSG00000131174	COX7B	1.1257	4.07E-04	8.90E-04	-1.1622
ENSG00000177311	ZBTB38	0.7839	4.08E-04	8.92E-04	-1.1648
ENSG00000206384	COL6A6	1.0329	4.09E-04	8.94E-04	-1.1664
ENSG00000138286	FAM149B1	1.1666	4.10E-04	8.95E-04	-1.1684
ENSG00000140030	GPR65	0.7930	4.10E-04	8.95E-04	-1.1685
ENSG00000244274	DBNDD2	1.1982	4.11E-04	8.97E-04	-1.1701
ENSG00000132768	DPH2	0.8206	4.11E-04	8.97E-04	-1.1709
ENSG00000183793	NPIPA5	1.3554	4.13E-04	9.00E-04	-1.1742
ENSG00000130706	ADRM1	1.1363	4.13E-04	9.01E-04	-1.1744
ENSG00000169436	COL22A1	1.1238	4.13E-04	9.02E-04	-1.1757
ENSG00000156642	NPTN	0.8533	4.13E-04	9.02E-04	-1.1760
ENSG00000189410	SH2D5	1.0443	4.13E-04	9.02E-04	-1.1759
ENSG00000096968	JAK2	0.8416	4.14E-04	9.03E-04	-1.1778
ENSG00000162747	FCGR3B	0.6877	4.15E-04	9.04E-04	-1.1787
ENSG00000169760	NLGN1	1.1197	4.15E-04	9.06E-04	-1.1806
ENSG00000263002	ZNF234	0.8224	4.16E-04	9.06E-04	-1.1809
ENSG00000137809	ITGA11	1.0321	4.16E-04	9.08E-04	-1.1827
ENSG00000148053	NTRK2	1.3876	4.17E-04	9.09E-04	-1.1845
ENSG00000116544	DLGAP3	1.1264	4.18E-04	9.11E-04	-1.1862
ENSG00000143603	KCNN3	1.3681	4.18E-04	9.11E-04	-1.1866
ENSG00000101282	RSPO4	1.1036	4.19E-04	9.14E-04	-1.1892
ENSG00000134538	SLCO1B1	1.0806	4.19E-04	9.14E-04	-1.1897

Gene ID	Gene Symbol	FC	P.Value	adj.P.Val	B
ENSG00000168374	ARF4	0.8782	4.21E-04	9.16E-04	-1.1922
ENSG00000214654	B3GALT9	0.7896	4.22E-04	9.19E-04	-1.1949
ENSG00000185774	KCNIP4	1.0949	4.23E-04	9.20E-04	-1.1964
ENSG00000204086	RPA4	1.0669	4.23E-04	9.21E-04	-1.1972
ENSG00000172366	MCRIP2	0.8841	4.24E-04	9.24E-04	-1.1999
ENSG00000141101	NOB1	0.8572	4.25E-04	9.26E-04	-1.2021
ENSG00000160050	CCDC28B	1.1541	4.25E-04	9.26E-04	-1.2023
ENSG00000178821	TMEM52	1.3371	4.25E-04	9.26E-04	-1.2027
ENSG00000177182	CLVS1	1.3133	4.26E-04	9.27E-04	-1.2034
ENSG00000164081	TEX264	0.8848	4.27E-04	9.29E-04	-1.2055
ENSG00000149503	INCENP	1.2187	4.27E-04	9.30E-04	-1.2067
ENSG00000204264	PSMB8	0.8585	4.28E-04	9.31E-04	-1.2082
ENSG00000197766	CFD	0.7151	4.28E-04	9.32E-04	-1.2092
ENSG00000125730	C3	1.1254	4.29E-04	9.32E-04	-1.2098
ENSG00000182463	TSHZ2	1.3129	4.29E-04	9.33E-04	-1.2102
ENSG00000164649	CDCA7L	1.2372	4.30E-04	9.35E-04	-1.2125
ENSG00000179151	EDC3	0.8452	4.30E-04	9.35E-04	-1.2132
ENSG00000249242	TMEM150C	1.1069	4.31E-04	9.37E-04	-1.2145
ENSG00000083844	ZNF264	1.1598	4.31E-04	9.37E-04	-1.2152
ENSG00000054282	SDCCAG8	1.1425	4.31E-04	9.38E-04	-1.2156
ENSG00000173548	SNX33	0.8368	4.32E-04	9.39E-04	-1.2168
ENSG00000152953	STK32B	1.1278	4.32E-04	9.39E-04	-1.2175
ENSG00000112462	OR12D3	1.0059	4.34E-04	9.43E-04	-1.2213
ENSG00000230054	TEX53	1.1219	4.35E-04	9.45E-04	-1.2229
ENSG00000107831	FGF8	1.0467	4.35E-04	9.45E-04	-1.2233
ENSG00000114812	VIPR1	1.2820	4.35E-04	9.45E-04	-1.2233
ENSG00000262576	PCDHGA4	1.1211	4.35E-04	9.45E-04	-1.2235
ENSG00000149403	GRIK4	0.7037	4.36E-04	9.46E-04	-1.2247
ENSG00000120457	KCNJ5	1.1360	4.36E-04	9.46E-04	-1.2254
ENSG00000153347	FAM81B	1.1434	4.37E-04	9.49E-04	-1.2281
ENSG00000185818	NAT8L	1.0157	4.38E-04	9.51E-04	-1.2300
ENSG00000165588	OTX2	1.1080	4.38E-04	9.51E-04	-1.2303
ENSG00000132837	DMGDH	1.1747	4.42E-04	9.60E-04	-1.2386
ENSG00000091527	CDV3	0.8491	4.44E-04	9.64E-04	-1.2429
ENSG00000175485	OR52W1	0.9876	4.46E-04	9.67E-04	-1.2463
ENSG00000113580	NR3C1	1.2092	4.47E-04	9.69E-04	-1.2483
ENSG00000172531	PPP1CA	1.1295	4.47E-04	9.69E-04	-1.2483
ENSG00000101132	PFDN4	1.1597	4.48E-04	9.71E-04	-1.2507
ENSG00000081026	MAGI3	1.3240	4.48E-04	9.72E-04	-1.2510
ENSG00000105808	RASA4	0.7850	4.49E-04	9.75E-04	-1.2540
ENSG00000184515	BEX5	1.5452	4.50E-04	9.75E-04	-1.2544
ENSG00000204070	SYS1	1.1494	4.50E-04	9.76E-04	-1.2557
ENSG00000119185	ITGB1BP1	1.1632	4.51E-04	9.78E-04	-1.2576
ENSG00000172086	KRCC1	0.7965	4.51E-04	9.78E-04	-1.2579
ENSG00000178921	PFAS	1.2060	4.52E-04	9.80E-04	-1.2593
ENSG00000122642	FKBP9	1.2965	4.54E-04	9.83E-04	-1.2631
ENSG00000198420	TCAF1	1.2067	4.54E-04	9.83E-04	-1.2631
ENSG00000169442	CD52	1.7076	4.54E-04	9.84E-04	-1.2639
ENSG00000169439	SDC2	1.3321	4.55E-04	9.87E-04	-1.2662
ENSG00000197608	ZNF841	1.3877	4.56E-04	9.87E-04	-1.2668
ENSG00000206190	ATP10A	1.1435	4.58E-04	9.91E-04	-1.2711
ENSG00000242498	ARPIN	0.8895	4.59E-04	9.94E-04	-1.2739
ENSG00000164038	SLC9B2	1.2204	4.60E-04	9.95E-04	-1.2748
ENSG00000038382	TRIO	1.5262	4.60E-04	9.96E-04	-1.2761

Gene ID	Gene Symbol	FC	P.Value	adj.P.Val	B
ENSG00000183762	KREMEN1	1.1146	4.61E-04	9.98E-04	-1.2780
ENSG00000167780	SOAT2	1.1286	4.62E-04	9.99E-04	-1.2791
ENSG00000243667	DNAAF10	0.8054	4.63E-04	1.00E-03	-1.2815
ENSG00000143127	ITGA10	1.0997	4.63E-04	1.00E-03	-1.2823
ENSG00000131732	ZCCHC9	0.8690	4.65E-04	1.01E-03	-1.2860
ENSG00000055163	CYFIP2	1.2785	4.66E-04	1.01E-03	-1.2883
ENSG00000113249	HAVCR1	1.0533	4.68E-04	1.01E-03	-1.2914
ENSG00000058866	DGKG	1.2361	4.69E-04	1.01E-03	-1.2933
ENSG00000166582	CENPV	1.2442	4.72E-04	1.02E-03	-1.2997
ENSG00000104490	NCALD	1.2996	4.74E-04	1.02E-03	-1.3032
ENSG00000008853	RHOBTB2	1.2455	4.75E-04	1.03E-03	-1.3050
ENSG00000112667	DNPH1	0.7761	4.75E-04	1.03E-03	-1.3049
ENSG00000119392	GLE1	0.8306	4.75E-04	1.03E-03	-1.3050
ENSG00000104881	PPP1R13L	1.2475	4.76E-04	1.03E-03	-1.3071
ENSG00000155926	SLA	1.4299	4.76E-04	1.03E-03	-1.3084
ENSG00000162624	LHX8	1.4099	4.77E-04	1.03E-03	-1.3093
ENSG00000178860	MSC	1.1641	4.77E-04	1.03E-03	-1.3097
ENSG00000160886	LY6K	1.0582	4.78E-04	1.03E-03	-1.3108
ENSG00000100197	CYP2D6	1.1631	4.81E-04	1.04E-03	-1.3182
ENSG00000244234	GMCL2	1.0120	4.82E-04	1.04E-03	-1.3195
ENSG00000043591	ADRB1	1.0757	4.82E-04	1.04E-03	-1.3197
ENSG00000184903	IMMP2L	0.8458	4.82E-04	1.04E-03	-1.3199
ENSG00000163623	NKX6-1	1.0172	4.84E-04	1.05E-03	-1.3242
ENSG00000100368	CSF2RB	0.6657	4.85E-04	1.05E-03	-1.3244
ENSG00000127580	WDR24	1.1485	4.85E-04	1.05E-03	-1.3249
ENSG00000170345	FOS	0.6613	4.85E-04	1.05E-03	-1.3251
ENSG00000197982	C1orf122	1.1559	4.85E-04	1.05E-03	-1.3258
ENSG00000059122	FLYWCH1	1.2007	4.86E-04	1.05E-03	-1.3277
ENSG00000196214	ZNF766	1.1392	4.87E-04	1.05E-03	-1.3287
ENSG00000050030	NEXMIF	1.1796	4.87E-04	1.05E-03	-1.3288
ENSG00000132341	RAN	1.1285	4.88E-04	1.05E-03	-1.3310
ENSG00000169213	RAB3B	1.2586	4.88E-04	1.05E-03	-1.3311
ENSG00000105198	LGALS13	1.0647	4.90E-04	1.06E-03	-1.3343
ENSG00000177700	POLR2L	0.8772	4.90E-04	1.06E-03	-1.3342
ENSG00000108961	RANGRF	1.1505	4.90E-04	1.06E-03	-1.3350
ENSG00000158301	GPRASP2	1.1904	4.91E-04	1.06E-03	-1.3357
ENSG00000160862	AZGP1	2.1085	4.91E-04	1.06E-03	-1.3357
ENSG00000215644	GCGR	1.0186	4.91E-04	1.06E-03	-1.3369
ENSG00000167393	PPP2R3B	1.2397	4.92E-04	1.06E-03	-1.3387
ENSG00000184999	SLC22A10	1.0221	4.93E-04	1.06E-03	-1.3396
ENSG00000214897	PNMA6E	1.0157	4.93E-04	1.06E-03	-1.3395
ENSG00000170248	PDCD6IP	1.1562	4.95E-04	1.07E-03	-1.3432
ENSG00000105483	CARD8	0.8546	4.97E-04	1.07E-03	-1.3477
ENSG00000082175	PGR	1.0724	5.00E-04	1.08E-03	-1.3531
ENSG00000119711	ALDH6A1	1.2190	5.00E-04	1.08E-03	-1.3544
ENSG00000160124	MIX23	0.8471	5.02E-04	1.08E-03	-1.3569
ENSG00000135083	CCNJL	1.1025	5.03E-04	1.08E-03	-1.3584
ENSG00000141425	RPRD1A	1.1535	5.03E-04	1.08E-03	-1.3591
ENSG00000158156	XKR8	1.2023	5.06E-04	1.09E-03	-1.3644
ENSG00000185664	PMEL	1.1597	5.06E-04	1.09E-03	-1.3648
ENSG00000164399	IL3	1.0164	5.07E-04	1.09E-03	-1.3665
ENSG00000196199	MPHOSPH8	0.8259	5.07E-04	1.09E-03	-1.3674
ENSG00000237330	RNF223	1.0409	5.09E-04	1.10E-03	-1.3706
ENSG00000153071	DAB2	0.6916	5.10E-04	1.10E-03	-1.3717

Gene ID	Gene Symbol	FC	P.Value	adj.P.Val	B
ENSG00000102780	DGKH	1.0991	5.14E-04	1.11E-03	-1.3794
ENSG00000169242	EFNA1	1.2205	5.15E-04	1.11E-03	-1.3812
ENSG00000130702	LAMA5	1.2986	5.15E-04	1.11E-03	-1.3819
ENSG00000089351	GRAMD1A	1.2328	5.17E-04	1.11E-03	-1.3851
ENSG00000146802	TMEM168	0.7997	5.18E-04	1.11E-03	-1.3857
ENSG00000176390	CRLF3	1.1276	5.18E-04	1.11E-03	-1.3858
ENSG00000186376	ZNF75D	0.8223	5.20E-04	1.12E-03	-1.3893
ENSG00000179761	PIPOX	1.1206	5.20E-04	1.12E-03	-1.3894
ENSG00000112319	EYA4	1.2493	5.21E-04	1.12E-03	-1.3912
ENSG00000151718	WWC2	1.3647	5.21E-04	1.12E-03	-1.3921
ENSG00000170298	LGALS9B	1.0931	5.23E-04	1.12E-03	-1.3946
ENSG00000177842	ZNF620	1.1151	5.25E-04	1.13E-03	-1.3989
ENSG00000257950	P2RX5-TAX1BP3	1.1244	5.25E-04	1.13E-03	-1.3989
ENSG00000256087	ZNF432	1.2222	5.26E-04	1.13E-03	-1.4006
ENSG00000123104	ITPR2	1.1872	5.26E-04	1.13E-03	-1.4008
ENSG00000130066	SAT1	1.3135	5.27E-04	1.13E-03	-1.4022
ENSG00000197122	SRC	1.3923	5.28E-04	1.13E-03	-1.4036
ENSG00000142606	MMEL1	1.2756	5.28E-04	1.13E-03	-1.4044
ENSG00000197362	ZNF786	0.8370	5.28E-04	1.13E-03	-1.4049
ENSG00000153037	SRP19	0.8720	5.29E-04	1.14E-03	-1.4065
ENSG00000178301	AQP11	0.8826	5.30E-04	1.14E-03	-1.4077
ENSG00000107593	PKD2L1	0.8624	5.30E-04	1.14E-03	-1.4085
ENSG00000124302	CHST8	1.1330	5.30E-04	1.14E-03	-1.4086
ENSG00000152492	CCDC50	1.2514	5.31E-04	1.14E-03	-1.4096
ENSG00000143869	GDF7	1.1347	5.32E-04	1.14E-03	-1.4106
ENSG00000162944	RFTN2	1.0667	5.32E-04	1.14E-03	-1.4120
ENSG00000153976	HS3ST3A1	1.1021	5.33E-04	1.14E-03	-1.4127
ENSG00000173320	STOX2	1.2192	5.36E-04	1.15E-03	-1.4175
ENSG00000188039	NWD1	1.0587	5.35E-04	1.15E-03	-1.4174
ENSG00000100522	GNPNAT1	1.1891	5.36E-04	1.15E-03	-1.4181
ENSG00000163534	FCRL1	0.7297	5.36E-04	1.15E-03	-1.4181
ENSG00000139192	TAPBPL	0.8267	5.36E-04	1.15E-03	-1.4187
ENSG00000102287	GABRE	1.0779	5.37E-04	1.15E-03	-1.4199
ENSG00000198040	ZNF84	1.2634	5.37E-04	1.15E-03	-1.4200
ENSG00000196747	H2AC13	1.4303	5.37E-04	1.15E-03	-1.4202
ENSG00000164871	SPAG11B	1.0211	5.38E-04	1.15E-03	-1.4212
ENSG00000256223	ZNF10	1.3079	5.41E-04	1.16E-03	-1.4265
ENSG00000131094	C1QL1	1.0231	5.41E-04	1.16E-03	-1.4273
ENSG00000102970	CCL17	1.0310	5.42E-04	1.16E-03	-1.4285
ENSG00000206172	HBA1	0.5317	5.43E-04	1.16E-03	-1.4300
ENSG00000140459	CYP11A1	1.0414	5.45E-04	1.17E-03	-1.4334
ENSG00000150787	PTS	1.1253	5.45E-04	1.17E-03	-1.4345
ENSG00000120647	CCDC77	1.1595	5.46E-04	1.17E-03	-1.4363
ENSG00000177455	CD19	0.6537	5.48E-04	1.17E-03	-1.4394
ENSG00000177932	ZNF354C	1.1109	5.51E-04	1.18E-03	-1.4434
ENSG00000100564	PIGH	1.1645	5.51E-04	1.18E-03	-1.4446
ENSG00000136098	NEK3	1.1881	5.52E-04	1.18E-03	-1.4454
ENSG00000057608	GDI2	0.8771	5.52E-04	1.18E-03	-1.4462
ENSG00000110031	LPXN	0.8069	5.53E-04	1.18E-03	-1.4473
ENSG00000198753	PLXNB3	1.2522	5.54E-04	1.18E-03	-1.4488
ENSG00000178222	RNF212	1.3032	5.56E-04	1.19E-03	-1.4526
ENSG00000117122	MFAP2	1.3752	5.57E-04	1.19E-03	-1.4545
ENSG00000159173	TNNI1	1.0498	5.59E-04	1.20E-03	-1.4581
ENSG00000184678	H2BC21	1.4059	5.62E-04	1.20E-03	-1.4617

Gene ID	Gene Symbol	FC	P.Value	adj.P.Val	B
ENSG00000198829	SUCNR1	0.9004	5.63E-04	1.20E-03	-1.4636
ENSG00000100979	PLTP	1.6351	5.64E-04	1.20E-03	-1.4650
ENSG00000004846	ABCB5	1.0479	5.64E-04	1.21E-03	-1.4654
ENSG00000161048	NAPEPLD	0.8413	5.65E-04	1.21E-03	-1.4675
ENSG00000127952	STYXL1	0.8551	5.66E-04	1.21E-03	-1.4684
ENSG00000165349	SLC7A3	1.0311	5.67E-04	1.21E-03	-1.4709
ENSG00000129675	ARHGEF6	0.7726	5.68E-04	1.21E-03	-1.4731
ENSG00000133028	SCO1	1.1620	5.69E-04	1.22E-03	-1.4743
ENSG00000212657	KRTAP16-1	1.0586	5.74E-04	1.23E-03	-1.4816
ENSG00000106524	ANKMY2	1.1469	5.74E-04	1.23E-03	-1.4818
ENSG00000128040	SPINK2	1.3920	5.80E-04	1.24E-03	-1.4918
ENSG00000159733	ZFYVE28	1.2996	5.80E-04	1.24E-03	-1.4917
ENSG00000167123	CERCAM	0.7552	5.80E-04	1.24E-03	-1.4918
ENSG00000123977	DAW1	1.0642	5.80E-04	1.24E-03	-1.4926
ENSG00000149564	ESAM	1.4185	5.80E-04	1.24E-03	-1.4925
ENSG00000035115	SH3YL1	0.8189	5.82E-04	1.24E-03	-1.4951
ENSG00000122741	DCAF10	0.8376	5.82E-04	1.24E-03	-1.4955
ENSG00000183208	GDPGP1	0.8583	5.83E-04	1.25E-03	-1.4974
ENSG00000007202	KIAA0100	1.1493	5.84E-04	1.25E-03	-1.4979
ENSG00000161992	PRR35	1.0162	5.84E-04	1.25E-03	-1.4983
ENSG00000118777	ABCG2	0.6617	5.85E-04	1.25E-03	-1.5006
ENSG00000096060	FKBP5	1.4123	5.86E-04	1.25E-03	-1.5015
ENSG00000137261	KIAA0319	1.0418	5.86E-04	1.25E-03	-1.5021
ENSG00000243710	CFAP57	1.0967	5.87E-04	1.25E-03	-1.5023
ENSG00000109323	MANBA	1.1424	5.87E-04	1.25E-03	-1.5024
ENSG00000120992	LYPLA1	1.1261	5.87E-04	1.25E-03	-1.5028
ENSG00000181061	HIGD1A	0.8502	5.87E-04	1.25E-03	-1.5028
ENSG00000136235	GPNMB	0.7331	5.87E-04	1.25E-03	-1.5032
ENSG00000109586	GALNT7	1.2253	5.89E-04	1.26E-03	-1.5066
ENSG00000157036	EXOG	1.1748	5.90E-04	1.26E-03	-1.5078
ENSG00000138080	EMILIN1	1.4046	5.93E-04	1.26E-03	-1.5128
ENSG00000255587	RAB44	1.1335	5.93E-04	1.26E-03	-1.5131
ENSG00000146021	KLHL3	1.1999	5.94E-04	1.27E-03	-1.5137
ENSG00000177181	RIMKLA	1.0720	5.94E-04	1.27E-03	-1.5147
ENSG00000248712	CCDC153	1.1174	5.96E-04	1.27E-03	-1.5166
ENSG00000170899	GSTA4	1.3329	5.96E-04	1.27E-03	-1.5169
ENSG00000106348	IMPDH1	1.1754	5.97E-04	1.27E-03	-1.5191
ENSG00000099901	RANBP1	1.1543	5.97E-04	1.27E-03	-1.5194
ENSG00000165694	FRMD7	1.0081	5.98E-04	1.27E-03	-1.5196
ENSG00000147381	MAGEA4	1.1917	5.98E-04	1.27E-03	-1.5198
ENSG00000100325	ASCC2	1.1429	5.99E-04	1.28E-03	-1.5219
ENSG00000012174	MBTPS2	1.1846	6.02E-04	1.28E-03	-1.5265
ENSG00000114841	DNAH1	1.2222	6.02E-04	1.28E-03	-1.5272
ENSG00000142039	CCDC97	0.8554	6.04E-04	1.28E-03	-1.5289
ENSG00000041982	TNC	1.2450	6.04E-04	1.29E-03	-1.5295
ENSG00000164039	BDH2	1.2424	6.05E-04	1.29E-03	-1.5304
ENSG00000138759	FRAS1	1.1170	6.06E-04	1.29E-03	-1.5323
ENSG00000255974	CYP2A6	1.0355	6.06E-04	1.29E-03	-1.5327
ENSG00000122862	SRGN	1.2400	6.07E-04	1.29E-03	-1.5340
ENSG00000006715	VPS41	1.2081	6.08E-04	1.29E-03	-1.5357
ENSG00000184402	SS18L1	1.1556	6.08E-04	1.29E-03	-1.5356
ENSG00000205269	TMEM170B	1.1718	6.10E-04	1.30E-03	-1.5390
ENSG00000169330	MINAR1	0.8637	6.12E-04	1.30E-03	-1.5417
ENSG00000137812	KNL1	1.2427	6.13E-04	1.30E-03	-1.5434

Gene ID	Gene Symbol	FC	P.Value	adj.P.Val	B
ENSG00000179532	DNHD1	1.1980	6.14E-04	1.30E-03	-1.5446
ENSG00000163286	ALPG	1.0163	6.15E-04	1.31E-03	-1.5463
ENSG00000214097	SMCO1	0.9323	6.16E-04	1.31E-03	-1.5472
ENSG00000109819	PPARGC1A	1.4325	6.17E-04	1.31E-03	-1.5494
ENSG00000090104	RGS1	1.7279	6.19E-04	1.32E-03	-1.5526
ENSG00000185875	THNSL1	0.8100	6.20E-04	1.32E-03	-1.5535
ENSG00000160856	FCRL3	1.3710	6.20E-04	1.32E-03	-1.5545
ENSG00000141750	STAC2	1.0256	6.21E-04	1.32E-03	-1.5551
ENSG00000149823	VPS51	0.8631	6.22E-04	1.32E-03	-1.5570
ENSG00000134258	VTCN1	1.0130	6.23E-04	1.32E-03	-1.5579
ENSG00000143344	RGL1	1.3597	6.23E-04	1.32E-03	-1.5583
ENSG00000168959	GRM5	1.0639	6.23E-04	1.32E-03	-1.5585
ENSG00000121057	AKAP1	0.8107	6.23E-04	1.32E-03	-1.5588
ENSG00000108509	CAMTA2	1.1602	6.24E-04	1.32E-03	-1.5599
ENSG00000124207	CSE1L	1.1593	6.24E-04	1.32E-03	-1.5599
ENSG00000133119	RFC3	1.2189	6.24E-04	1.32E-03	-1.5598
ENSG00000029725	RABEP1	1.1502	6.26E-04	1.33E-03	-1.5633
ENSG00000196581	AJAP1	0.6363	6.28E-04	1.33E-03	-1.5654
ENSG00000143878	RHOB	1.3782	6.29E-04	1.33E-03	-1.5668
ENSG00000171295	ZNF440	1.1555	6.29E-04	1.33E-03	-1.5674
ENSG00000108953	YWHAE	0.8678	6.30E-04	1.34E-03	-1.5694
ENSG00000110651	CD81	1.5748	6.30E-04	1.34E-03	-1.5693
ENSG00000147570	DNAJC5B	1.2702	6.31E-04	1.34E-03	-1.5705
ENSG00000127080	IPPK	1.1231	6.31E-04	1.34E-03	-1.5708
ENSG00000165006	UBAP1	1.1186	6.31E-04	1.34E-03	-1.5708
ENSG00000144579	CTDSP1	1.1537	6.33E-04	1.34E-03	-1.5727
ENSG00000112238	PRDM13	1.0598	6.38E-04	1.35E-03	-1.5802
ENSG00000184708	EIF4ENIF1	1.1507	6.39E-04	1.35E-03	-1.5821
ENSG00000172379	ARNT2	0.7831	6.41E-04	1.36E-03	-1.5854
ENSG00000176678	FOXL1	1.0128	6.42E-04	1.36E-03	-1.5862
ENSG00000104228	TRIM35	1.1546	6.43E-04	1.36E-03	-1.5885
ENSG00000143643	TTC13	1.1820	6.44E-04	1.36E-03	-1.5888
ENSG00000072110	ACTN1	1.3095	6.44E-04	1.36E-03	-1.5890
ENSG00000113739	STC2	1.2913	6.44E-04	1.36E-03	-1.5896
ENSG00000163735	CXCL5	1.0772	6.45E-04	1.37E-03	-1.5904
ENSG00000145348	TBCK	1.1692	6.46E-04	1.37E-03	-1.5928
ENSG00000157368	IL34	1.0757	6.48E-04	1.37E-03	-1.5946
ENSG00000205426	KRT81	1.0686	6.48E-04	1.37E-03	-1.5949
ENSG00000157212	PAXIP1	1.1684	6.49E-04	1.37E-03	-1.5969
ENSG00000164411	GJB7	1.1021	6.49E-04	1.37E-03	-1.5968
ENSG00000075891	PAX2	1.1021	6.50E-04	1.38E-03	-1.5979
ENSG00000154059	IMPACT	1.2510	6.51E-04	1.38E-03	-1.5994
ENSG00000170892	TSEN34	0.8747	6.52E-04	1.38E-03	-1.6004
ENSG00000105246	EBI3	0.8168	6.52E-04	1.38E-03	-1.6014
ENSG00000101966	XIAP	1.1962	6.54E-04	1.38E-03	-1.6030
ENSG00000126947	ARMCX1	1.2735	6.54E-04	1.38E-03	-1.6040
ENSG00000065609	SNAP91	1.0408	6.54E-04	1.38E-03	-1.6043
ENSG00000196109	ZNF676	1.0733	6.56E-04	1.39E-03	-1.6065
ENSG00000070814	TCOF1	1.1375	6.56E-04	1.39E-03	-1.6067
ENSG00000139540	SLC39A5	1.0653	6.56E-04	1.39E-03	-1.6067
ENSG00000213347	MXD3	1.2052	6.56E-04	1.39E-03	-1.6071
ENSG00000176771	NCKAP5	1.0707	6.59E-04	1.39E-03	-1.6106
ENSG00000198440	ZNF583	1.2282	6.60E-04	1.40E-03	-1.6127
ENSG00000077942	FBLN1	1.1184	6.62E-04	1.40E-03	-1.6148

Gene ID	Gene Symbol	FC	P.Value	adj.P.Val	B
ENSG00000166927	MS4A7	0.6792	6.62E-04	1.40E-03	-1.6156
ENSG00000143891	GALM	1.2402	6.63E-04	1.40E-03	-1.6169
ENSG00000175352	NRIP3	1.1069	6.66E-04	1.41E-03	-1.6202
ENSG00000204514	ZNF814	0.8311	6.66E-04	1.41E-03	-1.6212
ENSG00000152583	SPARCL1	1.2488	6.67E-04	1.41E-03	-1.6220
ENSG00000160190	SLC37A1	1.3655	6.68E-04	1.41E-03	-1.6228
ENSG00000159921	GNE	1.1665	6.68E-04	1.41E-03	-1.6240
ENSG00000172977	KAT5	1.1151	6.69E-04	1.41E-03	-1.6253
ENSG00000139144	PIK3C2G	1.0361	6.70E-04	1.41E-03	-1.6256
ENSG00000049323	LTBP1	0.7441	6.70E-04	1.41E-03	-1.6267
ENSG00000111335	OAS2	0.7840	6.71E-04	1.42E-03	-1.6271
ENSG00000159713	TPPP3	1.0967	6.71E-04	1.42E-03	-1.6274
ENSG00000214274	ANG	1.3645	6.71E-04	1.42E-03	-1.6280
ENSG00000187527	ATP13A5	1.0351	6.74E-04	1.42E-03	-1.6311
ENSG00000071564	TCF3	1.1677	6.74E-04	1.42E-03	-1.6316
ENSG00000138785	INTS12	1.1190	6.74E-04	1.42E-03	-1.6320
ENSG00000186452	TMPRSS12	1.0250	6.77E-04	1.43E-03	-1.6355
ENSG00000091428	RAPGEF4	0.7213	6.77E-04	1.43E-03	-1.6361
ENSG00000116750	UCHL5	1.1683	6.78E-04	1.43E-03	-1.6371
ENSG00000099341	PSMD8	0.8939	6.79E-04	1.43E-03	-1.6389
ENSG00000065989	PDE4A	1.1976	6.82E-04	1.44E-03	-1.6423
ENSG00000148335	NTMT1	0.8846	6.82E-04	1.44E-03	-1.6430
ENSG00000102189	EEA1	1.1513	6.83E-04	1.44E-03	-1.6445
ENSG00000100628	ASB2	1.3366	6.84E-04	1.44E-03	-1.6456
ENSG00000147724	FAM135B	1.0569	6.85E-04	1.44E-03	-1.6466
ENSG00000058804	NDC1	1.1649	6.86E-04	1.44E-03	-1.6477
ENSG00000187244	BCAM	1.1796	6.86E-04	1.44E-03	-1.6478
ENSG00000141542	RAB40B	1.1723	6.86E-04	1.45E-03	-1.6482
ENSG00000145757	SPATA9	0.8961	6.87E-04	1.45E-03	-1.6490
ENSG00000164118	CEP44	1.1738	6.87E-04	1.45E-03	-1.6494
ENSG00000182545	RNASE10	1.0943	6.89E-04	1.45E-03	-1.6524
ENSG00000118702	GHRH	1.1117	6.91E-04	1.45E-03	-1.6547
ENSG00000105552	BCAT2	0.8636	6.92E-04	1.46E-03	-1.6565
ENSG00000184486	POU3F2	1.0444	6.92E-04	1.46E-03	-1.6567
ENSG00000139151	PLCZ1	1.0571	6.94E-04	1.46E-03	-1.6594
ENSG00000110852	CLEC2B	1.6180	6.95E-04	1.46E-03	-1.6600
ENSG00000174943	KCTD13	1.1654	6.95E-04	1.46E-03	-1.6601
ENSG00000198879	SFMBT2	1.2067	6.98E-04	1.47E-03	-1.6643
ENSG00000168509	HJV	1.0192	7.01E-04	1.47E-03	-1.6675
ENSG00000177570	SAMD12	1.2041	7.02E-04	1.48E-03	-1.6700
ENSG00000183010	PYCR1	0.8032	7.04E-04	1.48E-03	-1.6720
ENSG00000108641	B9D1	0.8000	7.05E-04	1.48E-03	-1.6734
ENSG00000106100	NOD1	1.1727	7.07E-04	1.49E-03	-1.6759
ENSG00000185736	ADARB2	1.0926	7.08E-04	1.49E-03	-1.6770
ENSG00000184743	ATL3	1.1461	7.12E-04	1.50E-03	-1.6824
ENSG00000256980	KHDC1L	1.1101	7.12E-04	1.50E-03	-1.6826
ENSG00000266964	FXYD1	1.1962	7.13E-04	1.50E-03	-1.6837
ENSG00000148218	ALAD	0.8503	7.15E-04	1.50E-03	-1.6869
ENSG00000169951	ZNF764	1.1483	7.19E-04	1.51E-03	-1.6915
ENSG00000106105	GARS1	1.1678	7.26E-04	1.53E-03	-1.7006
ENSG00000071073	MGAT4A	1.2869	7.28E-04	1.53E-03	-1.7029
ENSG00000213337	ANKRD39	1.1538	7.28E-04	1.53E-03	-1.7033
ENSG00000158528	PPP1R9A	1.2051	7.29E-04	1.53E-03	-1.7050
ENSG00000078124	ACER3	1.1654	7.31E-04	1.54E-03	-1.7074

Gene ID	Gene Symbol	FC	P.Value	adj.P.Val	B
ENSG00000270181	BIVM-ERCC5	1.1423	7.31E-04	1.54E-03	-1.7075
ENSG00000180730	SHISA2	0.6688	7.34E-04	1.54E-03	-1.7109
ENSG00000173705	SUSD5	1.0496	7.34E-04	1.54E-03	-1.7110
ENSG00000112232	KHDRBS2	1.2856	7.36E-04	1.54E-03	-1.7129
ENSG00000123213	NLN	1.1601	7.36E-04	1.54E-03	-1.7132
ENSG00000166165	CKB	1.4822	7.36E-04	1.55E-03	-1.7140
ENSG00000235961	PNMA6A	1.3161	7.38E-04	1.55E-03	-1.7154
ENSG00000119927	GPAM	1.1500	7.38E-04	1.55E-03	-1.7156
ENSG00000136840	ST6GALNAC4	1.1866	7.38E-04	1.55E-03	-1.7156
ENSG00000167083	GNGT2	0.8132	7.39E-04	1.55E-03	-1.7178
ENSG00000255274	SMIM35	1.0824	7.43E-04	1.56E-03	-1.7225
ENSG00000174456	C12orf76	1.1227	7.45E-04	1.56E-03	-1.7241
ENSG00000187566	NHLRC1	1.0581	7.46E-04	1.56E-03	-1.7260
ENSG00000126890	CTAG2	1.6069	7.47E-04	1.57E-03	-1.7277
ENSG00000205238	SPDYE2	1.2339	7.50E-04	1.57E-03	-1.7306
ENSG00000095209	TMEM38B	1.1449	7.55E-04	1.58E-03	-1.7366
ENSG00000181029	TRAPPC5	0.8946	7.55E-04	1.58E-03	-1.7371
ENSG00000183688	RFLNB	0.6875	7.56E-04	1.59E-03	-1.7388
ENSG00000077713	SLC25A43	1.2297	7.60E-04	1.59E-03	-1.7431
ENSG00000122194	PLG	1.0996	7.60E-04	1.59E-03	-1.7438
ENSG00000187682	ERAS	1.0499	7.61E-04	1.60E-03	-1.7450
ENSG00000169016	E2F6	1.1895	7.62E-04	1.60E-03	-1.7453
ENSG00000177212	OR2T33	1.0296	7.64E-04	1.60E-03	-1.7475
ENSG00000244734	HBB	0.5573	7.64E-04	1.60E-03	-1.7477
ENSG00000168061	SAC3D1	0.7954	7.64E-04	1.60E-03	-1.7484
ENSG00000040731	CDH10	1.1595	7.67E-04	1.61E-03	-1.7519
ENSG00000087586	AURKA	1.2511	7.68E-04	1.61E-03	-1.7530
ENSG00000185633	NDUFA4L2	0.7585	7.69E-04	1.61E-03	-1.7546
ENSG00000241598	KRTAP5-4	1.0074	7.70E-04	1.61E-03	-1.7555
ENSG00000184110	EIF3C	1.1690	7.71E-04	1.61E-03	-1.7561
ENSG00000106236	NPTX2	1.0850	7.72E-04	1.62E-03	-1.7583
ENSG00000178917	ZNF852	0.8510	7.74E-04	1.62E-03	-1.7600
ENSG00000105289	TJP3	0.7976	7.74E-04	1.62E-03	-1.7606
ENSG00000152034	MCHR2	1.0312	7.76E-04	1.62E-03	-1.7626
ENSG00000176715	ACSF3	0.8430	7.79E-04	1.63E-03	-1.7664
ENSG00000101605	MYOM1	1.1358	7.80E-04	1.63E-03	-1.7674
ENSG00000117791	MTARC2	0.7441	7.81E-04	1.63E-03	-1.7683
ENSG00000196104	SPOCK3	1.1040	7.83E-04	1.64E-03	-1.7708
ENSG00000128050	PAICS	0.8071	7.87E-04	1.64E-03	-1.7753
ENSG00000157911	PEX10	1.1280	7.89E-04	1.65E-03	-1.7783
ENSG00000136574	GATA4	1.0920	7.91E-04	1.65E-03	-1.7798
ENSG00000163412	EIF4E3	1.1647	7.91E-04	1.65E-03	-1.7807
ENSG00000147082	CCNB3	1.0950	7.92E-04	1.66E-03	-1.7818
ENSG00000111404	RERGL	1.0621	7.94E-04	1.66E-03	-1.7839
ENSG00000067842	ATP2B3	1.0181	7.96E-04	1.66E-03	-1.7862
ENSG00000237441	RGL2	1.2059	7.96E-04	1.66E-03	-1.7865
ENSG00000129968	ABHD17A	0.9031	7.97E-04	1.66E-03	-1.7872
ENSG00000187569	DPPA3	1.0143	7.98E-04	1.67E-03	-1.7887
ENSG00000089123	TASP1	1.1259	7.98E-04	1.67E-03	-1.7889
ENSG00000114019	AMOTL2	1.1122	8.01E-04	1.67E-03	-1.7924
ENSG00000091583	APOH	1.0600	8.02E-04	1.67E-03	-1.7932
ENSG00000152413	HOMER1	0.7259	8.02E-04	1.67E-03	-1.7932
ENSG00000141337	ARSG	0.8327	8.04E-04	1.68E-03	-1.7959
ENSG00000187122	SLIT1	0.8337	8.04E-04	1.68E-03	-1.7960

Gene ID	Gene Symbol	FC	P.Value	adj.P.Val	B
ENSG00000140931	CMTM3	1.2468	8.05E-04	1.68E-03	-1.7962
ENSG00000116704	SLC35D1	1.1650	8.07E-04	1.68E-03	-1.7990
ENSG00000137124	ALDH1B1	0.8245	8.10E-04	1.69E-03	-1.8020
ENSG00000148248	SURF4	1.1131	8.10E-04	1.69E-03	-1.8026
ENSG00000150459	SAP18	1.1127	8.12E-04	1.69E-03	-1.8046
ENSG00000203724	C1orf53	0.8099	8.13E-04	1.70E-03	-1.8062
ENSG00000118200	CAMSAP2	1.2181	8.14E-04	1.70E-03	-1.8070
ENSG00000184857	TMEM186	0.8538	8.15E-04	1.70E-03	-1.8079
ENSG00000171433	GLOD5	1.1057	8.15E-04	1.70E-03	-1.8083
ENSG00000198093	ZNF649	1.1692	8.20E-04	1.71E-03	-1.8135
ENSG00000143365	RORC	1.2426	8.23E-04	1.72E-03	-1.8172
ENSG00000169432	SCN9A	1.2967	8.23E-04	1.72E-03	-1.8174
ENSG00000136021	SCYL2	0.7715	8.24E-04	1.72E-03	-1.8188
ENSG00000099364	FBXL19	1.1295	8.27E-04	1.72E-03	-1.8212
ENSG00000174165	ZDHHC24	0.8847	8.27E-04	1.72E-03	-1.8218
ENSG00000105609	LILRB5	0.7318	8.28E-04	1.73E-03	-1.8228
ENSG00000115738	ID2	1.4267	8.28E-04	1.73E-03	-1.8226
ENSG00000172164	SNTB1	1.3397	8.28E-04	1.73E-03	-1.8227
ENSG00000132329	RAMP1	1.0487	8.29E-04	1.73E-03	-1.8242
ENSG00000167165	UGT1A6	0.9203	8.29E-04	1.73E-03	-1.8243
ENSG00000243477	NAA80	1.1517	8.29E-04	1.73E-03	-1.8242
ENSG00000147697	GSDMC	1.0635	8.33E-04	1.74E-03	-1.8287
ENSG00000174607	UGT8	0.7655	8.35E-04	1.74E-03	-1.8301
ENSG00000144908	ALDH1L1	0.7591	8.36E-04	1.74E-03	-1.8319
ENSG00000136449	MYCBPAP	1.1901	8.36E-04	1.74E-03	-1.8322
ENSG00000143799	PARP1	1.2005	8.37E-04	1.74E-03	-1.8327
ENSG00000197872	CYRIA	1.4264	8.39E-04	1.75E-03	-1.8354
ENSG00000144290	SLC4A10	1.1458	8.43E-04	1.75E-03	-1.8390
ENSG00000134330	IAH1	1.1267	8.43E-04	1.75E-03	-1.8395
ENSG00000188771	PLET1	1.0376	8.44E-04	1.76E-03	-1.8401
ENSG00000181619	GPR135	1.1301	8.44E-04	1.76E-03	-1.8407
ENSG00000183150	GPR19	1.1504	8.46E-04	1.76E-03	-1.8429
ENSG00000100342	APOL1	0.7912	8.47E-04	1.76E-03	-1.8436
ENSG00000175471	MCTP1	1.2785	8.47E-04	1.76E-03	-1.8437
ENSG00000170891	CYTL1	1.2602	8.49E-04	1.77E-03	-1.8463
ENSG00000009335	UBE3C	1.1449	8.52E-04	1.77E-03	-1.8497
ENSG00000141179	PCTP	0.8564	8.52E-04	1.77E-03	-1.8497
ENSG00000138769	CDKL2	1.0798	8.55E-04	1.78E-03	-1.8523
ENSG00000007174	DNAH9	1.0596	8.57E-04	1.78E-03	-1.8551
ENSG00000142507	PSMB6	1.1437	8.60E-04	1.79E-03	-1.8579
ENSG00000118156	ZNF541	1.0361	8.61E-04	1.79E-03	-1.8596
ENSG00000187533	PRR27	1.0340	8.65E-04	1.80E-03	-1.8637
ENSG00000198056	PRIM1	1.2397	8.65E-04	1.80E-03	-1.8636
ENSG00000256453	DND1	0.9139	8.66E-04	1.80E-03	-1.8644
ENSG00000155099	PIP4P2	1.1653	8.66E-04	1.80E-03	-1.8648
ENSG00000152689	RASGRP3	1.4156	8.70E-04	1.81E-03	-1.8683
ENSG00000185963	BICD2	1.1364	8.70E-04	1.81E-03	-1.8686
ENSG00000140470	ADAMTS17	1.2886	8.70E-04	1.81E-03	-1.8688
ENSG00000171711	DEFB4A	1.2536	8.79E-04	1.82E-03	-1.8781
ENSG00000121577	POPDC2	1.1469	8.79E-04	1.83E-03	-1.8785
ENSG00000185650	ZFP36L1	1.2741	8.80E-04	1.83E-03	-1.8788
ENSG00000099949	LZTR1	1.1787	8.83E-04	1.83E-03	-1.8826
ENSG00000006025	OSBPL7	1.1789	8.84E-04	1.83E-03	-1.8834
ENSG00000185722	ANKFY1	1.1803	8.87E-04	1.84E-03	-1.8866

Gene ID	Gene Symbol	FC	P.Value	adj.P.Val	B
ENSG00000105707	HPN	1.1084	8.88E-04	1.84E-03	-1.8872
ENSG00000128951	DUT	0.8530	8.89E-04	1.85E-03	-1.8891
ENSG00000170667	RASA4B	0.8127	8.93E-04	1.85E-03	-1.8930
ENSG00000141219	C17orf80	0.8288	8.94E-04	1.85E-03	-1.8939
ENSG00000115232	ITGA4	1.2289	8.95E-04	1.86E-03	-1.8947
ENSG00000141316	SPACA3	0.6603	8.98E-04	1.86E-03	-1.8978
ENSG00000109063	MYH3	1.1440	9.05E-04	1.88E-03	-1.9056
ENSG00000241794	SPRR2A	1.2465	9.07E-04	1.88E-03	-1.9076
ENSG00000170965	PLAC1	1.3306	9.08E-04	1.88E-03	-1.9081
ENSG00000182712	CMC4	1.1774	9.12E-04	1.89E-03	-1.9120
ENSG00000131446	MGAT1	0.8968	9.16E-04	1.90E-03	-1.9166
ENSG00000111737	RAB35	1.1721	9.21E-04	1.91E-03	-1.9218
ENSG00000181513	ACBD4	0.8308	9.22E-04	1.91E-03	-1.9228
ENSG00000106460	TMEM106B	0.8157	9.24E-04	1.91E-03	-1.9242
ENSG00000071909	MYO3B	1.1652	9.25E-04	1.92E-03	-1.9256
ENSG00000112706	IMPG1	1.1037	9.28E-04	1.92E-03	-1.9289
ENSG00000085831	TTC39A	1.2132	9.30E-04	1.93E-03	-1.9304
ENSG00000117016	RIMS3	1.1547	9.32E-04	1.93E-03	-1.9326
ENSG00000076641	PAG1	0.7179	9.35E-04	1.94E-03	-1.9359
ENSG00000081087	OSTM1	0.8402	9.36E-04	1.94E-03	-1.9364
ENSG00000198626	RYR2	1.1194	9.37E-04	1.94E-03	-1.9378
ENSG00000198182	ZNF607	1.1390	9.42E-04	1.95E-03	-1.9419
ENSG00000125850	OVOL2	1.1045	9.42E-04	1.95E-03	-1.9421
ENSG00000180884	ZNF792	0.8822	9.44E-04	1.95E-03	-1.9444
ENSG00000084636	COL16A1	1.1695	9.46E-04	1.96E-03	-1.9464
ENSG00000073754	CD5L	0.6675	9.49E-04	1.96E-03	-1.9489
ENSG00000112576	CCND3	1.3860	9.49E-04	1.96E-03	-1.9496
ENSG00000137726	FXYD6	0.7850	9.50E-04	1.96E-03	-1.9499
ENSG00000165168	CYBB	1.5691	9.54E-04	1.97E-03	-1.9537
ENSG00000144130	NT5DC4	1.0569	9.54E-04	1.97E-03	-1.9546
ENSG00000180777	ANKRD30B	1.0974	9.54E-04	1.97E-03	-1.9546
ENSG00000169919	GUSB	1.1587	9.55E-04	1.98E-03	-1.9554
ENSG00000136169	SETDB2	0.8416	9.60E-04	1.98E-03	-1.9596
ENSG00000123329	ARHGAP9	0.7968	9.61E-04	1.99E-03	-1.9610
ENSG00000104518	GSDMD	0.8657	9.62E-04	1.99E-03	-1.9622
ENSG00000163216	SPRR2D	1.2390	9.65E-04	1.99E-03	-1.9647
ENSG00000158747	NBL1	1.0806	9.65E-04	1.99E-03	-1.9649
ENSG00000254126	CD8B2	1.2917	9.66E-04	2.00E-03	-1.9657
ENSG00000196704	AMZ2	1.1205	9.67E-04	2.00E-03	-1.9662
ENSG00000101311	FERMT1	1.0692	9.68E-04	2.00E-03	-1.9675
ENSG00000247626	MARS2	0.8249	9.71E-04	2.01E-03	-1.9706
ENSG00000187848	P2RX2	1.1175	9.74E-04	2.01E-03	-1.9731
ENSG00000160305	DIP2A	1.1582	9.74E-04	2.01E-03	-1.9737
ENSG00000239704	CDRT4	1.3846	9.75E-04	2.01E-03	-1.9741
ENSG00000180071	ANKRD18A	1.1631	9.75E-04	2.01E-03	-1.9744
ENSG00000100599	RIN3	1.2758	9.76E-04	2.01E-03	-1.9751
ENSG00000185716	MOSMO	0.8477	9.79E-04	2.02E-03	-1.9783
ENSG00000179915	NRXN1	1.3396	9.80E-04	2.02E-03	-1.9794
ENSG00000172901	LVRN	0.9120	9.83E-04	2.03E-03	-1.9818
ENSG00000116990	MYCL	0.8387	9.84E-04	2.03E-03	-1.9824
ENSG00000182768	NGRN	0.8769	9.87E-04	2.04E-03	-1.9852
ENSG00000138685	FGF2	1.3505	9.88E-04	2.04E-03	-1.9870
ENSG00000198039	ZNF273	1.1690	9.89E-04	2.04E-03	-1.9878
ENSG00000164949	GEM	1.5437	9.90E-04	2.04E-03	-1.9882

Gene ID	Gene Symbol	FC	P.Value	adj.P.Val	B
ENSG00000079215	SLC1A3	0.8115	9.92E-04	2.05E-03	-1.9904
ENSG00000127022	CANX	0.8696	9.92E-04	2.05E-03	-1.9905
ENSG00000187134	AKR1C1	1.1640	9.94E-04	2.05E-03	-1.9917
ENSG00000185155	MIXL1	0.7905	9.96E-04	2.05E-03	-1.9944
ENSG00000134597	RBMX2	1.1526	9.97E-04	2.06E-03	-1.9947
ENSG00000214193	SH3D21	1.1680	9.98E-04	2.06E-03	-1.9958
ENSG00000165695	AK8	1.0487	1.00E-03	2.07E-03	-1.9996
ENSG00000223443	USP17L2	1.0157	1.01E-03	2.08E-03	-2.0041
ENSG00000130844	ZNF331	1.2764	1.01E-03	2.08E-03	-2.0045
ENSG00000141642	ELAC1	1.1574	1.01E-03	2.08E-03	-2.0064
ENSG00000130305	NSUN5	1.1551	1.01E-03	2.09E-03	-2.0089
ENSG00000179943	FIZ1	0.8743	1.01E-03	2.09E-03	-2.0089
ENSG00000108821	COL1A1	1.2664	1.01E-03	2.09E-03	-2.0092
ENSG00000064393	HIPK2	1.2404	1.02E-03	2.10E-03	-2.0166
ENSG00000125848	FLRT3	1.0893	1.02E-03	2.11E-03	-2.0193
ENSG00000130544	ZNF557	1.1293	1.02E-03	2.11E-03	-2.0197
ENSG00000120669	SOHLH2	0.7500	1.03E-03	2.11E-03	-2.0209
ENSG00000163563	MNDA	0.6799	1.03E-03	2.11E-03	-2.0220
ENSG00000234284	ZNF879	1.1457	1.03E-03	2.11E-03	-2.0222
ENSG00000196277	GRM7	1.0936	1.03E-03	2.12E-03	-2.0234
ENSG00000121335	PRB2	1.1407	1.03E-03	2.12E-03	-2.0245
ENSG00000071242	RPS6KA2	1.3026	1.03E-03	2.13E-03	-2.0288
ENSG00000100033	PRODH	1.0984	1.03E-03	2.13E-03	-2.0288
ENSG00000147257	GPC3	1.0157	1.04E-03	2.13E-03	-2.0313
ENSG00000109956	B3GAT1	1.0404	1.04E-03	2.13E-03	-2.0316
ENSG00000189325	BNIP5	1.0501	1.04E-03	2.13E-03	-2.0316
ENSG00000174326	SLC16A11	1.1940	1.04E-03	2.14E-03	-2.0331
ENSG00000164151	ICE1	1.1400	1.04E-03	2.14E-03	-2.0358
ENSG00000168234	TTC39C	1.1611	1.04E-03	2.14E-03	-2.0364
ENSG00000198553	KCNRG	1.0491	1.04E-03	2.14E-03	-2.0368
ENSG00000137509	PRCP	0.8423	1.04E-03	2.15E-03	-2.0372
ENSG00000213699	SLC35F6	1.1466	1.04E-03	2.15E-03	-2.0382
ENSG00000137558	PI15	1.1710	1.05E-03	2.15E-03	-2.0386
ENSG00000182896	TMEM95	1.0121	1.05E-03	2.16E-03	-2.0426
ENSG00000066405	CLDN18	1.0103	1.05E-03	2.16E-03	-2.0428
ENSG00000185829	ARL17A	1.1911	1.05E-03	2.16E-03	-2.0432
ENSG00000167173	C15orf39	0.8144	1.05E-03	2.16E-03	-2.0447
ENSG00000100218	RSPH14	1.1392	1.05E-03	2.17E-03	-2.0471
ENSG00000243335	KCTD7	1.1549	1.06E-03	2.17E-03	-2.0493
ENSG00000084207	GSTP1	1.4224	1.06E-03	2.17E-03	-2.0502
ENSG00000237412	PRSS56	1.0132	1.06E-03	2.17E-03	-2.0504
ENSG00000179399	GPC5	1.0245	1.06E-03	2.18E-03	-2.0523
ENSG00000166126	AMN	0.8155	1.06E-03	2.18E-03	-2.0532
ENSG00000197629	MPEG1	0.6938	1.06E-03	2.18E-03	-2.0545
ENSG00000243317	STMP1	1.1269	1.06E-03	2.18E-03	-2.0548
ENSG00000150672	DLG2	1.2130	1.06E-03	2.18E-03	-2.0554
ENSG00000107159	CA9	1.0250	1.06E-03	2.18E-03	-2.0557
ENSG00000136816	TOR1B	0.8627	1.07E-03	2.19E-03	-2.0570
ENSG00000146285	SCML4	1.0622	1.08E-03	2.21E-03	-2.0652
ENSG00000231925	TAPBP	1.1466	1.08E-03	2.21E-03	-2.0672
ENSG00000006468	ETV1	1.6040	1.08E-03	2.22E-03	-2.0697
ENSG00000116991	SIPA1L2	1.2052	1.08E-03	2.22E-03	-2.0700
ENSG00000169499	PLEKHA2	1.2103	1.08E-03	2.22E-03	-2.0702
ENSG00000151005	TKTL2	1.0058	1.08E-03	2.22E-03	-2.0712

Gene ID	Gene Symbol	FC	P.Value	adj.P.Val	B
ENSG00000122861	PLAU	1.1587	1.08E-03	2.22E-03	-2.0716
ENSG00000145384	FABP2	1.0821	1.08E-03	2.22E-03	-2.0724
ENSG00000054356	PTPRN	1.0377	1.08E-03	2.22E-03	-2.0728
ENSG00000180210	F2	1.0125	1.09E-03	2.23E-03	-2.0737
ENSG00000140297	GCNT3	0.8042	1.09E-03	2.23E-03	-2.0750
ENSG00000184838	PRR16	1.0645	1.09E-03	2.23E-03	-2.0772
ENSG00000115816	CEBPZ	1.1272	1.09E-03	2.24E-03	-2.0798
ENSG00000127616	SMARCA4	1.1358	1.10E-03	2.25E-03	-2.0840
ENSG00000134200	TSHB	1.0374	1.10E-03	2.25E-03	-2.0839
ENSG00000254585	MAGEL2	1.1093	1.10E-03	2.25E-03	-2.0838
ENSG00000235631	RNF148	1.2567	1.10E-03	2.25E-03	-2.0845
ENSG00000162086	ZNF75A	1.1691	1.10E-03	2.25E-03	-2.0853
ENSG00000089289	IGBP1	0.8982	1.10E-03	2.25E-03	-2.0856
ENSG00000168229	PTGDR	0.7264	1.10E-03	2.25E-03	-2.0862
ENSG00000269891	ARHGAP19-SLIT1	1.1409	1.10E-03	2.26E-03	-2.0875
ENSG00000106771	TMEM245	0.8271	1.10E-03	2.26E-03	-2.0881
ENSG00000142910	TINAGL1	1.1755	1.11E-03	2.26E-03	-2.0903
ENSG00000086696	HSD17B2	1.0850	1.11E-03	2.26E-03	-2.0914
ENSG00000147614	ATP6V0D2	1.0515	1.11E-03	2.27E-03	-2.0939
ENSG00000172201	ID4	1.1233	1.11E-03	2.27E-03	-2.0942
ENSG00000112118	MCM3	1.2758	1.11E-03	2.27E-03	-2.0947
ENSG00000167110	GOLGA2	1.1417	1.11E-03	2.28E-03	-2.0970
ENSG00000174562	KLK15	1.0194	1.11E-03	2.28E-03	-2.0972
ENSG00000250722	SELENOP	0.7018	1.11E-03	2.28E-03	-2.0972
ENSG00000166840	GLYATL1	1.1177	1.12E-03	2.28E-03	-2.0988
ENSG00000000457	SCYL3	1.1610	1.12E-03	2.29E-03	-2.1028
ENSG00000002933	TMEM176A	1.4012	1.12E-03	2.29E-03	-2.1037
ENSG00000156976	EIF4A2	0.8940	1.12E-03	2.29E-03	-2.1038
ENSG00000155659	VSIG4	0.7019	1.12E-03	2.30E-03	-2.1065
ENSG00000164904	ALDH7A1	1.3466	1.13E-03	2.30E-03	-2.1067
ENSG00000181392	SYNE4	1.0684	1.13E-03	2.31E-03	-2.1117
ENSG00000167644	C19orf33	1.1481	1.13E-03	2.31E-03	-2.1122
ENSG00000206113	CFAP99	1.0684	1.14E-03	2.32E-03	-2.1159
ENSG00000250745	USP17L20	1.0075	1.14E-03	2.33E-03	-2.1186
ENSG00000136206	SPDYE1	1.1081	1.14E-03	2.34E-03	-2.1220
ENSG00000116701	NCF2	1.3190	1.15E-03	2.35E-03	-2.1258
ENSG00000164161	HHIP	1.2728	1.15E-03	2.35E-03	-2.1264
ENSG00000189164	ZNF527	1.1776	1.15E-03	2.35E-03	-2.1292
ENSG00000102878	HSF4	1.2972	1.15E-03	2.36E-03	-2.1297
ENSG00000100473	COCH	1.4649	1.15E-03	2.36E-03	-2.1308
ENSG00000140107	SLC25A47	1.0286	1.16E-03	2.36E-03	-2.1334
ENSG00000148356	LRSAM1	1.1355	1.16E-03	2.37E-03	-2.1344
ENSG00000103226	NOMO3	1.1732	1.16E-03	2.37E-03	-2.1355
ENSG00000166979	EVA1C	1.2537	1.16E-03	2.37E-03	-2.1358
ENSG00000064651	SLC12A2	1.1598	1.16E-03	2.37E-03	-2.1376
ENSG00000101938	CHRDL1	1.1786	1.16E-03	2.37E-03	-2.1379
ENSG00000213218	CSH2	1.0225	1.16E-03	2.38E-03	-2.1388
ENSG00000179981	TSHZ1	1.1561	1.17E-03	2.38E-03	-2.1411
ENSG00000150750	C11orf53	1.1645	1.17E-03	2.38E-03	-2.1415
ENSG00000136960	ENPP2	1.2828	1.17E-03	2.38E-03	-2.1418
ENSG00000176171	BNIP3	1.4261	1.17E-03	2.39E-03	-2.1426
ENSG00000205038	PKHD1L1	0.8126	1.17E-03	2.39E-03	-2.1433
ENSG00000163297	ANTXR2	1.2943	1.17E-03	2.39E-03	-2.1448
ENSG00000145703	IQGAP2	1.4285	1.17E-03	2.39E-03	-2.1449

Gene ID	Gene Symbol	FC	P.Value	adj.P.Val	B
ENSG00000158555	GDPD5	0.7726	1.17E-03	2.39E-03	-2.1454
ENSG00000198551	ZNF627	1.1229	1.18E-03	2.40E-03	-2.1489
ENSG00000183853	KIRREL1	1.1141	1.18E-03	2.40E-03	-2.1499
ENSG00000173918	C1QTNF1	1.1027	1.18E-03	2.41E-03	-2.1527
ENSG00000196227	FAM217B	1.1818	1.18E-03	2.41E-03	-2.1538
ENSG00000124839	RAB17	1.1510	1.19E-03	2.42E-03	-2.1569
ENSG00000109424	UCP1	1.0187	1.19E-03	2.42E-03	-2.1577
ENSG00000124074	ENKD1	1.1754	1.19E-03	2.42E-03	-2.1577
ENSG00000189190	ZNF600	1.2772	1.19E-03	2.42E-03	-2.1575
ENSG00000156261	CCT8	1.1200	1.19E-03	2.42E-03	-2.1591
ENSG00000196116	TDRD7	1.1440	1.19E-03	2.43E-03	-2.1614
ENSG00000070047	PHRF1	1.1456	1.19E-03	2.43E-03	-2.1623
ENSG00000184735	DDX53	1.1599	1.20E-03	2.43E-03	-2.1628
ENSG00000158793	NIT1	1.1845	1.20E-03	2.44E-03	-2.1635
ENSG00000168143	FAM83B	1.0863	1.20E-03	2.44E-03	-2.1645
ENSG00000158571	PFKFB1	1.0883	1.20E-03	2.44E-03	-2.1665
ENSG00000146856	AGBL3	1.2476	1.21E-03	2.46E-03	-2.1716
ENSG00000174705	SH3PXD2B	1.0425	1.21E-03	2.46E-03	-2.1724
ENSG00000170558	CDH2	0.6402	1.21E-03	2.46E-03	-2.1751
ENSG00000173431	RNASE8	1.0107	1.21E-03	2.47E-03	-2.1774
ENSG00000186635	ARAP1	0.8698	1.21E-03	2.47E-03	-2.1773
ENSG00000108474	PIGL	1.1523	1.22E-03	2.47E-03	-2.1789
ENSG00000134897	BIVM	1.1929	1.22E-03	2.48E-03	-2.1817
ENSG00000141013	GAS8	1.2527	1.22E-03	2.48E-03	-2.1817
ENSG00000167081	PBX3	1.2115	1.22E-03	2.48E-03	-2.1818
ENSG00000110395	CBL	1.1812	1.22E-03	2.49E-03	-2.1846
ENSG00000128000	ZNF780B	0.8623	1.23E-03	2.49E-03	-2.1872
ENSG00000269067	ZNF728	1.0375	1.23E-03	2.50E-03	-2.1881
ENSG00000143457	GOLPH3L	0.7973	1.23E-03	2.50E-03	-2.1897
ENSG00000166961	MS4A15	0.8379	1.23E-03	2.50E-03	-2.1908
ENSG00000146678	IGFBP1	1.0428	1.23E-03	2.50E-03	-2.1911
ENSG00000129204	USP6	1.1371	1.23E-03	2.51E-03	-2.1926
ENSG00000136052	SLC41A2	1.1733	1.24E-03	2.51E-03	-2.1941
ENSG00000095713	CRTAC1	0.8967	1.24E-03	2.51E-03	-2.1947
ENSG00000102387	TAF7L	1.0764	1.24E-03	2.51E-03	-2.1948
ENSG00000136848	DAB2IP	0.7797	1.24E-03	2.51E-03	-2.1947
ENSG00000216588	IGSF23	0.9097	1.24E-03	2.51E-03	-2.1949
ENSG00000163515	RETNLB	1.0725	1.24E-03	2.52E-03	-2.1968
ENSG00000101161	PRPF6	1.1263	1.24E-03	2.52E-03	-2.1982
ENSG00000257743	MGAM2	0.9527	1.24E-03	2.52E-03	-2.1990
ENSG00000115657	ABCB6	1.2008	1.25E-03	2.53E-03	-2.2004
ENSG00000134152	KATNBL1	0.8721	1.25E-03	2.53E-03	-2.2010
ENSG00000139985	ADAM21	0.9553	1.25E-03	2.54E-03	-2.2045
ENSG00000061918	GUCY1B1	1.1073	1.25E-03	2.54E-03	-2.2055
ENSG00000262628	OR1D5	1.0074	1.25E-03	2.54E-03	-2.2057
ENSG00000161031	PGLYRP2	1.1120	1.25E-03	2.54E-03	-2.2058
ENSG00000136279	DBNL	0.8561	1.25E-03	2.54E-03	-2.2072
ENSG00000130038	CRACR2A	1.1694	1.25E-03	2.55E-03	-2.2077
ENSG00000115946	PNO1	1.1141	1.26E-03	2.55E-03	-2.2088
ENSG00000177352	CCDC71	0.8495	1.26E-03	2.55E-03	-2.2098
ENSG00000066379	POLR1H	0.8752	1.26E-03	2.55E-03	-2.2103
ENSG00000117228	GBP1	1.3871	1.26E-03	2.55E-03	-2.2112
ENSG00000121318	TAS2R10	0.9120	1.26E-03	2.56E-03	-2.2123
ENSG00000112110	MRPL18	0.8479	1.26E-03	2.56E-03	-2.2125

Gene ID	Gene Symbol	FC	P.Value	adj.P.Val	B
ENSG00000162104	ADCY9	1.2281	1.26E-03	2.56E-03	-2.2135
ENSG00000162623	TYW3	1.1112	1.27E-03	2.57E-03	-2.2164
ENSG00000196711	ALKAL1	1.0146	1.27E-03	2.57E-03	-2.2164
ENSG00000063515	GSC2	1.0106	1.27E-03	2.57E-03	-2.2184
ENSG00000113327	GABRG2	1.2482	1.27E-03	2.58E-03	-2.2214
ENSG00000182667	NTM	1.1434	1.28E-03	2.59E-03	-2.2237
ENSG00000159885	ZNF222	1.2003	1.28E-03	2.60E-03	-2.2284
ENSG00000185290	NUPR2	1.0365	1.29E-03	2.61E-03	-2.2315
ENSG00000005961	ITGA2B	1.2003	1.29E-03	2.61E-03	-2.2322
ENSG00000132361	CLUH	1.1632	1.29E-03	2.61E-03	-2.2330
ENSG00000184677	ZBTB40	1.1997	1.29E-03	2.62E-03	-2.2359
ENSG00000080709	KCNN2	1.0791	1.30E-03	2.63E-03	-2.2387
ENSG00000271321	CTAGE6	1.0527	1.30E-03	2.63E-03	-2.2402
ENSG00000198400	NTRK1	1.2061	1.30E-03	2.63E-03	-2.2410
ENSG00000138231	DBR1	1.1309	1.30E-03	2.64E-03	-2.2419
ENSG00000167685	ZNF444	0.8891	1.30E-03	2.64E-03	-2.2428
ENSG00000256525	POLG2	1.1991	1.30E-03	2.64E-03	-2.2431
ENSG00000183615	FAM167B	1.1671	1.31E-03	2.64E-03	-2.2448
ENSG00000058085	LAMC2	1.0203	1.31E-03	2.65E-03	-2.2467
ENSG00000120820	GLT8D2	1.1556	1.31E-03	2.65E-03	-2.2474
ENSG00000101463	SYNDIG1	1.2102	1.31E-03	2.65E-03	-2.2495
ENSG00000103227	LMF1	0.8065	1.31E-03	2.65E-03	-2.2494
ENSG00000138768	USO1	0.8444	1.31E-03	2.65E-03	-2.2495
ENSG00000086232	EIF2AK1	1.1354	1.31E-03	2.66E-03	-2.2504
ENSG00000181192	DHTKD1	1.1731	1.32E-03	2.66E-03	-2.2522
ENSG00000186326	RGS9BP	0.9847	1.32E-03	2.66E-03	-2.2525
ENSG00000139899	CBLN3	1.2708	1.32E-03	2.66E-03	-2.2533
ENSG00000214736	TOMM6	0.9065	1.32E-03	2.67E-03	-2.2548
ENSG00000169403	PTAFR	0.8278	1.33E-03	2.68E-03	-2.2601
ENSG00000054523	KIF1B	1.1520	1.33E-03	2.68E-03	-2.2606
ENSG00000153395	LPCAT1	0.8407	1.33E-03	2.69E-03	-2.2623
ENSG00000185585	OLFML2A	1.0816	1.33E-03	2.69E-03	-2.2628
ENSG00000111790	FGFR1OP2	1.1283	1.33E-03	2.70E-03	-2.2644
ENSG00000174564	IL20RB	1.1186	1.34E-03	2.70E-03	-2.2654
ENSG00000122674	CCZ1	1.1025	1.34E-03	2.70E-03	-2.2655
ENSG00000136634	IL10	1.2470	1.34E-03	2.70E-03	-2.2670
ENSG00000197162	ZNF785	0.8475	1.34E-03	2.70E-03	-2.2676
ENSG00000173762	CD7	1.1791	1.35E-03	2.72E-03	-2.2734
ENSG00000119509	INVS	1.1539	1.35E-03	2.72E-03	-2.2747
ENSG00000109083	IFT20	1.1289	1.35E-03	2.73E-03	-2.2755
ENSG00000151445	VIPAS39	1.1360	1.35E-03	2.73E-03	-2.2755
ENSG00000197712	FAM114A1	1.2214	1.35E-03	2.73E-03	-2.2759
ENSG00000197540	GZMM	1.1489	1.35E-03	2.73E-03	-2.2776
ENSG00000213965	NUDT19	1.1154	1.36E-03	2.74E-03	-2.2808
ENSG00000172382	PRSS27	1.1120	1.36E-03	2.74E-03	-2.2825
ENSG00000141378	PTRH2	0.8638	1.37E-03	2.76E-03	-2.2874
ENSG00000169855	ROBO1	1.3852	1.37E-03	2.77E-03	-2.2895
ENSG00000092096	SLC22A17	1.6636	1.37E-03	2.77E-03	-2.2911
ENSG00000136155	SCEL	0.9582	1.37E-03	2.77E-03	-2.2913
ENSG00000023902	PLEKHO1	1.5418	1.37E-03	2.77E-03	-2.2915
ENSG00000157111	TMEM171	0.7435	1.38E-03	2.77E-03	-2.2929
ENSG00000189007	ADAT2	1.2016	1.38E-03	2.77E-03	-2.2930
ENSG00000110921	MVK	1.1275	1.38E-03	2.78E-03	-2.2934
ENSG00000071537	SEL1L	1.2129	1.38E-03	2.78E-03	-2.2935

Gene ID	Gene Symbol	FC	P.Value	adj.P.Val	B
ENSG00000184788	SATL1	1.1025	1.38E-03	2.78E-03	-2.2937
ENSG00000188800	TMCO2	1.0496	1.38E-03	2.78E-03	-2.2948
ENSG00000135517	MIP	1.0302	1.38E-03	2.78E-03	-2.2956
ENSG00000179041	RRS1	0.8395	1.38E-03	2.79E-03	-2.2976
ENSG00000261052	SULT1A3	1.2103	1.39E-03	2.79E-03	-2.2998
ENSG00000151876	FBXO4	0.8730	1.39E-03	2.80E-03	-2.3030
ENSG00000130508	PXDN	1.5486	1.39E-03	2.81E-03	-2.3053
ENSG00000079308	TNS1	1.1598	1.40E-03	2.81E-03	-2.3061
ENSG00000134588	USP26	1.0731	1.40E-03	2.81E-03	-2.3071
ENSG00000132423	COQ3	0.8509	1.40E-03	2.81E-03	-2.3076
ENSG00000197774	EME2	1.1618	1.41E-03	2.83E-03	-2.3121
ENSG00000221988	PPT2	0.7630	1.41E-03	2.83E-03	-2.3129
ENSG00000233954	UQCRHL	0.8968	1.41E-03	2.84E-03	-2.3145
ENSG00000169116	PARM1	1.3441	1.41E-03	2.84E-03	-2.3175
ENSG00000140553	UNC45A	0.8894	1.41E-03	2.84E-03	-2.3177
ENSG00000177058	SLC38A9	0.8840	1.41E-03	2.85E-03	-2.3183
ENSG00000155304	HSPA13	1.1653	1.42E-03	2.86E-03	-2.3221
ENSG00000148835	TAF5	1.1168	1.43E-03	2.87E-03	-2.3254
ENSG00000139292	LGR5	1.0185	1.43E-03	2.87E-03	-2.3275
ENSG00000112186	CAP2	1.0189	1.43E-03	2.88E-03	-2.3287
ENSG00000204624	DISP3	1.0953	1.43E-03	2.88E-03	-2.3303
ENSG00000156110	ADK	1.1112	1.43E-03	2.88E-03	-2.3305
ENSG00000126254	RBM42	1.1166	1.43E-03	2.88E-03	-2.3308
ENSG00000241468	ATP5MF	0.8906	1.44E-03	2.89E-03	-2.3326
ENSG00000165799	RNASE7	1.0145	1.44E-03	2.89E-03	-2.3338
ENSG00000121270	ABCC11	1.0659	1.44E-03	2.89E-03	-2.3349
ENSG00000198326	TMEM239	1.0120	1.44E-03	2.90E-03	-2.3361
ENSG00000186272	ZNF17	0.8766	1.45E-03	2.91E-03	-2.3385
ENSG00000154743	TSEN2	0.8508	1.45E-03	2.91E-03	-2.3407
ENSG00000164062	APEH	0.8645	1.45E-03	2.91E-03	-2.3409
ENSG00000062716	VMP1	1.1971	1.45E-03	2.91E-03	-2.3411
ENSG00000061987	MON2	1.1551	1.45E-03	2.92E-03	-2.3428
ENSG00000177954	RPS27	0.8867	1.46E-03	2.93E-03	-2.3467
ENSG00000187475	H1-6	1.1087	1.46E-03	2.94E-03	-2.3491
ENSG00000167397	VKORC1	0.8426	1.46E-03	2.94E-03	-2.3495
ENSG00000009790	TRAF3IP3	1.4174	1.47E-03	2.95E-03	-2.3529
ENSG00000203811	H3C14	1.2726	1.47E-03	2.95E-03	-2.3530
ENSG00000183067	IGSF5	1.0626	1.47E-03	2.95E-03	-2.3539
ENSG00000160325	CACFD1	1.1654	1.47E-03	2.95E-03	-2.3540
ENSG00000049239	H6PD	1.1589	1.47E-03	2.95E-03	-2.3544
ENSG00000197768	STPG3	1.1450	1.47E-03	2.96E-03	-2.3557
ENSG00000172831	CES2	1.1484	1.48E-03	2.96E-03	-2.3569
ENSG00000154162	CDH12	1.0860	1.48E-03	2.96E-03	-2.3580
ENSG00000162645	GBP2	1.4560	1.48E-03	2.96E-03	-2.3583
ENSG00000113248	PCDHB15	1.1012	1.48E-03	2.97E-03	-2.3590
ENSG00000127561	SYNGR3	1.3521	1.48E-03	2.97E-03	-2.3591
ENSG00000025708	TYMP	1.3188	1.48E-03	2.97E-03	-2.3594
ENSG00000166682	TMPRSS5	1.1666	1.48E-03	2.97E-03	-2.3594
ENSG00000140323	DISP2	0.8621	1.48E-03	2.97E-03	-2.3616
ENSG00000127663	KDM4B	1.1217	1.48E-03	2.97E-03	-2.3618
ENSG00000115386	REG1A	1.1158	1.48E-03	2.97E-03	-2.3625
ENSG00000170190	SLC16A5	1.1900	1.49E-03	2.98E-03	-2.3631
ENSG00000104427	ZC2HC1A	1.1913	1.49E-03	2.98E-03	-2.3651
ENSG00000116199	FAM20B	1.2018	1.49E-03	2.98E-03	-2.3655

Gene ID	Gene Symbol	FC	P.Value	adj.P.Val	B
ENSG00000186470	BTN3A2	0.7867	1.49E-03	2.99E-03	-2.3667
ENSG00000106443	PHF14	0.8484	1.49E-03	2.99E-03	-2.3671
ENSG00000169918	OTUD7A	1.1093	1.49E-03	2.99E-03	-2.3678
ENSG00000014257	ACP3	1.1692	1.50E-03	3.01E-03	-2.3727
ENSG00000153029	MR1	1.1666	1.50E-03	3.01E-03	-2.3728
ENSG00000111639	MRPL51	0.8646	1.50E-03	3.01E-03	-2.3736
ENSG00000171094	ALK	1.0446	1.50E-03	3.01E-03	-2.3745
ENSG00000154065	ANKRD29	1.1838	1.51E-03	3.02E-03	-2.3768
ENSG00000132478	UNK	1.1335	1.51E-03	3.02E-03	-2.3777
ENSG00000090975	PITPNM2	1.1969	1.51E-03	3.02E-03	-2.3779
ENSG00000114956	DGUOK	1.0939	1.51E-03	3.03E-03	-2.3810
ENSG00000160679	CHTOP	1.1651	1.52E-03	3.03E-03	-2.3816
ENSG00000173728	C1orf100	1.1256	1.52E-03	3.03E-03	-2.3819
ENSG00000120656	TAF12	0.8778	1.52E-03	3.04E-03	-2.3838
ENSG00000166323	C11orf65	0.8750	1.52E-03	3.04E-03	-2.3848
ENSG00000164615	CAMLG	0.8843	1.52E-03	3.05E-03	-2.3863
ENSG00000136457	CHAD	1.1102	1.53E-03	3.06E-03	-2.3910
ENSG00000205476	CCDC85C	1.1712	1.53E-03	3.06E-03	-2.3916
ENSG00000122254	HS3ST2	0.8494	1.53E-03	3.07E-03	-2.3925
ENSG00000155957	TMBIM4	0.8931	1.54E-03	3.07E-03	-2.3937
ENSG00000198538	ZNF28	1.2164	1.54E-03	3.07E-03	-2.3938
ENSG00000160318	CLDND2	1.2598	1.55E-03	3.09E-03	-2.4006
ENSG00000006576	PHTF2	1.1655	1.55E-03	3.11E-03	-2.4053
ENSG00000133619	KRBA1	1.1423	1.56E-03	3.12E-03	-2.4094
ENSG00000081041	CXCL2	1.3541	1.56E-03	3.12E-03	-2.4108
ENSG00000166450	PRTG	1.0121	1.56E-03	3.12E-03	-2.4108
ENSG00000111729	CLEC4A	0.8619	1.57E-03	3.13E-03	-2.4134
ENSG00000181374	CCL13	0.8823	1.57E-03	3.13E-03	-2.4136
ENSG00000170604	IRF2BP1	0.8521	1.57E-03	3.14E-03	-2.4142
ENSG00000180357	ZNF609	1.1526	1.57E-03	3.14E-03	-2.4160
ENSG00000178796	RIIAD1	1.0397	1.57E-03	3.14E-03	-2.4166
ENSG00000108878	CACNG1	0.9723	1.57E-03	3.14E-03	-2.4168
ENSG00000166289	PLEKHF1	1.2915	1.58E-03	3.15E-03	-2.4201
ENSG00000182986	ZNF320	1.3035	1.58E-03	3.15E-03	-2.4201
ENSG00000104691	UBXN8	0.8675	1.59E-03	3.17E-03	-2.4239
ENSG00000130724	CHMP2A	0.9113	1.59E-03	3.17E-03	-2.4264
ENSG00000124370	MCEE	0.8868	1.59E-03	3.18E-03	-2.4274
ENSG00000145734	BDP1	1.1271	1.60E-03	3.18E-03	-2.4292
ENSG00000100503	NIN	1.2100	1.60E-03	3.19E-03	-2.4300
ENSG00000174197	MGA	1.1899	1.60E-03	3.19E-03	-2.4301
ENSG00000184983	NDUFA6	1.1243	1.60E-03	3.19E-03	-2.4315
ENSG00000185085	INTS5	0.8596	1.60E-03	3.19E-03	-2.4315
ENSG00000204851	PNMA8B	0.9661	1.60E-03	3.20E-03	-2.4341
ENSG00000107566	ERLIN1	0.8459	1.60E-03	3.20E-03	-2.4346
ENSG00000172232	AZU1	1.4292	1.61E-03	3.20E-03	-2.4349
ENSG00000204438	GPANK1	0.8821	1.61E-03	3.21E-03	-2.4368
ENSG00000002745	WNT16	1.2637	1.61E-03	3.21E-03	-2.4385
ENSG00000179869	ABCA13	1.0864	1.61E-03	3.22E-03	-2.4395
ENSG00000138166	DUSP5	1.2759	1.62E-03	3.22E-03	-2.4416
ENSG00000041802	LSG1	1.1549	1.62E-03	3.22E-03	-2.4419
ENSG00000197705	KLHL14	0.6348	1.62E-03	3.23E-03	-2.4443
ENSG00000226430	USP17L7	1.0127	1.63E-03	3.24E-03	-2.4465
ENSG00000180767	CHST13	0.8944	1.63E-03	3.25E-03	-2.4483
ENSG00000074800	ENO1	1.1618	1.64E-03	3.27E-03	-2.4551

Gene ID	Gene Symbol	FC	P.Value	adj.P.Val	B
ENSG00000186891	TNFRSF18	1.6959	1.65E-03	3.28E-03	-2.4579
ENSG00000099954	CECR2	1.3077	1.65E-03	3.28E-03	-2.4583
ENSG00000185385	OR7A17	1.0430	1.65E-03	3.28E-03	-2.4587
ENSG00000205060	SLC35B4	1.1941	1.65E-03	3.29E-03	-2.4603
ENSG00000163879	DNALI1	1.0528	1.65E-03	3.30E-03	-2.4628
ENSG00000177889	UBE2N	1.1158	1.66E-03	3.30E-03	-2.4635
ENSG00000213462	ERV3-1	1.1705	1.66E-03	3.30E-03	-2.4646
ENSG00000187772	LIN28B	1.0768	1.66E-03	3.30E-03	-2.4650
ENSG00000064195	DLX3	1.0461	1.66E-03	3.31E-03	-2.4680
ENSG00000011376	LARS2	1.1381	1.66E-03	3.31E-03	-2.4682
ENSG00000088854	DNAAF9	1.1396	1.67E-03	3.31E-03	-2.4689
ENSG00000170290	SLN	1.0537	1.67E-03	3.32E-03	-2.4693
ENSG00000166947	EPB42	1.1497	1.67E-03	3.32E-03	-2.4697
ENSG00000113088	GZMK	0.8782	1.67E-03	3.32E-03	-2.4709
ENSG00000150275	PCDH15	1.0795	1.67E-03	3.32E-03	-2.4709
ENSG00000181790	ADGRB1	1.1258	1.67E-03	3.32E-03	-2.4709
ENSG00000106605	BLVRA	1.3019	1.67E-03	3.33E-03	-2.4732
ENSG00000185324	CDK10	1.2035	1.67E-03	3.33E-03	-2.4733
ENSG00000101958	GLRA2	1.0116	1.67E-03	3.33E-03	-2.4737
ENSG00000104213	PDGFRL	1.1309	1.68E-03	3.33E-03	-2.4751
ENSG00000120937	NPPB	1.0199	1.68E-03	3.34E-03	-2.4755
ENSG00000197008	ZNF138	0.8610	1.68E-03	3.34E-03	-2.4776
ENSG00000115594	IL1R1	1.1660	1.68E-03	3.34E-03	-2.4782
ENSG00000203760	CENPW	1.2427	1.69E-03	3.35E-03	-2.4809
ENSG00000146005	PSD2	1.0993	1.70E-03	3.37E-03	-2.4855
ENSG00000123999	INHA	1.0354	1.70E-03	3.37E-03	-2.4857
ENSG00000079785	DDX1	1.1265	1.70E-03	3.37E-03	-2.4864
ENSG00000188517	COL25A1	1.0527	1.70E-03	3.38E-03	-2.4889
ENSG00000157657	ZNF618	1.1440	1.71E-03	3.39E-03	-2.4906
ENSG00000183034	OTOP2	1.0102	1.71E-03	3.39E-03	-2.4925
ENSG00000079841	RIMS1	1.1579	1.72E-03	3.41E-03	-2.4962
ENSG00000099910	KLHL22	1.1585	1.72E-03	3.41E-03	-2.4963
ENSG00000165209	STRBP	1.1569	1.72E-03	3.41E-03	-2.4971
ENSG00000124702	KLHDC3	1.1426	1.72E-03	3.42E-03	-2.5002
ENSG00000132514	CLEC10A	0.9262	1.72E-03	3.42E-03	-2.5007
ENSG00000167037	SGSM1	1.0569	1.73E-03	3.42E-03	-2.5013
ENSG00000124260	MAGEA10	1.0892	1.73E-03	3.43E-03	-2.5024
ENSG00000050820	BCAR1	1.2308	1.73E-03	3.43E-03	-2.5025
ENSG00000169093	ASMTL	1.1408	1.73E-03	3.43E-03	-2.5038
ENSG00000163531	NFASC	1.1000	1.74E-03	3.46E-03	-2.5099
ENSG00000196636	SDHAF3	0.7562	1.74E-03	3.46E-03	-2.5107
ENSG00000131475	VPS25	1.1302	1.75E-03	3.48E-03	-2.5159
ENSG00000170954	ZNF415	1.1745	1.76E-03	3.48E-03	-2.5172
ENSG00000214338	SOGA3	1.1388	1.76E-03	3.48E-03	-2.5173
ENSG00000241935	HOGA1	0.8578	1.76E-03	3.48E-03	-2.5181
ENSG00000171843	MLLT3	0.7782	1.77E-03	3.50E-03	-2.5236
ENSG00000176463	SLCO3A1	1.4896	1.78E-03	3.52E-03	-2.5282
ENSG00000135272	MDFIC	1.1478	1.78E-03	3.53E-03	-2.5315
ENSG00000185899	TAS2R60	1.0131	1.78E-03	3.53E-03	-2.5315
ENSG00000066382	MPPED2	1.2971	1.79E-03	3.54E-03	-2.5326
ENSG00000004139	SARM1	1.2065	1.79E-03	3.54E-03	-2.5335
ENSG00000155254	MARVELD1	1.2765	1.79E-03	3.54E-03	-2.5334
ENSG00000156603	MED19	0.8806	1.80E-03	3.56E-03	-2.5386
ENSG00000158850	B4GALT3	1.1731	1.80E-03	3.56E-03	-2.5393

Gene ID	Gene Symbol	FC	P.Value	adj.P.Val	B
ENSG00000243989	ACY1	0.8516	1.80E-03	3.57E-03	-2.5408
ENSG00000258484	SPESP1	1.2041	1.80E-03	3.57E-03	-2.5417
ENSG00000134910	STT3A	0.8381	1.80E-03	3.57E-03	-2.5424
ENSG00000189280	GJB5	0.9595	1.81E-03	3.59E-03	-2.5456
ENSG00000111640	GAPDH	1.1506	1.82E-03	3.60E-03	-2.5502
ENSG00000197497	ZNF665	1.1961	1.82E-03	3.60E-03	-2.5507
ENSG00000183570	PCBP3	1.3027	1.83E-03	3.62E-03	-2.5538
ENSG00000164543	STK17A	1.2439	1.84E-03	3.63E-03	-2.5579
ENSG00000196793	ZNF239	1.1270	1.84E-03	3.63E-03	-2.5581
ENSG00000154258	ABCA9	1.1705	1.84E-03	3.64E-03	-2.5592
ENSG00000114670	NEK11	1.1824	1.84E-03	3.64E-03	-2.5601
ENSG00000189369	GSPT2	1.1717	1.84E-03	3.65E-03	-2.5621
ENSG00000152591	DSPP	1.0107	1.85E-03	3.66E-03	-2.5666
ENSG00000104047	DTWD1	0.8830	1.85E-03	3.67E-03	-2.5670
ENSG00000128011	LRFN1	1.0923	1.86E-03	3.67E-03	-2.5677
ENSG00000103356	EARS2	0.8453	1.86E-03	3.68E-03	-2.5714
ENSG00000072958	AP1M1	1.1273	1.86E-03	3.69E-03	-2.5726
ENSG00000134532	SOX5	1.1673	1.87E-03	3.69E-03	-2.5728
ENSG00000078808	SDF4	1.1312	1.87E-03	3.69E-03	-2.5742
ENSG00000153086	ACMSD	1.0495	1.88E-03	3.72E-03	-2.5806
ENSG00000071994	PDCD2	0.8989	1.88E-03	3.72E-03	-2.5812
ENSG00000172508	CARNS1	1.2004	1.88E-03	3.72E-03	-2.5817
ENSG00000179902	C1orf194	1.0698	1.89E-03	3.74E-03	-2.5862
ENSG00000142102	PGGHG	1.2284	1.90E-03	3.75E-03	-2.5887
ENSG00000113583	C5orf15	1.1119	1.90E-03	3.76E-03	-2.5910
ENSG00000178105	DDX10	0.8716	1.91E-03	3.76E-03	-2.5925
ENSG00000138614	INTS14	1.0997	1.91E-03	3.78E-03	-2.5961
ENSG00000172399	MYOZ2	1.0086	1.92E-03	3.79E-03	-2.5983
ENSG00000083635	NUFIP1	1.0954	1.92E-03	3.79E-03	-2.5991
ENSG00000115271	GCA	1.1986	1.92E-03	3.79E-03	-2.6002
ENSG00000137142	IGFBPL1	1.0966	1.92E-03	3.80E-03	-2.6005
ENSG00000251247	ZNF345	1.1469	1.92E-03	3.80E-03	-2.6008
ENSG00000177030	DEAF1	1.1356	1.93E-03	3.80E-03	-2.6023
ENSG00000215788	TNFRSF25	1.2215	1.93E-03	3.81E-03	-2.6035
ENSG00000168874	ATOH8	1.0356	1.93E-03	3.81E-03	-2.6038
ENSG00000103187	COTL1	1.4604	1.93E-03	3.81E-03	-2.6045
ENSG00000137460	FHDC1	0.8718	1.94E-03	3.83E-03	-2.6089
ENSG00000204149	AGAP6	1.1465	1.94E-03	3.83E-03	-2.6091
ENSG00000133636	NTS	1.0659	1.94E-03	3.83E-03	-2.6094
ENSG00000112977	DAP	1.1177	1.94E-03	3.83E-03	-2.6096
ENSG00000156136	DCK	1.2463	1.94E-03	3.83E-03	-2.6100
ENSG00000134539	KLRD1	1.1066	1.95E-03	3.85E-03	-2.6140
ENSG00000164897	TMUB1	1.1079	1.95E-03	3.85E-03	-2.6142
ENSG00000182240	BACE2	1.2837	1.96E-03	3.86E-03	-2.6168
ENSG00000104872	PIH1D1	1.1064	1.96E-03	3.87E-03	-2.6191
ENSG00000165584	SSX3	1.0267	1.96E-03	3.87E-03	-2.6199
ENSG00000049246	PER3	1.1852	1.97E-03	3.88E-03	-2.6218
ENSG00000115085	ZAP70	1.2985	1.97E-03	3.88E-03	-2.6228
ENSG00000206013	IFITM5	1.1451	1.97E-03	3.88E-03	-2.6236
ENSG00000102349	KLF8	1.2432	1.97E-03	3.89E-03	-2.6252
ENSG00000148411	NACC2	1.2278	1.97E-03	3.89E-03	-2.6252
ENSG00000116882	HAO2	0.9617	1.98E-03	3.90E-03	-2.6270
ENSG00000180354	MTURN	1.2166	1.98E-03	3.91E-03	-2.6289
ENSG00000103310	ZP2	1.0351	1.98E-03	3.91E-03	-2.6291

Gene ID	Gene Symbol	FC	P.Value	adj.P.Val	B
ENSG00000153930	ANKFN1	1.1487	1.99E-03	3.91E-03	-2.6307
ENSG00000250021	ARPIN-AP3S2	0.8656	1.99E-03	3.91E-03	-2.6310
ENSG00000169740	ZNF32	1.1889	1.99E-03	3.93E-03	-2.6340
ENSG00000013275	PSMC4	1.1048	2.00E-03	3.93E-03	-2.6355
ENSG00000105619	TFPT	0.8954	2.00E-03	3.94E-03	-2.6372
ENSG00000187097	ENTPD5	1.1325	2.00E-03	3.95E-03	-2.6389
ENSG00000120278	PLEKHG1	1.1469	2.01E-03	3.95E-03	-2.6407
ENSG00000125166	GOT2	1.1449	2.01E-03	3.95E-03	-2.6411
ENSG00000102230	PCYT1B	1.0141	2.01E-03	3.96E-03	-2.6417
ENSG00000144635	DYNC1LI1	1.1095	2.01E-03	3.96E-03	-2.6422
ENSG00000165269	AQP7	1.1219	2.01E-03	3.96E-03	-2.6427
ENSG00000105221	AKT2	1.1061	2.02E-03	3.96E-03	-2.6439
ENSG00000232119	MCTS1	0.8699	2.02E-03	3.97E-03	-2.6451
ENSG00000178700	DHFR2	0.8443	2.02E-03	3.97E-03	-2.6452
ENSG00000118046	STK11	0.8857	2.03E-03	3.99E-03	-2.6503
ENSG00000172987	HPSE2	1.0630	2.03E-03	3.99E-03	-2.6511
ENSG00000161860	SYCE2	1.1682	2.03E-03	4.00E-03	-2.6515
ENSG00000157379	DHRS1	1.1230	2.03E-03	4.00E-03	-2.6520
ENSG00000188710	QRFP	0.9367	2.04E-03	4.00E-03	-2.6528
ENSG00000123453	SARDH	1.3244	2.04E-03	4.00E-03	-2.6533
ENSG00000170266	GLB1	0.8725	2.04E-03	4.01E-03	-2.6546
ENSG00000197454	OR2L5	1.0073	2.04E-03	4.01E-03	-2.6552
ENSG00000138678	GPAT3	1.1503	2.04E-03	4.01E-03	-2.6565
ENSG00000198805	PNP	1.3154	2.04E-03	4.02E-03	-2.6572
ENSG00000104941	RSPH6A	1.0092	2.05E-03	4.03E-03	-2.6596
ENSG00000184276	DEFB108B	1.0241	2.06E-03	4.04E-03	-2.6620
ENSG00000154330	PGM5	1.2146	2.06E-03	4.04E-03	-2.6625
ENSG00000215397	SCRT2	0.9878	2.06E-03	4.04E-03	-2.6631
ENSG00000132825	PPP1R3D	1.1423	2.06E-03	4.04E-03	-2.6632
ENSG00000167065	DUSP18	1.1118	2.06E-03	4.05E-03	-2.6655
ENSG00000253873	PCDHGA11	0.8602	2.06E-03	4.05E-03	-2.6655
ENSG00000182450	KCNK4	1.0225	2.07E-03	4.05E-03	-2.6663
ENSG00000112208	BAG2	0.8183	2.07E-03	4.06E-03	-2.6667
ENSG00000227051	C14orf132	1.0926	2.07E-03	4.06E-03	-2.6669
ENSG00000183230	CTNNA3	1.2634	2.07E-03	4.06E-03	-2.6671
ENSG00000166704	ZNF606	1.2105	2.07E-03	4.07E-03	-2.6699
ENSG00000106246	PTCD1	1.1204	2.08E-03	4.07E-03	-2.6712
ENSG00000082074	FYB1	0.8018	2.08E-03	4.08E-03	-2.6721
ENSG00000159322	ADPGK	1.0995	2.08E-03	4.08E-03	-2.6726
ENSG00000260916	CCPG1	0.8683	2.08E-03	4.08E-03	-2.6725
ENSG00000244219	TMEM225B	0.8790	2.08E-03	4.08E-03	-2.6727
ENSG00000071991	CDH19	1.0801	2.09E-03	4.10E-03	-2.6766
ENSG00000077312	SNRPA	1.1130	2.09E-03	4.10E-03	-2.6778
ENSG00000181085	MAPK15	1.1455	2.09E-03	4.10E-03	-2.6781
ENSG00000140326	CDAN1	1.1340	2.10E-03	4.11E-03	-2.6797
ENSG00000066926	FECH	1.1593	2.10E-03	4.12E-03	-2.6817
ENSG00000134627	PIWIL4	1.1267	2.10E-03	4.12E-03	-2.6819
ENSG00000253309	SERPINE3	1.0833	2.10E-03	4.12E-03	-2.6822
ENSG00000013523	ANGEL1	0.8377	2.10E-03	4.12E-03	-2.6829
ENSG00000164818	DNAAF5	1.1362	2.11E-03	4.13E-03	-2.6852
ENSG00000152910	CNTNAP4	1.1388	2.12E-03	4.15E-03	-2.6885
ENSG00000109736	MFSD10	1.1666	2.12E-03	4.15E-03	-2.6897
ENSG00000255524	NPIPB8	1.0948	2.12E-03	4.16E-03	-2.6910
ENSG00000100196	KDELR3	0.7214	2.13E-03	4.18E-03	-2.6968

Gene ID	Gene Symbol	FC	P.Value	adj.P.Val	B
ENSG00000163576	EFHB	1.1112	2.14E-03	4.18E-03	-2.6970
ENSG00000131668	BARX1	1.0135	2.14E-03	4.19E-03	-2.6978
ENSG00000100983	GSS	1.1427	2.15E-03	4.21E-03	-2.7038
ENSG00000213341	CHUK	1.1147	2.15E-03	4.22E-03	-2.7045
ENSG00000167380	ZNF226	0.8572	2.15E-03	4.22E-03	-2.7049
ENSG00000133169	BEX1	1.2289	2.16E-03	4.22E-03	-2.7058
ENSG00000160396	HIPK4	1.0318	2.16E-03	4.22E-03	-2.7067
ENSG00000174672	BRSK2	0.8934	2.16E-03	4.23E-03	-2.7076
ENSG00000260001	TGFBR3L	1.3496	2.17E-03	4.24E-03	-2.7101
ENSG00000088888	MAVS	1.1654	2.19E-03	4.28E-03	-2.7187
ENSG00000169306	IL1RAPL1	1.0084	2.19E-03	4.28E-03	-2.7194
ENSG00000007376	RPUSD1	1.1118	2.19E-03	4.29E-03	-2.7208
ENSG00000203783	PRR9	1.0079	2.19E-03	4.29E-03	-2.7211
ENSG00000103353	UBFD1	1.1624	2.19E-03	4.29E-03	-2.7215
ENSG00000144867	SRPRB	0.8848	2.19E-03	4.29E-03	-2.7220
ENSG00000080031	PTPRH	1.0329	2.20E-03	4.29E-03	-2.7223
ENSG00000189221	MAOA	1.0948	2.20E-03	4.29E-03	-2.7223
ENSG00000167910	CYP7A1	1.0050	2.20E-03	4.29E-03	-2.7231
ENSG00000168216	LMBRD1	1.1330	2.20E-03	4.30E-03	-2.7243
ENSG00000198681	MAGEA1	1.4159	2.21E-03	4.32E-03	-2.7284
ENSG00000172345	STARD5	0.8087	2.22E-03	4.33E-03	-2.7305
ENSG00000115295	CLIP4	1.4186	2.22E-03	4.34E-03	-2.7322
ENSG00000183840	GPR39	1.0159	2.22E-03	4.34E-03	-2.7334
ENSG00000214050	FBXO16	0.8314	2.23E-03	4.37E-03	-2.7387
ENSG00000189144	ZNF573	1.1995	2.24E-03	4.37E-03	-2.7393
ENSG00000144909	OSBPL11	1.1257	2.24E-03	4.37E-03	-2.7399
ENSG00000244694	PTCHD4	1.0663	2.24E-03	4.38E-03	-2.7409
ENSG00000107404	DVL1	1.1248	2.24E-03	4.38E-03	-2.7421
ENSG00000135446	CDK4	1.1139	2.24E-03	4.38E-03	-2.7423
ENSG00000187510	PLEKHG7	1.2372	2.25E-03	4.38E-03	-2.7430
ENSG00000173376	NDNF	1.4758	2.25E-03	4.39E-03	-2.7437
ENSG00000189334	S100A14	1.0695	2.25E-03	4.39E-03	-2.7438
ENSG00000110057	UNC93B1	0.8475	2.25E-03	4.39E-03	-2.7455
ENSG00000102466	FGF14	0.8222	2.25E-03	4.40E-03	-2.7462
ENSG00000175874	CREG2	1.1086	2.25E-03	4.40E-03	-2.7465
ENSG00000180611	MB21D2	1.3589	2.26E-03	4.41E-03	-2.7481
ENSG00000177558	FAM187B	1.0535	2.26E-03	4.41E-03	-2.7488
ENSG00000147509	RGS20	1.1850	2.26E-03	4.41E-03	-2.7504
ENSG00000162188	GNG3	1.0875	2.26E-03	4.41E-03	-2.7504
ENSG00000196218	RYR1	1.1937	2.26E-03	4.42E-03	-2.7507
ENSG00000198133	TMEM229B	1.1319	2.27E-03	4.42E-03	-2.7516
ENSG00000131951	LRRC9	1.0394	2.27E-03	4.42E-03	-2.7520
ENSG00000160505	NLRP4	1.1033	2.27E-03	4.43E-03	-2.7529
ENSG00000136100	VPS36	0.8626	2.27E-03	4.43E-03	-2.7533
ENSG00000197915	HRNR	1.0187	2.27E-03	4.43E-03	-2.7543
ENSG00000155011	DKK2	0.9789	2.27E-03	4.43E-03	-2.7546
ENSG00000092439	TRPM7	1.1207	2.28E-03	4.44E-03	-2.7568
ENSG00000082556	OPRK1	1.0442	2.28E-03	4.44E-03	-2.7571
ENSG00000103811	CTSH	1.4858	2.28E-03	4.44E-03	-2.7573
ENSG00000152234	ATP5F1A	0.9072	2.28E-03	4.45E-03	-2.7581
ENSG00000132874	SLC14A2	1.1089	2.29E-03	4.45E-03	-2.7591
ENSG00000030582	GRN	0.8157	2.29E-03	4.46E-03	-2.7618
ENSG00000085514	PILRA	0.8223	2.29E-03	4.46E-03	-2.7619
ENSG00000071462	BUD23	1.1341	2.30E-03	4.48E-03	-2.7648

Gene ID	Gene Symbol	FC	P.Value	adj.P.Val	B
ENSG00000205423	CNEP1R1	1.1444	2.31E-03	4.50E-03	-2.7702
ENSG00000185040	SPDYE16	1.0237	2.32E-03	4.52E-03	-2.7727
ENSG00000175110	MRPS22	1.0884	2.32E-03	4.52E-03	-2.7736
ENSG00000118961	LDAH	0.8234	2.32E-03	4.52E-03	-2.7746
ENSG00000170516	COX7B2	1.2711	2.33E-03	4.53E-03	-2.7757
ENSG00000184384	MAML2	1.2346	2.33E-03	4.54E-03	-2.7786
ENSG00000115484	CCT4	1.1053	2.34E-03	4.55E-03	-2.7794
ENSG00000177508	IRX3	1.0572	2.35E-03	4.56E-03	-2.7832
ENSG00000178338	ZNF354B	1.2606	2.35E-03	4.57E-03	-2.7834
ENSG00000147676	MAL2	1.2621	2.35E-03	4.58E-03	-2.7858
ENSG00000267508	ZNF285	1.1057	2.35E-03	4.58E-03	-2.7857
ENSG00000145721	LIX1	1.0565	2.36E-03	4.58E-03	-2.7874
ENSG00000213648	SULT1A4	1.2549	2.36E-03	4.59E-03	-2.7880
ENSG00000206432	TMEM200C	1.0163	2.36E-03	4.59E-03	-2.7890
ENSG00000178201	VN1R1	0.8518	2.36E-03	4.59E-03	-2.7899
ENSG00000182732	RGS6	1.1238	2.37E-03	4.60E-03	-2.7919
ENSG00000185972	CCIN	1.0171	2.38E-03	4.63E-03	-2.7961
ENSG00000159167	STC1	1.0456	2.38E-03	4.64E-03	-2.7983
ENSG00000103145	HCFC1R1	1.1379	2.39E-03	4.64E-03	-2.7987
ENSG00000152078	TLCD4	0.7960	2.39E-03	4.64E-03	-2.7988
ENSG00000148288	GBGT1	0.8065	2.39E-03	4.64E-03	-2.7992
ENSG00000177707	NECTIN3	1.3458	2.39E-03	4.64E-03	-2.7995
ENSG00000184451	CCR10	1.4391	2.39E-03	4.64E-03	-2.8001
ENSG00000133818	RRAS2	1.5511	2.39E-03	4.64E-03	-2.8006
ENSG00000108561	C1QBP	0.8593	2.39E-03	4.65E-03	-2.8008
ENSG00000243364	EFNA4	0.8534	2.39E-03	4.65E-03	-2.8012
ENSG00000177191	B3GNT8	0.8786	2.40E-03	4.65E-03	-2.8026
ENSG00000183117	CSMD1	1.1332	2.40E-03	4.66E-03	-2.8044
ENSG00000170348	TMED10	1.1474	2.40E-03	4.67E-03	-2.8059
ENSG00000122687	MRM2	0.8685	2.41E-03	4.67E-03	-2.8069
ENSG00000130203	APOE	0.6317	2.41E-03	4.68E-03	-2.8083
ENSG00000172765	TMCC1	1.1064	2.42E-03	4.69E-03	-2.8100
ENSG00000242612	DECR2	1.1884	2.42E-03	4.69E-03	-2.8102
ENSG00000165794	SLC39A2	1.0159	2.42E-03	4.70E-03	-2.8119
ENSG00000179941	BBS10	0.8290	2.43E-03	4.72E-03	-2.8154
ENSG00000162961	DPY30	0.8542	2.43E-03	4.72E-03	-2.8160
ENSG00000110660	SLC35F2	1.1887	2.45E-03	4.76E-03	-2.8240
ENSG00000105669	COPE	0.9052	2.46E-03	4.77E-03	-2.8255
ENSG00000175229	GAL3ST3	1.1277	2.46E-03	4.77E-03	-2.8254
ENSG00000147408	CSGALNACT1	1.3297	2.46E-03	4.77E-03	-2.8266
ENSG00000111653	ING4	0.8544	2.46E-03	4.77E-03	-2.8272
ENSG00000182263	FIGN	1.2172	2.46E-03	4.77E-03	-2.8271
ENSG00000186628	FSD2	1.0711	2.47E-03	4.78E-03	-2.8287
ENSG00000140937	CDH11	1.1163	2.47E-03	4.78E-03	-2.8295
ENSG00000105607	GCDH	0.8556	2.47E-03	4.79E-03	-2.8300
ENSG00000127824	TUBA4A	1.1582	2.47E-03	4.79E-03	-2.8309
ENSG00000023191	RNH1	1.1109	2.47E-03	4.79E-03	-2.8314
ENSG00000170561	IRX2	1.0229	2.48E-03	4.80E-03	-2.8329
ENSG00000099957	P2RX6	1.0768	2.48E-03	4.80E-03	-2.8337
ENSG00000197961	ZNF121	1.1674	2.49E-03	4.82E-03	-2.8369
ENSG00000165841	CYP2C19	1.0160	2.49E-03	4.82E-03	-2.8373
ENSG00000136250	AOAH	0.8093	2.49E-03	4.82E-03	-2.8376
ENSG00000119608	PROX2	0.9302	2.50E-03	4.83E-03	-2.8396
ENSG00000088451	TGDS	0.8577	2.50E-03	4.84E-03	-2.8419

Gene ID	Gene Symbol	FC	P.Value	adj.P.Val	B
ENSG00000163577	EIF5A2	1.1062	2.50E-03	4.84E-03	-2.8419
ENSG00000106331	PAX4	1.0135	2.50E-03	4.85E-03	-2.8427
ENSG00000165959	CLMN	1.2471	2.52E-03	4.87E-03	-2.8472
ENSG00000114388	NPRL2	1.1067	2.52E-03	4.87E-03	-2.8474
ENSG00000197408	CYP2B6	1.0459	2.52E-03	4.88E-03	-2.8485
ENSG00000124535	WRNIP1	1.1159	2.52E-03	4.89E-03	-2.8503
ENSG00000205777	GAGE1	1.2224	2.53E-03	4.90E-03	-2.8529
ENSG00000171700	RGS19	0.8459	2.53E-03	4.90E-03	-2.8533
ENSG00000163053	SLC16A14	0.7489	2.54E-03	4.92E-03	-2.8567
ENSG00000147010	SH3KBP1	1.1545	2.54E-03	4.92E-03	-2.8570
ENSG00000168329	CX3CR1	1.2637	2.55E-03	4.93E-03	-2.8588
ENSG00000158373	H2BC5	0.7704	2.55E-03	4.93E-03	-2.8593
ENSG00000154134	ROBO3	1.4197	2.55E-03	4.94E-03	-2.8608
ENSG00000115255	REEP6	0.8018	2.56E-03	4.96E-03	-2.8643
ENSG00000074964	ARHGEF10L	1.2134	2.56E-03	4.96E-03	-2.8645
ENSG00000166562	SEC11C	1.1689	2.57E-03	4.97E-03	-2.8671
ENSG00000163617	CCDC191	1.2383	2.57E-03	4.98E-03	-2.8680
ENSG00000242252	BGLAP	1.2488	2.57E-03	4.98E-03	-2.8680
ENSG00000100413	POLR3H	0.8719	2.58E-03	4.98E-03	-2.8690
ENSG00000135960	EDAR	1.0102	2.59E-03	5.00E-03	-2.8730
ENSG00000100228	RAB36	1.3381	2.59E-03	5.00E-03	-2.8736
ENSG00000231924	PSG1	1.0256	2.60E-03	5.01E-03	-2.8756
ENSG00000156515	HK1	1.1796	2.60E-03	5.02E-03	-2.8759
ENSG00000250305	TRMT9B	1.2498	2.60E-03	5.03E-03	-2.8782
ENSG00000196961	AP2A1	1.1168	2.60E-03	5.03E-03	-2.8785
ENSG00000198585	NUDT16	1.1613	2.60E-03	5.03E-03	-2.8788
ENSG00000131171	SH3BGRL	1.1505	2.61E-03	5.05E-03	-2.8816
ENSG00000270011	ZNF559-ZNF177	0.8504	2.61E-03	5.05E-03	-2.8824
ENSG00000145220	LYAR	0.8247	2.62E-03	5.05E-03	-2.8828
ENSG00000050555	LAMC3	1.0543	2.62E-03	5.06E-03	-2.8840
ENSG00000255552	LY6G6E	1.0694	2.62E-03	5.06E-03	-2.8843
ENSG00000185909	KLHDC8B	0.8443	2.62E-03	5.06E-03	-2.8849
ENSG00000168303	MPLKIP	0.9079	2.62E-03	5.06E-03	-2.8856
ENSG00000088543	C3orf18	0.8241	2.64E-03	5.09E-03	-2.8901
ENSG00000099219	ERMP1	0.8548	2.64E-03	5.09E-03	-2.8901
ENSG00000240747	KRBOX1	1.1325	2.65E-03	5.10E-03	-2.8931
ENSG00000141748	ARL5C	1.0473	2.65E-03	5.11E-03	-2.8935
ENSG00000168389	MFSD2A	1.2422	2.65E-03	5.11E-03	-2.8938
ENSG00000216921	FAM240C	1.0516	2.65E-03	5.11E-03	-2.8944
ENSG00000177383	MAGEF1	1.2420	2.66E-03	5.13E-03	-2.8980
ENSG00000149136	SSRP1	1.1108	2.67E-03	5.14E-03	-2.9004
ENSG00000065000	AP3D1	1.1176	2.67E-03	5.16E-03	-2.9028
ENSG00000154277	UCHL1	2.1081	2.68E-03	5.16E-03	-2.9040
ENSG00000060971	ACAA1	1.0939	2.68E-03	5.16E-03	-2.9041
ENSG00000167394	ZNF668	1.1119	2.68E-03	5.16E-03	-2.9044
ENSG00000113966	ARL6	0.8394	2.68E-03	5.17E-03	-2.9052
ENSG00000105664	COMP	1.0783	2.69E-03	5.19E-03	-2.9095
ENSG00000197653	DNAH10	1.1159	2.70E-03	5.20E-03	-2.9113
ENSG00000164296	TIGD6	1.0991	2.70E-03	5.20E-03	-2.9115
ENSG00000153214	TMEM87B	1.1278	2.70E-03	5.21E-03	-2.9125
ENSG00000186818	LILRB4	1.7323	2.70E-03	5.21E-03	-2.9125
ENSG00000078725	BRINP1	0.9609	2.70E-03	5.21E-03	-2.9128
ENSG00000172817	CYP7B1	1.0396	2.70E-03	5.21E-03	-2.9134
ENSG00000136205	TNS3	1.2506	2.71E-03	5.22E-03	-2.9158

Gene ID	Gene Symbol	FC	P.Value	adj.P.Val	B
ENSG00000187997	C17orf99	1.0733	2.71E-03	5.23E-03	-2.9168
ENSG00000134376	CRB1	1.0285	2.72E-03	5.24E-03	-2.9190
ENSG00000163599	CTLA4	1.1336	2.72E-03	5.24E-03	-2.9197
ENSG00000183313	OR52L1	1.0083	2.72E-03	5.24E-03	-2.9200
ENSG00000116406	EDEM3	1.2306	2.73E-03	5.25E-03	-2.9206
ENSG00000173389	IQCF1	1.0388	2.73E-03	5.26E-03	-2.9225
ENSG00000138135	CH25H	1.1085	2.73E-03	5.26E-03	-2.9227
ENSG00000111341	MGP	1.1754	2.73E-03	5.26E-03	-2.9229
ENSG00000151577	DRD3	1.0222	2.74E-03	5.27E-03	-2.9244
ENSG00000110148	CCKBR	1.0168	2.74E-03	5.27E-03	-2.9247
ENSG00000164221	CCDC112	1.1513	2.74E-03	5.28E-03	-2.9259
ENSG00000175206	NPPA	1.0487	2.75E-03	5.28E-03	-2.9274
ENSG00000179242	CDH4	1.0320	2.75E-03	5.29E-03	-2.9289
ENSG00000107960	STN1	1.1156	2.75E-03	5.29E-03	-2.9292
ENSG00000081248	CACNA1S	1.0042	2.76E-03	5.31E-03	-2.9325
ENSG00000180787	ZFP3	0.8119	2.76E-03	5.31E-03	-2.9329
ENSG00000102359	SRPX2	0.9243	2.76E-03	5.31E-03	-2.9334
ENSG00000128917	DLL4	1.1288	2.77E-03	5.32E-03	-2.9348
ENSG00000055211	GINM1	1.1457	2.77E-03	5.32E-03	-2.9349
ENSG00000141506	PIK3R5	1.4061	2.77E-03	5.33E-03	-2.9355
ENSG00000186407	CD300E	1.1725	2.77E-03	5.33E-03	-2.9365
ENSG00000104442	ARMC1	0.8393	2.78E-03	5.33E-03	-2.9371
ENSG00000174276	ZNHIT2	0.8835	2.78E-03	5.34E-03	-2.9374
ENSG00000186501	TMEM222	1.1067	2.78E-03	5.34E-03	-2.9382
ENSG00000005175	RPAP3	1.1250	2.78E-03	5.34E-03	-2.9388
ENSG00000176988	FMR1NB	1.2125	2.78E-03	5.35E-03	-2.9396
ENSG00000196544	BORCS6	0.8592	2.79E-03	5.36E-03	-2.9412
ENSG00000138709	LARP1B	1.1291	2.79E-03	5.36E-03	-2.9415
ENSG00000110435	PDHX	1.1189	2.81E-03	5.39E-03	-2.9467
ENSG00000149809	TM7SF2	0.8646	2.81E-03	5.39E-03	-2.9472
ENSG00000133328	PLAAT2	1.4895	2.82E-03	5.42E-03	-2.9531
ENSG00000154529	CNTNAP3B	1.0847	2.83E-03	5.43E-03	-2.9537
ENSG00000196923	PDLIM7	1.2158	2.83E-03	5.44E-03	-2.9556
ENSG00000125870	SNRPB2	1.1279	2.84E-03	5.45E-03	-2.9580
ENSG00000227234	SPANXB1	1.0174	2.84E-03	5.46E-03	-2.9593
ENSG00000117691	NENF	1.1775	2.85E-03	5.46E-03	-2.9599
ENSG00000171695	LKAAEAR1	1.0735	2.85E-03	5.46E-03	-2.9601
ENSG00000174669	SLC29A2	1.1738	2.85E-03	5.46E-03	-2.9604
ENSG00000151413	NUBPL	1.1234	2.85E-03	5.47E-03	-2.9611
ENSG00000169764	UGP2	0.8756	2.85E-03	5.47E-03	-2.9617
ENSG00000178934	LGALS7B	1.0407	2.85E-03	5.47E-03	-2.9620
ENSG00000117215	PLA2G2D	1.2677	2.85E-03	5.47E-03	-2.9626
ENSG00000043093	DCUN1D1	1.1189	2.86E-03	5.48E-03	-2.9642
ENSG00000027644	INSRR	1.0276	2.86E-03	5.49E-03	-2.9647
ENSG00000161914	ZNF653	0.8786	2.86E-03	5.49E-03	-2.9656
ENSG00000204822	MRPL53	1.0888	2.87E-03	5.49E-03	-2.9664
ENSG00000136271	DDX56	1.1101	2.88E-03	5.52E-03	-2.9710
ENSG00000184270	H2AC21	0.7266	2.88E-03	5.53E-03	-2.9717
ENSG00000078114	NEBL	1.4012	2.88E-03	5.53E-03	-2.9721
ENSG00000124275	MTRR	1.1571	2.89E-03	5.53E-03	-2.9724
ENSG00000187260	WDR86	1.1071	2.89E-03	5.53E-03	-2.9725
ENSG00000103710	RASL12	1.0212	2.89E-03	5.54E-03	-2.9745
ENSG00000102678	FGF9	1.1345	2.89E-03	5.54E-03	-2.9748
ENSG00000061676	NCKAP1	0.7453	2.90E-03	5.55E-03	-2.9762

Gene ID	Gene Symbol	FC	P.Value	adj.P.Val	B
ENSG00000197461	PDGFA	1.2141	2.90E-03	5.55E-03	-2.9764
ENSG00000143340	FAM163A	1.0177	2.90E-03	5.56E-03	-2.9776
ENSG00000215612	HMX1	1.0133	2.91E-03	5.57E-03	-2.9794
ENSG00000107731	UNC5B	1.0808	2.91E-03	5.58E-03	-2.9812
ENSG00000149930	TAOK2	1.1294	2.91E-03	5.58E-03	-2.9815
ENSG00000082482	KCNK2	1.0747	2.93E-03	5.61E-03	-2.9868
ENSG00000139651	ZNF740	1.1502	2.93E-03	5.61E-03	-2.9869
ENSG00000079999	KEAP1	0.8724	2.94E-03	5.62E-03	-2.9881
ENSG00000183479	TREX2	1.1288	2.94E-03	5.62E-03	-2.9892
ENSG00000157423	HYDIN	1.1306	2.95E-03	5.64E-03	-2.9918
ENSG00000169085	VXN	1.2454	2.96E-03	5.66E-03	-2.9948
ENSG00000124126	PREX1	1.3672	2.96E-03	5.66E-03	-2.9955
ENSG00000141956	PRDM15	1.3455	2.96E-03	5.66E-03	-2.9962
ENSG00000146242	TPBG	1.4135	2.96E-03	5.67E-03	-2.9964
ENSG00000075399	VPS9D1	1.1686	2.96E-03	5.67E-03	-2.9969
ENSG00000112715	VEGFA	1.2090	2.97E-03	5.68E-03	-2.9996
ENSG00000213463	SYNJ2BP	0.8595	2.98E-03	5.69E-03	-3.0011
ENSG00000137478	FCHSD2	0.7858	2.98E-03	5.70E-03	-3.0019
ENSG00000099330	OCEL1	0.8592	2.99E-03	5.71E-03	-3.0035
ENSG00000167615	LENG8	1.1828	2.99E-03	5.72E-03	-3.0056
ENSG00000239306	RBM14	1.1537	3.00E-03	5.72E-03	-3.0066
ENSG00000168496	FEN1	1.2068	3.00E-03	5.73E-03	-3.0069
ENSG00000128039	SRD5A3	0.8357	3.00E-03	5.73E-03	-3.0076
ENSG00000023516	AKAP11	0.8584	3.00E-03	5.73E-03	-3.0082
ENSG00000170442	KRT86	1.0541	3.00E-03	5.73E-03	-3.0083
ENSG00000163083	INHBB	1.0329	3.00E-03	5.74E-03	-3.0088
ENSG00000106829	TLE4	1.1927	3.00E-03	5.74E-03	-3.0090
ENSG00000173531	MST1	1.1963	3.01E-03	5.75E-03	-3.0109
ENSG00000188033	ZNF490	1.1072	3.02E-03	5.76E-03	-3.0137
ENSG00000137507	LRRC32	1.1156	3.03E-03	5.78E-03	-3.0163
ENSG00000145423	SFRP2	1.1941	3.03E-03	5.79E-03	-3.0183
ENSG00000155714	PDZD9	1.0345	3.04E-03	5.80E-03	-3.0189
ENSG00000197632	SERPINB2	1.1915	3.04E-03	5.80E-03	-3.0190
ENSG00000204481	PRAMEF14	1.0077	3.04E-03	5.81E-03	-3.0213
ENSG00000162511	LAPTM5	1.7649	3.04E-03	5.81E-03	-3.0214
ENSG00000167523	SPATA33	1.1257	3.05E-03	5.82E-03	-3.0224
ENSG00000249992	TMEM158	1.0582	3.05E-03	5.82E-03	-3.0229
ENSG00000132872	SYT4	1.0649	3.05E-03	5.82E-03	-3.0239
ENSG00000213185	FAM24B	1.2439	3.06E-03	5.83E-03	-3.0245
ENSG00000178343	SHISA3	1.1467	3.06E-03	5.84E-03	-3.0258
ENSG00000167272	POP5	0.8613	3.06E-03	5.84E-03	-3.0259
ENSG00000244509	APOBEC3C	1.1637	3.07E-03	5.85E-03	-3.0285
ENSG00000165389	SPTSSA	1.1220	3.07E-03	5.85E-03	-3.0287
ENSG00000179091	CYC1	1.1084	3.08E-03	5.86E-03	-3.0307
ENSG00000044524	EPHA3	1.1189	3.08E-03	5.86E-03	-3.0309
ENSG00000198105	ZNF248	0.8589	3.10E-03	5.91E-03	-3.0383
ENSG00000177156	TALDO1	0.8798	3.10E-03	5.91E-03	-3.0388
ENSG00000171219	CDC42BPG	1.2508	3.11E-03	5.93E-03	-3.0412
ENSG00000135423	GLS2	1.1699	3.11E-03	5.93E-03	-3.0416
ENSG00000115616	SLC9A2	1.1486	3.11E-03	5.93E-03	-3.0420
ENSG00000171448	ZBTB26	0.8679	3.12E-03	5.94E-03	-3.0432
ENSG00000082212	ME2	0.8374	3.12E-03	5.95E-03	-3.0441
ENSG00000123901	GPR83	1.0585	3.13E-03	5.96E-03	-3.0463
ENSG00000130656	HBZ	1.0574	3.13E-03	5.96E-03	-3.0467

Gene ID	Gene Symbol	FC	P.Value	adj.P.Val	B
ENSG00000183978	COA3	0.8375	3.13E-03	5.97E-03	-3.0473
ENSG00000148362	PAXX	0.7957	3.14E-03	5.98E-03	-3.0494
ENSG00000243449	C4orf48	0.8016	3.14E-03	5.98E-03	-3.0501
ENSG00000162437	RAVER2	1.1517	3.14E-03	5.98E-03	-3.0504
ENSG00000213139	CRYGS	0.8531	3.14E-03	5.99E-03	-3.0507
ENSG00000163002	NUP35	1.1223	3.15E-03	5.99E-03	-3.0516
ENSG00000204231	RXRB	1.1155	3.15E-03	5.99E-03	-3.0516
ENSG00000171115	GIMAP8	0.8511	3.15E-03	6.00E-03	-3.0526
ENSG00000167554	ZNF610	1.1137	3.16E-03	6.01E-03	-3.0542
ENSG00000078900	TP73	1.1803	3.17E-03	6.02E-03	-3.0571
ENSG00000123570	RAB9B	1.0912	3.18E-03	6.06E-03	-3.0619
ENSG00000135747	ZNF670-ZNF695	1.1082	3.18E-03	6.06E-03	-3.0622
ENSG00000164105	SAP30	1.1539	3.18E-03	6.06E-03	-3.0622
ENSG00000151892	GFRA1	1.1012	3.19E-03	6.06E-03	-3.0634
ENSG00000228696	ARL17B	1.1333	3.20E-03	6.09E-03	-3.0674
ENSG00000109917	ZPR1	1.1107	3.21E-03	6.10E-03	-3.0683
ENSG00000168995	SIGLEC7	0.8239	3.21E-03	6.10E-03	-3.0695
ENSG00000169314	C22orf15	1.0644	3.21E-03	6.10E-03	-3.0694
ENSG00000174370	C11orf45	1.0846	3.21E-03	6.10E-03	-3.0694
ENSG00000044574	HSPA5	1.1814	3.21E-03	6.11E-03	-3.0704
ENSG00000105675	ATP4A	1.0141	3.21E-03	6.11E-03	-3.0710
ENSG00000183527	PSMG1	1.1318	3.22E-03	6.11E-03	-3.0712
ENSG00000186577	SMIM29	1.1285	3.22E-03	6.11E-03	-3.0714
ENSG00000117394	SLC2A1	1.1776	3.22E-03	6.11E-03	-3.0715
ENSG00000188848	BEND4	1.0590	3.23E-03	6.13E-03	-3.0743
ENSG00000010379	SLC6A13	0.8791	3.23E-03	6.14E-03	-3.0761
ENSG00000172244	C5orf34	1.1452	3.23E-03	6.14E-03	-3.0763
ENSG00000128283	CDC42EP1	1.2392	3.24E-03	6.15E-03	-3.0772
ENSG00000259288	BUB1B-PAK6	1.0362	3.26E-03	6.20E-03	-3.0846
ENSG00000038274	MAT2B	0.8783	3.26E-03	6.20E-03	-3.0849
ENSG00000166598	HSP90B1	1.1782	3.27E-03	6.21E-03	-3.0860
ENSG00000101871	MID1	1.1418	3.29E-03	6.24E-03	-3.0912
ENSG00000183844	FAM3B	1.2831	3.29E-03	6.24E-03	-3.0912
ENSG00000111291	GPRC5D	0.6999	3.29E-03	6.25E-03	-3.0921
ENSG00000143353	LYPLAL1	0.8448	3.29E-03	6.25E-03	-3.0924
ENSG00000071203	MS4A12	1.0156	3.30E-03	6.25E-03	-3.0936
ENSG00000132718	SYT11	1.0782	3.30E-03	6.27E-03	-3.0962
ENSG00000148484	RSU1	1.1485	3.31E-03	6.29E-03	-3.0986
ENSG00000135773	CAPN9	1.0173	3.32E-03	6.30E-03	-3.1012
ENSG00000172139	SLC9C1	0.8305	3.32E-03	6.30E-03	-3.1011
ENSG00000070371	CLTCL1	1.1031	3.32E-03	6.31E-03	-3.1015
ENSG00000100104	SRRD	1.1168	3.33E-03	6.31E-03	-3.1020
ENSG00000137841	PLCB2	1.3832	3.33E-03	6.32E-03	-3.1034
ENSG00000180185	FAHD1	0.8571	3.33E-03	6.32E-03	-3.1041
ENSG00000168273	SMIM4	0.8800	3.34E-03	6.32E-03	-3.1045
ENSG00000128973	CLN6	0.8287	3.34E-03	6.32E-03	-3.1046
ENSG00000172061	LRRC15	1.0072	3.34E-03	6.34E-03	-3.1066
ENSG00000179111	HES7	1.1302	3.35E-03	6.34E-03	-3.1073
ENSG00000101276	SLC52A3	1.0680	3.35E-03	6.34E-03	-3.1078
ENSG00000164251	F2RL1	0.9429	3.35E-03	6.35E-03	-3.1084
ENSG00000188234	AGAP4	1.2008	3.35E-03	6.35E-03	-3.1094
ENSG00000039123	MTREX	0.8726	3.36E-03	6.37E-03	-3.1112
ENSG00000138660	AP1AR	1.1411	3.36E-03	6.37E-03	-3.1119
ENSG00000103343	ZNF174	0.8775	3.37E-03	6.38E-03	-3.1133

Gene ID	Gene Symbol	FC	P.Value	adj.P.Val	B
ENSG00000076554	TPD52	1.1373	3.38E-03	6.40E-03	-3.1164
ENSG00000206177	HBM	0.7024	3.38E-03	6.40E-03	-3.1166
ENSG00000007933	FMO3	1.1870	3.38E-03	6.40E-03	-3.1170
ENSG00000110104	CCDC86	1.1793	3.38E-03	6.40E-03	-3.1171
ENSG00000163344	PMVK	1.1714	3.39E-03	6.41E-03	-3.1184
ENSG00000168517	HEXIM2	0.8869	3.39E-03	6.41E-03	-3.1186
ENSG00000016391	CHDH	1.2155	3.39E-03	6.41E-03	-3.1189
ENSG00000004766	VPS50	1.1255	3.39E-03	6.42E-03	-3.1198
ENSG00000074181	NOTCH3	1.1064	3.39E-03	6.42E-03	-3.1200
ENSG00000205869	KRTAP5-1	1.1042	3.41E-03	6.45E-03	-3.1238
ENSG00000121933	TMIGD3	0.8045	3.41E-03	6.46E-03	-3.1254
ENSG00000170214	ADRA1B	1.0179	3.41E-03	6.46E-03	-3.1256
ENSG00000122756	CNTFR	1.0388	3.42E-03	6.46E-03	-3.1267
ENSG00000135205	CCDC146	0.8632	3.42E-03	6.47E-03	-3.1274
ENSG00000004776	HSPB6	1.0515	3.42E-03	6.47E-03	-3.1280
ENSG00000198001	IRAK4	1.1354	3.43E-03	6.48E-03	-3.1287
ENSG00000157766	ACAN	1.0826	3.43E-03	6.48E-03	-3.1290
ENSG00000188389	PDCD1	1.2008	3.43E-03	6.48E-03	-3.1290
ENSG00000124786	SLC35B3	0.8664	3.43E-03	6.48E-03	-3.1300
ENSG00000116044	NFE2L2	1.1144	3.43E-03	6.49E-03	-3.1302
ENSG00000172116	CD8B	1.2824	3.43E-03	6.49E-03	-3.1308
ENSG00000139874	SSTR1	1.1020	3.44E-03	6.50E-03	-3.1327
ENSG00000139914	FITM1	1.0697	3.44E-03	6.50E-03	-3.1332
ENSG00000144120	TMEM177	0.8469	3.45E-03	6.52E-03	-3.1360
ENSG00000184347	SLIT3	1.0953	3.45E-03	6.53E-03	-3.1366
ENSG00000134248	LAMTOR5	1.0990	3.46E-03	6.54E-03	-3.1383
ENSG00000172262	ZNF131	1.0961	3.46E-03	6.55E-03	-3.1393
ENSG00000038210	PI4K2B	1.1115	3.47E-03	6.56E-03	-3.1412
ENSG00000231852	CYP21A2	1.2719	3.47E-03	6.56E-03	-3.1417
ENSG00000117877	POLR1G	0.8443	3.48E-03	6.56E-03	-3.1421
ENSG00000083544	TDRD3	0.8866	3.48E-03	6.57E-03	-3.1426
ENSG00000074660	SCARF1	0.8444	3.48E-03	6.57E-03	-3.1430
ENSG00000075407	ZNF37A	1.1433	3.49E-03	6.59E-03	-3.1454
ENSG00000139726	DENR	1.1242	3.50E-03	6.60E-03	-3.1477
ENSG00000175538	KCNE3	0.8948	3.50E-03	6.60E-03	-3.1481
ENSG00000160883	HK3	0.7604	3.50E-03	6.61E-03	-3.1486
ENSG00000174950	CD164L2	1.0632	3.51E-03	6.62E-03	-3.1500
ENSG00000163902	RPN1	0.8855	3.51E-03	6.63E-03	-3.1512
ENSG00000189306	RRP7A	1.1348	3.52E-03	6.64E-03	-3.1533
ENSG00000186487	MYT1L	1.1530	3.52E-03	6.64E-03	-3.1539
ENSG00000164306	PRIMPOL	1.1390	3.52E-03	6.65E-03	-3.1542
ENSG00000143977	SNRPG	0.8746	3.53E-03	6.66E-03	-3.1561
ENSG00000072736	NFATC3	1.1460	3.53E-03	6.67E-03	-3.1574
ENSG00000178732	GP5	1.0366	3.54E-03	6.67E-03	-3.1578
ENSG00000110079	MS4A4A	0.7633	3.55E-03	6.69E-03	-3.1602
ENSG00000159184	HOXB13	1.0191	3.55E-03	6.69E-03	-3.1608
ENSG00000135299	ANKRD6	1.1522	3.55E-03	6.70E-03	-3.1620
ENSG00000213401	MAGEA12	1.2671	3.55E-03	6.70E-03	-3.1619
ENSG00000157823	AP3S2	0.8659	3.55E-03	6.70E-03	-3.1623
ENSG00000100412	ACO2	1.1196	3.57E-03	6.72E-03	-3.1655
ENSG00000165526	RPUSD4	0.8853	3.57E-03	6.72E-03	-3.1656
ENSG00000144724	PTPRG	1.3067	3.57E-03	6.72E-03	-3.1659
ENSG00000117748	RPA2	0.8769	3.57E-03	6.74E-03	-3.1676
ENSG00000198131	ZNF544	0.8195	3.58E-03	6.75E-03	-3.1696

Gene ID	Gene Symbol	FC	P.Value	adj.P.Val	B
ENSG00000198914	POU3F3	1.0080	3.59E-03	6.76E-03	-3.1718
ENSG00000178397	FAM220A	0.8678	3.59E-03	6.77E-03	-3.1726
ENSG00000186868	MAPT	1.0735	3.60E-03	6.77E-03	-3.1729
ENSG00000037042	TUBG2	1.1764	3.60E-03	6.77E-03	-3.1730
ENSG00000116096	SPR	0.8408	3.61E-03	6.79E-03	-3.1760
ENSG00000189042	ZNF567	1.1342	3.61E-03	6.79E-03	-3.1761
ENSG00000156475	PPP2R2B	1.1794	3.62E-03	6.81E-03	-3.1782
ENSG00000145569	OTULINL	1.3249	3.62E-03	6.81E-03	-3.1787
ENSG00000168702	LRP1B	1.0383	3.63E-03	6.83E-03	-3.1819
ENSG00000124257	NEURL2	1.1336	3.64E-03	6.84E-03	-3.1829
ENSG00000114491	UMPS	0.8384	3.64E-03	6.84E-03	-3.1832
ENSG00000087095	NLK	0.8494	3.64E-03	6.86E-03	-3.1851
ENSG00000055118	KCNH2	1.3798	3.65E-03	6.88E-03	-3.1879
ENSG00000164309	CMYA5	1.0503	3.66E-03	6.88E-03	-3.1885
ENSG00000170962	PDGFD	1.2668	3.66E-03	6.89E-03	-3.1892
ENSG00000151617	EDNRA	1.0355	3.66E-03	6.89E-03	-3.1894
ENSG00000163626	COX18	0.8811	3.66E-03	6.89E-03	-3.1899
ENSG00000168734	PKIG	1.2344	3.68E-03	6.92E-03	-3.1942
ENSG00000006128	TAC1	1.1817	3.68E-03	6.93E-03	-3.1953
ENSG00000137727	ARHGAP20	1.0274	3.69E-03	6.94E-03	-3.1971
ENSG00000107798	LIPA	0.8221	3.70E-03	6.96E-03	-3.1991
ENSG00000162654	GBP4	1.2259	3.70E-03	6.96E-03	-3.1998
ENSG00000134996	OSTF1	0.8789	3.71E-03	6.97E-03	-3.2008
ENSG00000121900	TMEM54	1.3018	3.71E-03	6.97E-03	-3.2010
ENSG00000198225	FKBP1C	1.0486	3.71E-03	6.98E-03	-3.2018
ENSG00000106305	AIMP2	0.8547	3.72E-03	6.99E-03	-3.2037
ENSG00000161671	EMC10	1.1324	3.73E-03	7.01E-03	-3.2064
ENSG00000142444	TIMM29	1.1091	3.73E-03	7.01E-03	-3.2071
ENSG00000157557	ETS2	1.2661	3.73E-03	7.02E-03	-3.2074
ENSG00000213316	LTC4S	0.7943	3.75E-03	7.04E-03	-3.2109
ENSG00000259431	THTPA	0.8584	3.76E-03	7.06E-03	-3.2131
ENSG00000134987	WDR36	1.1205	3.77E-03	7.08E-03	-3.2160
ENSG00000114739	ACVR2B	1.1162	3.78E-03	7.10E-03	-3.2182
ENSG00000135045	C9orf40	0.8489	3.78E-03	7.10E-03	-3.2194
ENSG00000187537	POTEG	1.1065	3.78E-03	7.10E-03	-3.2194
ENSG00000186583	SPATC1	0.8626	3.80E-03	7.13E-03	-3.2228
ENSG00000166123	GPT2	1.2372	3.80E-03	7.13E-03	-3.2230
ENSG00000125686	MED1	1.1544	3.80E-03	7.14E-03	-3.2238
ENSG00000144792	ZNF660	1.1300	3.80E-03	7.14E-03	-3.2242
ENSG00000138411	HECW2	1.0706	3.81E-03	7.15E-03	-3.2257
ENSG00000170631	ZNF16	1.1018	3.81E-03	7.16E-03	-3.2268
ENSG00000133104	SPART	0.7739	3.82E-03	7.17E-03	-3.2278
ENSG00000204611	ZNF616	1.1530	3.82E-03	7.17E-03	-3.2282
ENSG00000105784	RUNDC3B	1.0815	3.82E-03	7.17E-03	-3.2287
ENSG00000123095	BHLHE41	1.2471	3.83E-03	7.19E-03	-3.2307
ENSG00000066248	NGEF	1.0728	3.84E-03	7.20E-03	-3.2328
ENSG00000180917	CMTR2	0.8642	3.84E-03	7.20E-03	-3.2327
ENSG00000106128	GHRHR	1.0845	3.85E-03	7.21E-03	-3.2341
ENSG00000153093	ACOXL	0.8048	3.86E-03	7.24E-03	-3.2383
ENSG00000196787	H2AC11	0.8198	3.87E-03	7.25E-03	-3.2387
ENSG00000168993	CPLX1	1.1223	3.87E-03	7.25E-03	-3.2396
ENSG00000126785	RHOJ	1.0252	3.87E-03	7.25E-03	-3.2399
ENSG00000151689	INPP1	1.1900	3.87E-03	7.25E-03	-3.2400
ENSG00000159961	OR3A3	0.9951	3.88E-03	7.26E-03	-3.2411

Gene ID	Gene Symbol	FC	P.Value	adj.P.Val	B
ENSG00000109851	DBX1	0.9037	3.89E-03	7.29E-03	-3.2445
ENSG00000155097	ATP6V1C1	1.1540	3.89E-03	7.29E-03	-3.2451
ENSG00000239642	MEIKIN	1.0833	3.91E-03	7.33E-03	-3.2499
ENSG00000100138	SNU13	1.0957	3.93E-03	7.36E-03	-3.2536
ENSG00000145850	TIMD4	0.6940	3.98E-03	7.45E-03	-3.2643
ENSG00000015171	ZMYND11	1.1551	3.98E-03	7.45E-03	-3.2647
ENSG00000154645	CHODL	0.8516	3.98E-03	7.46E-03	-3.2659
ENSG00000175093	SPSB4	0.9390	3.99E-03	7.48E-03	-3.2683
ENSG00000150457	LATS2	0.8631	4.01E-03	7.50E-03	-3.2717
ENSG00000126458	RRAS	1.2196	4.01E-03	7.51E-03	-3.2721
ENSG00000181817	LSM10	0.8964	4.02E-03	7.52E-03	-3.2734
ENSG00000140259	MFAP1	0.8522	4.03E-03	7.54E-03	-3.2766
ENSG00000157734	SNX22	1.1527	4.03E-03	7.54E-03	-3.2765
ENSG00000172031	EPHX4	1.0511	4.03E-03	7.54E-03	-3.2767
ENSG00000159199	ATP5MC1	0.8658	4.05E-03	7.58E-03	-3.2809
ENSG00000081870	HSPB11	0.8766	4.05E-03	7.58E-03	-3.2815
ENSG00000116183	PAPPA2	1.0594	4.06E-03	7.59E-03	-3.2824
ENSG00000144554	FANCD2	1.1794	4.07E-03	7.61E-03	-3.2848
ENSG00000130363	RSPH3	1.1325	4.07E-03	7.61E-03	-3.2852
ENSG00000111716	LDHB	0.8712	4.07E-03	7.61E-03	-3.2855
ENSG00000178021	TSPYL6	1.0293	4.08E-03	7.62E-03	-3.2870
ENSG00000135931	ARMC9	1.1643	4.08E-03	7.63E-03	-3.2880
ENSG00000112992	NNT	0.8871	4.09E-03	7.64E-03	-3.2892
ENSG00000183323	CCDC125	0.8789	4.10E-03	7.66E-03	-3.2913
ENSG00000197442	MAP3K5	1.1852	4.10E-03	7.66E-03	-3.2918
ENSG00000143801	PSEN2	0.8541	4.11E-03	7.68E-03	-3.2943
ENSG00000150510	FAM124A	1.0535	4.11E-03	7.68E-03	-3.2943
ENSG00000165832	TRUB1	1.1282	4.11E-03	7.68E-03	-3.2949
ENSG00000115507	OTX1	1.1596	4.12E-03	7.70E-03	-3.2968
ENSG00000196260	SFTA2	1.0544	4.12E-03	7.70E-03	-3.2970
ENSG00000125746	EML2	1.1404	4.12E-03	7.70E-03	-3.2972
ENSG00000128510	CPA4	1.0353	4.12E-03	7.71E-03	-3.2978
ENSG00000258588	TRIM6-TRIM34	0.9498	4.13E-03	7.71E-03	-3.2984
ENSG00000156299	TIAM1	1.3070	4.13E-03	7.72E-03	-3.3000
ENSG00000213471	TTLL13P	0.9603	4.14E-03	7.74E-03	-3.3017
ENSG00000197471	SPN	1.5074	4.15E-03	7.75E-03	-3.3033
ENSG00000172828	CES3	1.2258	4.15E-03	7.75E-03	-3.3035
ENSG00000177830	CHID1	0.8807	4.15E-03	7.75E-03	-3.3040
ENSG00000103479	RBL2	1.1729	4.16E-03	7.77E-03	-3.3055
ENSG00000254413	CHKB-CPT1B	1.1971	4.17E-03	7.79E-03	-3.3081
ENSG00000127249	ATP13A4	1.1480	4.17E-03	7.79E-03	-3.3083
ENSG00000100014	SPECC1L	1.1683	4.17E-03	7.79E-03	-3.3085
ENSG00000188263	IL17REL	1.0756	4.17E-03	7.79E-03	-3.3085
ENSG00000118855	MFSD1	0.8703	4.18E-03	7.80E-03	-3.3103
ENSG00000109472	CPE	1.0665	4.19E-03	7.81E-03	-3.3115
ENSG00000165066	NKX6-3	1.2211	4.19E-03	7.81E-03	-3.3115
ENSG00000044090	CUL7	1.1525	4.19E-03	7.82E-03	-3.3122
ENSG00000115504	EHBP1	1.1305	4.21E-03	7.85E-03	-3.3163
ENSG00000105642	KCNN1	1.0303	4.21E-03	7.85E-03	-3.3165
ENSG00000185127	C6orf120	0.8836	4.21E-03	7.86E-03	-3.3172
ENSG00000125170	DOK4	1.2356	4.22E-03	7.86E-03	-3.3179
ENSG00000132821	VSTM2L	1.1107	4.22E-03	7.87E-03	-3.3186
ENSG00000149506	ZP1	1.2290	4.22E-03	7.87E-03	-3.3189
ENSG00000168263	KCNV2	0.9788	4.23E-03	7.88E-03	-3.3202

Gene ID	Gene Symbol	FC	P.Value	adj.P.Val	B
ENSG00000157429	ZNF19	1.1155	4.23E-03	7.88E-03	-3.3205
ENSG00000132356	PRKAA1	1.1222	4.23E-03	7.89E-03	-3.3214
ENSG00000124216	SNAI1	1.2005	4.24E-03	7.89E-03	-3.3217
ENSG00000168887	C2orf68	1.1310	4.23E-03	7.89E-03	-3.3217
ENSG00000122971	ACADS	0.8395	4.24E-03	7.91E-03	-3.3235
ENSG00000188643	S100A16	1.1450	4.25E-03	7.92E-03	-3.3247
ENSG00000166169	POLL	0.8756	4.26E-03	7.93E-03	-3.3261
ENSG00000156471	PTDSS1	1.1052	4.26E-03	7.94E-03	-3.3271
ENSG00000166866	MYO1A	1.0731	4.26E-03	7.94E-03	-3.3280
ENSG00000156886	ITGAD	0.8291	4.27E-03	7.96E-03	-3.3297
ENSG00000182013	PNMA8A	1.0870	4.29E-03	7.99E-03	-3.3337
ENSG00000113269	RNF130	0.6930	4.29E-03	7.99E-03	-3.3342
ENSG00000008277	ADAM22	1.2160	4.30E-03	8.01E-03	-3.3358
ENSG00000124496	TRERF1	1.2309	4.30E-03	8.01E-03	-3.3359
ENSG00000226887	ERVMER34-1	0.7974	4.30E-03	8.01E-03	-3.3359
ENSG00000178035	IMPDH2	1.1140	4.31E-03	8.02E-03	-3.3372
ENSG00000131873	CHSY1	1.1941	4.31E-03	8.02E-03	-3.3375
ENSG00000085662	AKR1B1	1.2619	4.31E-03	8.02E-03	-3.3377
ENSG00000168661	ZNF30	0.8609	4.31E-03	8.02E-03	-3.3378
ENSG00000112981	NME5	1.1320	4.31E-03	8.02E-03	-3.3384
ENSG00000169877	AHSP	0.7025	4.31E-03	8.02E-03	-3.3384
ENSG00000206053	JPT2	0.8280	4.32E-03	8.04E-03	-3.3401
ENSG00000111275	ALDH2	0.7661	4.33E-03	8.05E-03	-3.3414
ENSG00000132321	IQCA1	1.1205	4.33E-03	8.05E-03	-3.3414
ENSG00000104812	GYS1	1.1280	4.33E-03	8.05E-03	-3.3422
ENSG00000168724	DNAJC21	1.0937	4.34E-03	8.07E-03	-3.3443
ENSG00000158516	CPA2	1.0209	4.35E-03	8.08E-03	-3.3451
ENSG00000175809	CBLL2	1.0089	4.36E-03	8.10E-03	-3.3472
ENSG00000164142	FHIP1A	0.8585	4.37E-03	8.12E-03	-3.3499
ENSG00000175193	PARL	1.0869	4.39E-03	8.15E-03	-3.3533
ENSG00000259803	SLC22A31	1.6141	4.39E-03	8.16E-03	-3.3544
ENSG00000140506	LMAN1L	1.0687	4.39E-03	8.16E-03	-3.3546
ENSG00000182578	CSF1R	0.8147	4.39E-03	8.17E-03	-3.3553
ENSG00000129993	CBFA2T3	0.8185	4.40E-03	8.18E-03	-3.3567
ENSG00000213588	ZBTB9	0.8572	4.40E-03	8.18E-03	-3.3568
ENSG00000125831	CST11	1.0411	4.40E-03	8.18E-03	-3.3571
ENSG00000011465	DCN	1.1660	4.41E-03	8.19E-03	-3.3582
ENSG00000248919	ATP5MF-PTCD1	0.8789	4.41E-03	8.19E-03	-3.3583
ENSG00000118242	MREG	1.2091	4.41E-03	8.19E-03	-3.3586
ENSG00000132153	DHX30	1.1054	4.41E-03	8.19E-03	-3.3586
ENSG00000204856	FAM216A	0.8625	4.41E-03	8.19E-03	-3.3589
ENSG00000113328	CCNG1	0.8888	4.41E-03	8.19E-03	-3.3591
ENSG00000173465	ZNRD2	0.9087	4.42E-03	8.21E-03	-3.3606
ENSG00000158874	APOA2	1.0707	4.42E-03	8.21E-03	-3.3614
ENSG00000100376	FAM118A	1.2660	4.43E-03	8.23E-03	-3.3632
ENSG00000150594	ADRA2A	1.0538	4.43E-03	8.23E-03	-3.3634
ENSG00000170160	CCDC144A	1.3545	4.43E-03	8.23E-03	-3.3633
ENSG00000196262	PPIA	0.8843	4.44E-03	8.23E-03	-3.3639
ENSG00000089505	CMTM1	1.1430	4.44E-03	8.23E-03	-3.3642
ENSG00000163517	HDAC11	1.1475	4.45E-03	8.25E-03	-3.3667
ENSG00000063587	ZNF275	1.1930	4.45E-03	8.26E-03	-3.3668
ENSG00000128045	RASL11B	1.0893	4.46E-03	8.27E-03	-3.3683
ENSG00000070367	EXOC5	1.1570	4.46E-03	8.28E-03	-3.3693
ENSG00000081800	SLC13A1	1.0127	4.47E-03	8.28E-03	-3.3701

Gene ID	Gene Symbol	FC	P.Value	adj.P.Val	B
ENSG00000166734	GOLM2	1.1059	4.47E-03	8.29E-03	-3.3706
ENSG00000140873	ADAMTS18	1.0154	4.49E-03	8.33E-03	-3.3754
ENSG00000170222	ADPRM	0.8997	4.51E-03	8.36E-03	-3.3783
ENSG00000181195	PENK	1.0497	4.51E-03	8.36E-03	-3.3793
ENSG00000131100	ATP6V1E1	1.1216	4.53E-03	8.40E-03	-3.3835
ENSG00000066933	MYO9A	1.1299	4.54E-03	8.41E-03	-3.3847
ENSG00000197702	PARVA	1.0524	4.54E-03	8.42E-03	-3.3854
ENSG00000273331	TM4SF19-DYNLT2B	1.0319	4.56E-03	8.45E-03	-3.3888
ENSG00000109670	FBXW7	1.1737	4.56E-03	8.45E-03	-3.3889
ENSG00000173482	PTPRM	1.2411	4.57E-03	8.46E-03	-3.3901
ENSG00000185670	ZBTB3	1.1119	4.57E-03	8.46E-03	-3.3901
ENSG00000250254	PTTG2	1.0937	4.57E-03	8.46E-03	-3.3902
ENSG00000109158	GABRA4	1.2661	4.57E-03	8.46E-03	-3.3908
ENSG00000134812	CBLIF	1.0195	4.57E-03	8.47E-03	-3.3912
ENSG00000001461	NIPAL3	0.8661	4.58E-03	8.48E-03	-3.3930
ENSG00000166411	IDH3A	0.8731	4.58E-03	8.48E-03	-3.3932
ENSG00000160256	SLX9	0.8743	4.59E-03	8.50E-03	-3.3953
ENSG00000173068	BNC2	1.1372	4.59E-03	8.50E-03	-3.3955
ENSG00000154027	AK5	1.1272	4.60E-03	8.50E-03	-3.3957
ENSG00000164729	SLC35G3	1.0146	4.62E-03	8.55E-03	-3.4006
ENSG00000028277	POU2F2	0.8357	4.62E-03	8.55E-03	-3.4008
ENSG00000126773	PCNX4	1.1456	4.63E-03	8.57E-03	-3.4028
ENSG00000177202	SPACA4	1.0278	4.63E-03	8.57E-03	-3.4034
ENSG00000177192	PUS1	1.1126	4.64E-03	8.59E-03	-3.4047
ENSG00000163273	NPPC	1.1138	4.64E-03	8.59E-03	-3.4049
ENSG00000152082	MZT2B	1.1024	4.65E-03	8.59E-03	-3.4058
ENSG00000126266	FFAR1	1.0457	4.67E-03	8.63E-03	-3.4102
ENSG00000214413	BBIP1	1.0968	4.67E-03	8.64E-03	-3.4106
ENSG00000169692	AGPAT2	1.3269	4.69E-03	8.68E-03	-3.4147
ENSG00000135519	KCNH3	1.1150	4.70E-03	8.68E-03	-3.4151
ENSG00000175189	INHBC	1.0881	4.72E-03	8.73E-03	-3.4202
ENSG00000115361	ACADL	1.0364	4.72E-03	8.73E-03	-3.4205
ENSG00000163923	RPL39L	1.1608	4.73E-03	8.74E-03	-3.4221
ENSG00000213171	LINGO4	1.0193	4.74E-03	8.75E-03	-3.4228
ENSG00000125826	RBCK1	1.1166	4.74E-03	8.75E-03	-3.4232
ENSG00000101951	PAGE4	1.2174	4.74E-03	8.75E-03	-3.4235
ENSG00000183207	RUVBL2	0.8950	4.74E-03	8.76E-03	-3.4242
ENSG00000172037	LAMB2	0.7765	4.75E-03	8.77E-03	-3.4254
ENSG00000083444	PLOD1	1.1656	4.77E-03	8.82E-03	-3.4301
ENSG00000128708	HAT1	1.1298	4.78E-03	8.82E-03	-3.4308
ENSG00000164197	RNF180	1.2225	4.78E-03	8.83E-03	-3.4318
ENSG00000226742	HSBP1L1	1.3293	4.78E-03	8.83E-03	-3.4323
ENSG00000085871	MGST2	1.3391	4.79E-03	8.85E-03	-3.4339
ENSG00000154642	C21orf91	1.1744	4.80E-03	8.86E-03	-3.4350
ENSG00000170234	PWWP2A	0.8569	4.82E-03	8.89E-03	-3.4385
ENSG00000116288	PARK7	0.9190	4.82E-03	8.90E-03	-3.4390
ENSG00000144057	ST6GAL2	1.0654	4.83E-03	8.92E-03	-3.4412
ENSG00000101986	ABCD1	1.1507	4.83E-03	8.92E-03	-3.4414
ENSG00000101542	CDH20	1.0651	4.84E-03	8.93E-03	-3.4422
ENSG00000158717	RNF166	1.1582	4.84E-03	8.93E-03	-3.4425
ENSG00000213246	SUPT4H1	1.0903	4.85E-03	8.95E-03	-3.4450
ENSG00000243789	JMJD7	0.8674	4.86E-03	8.96E-03	-3.4456
ENSG00000099194	SCD	0.7718	4.87E-03	8.98E-03	-3.4478
ENSG00000212128	TAS2R13	1.0157	4.87E-03	8.98E-03	-3.4479

Gene ID	Gene Symbol	FC	P.Value	adj.P.Val	B
ENSG00000171291	ZNF439	0.8558	4.87E-03	8.98E-03	-3.4482
ENSG00000151491	EPS8	1.1951	4.87E-03	8.98E-03	-3.4485
ENSG00000203782	LORICRIN	1.0915	4.88E-03	8.99E-03	-3.4499
ENSG00000114770	ABCC5	0.8484	4.88E-03	9.00E-03	-3.4502
ENSG00000157613	CREB3L1	1.0943	4.88E-03	9.00E-03	-3.4506
ENSG00000168748	CA7	0.9775	4.90E-03	9.02E-03	-3.4531
ENSG00000105401	CDC37	1.0824	4.90E-03	9.03E-03	-3.4536
ENSG00000181552	EDDM3B	1.0058	4.91E-03	9.05E-03	-3.4557
ENSG00000039650	PNKP	1.1144	4.92E-03	9.07E-03	-3.4584
ENSG00000115107	STEAP3	1.2527	4.93E-03	9.08E-03	-3.4595
ENSG00000118369	USP35	1.1214	4.93E-03	9.08E-03	-3.4596
ENSG00000140067	FAM181A	1.0119	4.95E-03	9.12E-03	-3.4630
ENSG00000175040	CHST2	0.7024	4.95E-03	9.12E-03	-3.4632
ENSG00000112699	GMDS	0.8844	4.95E-03	9.12E-03	-3.4636
ENSG00000167987	VPS37C	0.8803	4.97E-03	9.15E-03	-3.4665
ENSG00000188026	RILPL1	1.1058	4.98E-03	9.18E-03	-3.4690
ENSG00000042813	ZPBP	1.0744	4.99E-03	9.18E-03	-3.4696
ENSG00000134955	SLC37A2	1.1936	4.99E-03	9.19E-03	-3.4706
ENSG00000151320	AKAP6	1.1483	5.02E-03	9.24E-03	-3.4759
ENSG00000103423	DNAJA3	0.8558	5.04E-03	9.28E-03	-3.4795
ENSG00000240403	KIR3DL2	1.1438	5.04E-03	9.28E-03	-3.4801
ENSG00000183454	GRIN2A	1.0280	5.05E-03	9.28E-03	-3.4803
ENSG00000144401	METTL21A	1.1325	5.05E-03	9.29E-03	-3.4809
ENSG00000112379	ARFGEF3	1.2314	5.05E-03	9.30E-03	-3.4816
ENSG00000127241	MASP1	1.0480	5.06E-03	9.30E-03	-3.4824
ENSG00000140807	NKD1	1.0277	5.06E-03	9.31E-03	-3.4827
ENSG00000146426	TIAM2	1.1158	5.07E-03	9.32E-03	-3.4846
ENSG00000113387	SUB1	0.8948	5.09E-03	9.36E-03	-3.4877
ENSG00000130528	HRC	1.1606	5.10E-03	9.37E-03	-3.4892
ENSG00000127928	GNGT1	1.0629	5.10E-03	9.37E-03	-3.4892
ENSG00000183048	SLC25A10	1.1420	5.10E-03	9.38E-03	-3.4903
ENSG00000240771	ARHGEF25	0.8103	5.11E-03	9.39E-03	-3.4909
ENSG00000213445	SIPA1	0.8740	5.11E-03	9.39E-03	-3.4911
ENSG00000119698	PPP4R4	1.0585	5.12E-03	9.41E-03	-3.4931
ENSG00000197061	H4C3	0.7852	5.12E-03	9.41E-03	-3.4931
ENSG00000187005	KRTAP21-1	0.9615	5.13E-03	9.43E-03	-3.4958
ENSG00000104980	TIMM44	1.1076	5.14E-03	9.44E-03	-3.4963
ENSG00000117594	HSD11B1	1.1824	5.14E-03	9.45E-03	-3.4975
ENSG00000183571	PGPEP1L	1.0491	5.16E-03	9.48E-03	-3.5001
ENSG00000212126	TAS2R50	1.0205	5.16E-03	9.48E-03	-3.5009
ENSG00000091262	ABCC6	1.1041	5.16E-03	9.49E-03	-3.5014
ENSG00000131477	RAMP2	1.3251	5.17E-03	9.49E-03	-3.5017
ENSG00000161642	ZNF385A	1.2021	5.17E-03	9.50E-03	-3.5027
ENSG00000128513	POT1	1.1280	5.17E-03	9.50E-03	-3.5030
ENSG00000249853	HS3ST5	1.0131	5.20E-03	9.56E-03	-3.5084
ENSG00000101425	BPI	1.2625	5.21E-03	9.56E-03	-3.5087
ENSG00000181751	MACIR	0.8537	5.21E-03	9.57E-03	-3.5095
ENSG00000204962	PCDHA8	1.0166	5.21E-03	9.57E-03	-3.5099
ENSG00000043514	TRIT1	1.1185	5.24E-03	9.62E-03	-3.5146
ENSG00000183128	CALHM3	1.1274	5.25E-03	9.64E-03	-3.5166
ENSG00000205560	CPT1B	1.2027	5.25E-03	9.64E-03	-3.5165
ENSG00000175334	BANF1	0.8617	5.26E-03	9.65E-03	-3.5174
ENSG00000021574	SPAST	1.1178	5.26E-03	9.65E-03	-3.5175
ENSG00000188691	OR56A5	1.0206	5.26E-03	9.65E-03	-3.5180

Gene ID	Gene Symbol	FC	P.Value	adj.P.Val	B
ENSG00000258405	ZNF578	1.0814	5.27E-03	9.67E-03	-3.5196
ENSG00000117676	RPS6KA1	1.1858	5.27E-03	9.67E-03	-3.5204
ENSG00000127318	IL22	1.1277	5.30E-03	9.72E-03	-3.5250
ENSG00000168481	LGI3	1.0101	5.32E-03	9.75E-03	-3.5273
ENSG00000141526	SLC16A3	1.2701	5.32E-03	9.75E-03	-3.5275
ENSG00000084092	NOA1	0.9079	5.33E-03	9.78E-03	-3.5303
ENSG00000109625	CPZ	1.0616	5.33E-03	9.78E-03	-3.5303
ENSG00000183395	PMCH	1.0874	5.34E-03	9.79E-03	-3.5320
ENSG00000132432	SEC61G	0.8938	5.34E-03	9.80E-03	-3.5323
ENSG00000164744	SUN3	1.1075	5.35E-03	9.81E-03	-3.5332
ENSG00000198000	NOL8	1.1052	5.36E-03	9.83E-03	-3.5352
ENSG00000171606	ZNF274	1.1564	5.36E-03	9.83E-03	-3.5353
ENSG00000124721	DNAH8	0.8689	5.38E-03	9.86E-03	-3.5387
ENSG00000182389	CACNB4	1.0710	5.38E-03	9.86E-03	-3.5391
ENSG00000242259	C22orf39	0.8984	5.38E-03	9.86E-03	-3.5391
ENSG00000165807	PPP1R36	0.9142	5.40E-03	9.90E-03	-3.5420
ENSG00000188211	NCR3LG1	0.8936	5.40E-03	9.90E-03	-3.5424
ENSG00000187754	SSX7	1.0617	5.42E-03	9.92E-03	-3.5444
ENSG00000159069	FBXW5	1.1173	5.42E-03	9.92E-03	-3.5450
ENSG00000256061	DNAAF4	1.1001	5.42E-03	9.92E-03	-3.5450
ENSG00000145700	ANKRD31	0.9295	5.42E-03	9.93E-03	-3.5454
ENSG00000074211	PPP2R2C	1.0606	5.43E-03	9.94E-03	-3.5462
ENSG00000142002	DPP9	1.1076	5.43E-03	9.94E-03	-3.5469
ENSG00000113924	HGD	1.1142	5.44E-03	9.95E-03	-3.5477
ENSG00000117069	ST6GALNAC5	1.0626	5.44E-03	9.95E-03	-3.5477
ENSG00000125741	OPA3	0.8824	5.44E-03	9.95E-03	-3.5480
ENSG00000171819	ANGPTL7	1.0122	5.44E-03	9.95E-03	-3.5483
ENSG00000127364	TAS2R4	0.9291	5.46E-03	9.98E-03	-3.5511
ENSG00000184719	RNLS	0.8681	5.46E-03	9.99E-03	-3.5518
ENSG00000167601	AXL	0.8176	5.46E-03	9.99E-03	-3.5519
ENSG00000112195	TREML2	1.3417	5.46E-03	9.99E-03	-3.5521
ENSG00000187676	B3GLCT	1.1324	5.48E-03	1.00E-02	-3.5543
ENSG00000204334	ERICH2	1.0823	5.48E-03	1.00E-02	-3.5545
ENSG00000066827	ZFAT	1.2192	5.48E-03	1.00E-02	-3.5551
ENSG00000106571	GLI3	0.7957	5.48E-03	1.00E-02	-3.5557
ENSG00000254986	DPP3	1.1331	5.50E-03	1.01E-02	-3.5588
ENSG00000137103	TMEM8B	1.1538	5.51E-03	1.01E-02	-3.5592
ENSG00000255071	SAA2-SAA4	0.9837	5.51E-03	1.01E-02	-3.5595
ENSG00000243566	UPK3B	1.1008	5.51E-03	1.01E-02	-3.5600
ENSG00000096088	PGC	1.0203	5.51E-03	1.01E-02	-3.5603
ENSG00000164011	ZNF691	0.8552	5.52E-03	1.01E-02	-3.5616
ENSG00000176454	LPCAT4	1.1714	5.52E-03	1.01E-02	-3.5619
ENSG00000090316	MAEA	1.1253	5.52E-03	1.01E-02	-3.5620
ENSG00000162897	FCAMR	1.0094	5.53E-03	1.01E-02	-3.5628
ENSG00000136231	IGF2BP3	1.1289	5.53E-03	1.01E-02	-3.5637
ENSG00000132199	ENOSF1	1.2663	5.54E-03	1.01E-02	-3.5648
ENSG00000135046	ANXA1	1.6332	5.54E-03	1.01E-02	-3.5649
ENSG00000115457	IGFBP2	1.1159	5.54E-03	1.01E-02	-3.5652
ENSG00000256671	LIMS4	1.0249	5.55E-03	1.01E-02	-3.5668
ENSG00000130377	ACSBG2	1.0693	5.55E-03	1.01E-02	-3.5670
ENSG00000116833	NR5A2	1.1013	5.57E-03	1.02E-02	-3.5689
ENSG00000108255	CRYBA1	1.0543	5.57E-03	1.02E-02	-3.5691
ENSG00000173369	C1QB	0.6380	5.57E-03	1.02E-02	-3.5699
ENSG00000111981	ULBP1	1.0827	5.58E-03	1.02E-02	-3.5708

Gene ID	Gene Symbol	FC	P.Value	adj.P.Val	B
ENSG00000128272	ATF4	1.1132	5.58E-03	1.02E-02	-3.5713
ENSG00000143341	HMCN1	1.0669	5.58E-03	1.02E-02	-3.5715
ENSG00000165124	SVEP1	1.0588	5.58E-03	1.02E-02	-3.5715
ENSG00000133101	CCNA1	1.1168	5.59E-03	1.02E-02	-3.5720
ENSG00000152056	AP1S3	1.1409	5.60E-03	1.02E-02	-3.5738
ENSG00000082996	RNF13	0.9019	5.60E-03	1.02E-02	-3.5752
ENSG00000145916	RMND5B	0.8754	5.63E-03	1.03E-02	-3.5785
ENSG00000136802	LRRC8A	1.1255	5.63E-03	1.03E-02	-3.5789
ENSG00000132026	RTBDN	0.7728	5.63E-03	1.03E-02	-3.5801
ENSG00000125734	GPR108	0.9144	5.65E-03	1.03E-02	-3.5825
ENSG00000102996	MMP15	1.0838	5.66E-03	1.03E-02	-3.5848
ENSG00000177697	CD151	1.1697	5.68E-03	1.03E-02	-3.5865
ENSG00000156009	MAGEA8	1.0332	5.68E-03	1.03E-02	-3.5869
ENSG00000100422	CERK	1.1605	5.69E-03	1.04E-02	-3.5885
ENSG00000142347	MYO1F	1.2932	5.69E-03	1.04E-02	-3.5886
ENSG00000154760	SLFN13	1.2311	5.69E-03	1.04E-02	-3.5890
ENSG00000215568	GAB4	1.1134	5.69E-03	1.04E-02	-3.5893
ENSG00000009694	TENM1	1.1210	5.70E-03	1.04E-02	-3.5911
ENSG00000152932	RAB3C	1.0480	5.71E-03	1.04E-02	-3.5922
ENSG00000180878	C11orf42	1.0317	5.72E-03	1.04E-02	-3.5942
ENSG00000108312	UBTF	1.1521	5.75E-03	1.05E-02	-3.5978
ENSG00000117971	CHRNB4	1.0974	5.76E-03	1.05E-02	-3.5998
ENSG00000140830	TXNL4B	1.1117	5.77E-03	1.05E-02	-3.6010
ENSG00000198722	UNC13B	0.8460	5.77E-03	1.05E-02	-3.6013
ENSG00000130592	LSP1	1.3994	5.78E-03	1.05E-02	-3.6025
ENSG00000188730	VWC2	1.0880	5.78E-03	1.05E-02	-3.6030
ENSG00000241233	KRTAP5-8	1.0441	5.80E-03	1.06E-02	-3.6065
ENSG00000215883	CYB5RL	1.1118	5.81E-03	1.06E-02	-3.6083
ENSG00000204482	LST1	0.7265	5.83E-03	1.06E-02	-3.6107
ENSG00000102531	FNDC3A	1.1600	5.83E-03	1.06E-02	-3.6112
ENSG00000186136	TAS2R42	1.0144	5.84E-03	1.06E-02	-3.6116
ENSG00000176022	B3GALT6	0.8653	5.84E-03	1.06E-02	-3.6121
ENSG00000101460	MAP1LC3A	1.2456	5.84E-03	1.06E-02	-3.6123
ENSG00000139517	LNX2	1.0891	5.86E-03	1.07E-02	-3.6152
ENSG00000121903	ZSCAN20	1.0907	5.86E-03	1.07E-02	-3.6157
ENSG00000168824	NSG1	1.1747	5.87E-03	1.07E-02	-3.6172
ENSG00000179934	CCR8	1.0051	5.87E-03	1.07E-02	-3.6171
ENSG00000099960	SLC7A4	1.0241	5.88E-03	1.07E-02	-3.6177
ENSG00000140350	ANP32A	0.8558	5.89E-03	1.07E-02	-3.6205
ENSG00000234068	PAGE2	1.1745	5.90E-03	1.07E-02	-3.6219
ENSG00000214941	ZSWIM7	0.8992	5.91E-03	1.07E-02	-3.6235
ENSG00000163932	PRKCD	0.8552	5.93E-03	1.08E-02	-3.6263
ENSG00000171234	UGT2B7	1.0808	5.94E-03	1.08E-02	-3.6274
ENSG00000241476	SSX2	1.0681	5.94E-03	1.08E-02	-3.6283
ENSG00000174844	DNAH12	1.1071	5.95E-03	1.08E-02	-3.6291
ENSG00000197859	ADAMTSL2	1.0461	5.95E-03	1.08E-02	-3.6293
ENSG00000167612	ANKRD33	1.0091	5.96E-03	1.08E-02	-3.6300
ENSG00000008282	SYPL1	1.3119	5.96E-03	1.08E-02	-3.6313
ENSG00000101363	MANBAL	0.8970	5.97E-03	1.08E-02	-3.6322
ENSG00000166816	LDHD	1.2386	5.97E-03	1.08E-02	-3.6326
ENSG00000166478	ZNF143	1.1107	5.99E-03	1.09E-02	-3.6347
ENSG00000170092	SPDYE5	1.1056	6.00E-03	1.09E-02	-3.6371
ENSG00000163440	PDCL2	1.0110	6.00E-03	1.09E-02	-3.6374
ENSG00000184500	PROS1	1.1111	6.01E-03	1.09E-02	-3.6379

Gene ID	Gene Symbol	FC	P.Value	adj.P.Val	B
ENSG00000196565	HBG2	0.7328	6.03E-03	1.09E-02	-3.6405
ENSG00000172889	EGFL7	0.8206	6.03E-03	1.09E-02	-3.6410
ENSG00000204713	TRIM27	0.8695	6.03E-03	1.10E-02	-3.6418
ENSG00000092036	HAUS4	1.1349	6.04E-03	1.10E-02	-3.6421
ENSG00000180305	WFDC10A	1.0170	6.04E-03	1.10E-02	-3.6426
ENSG00000011201	ANOS1	1.0930	6.05E-03	1.10E-02	-3.6444
ENSG00000167333	TRIM68	0.8604	6.06E-03	1.10E-02	-3.6453
ENSG00000117593	DARS2	1.1946	6.07E-03	1.10E-02	-3.6476
ENSG00000205090	TMEM240	1.0362	6.07E-03	1.10E-02	-3.6478
ENSG00000088256	GNA11	1.1619	6.11E-03	1.11E-02	-3.6533
ENSG00000035499	DEPDC1B	1.1958	6.13E-03	1.11E-02	-3.6558
ENSG00000158445	KCNB1	1.0176	6.13E-03	1.11E-02	-3.6561
ENSG00000100292	HMOX1	0.7060	6.14E-03	1.11E-02	-3.6572
ENSG00000107187	LHX3	1.0484	6.14E-03	1.11E-02	-3.6575
ENSG00000114805	PLCH1	1.0152	6.14E-03	1.11E-02	-3.6575
ENSG00000256045	MTRNR2L10	1.0140	6.14E-03	1.11E-02	-3.6576
ENSG00000213934	HBG1	0.8292	6.14E-03	1.11E-02	-3.6580
ENSG00000180992	MRPL14	0.8904	6.15E-03	1.12E-02	-3.6596
ENSG00000101200	AVP	1.0373	6.17E-03	1.12E-02	-3.6620
ENSG00000167377	ZNF23	0.8787	6.18E-03	1.12E-02	-3.6634
ENSG00000110243	APOA5	1.0125	6.18E-03	1.12E-02	-3.6639
ENSG00000115042	FAHD2A	1.1123	6.19E-03	1.12E-02	-3.6650
ENSG00000119878	CRIPT	0.8757	6.20E-03	1.12E-02	-3.6657
ENSG00000136444	RSAD1	0.8732	6.20E-03	1.12E-02	-3.6662
ENSG00000130948	HSD17B3	0.8647	6.22E-03	1.13E-02	-3.6688
ENSG00000172005	MAL	1.5461	6.22E-03	1.13E-02	-3.6694
ENSG00000005893	LAMP2	1.1352	6.23E-03	1.13E-02	-3.6702
ENSG00000189431	RASSF10	1.0045	6.23E-03	1.13E-02	-3.6706
ENSG00000198176	TFDP1	1.2096	6.23E-03	1.13E-02	-3.6712
ENSG00000105974	CAV1	0.7890	6.25E-03	1.13E-02	-3.6731
ENSG00000136108	CKAP2	1.1576	6.27E-03	1.13E-02	-3.6759
ENSG00000204314	PRRT1	0.8653	6.30E-03	1.14E-02	-3.6800
ENSG00000130270	ATP8B3	1.1400	6.30E-03	1.14E-02	-3.6804
ENSG00000119321	FKBP15	0.8913	6.31E-03	1.14E-02	-3.6819
ENSG00000100201	DDX17	0.8274	6.31E-03	1.14E-02	-3.6824
ENSG00000135097	MSI1	1.0563	6.31E-03	1.14E-02	-3.6823
ENSG00000248713	C4orf54	1.0060	6.33E-03	1.14E-02	-3.6842
ENSG00000105668	UPK1A	1.0302	6.33E-03	1.15E-02	-3.6854
ENSG00000005187	ACSM3	0.8915	6.34E-03	1.15E-02	-3.6857
ENSG00000198077	CYP2A7	1.0271	6.35E-03	1.15E-02	-3.6879
ENSG00000179455	MKRN3	1.2118	6.36E-03	1.15E-02	-3.6889
ENSG00000105499	PLA2G4C	1.1777	6.36E-03	1.15E-02	-3.6898
ENSG00000105383	CD33	0.7332	6.37E-03	1.15E-02	-3.6900
ENSG00000135298	ADGRB3	0.7723	6.37E-03	1.15E-02	-3.6911
ENSG00000114315	HES1	0.8127	6.37E-03	1.15E-02	-3.6912
ENSG00000167562	ZNF701	1.1607	6.38E-03	1.15E-02	-3.6915
ENSG00000250719	P3R3URF	1.0147	6.38E-03	1.15E-02	-3.6922
ENSG00000165078	CPA6	0.9109	6.40E-03	1.16E-02	-3.6955
ENSG00000135373	EHF	1.1278	6.42E-03	1.16E-02	-3.6978
ENSG00000063761	ADCK1	1.1392	6.43E-03	1.16E-02	-3.6993
ENSG00000183474	GTF2H2C	0.8662	6.43E-03	1.16E-02	-3.6993
ENSG00000105376	ICAM5	1.2137	6.43E-03	1.16E-02	-3.6996
ENSG00000250799	PRODH2	1.0057	6.43E-03	1.16E-02	-3.6997
ENSG00000138399	FASTKD1	0.8614	6.44E-03	1.16E-02	-3.7011

Gene ID	Gene Symbol	FC	P.Value	adj.P.Val	B
ENSG00000121742	GJB6	1.1778	6.48E-03	1.17E-02	-3.7058
ENSG00000149968	MMP3	1.0146	6.49E-03	1.17E-02	-3.7070
ENSG00000197506	SLC28A3	1.0953	6.50E-03	1.17E-02	-3.7094
ENSG00000125449	ARMC7	0.8786	6.52E-03	1.18E-02	-3.7111
ENSG00000140465	CYP1A1	1.0116	6.54E-03	1.18E-02	-3.7144
ENSG00000099849	RASSF7	0.8733	6.55E-03	1.18E-02	-3.7156
ENSG00000108733	PEX12	0.8804	6.55E-03	1.18E-02	-3.7159
ENSG00000064042	LIMCH1	1.1273	6.56E-03	1.18E-02	-3.7164
ENSG00000112763	BTN2A1	1.1075	6.57E-03	1.19E-02	-3.7190
ENSG00000168461	RAB31	1.3396	6.58E-03	1.19E-02	-3.7195
ENSG00000204006	C1orf185	1.0279	6.59E-03	1.19E-02	-3.7206
ENSG00000189064	GAGE2A	1.2181	6.60E-03	1.19E-02	-3.7226
ENSG00000066044	ELAVL1	1.1071	6.61E-03	1.19E-02	-3.7240
ENSG00000165280	VCP	1.1022	6.62E-03	1.19E-02	-3.7247
ENSG00000133067	LGR6	0.9428	6.62E-03	1.19E-02	-3.7253
ENSG00000011600	TYROBP	0.6781	6.65E-03	1.20E-02	-3.7287
ENSG00000197265	GTF2E2	1.1230	6.67E-03	1.20E-02	-3.7317
ENSG00000198822	GRM3	0.8892	6.67E-03	1.20E-02	-3.7325
ENSG00000185033	SEMA4B	0.8371	6.70E-03	1.21E-02	-3.7356
ENSG00000244020	MT1HL1	1.0545	6.70E-03	1.21E-02	-3.7363
ENSG00000095752	IL11	1.1076	6.72E-03	1.21E-02	-3.7386
ENSG00000214929	SPATA31D1	1.0570	6.74E-03	1.21E-02	-3.7416
ENSG00000173275	ZNF449	1.1248	6.74E-03	1.22E-02	-3.7417
ENSG00000163703	CRELD1	0.8786	6.76E-03	1.22E-02	-3.7437
ENSG00000174428	GTF2IRD2B	1.1153	6.76E-03	1.22E-02	-3.7436
ENSG00000173992	CCS	0.8723	6.76E-03	1.22E-02	-3.7446
ENSG00000112293	GPLD1	1.1432	6.77E-03	1.22E-02	-3.7452
ENSG00000161905	ALOX15	0.8603	6.77E-03	1.22E-02	-3.7454
ENSG00000188393	CLEC2A	1.0068	6.77E-03	1.22E-02	-3.7454
ENSG00000112837	TBX18	1.0351	6.78E-03	1.22E-02	-3.7465
ENSG00000115289	PCGF1	1.1057	6.78E-03	1.22E-02	-3.7464
ENSG00000111199	TRPV4	0.9368	6.79E-03	1.22E-02	-3.7479
ENSG00000066294	CD84	1.2038	6.79E-03	1.22E-02	-3.7481
ENSG00000102977	ACD	1.1264	6.79E-03	1.22E-02	-3.7484
ENSG00000076826	CAMSAP3	1.2007	6.80E-03	1.22E-02	-3.7489
ENSG00000197465	GYPE	0.8612	6.80E-03	1.22E-02	-3.7498
ENSG00000214021	TTLL3	1.1587	6.83E-03	1.23E-02	-3.7535
ENSG00000111863	ADTRP	0.6641	6.83E-03	1.23E-02	-3.7539
ENSG00000147853	AK3	0.8948	6.84E-03	1.23E-02	-3.7550
ENSG00000147873	IFNA5	1.0268	6.86E-03	1.23E-02	-3.7577
ENSG00000137936	BCAR3	1.2577	6.88E-03	1.24E-02	-3.7595
ENSG00000175482	POLD4	0.8720	6.89E-03	1.24E-02	-3.7611
ENSG00000092009	CMA1	1.0294	6.90E-03	1.24E-02	-3.7622
ENSG00000244411	KRTAP5-7	1.0295	6.90E-03	1.24E-02	-3.7626
ENSG00000101144	BMP7	1.0295	6.91E-03	1.24E-02	-3.7633
ENSG00000204922	UQCC3	0.9135	6.92E-03	1.24E-02	-3.7648
ENSG00000170439	METTL7B	0.9129	6.93E-03	1.25E-02	-3.7657
ENSG00000111671	SPSB2	0.8713	6.95E-03	1.25E-02	-3.7686
ENSG00000160282	FTCD	0.8681	6.95E-03	1.25E-02	-3.7694
ENSG00000146386	ABRACL	0.8370	6.99E-03	1.26E-02	-3.7745
ENSG00000119335	SET	1.0987	7.00E-03	1.26E-02	-3.7759
ENSG00000196811	CHRNG	1.0358	7.01E-03	1.26E-02	-3.7762
ENSG00000127903	ZNF835	1.0788	7.01E-03	1.26E-02	-3.7768
ENSG00000162078	ZG16B	1.1055	7.03E-03	1.26E-02	-3.7789

Gene ID	Gene Symbol	FC	P.Value	adj.P.Val	B
ENSG00000171314	PGAM1	1.0940	7.03E-03	1.26E-02	-3.7790
ENSG00000141905	NFIC	1.1560	7.03E-03	1.26E-02	-3.7793
ENSG00000219607	PPP1R3G	1.1401	7.10E-03	1.28E-02	-3.7887
ENSG00000196090	PTPRT	0.9358	7.11E-03	1.28E-02	-3.7900
ENSG00000198841	KTI12	1.1038	7.12E-03	1.28E-02	-3.7906
ENSG00000088926	F11	1.0429	7.13E-03	1.28E-02	-3.7915
ENSG00000125492	BARHL1	1.0109	7.13E-03	1.28E-02	-3.7915
ENSG00000187079	TEAD1	1.3298	7.14E-03	1.28E-02	-3.7927
ENSG00000123454	DBH	1.1136	7.14E-03	1.28E-02	-3.7936
ENSG00000099804	CDC34	1.0961	7.15E-03	1.28E-02	-3.7939
ENSG00000164690	SHH	0.9476	7.15E-03	1.28E-02	-3.7944
ENSG00000243943	ZNF512	0.8531	7.17E-03	1.29E-02	-3.7975
ENSG00000101160	CTSZ	1.1720	7.18E-03	1.29E-02	-3.7980
ENSG00000182359	KBTBD3	0.8978	7.19E-03	1.29E-02	-3.7996
ENSG00000184436	THAP7	0.8919	7.19E-03	1.29E-02	-3.7996
ENSG00000074771	NOX3	1.0062	7.19E-03	1.29E-02	-3.7998
ENSG00000254870	ATP6V1G2-DDX39B	0.8334	7.19E-03	1.29E-02	-3.8000
ENSG00000120686	UFM1	1.1193	7.20E-03	1.29E-02	-3.8007
ENSG00000119547	ONECUT2	1.0394	7.20E-03	1.29E-02	-3.8009
ENSG00000120327	PCDHB14	0.8572	7.23E-03	1.30E-02	-3.8039
ENSG00000196628	TCF4	0.7562	7.23E-03	1.30E-02	-3.8049
ENSG00000133488	SEC14L4	1.0447	7.24E-03	1.30E-02	-3.8059
ENSG00000134569	LRP4	1.0564	7.24E-03	1.30E-02	-3.8057
ENSG00000198832	SELENOM	0.8095	7.24E-03	1.30E-02	-3.8059
ENSG00000043462	LCP2	1.4108	7.25E-03	1.30E-02	-3.8068
ENSG00000164532	TBX20	1.0161	7.25E-03	1.30E-02	-3.8071
ENSG00000164626	KCNK5	1.0555	7.27E-03	1.30E-02	-3.8093
ENSG00000145808	ADAMTS19	0.9240	7.27E-03	1.30E-02	-3.8094
ENSG00000163666	HESX1	0.8636	7.30E-03	1.31E-02	-3.8129
ENSG00000214078	CPNE1	1.1313	7.32E-03	1.31E-02	-3.8153
ENSG00000133710	SPINK5	1.3012	7.32E-03	1.31E-02	-3.8156
ENSG00000167460	TPM4	1.2055	7.33E-03	1.31E-02	-3.8167
ENSG00000143970	ASXL2	1.1517	7.33E-03	1.31E-02	-3.8170
ENSG00000101079	NDRG3	0.8613	7.34E-03	1.31E-02	-3.8181
ENSG00000109610	SOD3	1.0469	7.34E-03	1.31E-02	-3.8182
ENSG00000146090	RASGEF1C	1.0332	7.35E-03	1.32E-02	-3.8189
ENSG00000103023	PRSS54	1.0140	7.37E-03	1.32E-02	-3.8217
ENSG00000155962	CLIC2	1.2826	7.37E-03	1.32E-02	-3.8222
ENSG00000105135	ILVBL	1.0922	7.39E-03	1.32E-02	-3.8235
ENSG00000053108	FSTL4	1.1726	7.41E-03	1.33E-02	-3.8260
ENSG00000187902	SHISA7	1.0094	7.43E-03	1.33E-02	-3.8290
ENSG00000189134	NKAPL	0.9491	7.43E-03	1.33E-02	-3.8294
ENSG00000166136	NDUFB8	1.0823	7.45E-03	1.33E-02	-3.8309
ENSG00000159228	CBR1	0.7985	7.46E-03	1.33E-02	-3.8323
ENSG00000225110	PNMA6F	1.0050	7.47E-03	1.34E-02	-3.8343
ENSG00000183044	ABAT	1.1074	7.48E-03	1.34E-02	-3.8347
ENSG00000171759	PAH	1.1253	7.49E-03	1.34E-02	-3.8357
ENSG00000187021	PNLIPRP1	1.0657	7.49E-03	1.34E-02	-3.8366
ENSG00000123200	ZC3H13	1.0987	7.50E-03	1.34E-02	-3.8371
ENSG00000101180	HRH3	1.0073	7.50E-03	1.34E-02	-3.8377
ENSG00000147202	DIAPH2	1.2166	7.52E-03	1.34E-02	-3.8392
ENSG00000203756	TMEM244	0.8609	7.52E-03	1.34E-02	-3.8395
ENSG00000185551	NR2F2	1.1492	7.52E-03	1.34E-02	-3.8399
ENSG00000156162	DPY19L4	1.1232	7.53E-03	1.35E-02	-3.8408

Gene ID	Gene Symbol	FC	P.Value	adj.P.Val	B
ENSG00000179058	C9orf50	1.0085	7.53E-03	1.35E-02	-3.8411
ENSG00000154764	WNT7A	1.0070	7.54E-03	1.35E-02	-3.8421
ENSG00000139352	ASCL1	1.0891	7.54E-03	1.35E-02	-3.8425
ENSG00000147896	IFNK	1.0082	7.54E-03	1.35E-02	-3.8426
ENSG00000130429	ARPC1B	1.1517	7.57E-03	1.35E-02	-3.8452
ENSG00000147145	LPAR4	1.0755	7.57E-03	1.35E-02	-3.8455
ENSG00000130755	GMFG	0.8613	7.58E-03	1.35E-02	-3.8463
ENSG00000065534	MYLK	1.2510	7.58E-03	1.35E-02	-3.8464
ENSG00000165548	TMEM63C	0.7765	7.59E-03	1.36E-02	-3.8479
ENSG00000111432	FZD10	1.0039	7.59E-03	1.36E-02	-3.8483
ENSG00000151388	ADAMTS12	1.0335	7.60E-03	1.36E-02	-3.8495
ENSG00000012124	CD22	1.1151	7.62E-03	1.36E-02	-3.8518
ENSG00000122592	HOXA7	0.8150	7.62E-03	1.36E-02	-3.8517
ENSG00000133318	RTN3	1.1229	7.62E-03	1.36E-02	-3.8517
ENSG00000155906	RMND1	0.9000	7.62E-03	1.36E-02	-3.8518
ENSG00000184022	OR2T10	1.0112	7.62E-03	1.36E-02	-3.8520
ENSG00000073792	IGF2BP2	1.1690	7.64E-03	1.36E-02	-3.8543
ENSG00000165194	PCDH19	1.0063	7.65E-03	1.36E-02	-3.8547
ENSG00000065057	NTHL1	0.8019	7.65E-03	1.37E-02	-3.8555
ENSG00000188368	PRR19	1.0692	7.66E-03	1.37E-02	-3.8558
ENSG00000203965	EFCAB7	1.1354	7.66E-03	1.37E-02	-3.8561
ENSG00000159314	ARHGAP27	1.1527	7.66E-03	1.37E-02	-3.8568
ENSG00000198774	RASSF9	1.0049	7.67E-03	1.37E-02	-3.8580
ENSG00000065518	NDUFB4	0.9211	7.68E-03	1.37E-02	-3.8581
ENSG00000122643	NT5C3A	0.8870	7.69E-03	1.37E-02	-3.8594
ENSG00000112053	SLC26A8	1.0817	7.69E-03	1.37E-02	-3.8601
ENSG00000171346	KRT15	1.0758	7.69E-03	1.37E-02	-3.8602
ENSG00000203870	SMIM9	1.0490	7.70E-03	1.37E-02	-3.8605
ENSG00000106992	AK1	1.1956	7.71E-03	1.37E-02	-3.8619
ENSG00000182103	FAM181B	1.0459	7.72E-03	1.38E-02	-3.8633
ENSG00000197888	UGT2B17	0.6856	7.74E-03	1.38E-02	-3.8650
ENSG00000100591	AHSA1	1.1232	7.76E-03	1.38E-02	-3.8676
ENSG00000106689	LHX2	1.0340	7.77E-03	1.38E-02	-3.8687
ENSG00000100490	CDKL1	1.1378	7.78E-03	1.39E-02	-3.8697
ENSG00000128346	C22orf23	1.0941	7.78E-03	1.39E-02	-3.8700
ENSG00000122877	EGR2	0.7465	7.78E-03	1.39E-02	-3.8703
ENSG00000179593	ALOX15B	1.0500	7.78E-03	1.39E-02	-3.8705
ENSG00000178947	SMIM10L2A	1.0761	7.79E-03	1.39E-02	-3.8711
ENSG00000171916	LGALS9C	1.1027	7.82E-03	1.39E-02	-3.8752
ENSG00000125772	GPCPD1	1.1517	7.83E-03	1.39E-02	-3.8757
ENSG00000138115	CYP2C8	1.1390	7.86E-03	1.40E-02	-3.8792
ENSG00000065457	ADAT1	1.1513	7.87E-03	1.40E-02	-3.8801
ENSG00000165118	C9orf64	0.8616	7.87E-03	1.40E-02	-3.8800
ENSG00000222014	RAB6C	1.0580	7.87E-03	1.40E-02	-3.8809
ENSG00000203910	C1orf146	1.0446	7.87E-03	1.40E-02	-3.8810
ENSG00000182272	B4GALNT4	1.0467	7.88E-03	1.40E-02	-3.8813
ENSG00000137440	FGFBP1	1.0052	7.89E-03	1.40E-02	-3.8828
ENSG00000143416	SELENBP1	0.8226	7.91E-03	1.41E-02	-3.8847
ENSG00000251322	SHANK3	1.0456	7.92E-03	1.41E-02	-3.8858
ENSG00000271303	SRXN1	1.1403	7.93E-03	1.41E-02	-3.8872
ENSG00000206531	CD200R1L	1.0132	7.93E-03	1.41E-02	-3.8873
ENSG00000176635	HORMAD2	1.0546	7.93E-03	1.41E-02	-3.8878
ENSG00000270136	MICOS10-NBL1	0.9283	7.94E-03	1.41E-02	-3.8881
ENSG00000063438	AHRR	0.8965	7.95E-03	1.41E-02	-3.8894

Gene ID	Gene Symbol	FC	P.Value	adj.P.Val	B
ENSG00000104904	OAZ1	0.9265	7.95E-03	1.41E-02	-3.8894
ENSG00000157450	RNF111	1.1067	7.95E-03	1.41E-02	-3.8900
ENSG00000181019	NQO1	1.1473	7.96E-03	1.42E-02	-3.8911
ENSG00000170606	HSPA4	1.0901	7.99E-03	1.42E-02	-3.8940
ENSG00000198363	ASPH	0.8510	8.01E-03	1.42E-02	-3.8960
ENSG00000256294	ZNF225	1.0941	8.03E-03	1.43E-02	-3.8980
ENSG00000177105	RHOG	1.1333	8.03E-03	1.43E-02	-3.8990
ENSG00000183576	SETD3	1.0985	8.04E-03	1.43E-02	-3.8994
ENSG00000214842	RAD51AP2	1.0088	8.05E-03	1.43E-02	-3.9007
ENSG00000034713	GABARAPL2	1.1218	8.06E-03	1.43E-02	-3.9014
ENSG00000125885	MCM8	1.1814	8.06E-03	1.43E-02	-3.9015
ENSG00000213714	FAM209B	1.0955	8.06E-03	1.43E-02	-3.9023
ENSG00000204983	PRSS1	1.0649	8.08E-03	1.44E-02	-3.9041
ENSG00000175854	SWI5	0.9021	8.11E-03	1.44E-02	-3.9068
ENSG00000109790	KLHL5	1.1898	8.13E-03	1.44E-02	-3.9096
ENSG00000135778	NTPCR	1.1653	8.13E-03	1.44E-02	-3.9097
ENSG00000179528	LBX2	0.8995	8.14E-03	1.44E-02	-3.9104
ENSG00000144283	PKP4	1.1873	8.14E-03	1.44E-02	-3.9105
ENSG00000182175	RGMA	1.0548	8.14E-03	1.45E-02	-3.9108
ENSG00000145536	ADAMTS16	1.0379	8.15E-03	1.45E-02	-3.9114
ENSG00000238269	PAGE2B	1.1645	8.15E-03	1.45E-02	-3.9118
ENSG00000154719	MRPL39	1.1100	8.15E-03	1.45E-02	-3.9123
ENSG00000091127	PUS7	0.8424	8.17E-03	1.45E-02	-3.9144
ENSG00000028137	TNFRSF1B	1.2482	8.18E-03	1.45E-02	-3.9154
ENSG00000146166	LGSN	1.1002	8.19E-03	1.45E-02	-3.9160
ENSG00000033170	FUT8	0.7862	8.19E-03	1.45E-02	-3.9165
ENSG00000155393	HEATR3	0.8480	8.21E-03	1.46E-02	-3.9180
ENSG00000114745	GORASP1	1.1180	8.21E-03	1.46E-02	-3.9187
ENSG00000144736	SHQ1	1.1586	8.22E-03	1.46E-02	-3.9193
ENSG00000122481	RWDD3	1.1192	8.24E-03	1.46E-02	-3.9216
ENSG00000178217	SH2D4B	1.0186	8.24E-03	1.46E-02	-3.9215
ENSG00000255223	OR5M11	1.0181	8.24E-03	1.46E-02	-3.9221
ENSG00000144182	LIPT1	0.8850	8.25E-03	1.46E-02	-3.9222
ENSG00000006210	CX3CL1	1.0176	8.26E-03	1.46E-02	-3.9240
ENSG00000113296	THBS4	1.1299	8.28E-03	1.47E-02	-3.9258
ENSG00000125148	MT2A	1.3661	8.31E-03	1.47E-02	-3.9289
ENSG00000153531	ADPRHL1	0.8982	8.32E-03	1.47E-02	-3.9298
ENSG00000197646	PDCD1LG2	0.8598	8.32E-03	1.48E-02	-3.9307
ENSG00000088538	DOCK3	0.8316	8.34E-03	1.48E-02	-3.9322
ENSG00000101440	ASIP	1.0737	8.35E-03	1.48E-02	-3.9330
ENSG00000188321	ZNF559	0.8106	8.35E-03	1.48E-02	-3.9332
ENSG00000187559	FOXD4L3	0.9661	8.36E-03	1.48E-02	-3.9344
ENSG00000111058	ACSS3	1.1317	8.37E-03	1.48E-02	-3.9357
ENSG00000111348	ARHGDIB	1.2069	8.37E-03	1.48E-02	-3.9359
ENSG00000157680	DGKI	0.7863	8.37E-03	1.48E-02	-3.9361
ENSG00000204614	TRIM40	1.0142	8.39E-03	1.49E-02	-3.9380
ENSG00000177108	ZDHHC22	1.0091	8.41E-03	1.49E-02	-3.9398
ENSG00000127838	PNKD	1.1182	8.43E-03	1.49E-02	-3.9417
ENSG00000168269	FOXI1	1.0548	8.46E-03	1.50E-02	-3.9449
ENSG00000130921	MTRFR	1.1131	8.46E-03	1.50E-02	-3.9457
ENSG00000162711	NLRP3	1.1509	8.50E-03	1.50E-02	-3.9490
ENSG00000169474	SPRR1A	1.0378	8.50E-03	1.50E-02	-3.9498
ENSG00000169689	CENPX	1.1152	8.52E-03	1.51E-02	-3.9510
ENSG00000187026	KRTAP21-2	0.9647	8.52E-03	1.51E-02	-3.9517

Gene ID	Gene Symbol	FC	P.Value	adj.P.Val	B
ENSG00000261115	TMEM178B	1.0517	8.52E-03	1.51E-02	-3.9519
ENSG00000165898	ISCA2	0.8889	8.53E-03	1.51E-02	-3.9526
ENSG00000115944	COX7A2L	0.9332	8.54E-03	1.51E-02	-3.9537
ENSG00000149527	PLCH2	1.2190	8.54E-03	1.51E-02	-3.9540
ENSG00000164708	PGAM2	1.2332	8.55E-03	1.51E-02	-3.9545
ENSG00000189403	HMGB1	1.1060	8.55E-03	1.51E-02	-3.9547
ENSG00000198754	OXCT2	1.2113	8.55E-03	1.51E-02	-3.9547
ENSG00000118322	ATP10B	0.6568	8.57E-03	1.52E-02	-3.9568
ENSG00000133124	IRS4	1.0419	8.60E-03	1.52E-02	-3.9594
ENSG00000186765	FSCN2	0.8727	8.60E-03	1.52E-02	-3.9597
ENSG00000196388	INCA1	0.8691	8.60E-03	1.52E-02	-3.9601
ENSG00000178015	GPR150	0.8877	8.62E-03	1.52E-02	-3.9616
ENSG00000079393	DUSP13	0.8015	8.65E-03	1.53E-02	-3.9652
ENSG00000121075	TBX4	1.0275	8.66E-03	1.53E-02	-3.9663
ENSG00000234511	C5orf58	1.2149	8.67E-03	1.53E-02	-3.9670
ENSG00000107821	KAZALD1	1.1432	8.67E-03	1.53E-02	-3.9674
ENSG00000204583	LRCOL1	1.0769	8.70E-03	1.54E-02	-3.9700
ENSG00000155052	CNTNAP5	1.0435	8.73E-03	1.54E-02	-3.9732
ENSG00000137100	DCTN3	0.9156	8.73E-03	1.54E-02	-3.9734
ENSG00000184293	CLECL1	1.2971	8.76E-03	1.55E-02	-3.9765
ENSG00000188095	MESP2	1.1101	8.77E-03	1.55E-02	-3.9773
ENSG00000178814	OPLAH	1.2417	8.78E-03	1.55E-02	-3.9780
ENSG00000122786	CALD1	1.1315	8.80E-03	1.55E-02	-3.9803
ENSG00000229474	PATL2	1.1950	8.82E-03	1.56E-02	-3.9824
ENSG00000237440	ZNF737	1.2203	8.83E-03	1.56E-02	-3.9835
ENSG00000196967	ZNF585A	1.2095	8.84E-03	1.56E-02	-3.9842
ENSG00000196411	EPHB4	1.1256	8.84E-03	1.56E-02	-3.9846
ENSG00000108479	GALK1	0.8861	8.84E-03	1.56E-02	-3.9847
ENSG00000099940	SNAP29	1.1061	8.85E-03	1.56E-02	-3.9855
ENSG00000123405	NFE2	0.8252	8.86E-03	1.56E-02	-3.9865
ENSG00000133739	LRRCC1	1.1858	8.86E-03	1.56E-02	-3.9870
ENSG00000159079	CFAP298	1.1134	8.87E-03	1.56E-02	-3.9875
ENSG00000128016	ZFP36	1.1996	8.91E-03	1.57E-02	-3.9920
ENSG00000180828	BHLHE22	1.0857	8.92E-03	1.57E-02	-3.9927
ENSG00000168016	TRANK1	0.8177	8.94E-03	1.58E-02	-3.9943
ENSG00000130193	THEM6	0.8484	8.98E-03	1.58E-02	-3.9984
ENSG00000086200	IPO11	1.1102	8.98E-03	1.58E-02	-3.9987
ENSG00000203685	STUM	1.0447	8.99E-03	1.58E-02	-3.9992
ENSG00000121210	TMEM131L	1.2401	8.99E-03	1.58E-02	-3.9997
ENSG00000198498	TMA16	0.8958	8.99E-03	1.58E-02	-3.9996
ENSG00000151572	ANO4	1.0454	9.02E-03	1.59E-02	-4.0029
ENSG00000196932	TMEM26	0.9254	9.04E-03	1.59E-02	-4.0040
ENSG00000140471	LINS1	0.8801	9.04E-03	1.59E-02	-4.0042
ENSG00000160111	CPAMD8	1.1396	9.04E-03	1.59E-02	-4.0044
ENSG00000115840	SLC25A12	0.9007	9.04E-03	1.59E-02	-4.0046
ENSG00000123472	ATPAF1	0.9028	9.05E-03	1.59E-02	-4.0056
ENSG00000135077	HAVCR2	1.1612	9.06E-03	1.60E-02	-4.0066
ENSG00000140548	ZNF710	0.8846	9.07E-03	1.60E-02	-4.0078
ENSG00000187840	EIF4EBP1	1.2134	9.09E-03	1.60E-02	-4.0095
ENSG00000120500	ARR3	1.0808	9.09E-03	1.60E-02	-4.0098
ENSG00000132591	ERAL1	1.1034	9.10E-03	1.60E-02	-4.0101
ENSG00000162006	MSLNL	1.0077	9.10E-03	1.60E-02	-4.0101
ENSG00000223953	C1QTNF5	0.9102	9.10E-03	1.60E-02	-4.0100
ENSG00000108602	ALDH3A1	1.0252	9.10E-03	1.60E-02	-4.0108

Gene ID	Gene Symbol	FC	P.Value	adj.P.Val	B
ENSG00000117450	PRDX1	0.8440	9.11E-03	1.60E-02	-4.0109
ENSG00000001167	NFYA	1.1337	9.11E-03	1.60E-02	-4.0116
ENSG00000079459	FDFT1	1.1416	9.15E-03	1.61E-02	-4.0154
ENSG00000230124	ACBD6	1.1543	9.15E-03	1.61E-02	-4.0156
ENSG00000181031	RPH3AL	0.8404	9.16E-03	1.61E-02	-4.0159
ENSG00000134070	IRAK2	1.2266	9.18E-03	1.61E-02	-4.0179
ENSG00000102743	SLC25A15	0.8716	9.19E-03	1.62E-02	-4.0192
ENSG00000136997	MYC	0.7386	9.20E-03	1.62E-02	-4.0199
ENSG00000122547	EEPD1	1.1297	9.22E-03	1.62E-02	-4.0221
ENSG00000164902	PHAX	1.1250	9.23E-03	1.62E-02	-4.0230
ENSG00000214194	SMIM30	0.8480	9.23E-03	1.62E-02	-4.0230
ENSG00000102401	ARMCX3	1.1261	9.24E-03	1.62E-02	-4.0236
ENSG00000204301	NOTCH4	1.1047	9.25E-03	1.63E-02	-4.0247
ENSG00000197360	ZNF98	1.0482	9.26E-03	1.63E-02	-4.0258
ENSG00000140678	ITGAX	1.2704	9.30E-03	1.63E-02	-4.0296
ENSG00000171053	PATE1	1.0053	9.32E-03	1.64E-02	-4.0316
ENSG00000229314	ORM1	1.2405	9.32E-03	1.64E-02	-4.0318
ENSG00000238210	ETDA	1.0709	9.34E-03	1.64E-02	-4.0339
ENSG00000184220	CMSS1	0.8952	9.35E-03	1.64E-02	-4.0343
ENSG00000167107	ACSF2	1.1447	9.35E-03	1.64E-02	-4.0346
ENSG00000008311	AASS	1.2024	9.36E-03	1.64E-02	-4.0358
ENSG00000184584	STING1	1.3466	9.37E-03	1.65E-02	-4.0366
ENSG00000136986	DERL1	1.0957	9.38E-03	1.65E-02	-4.0375
ENSG00000164929	BAALC	1.0740	9.38E-03	1.65E-02	-4.0378
ENSG00000213096	ZNF254	1.1992	9.39E-03	1.65E-02	-4.0387
ENSG00000160180	TFF3	0.8453	9.39E-03	1.65E-02	-4.0388
ENSG00000167617	CDC42EP5	1.0727	9.45E-03	1.66E-02	-4.0444
ENSG00000184984	CHRM5	1.0429	9.47E-03	1.66E-02	-4.0455
ENSG00000187800	PEAR1	1.1806	9.48E-03	1.66E-02	-4.0465
ENSG00000117335	CD46	1.1514	9.48E-03	1.66E-02	-4.0471
ENSG00000102967	DHODH	0.8543	9.50E-03	1.67E-02	-4.0490
ENSG00000154096	THY1	1.1901	9.51E-03	1.67E-02	-4.0500
ENSG00000043039	BARX2	0.6685	9.54E-03	1.67E-02	-4.0524
ENSG00000214140	PRCD	0.8247	9.54E-03	1.67E-02	-4.0526
ENSG00000160360	GPSM1	1.1071	9.57E-03	1.68E-02	-4.0549
ENSG00000145293	ENOPH1	1.0953	9.57E-03	1.68E-02	-4.0555
ENSG00000117154	IGSF21	0.8771	9.58E-03	1.68E-02	-4.0559
ENSG00000134285	FKBP11	0.8729	9.60E-03	1.68E-02	-4.0581
ENSG00000213563	C8orf82	1.1663	9.63E-03	1.69E-02	-4.0606
ENSG00000153936	HS2ST1	0.8552	9.63E-03	1.69E-02	-4.0612
ENSG00000167674	HDGFL2	0.8941	9.65E-03	1.69E-02	-4.0625
ENSG00000134028	ADAMDEC1	1.0895	9.65E-03	1.69E-02	-4.0630
ENSG00000036448	MYOM2	1.2342	9.67E-03	1.70E-02	-4.0645
ENSG00000148331	ASB6	1.0896	9.71E-03	1.70E-02	-4.0678
ENSG00000119673	ACOT2	1.1522	9.72E-03	1.70E-02	-4.0688
ENSG00000173809	TDRD12	1.0978	9.72E-03	1.70E-02	-4.0688
ENSG00000185090	MANEAL	0.8388	9.72E-03	1.70E-02	-4.0688
ENSG00000104826	LHB	0.8873	9.75E-03	1.71E-02	-4.0715
ENSG00000174473	GALNTL6	1.0382	9.77E-03	1.71E-02	-4.0733
ENSG00000163463	KRTCAP2	1.1583	9.82E-03	1.72E-02	-4.0780
ENSG00000070214	SLC44A1	0.8523	9.82E-03	1.72E-02	-4.0787
ENSG00000081177	EXD2	0.8748	9.83E-03	1.72E-02	-4.0788
ENSG00000116667	C1orf21	1.3574	9.83E-03	1.72E-02	-4.0795
ENSG00000188612	SUMO2	1.0805	9.84E-03	1.72E-02	-4.0800

Gene ID	Gene Symbol	FC	P.Value	adj.P.Val	B
ENSG00000255251	PRR23D1	1.0184	9.84E-03	1.72E-02	-4.0800
ENSG00000144355	DLX1	0.9641	9.85E-03	1.73E-02	-4.0812
ENSG00000204671	IL31	1.0116	9.85E-03	1.73E-02	-4.0814
ENSG00000030419	IKZF2	1.1497	9.86E-03	1.73E-02	-4.0819
ENSG00000183155	RABIF	1.1383	9.86E-03	1.73E-02	-4.0822
ENSG00000081148	IMPG2	1.0533	9.87E-03	1.73E-02	-4.0831
ENSG00000155980	KIF5A	1.0720	9.90E-03	1.73E-02	-4.0853
ENSG00000101280	ANGPT4	1.0271	9.90E-03	1.73E-02	-4.0858
ENSG00000198128	OR2L3	1.0087	9.93E-03	1.74E-02	-4.0886
ENSG00000184254	ALDH1A3	0.8915	9.94E-03	1.74E-02	-4.0890
ENSG00000004455	AK2	0.8794	9.97E-03	1.74E-02	-4.0919
ENSG00000181481	RNF135	1.1106	9.99E-03	1.75E-02	-4.0938
ENSG00000078596	ITM2A	1.2864	9.99E-03	1.75E-02	-4.0940
ENSG00000174151	CYB561D1	0.8917	1.00E-02	1.75E-02	-4.0960
ENSG00000185666	SYN3	1.1219	1.00E-02	1.75E-02	-4.0965
ENSG00000234438	KBTBD13	1.0059	1.00E-02	1.76E-02	-4.0988
ENSG00000164303	ENPP6	0.8883	1.01E-02	1.76E-02	-4.0995
ENSG00000214107	MAGEB1	1.1623	1.01E-02	1.76E-02	-4.0998
ENSG00000183644	HOATZ	1.0306	1.01E-02	1.76E-02	-4.1012
ENSG00000262874	C19orf84	1.0365	1.01E-02	1.76E-02	-4.1022
ENSG00000040633	PHF23	0.8757	1.01E-02	1.77E-02	-4.1054
ENSG00000171481	OR1L3	0.9725	1.01E-02	1.77E-02	-4.1063
ENSG00000128872	TMOD2	0.8479	1.02E-02	1.77E-02	-4.1080
ENSG00000006118	TMEM132A	1.1957	1.02E-02	1.77E-02	-4.1081
ENSG00000127152	BCL11B	1.0322	1.02E-02	1.78E-02	-4.1097
ENSG00000067704	IARS2	1.1533	1.02E-02	1.78E-02	-4.1115
ENSG00000182534	MXRA7	1.2732	1.02E-02	1.78E-02	-4.1118
ENSG00000104537	ANXA13	1.0956	1.02E-02	1.78E-02	-4.1135
ENSG00000256870	SLC5A8	1.0061	1.02E-02	1.79E-02	-4.1147
ENSG00000044459	CNTLN	1.1306	1.02E-02	1.79E-02	-4.1150
ENSG00000115590	IL1R2	1.3094	1.02E-02	1.79E-02	-4.1151
ENSG00000118972	FGF23	1.0155	1.02E-02	1.79E-02	-4.1161
ENSG00000213995	NAXD	0.8622	1.03E-02	1.80E-02	-4.1203
ENSG00000100604	CHGA	1.0241	1.03E-02	1.80E-02	-4.1213
ENSG00000077152	UBE2T	1.2598	1.03E-02	1.80E-02	-4.1226
ENSG00000129654	FOXJ1	1.1617	1.03E-02	1.80E-02	-4.1228
ENSG00000212127	TAS2R14	1.1029	1.03E-02	1.80E-02	-4.1235
ENSG00000148600	CDHR1	1.1867	1.03E-02	1.81E-02	-4.1246
ENSG00000123096	SSPN	0.8295	1.03E-02	1.81E-02	-4.1251
ENSG00000214113	LYRM4	0.9098	1.04E-02	1.81E-02	-4.1261
ENSG00000228049	POLR2J2	1.1905	1.04E-02	1.81E-02	-4.1268
ENSG00000135222	CSN2	1.0515	1.04E-02	1.81E-02	-4.1275
ENSG00000181449	SOX2	1.1656	1.04E-02	1.81E-02	-4.1283
ENSG00000173093	CCDC63	1.0190	1.04E-02	1.81E-02	-4.1284
ENSG00000204335	SP5	1.0174	1.04E-02	1.81E-02	-4.1287
ENSG00000105889	STEAP1B	0.8018	1.04E-02	1.81E-02	-4.1293
ENSG00000240344	PPIL3	1.0897	1.04E-02	1.82E-02	-4.1317
ENSG00000163785	RYK	1.1093	1.04E-02	1.82E-02	-4.1324
ENSG00000163513	TGFBR2	1.3276	1.05E-02	1.83E-02	-4.1352
ENSG00000095981	KCNK16	0.9564	1.05E-02	1.83E-02	-4.1354
ENSG00000183087	GAS6	0.7932	1.05E-02	1.83E-02	-4.1380
ENSG00000169490	TM2D2	0.8732	1.05E-02	1.83E-02	-4.1387
ENSG00000144452	ABCA12	1.0106	1.05E-02	1.83E-02	-4.1391
ENSG00000135218	CD36	0.7934	1.05E-02	1.83E-02	-4.1396

Gene ID	Gene Symbol	FC	P.Value	adj.P.Val	B
ENSG00000164002	EXO5	1.1502	1.05E-02	1.84E-02	-4.1406
ENSG00000126821	SGPP1	1.2013	1.05E-02	1.84E-02	-4.1414
ENSG00000164187	LMBRD2	0.8679	1.05E-02	1.84E-02	-4.1419
ENSG00000257727	CNPY2	0.9007	1.06E-02	1.84E-02	-4.1427
ENSG00000122641	INHBA	1.0355	1.06E-02	1.85E-02	-4.1478
ENSG00000110675	ELMOD1	0.7728	1.06E-02	1.85E-02	-4.1485
ENSG00000214575	CPEB1	1.1123	1.06E-02	1.85E-02	-4.1498
ENSG00000141295	SCRN2	0.8901	1.07E-02	1.86E-02	-4.1514
ENSG00000083307	GRHL2	1.0340	1.07E-02	1.86E-02	-4.1516
ENSG00000171385	KCND3	1.1032	1.07E-02	1.86E-02	-4.1527
ENSG00000159714	ZDHHC1	1.1628	1.07E-02	1.86E-02	-4.1538
ENSG00000164123	C4orf45	1.0554	1.07E-02	1.86E-02	-4.1552
ENSG00000197361	FBXL22	1.0744	1.07E-02	1.87E-02	-4.1571
ENSG00000167183	PRR15L	0.8707	1.07E-02	1.87E-02	-4.1573
ENSG00000196071	OR2L13	1.0245	1.08E-02	1.87E-02	-4.1590
ENSG00000146574	CCZ1B	1.0921	1.08E-02	1.87E-02	-4.1592
ENSG00000173272	MZT2A	1.1009	1.08E-02	1.87E-02	-4.1594
ENSG00000134061	CD180	0.7537	1.08E-02	1.88E-02	-4.1639
ENSG00000185697	MYBL1	1.2959	1.08E-02	1.88E-02	-4.1641
ENSG00000198851	CD3E	1.3357	1.08E-02	1.88E-02	-4.1644
ENSG00000156414	TDRD9	1.0816	1.08E-02	1.88E-02	-4.1645
ENSG00000115718	PROC	0.9185	1.09E-02	1.89E-02	-4.1671
ENSG00000183186	C2CD4C	1.0098	1.09E-02	1.89E-02	-4.1676
ENSG00000120256	LRP11	1.1911	1.09E-02	1.90E-02	-4.1717
ENSG00000198937	CCDC167	1.1892	1.09E-02	1.90E-02	-4.1723
ENSG00000105726	ATP13A1	1.1103	1.09E-02	1.90E-02	-4.1738
ENSG00000116721	PRAMEF1	1.0064	1.10E-02	1.91E-02	-4.1771
ENSG00000126460	PRRG2	0.8419	1.10E-02	1.91E-02	-4.1777
ENSG00000179639	FCER1A	0.9470	1.10E-02	1.92E-02	-4.1811
ENSG00000163104	SMARCAD1	1.1383	1.10E-02	1.92E-02	-4.1824
ENSG00000134245	WNT2B	1.0310	1.10E-02	1.92E-02	-4.1833
ENSG00000141449	GREB1L	1.0407	1.11E-02	1.92E-02	-4.1840
ENSG00000143067	ZNF697	0.8664	1.11E-02	1.92E-02	-4.1852
ENSG00000182551	ADI1	1.1118	1.11E-02	1.92E-02	-4.1855
ENSG00000136122	BORA	0.8950	1.11E-02	1.93E-02	-4.1859
ENSG00000204941	PSG5	1.0747	1.11E-02	1.93E-02	-4.1874
ENSG00000038002	AGA	1.1852	1.11E-02	1.93E-02	-4.1881
ENSG00000137692	DCUN1D5	0.8982	1.11E-02	1.94E-02	-4.1913
ENSG00000162493	PDPN	1.0292	1.12E-02	1.94E-02	-4.1918
ENSG00000186226	LCE1E	1.0251	1.12E-02	1.94E-02	-4.1918
ENSG00000189108	IL1RAPL2	1.0251	1.12E-02	1.94E-02	-4.1928
ENSG00000100116	GCAT	0.8028	1.12E-02	1.94E-02	-4.1942
ENSG00000178235	SLITRK1	1.0404	1.12E-02	1.94E-02	-4.1947
ENSG00000226174	TEX22	0.8799	1.12E-02	1.94E-02	-4.1950
ENSG00000163110	PDLIM5	1.1597	1.12E-02	1.94E-02	-4.1955
ENSG00000198055	GRK6	0.8746	1.12E-02	1.95E-02	-4.1968
ENSG00000061492	WNT8A	1.0133	1.12E-02	1.95E-02	-4.1971
ENSG00000185133	INPP5J	0.8534	1.12E-02	1.95E-02	-4.1972
ENSG00000186160	CYP4Z1	1.0099	1.12E-02	1.95E-02	-4.1975
ENSG00000165271	NOL6	0.8930	1.12E-02	1.95E-02	-4.1983
ENSG00000224712	NPIPA3	1.1521	1.12E-02	1.95E-02	-4.1992
ENSG00000263201	DPEP2NB	1.0095	1.13E-02	1.95E-02	-4.2004
ENSG00000005022	SLC25A5	1.1041	1.13E-02	1.96E-02	-4.2012
ENSG00000136488	CSH1	1.0066	1.13E-02	1.96E-02	-4.2047

Gene ID	Gene Symbol	FC	P.Value	adj.P.Val	B
ENSG00000088882	CPXM1	1.1393	1.14E-02	1.97E-02	-4.2073
ENSG00000120509	PDZD11	1.1026	1.14E-02	1.97E-02	-4.2088
ENSG00000121905	HPCA	1.0534	1.14E-02	1.97E-02	-4.2092
ENSG00000171540	OTP	1.0178	1.14E-02	1.97E-02	-4.2092
ENSG00000196139	AKR1C3	1.2026	1.14E-02	1.97E-02	-4.2094
ENSG00000196937	FAM3C	1.1410	1.14E-02	1.97E-02	-4.2102
ENSG00000100003	SEC14L2	1.0885	1.14E-02	1.98E-02	-4.2116
ENSG00000268975	MIA-RAB4B	0.9896	1.14E-02	1.98E-02	-4.2118
ENSG00000162782	TDRD5	1.0615	1.14E-02	1.98E-02	-4.2143
ENSG00000204544	MUC21	1.0253	1.14E-02	1.98E-02	-4.2144
ENSG00000174307	PHLDA3	1.2422	1.15E-02	1.98E-02	-4.2152
ENSG00000271723	MROH7-TTC4	1.0242	1.15E-02	1.99E-02	-4.2159
ENSG00000177272	KCNA3	0.8143	1.15E-02	1.99E-02	-4.2161
ENSG00000120129	DUSP1	0.7754	1.15E-02	1.99E-02	-4.2174
ENSG00000134709	HOOK1	1.3555	1.15E-02	1.99E-02	-4.2177
ENSG00000152778	IFIT5	1.1632	1.15E-02	1.99E-02	-4.2181
ENSG00000172969	FRG2C	1.0183	1.15E-02	1.99E-02	-4.2180
ENSG00000184908	CLCNKB	1.0441	1.15E-02	2.00E-02	-4.2210
ENSG00000162891	IL20	1.0144	1.15E-02	2.00E-02	-4.2224
ENSG00000162373	BEND5	1.2767	1.16E-02	2.00E-02	-4.2233
ENSG00000143469	SYT14	1.0248	1.16E-02	2.00E-02	-4.2240
ENSG00000140025	EFCAB11	1.1276	1.16E-02	2.00E-02	-4.2243
ENSG00000081386	ZNF510	1.0964	1.16E-02	2.01E-02	-4.2256
ENSG00000151233	GXYLT1	1.1251	1.16E-02	2.01E-02	-4.2268
ENSG00000173578	XCR1	0.8395	1.16E-02	2.01E-02	-4.2268
ENSG00000235699	CXorf51B	1.0572	1.16E-02	2.01E-02	-4.2286
ENSG00000159307	SCUBE1	0.7738	1.16E-02	2.01E-02	-4.2287
ENSG00000107984	DKK1	0.6722	1.16E-02	2.01E-02	-4.2291
ENSG00000155016	CYP2U1	1.1618	1.17E-02	2.02E-02	-4.2307
ENSG00000175868	CALCB	0.7824	1.17E-02	2.02E-02	-4.2307
ENSG00000133800	LYVE1	0.8311	1.17E-02	2.02E-02	-4.2310
ENSG00000153495	TEX29	0.9366	1.17E-02	2.02E-02	-4.2314
ENSG00000130477	UNC13A	1.0540	1.17E-02	2.02E-02	-4.2329
ENSG00000105991	HOXA1	0.9235	1.17E-02	2.03E-02	-4.2352
ENSG00000143545	RAB13	0.8259	1.17E-02	2.03E-02	-4.2354
ENSG00000131196	NFATC1	1.1935	1.17E-02	2.03E-02	-4.2367
ENSG00000244115	DNAJC25-GNG10	0.8989	1.18E-02	2.04E-02	-4.2402
ENSG00000204970	PCDHA1	1.0174	1.18E-02	2.04E-02	-4.2406
ENSG00000158517	NCF1	0.8809	1.18E-02	2.04E-02	-4.2423
ENSG00000141456	PELP1	1.1406	1.18E-02	2.04E-02	-4.2439
ENSG00000124743	KLHL31	0.9690	1.18E-02	2.05E-02	-4.2445
ENSG00000130307	USHBP1	1.0897	1.18E-02	2.05E-02	-4.2449
ENSG00000114166	KAT2B	1.1285	1.18E-02	2.05E-02	-4.2451
ENSG00000169217	CD2BP2	1.0930	1.19E-02	2.05E-02	-4.2454
ENSG00000151502	VPS26B	1.1036	1.19E-02	2.05E-02	-4.2462
ENSG00000151640	DPYSL4	0.9197	1.19E-02	2.05E-02	-4.2467
ENSG00000165816	VWA2	1.0151	1.19E-02	2.05E-02	-4.2467
ENSG00000167703	SLC43A2	1.1751	1.19E-02	2.05E-02	-4.2493
ENSG00000102239	BRS3	1.0063	1.20E-02	2.07E-02	-4.2545
ENSG00000132471	WBP2	1.0984	1.20E-02	2.07E-02	-4.2545
ENSG00000152977	ZIC1	1.0911	1.20E-02	2.07E-02	-4.2555
ENSG00000167716	WDR81	1.1306	1.20E-02	2.07E-02	-4.2560
ENSG00000198930	CSAG1	1.2649	1.20E-02	2.08E-02	-4.2591
ENSG00000179148	ALOXE3	1.0265	1.21E-02	2.08E-02	-4.2608

Gene ID	Gene Symbol	FC	P.Value	adj.P.Val	B
ENSG00000102181	CD99L2	1.1461	1.21E-02	2.08E-02	-4.2611
ENSG00000182256	GABRG3	1.0745	1.21E-02	2.08E-02	-4.2619
ENSG00000156990	RPUSD3	0.8845	1.21E-02	2.08E-02	-4.2629
ENSG00000178726	THBD	1.1333	1.21E-02	2.09E-02	-4.2632
ENSG00000197746	PSAP	1.1382	1.21E-02	2.09E-02	-4.2636
ENSG00000154143	PANX3	1.0133	1.21E-02	2.09E-02	-4.2663
ENSG00000125144	MT1G	1.2773	1.22E-02	2.10E-02	-4.2677
ENSG00000124780	KCNK17	1.0775	1.22E-02	2.10E-02	-4.2679
ENSG00000183077	AFMID	0.8577	1.22E-02	2.10E-02	-4.2687
ENSG00000135436	FAM186B	1.0709	1.22E-02	2.10E-02	-4.2690
ENSG00000160181	TFF2	1.1561	1.22E-02	2.10E-02	-4.2691
ENSG00000124232	RBPJL	1.0149	1.22E-02	2.10E-02	-4.2697
ENSG00000180155	LYNX1	1.1221	1.22E-02	2.11E-02	-4.2723
ENSG00000110696	C11orf58	1.0802	1.22E-02	2.11E-02	-4.2731
ENSG00000121064	SCPEP1	1.1869	1.22E-02	2.11E-02	-4.2732
ENSG00000167565	SERTAD3	1.1469	1.22E-02	2.11E-02	-4.2740
ENSG00000244045	TMEM199	1.1009	1.22E-02	2.11E-02	-4.2745
ENSG00000197870	PRB3	1.0560	1.23E-02	2.11E-02	-4.2751
ENSG00000258818	RNASE4	1.2455	1.23E-02	2.11E-02	-4.2754
ENSG00000175463	TBC1D10C	1.2373	1.23E-02	2.12E-02	-4.2771
ENSG00000179044	EXOC3L1	1.0474	1.23E-02	2.12E-02	-4.2780
ENSG00000100311	PDGFB	1.0816	1.24E-02	2.13E-02	-4.2823
ENSG00000103363	ELOB	1.0875	1.24E-02	2.13E-02	-4.2824
ENSG00000197372	ZNF675	1.2029	1.24E-02	2.13E-02	-4.2838
ENSG00000164144	ARFIP1	1.0913	1.24E-02	2.13E-02	-4.2841
ENSG00000188089	PLA2G4E	1.0180	1.24E-02	2.13E-02	-4.2844
ENSG00000269897	COMMD3-BMI1	0.8397	1.24E-02	2.14E-02	-4.2862
ENSG00000152760	DYNLT5	0.9014	1.24E-02	2.14E-02	-4.2863
ENSG00000206557	TRIM71	1.0317	1.24E-02	2.14E-02	-4.2872
ENSG00000168333	PPDPFL	1.0078	1.24E-02	2.14E-02	-4.2873
ENSG00000176566	DCAF4L2	1.1078	1.24E-02	2.14E-02	-4.2881
ENSG00000164116	GUCY1A1	1.2690	1.24E-02	2.14E-02	-4.2885
ENSG00000198488	B3GNT6	1.0306	1.24E-02	2.14E-02	-4.2887
ENSG00000196878	LAMB3	1.1893	1.25E-02	2.14E-02	-4.2894
ENSG00000241149	ZNF722P	1.0525	1.25E-02	2.14E-02	-4.2893
ENSG00000054148	PHPT1	0.8962	1.25E-02	2.14E-02	-4.2896
ENSG00000242110	AMACR	0.8319	1.25E-02	2.15E-02	-4.2912
ENSG00000136999	CCN3	1.0575	1.25E-02	2.15E-02	-4.2921
ENSG00000141622	RNF165	1.1930	1.25E-02	2.16E-02	-4.2952
ENSG00000160113	NR2F6	0.8782	1.25E-02	2.16E-02	-4.2955
ENSG00000162396	PARS2	0.8965	1.25E-02	2.16E-02	-4.2956
ENSG00000198398	TMEM207	1.0122	1.26E-02	2.16E-02	-4.2979
ENSG00000120942	UBIAD1	0.8876	1.26E-02	2.16E-02	-4.2982
ENSG00000165886	UBTD1	1.1328	1.26E-02	2.16E-02	-4.2981
ENSG00000170893	TRH	1.0082	1.26E-02	2.16E-02	-4.2983
ENSG00000107099	DOCK8	0.8811	1.26E-02	2.16E-02	-4.2993
ENSG00000255150	EID3	1.2157	1.26E-02	2.17E-02	-4.3006
ENSG00000170323	FABP4	0.7827	1.26E-02	2.17E-02	-4.3009
ENSG00000144891	AGTR1	1.0260	1.26E-02	2.17E-02	-4.3014
ENSG00000185432	METTL7A	0.8408	1.26E-02	2.17E-02	-4.3025
ENSG00000141977	CIB3	1.0953	1.27E-02	2.17E-02	-4.3036
ENSG00000144026	ZNF514	1.1586	1.27E-02	2.17E-02	-4.3043
ENSG00000172733	PURG	1.0103	1.27E-02	2.18E-02	-4.3047
ENSG00000215041	NEURL4	1.1368	1.27E-02	2.18E-02	-4.3054

Gene ID	Gene Symbol	FC	P.Value	adj.P.Val	B
ENSG00000196866	H2AC7	0.7857	1.27E-02	2.18E-02	-4.3079
ENSG00000196517	SLC6A9	0.8290	1.27E-02	2.19E-02	-4.3098
ENSG00000048540	LMO3	1.2030	1.27E-02	2.19E-02	-4.3101
ENSG00000198844	ARHGEF15	1.0371	1.27E-02	2.19E-02	-4.3102
ENSG00000010610	CD4	0.7581	1.28E-02	2.19E-02	-4.3103
ENSG00000224916	APOC4-APOC2	0.9879	1.28E-02	2.19E-02	-4.3111
ENSG00000168653	NDUFS5	0.9175	1.28E-02	2.19E-02	-4.3127
ENSG00000189339	SLC35E2B	0.8688	1.28E-02	2.19E-02	-4.3127
ENSG00000205784	ARRDC5	0.9294	1.28E-02	2.20E-02	-4.3138
ENSG00000131044	TTLL9	1.0476	1.28E-02	2.20E-02	-4.3150
ENSG00000055147	FAM114A2	0.8982	1.28E-02	2.20E-02	-4.3155
ENSG00000197658	SLC22A24	1.0107	1.29E-02	2.21E-02	-4.3178
ENSG00000256892	MTRNR2L7	1.0105	1.29E-02	2.21E-02	-4.3183
ENSG00000151353	TMEM18	0.9150	1.29E-02	2.21E-02	-4.3185
ENSG00000070985	TRPM5	1.0771	1.29E-02	2.21E-02	-4.3188
ENSG00000154822	PLCL2	1.1378	1.29E-02	2.21E-02	-4.3188
ENSG00000165572	KBTBD6	0.9194	1.29E-02	2.21E-02	-4.3195
ENSG00000007541	PIGQ	1.1102	1.29E-02	2.21E-02	-4.3197
ENSG00000100077	GRK3	1.1031	1.29E-02	2.21E-02	-4.3219
ENSG00000125637	PSD4	1.1259	1.29E-02	2.22E-02	-4.3226
ENSG00000154127	UBASH3B	1.1595	1.29E-02	2.22E-02	-4.3229
ENSG00000205439	KRTAP12-3	1.0383	1.29E-02	2.22E-02	-4.3238
ENSG00000113597	TRAPPC13	1.0868	1.30E-02	2.22E-02	-4.3258
ENSG00000114541	FRMD4B	1.1932	1.30E-02	2.23E-02	-4.3301
ENSG00000074410	CA12	1.0219	1.30E-02	2.24E-02	-4.3307
ENSG00000164117	FBXO8	0.8957	1.31E-02	2.24E-02	-4.3309
ENSG00000185104	FAF1	1.1264	1.31E-02	2.24E-02	-4.3313
ENSG00000196209	SIRPB2	0.8887	1.31E-02	2.24E-02	-4.3324
ENSG00000042832	TG	1.1722	1.31E-02	2.24E-02	-4.3339
ENSG00000145642	SHISAL2B	1.0332	1.31E-02	2.24E-02	-4.3340
ENSG00000136026	CKAP4	0.8695	1.31E-02	2.25E-02	-4.3360
ENSG00000176058	TPRN	0.8980	1.31E-02	2.25E-02	-4.3363
ENSG00000182674	KCNB2	1.0442	1.31E-02	2.25E-02	-4.3362
ENSG00000168237	GLYCTK	0.8913	1.31E-02	2.25E-02	-4.3365
ENSG00000169169	CPT1C	1.1956	1.31E-02	2.25E-02	-4.3371
ENSG00000151748	SAV1	1.1178	1.32E-02	2.25E-02	-4.3383
ENSG00000255393	OOSP4B	1.0081	1.32E-02	2.25E-02	-4.3392
ENSG00000085491	SLC25A24	1.1301	1.32E-02	2.26E-02	-4.3412
ENSG00000064886	CHI3L2	1.1272	1.32E-02	2.26E-02	-4.3413
ENSG00000114270	COL7A1	0.8616	1.32E-02	2.26E-02	-4.3421
ENSG00000173302	GPR148	1.0043	1.32E-02	2.26E-02	-4.3421
ENSG00000112115	IL17A	1.0158	1.32E-02	2.26E-02	-4.3425
ENSG00000116717	GADD45A	1.2993	1.33E-02	2.27E-02	-4.3448
ENSG00000180592	SKIDA1	0.9418	1.33E-02	2.27E-02	-4.3449
ENSG00000143595	AQP10	1.0362	1.33E-02	2.27E-02	-4.3467
ENSG00000156931	VPS8	1.1126	1.33E-02	2.27E-02	-4.3478
ENSG00000175287	PHYHD1	1.3682	1.33E-02	2.28E-02	-4.3484
ENSG00000184478	OR56A3	1.0170	1.33E-02	2.28E-02	-4.3485
ENSG00000143819	EPHX1	1.1547	1.33E-02	2.28E-02	-4.3494
ENSG00000153774	CFDP1	1.1032	1.33E-02	2.28E-02	-4.3496
ENSG00000119514	GALNT12	0.8457	1.33E-02	2.28E-02	-4.3506
ENSG00000156510	HKDC1	1.0748	1.33E-02	2.28E-02	-4.3507
ENSG00000216490	IFI30	0.7740	1.34E-02	2.28E-02	-4.3511
ENSG00000183036	PCP4	1.1264	1.34E-02	2.28E-02	-4.3525

Gene ID	Gene Symbol	FC	P.Value	adj.P.Val	B
ENSG00000175202	HIGD2B	1.0193	1.34E-02	2.29E-02	-4.3526
ENSG00000259112	NDUFC2-KCTD14	1.1468	1.34E-02	2.29E-02	-4.3528
ENSG00000188124	OR2AG2	1.0545	1.34E-02	2.29E-02	-4.3546
ENSG00000104327	CALB1	1.1026	1.34E-02	2.29E-02	-4.3547
ENSG00000172769	OR5B3	0.9944	1.34E-02	2.29E-02	-4.3561
ENSG00000105063	PPP6R1	1.0998	1.34E-02	2.29E-02	-4.3562
ENSG00000165752	STK32C	1.1308	1.34E-02	2.30E-02	-4.3570
ENSG00000129646	QRICH2	0.8910	1.34E-02	2.30E-02	-4.3571
ENSG00000168487	BMP1	1.1045	1.35E-02	2.30E-02	-4.3581
ENSG00000130005	GAMT	0.8369	1.35E-02	2.30E-02	-4.3591
ENSG00000187258	NPSR1	1.0253	1.35E-02	2.30E-02	-4.3592
ENSG00000215148	PRSS41	1.0054	1.35E-02	2.30E-02	-4.3596
ENSG00000109084	TMEM97	1.1654	1.35E-02	2.30E-02	-4.3607
ENSG00000172461	FUT9	1.0574	1.35E-02	2.30E-02	-4.3612
ENSG00000127324	TSPAN8	1.0656	1.35E-02	2.31E-02	-4.3617
ENSG00000123388	HOXC11	1.0183	1.35E-02	2.31E-02	-4.3621
ENSG00000130382	MLLT1	0.8824	1.36E-02	2.31E-02	-4.3644
ENSG00000153574	RPIA	1.1294	1.36E-02	2.32E-02	-4.3658
ENSG00000162620	LRRIQ3	0.8782	1.36E-02	2.32E-02	-4.3668
ENSG00000182890	GLUD2	1.2545	1.36E-02	2.32E-02	-4.3671
ENSG00000095627	TDRD1	1.0368	1.36E-02	2.32E-02	-4.3675
ENSG00000146576	C7orf26	0.9046	1.36E-02	2.32E-02	-4.3676
ENSG00000008118	CAMK1G	1.0255	1.36E-02	2.32E-02	-4.3689
ENSG00000130713	EXOSC2	0.8840	1.37E-02	2.33E-02	-4.3708
ENSG00000178033	CALHM5	1.0357	1.37E-02	2.33E-02	-4.3721
ENSG00000224578	HNRNPA1P48	0.9288	1.37E-02	2.34E-02	-4.3738
ENSG00000167531	LALBA	1.0050	1.37E-02	2.34E-02	-4.3739
ENSG00000184388	PABPC1L2B	1.0267	1.37E-02	2.34E-02	-4.3749
ENSG00000121753	ADGRB2	0.8452	1.37E-02	2.34E-02	-4.3750
ENSG00000178965	ERICH3	1.0136	1.37E-02	2.34E-02	-4.3755
ENSG00000204209	DAXX	0.8863	1.37E-02	2.34E-02	-4.3768
ENSG00000197245	FAM110D	1.0178	1.38E-02	2.35E-02	-4.3781
ENSG00000198839	ZNF277	0.9128	1.38E-02	2.35E-02	-4.3787
ENSG00000178750	STX19	1.0165	1.38E-02	2.36E-02	-4.3828
ENSG00000164185	ZNF474	0.9660	1.39E-02	2.36E-02	-4.3841
ENSG00000136436	CALCOCO2	1.0876	1.39E-02	2.36E-02	-4.3843
ENSG00000120049	KCNIP2	1.2006	1.39E-02	2.36E-02	-4.3851
ENSG00000122696	SLC25A51	1.1046	1.39E-02	2.36E-02	-4.3851
ENSG00000178685	PARP10	0.8452	1.39E-02	2.36E-02	-4.3856
ENSG00000158578	ALAS2	0.8052	1.39E-02	2.37E-02	-4.3868
ENSG00000168619	ADAM18	1.0391	1.39E-02	2.37E-02	-4.3884
ENSG00000175077	RTP1	1.0054	1.40E-02	2.38E-02	-4.3906
ENSG00000140386	SCAPER	0.8850	1.40E-02	2.38E-02	-4.3919
ENSG00000129009	ISLR	0.7126	1.40E-02	2.38E-02	-4.3924
ENSG00000134240	HMGCS2	1.0141	1.40E-02	2.38E-02	-4.3939
ENSG00000134697	GNL2	1.0932	1.40E-02	2.38E-02	-4.3938
ENSG00000197054	ZNF763	0.8879	1.40E-02	2.39E-02	-4.3942
ENSG00000155115	GTF3C6	0.8822	1.40E-02	2.39E-02	-4.3950
ENSG00000173915	ATP5MK	0.9091	1.40E-02	2.39E-02	-4.3961
ENSG00000197415	VEPH1	0.8376	1.41E-02	2.39E-02	-4.3971
ENSG00000115041	KCNIP3	1.0634	1.41E-02	2.39E-02	-4.3972
ENSG00000213366	GSTM2	1.2342	1.41E-02	2.39E-02	-4.3973
ENSG00000120693	SMAD9	1.0291	1.41E-02	2.40E-02	-4.3989
ENSG00000159674	SPON2	1.2321	1.41E-02	2.40E-02	-4.3989

Gene ID	Gene Symbol	FC	P.Value	adj.P.Val	B
ENSG00000071246	VASH1	1.0716	1.41E-02	2.40E-02	-4.3998
ENSG00000164822	DEFA6	0.9284	1.41E-02	2.40E-02	-4.4005
ENSG00000206503	HLA-A	1.1679	1.41E-02	2.40E-02	-4.4020
ENSG00000134551	PRH2	1.0433	1.42E-02	2.41E-02	-4.4038
ENSG00000203859	HSD3B2	0.9514	1.42E-02	2.41E-02	-4.4038
ENSG00000253305	PCDHGB6	0.8508	1.42E-02	2.41E-02	-4.4043
ENSG00000137070	IL11RA	0.8703	1.42E-02	2.41E-02	-4.4051
ENSG00000168484	SFTPC	1.0341	1.42E-02	2.41E-02	-4.4059
ENSG00000110218	PANX1	1.1047	1.42E-02	2.42E-02	-4.4064
ENSG00000161594	KLHL10	1.0329	1.42E-02	2.42E-02	-4.4085
ENSG00000111231	GPN3	1.1090	1.43E-02	2.42E-02	-4.4092
ENSG00000164414	SLC35A1	0.8861	1.43E-02	2.43E-02	-4.4105
ENSG00000147162	OGT	1.1675	1.43E-02	2.43E-02	-4.4111
ENSG00000164919	COX6C	1.0899	1.43E-02	2.43E-02	-4.4136
ENSG00000196268	ZNF493	1.1738	1.43E-02	2.43E-02	-4.4138
ENSG00000064961	HMG20B	1.0840	1.43E-02	2.44E-02	-4.4147
ENSG00000108465	CDK5RAP3	1.1237	1.44E-02	2.44E-02	-4.4153
ENSG00000184185	KCNJ12	1.0245	1.44E-02	2.44E-02	-4.4153
ENSG00000226784	PGAM4	1.0668	1.44E-02	2.44E-02	-4.4164
ENSG00000070778	PTPN21	1.0374	1.44E-02	2.44E-02	-4.4170
ENSG00000105438	KDELR1	0.9093	1.44E-02	2.45E-02	-4.4186
ENSG00000072201	LNX1	1.0720	1.44E-02	2.45E-02	-4.4190
ENSG00000162344	FGF19	1.0096	1.45E-02	2.45E-02	-4.4211
ENSG00000166263	STXBP4	1.0940	1.45E-02	2.45E-02	-4.4221
ENSG00000172465	TCEAL1	1.1257	1.45E-02	2.46E-02	-4.4226
ENSG00000133731	IMPA1	1.1084	1.45E-02	2.46E-02	-4.4230
ENSG00000173406	DAB1	0.9460	1.45E-02	2.46E-02	-4.4253
ENSG00000159200	RCAN1	0.8793	1.45E-02	2.46E-02	-4.4255
ENSG00000233041	PHGR1	0.9345	1.45E-02	2.46E-02	-4.4261
ENSG00000175906	ARL4D	1.0711	1.46E-02	2.47E-02	-4.4273
ENSG00000227057	WDR46	0.9027	1.46E-02	2.47E-02	-4.4281
ENSG00000196074	SYCP2	1.1766	1.46E-02	2.47E-02	-4.4283
ENSG00000151834	GABRA2	1.0540	1.46E-02	2.47E-02	-4.4291
ENSG00000198586	TLK1	1.1114	1.46E-02	2.48E-02	-4.4319
ENSG00000197822	OCLN	0.8400	1.46E-02	2.48E-02	-4.4329
ENSG00000170175	CHRNB1	1.1195	1.47E-02	2.49E-02	-4.4342
ENSG00000059377	TBXAS1	0.7798	1.47E-02	2.49E-02	-4.4344
ENSG00000104899	AMH	1.2379	1.47E-02	2.49E-02	-4.4347
ENSG00000129084	PSMA1	1.0921	1.47E-02	2.49E-02	-4.4356
ENSG00000185739	SRL	1.0230	1.47E-02	2.49E-02	-4.4363
ENSG00000161654	LSM12	1.0717	1.47E-02	2.50E-02	-4.4384
ENSG00000189326	SPANXN4	0.9891	1.47E-02	2.50E-02	-4.4388
ENSG00000178404	CEP295NL	1.1801	1.48E-02	2.50E-02	-4.4396
ENSG00000075618	FSCN1	0.8462	1.48E-02	2.50E-02	-4.4399
ENSG00000212122	TSSK1B	0.9935	1.48E-02	2.51E-02	-4.4426
ENSG00000122176	FMOD	1.1321	1.48E-02	2.51E-02	-4.4435
ENSG00000104957	YJU2B	1.1106	1.48E-02	2.51E-02	-4.4441
ENSG00000102313	ITIH6	1.0122	1.48E-02	2.51E-02	-4.4449
ENSG00000161133	USP41	0.8615	1.49E-02	2.51E-02	-4.4454
ENSG00000154016	GRAP	0.8487	1.49E-02	2.52E-02	-4.4455
ENSG00000105519	CAPS	0.8826	1.49E-02	2.52E-02	-4.4463
ENSG00000171551	ECEL1	1.0141	1.49E-02	2.53E-02	-4.4505
ENSG00000110848	CD69	1.4642	1.49E-02	2.53E-02	-4.4506
ENSG00000251258	RFPL4B	1.1485	1.50E-02	2.53E-02	-4.4511

Gene ID	Gene Symbol	FC	P.Value	adj.P.Val	B
ENSG00000168994	PXDC1	1.2382	1.50E-02	2.53E-02	-4.4514
ENSG00000130812	ANGPTL6	0.8874	1.50E-02	2.53E-02	-4.4522
ENSG00000162191	UBXN1	1.1019	1.50E-02	2.54E-02	-4.4531
ENSG00000180934	OR56A1	1.0070	1.50E-02	2.54E-02	-4.4542
ENSG00000248144	ADH1C	1.0199	1.50E-02	2.55E-02	-4.4567
ENSG00000070388	FGF22	0.9206	1.51E-02	2.55E-02	-4.4573
ENSG00000095539	SEMA4G	1.1273	1.51E-02	2.55E-02	-4.4578
ENSG00000142494	SLC47A1	0.7367	1.51E-02	2.55E-02	-4.4591
ENSG00000158077	NLRP14	1.1249	1.51E-02	2.56E-02	-4.4604
ENSG00000213297	ZNF625-ZNF20	0.9244	1.51E-02	2.56E-02	-4.4607
ENSG00000178252	WDR6	1.1208	1.51E-02	2.56E-02	-4.4619
ENSG00000164749	HNF4G	1.1696	1.51E-02	2.56E-02	-4.4625
ENSG00000106511	MEOX2	1.0071	1.52E-02	2.56E-02	-4.4639
ENSG00000101349	PAK5	1.0215	1.52E-02	2.57E-02	-4.4650
ENSG00000183434	TFDP3	1.0565	1.52E-02	2.57E-02	-4.4655
ENSG00000115112	TFCP2L1	1.0253	1.52E-02	2.57E-02	-4.4665
ENSG00000159905	ZNF221	1.0597	1.53E-02	2.58E-02	-4.4689
ENSG00000253159	PCDHGA12	0.8954	1.53E-02	2.58E-02	-4.4700
ENSG00000132182	NUP210	1.1569	1.53E-02	2.58E-02	-4.4707
ENSG00000101000	PROCR	0.9229	1.53E-02	2.58E-02	-4.4713
ENSG00000100714	MTHFD1	1.1403	1.53E-02	2.59E-02	-4.4729
ENSG00000119681	LTBP2	1.0877	1.53E-02	2.59E-02	-4.4737
ENSG00000136068	FLNB	1.2526	1.53E-02	2.59E-02	-4.4738
ENSG00000186575	NF2	1.1065	1.53E-02	2.59E-02	-4.4740
ENSG00000172476	RAB40A	1.0687	1.54E-02	2.59E-02	-4.4750
ENSG00000136828	RALGPS1	0.8679	1.54E-02	2.59E-02	-4.4751
ENSG00000174453	VWC2L	1.0145	1.54E-02	2.60E-02	-4.4758
ENSG00000173928	SWSAP1	0.9219	1.54E-02	2.60E-02	-4.4779
ENSG00000185038	MROH2A	0.7832	1.55E-02	2.61E-02	-4.4798
ENSG00000034510	TMSB10	1.1221	1.55E-02	2.61E-02	-4.4820
ENSG00000125975	C20orf173	1.0096	1.55E-02	2.61E-02	-4.4823
ENSG00000105479	ODAD1	1.1233	1.55E-02	2.61E-02	-4.4824
ENSG00000147804	SLC39A4	1.1936	1.55E-02	2.62E-02	-4.4831
ENSG00000144199	FAHD2B	1.1181	1.55E-02	2.62E-02	-4.4835
ENSG00000214872	SMTNL1	1.0988	1.55E-02	2.62E-02	-4.4834
ENSG00000100626	GALNT16	0.9623	1.56E-02	2.63E-02	-4.4876
ENSG00000102760	RGCC	1.3152	1.57E-02	2.64E-02	-4.4919
ENSG00000150783	TEX12	1.0752	1.57E-02	2.65E-02	-4.4937
ENSG00000113758	DBN1	1.2657	1.58E-02	2.66E-02	-4.4976
ENSG00000040933	INPP4A	1.1523	1.58E-02	2.66E-02	-4.4981
ENSG00000247595	SPTY2D1OS	0.9124	1.58E-02	2.66E-02	-4.4998
ENSG00000144681	STAC	1.0446	1.58E-02	2.66E-02	-4.5001
ENSG00000151743	AMN1	1.0902	1.58E-02	2.67E-02	-4.5004
ENSG00000187918	OR51I2	1.0090	1.58E-02	2.67E-02	-4.5008
ENSG00000066032	CTNNA2	0.7655	1.58E-02	2.67E-02	-4.5015
ENSG00000152266	PTH	1.0077	1.59E-02	2.67E-02	-4.5033
ENSG00000176946	THAP4	1.0803	1.59E-02	2.69E-02	-4.5075
ENSG00000064102	INTS13	1.1140	1.60E-02	2.69E-02	-4.5087
ENSG00000069329	VPS35	1.1371	1.60E-02	2.70E-02	-4.5105
ENSG00000167291	TBC1D16	1.1698	1.60E-02	2.70E-02	-4.5116
ENSG00000179542	SLITRK4	1.0914	1.60E-02	2.70E-02	-4.5117
ENSG00000181588	MEX3D	1.1189	1.60E-02	2.70E-02	-4.5124
ENSG00000183783	KCTD8	1.0530	1.60E-02	2.70E-02	-4.5126
ENSG00000197302	ZNF720	1.0922	1.60E-02	2.70E-02	-4.5127

Gene ID	Gene Symbol	FC	P.Value	adj.P.Val	B
ENSG00000169271	HSPB3	1.0089	1.60E-02	2.70E-02	-4.5128
ENSG00000120280	TASL	1.2108	1.61E-02	2.71E-02	-4.5176
ENSG00000198788	MUC2	1.0041	1.61E-02	2.71E-02	-4.5176
ENSG00000135374	ELF5	0.9622	1.62E-02	2.72E-02	-4.5193
ENSG00000143333	RGS16	1.2854	1.62E-02	2.72E-02	-4.5199
ENSG00000169926	KLF13	0.8859	1.62E-02	2.72E-02	-4.5211
ENSG00000164325	TMEM174	1.0046	1.62E-02	2.73E-02	-4.5216
ENSG00000185053	SGCZ	1.0203	1.62E-02	2.73E-02	-4.5223
ENSG00000084674	APOB	0.9747	1.62E-02	2.73E-02	-4.5225
ENSG00000213903	LTB4R	1.1346	1.63E-02	2.74E-02	-4.5253
ENSG00000188290	HES4	1.1070	1.63E-02	2.74E-02	-4.5254
ENSG00000113749	HRH2	0.8912	1.63E-02	2.75E-02	-4.5290
ENSG00000134760	DSG1	1.0364	1.64E-02	2.75E-02	-4.5299
ENSG00000162139	NEU3	1.1649	1.64E-02	2.76E-02	-4.5315
ENSG00000198075	SULT1C4	1.0886	1.64E-02	2.76E-02	-4.5315
ENSG00000142856	ITGB3BP	1.1443	1.64E-02	2.76E-02	-4.5321
ENSG00000173456	RNF26	1.1012	1.64E-02	2.76E-02	-4.5322
ENSG00000225950	NTF4	1.0594	1.64E-02	2.76E-02	-4.5325
ENSG00000154146	NRGN	1.2051	1.64E-02	2.76E-02	-4.5326
ENSG00000171478	SPACA5B	1.0124	1.64E-02	2.76E-02	-4.5331
ENSG00000213901	SLC23A3	1.0975	1.64E-02	2.76E-02	-4.5338
ENSG00000103021	CCDC113	1.0666	1.65E-02	2.77E-02	-4.5353
ENSG00000205076	LGALS7	1.0193	1.65E-02	2.77E-02	-4.5368
ENSG00000118257	NRP2	1.1975	1.65E-02	2.77E-02	-4.5374
ENSG00000169129	AFAP1L2	1.0853	1.65E-02	2.77E-02	-4.5379
ENSG00000242441	GTF2A1L	1.0520	1.65E-02	2.77E-02	-4.5384
ENSG00000172269	DPAGT1	0.8954	1.66E-02	2.78E-02	-4.5413
ENSG00000204740	MALRD1	1.1001	1.66E-02	2.79E-02	-4.5428
ENSG00000166049	PASD1	1.0796	1.66E-02	2.79E-02	-4.5437
ENSG00000106603	COA1	0.9217	1.66E-02	2.80E-02	-4.5455
ENSG00000166959	MS4A8	1.0110	1.66E-02	2.80E-02	-4.5456
ENSG00000180043	FAM71E2	1.0063	1.67E-02	2.80E-02	-4.5474
ENSG00000122859	NEUROG3	1.1151	1.67E-02	2.80E-02	-4.5476
ENSG00000111667	USP5	1.1355	1.68E-02	2.81E-02	-4.5518
ENSG00000060566	CREB3L3	1.0324	1.68E-02	2.81E-02	-4.5519
ENSG00000099840	IZUMO4	1.2310	1.68E-02	2.82E-02	-4.5523
ENSG00000152822	GRM1	1.0265	1.68E-02	2.82E-02	-4.5526
ENSG00000176485	PLAAT3	0.8200	1.68E-02	2.82E-02	-4.5541
ENSG00000177875	CCDC184	1.0248	1.68E-02	2.82E-02	-4.5546
ENSG00000107537	PHYH	1.1289	1.68E-02	2.82E-02	-4.5554
ENSG00000170624	SGCD	1.0733	1.68E-02	2.82E-02	-4.5554
ENSG00000205838	TTC23L	1.0726	1.69E-02	2.83E-02	-4.5564
ENSG00000215218	UBE2QL1	0.8067	1.69E-02	2.83E-02	-4.5579
ENSG00000257987	TEX49	1.0491	1.69E-02	2.83E-02	-4.5583
ENSG00000162460	TMEM82	1.0056	1.69E-02	2.83E-02	-4.5587
ENSG00000243130	PSG11	1.0260	1.69E-02	2.83E-02	-4.5590
ENSG00000106078	COBL	1.1986	1.69E-02	2.84E-02	-4.5593
ENSG00000125630	POLR1B	1.1282	1.69E-02	2.84E-02	-4.5594
ENSG00000186912	P2RY4	1.0271	1.69E-02	2.84E-02	-4.5594
ENSG00000132793	LPIN3	1.2361	1.69E-02	2.84E-02	-4.5607
ENSG00000089006	SNX5	1.1019	1.69E-02	2.84E-02	-4.5612
ENSG00000070061	ELP1	0.9103	1.70E-02	2.84E-02	-4.5616
ENSG00000073734	ABCB11	1.0300	1.70E-02	2.84E-02	-4.5619
ENSG00000043355	ZIC2	1.0247	1.70E-02	2.85E-02	-4.5633

Gene ID	Gene Symbol	FC	P.Value	adj.P.Val	B
ENSG00000170390	DCLK2	1.0843	1.70E-02	2.85E-02	-4.5654
ENSG00000106123	EPHB6	1.0867	1.71E-02	2.86E-02	-4.5675
ENSG00000222036	POTEM	1.0932	1.71E-02	2.86E-02	-4.5676
ENSG00000174306	ZHX3	1.1207	1.71E-02	2.86E-02	-4.5678
ENSG00000166548	TK2	0.8746	1.71E-02	2.86E-02	-4.5686
ENSG00000163512	AZI2	1.0862	1.71E-02	2.87E-02	-4.5698
ENSG00000008394	MGST1	1.3052	1.71E-02	2.87E-02	-4.5708
ENSG00000054938	CHRDL2	0.8891	1.71E-02	2.87E-02	-4.5712
ENSG00000165923	AGBL2	1.1116	1.71E-02	2.87E-02	-4.5712
ENSG00000105261	OVOL3	1.1307	1.72E-02	2.87E-02	-4.5723
ENSG00000132932	ATP8A2	0.8224	1.72E-02	2.88E-02	-4.5735
ENSG00000198598	MMP17	1.0912	1.72E-02	2.88E-02	-4.5740
ENSG00000135040	NAA35	1.0808	1.72E-02	2.88E-02	-4.5746
ENSG00000143028	SYPL2	1.0316	1.73E-02	2.89E-02	-4.5771
ENSG00000171054	OR13H1	1.0134	1.73E-02	2.89E-02	-4.5786
ENSG00000198881	ASB12	1.0357	1.73E-02	2.89E-02	-4.5786
ENSG00000135973	GPR45	0.9817	1.73E-02	2.89E-02	-4.5790
ENSG00000165626	BEND7	1.2041	1.73E-02	2.89E-02	-4.5791
ENSG00000104660	LEPROTL1	1.0827	1.73E-02	2.90E-02	-4.5795
ENSG00000164946	FREM1	1.0307	1.74E-02	2.90E-02	-4.5819
ENSG00000052802	MSMO1	0.8565	1.74E-02	2.91E-02	-4.5828
ENSG00000196860	TOMM20L	1.0639	1.74E-02	2.91E-02	-4.5831
ENSG00000047936	ROS1	0.9940	1.74E-02	2.91E-02	-4.5855
ENSG00000164530	PI16	0.9682	1.74E-02	2.92E-02	-4.5863
ENSG00000155827	RNF20	0.8806	1.75E-02	2.92E-02	-4.5868
ENSG00000177854	TMEM187	0.8726	1.74E-02	2.92E-02	-4.5867
ENSG00000100095	SEZ6L	1.0389	1.75E-02	2.92E-02	-4.5878
ENSG00000141759	TXNL4A	1.0788	1.75E-02	2.92E-02	-4.5889
ENSG00000165416	SUGT1	0.9248	1.75E-02	2.93E-02	-4.5897
ENSG00000158639	PAGE5	1.4026	1.75E-02	2.93E-02	-4.5903
ENSG00000231989	PPP1R2B	1.0508	1.76E-02	2.93E-02	-4.5919
ENSG00000147471	PLPBP	0.8919	1.76E-02	2.93E-02	-4.5922
ENSG00000168781	PPIP5K1	0.8567	1.76E-02	2.93E-02	-4.5922
ENSG00000082641	NFE2L1	1.1803	1.76E-02	2.93E-02	-4.5924
ENSG00000007952	NOX1	1.0580	1.76E-02	2.94E-02	-4.5946
ENSG00000204450	TRIM64	0.9947	1.76E-02	2.95E-02	-4.5965
ENSG00000003147	ICA1	0.7899	1.77E-02	2.96E-02	-4.5991
ENSG00000152669	CCNO	0.8917	1.77E-02	2.96E-02	-4.5995
ENSG00000148110	MFSD14B	0.8973	1.77E-02	2.96E-02	-4.5996
ENSG00000120729	MYOT	0.8938	1.77E-02	2.96E-02	-4.6002
ENSG00000150051	MKX	0.7693	1.77E-02	2.96E-02	-4.6005
ENSG00000140718	FTO	1.1307	1.77E-02	2.96E-02	-4.6014
ENSG00000172081	MOB3A	1.2534	1.77E-02	2.96E-02	-4.6014
ENSG00000187546	AGMO	1.1800	1.78E-02	2.96E-02	-4.6020
ENSG00000177494	ZBED2	1.0041	1.78E-02	2.97E-02	-4.6028
ENSG00000196943	NOP9	1.1006	1.78E-02	2.97E-02	-4.6034
ENSG00000127554	GFER	1.0832	1.78E-02	2.97E-02	-4.6036
ENSG00000189180	ZNF33A	1.0989	1.78E-02	2.97E-02	-4.6036
ENSG00000148795	CYP17A1	1.0830	1.78E-02	2.97E-02	-4.6041
ENSG00000163956	LRPAP1	1.1155	1.78E-02	2.97E-02	-4.6042
ENSG00000143443	C1orf56	1.1398	1.78E-02	2.97E-02	-4.6053
ENSG00000175066	GK5	0.8931	1.78E-02	2.97E-02	-4.6055
ENSG00000054598	FOXC1	1.0883	1.78E-02	2.97E-02	-4.6056
ENSG00000171033	PKIA	1.1619	1.78E-02	2.98E-02	-4.6062

Gene ID	Gene Symbol	FC	P.Value	adj.P.Val	B
ENSG00000176165	FOXG1	1.0390	1.78E-02	2.98E-02	-4.6063
ENSG00000162949	CAPN13	1.0465	1.79E-02	2.98E-02	-4.6068
ENSG00000168453	HR	1.0335	1.79E-02	2.98E-02	-4.6068
ENSG00000115694	STK25	1.1133	1.79E-02	2.98E-02	-4.6081
ENSG00000167476	JSRP1	1.4502	1.79E-02	2.98E-02	-4.6081
ENSG00000167207	NOD2	1.2121	1.79E-02	2.98E-02	-4.6086
ENSG00000114353	GNAI2	1.0848	1.79E-02	2.98E-02	-4.6089
ENSG00000137185	ZSCAN9	1.0959	1.79E-02	2.98E-02	-4.6089
ENSG00000108849	PPY	1.0168	1.79E-02	2.99E-02	-4.6107
ENSG00000154856	APCDD1	1.0441	1.79E-02	2.99E-02	-4.6106
ENSG00000198092	TMPRSS11F	1.0699	1.80E-02	2.99E-02	-4.6123
ENSG00000172519	OR10H5	1.0118	1.80E-02	3.00E-02	-4.6143
ENSG00000176040	TMPRSS7	1.0108	1.80E-02	3.00E-02	-4.6152
ENSG00000105171	POP4	1.0733	1.80E-02	3.00E-02	-4.6154
ENSG00000215269	GAGE12G	1.2678	1.80E-02	3.01E-02	-4.6162
ENSG00000236362	GAGE12F	1.2678	1.80E-02	3.01E-02	-4.6162
ENSG00000134291	TMEM106C	1.1476	1.81E-02	3.02E-02	-4.6194
ENSG00000114859	CLCN2	1.1095	1.81E-02	3.02E-02	-4.6201
ENSG00000141068	KSR1	1.2215	1.81E-02	3.02E-02	-4.6201
ENSG00000099998	GGT5	1.1290	1.81E-02	3.02E-02	-4.6203
ENSG00000135406	PRPH	1.0124	1.81E-02	3.02E-02	-4.6208
ENSG00000215845	TSTD1	1.1155	1.82E-02	3.02E-02	-4.6219
ENSG00000222018	FAM243A	0.9098	1.82E-02	3.02E-02	-4.6224
ENSG00000112308	C6orf62	1.1113	1.82E-02	3.02E-02	-4.6225
ENSG00000167676	PLIN4	1.0900	1.82E-02	3.02E-02	-4.6225
ENSG00000186471	AKAP14	1.1036	1.82E-02	3.03E-02	-4.6234
ENSG00000187742	SECISBP2	0.9047	1.82E-02	3.03E-02	-4.6245
ENSG00000148357	HMCN2	0.7489	1.82E-02	3.03E-02	-4.6253
ENSG00000175920	DOK7	1.0498	1.82E-02	3.03E-02	-4.6254
ENSG00000187912	CLEC17A	1.0694	1.82E-02	3.03E-02	-4.6259
ENSG00000112242	E2F3	0.8935	1.83E-02	3.04E-02	-4.6268
ENSG00000187010	RHD	1.1006	1.83E-02	3.04E-02	-4.6274
ENSG00000167311	ART5	1.0103	1.83E-02	3.04E-02	-4.6284
ENSG00000070366	SMG6	1.1011	1.83E-02	3.05E-02	-4.6304
ENSG00000189152	GRAPL	0.8447	1.84E-02	3.05E-02	-4.6309
ENSG00000146067	FAM193B	1.1191	1.84E-02	3.06E-02	-4.6326
ENSG00000182208	MOB2	1.0865	1.84E-02	3.06E-02	-4.6345
ENSG00000198520	ARMH1	1.1481	1.85E-02	3.07E-02	-4.6360
ENSG00000081842	PCDHA6	1.0246	1.85E-02	3.07E-02	-4.6376
ENSG00000171467	ZNF318	1.1348	1.85E-02	3.08E-02	-4.6387
ENSG00000167536	DHRS13	1.1208	1.86E-02	3.08E-02	-4.6405
ENSG00000215217	C5orf49	1.0063	1.86E-02	3.08E-02	-4.6406
ENSG00000092295	TGM1	0.8931	1.86E-02	3.08E-02	-4.6409
ENSG00000176219	OR11H6	0.9954	1.86E-02	3.10E-02	-4.6443
ENSG00000113460	BRIX1	1.0896	1.86E-02	3.10E-02	-4.6446
ENSG00000171357	LURAP1	1.0553	1.86E-02	3.10E-02	-4.6446
ENSG00000187554	TLR5	1.0957	1.87E-02	3.10E-02	-4.6465
ENSG00000117983	MUC5B	1.0345	1.87E-02	3.10E-02	-4.6466
ENSG00000132196	HSD17B7	1.1249	1.87E-02	3.11E-02	-4.6483
ENSG00000103742	IGDCC4	0.9743	1.87E-02	3.11E-02	-4.6485
ENSG00000165507	DEPP1	1.3663	1.88E-02	3.11E-02	-4.6500
ENSG00000172315	TP53RK	0.8744	1.88E-02	3.11E-02	-4.6502
ENSG00000105011	ASF1B	1.1912	1.88E-02	3.13E-02	-4.6539
ENSG00000204560	DHX16	1.1010	1.88E-02	3.13E-02	-4.6540

Gene ID	Gene Symbol	FC	P.Value	adj.P.Val	B
ENSG00000227868	TEX46	1.0250	1.89E-02	3.13E-02	-4.6544
ENSG00000149269	PAK1	0.8790	1.89E-02	3.14E-02	-4.6573
ENSG00000205186	FABP9	1.0092	1.89E-02	3.14E-02	-4.6573
ENSG00000139055	ERP27	1.1067	1.90E-02	3.15E-02	-4.6604
ENSG00000100575	TIMM9	0.9222	1.90E-02	3.15E-02	-4.6612
ENSG00000116039	ATP6V1B1	1.0240	1.90E-02	3.16E-02	-4.6625
ENSG00000136490	LIMD2	1.1913	1.91E-02	3.16E-02	-4.6643
ENSG00000152782	PANK1	1.1094	1.91E-02	3.16E-02	-4.6650
ENSG00000124749	COL21A1	1.0590	1.91E-02	3.17E-02	-4.6664
ENSG00000183706	OR4N4	1.0066	1.91E-02	3.17E-02	-4.6670
ENSG00000255181	CCDC166	1.0095	1.91E-02	3.17E-02	-4.6673
ENSG00000140939	NOL3	0.8679	1.92E-02	3.18E-02	-4.6690
ENSG00000145476	CYP4V2	1.1709	1.92E-02	3.18E-02	-4.6697
ENSG00000139168	ZCRB1	1.0768	1.92E-02	3.19E-02	-4.6714
ENSG00000144214	LYG1	0.9064	1.93E-02	3.19E-02	-4.6733
ENSG00000251201	TMED7-TICAM2	0.9040	1.93E-02	3.19E-02	-4.6739
ENSG00000130545	CRB3	0.7984	1.93E-02	3.19E-02	-4.6740
ENSG00000164237	CMBL	1.1951	1.93E-02	3.20E-02	-4.6752
ENSG00000187634	SAMD11	1.3954	1.93E-02	3.20E-02	-4.6758
ENSG00000160049	DFFA	1.1085	1.93E-02	3.20E-02	-4.6766
ENSG00000079462	PAFAH1B3	0.8921	1.93E-02	3.20E-02	-4.6769
ENSG00000168538	TRAPPC11	1.1019	1.94E-02	3.21E-02	-4.6790
ENSG00000183629	GOLGA8G	0.9785	1.94E-02	3.21E-02	-4.6798
ENSG00000159212	CLIC6	1.1018	1.94E-02	3.22E-02	-4.6805
ENSG00000175662	TOM1L2	1.1171	1.94E-02	3.22E-02	-4.6811
ENSG00000148341	SH3GLB2	0.9009	1.94E-02	3.22E-02	-4.6815
ENSG00000260456	C16orf95	0.8668	1.95E-02	3.22E-02	-4.6822
ENSG00000183734	ASCL2	0.9621	1.95E-02	3.22E-02	-4.6825
ENSG00000157060	SHCBP1L	1.0208	1.95E-02	3.22E-02	-4.6829
ENSG00000172113	NME6	0.8966	1.95E-02	3.23E-02	-4.6835
ENSG00000177951	BET1L	0.9164	1.95E-02	3.23E-02	-4.6847
ENSG00000115902	SLC1A4	1.1297	1.95E-02	3.23E-02	-4.6853
ENSG00000177674	AGTRAP	1.1470	1.95E-02	3.23E-02	-4.6857
ENSG00000169181	GSG1L	1.0408	1.96E-02	3.24E-02	-4.6876
ENSG00000166946	CCNDBP1	0.9353	1.96E-02	3.24E-02	-4.6884
ENSG00000115318	LOXL3	1.0870	1.96E-02	3.25E-02	-4.6896
ENSG00000123560	PLP1	1.0345	1.96E-02	3.25E-02	-4.6896
ENSG00000169851	PCDH7	1.1165	1.97E-02	3.25E-02	-4.6916
ENSG00000143147	GPR161	1.0588	1.97E-02	3.26E-02	-4.6924
ENSG00000154473	BUB3	0.8972	1.97E-02	3.26E-02	-4.6937
ENSG00000112852	PCDHB2	1.1355	1.98E-02	3.27E-02	-4.6957
ENSG00000124731	TREM1	1.2740	1.98E-02	3.27E-02	-4.6964
ENSG00000115275	MOGS	1.0957	1.98E-02	3.28E-02	-4.6988
ENSG00000187609	EXD3	0.8871	1.99E-02	3.28E-02	-4.6997
ENSG00000184307	ZDHHC23	0.8791	1.99E-02	3.29E-02	-4.7009
ENSG00000251493	FOXD1	1.0952	1.99E-02	3.29E-02	-4.7018
ENSG00000138684	IL21	1.0134	1.99E-02	3.30E-02	-4.7034
ENSG00000237651	C2orf74	0.8679	1.99E-02	3.30E-02	-4.7038
ENSG00000125816	NKX2-4	1.0031	2.00E-02	3.30E-02	-4.7045
ENSG00000123496	IL13RA2	1.0457	2.00E-02	3.30E-02	-4.7050
ENSG00000189367	KIAA0408	1.1333	2.00E-02	3.30E-02	-4.7056
ENSG00000106244	PDAP1	1.0840	2.00E-02	3.31E-02	-4.7072
ENSG00000011132	APBA3	0.9086	2.00E-02	3.31E-02	-4.7074
ENSG00000171522	PTGER4	1.2334	2.00E-02	3.31E-02	-4.7075

Gene ID	Gene Symbol	FC	P.Value	adj.P.Val	B
ENSG00000164458	TBXT	1.0041	2.01E-02	3.31E-02	-4.7091
ENSG00000189319	FAM53B	1.1583	2.01E-02	3.31E-02	-4.7092
ENSG00000145283	SLC10A6	1.0243	2.01E-02	3.32E-02	-4.7094
ENSG00000180881	CAPS2	1.1448	2.01E-02	3.32E-02	-4.7097
ENSG00000002919	SNX11	1.0834	2.01E-02	3.32E-02	-4.7104
ENSG00000128591	FLNC	1.0161	2.01E-02	3.32E-02	-4.7105
ENSG00000204347	BTBD17	1.0537	2.01E-02	3.32E-02	-4.7120
ENSG00000204228	HSD17B8	0.8860	2.01E-02	3.33E-02	-4.7124
ENSG00000118690	ARMC2	0.8326	2.02E-02	3.34E-02	-4.7158
ENSG00000091138	SLC26A3	1.0158	2.02E-02	3.34E-02	-4.7160
ENSG00000186526	CYP4F8	1.0342	2.03E-02	3.35E-02	-4.7185
ENSG00000128284	APOL3	0.7561	2.03E-02	3.35E-02	-4.7192
ENSG00000177201	OR2T12	1.0035	2.03E-02	3.35E-02	-4.7192
ENSG00000170468	RIOX1	1.0939	2.04E-02	3.36E-02	-4.7218
ENSG00000173611	SCAI	1.0937	2.04E-02	3.36E-02	-4.7224
ENSG00000168528	SERINC2	1.2444	2.04E-02	3.36E-02	-4.7230
ENSG00000095139	ARCN1	1.0915	2.04E-02	3.37E-02	-4.7235
ENSG00000157851	DPYSL5	1.0319	2.04E-02	3.37E-02	-4.7254
ENSG00000163406	SLC15A2	0.8225	2.05E-02	3.37E-02	-4.7258
ENSG00000141431	ASXL3	1.0838	2.05E-02	3.37E-02	-4.7263
ENSG00000127191	TRAF2	1.1259	2.05E-02	3.38E-02	-4.7266
ENSG00000185737	NRG3	0.7880	2.05E-02	3.38E-02	-4.7267
ENSG00000170423	KRT78	1.0250	2.05E-02	3.38E-02	-4.7269
ENSG00000197044	ZNF441	0.8780	2.05E-02	3.38E-02	-4.7287
ENSG00000147255	IGSF1	1.1451	2.05E-02	3.38E-02	-4.7288
ENSG00000113805	CNTN3	1.0427	2.05E-02	3.38E-02	-4.7290
ENSG00000168913	ENHO	1.0396	2.06E-02	3.39E-02	-4.7299
ENSG00000198483	ANKRD35	0.9781	2.06E-02	3.39E-02	-4.7300
ENSG00000163754	GYG1	0.9079	2.06E-02	3.39E-02	-4.7316
ENSG00000188042	ARL4C	1.3409	2.06E-02	3.40E-02	-4.7324
ENSG00000137364	TPMT	1.1286	2.06E-02	3.40E-02	-4.7336
ENSG00000204839	MROH6	1.0990	2.07E-02	3.40E-02	-4.7346
ENSG00000183260	ABHD16B	0.8859	2.07E-02	3.40E-02	-4.7347
ENSG00000215277	RNF212B	1.0744	2.07E-02	3.41E-02	-4.7351
ENSG00000136859	ANGPTL2	1.1146	2.07E-02	3.41E-02	-4.7354
ENSG00000126749	EMG1	1.1036	2.07E-02	3.41E-02	-4.7367
ENSG00000221909	FAM200A	0.9075	2.07E-02	3.41E-02	-4.7366
ENSG00000133895	MEN1	1.1043	2.08E-02	3.42E-02	-4.7394
ENSG00000173612	GPRC6A	1.0248	2.08E-02	3.42E-02	-4.7401
ENSG00000061455	PRDM6	0.9472	2.08E-02	3.42E-02	-4.7403
ENSG00000188816	HMX2	0.9457	2.08E-02	3.43E-02	-4.7411
ENSG00000148290	SURF1	0.9327	2.08E-02	3.43E-02	-4.7416
ENSG00000135903	PAX3	1.0281	2.09E-02	3.43E-02	-4.7427
ENSG00000133055	MYBPH	1.0263	2.09E-02	3.43E-02	-4.7432
ENSG00000162298	SYVN1	1.1150	2.09E-02	3.45E-02	-4.7462
ENSG00000163516	ANKZF1	1.1206	2.10E-02	3.45E-02	-4.7471
ENSG00000145439	CBR4	0.8783	2.10E-02	3.45E-02	-4.7484
ENSG00000019102	VSIG2	1.0724	2.10E-02	3.46E-02	-4.7498
ENSG00000090013	BLVRB	0.8570	2.10E-02	3.46E-02	-4.7500
ENSG00000121895	TMEM156	0.7260	2.10E-02	3.46E-02	-4.7503
ENSG00000163814	CDCP1	1.0412	2.10E-02	3.46E-02	-4.7506
ENSG00000173610	UGT2A1	1.1258	2.10E-02	3.46E-02	-4.7507
ENSG00000241644	INMT	0.8972	2.11E-02	3.46E-02	-4.7510
ENSG00000267368	UPK3BL1	1.1566	2.11E-02	3.46E-02	-4.7514

Gene ID	Gene Symbol	FC	P.Value	adj.P.Val	B
ENSG00000129450	SIGLEC9	0.8675	2.11E-02	3.46E-02	-4.7518
ENSG00000126522	ASL	1.1023	2.11E-02	3.47E-02	-4.7522
ENSG00000139714	MORN3	1.0651	2.11E-02	3.47E-02	-4.7524
ENSG00000088812	ATRN	1.1302	2.11E-02	3.47E-02	-4.7531
ENSG00000075234	TTC38	1.1088	2.12E-02	3.48E-02	-4.7553
ENSG00000103876	FAH	0.8948	2.12E-02	3.48E-02	-4.7557
ENSG00000137968	SLC44A5	1.2201	2.12E-02	3.49E-02	-4.7579
ENSG00000112246	SIM1	1.0081	2.12E-02	3.49E-02	-4.7581
ENSG00000081479	LRP2	1.0880	2.12E-02	3.49E-02	-4.7582
ENSG00000175322	ZNF519	1.1528	2.13E-02	3.49E-02	-4.7591
ENSG00000087116	ADAMTS2	1.0791	2.13E-02	3.50E-02	-4.7606
ENSG00000143473	KCNH1	1.0038	2.13E-02	3.50E-02	-4.7611
ENSG00000173599	PC	1.2108	2.13E-02	3.50E-02	-4.7611
ENSG00000169554	ZEB2	1.2035	2.14E-02	3.51E-02	-4.7633
ENSG00000162738	VANGL2	1.0783	2.14E-02	3.51E-02	-4.7643
ENSG00000168067	MAP4K2	1.1101	2.14E-02	3.51E-02	-4.7644
ENSG00000183291	SELENOF	1.0979	2.14E-02	3.51E-02	-4.7647
ENSG00000167778	SPRYD3	0.8948	2.14E-02	3.52E-02	-4.7657
ENSG00000114650	SCAP	1.0999	2.14E-02	3.52E-02	-4.7659
ENSG00000176490	DIRAS1	1.2314	2.15E-02	3.52E-02	-4.7673
ENSG00000177380	PPFIA3	0.8874	2.15E-02	3.52E-02	-4.7673
ENSG00000075142	SRI	1.1004	2.15E-02	3.52E-02	-4.7677
ENSG00000167461	RAB8A	0.8891	2.15E-02	3.53E-02	-4.7696
ENSG00000105738	SIPA1L3	1.1272	2.15E-02	3.53E-02	-4.7710
ENSG00000221836	OR2A5	0.9890	2.16E-02	3.54E-02	-4.7720
ENSG00000108551	RASD1	1.3226	2.16E-02	3.55E-02	-4.7741
ENSG00000181323	SPEM1	1.0028	2.16E-02	3.55E-02	-4.7751
ENSG00000137713	PPP2R1B	1.1181	2.17E-02	3.55E-02	-4.7755
ENSG00000006747	SCIN	1.1372	2.17E-02	3.55E-02	-4.7756
ENSG00000081237	PTPRC	1.3167	2.17E-02	3.56E-02	-4.7766
ENSG00000138175	ARL3	0.8903	2.17E-02	3.56E-02	-4.7769
ENSG00000036565	SLC18A1	1.0220	2.17E-02	3.56E-02	-4.7770
ENSG00000239474	KLHL41	0.9334	2.17E-02	3.56E-02	-4.7778
ENSG00000130304	SLC27A1	1.1074	2.17E-02	3.56E-02	-4.7779
ENSG00000078399	HOXA9	0.8672	2.18E-02	3.57E-02	-4.7798
ENSG00000100028	SNRPD3	1.0990	2.18E-02	3.57E-02	-4.7800
ENSG00000175325	PROP1	1.0037	2.18E-02	3.57E-02	-4.7805
ENSG00000065427	KARS1	1.0746	2.18E-02	3.57E-02	-4.7807
ENSG00000133265	HSPBP1	0.9078	2.18E-02	3.57E-02	-4.7807
ENSG00000166997	CNPY4	1.1373	2.18E-02	3.57E-02	-4.7809
ENSG00000048545	GUCA1A	1.1031	2.18E-02	3.58E-02	-4.7822
ENSG00000255346	NOX5	1.1234	2.18E-02	3.58E-02	-4.7829
ENSG00000073146	MOV10L1	1.0344	2.18E-02	3.58E-02	-4.7830
ENSG00000101203	COL20A1	1.0723	2.19E-02	3.58E-02	-4.7847
ENSG00000101190	TCFL5	1.1557	2.19E-02	3.59E-02	-4.7850
ENSG00000198478	SH3BGRL2	0.7975	2.19E-02	3.59E-02	-4.7851
ENSG00000198951	NAGA	1.1260	2.19E-02	3.59E-02	-4.7852
ENSG00000132031	MATN3	1.0249	2.19E-02	3.59E-02	-4.7855
ENSG00000034239	EFCAB1	1.0598	2.19E-02	3.59E-02	-4.7870
ENSG00000148429	USP6NL	1.1905	2.19E-02	3.59E-02	-4.7870
ENSG00000171311	EXOSC1	0.9268	2.20E-02	3.60E-02	-4.7890
ENSG00000204520	MICA	0.8954	2.20E-02	3.60E-02	-4.7892
ENSG00000204540	PSORS1C1	1.0689	2.21E-02	3.61E-02	-4.7916
ENSG00000166634	SERPINB12	1.0052	2.22E-02	3.63E-02	-4.7956

Gene ID	Gene Symbol	FC	P.Value	adj.P.Val	B
ENSG00000112297	CRYBG1	1.1429	2.22E-02	3.63E-02	-4.7976
ENSG00000049089	COL9A2	1.3370	2.22E-02	3.63E-02	-4.7977
ENSG00000214447	FAM187A	1.0161	2.22E-02	3.64E-02	-4.7985
ENSG00000145979	TBC1D7	1.1158	2.22E-02	3.64E-02	-4.7987
ENSG00000109814	UGDH	0.8740	2.23E-02	3.64E-02	-4.7992
ENSG00000102547	CAB39L	0.9083	2.23E-02	3.64E-02	-4.7995
ENSG00000156531	PHF6	1.1452	2.23E-02	3.64E-02	-4.8002
ENSG00000141349	G6PC3	1.1011	2.23E-02	3.64E-02	-4.8006
ENSG00000106302	HYAL4	1.0590	2.23E-02	3.65E-02	-4.8010
ENSG00000158473	CD1D	0.7556	2.24E-02	3.66E-02	-4.8033
ENSG00000022556	NLRP2	0.8558	2.24E-02	3.66E-02	-4.8037
ENSG00000083123	BCKDHB	1.1413	2.24E-02	3.66E-02	-4.8043
ENSG00000225899	FRG2B	1.0202	2.24E-02	3.66E-02	-4.8050
ENSG00000183751	TBL3	1.0881	2.24E-02	3.66E-02	-4.8054
ENSG00000172238	ATOH1	1.0117	2.24E-02	3.67E-02	-4.8068
ENSG00000003137	CYP26B1	1.0208	2.25E-02	3.67E-02	-4.8077
ENSG00000137960	GIPC2	1.0402	2.25E-02	3.67E-02	-4.8079
ENSG00000189023	MAGEB16	1.1284	2.25E-02	3.67E-02	-4.8085
ENSG00000234469	CLDN34	1.0054	2.25E-02	3.67E-02	-4.8084
ENSG00000171130	ATP6V0E2	1.2269	2.25E-02	3.68E-02	-4.8103
ENSG00000185252	ZNF74	0.8775	2.26E-02	3.69E-02	-4.8120
ENSG00000158865	SLC5A11	1.0573	2.26E-02	3.69E-02	-4.8129
ENSG00000132669	RIN2	1.0638	2.26E-02	3.69E-02	-4.8132
ENSG00000188803	SHISA6	1.0050	2.26E-02	3.70E-02	-4.8142
ENSG00000136932	TRMO	1.0850	2.26E-02	3.70E-02	-4.8145
ENSG00000189320	FAM180A	1.0094	2.27E-02	3.70E-02	-4.8150
ENSG00000215547	DEFB115	1.0251	2.27E-02	3.70E-02	-4.8155
ENSG00000119888	EPCAM	1.3217	2.27E-02	3.70E-02	-4.8157
ENSG00000100884	CPNE6	1.0373	2.27E-02	3.71E-02	-4.8172
ENSG00000172014	ANKRD20A4P	1.1447	2.27E-02	3.71E-02	-4.8182
ENSG00000153015	CWC27	0.9124	2.28E-02	3.72E-02	-4.8189
ENSG00000197576	HOXA4	0.9167	2.28E-02	3.72E-02	-4.8195
ENSG00000167535	CACNB3	1.1585	2.28E-02	3.72E-02	-4.8211
ENSG00000126062	TMEM115	1.0893	2.29E-02	3.73E-02	-4.8230
ENSG00000127995	CASD1	1.1176	2.29E-02	3.73E-02	-4.8232
ENSG00000163645	ERICH6	1.0728	2.29E-02	3.74E-02	-4.8246
ENSG00000162066	AMDHD2	1.1063	2.29E-02	3.74E-02	-4.8251
ENSG00000169131	ZNF354A	0.9031	2.29E-02	3.74E-02	-4.8251
ENSG00000130701	RBBP8NL	1.0342	2.29E-02	3.74E-02	-4.8252
ENSG00000203857	HSD3B1	0.9451	2.29E-02	3.74E-02	-4.8256
ENSG00000145907	G3BP1	0.8741	2.30E-02	3.75E-02	-4.8272
ENSG00000160999	SH2B2	1.1610	2.30E-02	3.75E-02	-4.8277
ENSG00000117480	FAAH	1.1713	2.31E-02	3.76E-02	-4.8298
ENSG00000139915	MDGA2	1.0464	2.31E-02	3.76E-02	-4.8298
ENSG00000183873	SCN5A	1.0377	2.30E-02	3.76E-02	-4.8297
ENSG00000171224	FAM241B	1.2042	2.31E-02	3.76E-02	-4.8304
ENSG00000146425	DYNLT1	1.1207	2.31E-02	3.76E-02	-4.8307
ENSG00000189114	BLOC1S3	1.1057	2.31E-02	3.76E-02	-4.8308
ENSG00000182916	TCEAL7	1.0286	2.31E-02	3.77E-02	-4.8327
ENSG00000135838	NPL	0.8554	2.31E-02	3.77E-02	-4.8331
ENSG00000143867	OSR1	1.0287	2.32E-02	3.77E-02	-4.8337
ENSG00000130695	CEP85	1.1344	2.32E-02	3.78E-02	-4.8362
ENSG00000107758	PPP3CB	0.9029	2.32E-02	3.78E-02	-4.8368
ENSG00000262664	OVCA2	1.0762	2.32E-02	3.78E-02	-4.8367

Gene ID	Gene Symbol	FC	P.Value	adj.P.Val	B
ENSG00000102030	NAA10	1.1011	2.33E-02	3.79E-02	-4.8380
ENSG00000137513	NARS2	0.8899	2.33E-02	3.79E-02	-4.8385
ENSG00000115963	RND3	1.4418	2.33E-02	3.80E-02	-4.8394
ENSG00000157322	CLEC18A	0.9256	2.34E-02	3.80E-02	-4.8417
ENSG00000137707	BTG4	1.0178	2.34E-02	3.81E-02	-4.8428
ENSG00000114115	RBP1	1.2453	2.34E-02	3.82E-02	-4.8443
ENSG00000131943	C19orf12	0.9128	2.34E-02	3.82E-02	-4.8443
ENSG00000136273	HUS1	1.0817	2.35E-02	3.82E-02	-4.8450
ENSG00000157827	FMNL2	1.1544	2.35E-02	3.82E-02	-4.8456
ENSG00000102763	VWA8	0.9150	2.35E-02	3.82E-02	-4.8464
ENSG00000171872	KLF17	1.0187	2.36E-02	3.83E-02	-4.8485
ENSG00000197757	HOXC6	1.0612	2.36E-02	3.83E-02	-4.8488
ENSG00000173175	ADCY5	0.8758	2.36E-02	3.83E-02	-4.8491
ENSG00000163382	NAXE	1.1177	2.36E-02	3.84E-02	-4.8503
ENSG00000010626	LRRC23	1.1163	2.36E-02	3.84E-02	-4.8509
ENSG00000155087	ODF1	1.0281	2.36E-02	3.84E-02	-4.8517
ENSG00000148444	COMMD3	0.8630	2.36E-02	3.85E-02	-4.8519
ENSG00000082269	FAM135A	1.1192	2.37E-02	3.85E-02	-4.8533
ENSG00000134757	DSG3	1.0147	2.37E-02	3.85E-02	-4.8540
ENSG00000113555	PCDH12	0.9076	2.38E-02	3.86E-02	-4.8558
ENSG00000160588	MPZL3	1.1621	2.38E-02	3.87E-02	-4.8583
ENSG00000188820	CALHM6	0.7631	2.38E-02	3.87E-02	-4.8586
ENSG00000177047	IFNW1	0.9717	2.39E-02	3.88E-02	-4.8599
ENSG00000182459	TEX19	0.9158	2.39E-02	3.88E-02	-4.8610
ENSG00000150556	LYPD6B	0.7538	2.40E-02	3.89E-02	-4.8631
ENSG00000166200	COPS2	1.1059	2.40E-02	3.89E-02	-4.8634
ENSG00000171132	PRKCE	1.1847	2.40E-02	3.90E-02	-4.8640
ENSG00000091656	ZFHX4	0.8645	2.40E-02	3.90E-02	-4.8653
ENSG00000110583	NAA40	1.1015	2.40E-02	3.91E-02	-4.8662
ENSG00000162494	LRRC38	1.0739	2.42E-02	3.92E-02	-4.8705
ENSG00000255837	TAS2R20	1.0569	2.42E-02	3.93E-02	-4.8715
ENSG00000170927	PKHD1	1.0682	2.42E-02	3.93E-02	-4.8728
ENSG00000213973	ZNF99	1.0083	2.43E-02	3.95E-02	-4.8765
ENSG00000170265	ZNF282	1.0866	2.43E-02	3.95E-02	-4.8766
ENSG00000144045	DQX1	1.1437	2.44E-02	3.95E-02	-4.8775
ENSG00000163630	SYNPR	1.0258	2.44E-02	3.96E-02	-4.8783
ENSG00000147383	NSDHL	1.1181	2.44E-02	3.96E-02	-4.8785
ENSG00000183018	SPNS2	1.1044	2.44E-02	3.96E-02	-4.8786
ENSG00000197965	MPZL1	1.1248	2.44E-02	3.96E-02	-4.8795
ENSG00000171533	MAP6	1.0395	2.44E-02	3.97E-02	-4.8806
ENSG00000099817	POLR2E	0.9295	2.45E-02	3.97E-02	-4.8819
ENSG00000132386	SERPINF1	1.1394	2.45E-02	3.98E-02	-4.8837
ENSG00000147117	ZNF157	1.0345	2.46E-02	3.99E-02	-4.8852
ENSG00000125650	PSPN	0.9478	2.46E-02	3.99E-02	-4.8856
ENSG00000181499	OR6T1	1.0032	2.46E-02	4.00E-02	-4.8875
ENSG00000234224	TMEM229A	1.0574	2.46E-02	4.00E-02	-4.8876
ENSG00000142065	ZFP14	1.0936	2.47E-02	4.00E-02	-4.8882
ENSG00000180448	ARHGAP45	1.1063	2.47E-02	4.00E-02	-4.8889
ENSG00000102243	VGLL1	1.0071	2.47E-02	4.01E-02	-4.8897
ENSG00000134815	DHX34	0.8914	2.48E-02	4.02E-02	-4.8927
ENSG00000178307	TMEM11	1.0768	2.48E-02	4.02E-02	-4.8933
ENSG00000149418	ST14	1.4039	2.48E-02	4.02E-02	-4.8934
ENSG00000157119	KLHL40	1.0045	2.49E-02	4.03E-02	-4.8955
ENSG00000112761	CCN6	1.0272	2.49E-02	4.04E-02	-4.8965

Gene ID	Gene Symbol	FC	P.Value	adj.P.Val	B
ENSG00000107938	EDRF1	1.1169	2.49E-02	4.04E-02	-4.8978
ENSG00000007171	NOS2	1.0102	2.50E-02	4.05E-02	-4.8991
ENSG00000111907	TPD52L1	0.8617	2.50E-02	4.05E-02	-4.8993
ENSG00000181315	ZNF322	0.8704	2.50E-02	4.05E-02	-4.9007
ENSG00000142279	WTIP	0.9697	2.50E-02	4.05E-02	-4.9009
ENSG00000120742	SERP1	0.9341	2.50E-02	4.06E-02	-4.9017
ENSG00000112333	NR2E1	0.9789	2.51E-02	4.06E-02	-4.9028
ENSG00000120437	ACAT2	1.1060	2.51E-02	4.06E-02	-4.9030
ENSG00000087303	NID2	1.2006	2.51E-02	4.07E-02	-4.9035
ENSG00000205108	FAM205A	0.9947	2.51E-02	4.07E-02	-4.9053
ENSG00000112562	SMOC2	1.0690	2.52E-02	4.07E-02	-4.9056
ENSG00000162999	DUSP19	0.9393	2.52E-02	4.07E-02	-4.9056
ENSG00000121068	TBX2	1.0382	2.52E-02	4.09E-02	-4.9087
ENSG00000205213	LGR4	1.2267	2.53E-02	4.09E-02	-4.9091
ENSG00000204278	TMEM235	1.0039	2.53E-02	4.10E-02	-4.9111
ENSG00000163823	CCR1	0.7650	2.53E-02	4.10E-02	-4.9116
ENSG00000175711	B3GNTL1	0.8546	2.53E-02	4.10E-02	-4.9118
ENSG00000186523	FAM86B1	1.1314	2.53E-02	4.10E-02	-4.9118
ENSG00000074416	MGLL	1.2788	2.54E-02	4.11E-02	-4.9134
ENSG00000136425	CIB2	0.8655	2.54E-02	4.11E-02	-4.9133
ENSG00000163499	CRYBA2	1.0280	2.54E-02	4.11E-02	-4.9148
ENSG00000133958	UNC79	1.0416	2.54E-02	4.11E-02	-4.9149
ENSG00000104412	EMC2	0.8894	2.55E-02	4.12E-02	-4.9163
ENSG00000136931	NR5A1	1.0207	2.55E-02	4.12E-02	-4.9164
ENSG00000112077	RHAG	0.8743	2.55E-02	4.12E-02	-4.9167
ENSG00000172137	CALB2	1.0174	2.55E-02	4.13E-02	-4.9179
ENSG00000226792	C13orf42	1.0582	2.55E-02	4.13E-02	-4.9180
ENSG00000075043	KCNQ2	1.0103	2.56E-02	4.13E-02	-4.9191
ENSG00000196734	LCE1B	1.0184	2.56E-02	4.14E-02	-4.9205
ENSG00000075945	KIFAP3	1.1434	2.56E-02	4.14E-02	-4.9218
ENSG00000161270	NPHS1	1.0393	2.57E-02	4.15E-02	-4.9224
ENSG00000080493	SLC4A4	1.0659	2.57E-02	4.15E-02	-4.9234
ENSG00000131910	NR0B2	1.0101	2.57E-02	4.15E-02	-4.9241
ENSG00000255298	OR8G5	1.0217	2.57E-02	4.16E-02	-4.9247
ENSG00000164379	FOXQ1	1.0373	2.57E-02	4.16E-02	-4.9255
ENSG00000186860	KRTAP17-1	1.0130	2.58E-02	4.16E-02	-4.9259
ENSG00000196476	C20orf96	1.1250	2.58E-02	4.16E-02	-4.9261
ENSG00000213906	LTB4R2	1.1379	2.58E-02	4.17E-02	-4.9267
ENSG00000120149	MSX2	1.0167	2.58E-02	4.17E-02	-4.9268
ENSG00000163219	ARHGAP25	0.8992	2.58E-02	4.17E-02	-4.9277
ENSG00000112139	MDGA1	1.2344	2.58E-02	4.17E-02	-4.9289
ENSG00000153157	SYCP2L	1.0549	2.59E-02	4.18E-02	-4.9296
ENSG00000117298	ECE1	1.1095	2.59E-02	4.18E-02	-4.9306
ENSG00000147419	CCDC25	1.0854	2.59E-02	4.18E-02	-4.9307
ENSG00000166923	GREM1	0.9367	2.59E-02	4.18E-02	-4.9310
ENSG00000173546	CSPG4	1.0345	2.60E-02	4.19E-02	-4.9330
ENSG00000149634	SPATA25	1.0988	2.60E-02	4.19E-02	-4.9331
ENSG00000229183	PGA4	0.9727	2.60E-02	4.20E-02	-4.9338
ENSG00000105996	HOXA2	0.9683	2.60E-02	4.20E-02	-4.9350
ENSG00000164284	GRPEL2	1.1059	2.60E-02	4.20E-02	-4.9350
ENSG00000229937	PRPS1L1	1.0143	2.60E-02	4.20E-02	-4.9355
ENSG00000153246	PLA2R1	1.0362	2.61E-02	4.21E-02	-4.9366
ENSG00000060762	MPC1	1.1259	2.61E-02	4.21E-02	-4.9380
ENSG00000065308	TRAM2	1.1454	2.61E-02	4.21E-02	-4.9380

Gene ID	Gene Symbol	FC	P.Value	adj.P.Val	B
ENSG00000171004	HS6ST2	1.0810	2.61E-02	4.22E-02	-4.9383
ENSG00000050426	LETMD1	0.9237	2.61E-02	4.22E-02	-4.9387
ENSG00000132330	SCLY	1.1058	2.62E-02	4.22E-02	-4.9392
ENSG00000181803	OR6S1	0.9745	2.62E-02	4.22E-02	-4.9396
ENSG00000173208	ABCD2	1.1498	2.62E-02	4.22E-02	-4.9401
ENSG00000205846	CLEC6A	0.9848	2.62E-02	4.22E-02	-4.9401
ENSG00000140932	CMTM2	0.8378	2.62E-02	4.23E-02	-4.9409
ENSG00000134762	DSC3	1.0093	2.62E-02	4.23E-02	-4.9410
ENSG00000185532	PRKG1	0.7940	2.62E-02	4.23E-02	-4.9421
ENSG00000126950	TMEM35A	1.0216	2.63E-02	4.23E-02	-4.9425
ENSG00000151962	RBM46	1.0648	2.63E-02	4.23E-02	-4.9428
ENSG00000183309	ZNF623	1.1046	2.63E-02	4.24E-02	-4.9440
ENSG00000124766	SOX4	1.2708	2.63E-02	4.25E-02	-4.9455
ENSG00000203985	LDLRAD1	1.0063	2.64E-02	4.25E-02	-4.9460
ENSG00000109680	TBC1D19	1.0847	2.64E-02	4.25E-02	-4.9462
ENSG00000259207	ITGB3	1.0856	2.64E-02	4.25E-02	-4.9471
ENSG00000130958	SLC35D2	0.7815	2.64E-02	4.25E-02	-4.9473
ENSG00000186297	GABRA5	1.0835	2.64E-02	4.25E-02	-4.9476
ENSG00000134470	IL15RA	1.1084	2.64E-02	4.26E-02	-4.9484
ENSG00000130988	RGN	1.0742	2.64E-02	4.26E-02	-4.9486
ENSG00000125505	MBOAT7	1.1050	2.65E-02	4.26E-02	-4.9499
ENSG00000189091	SF3B3	1.1109	2.65E-02	4.27E-02	-4.9502
ENSG00000155850	SLC26A2	0.8889	2.66E-02	4.28E-02	-4.9534
ENSG00000100568	VTI1B	0.9187	2.66E-02	4.29E-02	-4.9545
ENSG00000038295	TLL1	1.0170	2.67E-02	4.29E-02	-4.9558
ENSG00000165678	GHITM	1.0789	2.67E-02	4.29E-02	-4.9557
ENSG00000272899	ATP6V1FNB	0.8785	2.67E-02	4.29E-02	-4.9560
ENSG00000162572	SCNN1D	1.1353	2.67E-02	4.30E-02	-4.9568
ENSG00000129696	TTI2	0.9106	2.67E-02	4.30E-02	-4.9571
ENSG00000116209	TMEM59	0.8984	2.68E-02	4.31E-02	-4.9595
ENSG00000126432	PRDX5	0.9250	2.68E-02	4.32E-02	-4.9611
ENSG00000187372	PCDHB13	0.9238	2.69E-02	4.32E-02	-4.9623
ENSG00000075884	ARHGAP15	1.1495	2.69E-02	4.33E-02	-4.9637
ENSG00000163291	PAQR3	1.1167	2.69E-02	4.33E-02	-4.9640
ENSG00000126337	KRT36	0.9934	2.70E-02	4.34E-02	-4.9654
ENSG00000147256	ARHGAP36	1.0206	2.70E-02	4.34E-02	-4.9654
ENSG00000118894	EEF2KMT	0.8717	2.70E-02	4.34E-02	-4.9657
ENSG00000261594	TPBGL	1.0109	2.70E-02	4.35E-02	-4.9680
ENSG00000173950	XXYLT1	1.1114	2.71E-02	4.35E-02	-4.9686
ENSG00000183665	TRMT12	1.0835	2.71E-02	4.35E-02	-4.9686
ENSG00000124813	RUNX2	1.2916	2.71E-02	4.35E-02	-4.9687
ENSG00000213760	ATP6V1G2	1.1032	2.71E-02	4.35E-02	-4.9689
ENSG00000169598	DFFB	0.8952	2.71E-02	4.36E-02	-4.9701
ENSG00000198546	ZNF511	1.0834	2.71E-02	4.36E-02	-4.9703
ENSG00000176845	METRNL	1.3231	2.71E-02	4.36E-02	-4.9704
ENSG00000196632	WNK3	1.0702	2.71E-02	4.36E-02	-4.9709
ENSG00000212864	RNF208	0.8912	2.71E-02	4.36E-02	-4.9713
ENSG00000185730	ZNF696	0.8738	2.72E-02	4.37E-02	-4.9720
ENSG00000228594	FNDC10	1.0799	2.72E-02	4.37E-02	-4.9728
ENSG00000080644	CHRNA3	1.0451	2.72E-02	4.37E-02	-4.9729
ENSG00000122707	RECK	1.1345	2.73E-02	4.38E-02	-4.9752
ENSG00000173436	MICOS10	1.0703	2.73E-02	4.38E-02	-4.9754
ENSG00000142949	PTPRF	1.1642	2.73E-02	4.39E-02	-4.9767
ENSG00000185352	HS6ST3	0.9914	2.73E-02	4.39E-02	-4.9768

Gene ID	Gene Symbol	FC	P.Value	adj.P.Val	B
ENSG00000186446	ZNF501	0.8942	2.73E-02	4.39E-02	-4.9775
ENSG00000213937	CLDN9	1.0476	2.73E-02	4.39E-02	-4.9774
ENSG00000176973	FAM89B	1.1238	2.74E-02	4.39E-02	-4.9782
ENSG00000112499	SLC22A2	1.0178	2.74E-02	4.39E-02	-4.9786
ENSG00000144015	TRIM43	1.0075	2.74E-02	4.40E-02	-4.9787
ENSG00000083457	ITGAE	1.1097	2.74E-02	4.40E-02	-4.9794
ENSG00000110756	HPS5	0.8939	2.74E-02	4.40E-02	-4.9801
ENSG00000214414	TRIM77	1.0181	2.74E-02	4.40E-02	-4.9804
ENSG00000148513	ANKRD30A	1.0744	2.74E-02	4.40E-02	-4.9807
ENSG00000214237	MINDY4B	1.0184	2.74E-02	4.40E-02	-4.9807
ENSG00000178096	BOLA1	0.8605	2.75E-02	4.41E-02	-4.9820
ENSG00000111885	MAN1A1	1.1759	2.75E-02	4.41E-02	-4.9822
ENSG00000198894	CIPC	1.0996	2.75E-02	4.41E-02	-4.9828
ENSG00000177151	OR2T35	1.0049	2.76E-02	4.42E-02	-4.9848
ENSG00000172171	TEFM	0.9021	2.76E-02	4.43E-02	-4.9858
ENSG00000160460	SPTBN4	1.1138	2.76E-02	4.43E-02	-4.9859
ENSG00000169026	SLC49A3	1.1902	2.76E-02	4.43E-02	-4.9863
ENSG00000138028	CGREF1	0.7538	2.77E-02	4.44E-02	-4.9877
ENSG00000198298	ZNF485	0.8953	2.77E-02	4.44E-02	-4.9884
ENSG00000115841	RMDN2	0.8850	2.78E-02	4.45E-02	-4.9905
ENSG00000124237	C20orf85	1.0069	2.78E-02	4.45E-02	-4.9913
ENSG00000091483	FH	1.1386	2.78E-02	4.45E-02	-4.9916
ENSG00000103490	PYCARD	0.8607	2.78E-02	4.46E-02	-4.9924
ENSG00000109099	PMP22	1.1221	2.79E-02	4.47E-02	-4.9939
ENSG00000198807	PAX9	1.0311	2.79E-02	4.47E-02	-4.9944
ENSG00000168395	ING5	1.0984	2.79E-02	4.47E-02	-4.9949
ENSG00000251692	PTX4	1.0242	2.79E-02	4.48E-02	-4.9959
ENSG00000170835	CEL	1.1003	2.80E-02	4.48E-02	-4.9970
ENSG00000130772	MED18	0.8843	2.80E-02	4.48E-02	-4.9976
ENSG00000101210	EEF1A2	1.1266	2.80E-02	4.49E-02	-4.9986
ENSG00000120160	EQTN	0.9722	2.80E-02	4.49E-02	-4.9991
ENSG00000155530	LRGUK	1.0576	2.80E-02	4.49E-02	-4.9991
ENSG00000115956	PLEK	1.2817	2.80E-02	4.49E-02	-4.9996
ENSG00000023572	GLRX2	1.1222	2.81E-02	4.49E-02	-4.9999
ENSG00000180370	PAK2	1.0980	2.81E-02	4.49E-02	-4.9998
ENSG00000084774	CAD	1.1480	2.81E-02	4.50E-02	-5.0009
ENSG00000157916	RER1	1.0806	2.81E-02	4.50E-02	-5.0018
ENSG00000115596	WNT6	0.8883	2.82E-02	4.51E-02	-5.0031
ENSG00000167653	PSCA	1.0695	2.82E-02	4.51E-02	-5.0036
ENSG00000237172	B3GNT9	0.8546	2.82E-02	4.52E-02	-5.0051
ENSG00000163001	CFAP36	1.1251	2.82E-02	4.52E-02	-5.0054
ENSG00000171873	ADRA1D	1.0175	2.83E-02	4.52E-02	-5.0065
ENSG00000183246	RIMBP3C	0.9788	2.83E-02	4.52E-02	-5.0065
ENSG00000151575	TEX9	1.2163	2.84E-02	4.54E-02	-5.0092
ENSG00000163217	BMP10	1.0065	2.84E-02	4.54E-02	-5.0093
ENSG00000134253	TRIM45	1.1150	2.84E-02	4.54E-02	-5.0098
ENSG00000163431	LMOD1	1.0295	2.84E-02	4.54E-02	-5.0101
ENSG00000175105	ZNF654	1.0891	2.84E-02	4.54E-02	-5.0104
ENSG00000167633	KIR3DL1	1.0514	2.84E-02	4.55E-02	-5.0109
ENSG00000078401	EDN1	1.2479	2.84E-02	4.55E-02	-5.0110
ENSG00000110446	SLC15A3	1.2808	2.84E-02	4.55E-02	-5.0117
ENSG00000188070	ZFTA	1.1280	2.85E-02	4.55E-02	-5.0126
ENSG00000113048	MRPS27	0.8769	2.85E-02	4.55E-02	-5.0128
ENSG00000108666	C17orf75	1.1026	2.85E-02	4.56E-02	-5.0148

Gene ID	Gene Symbol	FC	P.Value	adj.P.Val	B
ENSG00000162591	MEGF6	1.0940	2.86E-02	4.56E-02	-5.0150
ENSG00000105492	SIGLEC6	1.0618	2.86E-02	4.57E-02	-5.0169
ENSG00000070526	ST6GALNAC1	1.0396	2.86E-02	4.58E-02	-5.0175
ENSG00000178031	ADAMTSL1	0.8865	2.87E-02	4.58E-02	-5.0190
ENSG00000196549	MME	1.1795	2.87E-02	4.58E-02	-5.0189
ENSG00000107331	ABCA2	0.8502	2.87E-02	4.59E-02	-5.0207
ENSG00000186723	OR10H1	1.0037	2.88E-02	4.60E-02	-5.0218
ENSG00000169903	TM4SF4	0.9827	2.88E-02	4.60E-02	-5.0223
ENSG00000197496	SLC2A10	1.2555	2.88E-02	4.60E-02	-5.0224
ENSG00000251664	PCDHA12	1.0099	2.88E-02	4.61E-02	-5.0237
ENSG00000182508	LHFPL1	1.0925	2.89E-02	4.61E-02	-5.0244
ENSG00000130598	TNNI2	1.1691	2.89E-02	4.61E-02	-5.0247
ENSG00000135392	DNAJC14	1.1074	2.89E-02	4.61E-02	-5.0251
ENSG00000167842	MIS12	1.1207	2.89E-02	4.62E-02	-5.0254
ENSG00000182903	ZNF721	1.1436	2.89E-02	4.62E-02	-5.0260
ENSG00000120896	SORBS3	1.1973	2.89E-02	4.62E-02	-5.0262
ENSG00000196539	OR2T3	1.0051	2.90E-02	4.63E-02	-5.0277
ENSG00000230453	ANKRD18B	1.0944	2.90E-02	4.63E-02	-5.0279
ENSG00000236446	CT47B1	1.0391	2.90E-02	4.63E-02	-5.0283
ENSG00000212659	KRTAP9-6	1.0107	2.91E-02	4.64E-02	-5.0303
ENSG00000170820	FSHR	1.0049	2.91E-02	4.64E-02	-5.0307
ENSG00000113384	GOLPH3	0.9182	2.91E-02	4.64E-02	-5.0308
ENSG00000165092	ALDH1A1	0.8443	2.91E-02	4.65E-02	-5.0316
ENSG00000115866	DARS1	1.0803	2.92E-02	4.66E-02	-5.0338
ENSG00000188386	PPP3R2	0.9942	2.92E-02	4.66E-02	-5.0340
ENSG00000118160	SLC8A2	1.0230	2.92E-02	4.66E-02	-5.0345
ENSG00000214782	MS4A18	1.0079	2.93E-02	4.67E-02	-5.0359
ENSG00000204348	DXO	1.0919	2.93E-02	4.67E-02	-5.0360
ENSG00000260802	SERTM2	1.0783	2.93E-02	4.67E-02	-5.0367
ENSG00000120925	RNF170	0.8836	2.93E-02	4.68E-02	-5.0385
ENSG00000125485	DDX31	1.0749	2.93E-02	4.68E-02	-5.0385
ENSG00000148450	MSRB2	1.1049	2.93E-02	4.68E-02	-5.0385
ENSG00000198759	EGFL6	1.0397	2.93E-02	4.68E-02	-5.0385
ENSG00000214511	HIGD1C	0.9236	2.94E-02	4.68E-02	-5.0394
ENSG00000133742	CA1	0.7513	2.94E-02	4.69E-02	-5.0402
ENSG00000139835	GRTP1	1.1444	2.94E-02	4.69E-02	-5.0402
ENSG00000083535	PIBF1	0.9015	2.94E-02	4.69E-02	-5.0404
ENSG00000139044	B4GALNT3	0.9402	2.94E-02	4.69E-02	-5.0410
ENSG00000176896	TCEANC	1.0957	2.94E-02	4.69E-02	-5.0413
ENSG00000139988	RDH12	1.1348	2.95E-02	4.69E-02	-5.0421
ENSG00000145632	PLK2	1.2385	2.95E-02	4.70E-02	-5.0433
ENSG00000042753	AP2S1	0.9257	2.95E-02	4.70E-02	-5.0434
ENSG00000164010	ERMAP	0.9197	2.95E-02	4.71E-02	-5.0443
ENSG00000173678	SPDYE2B	1.0603	2.95E-02	4.71E-02	-5.0443
ENSG00000124196	GTSF1L	1.0419	2.96E-02	4.71E-02	-5.0454
ENSG00000151773	CCDC122	1.0930	2.96E-02	4.72E-02	-5.0467
ENSG00000110442	COMMD9	1.0809	2.97E-02	4.72E-02	-5.0478
ENSG00000151240	DIP2C	1.1353	2.97E-02	4.73E-02	-5.0482
ENSG00000119446	RBM18	1.0767	2.97E-02	4.73E-02	-5.0483
ENSG00000170950	PGK2	1.0064	2.97E-02	4.73E-02	-5.0492
ENSG00000257008	GPR142	1.0513	2.97E-02	4.73E-02	-5.0498
ENSG00000167487	KLHL26	1.0820	2.97E-02	4.73E-02	-5.0499
ENSG00000083097	DOP1A	1.1136	2.98E-02	4.75E-02	-5.0530
ENSG00000146701	MDH2	1.0772	2.99E-02	4.75E-02	-5.0538

Gene ID	Gene Symbol	FC	P.Value	adj.P.Val	B
ENSG00000164128	NPY1R	1.0171	2.99E-02	4.76E-02	-5.0546
ENSG00000257046	SLCO1B3-SLCO1B7	1.0171	2.99E-02	4.76E-02	-5.0552
ENSG00000115257	PCSK4	1.1796	2.99E-02	4.76E-02	-5.0557
ENSG00000197616	MYH6	1.0078	2.99E-02	4.76E-02	-5.0558
ENSG00000147475	ERLIN2	1.1144	3.00E-02	4.77E-02	-5.0562
ENSG00000136950	ARPC5L	1.1214	3.00E-02	4.77E-02	-5.0568
ENSG00000151623	NR3C2	1.1367	3.00E-02	4.78E-02	-5.0583
ENSG00000141076	UTP4	1.0839	3.01E-02	4.78E-02	-5.0592
ENSG00000182253	SYNM	1.2508	3.02E-02	4.80E-02	-5.0627
ENSG00000196805	SPRR2B	1.0116	3.02E-02	4.80E-02	-5.0628
ENSG00000171487	NLRP5	1.0214	3.02E-02	4.80E-02	-5.0631
ENSG00000253293	HOXA10	0.8950	3.02E-02	4.80E-02	-5.0632
ENSG00000203778	FAM229B	1.1699	3.02E-02	4.81E-02	-5.0643
ENSG00000166801	FAM111A	0.8618	3.02E-02	4.81E-02	-5.0645
ENSG00000086504	MRPL28	1.0873	3.03E-02	4.81E-02	-5.0648
ENSG00000173585	CCR9	1.0812	3.04E-02	4.82E-02	-5.0676
ENSG00000221989	OR2A2	0.9913	3.04E-02	4.82E-02	-5.0677
ENSG00000140285	FGF7	0.9059	3.04E-02	4.83E-02	-5.0691
ENSG00000152430	BOLL	1.0458	3.04E-02	4.84E-02	-5.0702
ENSG00000105771	SMG9	1.1168	3.05E-02	4.84E-02	-5.0705
ENSG00000196290	NIF3L1	0.8945	3.05E-02	4.85E-02	-5.0725
ENSG00000151611	MMAA	1.0786	3.05E-02	4.85E-02	-5.0731
ENSG00000100216	TOMM22	0.9344	3.06E-02	4.86E-02	-5.0739
ENSG00000183496	MEX3B	1.1215	3.06E-02	4.86E-02	-5.0743
ENSG00000086300	SNX10	0.8476	3.06E-02	4.86E-02	-5.0747
ENSG00000106049	HIBADH	0.9043	3.07E-02	4.87E-02	-5.0762
ENSG00000198838	RYR3	1.1171	3.07E-02	4.88E-02	-5.0780
ENSG00000101115	SALL4	1.0889	3.07E-02	4.88E-02	-5.0785
ENSG00000161298	ZNF382	1.0824	3.08E-02	4.89E-02	-5.0809
ENSG00000173349	SFT2D3	1.1905	3.08E-02	4.89E-02	-5.0811
ENSG00000032444	PNPLA6	1.0868	3.09E-02	4.91E-02	-5.0837
ENSG00000148426	PROSER2	1.0422	3.09E-02	4.91E-02	-5.0837
ENSG00000169562	GJB1	1.0038	3.10E-02	4.91E-02	-5.0843
ENSG00000113396	SLC27A6	1.0180	3.10E-02	4.91E-02	-5.0849
ENSG00000152683	SLC30A6	0.8900	3.10E-02	4.92E-02	-5.0852
ENSG00000008018	PSMB1	1.0781	3.10E-02	4.92E-02	-5.0860
ENSG00000134480	CCNH	1.1156	3.10E-02	4.92E-02	-5.0867
ENSG00000095002	MSH2	1.1700	3.11E-02	4.93E-02	-5.0872
ENSG00000181418	DDN	1.1172	3.11E-02	4.93E-02	-5.0874
ENSG00000067177	PHKA1	1.1652	3.11E-02	4.93E-02	-5.0879
ENSG00000232040	ZBED9	0.8842	3.12E-02	4.94E-02	-5.0899
ENSG00000183185	GABRR3	1.0290	3.12E-02	4.94E-02	-5.0902
ENSG00000223591	CENPVL1	1.0116	3.12E-02	4.94E-02	-5.0907
ENSG00000212747	RTL8B	1.1362	3.12E-02	4.95E-02	-5.0911
ENSG00000161249	DMKN	1.2623	3.12E-02	4.95E-02	-5.0923
ENSG00000184979	USP18	0.8147	3.13E-02	4.96E-02	-5.0937
ENSG00000164889	SLC4A2	1.0891	3.13E-02	4.97E-02	-5.0948
ENSG00000178764	ZHX2	1.1073	3.13E-02	4.97E-02	-5.0951
ENSG00000172020	GAP43	1.0593	3.14E-02	4.98E-02	-5.0979
ENSG00000136872	ALDOB	1.1042	3.15E-02	5.00E-02	-5.1000
ENSG00000168876	ANKRD49	1.0981	3.15E-02	5.00E-02	-5.1005

Supplementary Table S3. List of significant gene sets enriched in last versus first NONO quartile (194 MM in each group) by GSEA analysis. Up- and down-regulated gene sets (red and blue, respectively) of Kegg, Hallmark, Reactome collections (version 7.2) are ordered according to nominal p-value and Normalized Enrichment Score (NES).

NAME	SIZE	NES	NOM p-val	FDR q-val
HALLMARK_G2M_CHECKPOINT	173	3.907	0.0000	0.0000
HALLMARK_TNFA_SIGNALING_VIA_NFKB	145	3.592	0.0000	0.0000
HALLMARK_APOPTOSIS	106	3.411	0.0000	0.0000
HALLMARK_MITOTIC_SPINDLE	171	3.319	0.0000	0.0000
HALLMARK_E2F_TARGETS	168	3.297	0.0000	0.0000
KEGG_CELL_CYCLE	98	3.237	0.0000	0.0000
REACTOME_CELL_CYCLE_MITOTIC	422	3.133	0.0000	0.0000
REACTOME_RESOLUTION_OF_SISTER_CHROMATID_COHESION	105	3.120	0.0000	0.0000
REACTOME_MITOTIC_PROMETAPHASE	169	3.030	0.0000	0.0000
REACTOME_CHROMATIN_MODIFYING_ENZYMES	174	3.005	0.0000	0.0000
REACTOME_CELL_CYCLE_CHECKPOINTS	210	2.892	0.0000	0.0000
REACTOME_RHO_GTPASES_ACTIVATE_FORMINS	111	2.873	0.0000	0.0000
KEGG_P53_SIGNALING_PATHWAY	51	2.860	0.0000	0.0000
REACTOME_M_PHASE	302	2.841	0.0000	0.0000
REACTOME_RHO_GTPASE_EFFECTORS	217	2.799	0.0000	0.0000
REACTOME_MITOTIC_METAPHASE_AND_ANAPHASE	188	2.756	0.0000	0.0001
REACTOME_RHO_GTPASE_CYCLE	340	2.741	0.0000	0.0001
KEGG_ACUTE_MYELOID_LEUKEMIA	44	2.736	0.0000	0.0000
KEGG_PROSTATE_CANCER	72	2.735	0.0000	0.0000
HALLMARK_IL2_STAT5_SIGNALING	137	2.731	0.0000	0.0000
REACTOME_SUMOYLATION	134	2.718	0.0000	0.0001
REACTOME_ONCOGENIC_MAPK_SIGNALING	55	2.718	0.0000	0.0001
REACTOME_CELLULAR_SENESCENCE	118	2.713	0.0000	0.0001
REACTOME_OXIDATIVE_STRESS_INDUCED_SENESCENCE	69	2.688	0.0000	0.0001
KEGG_COLORECTAL_CANCER	47	2.663	0.0000	0.0000
REACTOME_HDMS_DEMETHYLATE_HISTONES	19	2.655	0.0000	0.0001
REACTOME_NUCLEAR_ENVELOPE_NE_REASSEMBLY	59	2.630	0.0000	0.0001
REACTOME_SIGNALING_BY_NTRKS	92	2.621	0.0000	0.0001
KEGG_CHRONIC_MYELOID_LEUKEMIA	56	2.619	0.0000	0.0000
KEGG_ERBB_SIGNALING_PATHWAY	65	2.613	0.0000	0.0000
REACTOME_MITOTIC_SPINDLE_CHECKPOINT	88	2.612	0.0000	0.0002
KEGG_NEUROTROPHIN_SIGNALING_PATHWAY	99	2.607	0.0000	0.0000
REACTOME_PKMTS_METHYLATE_HISTONE_LYSINES	39	2.589	0.0000	0.0001
REACTOME_SIGNALING_BY_ERYTHROPOIETIN	20	2.587	0.0000	0.0002
REACTOME_ESTROGEN_DEPENDENT_GENE_EXPRESSION	82	2.585	0.0000	0.0002
REACTOME_SEPARATION_OF_SISTER_CHROMATIDS	152	2.582	0.0000	0.0002
REACTOME_AURKA_ACTIVATION_BY_TPX2	61	2.579	0.0000	0.0002
REACTOME_MITOTIC_PROPHASE	80	2.572	0.0000	0.0002
HALLMARK_TGF_BETA_SIGNALING	40	2.566	0.0000	0.0000
REACTOME_ANCHORING_OF_THE_BASAL_BODY_TO_THE_PLASMA_MEMBRANE	83	2.562	0.0000	0.0002
REACTOME_ONCOGENE_INDUCED_SENESCENCE	31	2.537	0.0000	0.0002
REACTOME_CIRCADIAN_CLOCK	59	2.531	0.0000	0.0002
REACTOME_SIGNALING_BY_ALK_IN_CANCER	45	2.529	0.0000	0.0002
REACTOME_NUCLEAR_ENVELOPE_BREAKDOWN	42	2.525	0.0000	0.0002
REACTOME_HDACS_DEACETYLATE_HISTONES	39	2.513	0.0000	0.0002
REACTOME_SIGNALING_BY_TGF_BETA_RECEPTOR_COMPLEX _ _	57	2.513	0.0000	0.0002
EROTRIMER	38	2.513	0.0000	0.0002
REACTOME_REGULATION_OF_TP53_ACTIVITY	126	2.502	0.0000	0.0002
REACTOME_HCMV_EARLY_EVENTS	71	2.500	0.0000	0.0002
REACTOME_RHOC_GTPASE_CYCLE	59	2.486	0.0000	0.0002
KEGG_PATHOGENIC_ESCHERICHIA_COLI_INFECTION	39	2.468	0.0000	0.0002
REACTOME_TRANSPORT_OF_MATURE_TRANSCRIPT_TO_CYTOPLASM	69	2.467	0.0000	0.0003
REACTOME_EPIGENETIC_REGULATION_OF_GENE_EXPRESSION	87	2.467	0.0000	0.0003

NAME	SIZE	NES	NOM p-val	FDR q-val
REACTOME_SUMOYLATION_OF_DNA_DAMAGE_RESPONSE_AND_REPAIR_PROTEINS	61	2.463	0.0000	0.0003
REACTOME_RECRUITMENT_OF_MITOTIC_CENTROSOME_PROTEINS_AND_COMPLEXES	70	2.456	0.0000	0.0003
REACTOME_ACTIVATION_OF_BH3_ONLY_PROTEINS	26	2.449	0.0000	0.0004
KEGG_B_CELL_RECEPTOR_SIGNALING_PATHWAY	53	2.443	0.0000	0.0003
REACTOME_TRANSCRIPTIONAL_REGULATION_BY_TP53	273	2.438	0.0000	0.0004
REACTOME_ESR_MEDIATED_SIGNALING	133	2.434	0.0000	0.0004
REACTOME_SIGNALING_BY_ALK	18	2.432	0.0000	0.0003
REACTOME_DISEASES_OF_SIGNAL_TRANSDUCTION_BY_GROWTH_FACTOR_RECEPTORS_AND_SECOND_MESSENGERS	307	2.432	0.0000	0.0003
REACTOME_RHOF_GTPASE_CYCLE	34	2.429	0.0000	0.0003
HALLMARK_INTERFERON_GAMMA_RESPONSE	116	2.428	0.0000	0.0000
REACTOME_INITIATION_OF_NUCLEAR_ENVELOPE_NE_REFORMATION	15	2.428	0.0000	0.0003
REACTOME_SUMOYLATION_OF_DNA_REPLICATION_PROTEINS	39	2.425	0.0000	0.0004
REACTOME_RAC2_GTPASE_CYCLE	71	2.417	0.0000	0.0004
REACTOME_DEATH_RECEPTOR_SIGNALLING	102	2.415	0.0000	0.0004
REACTOME_RHOQ_GTPASE_CYCLE	46	2.415	0.0000	0.0004
REACTOME_MITOTIC_G1_PHASE_AND_G1_S_TRANSITION	121	2.410	0.0000	0.0004
REACTOME_RECRUITMENT_OF_NUMA_TO_MITOTIC_CENTROSOMES	79	2.409	0.0000	0.0004
KEGG_PROGESTERONE_MEDIATED_OOCYTE_MATURATION	66	2.408	0.0000	0.0004
REACTOME_PROCESSING_OF_CAPPED_INTRON_CONTAINING_PRE_MRNA	198	2.399	0.0000	0.0004
REACTOME_G2_M_CHECKPOINTS	110	2.398	0.0000	0.0004
REACTOME_NS1_MEDIATED_EFFECTS_ON_HOST_PATHWAYS	34	2.392	0.0000	0.0004
HALLMARK_P53_PATHWAY	144	2.391	0.0000	0.0000
REACTOME_ACTIVATION_OF_THE_PRE_REPLICATIVE_COMPLEX	28	2.386	0.0000	0.0005
REACTOME_RHOB_GTPASE_CYCLE	55	2.378	0.0000	0.0006
REACTOME_FACTORS_INVOLVED_IN_MEGAKARYOCYTE_DEVELOPMENT_AND_PLATELET_PRODUCTION	113	2.377	0.0000	0.0006
REACTOME_RHOA_GTPASE_CYCLE	108	2.374	0.0000	0.0006
KEGG_TOLL_LIKE_RECEPTOR_SIGNALING_PATHWAY	50	2.369	0.0000	0.0005
REACTOME_DDX58_IFIH1_MEDIATED_INDUCTION_OF_INTERFERON_ALPHA_BETA	51	2.365	0.0000	0.0006
REACTOME_SIGNALING_BY_BRAF_AND_RAF1_FUSIONS	43	2.364	0.0000	0.0006
REACTOME_REGULATION_OF_PLK1_ACTIVITY_AT_G2_M_TRANSITION	73	2.363	0.0000	0.0006
REACTOME_SIGNALLING_TO_ERKS	25	2.360	0.0000	0.0006
REACTOME_REGULATION_OF_MECP2_EXPRESSION_AND_ACTIVITY	28	2.359	0.0000	0.0006
REACTOME_MITOTIC_G2_G2_M_PHASES	162	2.353	0.0000	0.0007
REACTOME_G2_M_DNA_DAMAGE_CHECKPOINT	49	2.348	0.0000	0.0007
REACTOME_DOWNREGULATION_OF_SMAD2_3_SMAD4_TRANSCRIPTIONAL_ACTIVITY	25	2.347	0.0000	0.0007
REACTOME_P75_NTR_RECEPTOR_MEDIATED_SIGNALLING	68	2.347	0.0000	0.0007
REACTOME_CELLULAR_RESPONSE_TO_HEAT_STRESS	75	2.344	0.0000	0.0007
KEGG_ENDOMETRIAL_CANCER	42	2.337	0.0000	0.0005
REACTOME_GENE_SILENCING_BY_RNA	73	2.326	0.0000	0.0009
REACTOME_FCERI_MEDIATED_MAPK_ACTIVATION	22	2.322	0.0000	0.0009
REACTOME_PI_METABOLISM	66	2.318	0.0000	0.0009
KEGG_T_CELL_RECEPTOR_SIGNALING_PATHWAY	77	2.312	0.0000	0.0007
REACTOME_RHOD_GTPASE_CYCLE	46	2.310	0.0000	0.0010
HALLMARK_PI3K_AKT_MTOR_SIGNALING	75	2.310	0.0000	0.0000
REACTOME_RUNX1_INTERACTS_WITH_CO_FACTORS_WHOSE_PRECISE_EFFECT_ON_RUNX1_TARGETS_IS_NOT_KNOWN	31	2.308	0.0000	0.0010
KEGG_INSULIN_SIGNALING_PATHWAY	97	2.308	0.0000	0.0007
REACTOME_PRC2_METHYLATES_HISTONES_AND_DNA	26	2.298	0.0000	0.0011
REACTOME_KINESINS	48	2.295	0.0000	0.0011
REACTOME_RHO_GTPASES_ACTIVATE_CIT	17	2.289	0.0000	0.0011
KEGG_ADHERENS_JUNCTION	61	2.287	0.0000	0.0006

NAME	SIZE	NES	NOM p-val	FDR q-val
REACTOME_RAC1_GTPASE_CYCLE	145	2.286	0.0000	0.0011
REACTOME_CDC42_GTPASE_CYCLE	115	2.284	0.0000	0.0011
REACTOME_RAC3_GTPASE_CYCLE	75	2.282	0.0000	0.0011
REACTOME_SUMOYLATION_OF_RNA_BINDING_PROTEINS	39	2.281	0.0000	0.0011
REACTOME_TRANSPORT_OF_MATURE_MRNAS_DERIVED_FROM_INTRONLES S_TRANSCRIPTS	36	2.281	0.0000	0.0011
KEGG_MAPK_SIGNALING_PATHWAY	182	2.280	0.0000	0.0007
REACTOME_CYTOKINE_SIGNALING_IN_IMMUNE_SYSTEM	442	2.278	0.0000	0.0011
REACTOME_TRANSCRIPTIONAL_REGULATION_BY_E2F6	27	2.276	0.0000	0.0011
REACTOME_TRANSCRIPTIONAL_REGULATION_BY_SMALL_RNAS	51	2.274	0.0000	0.0011
REACTOME_SIGNALING_BY_SCF_KIT	34	2.273	0.0000	0.0011
REACTOME_INTRACELLULAR_SIGNALING_BY_SECOND_MESSENGERS	219	2.272	0.0000	0.0011
REACTOME_PRE_NOTCH_EXPRESSION_AND_PROCESSING	50	2.269	0.0000	0.0011
REACTOME_RHOG_GTPASE_CYCLE	55	2.264	0.0000	0.0012
REACTOME_MRNA_SPLICING	154	2.263	0.0000	0.0012
KEGG_PANCREATIC_CANCER	52	2.261	0.0000	0.0007
REACTOME_INTERLEUKIN_3_INTERLEUKIN_5_AND_GM_CSF_SIGNALING	36	2.260	0.0000	0.0012
REACTOME_NUCLEAR_PORE_COMPLEX_NPC_DISASSEMBLY	28	2.254	0.0000	0.0012
REACTOME_CD209_DC_SIGN_SIGNALING	18	2.251	0.0000	0.0012
REACTOME_RHO_GTPASES_ACTIVATE_IQGAPS	25	2.251	0.0000	0.0012
KEGG_LEUKOCYTE_TRANSENDOTHELIAL_MIGRATION	74	2.245	0.0000	0.0007
REACTOME_TRANSPORT_OF_THE_SLBP_DEPENDANT_MATURE_MRNA	31	2.244	0.0000	0.0013
KEGG_GLIOMA	45	2.242	0.0000	0.0008
REACTOME_TRANSCRIPTIONAL_REGULATION_OF_WHITE_ADIPOCYTE_DIFFE RENTIATION	59	2.235	0.0000	0.0014
REACTOME_TOLL_LIKE_RECEPTOR_9_TLR9_CASCADE	65	2.231	0.0000	0.0014
REACTOME_MYD88_INDEPENDENT_TLR4_CASCADE	66	2.226	0.0000	0.0015
REACTOME_HOMOLOGY_DIRECTED_REPAIR	87	2.226	0.0000	0.0015
HALLMARK_HYPOXIA	145	2.220	0.0000	0.0001
REACTOME_CONDENSATION_OF_PROPHASE_CHROMOSOMES	25	2.210	0.0000	0.0017
REACTOME_SIGNALING_BY_INTERLEUKINS	299	2.210	0.0000	0.0017
REACTOME_INTERLEUKIN_4_AND_INTERLEUKIN_13_SIGNALING	66	2.207	0.0000	0.0018
REACTOME_CILIUM_ASSEMBLY	161	2.202	0.0000	0.0018
KEGG_INOSITOL_PHOSPHATE_METABOLISM	45	2.199	0.0000	0.0016
REACTOME_FLT3_SIGNALING	30	2.197	0.0000	0.0019
REACTOME_REGULATION_OF_TP53_ACTIVITY_THROUGH_ACETYLATION	23	2.197	0.0000	0.0019
REACTOME_DEUBIQUITINATION	203	2.195	0.0000	0.0019
REACTOME_DNA_REPAIR	216	2.194	0.0000	0.0019
REACTOME_RNA_POLYMERASE_II_TRANSCRIPTION_TERMINATION	54	2.192	0.0000	0.0019
REACTOME_RHOU_GTPASE_CYCLE	32	2.190	0.0000	0.0019
REACTOME_ORGANELLE_BIOGENESIS_AND_MAINTENANCE	225	2.183	0.0000	0.0020
REACTOME_ACTIVATION_OF_ATR_IN_RESPONSE_TO_REPLICATION_STRESS	30	2.178	0.0000	0.0021
REACTOME_S_PHASE	126	2.177	0.0000	0.0021
REACTOME_TRANSCRIPTIONAL_REGULATION_BY_RUNX1	143	2.171	0.0000	0.0022
REACTOME_RHO_GTPASES_ACTIVATE_PKNS	42	2.170	0.0000	0.0022
REACTOME_SIGNALING_BY_NUCLEAR_RECEPTORS	177	2.169	0.0000	0.0022
REACTOME_SUMOYLATION_OF_TRANSCRIPTION_COFACTORS	38	2.168	0.0000	0.0022
REACTOME_REGULATION_OF_HSF1_MEDIATED_HEAT_SHOCK_RESPONSE	62	2.150	0.0000	0.0026
REACTOME_HCMV_INFECTION	86	2.148	0.0000	0.0026
REACTOME_SIGNALING_BY_KIT_IN_DISEASE	18	2.147	0.0000	0.0026
REACTOME_PTEN_REGULATION	112	2.138	0.0000	0.0028
REACTOME_UB_SPECIFIC_PROCESSING_PROTEASES	139	2.136	0.0000	0.0029
REACTOME_FCGAMMA_RECEPTOR_FCGR_DEPENDENT_PHAGOCYTOSIS	58	2.135	0.0000	0.0029
REACTOME_PROCESSING_OF_DNA_DOUBLE_STRAND_BREAK_ENDS	55	2.133	0.0000	0.0029
REACTOME_HDR_THROUGH_HOMOLOGOUS_RECOMBINATION_HRR	52	2.125	0.0000	0.0031
REACTOME_INTERACTIONS_OF_REV_WITH_HOST_CELLULAR_PROTEINS	31	2.124	0.0000	0.0031

NAME	SIZE	NES	NOM p-val	FDR q-val
REACTOME_PARASITE_INFECTION	40	2.121	0.0000	0.0032
REACTOME_RUNX1_REGULATES_GENES_INVOLVED_IN_MEGAKARYOCYTE_DIFFERENTIATION_AND_PLATELET_FUNCTION	41	2.121	0.0000	0.0032
REACTOME_INTERLEUKIN_12_FAMILY_SIGNALING	45	2.117	0.0000	0.0033
REACTOME_APOPTOSIS	136	2.117	0.0000	0.0033
REACTOME_SIGNALING_BY_ERBB2	44	2.117	0.0000	0.0032
REACTOME_HDR_THROUGH_SINGLE_STRAND_ANNEALING_SSA	28	2.116	0.0000	0.0032
REACTOME_TRANSCRIPTIONAL_REGULATION_BY_MECP2	47	2.115	0.0000	0.0032
REACTOME_SIGNALING_BY_EGFR	34	2.114	0.0000	0.0032
REACTOME_SIGNALING_BY_FLT3_ITD_AND_TKD_MUTANTS	15	2.111	0.0000	0.0033
REACTOME_ANTIVIRAL_MECHANISM_BY_IFN_STIMULATED_GENES	65	2.109	0.0000	0.0033
REACTOME_CELL_DEATH_SIGNALLING_VIA_NRAGE_NRIF_AND_NADE	53	2.107	0.0000	0.0034
REACTOME_DOWNSTREAM_SIGNAL_TRANSDUCTION	23	2.102	0.0000	0.0034
KEGG_PATHWAYS_IN_CANCER	214	2.101	0.0000	0.0039
REACTOME_EXPORT_OF_VIRAL_RIBONUCLEOPROTEINS_FROM_NUCLEUS	28	2.100	0.0000	0.0035
REACTOME_HOMOLOGOUS_DNA_PAIRING_AND_STRAND_EXCHANGE	33	2.098	0.0000	0.0035
REACTOME_RHOV_GTPASE_CYCLE	34	2.098	0.0000	0.0035
REACTOME_REGULATION_OF_LIPID_METABOLISM_BY_PPARALPHA	88	2.090	0.0000	0.0037
REACTOME_DNA_REPLICATION	117	2.087	0.0000	0.0037
REACTOME_PROGRAMMED_CELL_DEATH	153	2.083	0.0000	0.0039
REACTOME_HATS_ACETYLATE_HISTONES	74	2.081	0.0000	0.0039
REACTOME_CD28_DEPENDENT_PI3K_AKT_SIGNALING	16	2.071	0.0000	0.0042
REACTOME_SIGNALING_BY_MODERATE_KINASE_ACTIVITY_BRAF_MUTANTS	31	2.070	0.0000	0.0042
REACTOME_GOLGI_TO_ER_RETROGRADE_TRANSPORT	106	2.067	0.0000	0.0042
REACTOME_REGULATION_OF_TP53_ACTIVITY_THROUGH_PHOSPHORYLATION	72	2.066	0.0000	0.0042
HALLMARK_ANDROGEN_RESPONSE	66	2.064	0.0000	0.0013
REACTOME_SIGNALING_BY_RECEPTOR_TYROSINE_KINASES	340	2.061	0.0000	0.0044
REACTOME_HEME_SIGNALING	38	2.058	0.0000	0.0045
REACTOME_SIGNALING_BY_PDGF	40	2.052	0.0000	0.0047
REACTOME_RHO_GTPASES_ACTIVATE_ROCKS	18	2.046	0.0000	0.0049
REACTOME_TRANSCRIPTIONAL_REGULATION_OF_GRANULOPOIESIS	33	2.042	0.0000	0.0050
KEGG_SPLICEOSOME	106	2.038	0.0000	0.0063
REACTOME_SIGNALING_BY_TGFB_FAMILY_MEMBERS	73	2.022	0.0000	0.0056
REACTOME_SIGNALING_BY_NOTCH	138	2.018	0.0000	0.0058
HALLMARK_COMPLEMENT	127	2.017	0.0000	0.0019
REACTOME_RHOJ_GTPASE_CYCLE	46	2.006	0.0000	0.0063
REACTOME_DISEASES_OF_PROGRAMMED_CELL_DEATH	50	1.999	0.0000	0.0065
HALLMARK_UV_RESPONSE_DN	102	1.996	0.0000	0.0019
REACTOME_ERCC6_CSB_AND_EHMT2_G9A_POSITIVELY_REGULATE_RRNA_EXPRESSION	26	1.995	0.0000	0.0066
KEGG_PHOSPHATIDYLINOSITOL_SIGNALING_SYSTEM	60	1.992	0.0000	0.0072
REACTOME_FC_EPSILON_RECEPTOR_FCERI_SIGNALING	98	1.987	0.0000	0.0069
REACTOME_RHOBTB_GTPASE_CYCLE	29	1.987	0.0000	0.0069
REACTOME_HEMOSTASIS	392	1.984	0.0000	0.0070
REACTOME_NEGATIVE_REGULATION_OF_THE_PI3K_AKT_NETWORK	73	1.974	0.0000	0.0075
KEGG_ENDOCYTOSIS	129	1.971	0.0000	0.0079
REACTOME_MAPK_FAMILY_SIGNALING_CASCADES	228	1.957	0.0000	0.0083
KEGG_FOCAL_ADHESION	138	1.952	0.0000	0.0084
KEGG_REGULATION_OF_ACTIN_CYTOSKELETON	149	1.924	0.0000	0.0098
REACTOME_INTRA_GOLGI_AND_RETROGRADE_GOLGI_TO_ER_TRAFFIC	155	1.911	0.0000	0.0109
REACTOME_CHROMOSOME_MAINTENANCE	85	1.907	0.0000	0.0113
REACTOME_MEMBRANE_TRAFFICKING	450	1.905	0.0000	0.0114
REACTOME_VESICLE_MEDIATED_TRANSPORT	468	1.843	0.0000	0.0169
REACTOME_NEUTROPHIL_DEGRANULATION	284	1.793	0.0000	0.0226
REACTOME_PHOSPHOLIPID_METABOLISM	154	1.953	0.0010	0.0085

NAME	SIZE	NES	NOM p-val	FDR q-val
REACTOME_DNA_DOUBLE_STRAND_BREAK_REPAIR	109	2.185	0.0010	0.0020
HALLMARK_UV_RESPONSE_UP	103	1.892	0.0010	0.0049
KEGG_JAK_STAT_SIGNALING_PATHWAY	94	2.009	0.0010	0.0068
REACTOME_TOLL_LIKE_RECEPTOR_CASCADES	99	1.922	0.0010	0.0102
REACTOME_DNA_REPLICATION_PRE_INITIATION	87	1.941	0.0010	0.0091
REACTOME_COPI_DEPENDENT_GOLGI_TO_ER_RETROGRADE_TRAFFIC	78	1.994	0.0010	0.0067
REACTOME_ACTIVATION_OF_ANTERIOR_HOX_GENES_IN_HINDBRAIN_DEVELOPMENT_DURING_EARLY_EMBRYOGENESIS	58	2.030	0.0010	0.0054
REACTOME_SENESCENCE_ASSOCIATED_SECRETORY_PHENOTYPE_SASP	54	2.135	0.0010	0.0028
REACTOME_MEIOSIS	59	1.991	0.0010	0.0068
REACTOME_PI3K_AKT_SIGNALING_IN_CANCER	66	1.973	0.0010	0.0075
REACTOME_SEMAPHORIN_INTERACTIONS	49	2.040	0.0011	0.0051
KEGG_FC_EPSILON_RI_SIGNALING_PATHWAY	52	2.040	0.0011	0.0066
REACTOME_SUMOYLATION_OF_CHROMATIN_ORGANIZATION_PROTEINS	46	2.227	0.0011	0.0015
REACTOME_NUCLEAR_EVENTS_KINASE_AND_TRANSCRIPTION_FACTOR_ACTIVATION	41	2.270	0.0011	0.0011
REACTOME_FOXO_MEDIATED_TRANSCRIPTION	46	2.136	0.0011	0.0029
REACTOME_SYNTHESIS_OF_PIPS_AT_THE_PLASMA_MEMBRANE	45	2.350	0.0011	0.0007
KEGG_NON_SMALL_CELL_LUNG_CANCER	38	2.071	0.0011	0.0050
REACTOME_FORMATION_OF_THE_BETA_CATENIN_TCF_TRANSACTIVATING_COMPLEX	38	1.933	0.0011	0.0094
REACTOME_NR1H2_AND_NR1H3_MEDIATED_SIGNALING	35	2.113	0.0011	0.0032
REACTOME_NRAGE_SIGNALS_DEATH_THROUGH_JNK	38	2.077	0.0011	0.0040
REACTOME_CYCLIN_D_ASSOCIATED_EVENTS_IN_G1	40	2.036	0.0011	0.0052
REACTOME_SIGNALING_BY_FGFR3	32	2.013	0.0011	0.0059
REACTOME_VIRAL_MESSENGER_RNA_SYNTHESIS	34	1.975	0.0011	0.0075
REACTOME_TP53_REGULATES_TRANSCRIPTION_OF_CELL_CYCLE_GENES	37	2.281	0.0011	0.0011
KEGG_RIG_I_LIKE_RECEPTOR_SIGNALING_PATHWAY	35	1.981	0.0011	0.0076
REACTOME_GENE_AND_PROTEIN_EXPRESSION_BY_JAK_STAT_SIGNALING_AFTER_INTERLEUKIN_12_STIMULATION	31	2.042	0.0011	0.0050
REACTOME_REGULATION_OF_TP53_EXPRESSION_AND_DEGRADATION	29	2.113	0.0011	0.0032
REACTOME_APOPTOTIC_CLEAVAGE_OF_CELLULAR_PROTEINS	29	2.089	0.0011	0.0037
REACTOME_RESOLUTION_OF_D_LOOP_STRUCTURES	28	2.158	0.0011	0.0024
REACTOME_CD28_CO_STIMULATION	25	2.196	0.0011	0.0019
REACTOME_INTERLEUKIN_2_FAMILY_SIGNALING	29	2.003	0.0011	0.0063
REACTOME_NUCLEAR_IMPORT_OF_REV_PROTEIN	28	2.007	0.0011	0.0062
REACTOME_G1_S_SPECIFIC_TRANSCRIPTION	22	2.045	0.0012	0.0049
REACTOME_SIGNALING_BY_CSF3_G_CSF	25	1.996	0.0012	0.0066
REACTOME_DEADENYLATION_OF_MRNA	20	1.973	0.0012	0.0075
REACTOME_SIGNALING_BY_FLT3_FUSION_PROTEINS	19	2.236	0.0012	0.0014
REACTOME_SEMA4D_INDUCED_CELL_MIGRATION_AND_GROWTH_CONE_COLLAPSE	17	2.093	0.0012	0.0037
REACTOME_MICRORNA_MIRNA_BIOGENESIS	16	2.038	0.0012	0.0051
REACTOME_RAF_INDEPENDENT_MAPK1_3_ACTIVATION	15	1.936	0.0012	0.0094
HALLMARK_INFLAMMATORY_RESPONSE	128	1.783	0.0020	0.0127
REACTOME_INTERFERON_SIGNALING	115	1.867	0.0020	0.0146
KEGG_TIGHT_JUNCTION	90	1.797	0.0020	0.0205
REACTOME_SIGNALING_BY_VEGF	71	1.956	0.0020	0.0083
REACTOME_TOLL_LIKE_RECEPTOR_TLR1_TLR2_CASCADE	63	1.992	0.0021	0.0067
REACTOME_POSITIVE_EPIGENETIC_REGULATION_OF_RRNA_EXPRESSION	53	2.030	0.0021	0.0053
KEGG_RENAL_CELL_CARCINOMA	56	1.949	0.0021	0.0082
REACTOME_INTRINSIC_PATHWAY_FOR_APOPTOSIS	47	1.945	0.0021	0.0090
REACTOME_TRANSLOCATION_OF_SLC2A4_GLUT4_TO_THE_PLASMA_MEMBRANE	55	2.040	0.0021	0.0051
REACTOME_REGULATION_OF_PTEN_GENE_TRANSCRIPTION	48	2.229	0.0021	0.0015

NAME	SIZE	NES	NOM p-val	FDR q-val
REACTOME_TRANSCRIPTIONAL_ACTIVATION_OF_MITOCHONDRIAL_BIOGENESIS	40	1.959	0.0021	0.0082
REACTOME_INSULIN_RECEPTOR_SIGNALLING_CASCADE	39	1.935	0.0022	0.0093
REACTOME_APOPTOTIC_EXECUTION_PHASE	37	2.015	0.0022	0.0059
REACTOME_SIGNALING_BY_FGFR1	36	1.935	0.0022	0.0094
REACTOME_SUMOYLATION_OF_UBIQUITINYLATION_PROTEINS	32	2.053	0.0022	0.0047
REACTOME_NR1H3_NR1H2_REGULATE_GENE_EXPRESSION_LINKED_TO_CHOLESTEROL_TRANSPORT_AND_EFFLUX	29	2.115	0.0022	0.0032
REACTOME_TRANSCRIPTIONAL_REGULATION_BY_VENTX	27	2.206	0.0023	0.0018
REACTOME_DNA_STRAND_ELONGATION	28	2.104	0.0023	0.0034
REACTOME_FLT3_SIGNALING_IN_DISEASE	26	2.011	0.0023	0.0060
REACTOME_POSTMITOTIC_NUCLEAR_PORE_COMPLEX_NPC_REFORMATION	23	2.014	0.0023	0.0059
REACTOME_MAPK_TARGETS_NUCLEAR_EVENTS_MEDIATED_BY_MAP_KINASES	19	2.052	0.0024	0.0047
KEGG_THYROID_CANCER	20	1.971	0.0024	0.0077
REACTOME_SIGNALING_BY_ERBB2_IN_CANCER	22	2.089	0.0024	0.0037
REACTOME_G0_AND_EARLY_G1	20	2.121	0.0024	0.0032
REACTOME_DAP12_SIGNALING	18	2.046	0.0025	0.0049
REACTOME_SIGNALING_BY_PDGFR_IN_DISEASE	15	1.957	0.0025	0.0083
REACTOME_CLASS_I_MHC_MEDIATED_ANTIGEN_PROCESSING_PRESENTATION	268	1.637	0.0030	0.0535
REACTOME_ANTIGEN_PROCESSING_UBIQUITINATION_PROTEASOME_DEGRADATION	239	1.656	0.0030	0.0489
HALLMARK_EPITHELIAL_MESENCHYMAL_TRANSITION	121	1.778	0.0030	0.0126
REACTOME_MHC_CLASS_II_ANTIGEN_PRESENTATION	87	1.850	0.0030	0.0163
KEGG_OOCYTE_MEIOSIS	80	1.933	0.0031	0.0093
KEGG_NATURAL_KILLER_CELL_MEDIATED_CYTOTOXICITY	71	1.876	0.0031	0.0133
KEGG_GNRH_SIGNALING_PATHWAY	66	1.957	0.0031	0.0082
REACTOME_SIGNALING_BY_ERBB4	40	1.970	0.0033	0.0075
REACTOME_INTERLEUKIN_12_SIGNALING	38	1.972	0.0033	0.0075
KEGG_DNA_REPLICATION	29	2.000	0.0034	0.0071
KEGG_BLADDER_CANCER	30	2.035	0.0034	0.0062
REACTOME_TP53_REGULATES_TRANSCRIPTION_OF_CELL_DEATH_GENES	30	2.026	0.0034	0.0055
REACTOME_INTERACTIONS_OF_VPR_WITH_HOST_CELLULAR_PROTEINS	29	1.978	0.0034	0.0073
REACTOME_TRANSCRIPTIONAL_REGULATION_BY_THE_AP_2_TFAP2_FAMILY_OF_TRANSCRIPTION_FACTORS	26	2.070	0.0034	0.0042
REACTOME_SEMA4D_IN_SEMAPHORIN_SIGNALING	20	1.935	0.0036	0.0094
REACTOME_GROWTH_HORMONE_RECEPTOR_SIGNALING	16	2.037	0.0037	0.0051
REACTOME_ABERRANT_REGULATION_OF_MITOTIC_G1_S_TRANSITION_IN_CANCER_DUE_TO_RB1_DEFECTS	16	1.972	0.0037	0.0075
KEGG_CHEMOKINE_SIGNALING_PATHWAY	112	1.825	0.0040	0.0179
KEGG_UBIQUITIN_MEDIATED_PROTEOLYSIS	99	1.758	0.0040	0.0249
KEGG_FC_GAMMA_R_MEDIATED_PHAGOCYTOSIS	64	2.012	0.0042	0.0069
REACTOME_POTENTIAL_THERAPEUTICS_FOR_SARS	63	1.810	0.0042	0.0206
REACTOME_G_ALPHA_12_13_SIGNALLING_EVENTS	58	1.734	0.0042	0.0322
REACTOME_NEGATIVE_EPIGENETIC_REGULATION_OF_RRNA_EXPRESSION	53	1.863	0.0043	0.0150
REACTOME_CYTOSOLIC_SENSORS_OF_PATHOGEN_ASSOCIATED_DNA	45	1.821	0.0043	0.0192
REACTOME_COPI_INDEPENDENT_GOLGI_TO_ER_RETROGRADE_TRAFFIC	42	1.877	0.0043	0.0138
REACTOME_DISEASES_OF_MITOTIC_CELL_CYCLE	28	1.924	0.0045	0.0100
REACTOME_ASSOCIATION_OF_TRIC_CCT_WITH_TARGET_PROTEINS_DURING_BIOSYNTHESIS	28	1.897	0.0046	0.0121
REACTOME_DISEASES_OF_DNA_REPAIR	25	1.809	0.0046	0.0206
REACTOME_SUMOYLATION_OF_SUMOYLATION_PROTEINS	29	1.985	0.0046	0.0069
REACTOME_MAP2K_AND_MAPK_ACTIVATION	27	1.879	0.0046	0.0135
REACTOME_REGULATION_OF_GLUCOKINASE_BY_GLUCOKINASE_REGULATORY_PROTEIN	26	1.988	0.0047	0.0069

NAME	SIZE	NES	NOM p-val	FDR q-val
REACTOME_RESOLUTION_OF_D_LOOP_STRUCTURES_THROUGH_SYNTHESIS_DEPENDENT_STRAND_ANNEALING_SDSA	22	1.971	0.0048	0.0075
REACTOME_RAB_REGULATION_OF_TRAFFICKING	92	1.810	0.0051	0.0206
KEGG_MELANOMA	49	1.801	0.0053	0.0205
REACTOME_HSP90_CHAPERONE_CYCLE_FOR_STEROID_HORMONE_RECEPTORS_SHR_IN_THE_PRESENCE_OF_LIGAND	47	1.867	0.0053	0.0146
REACTOME_INTRAFLAGELLAR_TRANSPORT	48	1.823	0.0054	0.0192
REACTOME_SIGNALING_BY_NOTCH1_PEST_DOMAIN_MUTANTS_IN_CANCER	41	1.871	0.0054	0.0144
REACTOME_NEGATIVE_REGULATION_OF_MAPK_PATHWAY	34	1.940	0.0055	0.0091
REACTOME_RET_SIGNALING	31	1.881	0.0056	0.0134
REACTOME_RAF_ACTIVATION	28	1.864	0.0057	0.0149
REACTOME_SIGNALING_BY_NTRK2_TRKB	23	1.949	0.0058	0.0087
REACTOME_BMAL1_CLOCK_NPAS2_ACTIVATES_CIRCADIAN_GENE_EXPRESSION	24	1.960	0.0059	0.0082
REACTOME_INTERLEUKIN_7_SIGNALING	18	1.942	0.0060	0.0091
KEGG_WNT_SIGNALING_PATHWAY	97	1.794	0.0060	0.0205
REACTOME_CELL_SURFACE_INTERACTIONS_AT_THE_VASCULAR_WALL	84	1.746	0.0061	0.0301
REACTOME_HIV_LIFE_CYCLE	107	1.679	0.0061	0.0435
REACTOME_SARS_COV_INFECTIONS	111	1.722	0.0061	0.0344
REACTOME_SYNTHESIS_OF_DNA	92	1.809	0.0061	0.0206
REACTOME_SIGNALING_BY_THE_B_CELL_RECEPTOR_BCR	79	1.736	0.0062	0.0318
REACTOME_TRANSCRIPTIONAL_REGULATION_BY_RUNX3	78	1.801	0.0062	0.0215
REACTOME_INTERLEUKIN_20_FAMILY_SIGNALING	17	1.915	0.0062	0.0107
REACTOME_SIGNALING_BY_EGFR_IN_CANCER	18	1.930	0.0062	0.0095
REACTOME_ACTIVATION_OF_NMDA_RECEPTORS_AND_POSTSYNAPTIC_EVENTS	63	1.816	0.0062	0.0199
REACTOME_TRNA_PROCESSING_IN_THE_NUCLEUS	46	1.882	0.0063	0.0134
REACTOME_COSTIMULATION_BY_THE_CD28_FAMILY	41	1.930	0.0064	0.0096
KEGG_EPITHELIAL_CELL_SIGNALING_IN_HELICOBACTER_PYLORI_INFECTION	42	1.826	0.0065	0.0181
KEGG_VASOPRESSIN_REGULATED_WATER_REABSORPTION	35	1.827	0.0068	0.0184
HALLMARK_MTORC1_SIGNALING	140	1.655	0.0070	0.0287
REACTOME_INACTIVATION_OF_CSF3_G_CSF_SIGNALING	20	1.830	0.0070	0.0184
REACTOME_PLATELET_ACTIVATION_SIGNALING_AND_AGGREGATION	162	1.610	0.0070	0.0610
HALLMARK_KRAS_SIGNALING_UP	118	1.736	0.0070	0.0159
REACTOME_ASSEMBLY_OF_THE_ORC_COMPLEX_AT_THE_ORIGIN_OF_REPLICATION	21	1.914	0.0071	0.0107
REACTOME_CLATHRIN_MEDIATED_ENDOCYTOSIS	99	1.681	0.0071	0.0434
REACTOME_SIRT1_NEGATIVELY_REGULATES_RRNA_EXPRESSION	21	1.890	0.0071	0.0126
REACTOME_EXTRA_NUCLEAR_ESTROGEN_SIGNALING	51	1.794	0.0074	0.0225
REACTOME_PROCESSING_OF_INTRONLESS_PRE_MRNAS	16	1.932	0.0074	0.0095
REACTOME_TP53_REGULATES_TRANSCRIPTION_OF_ADDITIONAL_CELL_CYCLE_GENES_WHOSE_EXACT_ROLE_IN_THE_P53_PATHWAY_REMAIN_UNCERTAIN	16	1.836	0.0074	0.0179
REACTOME_SIGNALING_BY_FGFR1_IN_DISEASE	31	1.871	0.0079	0.0143
REACTOME_NGF_STIMULATED_TRANSCRIPTION	28	1.890	0.0080	0.0126
REACTOME_CELL_CELL_COMMUNICATION	86	1.702	0.0081	0.0387
REACTOME_FCERI_MEDIATED_CA_2_MOBILIZATION	21	1.855	0.0082	0.0158
REACTOME_RECOGNITION_OF_DNA_DAMAGE_BY_PCNA_CONTAINING_REPLICATION_COMPLEX	24	1.818	0.0082	0.0196
REACTOME_SIGNALING_BY_FGFR	62	1.750	0.0082	0.0294
REACTOME_DOWNSTREAM_SIGNALING_OF_ACTIVATED_FGFR3	20	1.874	0.0084	0.0140
REACTOME_MEIOTIC_SYNAPSIS	39	1.760	0.0087	0.0277
HALLMARK_ESTROGEN_RESPONSE_LATE	132	1.654	0.0090	0.0277
HALLMARK_ESTROGEN_RESPONSE_EARLY	143	1.670	0.0091	0.0267
REACTOME_AUTOPHAGY	116	1.662	0.0091	0.0477
REACTOME_DOWNREGULATION_OF_TGF_BETA_RECEPTOR_SIGNALING	23	1.848	0.0096	0.0164

NAME	SIZE	NES	NOM p-val	FDR q-val
REACTOME_RESOLUTION_OF_AP_SITES_VIA_THE_MULTIPLE_NUCLEOTIDE_PATCH_REPLACEMENT_PATHWAY	19	1.785	0.0096	0.0237
REACTOME_DNA_DAMAGE_BYPASS	37	1.830	0.0097	0.0184
REACTOME_DEPOSITION_OF_NEW_CENPA_CONTAINING_NUCLEOSOMES_AT_THE_CENTROMERE	34	1.848	0.0099	0.0164
REACTOME_RAB_GEFS_EXCHANGE_GTP_FOR_GDP_ON_RABS	69	1.778	0.0101	0.0247
KEGG_SNARE_INTERACTIONS_IN_VESICULAR_TRANSPORT	26	1.812	0.0103	0.0194
HALLMARK_IL6_JAK_STAT3_SIGNALING	50	1.839	0.0107	0.0080
REACTOME_CONSTITUTIVE_SIGNALING_BY_AKT1_E17K_IN_CANCER	18	1.803	0.0111	0.0212
REACTOME_NUCLEOTIDE_EXCISION_REPAIR	79	1.684	0.0111	0.0427
REACTOME_SIGNALING_BY_NOTCH1	49	1.845	0.0117	0.0167
REACTOME_SIGNALING_BY_PTK6	43	1.829	0.0118	0.0184
REACTOME_CYCLIN_A_B1_B2_ASSOCIATED_EVENTS_DURING_G2_M_TRANSITION	21	1.862	0.0120	0.0150
REACTOME_DNA_DOUBLE_STRAND_BREAK_RESPONSE	38	1.781	0.0120	0.0244
REACTOME_ER_TO_GOLGI_ANTEROGRADE_TRANSPORT	114	1.642	0.0121	0.0526
REACTOME_REGULATION_OF_SIGNALING_BY_CBL	17	1.806	0.0123	0.0208
REACTOME_CONSTITUTIVE_SIGNALING_BY_LIGAND_RESPONSIVE_EGFR_CANCER_VARIANTS	17	1.806	0.0123	0.0209
REACTOME_REPRODUCTION	69	1.739	0.0124	0.0314
HALLMARK_CHOLESTEROL_HOMEOSTASIS	49	1.790	0.0126	0.0127
REACTOME_TRANS_GOLGI_NETWORK_VESICLE_BUDDING	51	1.679	0.0127	0.0436
REACTOME_RMTS_METHYLATE_HISTONE_ARGININES	31	1.721	0.0134	0.0344
REACTOME_TGF_BETA_RECEPTOR_SIGNALING_ACTIVATES_SMADS	25	1.827	0.0137	0.0186
KEGG_GAP_JUNCTION	66	1.697	0.0143	0.0371
KEGG_LYSINE_DEGRADATION	33	1.793	0.0143	0.0202
REACTOME_DAP12_INTERACTIONS	21	1.850	0.0144	0.0163
REACTOME_TELOMERE_MAINTENANCE	62	1.730	0.0146	0.0330
REACTOME_MEIOTIC_RECOMBINATION	32	1.742	0.0147	0.0308
REACTOME_NEGATIVE_REGULATORS_OF_DDX58_IFIH1_SIGNALING	28	1.693	0.0148	0.0405
REACTOME_DNA_METHYLATION	18	1.725	0.0156	0.0339
KEGG_SMALL_CELL_LUNG_CANCER	62	1.719	0.0156	0.0327
REACTOME_SMAD2_SMAD3_SMAD4_HETEROTRIMER_REGULATES_TRANSCRIPTION	25	1.742	0.0160	0.0309
REACTOME_B_WICH_COMPLEX_POSITIVELY_REGULATES_RRNA_EXPRESSION	42	1.765	0.0162	0.0269
REACTOME_GPVI_MEDIATED_ACTIVATION_CASCADE	22	1.832	0.0165	0.0183
REACTOME_SIGNALING_BY_TYPE_1_INSULIN_LIKE_GROWTH_FACTOR_1_RECEPTOR_IGF1R	38	1.758	0.0166	0.0279
REACTOME_RHOBTB1_GTPASE_CYCLE	19	1.768	0.0166	0.0264
HALLMARK_ANGIOGENESIS	18	1.738	0.0169	0.0165
REACTOME_NEPHRIN_FAMILY_INTERACTIONS	17	1.722	0.0170	0.0344
REACTOME_FORMATION_OF_TUBULIN_FOLDING_INTERMEDIATES_BY_CCT_TRIC	20	1.700	0.0174	0.0391
KEGG_VEGF_SIGNALING_PATHWAY	53	1.656	0.0178	0.0465
REACTOME_RHOBTB2_GTPASE_CYCLE	19	1.780	0.0180	0.0245
REACTOME_HSF1_DEPENDENT_TRANSACTIVATION	27	1.716	0.0181	0.0356
REACTOME_SIGNALING_BY_FGFR_IN_DISEASE	47	1.667	0.0183	0.0467
REACTOME_NCAM_SIGNALING_FOR_NEURITE_OUT_GROWTH	42	1.706	0.0194	0.0380
REACTOME_ANTIGEN_ACTIVATES_B_CELL_RECEPTOR_BCR_LEADING_TO_GENERATION_OF_SECOND_MESSENGERS	16	1.732	0.0194	0.0326
REACTOME_REGULATION_OF_CHOLESTEROL_BIOSYNTHESIS_BY_SREBP_SREBF	44	1.695	0.0195	0.0403
REACTOME_AMYLOID_FIBER_FORMATION	37	1.723	0.0196	0.0342
REACTOME_RND1_GTPASE_CYCLE	36	1.703	0.0197	0.0385
REACTOME_CA2_PATHWAY	47	1.618	0.0203	0.0586
REACTOME_DUAL_INCISION_IN_TC_NER	44	1.704	0.0205	0.0384
REACTOME_HCMV_LATE_EVENTS	52	1.665	0.0211	0.0471

NAME	SIZE	NES	NOM p-val	FDR q-val
REACTOME_SIGNALING_BY_FGFR4	32	1.668	0.0213	0.0464
REACTOME_INTERLEUKIN_17_SIGNALING	50	1.659	0.0216	0.0485
KEGG_NOD_LIKE_RECEPTOR_SIGNALING_PATHWAY	42	1.681	0.0218	0.0396
REACTOME_THE_ROLE_OF_GTSE1_IN_G2_M_PROGRESSION_AFTER_G2_CHECKPOINT	62	1.660	0.0218	0.0483
REACTOME_SIGNALING_BY_MET	60	1.625	0.0219	0.0566
REACTOME_TRANSCRIPTION_COUPLED_NUCLEOTIDE_EXCISION_REPAIR_TC_NER	51	1.640	0.0231	0.0528
REACTOME_CTLA4_INHIBITORY_SIGNALING	16	1.700	0.0243	0.0390
REACTOME_IRS_MEDIATED_SIGNALLING	35	1.639	0.0246	0.0532
KEGG_VIRAL_MYOCARDITIS	32	1.687	0.0248	0.0391
REACTOME_CASPASE_ACTIVATION_VIA_EXTRINSIC_APOPTOTIC_SIGNALLING_PATHWAY	16	1.677	0.0248	0.0439
REACTOME_GOLGI_ASSOCIATED_VESICLE_BIOGENESIS	41	1.695	0.0249	0.0403
REACTOME_RNA_POLYMERASE_I_TRANSCRIPTION	53	1.635	0.0253	0.0539
REACTOME_DUAL_INCISION_IN_GG_NER	29	1.638	0.0255	0.0532
REACTOME_ATTENUATION_PHASE	22	1.725	0.0258	0.0338
HALLMARK_HEME_METABOLISM	145	1.499	0.0261	0.0676
KEGG_APOPTOSIS	63	1.601	0.0270	0.0661
REACTOME_SIGNALING_BY_HEDGEHOG	117	1.498	0.0272	0.1047
REACTOME_TNF_SIGNALING	35	1.655	0.0274	0.0491
REACTOME_BASE_EXCISION_REPAIR	43	1.629	0.0275	0.0557
REACTOME_LAGGING_STRAND_SYNTHESIS	16	1.694	0.0277	0.0405
REACTOME_PROTEIN_FOLDING	71	1.597	0.0279	0.0653
REACTOME_MITOCHONDRIAL_BIOGENESIS	64	1.577	0.0281	0.0726
REACTOME_SPHINGOLIPID_DE_NOVO_BIOSYNTHESIS	27	1.676	0.0283	0.0441
REACTOME_DEADENYLATION_DEPENDENT_MRNA_DECAY	40	1.641	0.0285	0.0528
HALLMARK_ALLOGRAFT_REJECTION	114	1.529	0.0292	0.0595
REACTOME_INTERLEUKIN_RECEPTOR_SHC_SIGNALING	19	1.628	0.0298	0.0558
REACTOME_COPI_MEDIATED_ANTEROGRADE_TRANSPORT	76	1.609	0.0299	0.0614
HALLMARK_MYOGENESIS	132	1.528	0.0302	0.0578
REACTOME_INTRA_GOLGI_TRAFFIC	28	1.635	0.0303	0.0538
KEGG_ALDOSTERONE_REGULATED_SODIUM_REABSORPTION	28	1.581	0.0304	0.0732
REACTOME_INTERFERON_ALPHA_BETA_SIGNALING	24	1.684	0.0310	0.0427
REACTOME_NUCLEOTIDE_BINDING_DOMAIN_LEUCINE_RICH_REPEAT_CONTAINING_RECEPTOR_NLR_SIGNALING_PATHWAYS	46	1.599	0.0311	0.0647
REACTOME_RECYCLING_PATHWAY_OF_L1	36	1.635	0.0324	0.0537
REACTOME_PCNA_DEPENDENT_LONG_PATCH_BASE_EXCISION_REPAIR	15	1.613	0.0331	0.0601
REACTOME_CONSTITUTIVE_SIGNALING_BY_ABERRANT_PI3K_IN_CANCER	47	1.658	0.0333	0.0486
REACTOME_SIGNALING_BY_FGFR2	52	1.628	0.0334	0.0558
REACTOME_INTERLEUKIN_10_SIGNALING	25	1.618	0.0335	0.0585
REACTOME_SIGNALING_BY_CYTOSOLIC_FGFR1_FUSION_MUTANTS	15	1.656	0.0343	0.0490
REACTOME_INTERLEUKIN_1_FAMILY_SIGNALING	97	1.495	0.0355	0.1056
REACTOME_UPTAKE_AND_ACTIONS_OF_BACTERIAL_TOXINS	21	1.622	0.0355	0.0575
KEGG_HOMOLOGOUS_RECOMBINATION	20	1.594	0.0367	0.0678
REACTOME_TBC_RABGAPS	32	1.645	0.0367	0.0522
REACTOME_RHOH_GTPASE_CYCLE	31	1.641	0.0368	0.0528
REACTOME_HIV_INFECTION	169	1.472	0.0371	0.1162
HALLMARK_INTERFERON_ALPHA_RESPONSE	56	1.579	0.0372	0.0448
REACTOME_IRE1ALPHA_ACTIVATES_CHAPERONES	37	1.619	0.0374	0.0583
REACTOME_SEALING_OF_THE_NUCLEAR_ENVELOPE_NE_BY_ESCRT_III	23	1.644	0.0380	0.0521
REACTOME_GLOBAL_GENOME_NUCLEOTIDE_EXCISION_REPAIR_GG_NER	61	1.587	0.0381	0.0689
REACTOME_OVARIAN_TUMOR_DOMAIN_PROTEASES	33	1.651	0.0382	0.0501
REACTOME_SNRNP_ASSEMBLY	41	1.585	0.0386	0.0697
REACTOME_HEDGEHOG_OFF_STATE	89	1.571	0.0398	0.0748
REACTOME_SHC1_EVENTS_IN_ERBB2_SIGNALING	17	1.602	0.0404	0.0640

NAME	SIZE	NES	NOM p-val	FDR q-val
REACTOME_DOWNSTREAM_SIGNALING_OF_ACTIVATED_FGFR4	21	1.601	0.0418	0.0642
REACTOME_CELL_CELL_JUNCTION_ORGANIZATION	40	1.597	0.0423	0.0653
REACTOME_NOTCH_HLH_TRANSCRIPTION_PATHWAY	24	1.621	0.0426	0.0577
REACTOME_DOWNSTREAM_SIGNALING_OF_ACTIVATED_FGFR2	22	1.550	0.0437	0.0829
REACTOME_ACTIVATED_PKN1_STIMULATES_TRANSCRIPTION_OF_AR_ANDROGEN_RECEPTOR_REGULATED_GENES_KLK2_AND_KLK3	19	1.581	0.0438	0.0714
REACTOME_MAPK6_MAPK4_SIGNALING	74	1.499	0.0439	0.1044
REACTOME_INTERLEUKIN_37_SIGNALING	15	1.598	0.0448	0.0652
REACTOME_RND2_GTPASE_CYCLE	38	1.543	0.0457	0.0852
REACTOME_SIGNALING_BY_INSULIN_RECEPTOR	54	1.547	0.0459	0.0840
REACTOME_INOSITOL_PHOSPHATE_METABOLISM	40	1.537	0.0470	0.0873
REACTOME_SYNTHESIS_OF_IP3_AND_IP4_IN_THE_CYTOSOL	23	1.579	0.0473	0.0722
REACTOME_RIPK1_MEDIATED_REGULATED_NECROSIS	23	1.551	0.0475	0.0828
REACTOME_BETA_CATENIN_INDEPENDENT_WNT_SIGNALING	110	1.455	0.0475	0.1258
REACTOME_ACTIVATION_OF_GENE_EXPRESSION_BY_SREBF_SREBP	33	1.564	0.0491	0.0778
KEGG_MTOR_SIGNALING_PATHWAY	43	1.531	0.0491	0.0927
REACTOME_RND3_GTPASE_CYCLE	38	1.522	0.0498	0.0942

NAME	SIZE	NES	NOM p-val	FDR q-val
KEGG_RIBOSOME	81	-9.949	0.0000	0.0000
REACTOME_EUKARYOTIC_TRANSLATION_ELONGATION	88	-9.915	0.0000	0.0000
REACTOME_SRP_DEPENDENT_COTRANSLATIONAL_PROTEIN_TARGETING_TO_	100	-8.410	0.0000	0.0000
REACTOME_SELENOAMINO_ACID_METABOLISM	101	-7.955	0.0000	0.0000
REACTOME_RESPONSE_OF_EIF2AK4_GCN2_TO_AMINO_ACID_DEFICIENCY	94	-7.420	0.0000	0.0000
REACTOME_EUKARYOTIC_TRANSLATION_INITIATION	107	-7.397	0.0000	0.0000
REACTOME_NONSENSE_MEDIATED_DECAY_NMD	103	-6.132	0.0000	0.0000
REACTOME_INFLUENZA_INFECTION	137	-5.664	0.0000	0.0000
REACTOME_CELLULAR_RESPONSE_TO_STARVATION	135	-5.261	0.0000	0.0000
KEGG_NEUROACTIVE_LIGAND_RECEPTOR_INTERACTION	141	-5.142	0.0000	0.0000
REACTOME_GPCR_LIGAND_BINDING	227	-4.997	0.0000	0.0000
REACTOME_RRNA_PROCESSING	173	-4.993	0.0000	0.0000
REACTOME_REGULATION_OF_EXPRESSION_OF_SLITS_AND_ROBOS	150	-4.746	0.0000	0.0000
REACTOME_ACTIVATION_OF_THE_MRNA_UPON_BINDING_OF_THE_CAP_BIN	52	-4.673	0.0000	0.0000
KEGG_OLFACTORY_TRANSDUCTION	57	-4.112	0.0000	0.0000
REACTOME_RESPIRATORY_ELECTRON_TRANSPORT_ATP_SYNTHESIS_BY_CHEI	83	-3.990	0.0000	0.0000
REACTOME_CLASS_A_1_RHODOPSIN_LIKE_RECEPTORS	153	-3.965	0.0000	0.0000
REACTOME_SIGNALING_BY_ROBO_RECEPTORS	181	-3.864	0.0000	0.0000
REACTOME_MITOCHONDRIAL_TRANSLATION	52	-3.753	0.0000	0.0000
REACTOME_RESPIRATORY_ELECTRON_TRANSPORT	68	-3.704	0.0000	0.0000
REACTOME_THE_CITRIC_ACID_TCA_CYCLE_AND_RESPIRATORY_ELECTRON_TI	119	-3.633	0.0000	0.0000
KEGG_OXIDATIVE_PHOSPHORYLATION	78	-3.486	0.0000	0.0000
REACTOME_COMPLEX_I_BIOGENESIS	41	-3.473	0.0000	0.0000
KEGG_PARKINSONS_DISEASE	80	-3.378	0.0000	0.0000
REACTOME_CLASS_C_3_METABOTROPIC_GLUTAMATE_PHEROMONE_RECEPT	26	-3.056	0.0000	0.0000
REACTOME_PEPTIDE_LIGAND_BINDING_RECEPTORS	85	-2.989	0.0000	0.0000
KEGG_ARACHIDONIC_ACID_METABOLISM	35	-2.920	0.0000	0.0005
HALLMARK_OXIDATIVE_PHOSPHORYLATION	140	-2.807	0.0000	0.0000
KEGG_STEROID_HORMONE_BIOSYNTHESIS	27	-2.740	0.0000	0.0004
REACTOME_ARACHIDONIC_ACID_METABOLISM	37	-2.525	0.0000	0.0007
REACTOME_BIOLOGICAL_OXIDATIONS	129	-2.482	0.0000	0.0007
KEGG_TYROSINE_METABOLISM	27	-2.456	0.0000	0.0021
KEGG_RETINOL_METABOLISM	33	-2.386	0.0000	0.0025
REACTOME_PHASE_I_FUNCTIONALIZATION_OF_COMPOUNDS	69	-2.383	0.0000	0.0016
REACTOME_FATTY_ACID_METABOLISM	104	-2.292	0.0000	0.0031
REACTOME_KERATINIZATION	49	-2.223	0.0000	0.0049
KEGG_DRUG_METABOLISM_CYTOCHROME_P450	32	-2.149	0.0000	0.0065
KEGG_ALZHEIMERS_DISEASE	112	-2.024	0.0000	0.0141
KEGG_METABOLISM_OF_XENOBIOTICS_BY_CYTOCHROME_P450	33	-1.968	0.0000	0.0195

NAME	SIZE	NES	NOM p-val	FDR q-val
KEGG_PORPHYRIN_AND_CHLOROPHYLL_METABOLISM	22	-1.954	0.0000	0.0191
KEGG_VALINE_LEUCINE_AND_ISOLEUCINE_DEGRADATION	31	-1.930	0.0000	0.0192
KEGG_PROPANOATE_METABOLISM	19	-1.901	0.0000	0.0212
REACTOME_DISEASES_OF_METABOLISM	157	-1.649	0.0000	0.0910
REACTOME_PROTEIN_LOCALIZATION	110	-1.604	0.0000	0.1111
KEGG_HUNTINGTONS_DISEASE	125	-1.521	0.0000	0.1165
REACTOME_G_ALPHA_Q_SIGNALLING_EVENTS	128	-1.507	0.0000	0.1492
REACTOME_VISUAL_PHOTOTRANSDUCTION	57	-1.490	0.0000	0.1573
REACTOME_FORMATION_OF_FIBRIN_CLOT_CLOTTING_CASCADE	17	-1.996	0.0062	0.0198
REACTOME_VOLTAGE_GATED_POTASSIUM_CHANNELS	29	-1.752	0.0095	0.0627
REACTOME_AMINE_LIGAND_BINDING_RECEPTORS	19	-1.942	0.0113	0.0258
REACTOME_FORMATION_OF_THE_CORNIFIED_ENVELOPE	39	-1.542	0.0118	0.1411
REACTOME_METABOLIC_DISORDERS_OF_BIOLOGICAL_OXIDATION_ENZYMES	20	-1.845	0.0123	0.0411
REACTOME_CYTOCHROME_P450_ARRANGED_BY_SUBSTRATE_TYPE	42	-1.861	0.0137	0.0393
KEGG_PEROXISOME	50	-1.624	0.0164	0.0749
REACTOME_CRISTAE_FORMATION	19	-1.727	0.0180	0.0700
KEGG_GLYCOSAMINOGLYCAN_BIOSYNTHESIS_HEPARAN_SULFATE	16	-1.739	0.0266	0.0445
REACTOME_TRANSPORT_OF_VITAMINS_NUCLEOSIDES_AND_RELATED_MOLE	31	-1.649	0.0275	0.0931
REACTOME_PEROXISOMAL_LIPID_METABOLISM	16	-1.655	0.0284	0.0941
REACTOME_ADORA2B_MEDIATED_ANTI_INFLAMMATORY_CYTOKINES_PROD	69	-1.772	0.0313	0.0577
REACTOME_METABOLISM_OF_STEROID_HORMONES	18	-1.602	0.0343	0.1101
REACTOME_BRANCHED_CHAIN_AMINO_ACID_CATABOLISM	17	-1.672	0.0372	0.0899
KEGG_GLYCOLYSIS_GLUCONEOGENESIS	37	-1.502	0.0405	0.1167
REACTOME_WNT_LIGAND_BIOGENESIS_AND_TRAFFICKING	15	-1.542	0.0433	0.1442
REACTOME_THE_PHOTOTRANSDUCTION_CASCADE	23	-1.453	0.0455	0.1806
REACTOME_G_ALPHA_S_SIGNALLING_EVENTS	79	-1.263	0.0455	0.3207
REACTOME_SULFUR_AMINO_ACID_METABOLISM	20	-1.526	0.0483	0.1429
REACTOME_XENOBIOTICS	18	-1.512	0.0492	0.1486

Supplementary Table S4.Gene sets related to oxidative phosphorylation negatively associated with NONO IV Quartile compared to NONO I Quartile in CoMMpass database. Core Enrichment genes are shown in bold

NAME	SIZE	NES	NOM p-val	gene list
KEGG_OXIDATIVE_PHOSPHORYLATION	78	-3.49	0	ATP6V0A1, ATP6V0C, ATP6V1C2, NDUFS2, **COX6B2, ATP6V0B, COX7B2, ATP6V0A2, ATP6V0D1, NDUFV2, NDUFS1, NDUFA9, ATP6V0E2, COX10, ATP6V1H, ATP5F1B, ATP6V1B2, SDHC, SDHA, ATP6V1C1, NDUFV1, SDHB, COX7A1, NDUFA10, COX7B, NDUFA6, ATP6V1E1, CYC1, NDUFS3, ATP6V1G2, COX6C, NDUFB8, ATP6V0D2, COX8C, ATP6V1B1, ATP4A, COX7A2L, NDUFB4, NDUFS5, ATP5F1A, UQCRHL, ATP5MF, ATP5MC3, ATP5MC1, ATP5MC2, SDHD, NDUFAB1, COX17, NDUFA7, NDUFS8, NDUFA11, PPA1, ATP5PO, UQCRB, COX6B1, NDUFA3, UQCRQ, NDUFA2, NDUFB2, UQCRH, NDUFB6, ATP5PD, ATP5PB, NDUFA4L2, ATP5MG, NDUFB3, NDUFB1, NDUFS7, NDUFB7, ATP5F1D, NDUFV3, UQCR11, NDUFS4, COX7C, COX5A, ATP6V0E1, NDUFA8, NDUFB5**
REACTOME_RESPIRATORY_ELECTRON_TRANSPORT	68	-3.7	0	NDUFAF5, COQ10B, ETFDH, NDUFAF6, NDUFS2, LRPPRC, COQ10A, COX19, NDUFV2, NDUFAF7, NDUFS1, NDUFA9, SDHC, SDHA, SCO1, NDUFV1, SDHB, TACO1, NDUFA10, COX7B, NDUFA6, NUBPL, CYC1, NDUFS3, COX6C, NDUFB8, **COX7A2L, SURF1, COA1, NDUFB4, NDUFS5, COX18, NDUFA13, SDHD, TMEM186, NDUFAB1, NDUFA7, TMEM126B, NDUFAF4, NDUFS8, NDUFA11, UQCRB, CYCS, COX6B1, COX16, NDUFA3, UQCRQ, NDUFAF3, NDUFA2, NDUFB2, UQCRH, NDUFB6, NDUFB3, NDUFB1, NDUFS7, NDUFB7, NDUFAF2, NDUFV3, UQCR11, ETFA, TIMMDC1, NDUFS4, ECSIT, COX7C, COX5A, NDUFA8, NDUFAF1, NDUFB5**
REACTOME_THE_CITRIC_ACID_TCA_CYCLE_AND_RESPIRATORY_ELECTRON_TRANSPORT	119	-3.63	0	PDP1, SLC25A27, NDUFAF5, COQ10B, SLC25A14, DLST, CS, IDH3B, SLC16A1, ETFDH, IDH3G, NDUFAF6, MPC2, NDUFS2, PPARD, SLC16A8, PDK3, LRPPRC, D2HGDH, PDHA1, COQ10A, PDP2, SLC16A3, COX19, PDPR, LDHA, NDUFV2, NDUFAF7, UCP3, NDUFS1, NDUFA9, ATP5F1B, L2HGDH, SDHC, DLD, SDHA, SCO1, NDUFV1, SDHB, TACO1, NDUFA10, FH, SUCLG1, MPC1, COX7B, NDUFA6, NUBPL, ACO2, PDHX, CYC1, NDUFS3, COX6C, NDUFB8, MDH2, UCP1, **COX7A2L, SURF1, COA1, NDUFB4, NDUFS5, ATP5F1A, ATP5MF, NNT, COX18, IDH3A, NDUFA13, ATP5MC3, LDHB, ATP5MC1, ATP5MC2, FAHD1, SDHD, PDHB, TMEM186, SUCLA2, NDUFAB1, NDUFA7, TMEM126B, NDUFAF4, SUCLG2, ME2, NDUFS8, NDUFA11, ATP5PO, UQCRB, CYCS, DLAT, COX6B1, COX16, NDUFA3, UQCRQ, NDUFAF3, NDUFA2, OGDH, NDUFB2, UQCRH, GSTZ1, NDUFB6, ATP5PD, ATP5PB, ATP5MG, NDUFB3, NDUFB1, IDH2, NDUFS7, NDUFB7, NDUFAF2, ATP5F1D, NDUFV3, UQCR11, ETFA, TIMMDC1, NDUFS4, ECSIT, COX7C, COX5A, NDUFA8, NDUFAF1, NDUFB5**
REACTOME_MITOCHONDRIAL_TRANSLATION	52	-3.75	0	MRPL9, MTRF1L, MRPL55, MRPL10, MRPL22, MRPL38, PTCD3, TUFM, MRPL44, DAP3, OXA1L, TSFM, MRPL39, ERAL1, MRPL53, MRPS22, MRPL28, **MRPL14, MRPS27, MRPL51, MRPS12, MRPL18, MRPL3, MRPL50, MRPL16, MRRF, MRPL20, MRPL21, MRPL32, MRPL11, MRPL41, MRPL42, MRPL35, MRPL36, MRPS17, MRPS11, MRPL17, CHCHD1, MRPL54, MTFMT, GADD45GIP1, MRPL57, MRPS24, MRPL12, MRPL46, MRPL48, MRPS36, MRPS33, MRPL27, MRPL34, MRPS31, MTIF3**
HALLMARK_OXIDATIVE_PHOSPHORYLATION	140	-2.81	0	PDP1, SUPV3L1, CASP7, ACADVL, DLST, TIMM17A, CS, ATP6V0C, OPA1, IDH3B, AFG3L2, ETFDH, IDH3G, SLC25A3, NDUFS2, ISCU, GLUD1, MFN2, CYB5R3, LRPPRC, PDHA1, ISCA1, HCCS, ATP6V0B, HSPA9, LDHA, NDUFV2, BDH2, NDUFS1, NDUFA9, RETSAT, COX10, ATP6V1H, ALDH6A1, ATP5F1B, PMPCA, HTRA2, RHOT2, SDHC, DLD, ACADSB, OXA1L, ACAA2, TOMM70, HSD17B10, SDHA, POR, MTRR, ATP6V1C1, NDUFV1, SDHB, ABCB7, GOT2, FH, SUCLG1, PHYH, VDAC2, MPC1, COX7B, NDUFA6, ATP6V1E1, ACO2, PDHX, CYC1, NDUFS3, SLC25A5, ACAA1, COX6C, MRPS22, NDUFB8, MDH2, MAOB, **TOMM22, COX7A2L, SURF1, TIMM9, NDUFB4, ATP5F1A, SLC25A12, ATP5MF, NNT, IDH3A, ATP5MC3, LDHB, ATP5MC1, ATP5MC2, MRPS12, SDHD, PDHB, SUCLA2, NDUFAB1, COX17, NDUFA7, POLR2F, BCKDHA, TIMM10, NDUFS8, ACAT1, ATP5PO, SLC25A11, UQCRB, CYCS, DLAT, COX6B1, ECI1, NDUFA3, UQCRQ, NDUFA2, MRPL11, PRDX3, OGDH, MRPL35, NDUFB2, IDH1, UQCRH, MRPS11, SLC25A6, NDUFB6, ATP5PD, ATP5PB, ATP5MG, NDUFB3, NDUFB1, IDH2, NDUFS7, NDUFB7, ATP5F1D, ACADM, UQCR11, ETFA, SLC25A20, NDUFS4, TIMM13, COX7C, COX5A, ATP6V0E1, MRPL34, NDUFA8, TIMM8B, NDUFB5**
